# A unified platform for the rapid assembly of glutarimides for Cereblon E3 ligase modulatory drugs

**DOI:** 10.1038/s41467-026-74673-x

**Published:** 2026-06-21

**Authors:** David M. Whalley, Olivier Lorthioir, Niall A. Anderson, Erin Braybrooke, Susannah C. Coote, Sylvain Demanze, Holly L. Douglas, Okky Dwichandra Putra, Katie Proctor, Yuanyuan Si, Sam Staniland, Stephen Stokes, Alfie Woodhouse

**Affiliations:** 1 Oncology R&D, The Discovery Centre, AstraZeneca, Cambridge, UK; 2 Department of Chemistry, University of Bath, Claverton Down, Bath, UK; 3 Early Product Development and Manufacturing, Pharmaceutical Sciences R&D, AstraZeneca, Pepparedsleden 1, Mölndal, Sweden; 4 Biopharmaceuticals R&D, AstraZeneca, 35 Gatehouse Dr, Waltham, MA USA

**Keywords:** Drug discovery and development, Synthetic chemistry methodology, Organocatalysis

## Abstract

Glutarimide-containing Cereblon (CRBN) ligands are critical motifs for PROTACs, molecular glue degraders and next-generation Cereblon E3 ligase modulatory drugs (CELMoDs), which represent promising therapeutic modalities in targeted protein degradation. However, the multistep synthetic routes required to access glutarimide scaffolds continue to present formidable challenges for medicinal chemists, limiting rapid structure–activity relationship (SAR) exploration and late-stage diversification. To streamline access to these privileged motifs, modular and efficient methodologies are still highly desirable. Here, we report a unified organocatalytic synthesis platform for the rapid assembly of diverse glutarimide derivatives from readily available nitrogen heterocycles. Employing a sequence of phosphine-catalysed C–N bond formation, metal-free Giese addition and acid-mediated cyclisation, this approach provides high selectivity, broad functional group tolerance and operational simplicity under conditions amenable to both multigram synthesis and high-throughput parallel synthesis. Using this platform, we rapidly prepare CRBN binder libraries, access control analogues (for example, *N*‑alkylated glutarimides) and perform late‑stage functionalisation of bioactive molecules. This strategy could offer a transformative solution for the efficient and cost-effective synthesis of CRBN-targeted therapeutics and chemical biology probes, overcoming longstanding synthetic bottlenecks in the field.

## Abstract (web summary)

Glutarimide-containing CRBN ligands are central to targeted protein degradation but remain synthetically challenging. Here, the authors report a unified organocatalytic platform enabling rapid, selective, and scalable assembly of diverse glutarimides, facilitating library synthesis and late-stage functionalisation.

## Introduction

The clinical success of drugs such as thalidomide, lenalidomide, and pomalidomide has invigorated efforts to design next-generation protein degraders using the underlying mechanism of Cereblon E3-ligase (CRBN) recruitment^1–3^. These small molecules act as either molecular glues, inducing or stabilising interactions between CRBN and substrate proteins, or as key ligands within bifunctional architectures known as PROTACs (Proteolysis Targeting Chimeras)^4^. PROTACs that utilise CRBN chemically tether an E3-ligase-binding fragment to a ligand for a target protein, forming a degrader that directs disease-relevant proteins to CRBN for ubiquitination and subsequent proteolysis^5,6^. The success of Cereblon E3 Ligase Modulatory Drugs (CELMoDs) in oncology and immunology underscores the need for versatile platforms to access structurally diverse and potent CRBN binders.

**Fig. 1:**
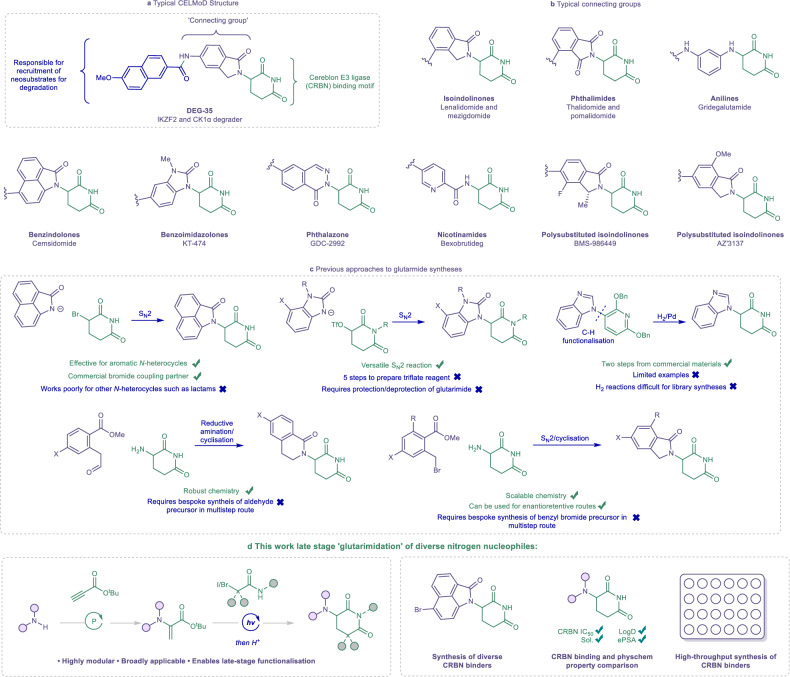
Late-stage ‘glutarimidation’ of diverse nitrogen nucleophiles. **a** DEG-35 as an example of a typical glutarimide-containing CELMoD or CRBN-dependent molecular glue degrader. **b** Typical connecting groups in molecular glue degraders/CELMoDs and PROTACs. **c** Previously explored approaches to incorporate the connecting heterocycle to the glutarimide scaffold. **d** Our late-stage organocatalytic ‘glutarimidation’ cascade with applications to CRBN binder synthesis.

At the heart of most CRBN-targeting molecules lies a glutarimide scaffold, a six-membered cyclic imide that binds to a small hydrophobic pocket (tri-Trp pocket) in the thalidomide-binding domain (TBD) of CRBN^7,8^. The amine nitrogen of the glutarimide is bound to the rest of the degrader structure via a ‘connecting group’ (Fig. 1a). Commonly employed connecting groups include isoindolinone (utilised in lenalidomide, the PROTAC vepdegestrant and the molecular glue degrader mezigdomide), phthalimides (used in thalidomide and pomalidomide) and anilines (such as in gridegalutamide) (Fig. 1b)^9^. In the pursuit of improved properties, designers have looked to a wide variety of architectures to constitute this connecting group, with structures like benzindolone, benzimidazolone and phthalazone featuring in a number of degrader scaffolds, as well as pyridyl amides and poly-substituted isoindolinones^10–15^.

However, synthetic limitations have historically hindered the exploration of novel glutarimide chemotypes^16^. Whilst Buchwald-Hartwig-inspired approaches to the synthesis of dihydrouracils^17^, aniline-linked glutarimides and C(sp^3^)-C(sp^2^)-linked glutarimides have been established^18–20^, there is a distinct lack of unified methods to access the broad diversity of *N*-heterocyclic glutarimides that modern protein degrader design demands. Practitioners in the field looking to compile a diverse library of CRBN-binders often must resort to a plethora of synthetic techniques (Fig. 1c). In a few cases, *N*-heterocycles can be installed using the nucleophilic displacement of a 3-bromo-glutarimide; however, these reactions can be low yielding, often requiring the use of pyrophoric bases, therefore making scale-up difficult and high-throughput chemistry challenging^21^. Alternatively, for the synthesis of the PROTAC KT-474, Zheng and co-workers opted for the nucleophilic displacement of an *N*-protected glutarimide triflate, prepared in a five-step sequence, delivering the final PROTAC warhead fragment in seven steps^11^. An alternative approach includes the functionalisation of a 2,6-bis(benzyloxy)pyridiyl precursor via a C-N bond formation, followed by reduction to the respective glutarimide scaffold. However, adoption of this method has been slow, potentially due to the reliance on hydrogenation chemistry^21,22^. For isoindolinones or other lactam-based heterocycles, *de-novo* ring synthesis is the most commonly employed strategy, whereby a central benzene ring is poly-functionalised in a multistep route before eventual lactamisation with 3-aminoglutarimide^23^.

In this work, we present a unified “glutarimidation” platform that converts readily available nitrogen heterocycles into glutarimide CRBN binders. The sequence merges phosphine‑catalysed α‑addition, metal‑free photocatalytic Giese coupling, and acid‑triggered cyclisation under amenable conditions. It shows broad functional‑group tolerance, delivers canonical and under‑represented connecting motifs and translates from multigram synthesis to high‑throughput formats.

## Results and discussion

### Multiparameter optimisation

We set out to address these challenges through the development of a general synthetic platform for *N*-heterocycle-linked glutarimides (Fig. 1d). Our reaction design converts commercial nitrogen-based nucleophiles into the corresponding *N*-substituted acrylate esters via α-addition to *tert*-butyl propiolate, furnishing competent Giese acceptors for coupling with α-haloacetamides. Subsequent acidification triggers cyclisation, delivering the fully elaborated CRBN-binding warhead. We aimed to achieve a very broad *N*-heterocycle scope, allowing medicinal chemists to attempt new CRBN-binder designs as well as rapidly construct canonical binders directly from the parent heterocycle. Furthermore, the reaction design should be capable of delivering gram-scale quantities of CRBN binders in an economical fashion for downstream in-vivo studies or further chemical manipulations. Likewise, translatability to high-throughput experimentation (HTE) would enable unparalleled synthetic efficiency, enabling rapid evaluation of CRBN binder libraries for next‑generation molecular glues and PROTACs.

**Fig. 2:**
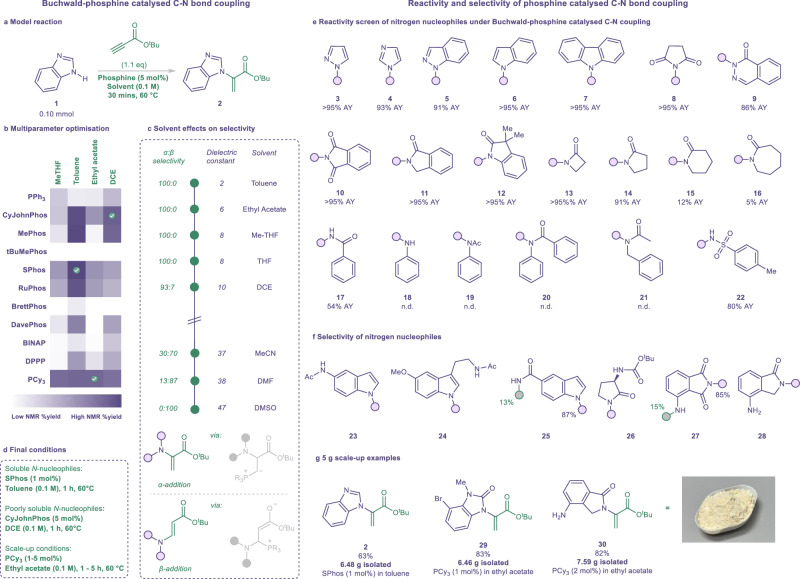
Buchwald-phosphine catalysed C-N bond forming reaction development. **a** Model reaction for the C-N coupling of *tert*-butylpropiolate and benzimidazole. **b** Phosphine and solvent multiparameter optimisation screen on the model reaction using 5 mol% phosphine. NMR yields given against a 1,2-dimethoxyethane internal standard. **c** Correlation between solvent dielectric constant and α:β selectivity of the reaction using SPhos 1 mol%, 60 °C, 30 min. **d** Optimised conditions for the C-N bond coupling reaction. **e** A reactivity screen for different nitrogen nucleophiles for the C-N bond coupling using CyJohnPhos (5 mol%) in DCE (0.1 M), 60 °C, 1 h; ^1^H NMR yields given against a 1,2-dimethoxyethane internal standard. AY = assay yield, n.d. = not detected **f** Trends in the reaction chemoselectivity using CyJohnPhos (5 mol%) in DCE (0.1 M), 60 °C, 1 h, with ratios determined via integration of the crude ^1^H NMR spectrum and/or COSY NMR. **g** C-N bond coupling on a five-gram scale. Reaction times: **2** = 1 h, **29** = 2 h, **30** = 5 h.

The first challenge to address was the synthesis of *N*-substituted acrylate esters via α-addition to *tert*-butyl propiolate that would be applicable to both multigram-scale up and HTE-plate format. Trost had previously shown that the addition of phthalimide and primary sulfonamides to alkynoate esters at 105 °C could deliver exclusive α-selectivity using a triphenylphosphine catalyst with acetic acid/acetate additives after eighteen hours^24^. While subsequent studies extended the scope to other simple azoles, conditions relied on high catalyst loadings of triphenylphosphine or on pyrophoric trialkylphosphines^25,26^. These limitations in phosphine loading, pyrophoricity, reaction time and temperature would make these strategies difficult to apply to our synthetic platform. However, we reasoned that the modern plethora of cross-coupling phosphine ligands could reveal a milder set of conditions, owing to their unique steric and electronic properties. To explore this hypothesis, we employed benzimidazole (**1**) as a model nucleophile with a *tert*-butyl propiolate acceptor (Fig. 2a), screening a range of commercial phosphines in four non-polar solvents (Fig. 2b). Using this approach, we uncovered that particular types of Buchwald phosphines (along with tricyclohexylphosphine) in certain non-polar solvents offered vastly superior reaction conditions, outperforming triphenylphosphine and bis-phosphines (such as BINAP and DPPP). The desired product (**2**) was obtained in near-quantitative yield, with exclusive α-selectivity, in under 1 hour with catalyst loadings as low as 1 mol%, an invaluable development that unlocked the reaction’s applicability to scale-up chemistry and HTE library synthesis. Furthermore, during initial screening, an intriguing solvent-dependent selectivity emerged: high dielectric constant solvents, such as dimethylsulfoxide (DMSO), completely inverted the selectivity to favour the β-addition product (Fig. 2c). Control reactions confirmed that this transformation remained phosphine-catalysed, representing a novel base-free Michael addition of weak nucleophiles to propiolate acceptors at modest temperature (see Supplementary Information for mechanistic discussion).

The optimised conditions employed low catalyst loadings, with either SPhos in toluene or CyJohnPhos in 1,2-dichloroethane (DCE) selected as general protocols for scope exploration and PCy_3_ in ethyl acetate for scale-up synthesis (Fig. 2d). The use of CyJohnPhos in DCE was particularly advantageous, accommodating substrates with lower solubility. The scope of coupling partners was further evaluated under these conditions (Fig. 2e). Acidic azoles including pyrazole (**3**), imidazole (**4**), indazole (**5**), indole (**6**), and carbazole (**7**), all underwent near-quantitative conversion on a 0.10 mmol scale (Fig. 2e). Coupling partners relevant to the synthesis of medicinally important glutarimides, such as succinimide (**8**), phthalazinone (**9**), thalidomide (**10**), isoindolinone (**11**), and oxindole (**12**), also proved highly efficient, consistently yielding products with exclusive α-selectivity. Notably, a correlation was observed with cyclic lactam substrates (**13–16**): decreasing ring size led to improved reaction conversion, likely owing to the respective nucleophilicity of the lactam nitrogens. In contrast, the reaction with a primary amide (**17**) resulted in modest yields, and substrates such as aniline (**18**), acetanilide (**19**), or benzanilide (**20**) or amide **21** did not afford any product. This selectivity could be strategically valuable when reacting to partners featuring multiple nitrogen-based functional groups. The protocol demonstrates high chemoselectivity across several model substrates (**23–28**, Fig. 2f), consistently favouring reactivity at indole, lactam and imide nitrogens in the presence of linear amides, carbamates or free anilines. Lastly, we tested the robustness of the methodology by conducting the C-N bond coupling reaction on a five-gram scale (Fig. 2g). The optimised conditions delivered over six grams of acrylate product **2** and likewise, benzimidazolone gave **29** in high yield using the tricyclohexylphosphine-catalysed conditions. 4-Aminoisoindolinone delivered over seven grams of **30**, selectively functionalising the lactam nitrogen, paving the way to a selective lenalidomide synthesis later in the substrate scope.

**Fig. 3:**
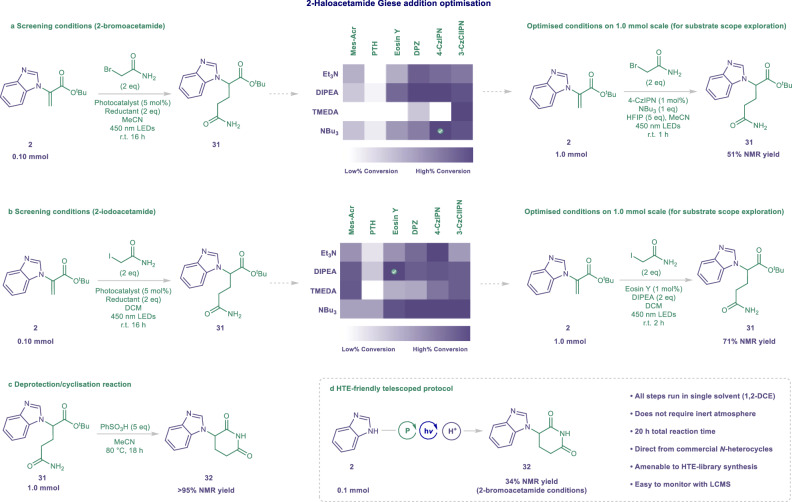
Giese addition/cyclisation cascade reaction development. **a** Screening conditions for the addition of 2-bromoacetamide to acceptor **2** and optimised reaction conditions thereof. **b** Screening conditions for the addition of 2-iodoacetamide to acceptor **2** and optimised reaction conditions thereof. **c** Deprotection/cyclisation reaction conditions of intermediate **31** to give product **32**. **d** HTE-friendly telescoped protocol for the conversion of **2** into **32**. ^1^H NMR yields given against a 1,2-dimethoxyethane internal standard. Mes-Acr = 9-mesityl-10-methylacridinium tetrafluoroborate; PTH = 10-phenylphenothiazine; DPZ = 5,6-bis(5-methoxythiophen-2-yl)pyrazine-2,3-dicarbonitrile; 4-CzIPN = 2,4,5,6-tetrakis(carbazol-9-yl)-1,3-dicyanobenzene; 3-CzClIPN = 2,4,6-tri(9H-carbazol-9-yl)-5-chloroisophthalonitrile. DIPEA = *N,N*-diisopropylethylamine; TMEDA = *N,N,N’,N’*-tetramethylethylenediamine; HFIP = (1,1,1,3,3,3-hexafluoro-2-propanol).

Complementing the mild organocatalysis in the phosphine-catalysed C-N bond formation, a high-throughput screen of metal-free Giese additions was initiated on benzimidazole substrate **2**. Despite previous examples of Giese additions being employed to yield unnatural amino acids and the ready availability of 2-bromo- and 2-iodoacetamide, syntheses of glutamine derivatives via this strategy remain rare^27^. Organic photocatalyst and tertiary amine reductant combinations were screened by irradiating at 450 nm before optimisation of the quantitative parameters was carried out. 1,2,3,5-Tetrakis(carbazol-9-yl)-4,6-dicyanobenzene (4CzIPN) with a tributylamine reductant and Eosin Y/diisopropylethylamine (DIPEA) gave superior conversion to the Giese product for 2-bromoacetamide and 2-iodoacetamide, respectively (Fig. 3a, b), giving rise to two sets of conditions, which were later found to be critical to encompass a broad range of acrylate substrates. Further optimisation facilitated reduction of the catalyst loading to 1 mol% and the reaction times to 1 or 2 h at a 1.0 mmol scale. Acid-mediated cyclisation of **31** could yield the fully elaborated glutarimide product (**32**) via a *tert*-butyl deprotection/dehydrative cascade (Fig. 3c)^28^. Alternatively, the Giese/cyclisation steps can be telescoped to give **32** directly from the acrylate intermediate. The optimisation campaign culminated in the demonstration that benzimidazole (**1**) can be converted directly into the bioactive glutarimide **32**, telescoping all three steps with no solvent-swaps in as little as twenty hours total reaction time, ideal for the HTE synthesis of glutarimide libraries (Fig. 3d).

### Scope of *N*-heterocycle glutarimidation

**Fig. 4:**
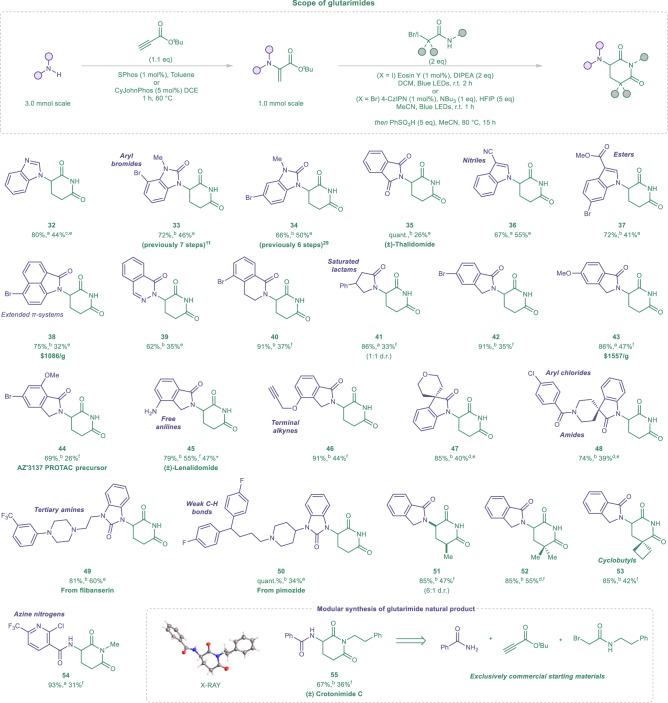
Scope of glutarimidation. **a** SPhos (1 mol%) in toluene (0.1 M), for 1 h at 60 °C; **b** CyJohnPhos (5 mol%) in 1,2-dichloroethane (0.1 M), for 1 h at 60 °C; **c** Isolated as the benzenesulfonic acid salt; **d** Required extended reaction times, see Supplementary Information; **e** 2-iodoacetamide (2 eq), Eosin Y (1 mol%), DIPEA (2 eq), in DCM for 2 h at r.t., 450 nm LEDs; **f** 2-bromoacetamide (2 eq), 4-CzIPN (1 mol%), NBu_3_ (1 eq), HFIP (5 eq) in MeCN for 1 h at r.t., 450 nm LEDs;^* 1^H NMR yield. Prices for **38** and **43** are from the Enamine Ltd catalogue.

With these optimised conditions established, we were then able to apply our approach to a broad range of nitrogen nucleophiles to explore the scope of the ‘glutarimidation’ sequence. Electing to use a two-step procedure allows for assessment of the efficiency of the phosphine catalysis and Giese/cyclisation cascade (Fig. 4) whilst remaining competitive in terms of step-count with previously published methods. Building on the success of **32**, we were then able to apply the same conditions to bromo-substituted *N*-methyl-benzimidazolinones **33** and **34**, targets that have initially been accessed in seven steps for the former (via the S_N_2 displacement of a triflate group, as discussed in Fig. 1d) or in six steps by AstraZeneca, respectively^11,29^. Gratifyingly, no dehalogenation was observed, presumably due to the single equivalent of tributylamine reductant employed in the protocol, favouring reduction of the more activated 2-bromoacetamide coupling partner^30^. Phthalimide could be engaged in the glutarimidation process, yielding (±)-thalidomide (**35**), whilst indoles **36** and **37** bearing useful functional handles such as nitriles and esters also reacted smoothly in the reaction.

Similarly, benzindolones proved to be compatible reaction partners (**38**), highlighting the method’s tolerance for extended π-systems. Benzindolone-based glutarimides have garnered interest within medicinal chemistry, especially for the development of degraders such as cemsidomide—an IKZF1/3 (Ikaros/Aiolos) degrader with molecular glue activity, as well as PROTACs targeting the previously “undruggable” BRD9 (bromodomain-containing protein 9)^10,31^. Phthalazone also performed smoothly, furnishing example **39**, and effectively incorporating the warhead found in Genentech’s heterobifunctional androgen receptor antagonist and degrader, GDC-2992^32^. Previous routes to this example have involved the *N*-alkylation of the parent heterocycle with a 2-bromoglutarate ester (synthesised in three steps), followed by cyclisation with sodium amide under cryogenic conditions, making scalability a key challenge^12^. With lactam-based connecting groups proving to be a popular choice for the design of protein degraders, pleasingly γ-lactam **41** could be easily synthesised, with a previous route to access this molecule via S_N_2 chemistry giving an isolated yield of 9%^33^, whilst relying on hazardous mixtures of sodium hydride and dimethylformamide^34^. Substituted isoindolinones **42–46** were also good substrates for this methodology, allowing application to both canonical degrader scaffolds and underrepresented motifs. For example, brominated isoindolinone (**42**) has served as a functionalisable warhead for the synthesis of protein degraders such as NVP-DKY709^35^, LYG-409^36^, and Eragidomide^37^, whereas **44** serves as the warhead in AstraZeneca’s androgen receptor PROTAC (AZ’3137)^14^. The marketed drug lenalidomide (**45**) was synthesised in three steps to aid purification, avoiding the hazardous nitro-reduction steps employed in previous routes^38^. Example **46** demonstrated the method’s tolerance for terminal alkynes, giving a product that contains an effective click-chemistry handle for the construction of novel PROTACs^39^. Pleasingly, we could employ unusual spirocyclic oxindoles in our glutarimidation cascade with tetrahydropyran- and piperidyl-spirocyclic oxindoles (**47** and **48**), affording unique, three-dimensional glutarimides.

We then sought to determine whether the glutarimidation process could be extended to late-stage functionalisation of structurally complex scaffolds. Notably, both flibanserin (a serotonin modulator) and pimozide (a neuroleptic drug) were efficiently transformed into their corresponding glutarimides (**49** and **50**), even in the presence of sensitive trialkylamine and anilinoamine groups^40,41^. Furthermore, we explored the modular nature of the method and used alternative 2-halo-acetamide partners, successfully generating a range of polysubstituted glutarimides (**51–53**) that would have been challenging to access from the corresponding non-natural amino acids^42,43^. For example, 5-methyl-glutarimide derivatives have previously been prepared by Egers and co-workers in a linear four-step sequence^44^.

Lastly, benzamides and heterobenzamides demonstrated robust reactivity under the optimised conditions^45^. Using 2-bromo-*N*-methylacetamide as the coupling partner enabled the direct synthesis of **54**. *N*-methylated glutarimides have been widely employed as controls in degradation assays, and this method provides rapid access to *N*-alkylated glutarimides, complementing the preparation of their unsubstituted analogues. This approach was further exemplified by the multicomponent synthesis of (±)-crotonimide C (**55**), a member of the family of glutarimide-containing natural products isolated from *Croton alienus*^46^. Significantly, the entire synthetic sequence relied solely on commercially available materials, and the structure of the final product was unequivocally confirmed by single-crystal X-ray analysis.

We next evaluated the CRBN binding affinities of the examples prepared above as well as their relevant physicochemical properties (Table 1). Pleasingly, the broad scope of synthesised glutarimides was mirrored by a wide breadth of properties across examples **32–55**, providing both new degrader structures as well as ways to finely tune their properties. For instance, a warhead based around benzimidazole (example **32**) could provide improved solubility and lower the LogD of a given degrader design whilst retaining excellent binding to CRBN. Conversely, more polar degraders could benefit from a benzindolone-based warhead, offering comparable LLE to benzimidazole whilst providing additional lipophilicity (LogD = 2.0). Interestingly, underrepresented motifs such as indole (**36** and **37**), γ-lactam (**41**) and spirocycles (**47–48**) were all competent CRBN binders. We were encouraged that late-stage functionalisation examples **49** and **50** bound to CRBN and occupied drug-relevant LogD space, highlighting a practical route to rapidly generate fully elaborated libraries for screening new CELMoD activity. As expected, substitution on the glutarimide ring itself was not tolerated. Nonetheless, these atypical motifs provide a means to incorporate glutarimide-like scaffolds into programmes outside protein degradation where avoidance of CRBN binding is desirable, with examples including novel tuberculosis antibiotics^47^.

**Table 1:** CRBN binding and physicochemical data for glutarimides in Fig. 4

Compound #	ePSA (Å^2^)	LogD (pH 7.4)	Solubility (µM)	HLM CL_int_ (µL/min/mg)	Caco-2 intrinsic permeability A to B Papp (×10^–6^ cm/s)	TR-FRET CRBN pIC_50_	LLE
**32**	93	0.2	480	<3.0	8.8	6.6	6.4
**33**	60	1.5	50	<3.0	53	7.9	6.4
**34**	61	1.2	52	<3.0	58	7.9	6.7
**35**	76	0.5	180	<3.0	98	6.7	6.2
**36**	97	1.1	283	7.5	51	6.4	5.3
**37**	95	2.2	320	21	63	6.8	4.6
**38**	95	2.3	2	<3.0	70	8.7	6.4
**39**	83	0.3	240	<3.0	72	6.4	6.1
**40**	88	1.4	160	<3.0	53	6.3	4.9
**41**	82	0.5	610	<3.0	48	6.2	5.7
**42**	91	1.0	72	<3.0	67	7.0	6.0
**43**	90	0.3	160	<3.0	52	7.1	6.8
**44**	97	0.7	140	<3.0	35	6.8	6.1
**45**	105	-0.4	680	<3.0	2.6	6.8	7.2
**46**	90	0.6	590	<3.0	42	8.0	7.4
**47**	80	0.6	650	<3.0	45	6.2	5.6
**48**	95	2.1	7	11	53	6.5	4.4
**49**	91	2.9	94	>300	65	8.0	5.1
**50**	101	3.6	2	47	-	7.1	3.5
**51 (cis)**	84	0.6	850	<3.0	57	<5	-
**51 (trans)**	83	0.5	710	<3.0	72	<5	-
**52**	79	0.9	620	<3.0	85	<5	-
**53**	84	1.2	120	<3.0	64	<5	-
**54**	72	1.1	320	<3.0	64	<5	-
**55**	77	2.6	6	24	77	<5	-

ePSA = experimental polar surface area; HLM = human liver microsomes; HLM CL_int_ = human liver microsomes; intrinsic clearance; TR-FRET = time-resolved Förster resonance energy transfer; LLE = ligand-lipophilicity efficiency (calculated as pIC_50_ – LogD). See Supplementary Information for further assay details.

### Multigram-scale glutarimidation

**Fig. 5:**
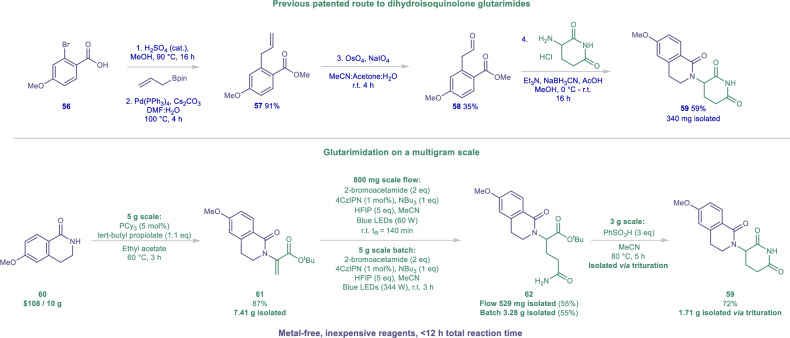
Comparison of a patented route to a dihydroisoquinolone glutarimide to this methodology on a multigram scale. Price for **60** from Combi-Blocks, Ltd.

For an evaluation of the glutarimidation methodology on a multigram scale, we focused on dihydroisoquinolone-glutarimides as targets, owing to the scarcity of routes to this motif. One previous patent to a dihydroisoquinolone-based glutarimide bearing a methoxy group involved a four-step sequence starting from benzoic acid **56**, employing classical Fischer esterification and palladium-catalysed Suzuki coupling with allylboronic acid pinacol ester to give intermediate **57** (Fig. 5)^48^. Osmium-catalysed Lemieux–Johnson oxidation furnished aldehyde **58**, which underwent a reductive amination/cyclisation cascade to provide **59** in 19% overall yield across four steps, with three chromatographic purifications and a forty-hour total reaction time. In contrast, we were able to initiate a multigram-scale synthesis starting from the inexpensive, commercially available 6-methoxy-3,4-dihydroisoquinolin-1-one (**60**), which reacted smoothly in the α-addition to *tert*-butyl propiolate via slow addition of tricyclohexylphosphine over 3 h, using a sustainable solvent, yielding more than 7 g of **61**. The feasibility of scaling the glutamine synthesis was then evaluated in both batch and flow. A flow setup using a 40 W LED source achieved excellent conversion to **62** (55% isolated yield), albeit at extended residence times. Carrying out the Giese addition in a Lucent360™ 700 mL water-cooled batch reactor delivered over three grams of material in three hours (see Supplementary Information for details) in identical yield. Subsequent acid-mediated cyclisation cleanly delivered the final glutarimide, allowing **59** to be isolated via chromatography-free trituration, yielding the product in three steps and 34% overall yield. Notably, the route also negates the need for precious metal catalysts, including highly toxic osmium tetroxide.

### Glutarimidation for advanced intermediate access for SAR mapping

**Fig. 6:**
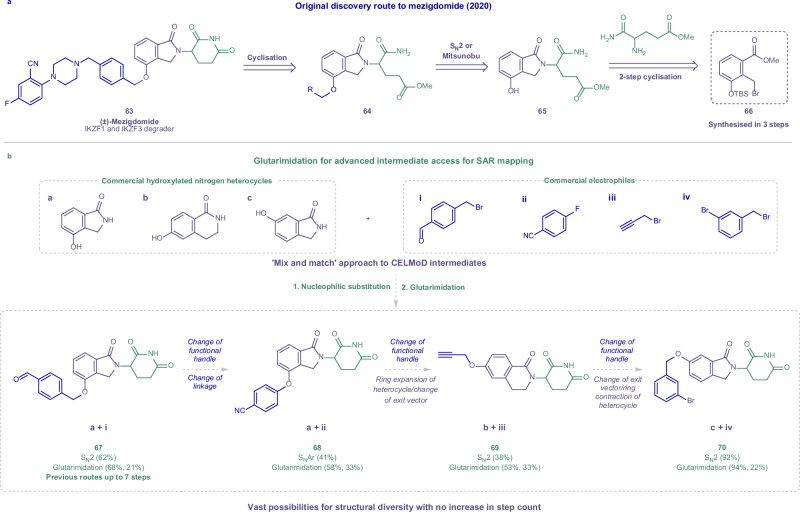
Application of the glutarimidation process to the construction of late-stage intermediates for the synthesis and optimisation of CRBN-dependent molecular glue degraders. **a** The original discovery route to mezigdomide (2020). **b** Application of this methodology for the synthesis of advanced intermediates for SAR mapping. To illustrate combinatorial breadth, the set consists of three nitrogen heterocycles and four electrophiles. In principle, this matrix yields 12 pairwise combinations; the products showcase four of these, underscoring the breadth of accessible glutarimide derivatives.

The next application of glutarimidation we sought to explore was the synthesis of late-stage, diversity-oriented building blocks for the discovery and optimisation of new molecular glue degraders. For the SAR exploration of mezigdomide (**63**), Hansen and co-workers developed a strategy in which the glutarimide could be derived from linear precursor **64**, with diversity introduced early on in this synthesis (via nucleophilic substitution or Mitsunobu chemistry on intermediate **65**, see Fig. 6a)^23^. The isoindolinone was synthesised in two steps using a glutamine derivative and **66**, which in turn was accessed in a further three steps. We reasoned that our glutarimidation strategy could be used to expedite SAR studies by enabling the synthesis of advanced intermediates for CELMoD. To achieve this, a pool of analogous hydroxylated benzolactam heterocycles (varying in ring size and exit vectors) could undergo S_N_2 or S_N_Ar reactions with commercial electrophiles via a convenient “mix-and-match” approach and then undergo the glutarimidation cascade to yield diversity-oriented building blocks bearing useful synthetic handles (Fig. 6b).

For example, **67** bearing a benzaldehyde moiety could enable the expedient construction of mezigdomide-like analogues, whereas **68** demonstrates a linker switch to a biaryl ether with a pendant nitrile. Likewise, **69** represents a ring-expanded variant with an alternative exit vector and a potential “click” handle, while **70** offers a distinct exit vector alongside a cross-coupling handle. Pertinent to these examples, a multitude of functionalisation reactions in the presence of glutarimides have been reported, including nitrile reductions^37^, click chemistry^49^, and transition metal cross-couplings^50^. Importantly, glutarimidation allows rapid exploration of molecular glue degrader analogues with no increase in step count from one analogue to the next, thereby streamlining SAR while preserving operational simplicity.

### High-throughput library synthesis

We ultimately translated the methodology into a high-throughput platform for the rapid synthesis of CRBN binder libraries. This workflow allows medicinal chemists to convert nitrogen-heterocycle libraries into diverse binder arrays with minimal route design, replacing bespoke multistep syntheses with streamlined, parallel execution. By accelerating make–test cycles, the platform delivers binding and physicochemical data at speed, enabling faster prioritisation and design iteration.

**Fig. 7:**
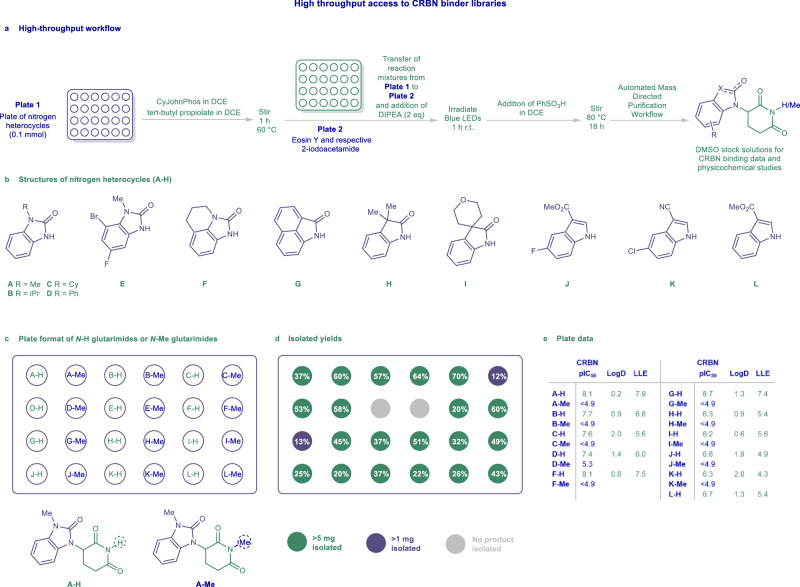
Glutarimidation applied to a high throughput experimentation platform. **a** Conditions: CyJohnPhos (5 mol%), 1.1 eq *tert*-butylpropiolate, DCE (0.1 M). Eosin Y (1 mol%), DIPEA (2 eq), 2-iodoacetamide (2 eq) or 2-*N*-methyl-iodoacetamide (2 eq), benzenesulfonic acid (3 eq) in DCE (1.5 M); **b** Structures of nitrogen heterocycles employed in the high-throughput platform; **c** Format of the plate with A-L indicating the species of nitrogen heterocycle and ‘H’ or ‘Me’ indicating 2-iodoacetamide or 2-*N*-methyl-iodoacetamide coupling partner used; **d** Isolated yield of the glutarimide product; **e** CRBN pIC_50_ binding data given in plate format and active glutarimides A-H to L-H tabulated alongside LogD and ligand-lipophilicity efficiency (LLE) where LLE = pIC_50_ – LogD.

We established a streamlined, telescoped workflow (Fig. 7a). A 24-vial plate (Plate 1) was charged with a library of twelve heterocycles that were paired with either 2-iodoacetamide or *N*-Me-2-iodoacetamide to give rise to active CRBN binders or their respective negative control. The heterocycle library included a variety of *N*-substituted benzoimidazones for matched-pair comparison, as well as a benzoindolone, several benzimidazoles, and three substituted indoles (Fig. 7b).

Solutions of CyJohnPhos (5 mol%) and *tert*-butyl propiolate were added, and the vials were stirred at 60 °C for 1 hour. In parallel, a second plate (Plate 2) was charged with Eosin Y (1 mol%) and either 2-iodoacetamide or *N*-Me-2-iodoacetamide, dispensed using a robotic powder handler. Reaction solutions from Plate 1 were then transferred to Plate 2, DIPEA was added, and each vial was irradiated for 1 hour in a carousel photoreactor (Lucent360™). Notably, the photoreactions proceeded unimpeded by residual phosphine, enabling preparation of a plate of linear glutamines in only 2 hours, building on our previous experience in merging phosphine catalysis and photocatalysis for the rapid construction of medicinally relevant compounds^51^. Subsequently, a concentrated solution of benzenesulfonic acid in DCE was added, and the plate was heated overnight to effect cyclisation. The final glutarimide products were isolated via automated mass-directed purification, giving both the free *N*H-glutarimide and the respective *N*Me negative control (Fig. 7c). Pleasingly, 22 out of 24 glutarimides (92% success rate) were isolated with suitable purity for determining CRBN IC₅₀ values and LogD measurements (Fig. 7d, e). This high-throughput glutarimidation, enabled by robotic reagent dispensing and a carousel photoreactor, powerfully compresses route design, synthesis and data generation into a single, telescoped workflow that has not been reported previously. Previous approaches to the high-throughput construction of glutarimide libraries generally employ a fixed, functionalised glutarimide containing fragment that preclude diversity of the connecting group and warhead itself^52–57^. A key limitation of the workflow to address in future iterations is the need to develop strategies for incorporating highly polar heterocycles, which have so far reacted poorly. Likewise, future HTE methods might aim to replace the chlorinated solvent with a more sustainable alternative, reduce the reaction time of the acid-mediated cyclisation, or combine the method with direct-to-biology techniques.

In conclusion, this work presents a robust organocatalytic synthesis platform enabling the rapid assembly of glutarimides, a privileged motif central to CELMoDs, molecular glues and PROTACs. The methodology supports parallel library synthesis in both a scalable and high-throughput format, combining operational simplicity with broad substrate scope. The platform accommodates electronically diverse heterocycles, canonical and underrepresented motifs, including benzindolones, phthalazones, oxindoles, and advanced cyclic lactams and enables the late-stage functionalisation of complex pharmaceutical scaffolds, in quantities and purity suitable for systematic biological and physicochemical evaluation. Notably, this approach overcomes previous synthetic bottlenecks, avoiding multistep route scouting and facilitating the construction of focused libraries for structure–activity relationship (SAR) studies, hit-to-lead optimisation, and the design of novel CRBN-targeted degraders. The ability to assemble glutarimide derivatives from readily available building blocks significantly expands the chemical space for targeted protein degradation, offering new approaches to previously undruggable proteins. Taken together, this platform represents a promising opportunity to accelerate the discovery and optimisation of Cereblon E3 ligase modulatory drugs and chemical probes, paving the way for next-generation therapeutics in protein degradation.

## Methods

### Phosphine catalysed C-N coupling (conditions A)

A round-bottom flask was charged with a stirrer bar, SPhos (12 mg, 0.03 mmol, 1 mol%) and the respective nitrogen nucleophile (3.0 mmol, 1.0 eq). Anhydrous toluene (30 mL) was added and a reflux condenser was fitted to the flask. The reaction mixture was then heated to 60 °C and tert-butyl propiolate (0.453 mL, 3.30 mmol, 1.1 eq) was added slowly. The suspension was then stirred at 60 °C for 15 mins to 1 h, monitored by LCMS. The reaction mixture was then concentrated under reduced pressure and the reaction mixture was purified directly by flash column chromatography, 0–100% ethyl acetate in heptane. The pure fractions were evaporated to dryness to afford the acrylate products.

### Phosphine catalysed C-N coupling (conditions B)

A round-bottom flask was charged with a stirrer bar, CyJohnPhos (53 mg, 0.15 mmol, 5 mol%) and the respective nitrogen nucleophile (3.0 mmol, 1.0 eq). Anhydrous 1,2-dichloroethane (30 mL) was added and a reflux condenser was fitted to the flask. The reaction mixture was then heated to 60 °C, and tert-butyl propiolate (0.453 mL, 3.30 mmol, 1.1 eq) was added slowly. The suspension was then stirred at 60 °C for 1 h, monitored by LCMS. The reaction mixture was then concentrated under reduced pressure and the reaction mixture was purified directly by flash column chromatography, 0–100% ethyl acetate in heptane. The pure fractions were evaporated to dryness to afford the acrylate products.

### Giese addition/acid-catalysed cyclisation (Conditions A)

General Procedure C: A 40 mL septum capped vial was charged with a stirrer bar, the respective acrylate (1.0 mmol, 1.0 eq), iodoacetamide (2.0 mmol, 2 eq) and Eosin Y (0.01 mmol, 1 mol%). The vial underwent three vacuum/nitrogen cycles, then anhydrous dichloromethane (10 mL) and DIPEA (2.0 mmol, 2 eq) were added under nitrogen. The vial was then placed in a Lucent360^TM^ photoreactor, with water-cooling set to room temperature and irradiated with 5 × 450 nm LEDs set to 75% intensity for 2 hours. After the reaction was complete (LCMS), the solvent was removed under reduced pressure then the residue was dissolved in anhydrous acetonitrile (0.1 M) and transferred to a three-neck round bottom flask. Benzenesulfonic acid (5.0 mmol, 5.0 eq) was then added and the flask was fitted with a thermometer and reflux condenser. The reaction mixture was heated to reflux (measuring internal temperature) overnight (c.a. 18 h).

### Giese addition/acid catalysed cyclisation (Conditions B)

A 40 mL septum capped vial was charged with a stirrer bar, the respective acrylate (1.0 mmol, 1.0 eq), 2-bromoacetamide (2 mmol, 2 eq) and 4-CzIPN (0.01 mmol, 1 mol%). The vial underwent three vacuum/nitrogen cycles, then anhydrous MeCN (0.1 M), HFIP (5.0 mmol, 5 eq) and tributylamine (1.0 mmol, 1.0 eq) were added under nitrogen. The vial was then placed in a Lucent360^TM^ photoreactor, with water-cooling set to room temperature and irradiated with 5 × 450 nm LEDs set to 75% intensity for 1 h. After the reaction was complete (LCMS), the reaction mixture was transferred to a 50 mL round-bottom flask and benzenesulfonic acid (5.0 mmol, 5.0 eq) was added and the flask was fitted with a thermometer and reflux condenser. The reaction mixture was heated to reflux (measuring internal temperature) overnight (c.a. 18 h).

### General software information

NMR data were measured using TopSpin v4.41 and IconNMR v6.2.1 and analysed using MestReNova. UPLC-MS data were collected and analysed using MassLynx v4. Collection and refinement of X-ray diffraction data and refinement were performed with CrysAlisPro 1.171.42.35a, ShelXT, Olex2, and ShelXL. Mercury 2024.3.0 and MOE 2022.02 were used to visualise X-ray structures.

### Reporting summary

Further information on research design is available in the Nature Portfolio Reporting Summary linked to this article.

## Supplementary information

Supplementary Information

Peer Review file

Reporting Summary

## Data Availability

The authors declare that the data supporting the findings of this study are available within the paper and its Supplementary Information files. Should any raw data files be needed in another format they are available from the corresponding author upon request. Crystallographic data for compound 55 has been deposited at the Cambridge Crystallographic Data Centre, under deposition number CCDC 2519893.
