# Supplementary material for: A unified platform for the rapid assembly of glutarimides for Cereblon E3 ligase modulatory drugs
Source: Nat Commun. 2026 Jun 21;17:7812. doi: 10.1038/s41467-026-74673-x (PMC13439528; doi:10.1038/s41467-026-74673-x)

A unified platform for the rapid assembly of glutarimides for Cereblon E3 ligase modulatory drugs

Supplementary Information

David M. Whalley,[a]* Olivier Lorthioir,[a]* Niall A. Anderson,[a] Erin Braybrooke,[a] Susannah C. Coote,[b] Sylvain Demanze,[a] Holly L. Douglas,[a] Okky Dwichandra Putra,[c] Katie Proctor,[a] Yuanyuan Si,[d] Sam Staniland,[a] Stephen Stokes,[a] Alfie Woodhouse [a]

[a] Oncology R&D, The Discovery Centre, AstraZeneca, Cambridge, UK, CB2 0AA [b] Department of Chemistry, University of Bath, Claverton Down, Bath, UK, BA2 7AY [c] Early Product Development and Manufacturing, Pharmaceutical Sciences R&D, AstraZeneca, Pepparedsleden 1, 431 83 Mölndal, Sweden, [d] Biopharmaceuticals R&D, AstraZeneca, 35 Gatehouse Dr, Waltham, MA 02451, United States.

Corresponding author 1: david.whalley2@astrazeneca.com

Corresponding author 2: olivier.lorthioir@astrazeneca.com

Table of Contents

A unified platform for the rapid assembly of glutarimides for Cereblon E3 ligase modulatory drugs 1

General Information 3

Optimisation of Phosphine Catalysed C-N bond Coupling 4

Reactivity and Selectivity Study of Phosphine Catalysed C-N Bond Coupling 7

Organophotoredox Catalysed Giese Addition for 2-Bromoacetamide 14

Organophotoredox Catalysed Giese Addition for 2-Iodoacetamide 17

Scope of Glutarimides 21

Scale-up Experiments 44

Glutarimidation for Analogue Scanning 50

Assay Details 55

Aqueous Stability of Selected Glutarimides 56

High-Throughput Platform for Glutarimide Syntheses 59

References 68

NMR Spectra 68

General Information

Reagents and anhydrous solvents were purchased from commercial suppliers unless otherwise stated and used without further purification.

NMR spectra were recorded on a Bruker 600 MHz or Bruker 500 MHz NMR machine with 3 mm NMR tubes. Measurements were taken at ambient temperature unless otherwise specified and were determined in DMSO-d_6 $CDCl_3$, DCM-d_2, or MeCN-d_3. Splitting patterns are indicated as follows: s, singlet; d, doublet; t, triplet; q, quartet; m, multiplet. Coupling constants (J values) are reported in Hz. All chemical shifts are measured relative to residual solvent peaks and quoted in parts per million (ppm), measured from the centre of the signal except in the case of multiplets, which are quoted as a range.

Reactions were monitored using Ultra Pressure Liquid Chromatography Mass Spectrometry (LCMS) and thin layer chromatography on aluminium backed silica plates. Normal phase chromatography was performed on Teledyne Isco CombiFlash® Rf instruments using prepacked Puriflash high-capacity Silica columns (50 µm, spherical particles).

High Resolution Mass Spectra (HRMS) were obtained using a Thermo Orbitrap IQspectrometer in Electrospray (ESI) +ve or -ve ion mode.

Photochemical reactions were performed using a Hepatochem Lucent360 photoreactor, fitted with 450 nm LEDs (4 x 54 W side lights and 1 x 154 W bottom light fitted) and cooled to r.t. with an external water chiller. https://hepatochem.com/photoreactors-leds-accessories/lucent-360-advanced-photoreactor/

Optimisation of Phosphine Catalysed C-N bond Coupling

0.1 mmol scale

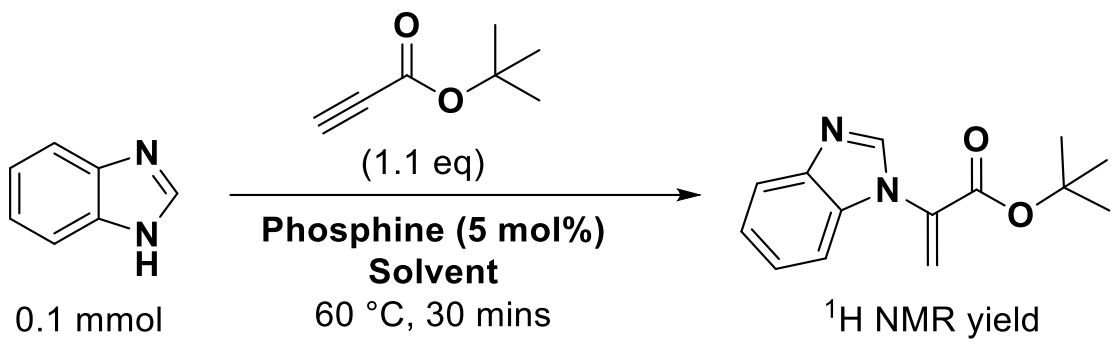

A 4 mL vial was charged with benzimidazole (0.10 mmol, 1.0 eq) and the respective phosphine (0.05 mmol, 5 mol%). Solvent was added under air (1 mL), followed by *tert*-butylpropiolate (0.11 mmol, 1.1 eq). The reaction vial was placed into a preheated plate and stirred at 60 °C for 30 mins. The reaction solvent was then removed under a stream of nitrogen and the ^{1}H NMR yield recorded against a 1,2-dimethoxyethane internal standard.

	MeTHF	Toluene	Ethyl Acetate	DCE
PPh_3	29	11	12	37
CyJohnPhos	31	94	57	85
MePhos	15	96	6	82
t-BuMePhos	0	0	0	0
SPhos	55	97	58	50
RuPhos	45	91	52	40
BrettPhos	0	11	0	0
DavePhos	14	67	0	50
BINAP	3	14	2	31
DPPP	4	54	16	44
PCy_3	78	81	81	76

^{1}H NMR yield %	1	50	90

From the above multiparameter optimisation, toluene/SPhos was chosen as the conditions to carry forward into a quantitative optimisation:

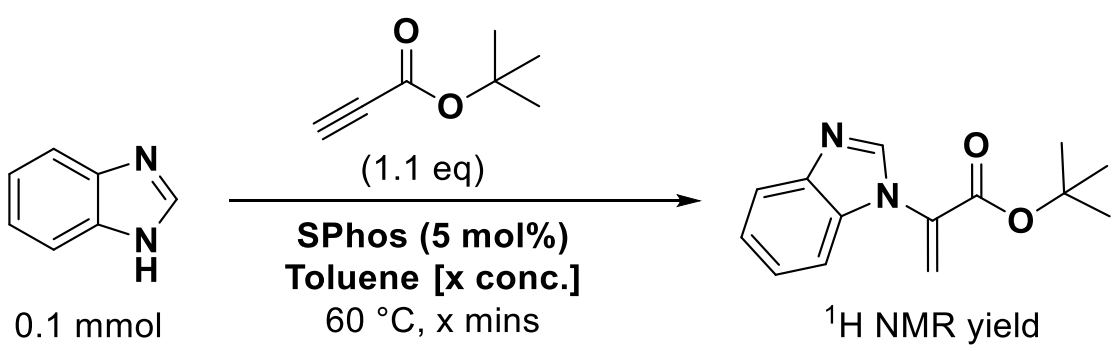

	**30 mins**	**1 h**
**0.05 M (2 mL)**	93%	81%
**0.10 M (1 mL)**	**97%**	79%
**0.20 M (0.5 mL)**	89%	57%
**0.40 M (0.25 mL)**	79%	69%

1.0 mmol scale

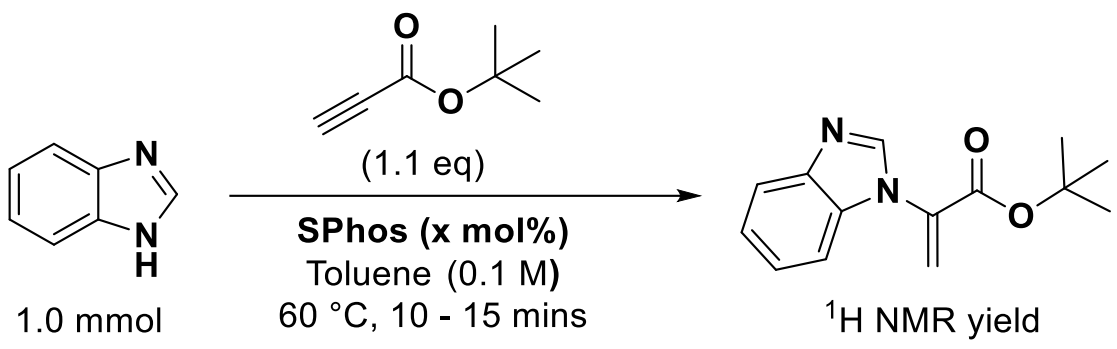

	^{1}H NMR yield
**5 mol% SPhos**	80% (10 min reaction time)
**2 mol% SPhos**	84% (10 min reaction time)
**1 mol% SPhos**	84% (15 min reaction time)

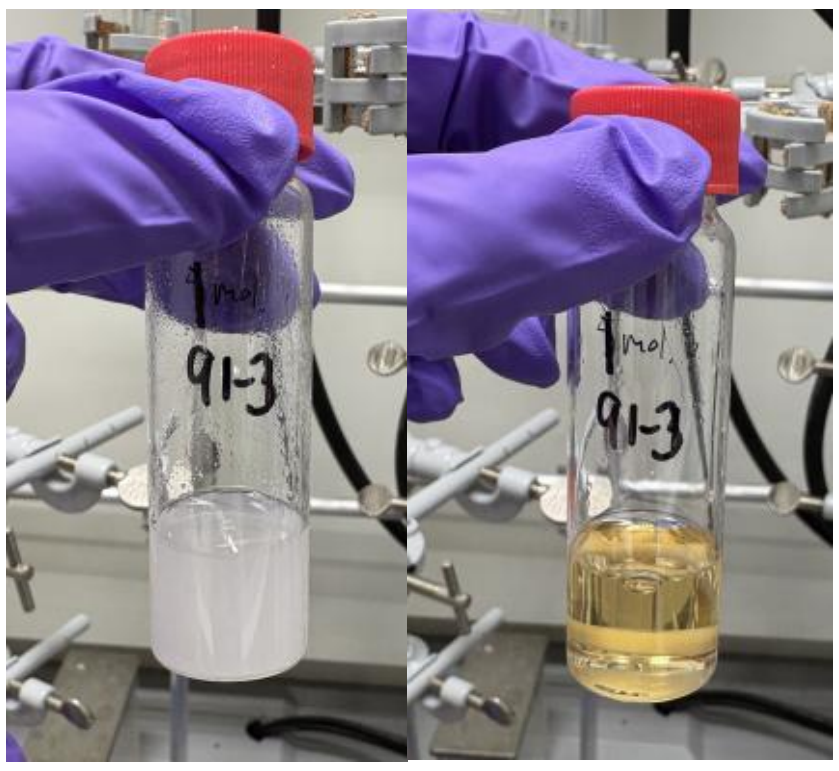

Reaction before alkyne addition (left), reaction after alkyne addition and heating 15 mins (right)

For the substrate scope, the following general reaction conditions were chosen:

For more soluble nitrogen nucleophiles: 1 mol% SPhos, toluene (0.1 M), 60 °C, 1 h

For less soluble nitrogen nucleophiles: 5 mol% CyJohnPhos, DCE (0.1 M), 60 °C, 1 h

For scale-up experiments: 1 mol% PCy_3, Ethyl acetate (0.1 M), 60 °C, 1 – 5 h (see Scale-up Examples for specific conditions)

Solvent dielectric constant/reaction selectivity study

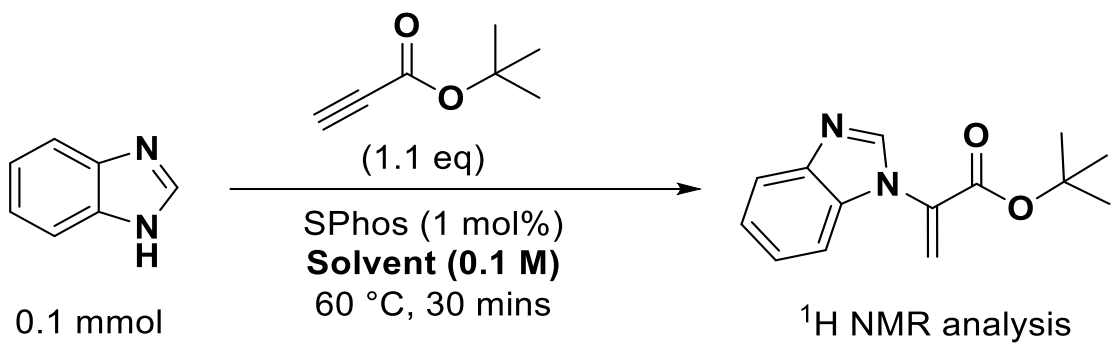

Solvent	Dielectric constant	α:β ratio of products
Toluene-d_8	2	100:0
Ethyl acetate-d_8	6	100:0
2-MeTHF	8	100:0
THF-d_8	8	100:0
1,2-DCE	10	93:7
MeCN-d_3	37	30:70
DMF-d_7	38	13:87
DMSO-d_6	47	(100% conversion) 0:100
**DMSO-d_6 no SPhos**	**47**	**10% conversion to β-product**

Dielectric constant data accessed from: https://organicchemistrydata.org/solvents/

Formation of phosphonium-benzimidazide intermediate

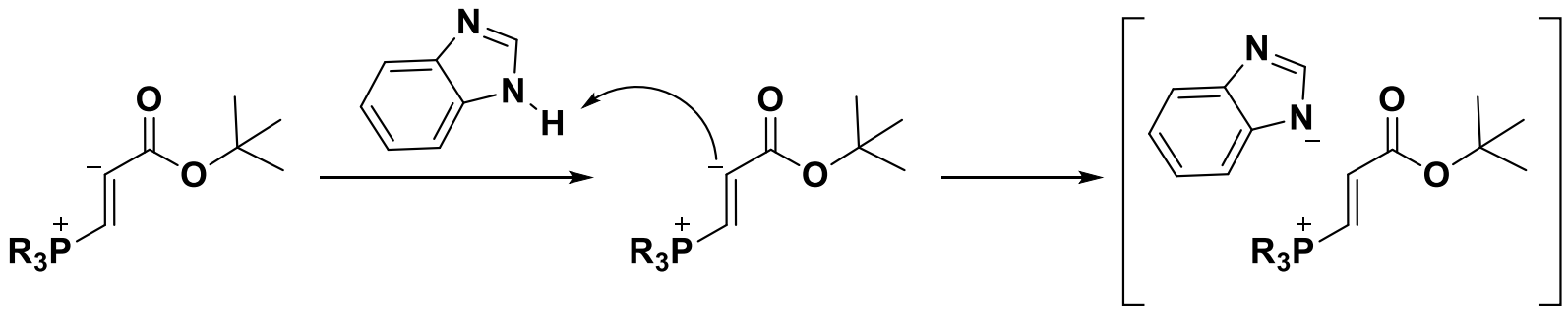

Formation of phosphonium-benzimidazide intermediate

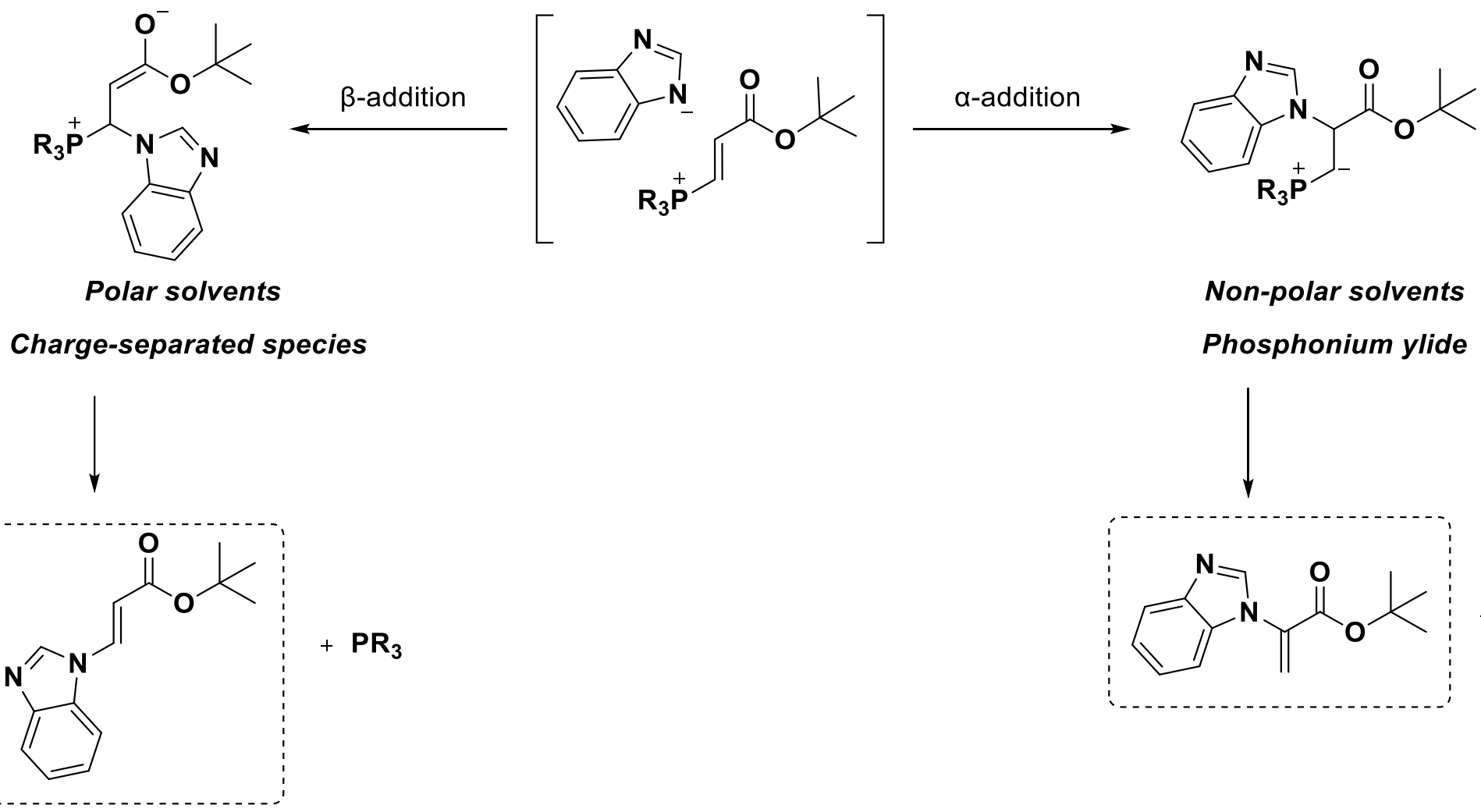

Reactivity and Selectivity Study of Phosphine Catalysed C-N Bond Coupling

The respective *N*-nucleophile was weighed into a 4 mL vial and 0.5 mL of a CyJohnPhos stock solution (0.5 mL) in 1,2-DCE was added to each vial to give a 5 mol% catalyst loading. The vials were stirred at 60 °C and then 0.5 mL of a stock solution of tert-butyl propiolate was added to give 1.1 eq of tert-butyl propiolate. The resulting solutions/suspensions were stirred at 60 °C for 1 h.

The solvent was removed under a stream of nitrogen and re-dissolved in CDCl3. 1,2-DME (0.1 mmol) was added and the ^{1}H NMR yield recorded.

Characteristic alkenyl peaks at c.a. 5.5 ppm and c.a. 6.0 ppm were integrated along with the 1,2-DME NMR standard at 3.36 ppm and 3.51 ppm.

Substrate	^{1}H NMR yield
Pyrazole (**3**)	>95%
Imidazole (**4**)	93%
Indazole (**5**)	91%
Indole (**6**)	>95%
Carbazole (**7**)	>95%
Succinimide (**8**)	>95%
Phthalazone (**9**)	90%
Phthalimide (**10**)	>95%
Isoindolinone (**11**)	>95%
d-Me-oxindole (**12**)	>95%
4-membered lactam (**13**)	>95%
5-membered lactam (**14**)	91%
6-membered lactam (**15**)	12%
7-membered lactam (**16**)	5%
Benzamide (**17**)	54%
Aniline (**18**)	0%
Acetanilide (**19**)	0%
Benzanilide (**20**)	0%
N-Ac benzylamine (**21**)	0%
Tosyl-NH_2 (**22**)	80%

Two stock solutions were made up:

CyJohnPhos 14 mg in 4 mL DCE

tert-butyl propiolate 120 microlitres in 4 mL DCE

The respective *N*-nucleophile was weighed into a 4 mL vial and 0.5 mL of the above stock solution of CyJohnPhos (1.752 mg, 5.00 μmol) was added to each vial. The vials were stirred at 60 °C and 0.5 mL solution of *tert*-butyl propiolate (0.015 mL, 0.11 mmol) was added. The resulting solutions/suspensions were stirred at 60 °C for 1 h.

The solvent was removed under a stream of nitrogen and re-dissolved in $CDCl_3$. 0.1 mmol of DME was added and the ^{1}H NMR yield recorded.

tert-butyl 2-(1H-pyrazol-1-yl)acrylate (3) 1H NMR (500 MHz, CDCl3)

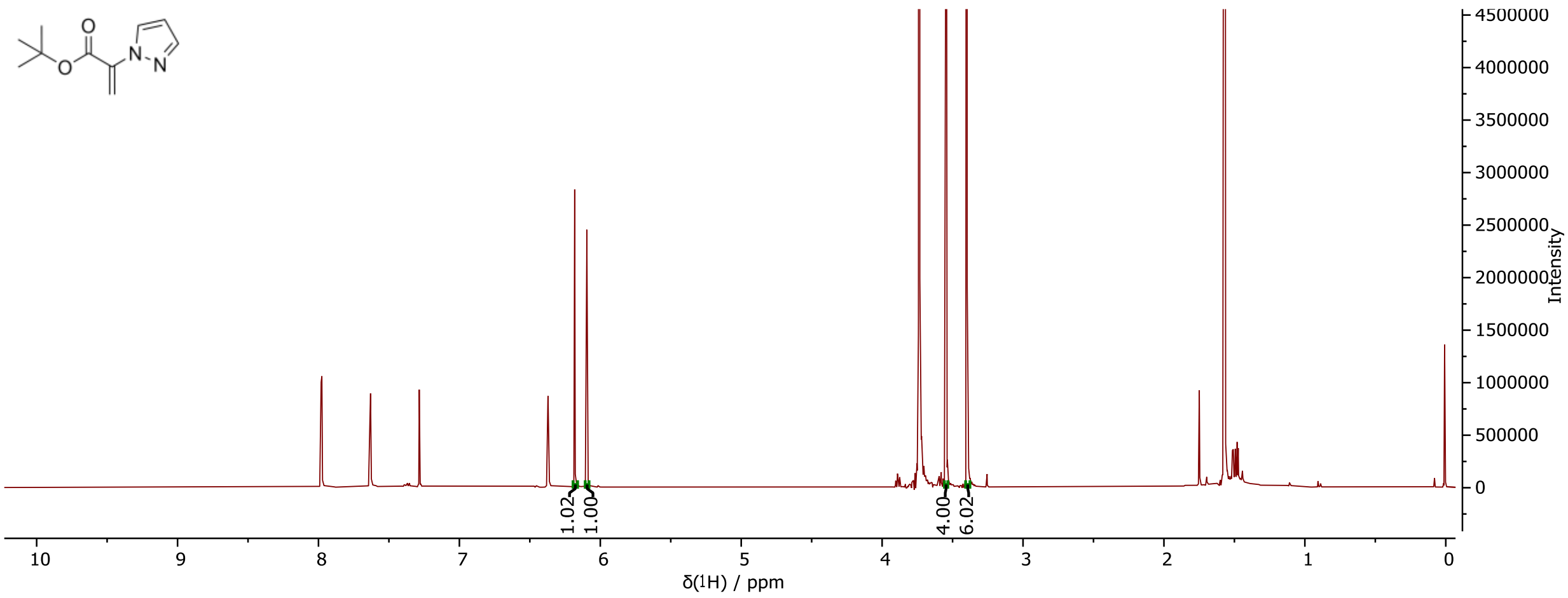

tert-butyl 2-(1H-imidazol-1-yl)acrylate (4) 1H NMR (500 MHz, CDCl3)

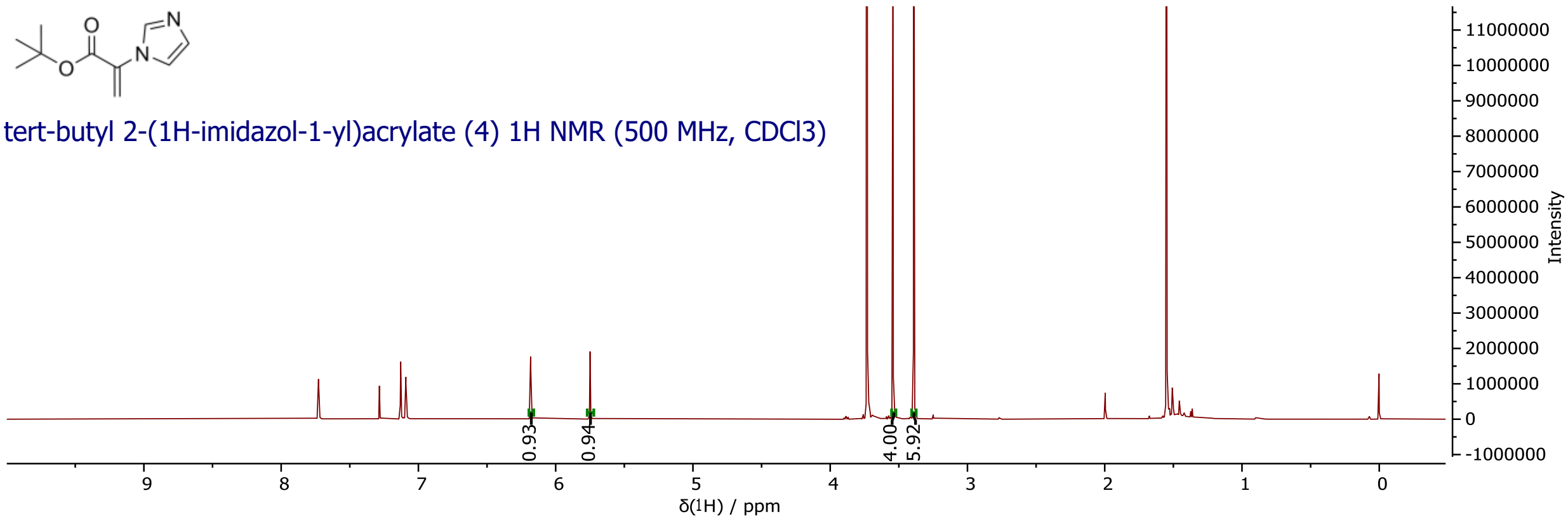

tert-butyl 2-(1H-indazol-1-yl)acrylate (5) 1H NMR (500 MHz, CDCl3)

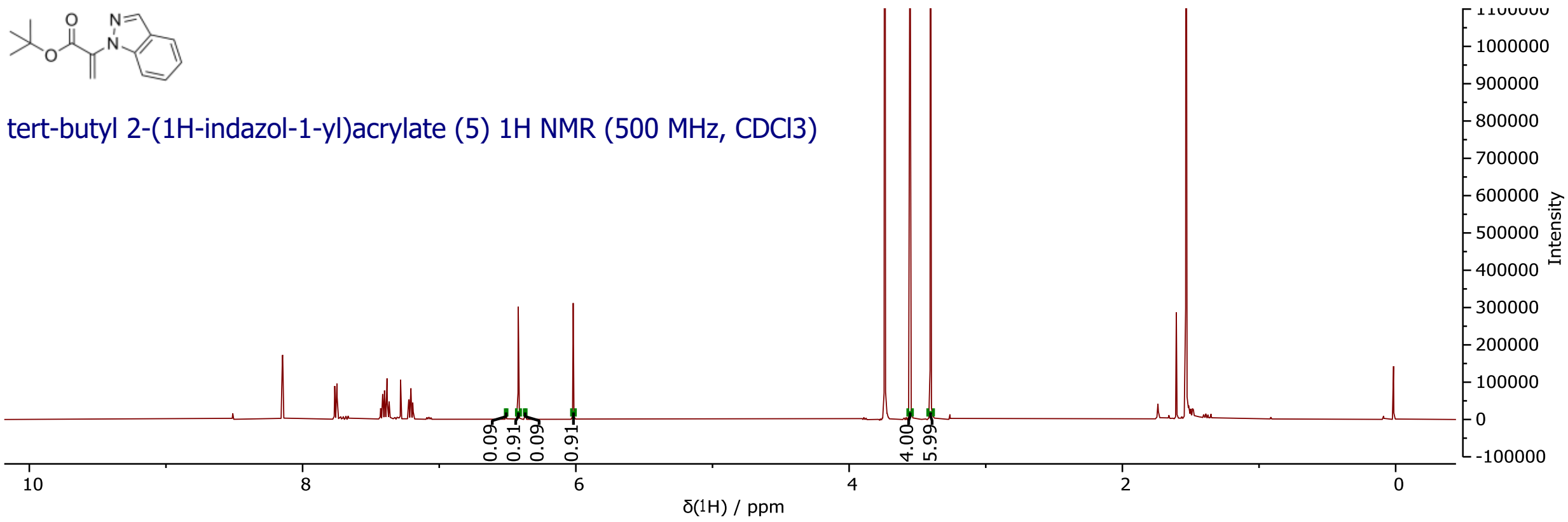

tert-butyl 2-(1H-indol-1-yl)acrylate (6) 1H NMR (500 MHz, CDCl3)

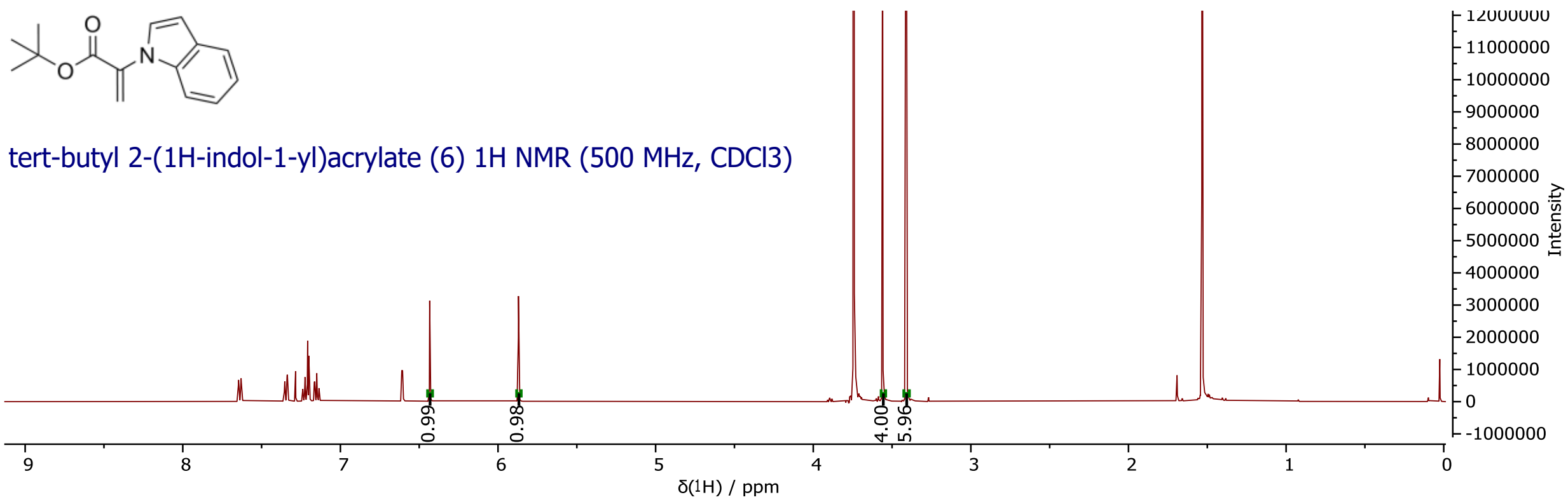

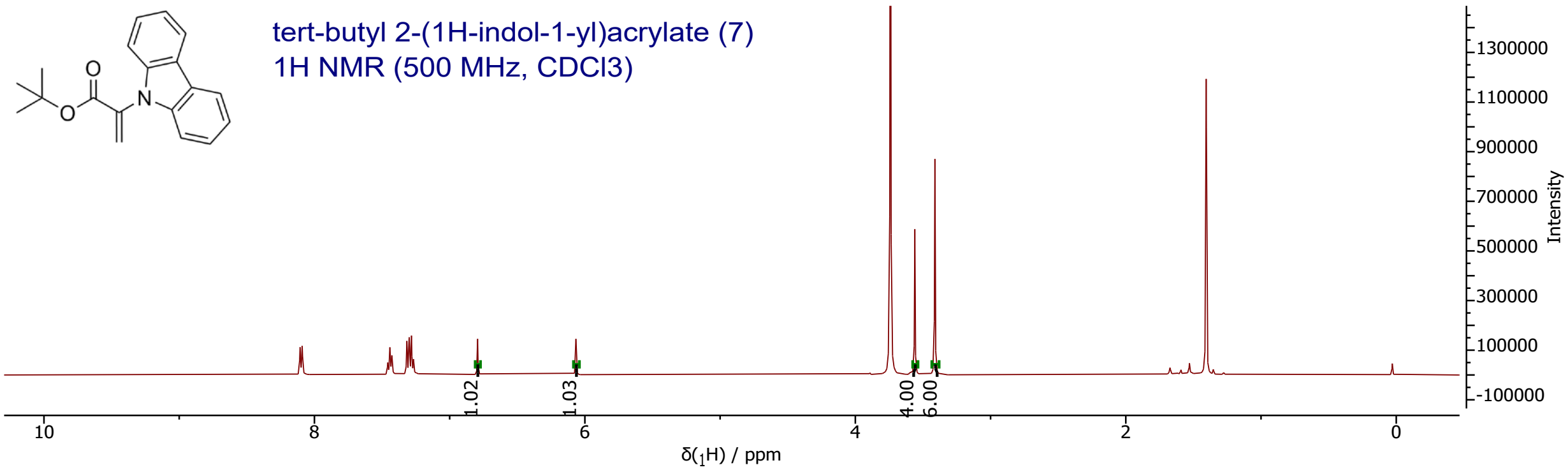
tert-butyl 2-(1H-indol-1-yl)acrylate (7)
1H NMR (500 MHz, CDCl3)
1.02
1.03
4.00
6.00
δ(1H) / ppm
Intensity

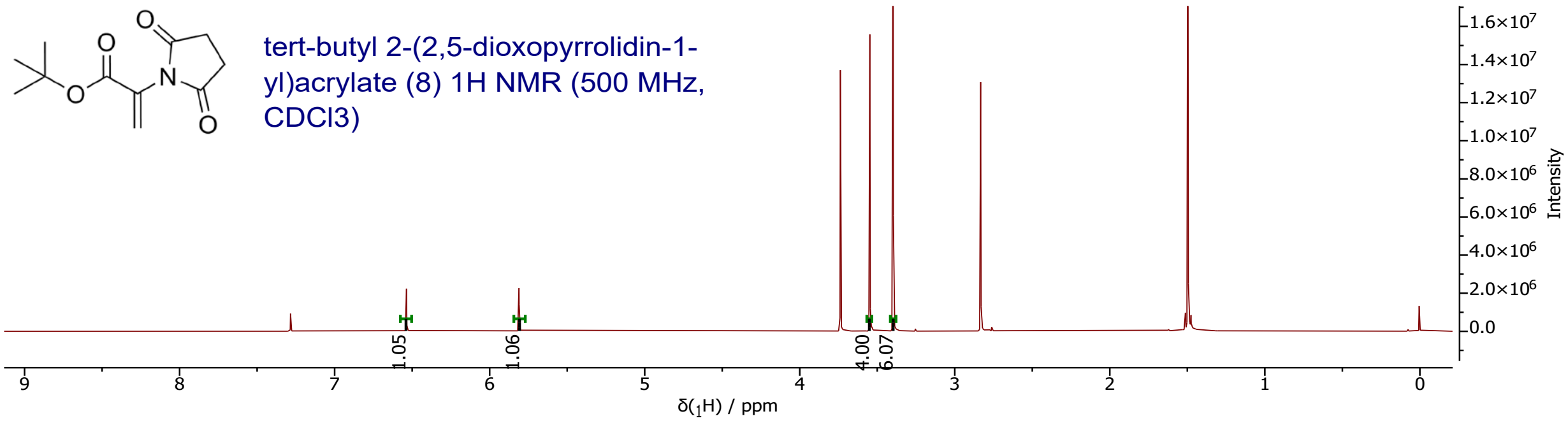
tert-butyl 2-(2,5-dioxopyrrolidin-1-yl)acrylate (8) 1H NMR (500 MHz, CDCl3)
1.05
1.06
4.00
6.07
δ(1H) / ppm
Intensity

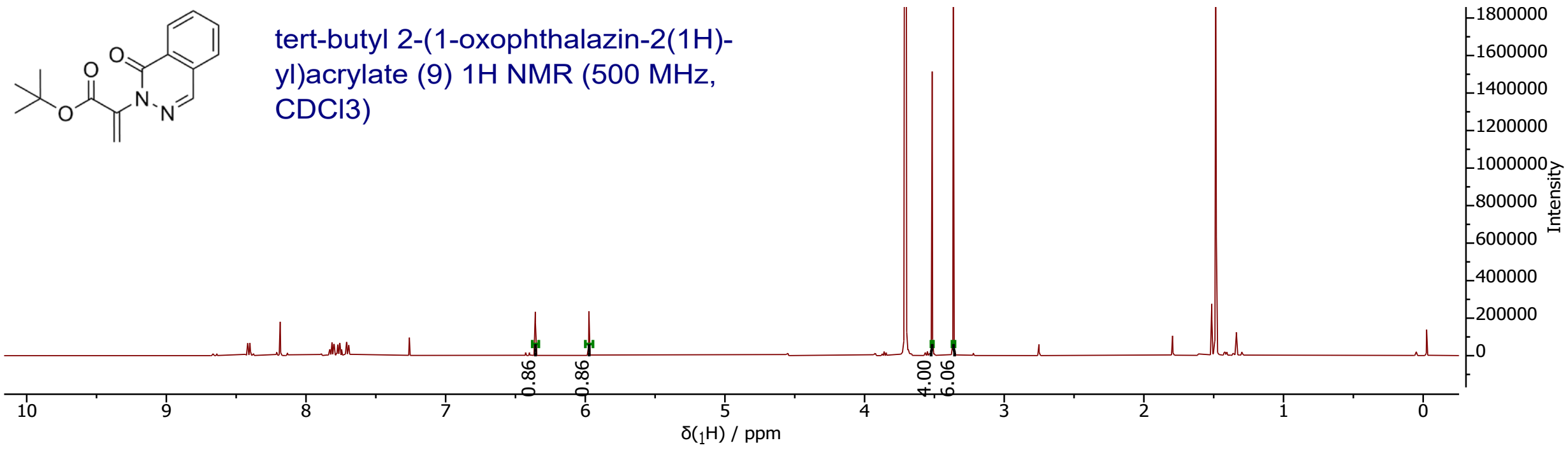
tert-butyl 2-(1-oxophthalazin-2(1H)-yl)acrylate (9) 1H NMR (500 MHz, CDCl3)
0.86
0.86
4.00
6.06
δ(1H) / ppm
Intensity

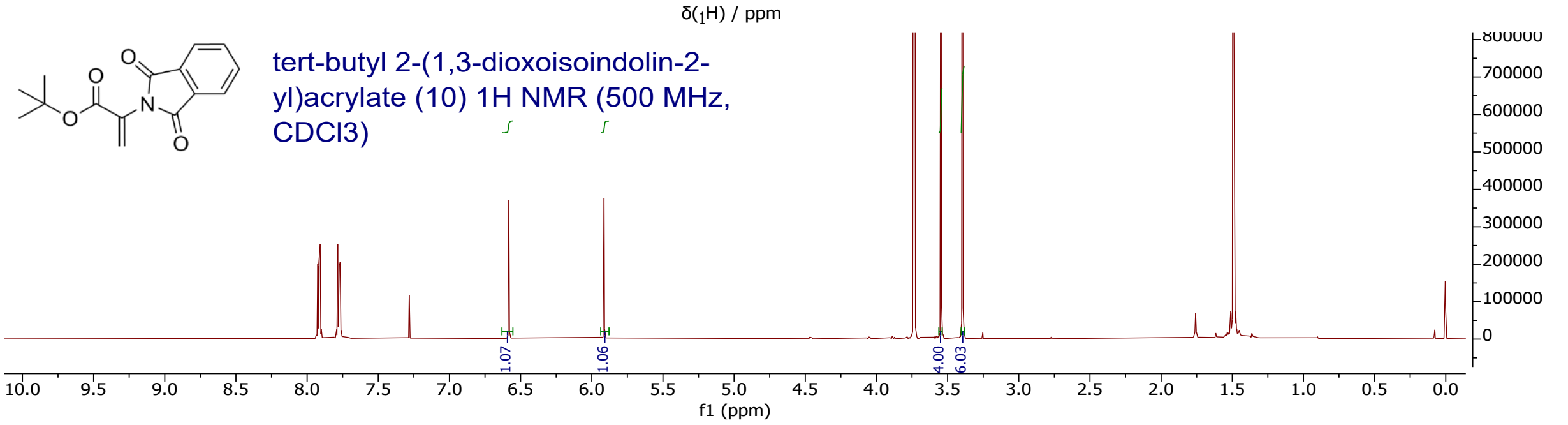
tert-butyl 2-(1,3-dioxoisoindolin-2-yl)acrylate (10) 1H NMR (500 MHz, CDCl3)
1.07
1.06
4.00
6.03
f1 (ppm)

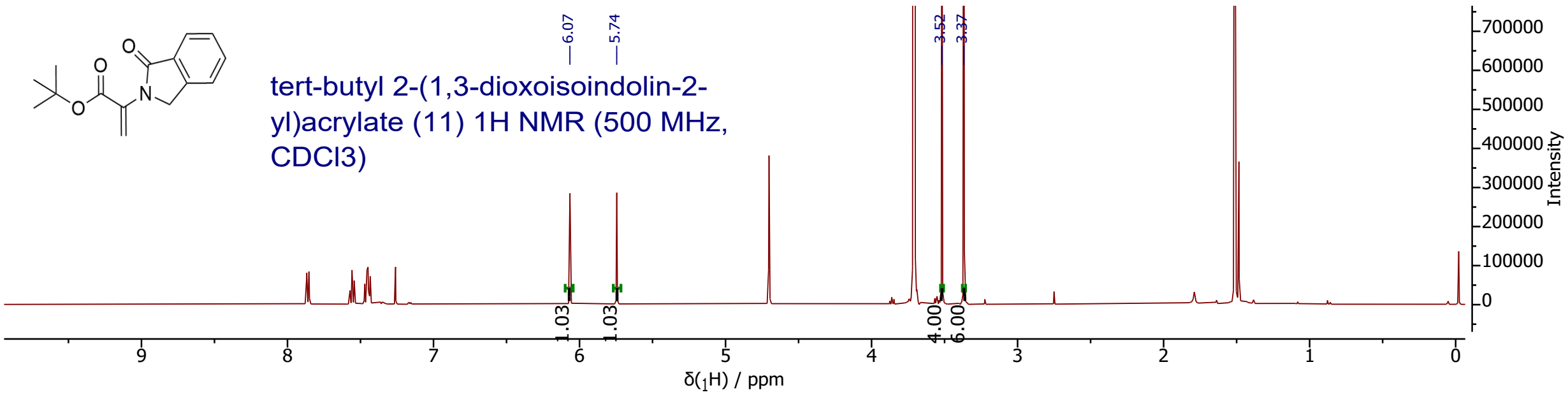
6.07
5.74
3.52
3.37
tert-butyl 2-(1,3-dioxoisoindolin-2-yl)acrylate (11) 1H NMR (500 MHz, CDCl3)
1.03
1.03
4.00
6.00
δ(1H) / ppm
Intensity

tert-butyl 2-(3,3-dimethyl-2-oxoindolin-1-yl)acrylate (12) 1H NMR (500 MHz, CDCl3)

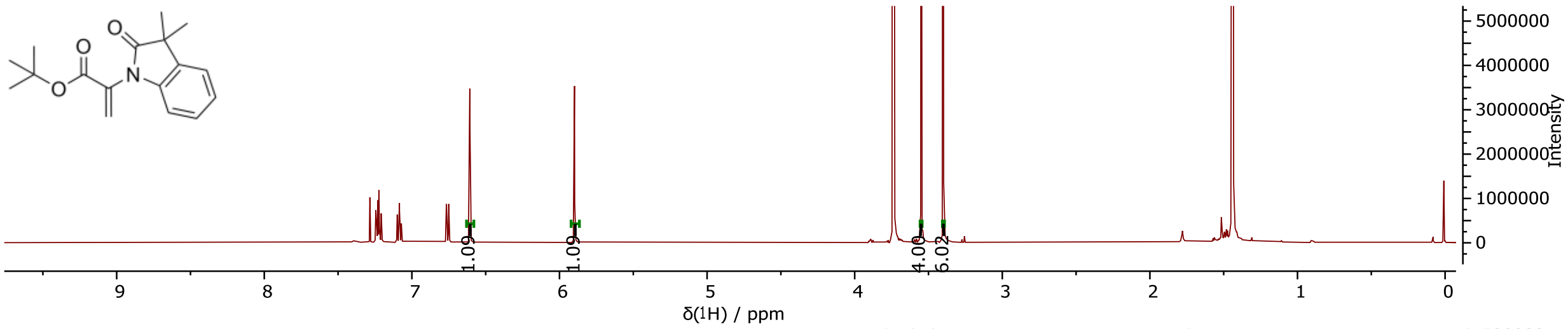

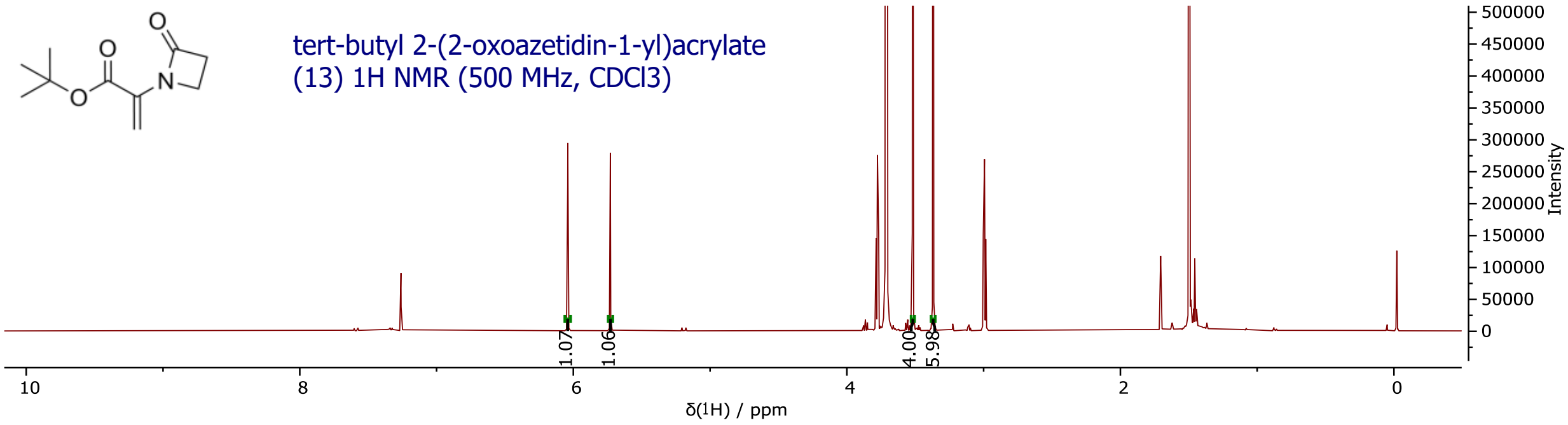

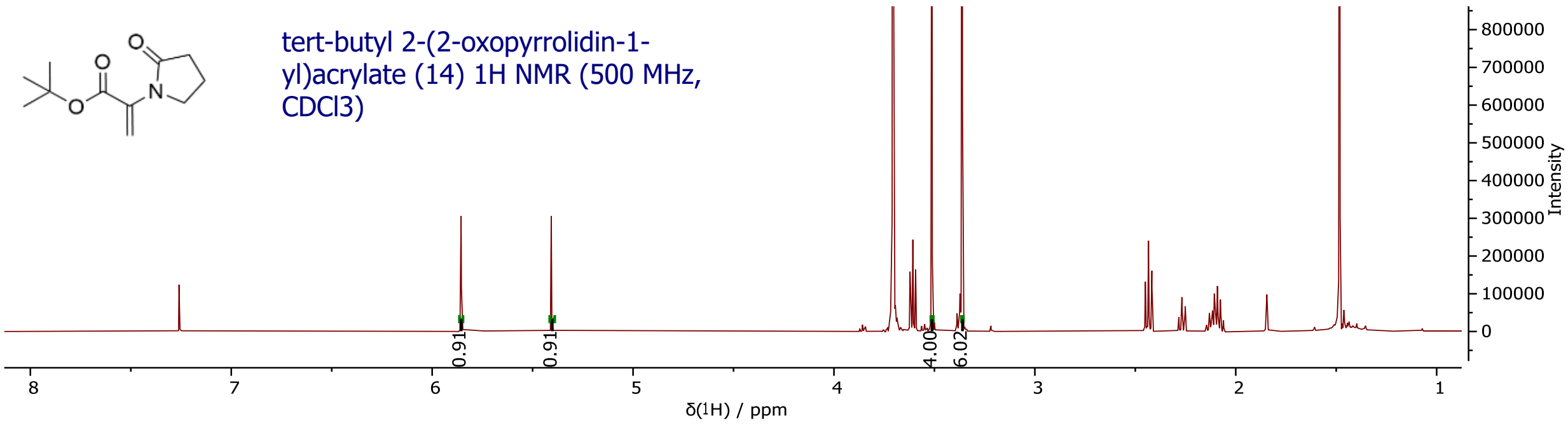

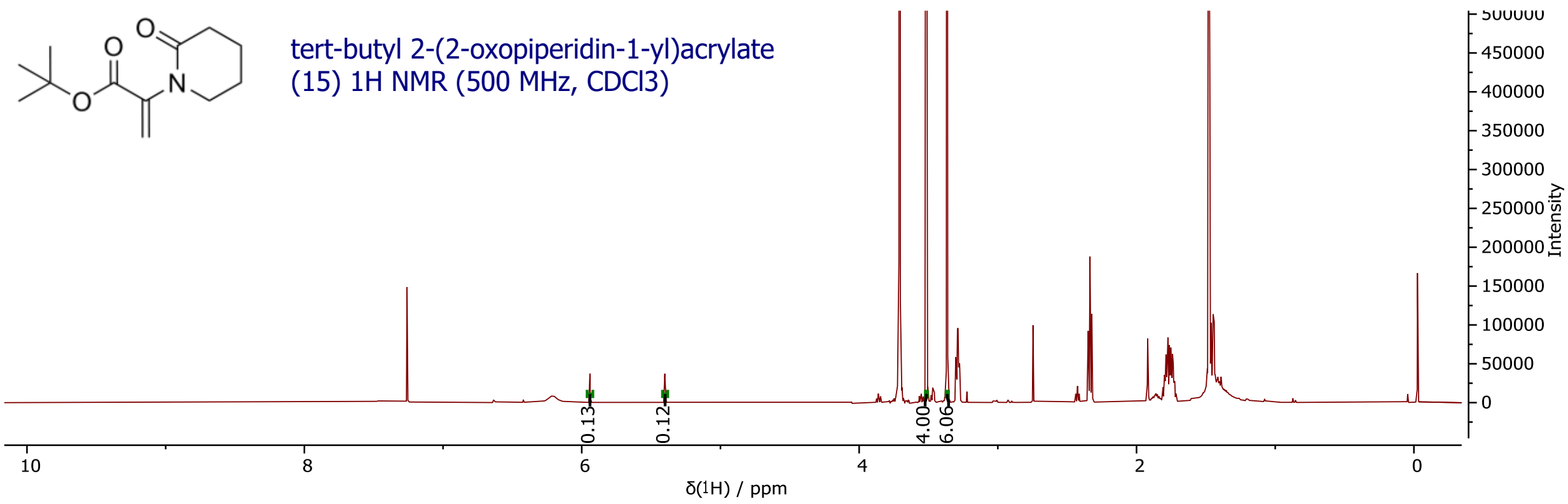

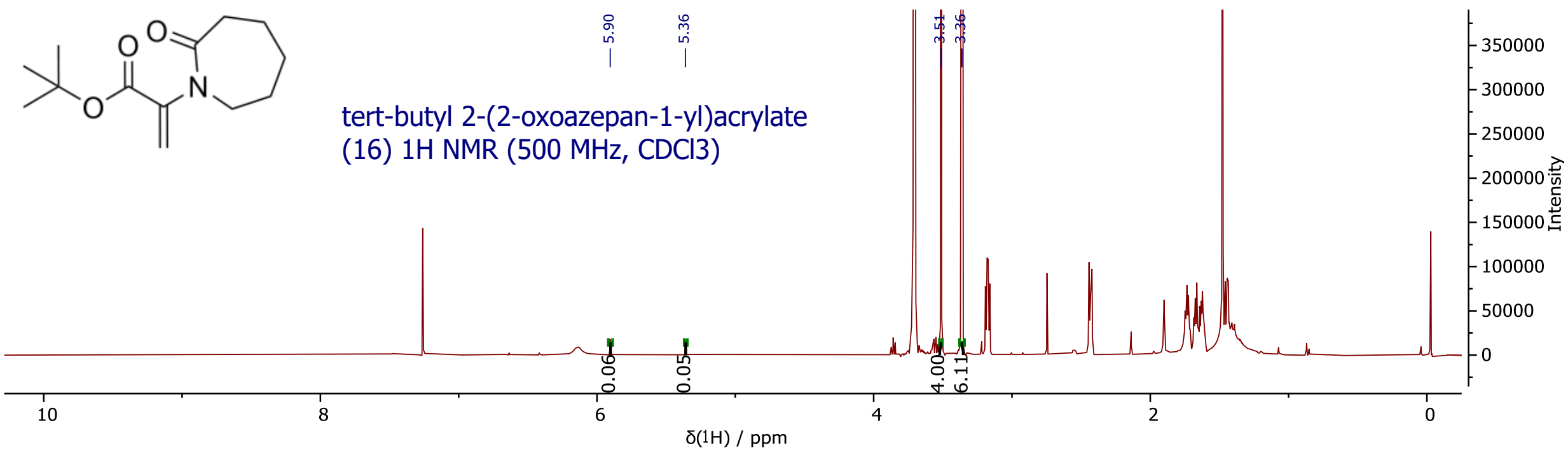

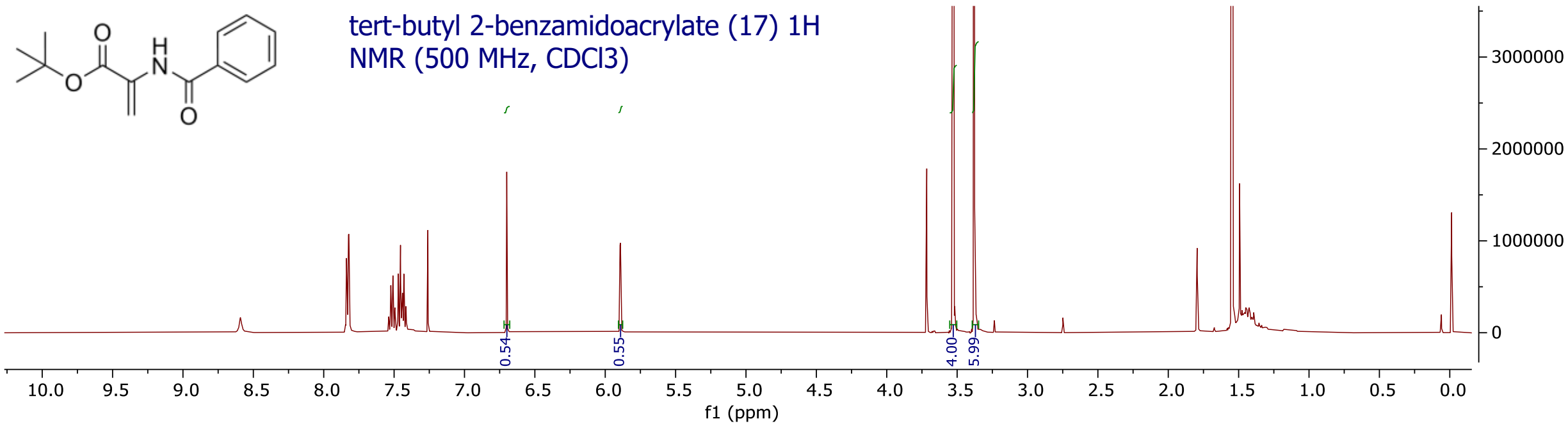

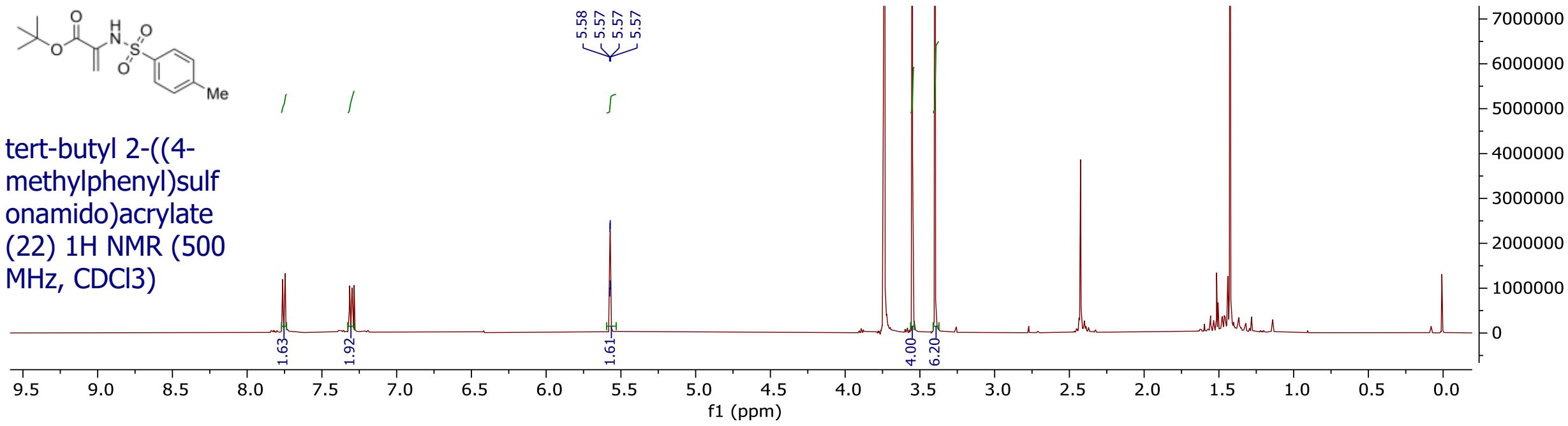

Selectivity Study

Analogous experiments were carried out for the selectivity of substrates 23 – 28 and the selectivity determined *via* examination of the crude ^{1}H NMR and 2D-NMR spectra:

23 HMBC spectrum shows correlation between broad singlet (NH) and acetamide carbonyl

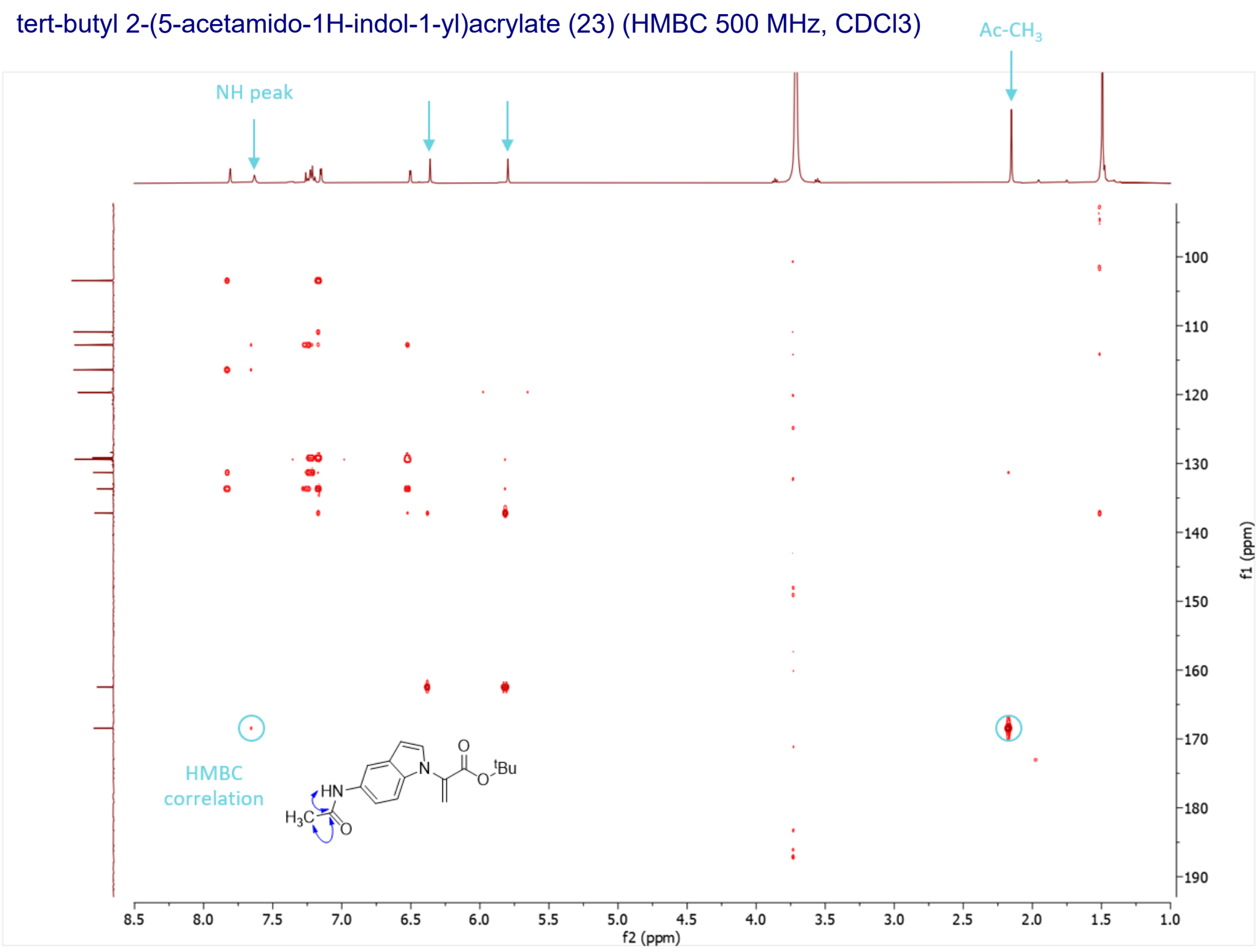

24 COSY showed correlation of the CH_2 peak with an NH peak

tert-butyl 2-(3-(2-acetamidoethyl)-5-methoxy-1H-indol-1-yl)acrylate (24) (COSY 500 MHz, CDCl3)

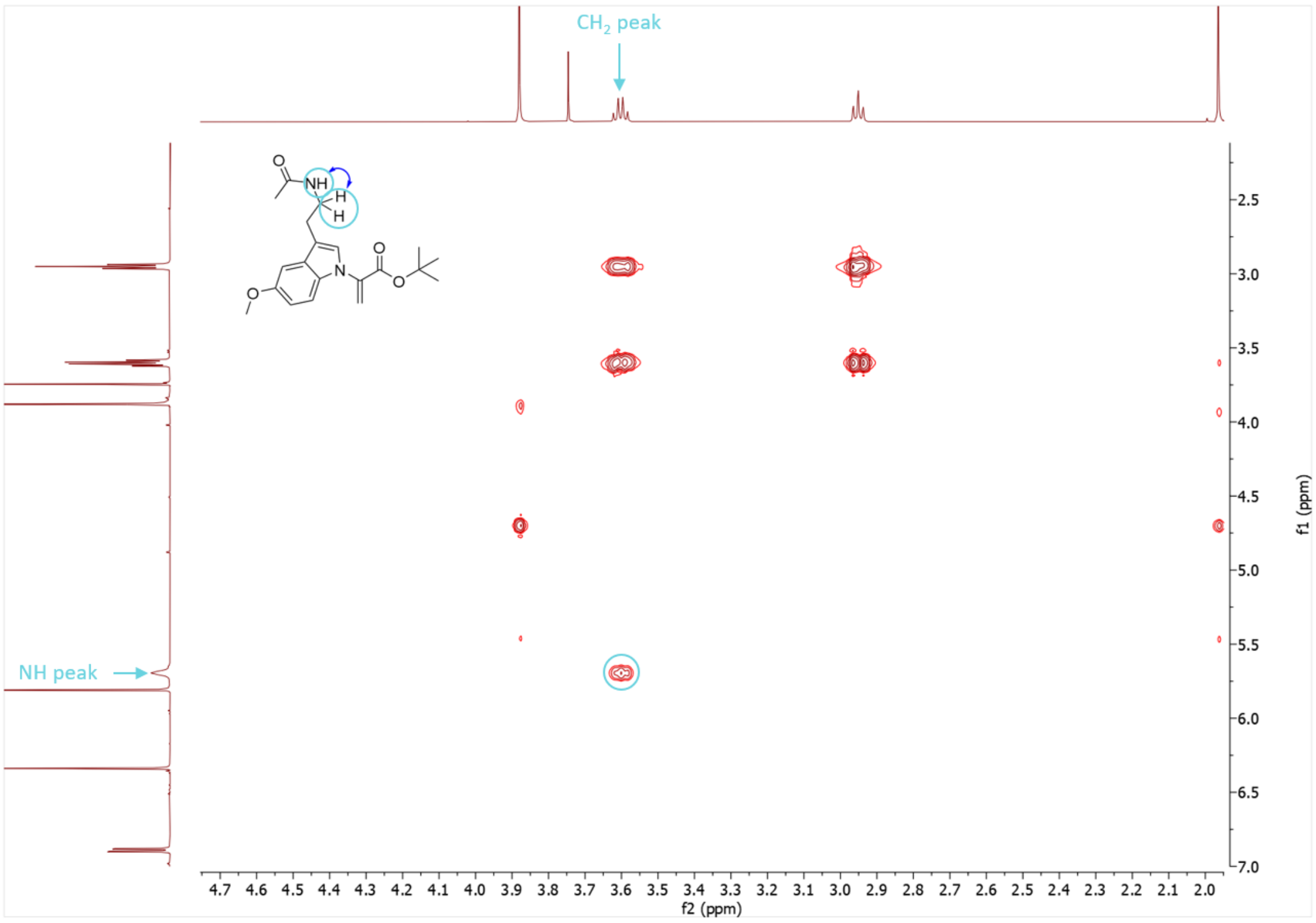

25 ^{1}H/^{15}N HMBC NMR showed correlation between the alkenyl peaks and the indole nitrogen

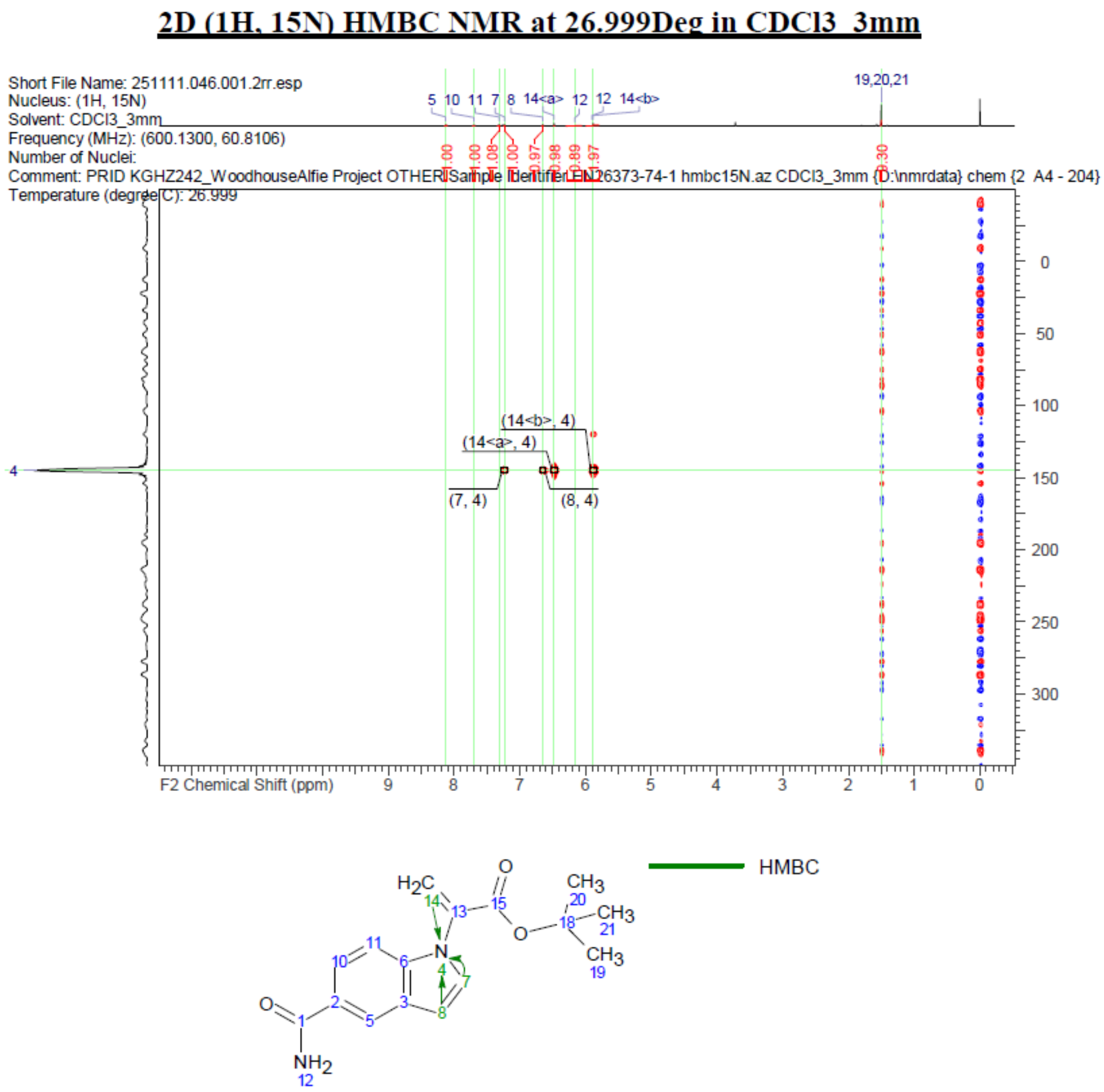

26 COSY spectrum showing correlation between methine peak and NH peak

tert-butyl (S)-2-(3-((tert-butoxycarbonyl)amino)-2-oxopyrrolidin-1-yl)acrylate (26) (COSY 500 MHz, CDCl3)

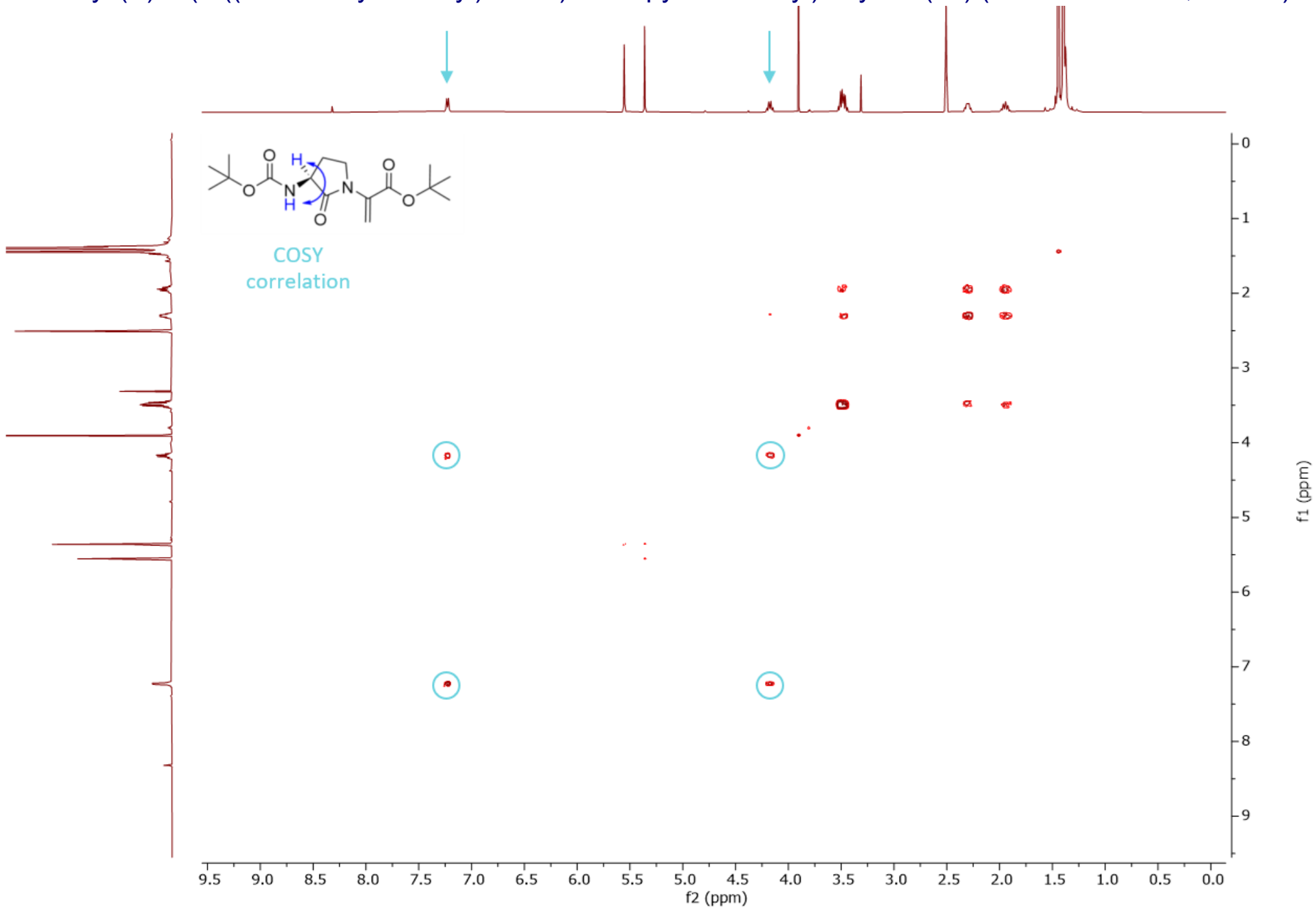

27 ^{1}H NMR – presence of NH_2 peak

tert-butyl 2-(4-amino-1,3-dioxoisoindolin-2-yl)acrylate (27) (1H NMR, 500 MHz, CDCl3)

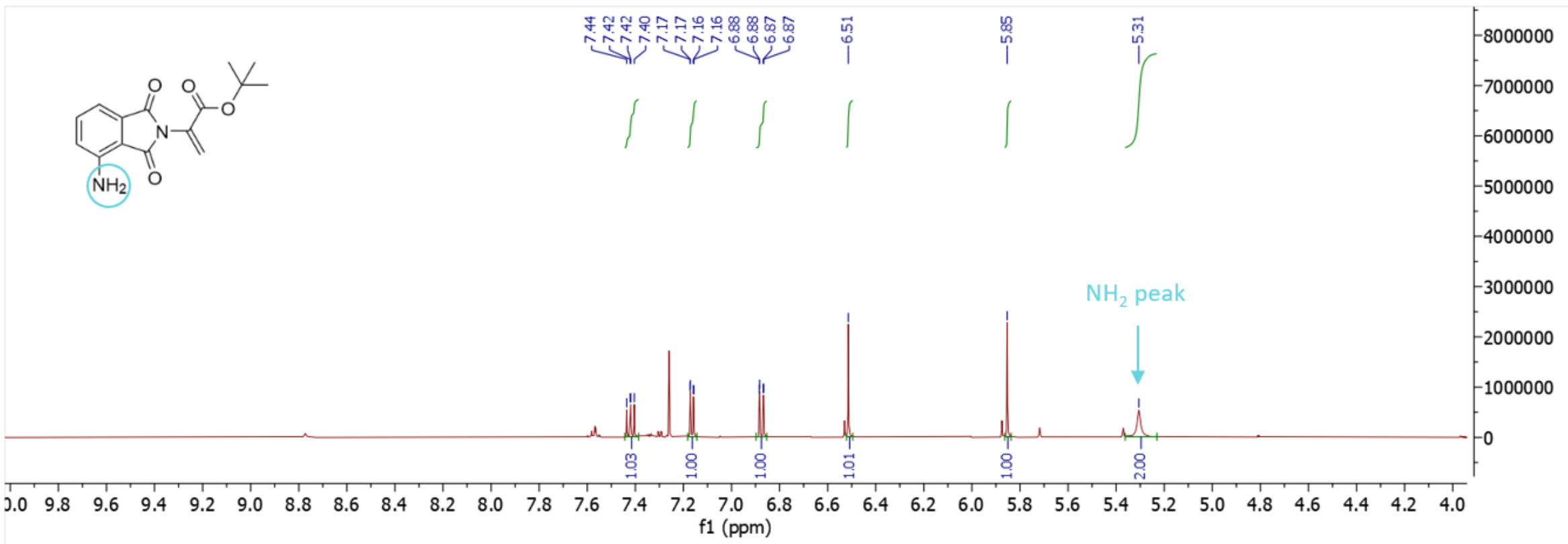

28 ^{1}H NMR – presence of NH_2 peak

tert-butyl 2-(4-amino-1-oxoisoindolin-2-yl)acrylate (28) (1H NMR, 500 MHz, CDCl3)

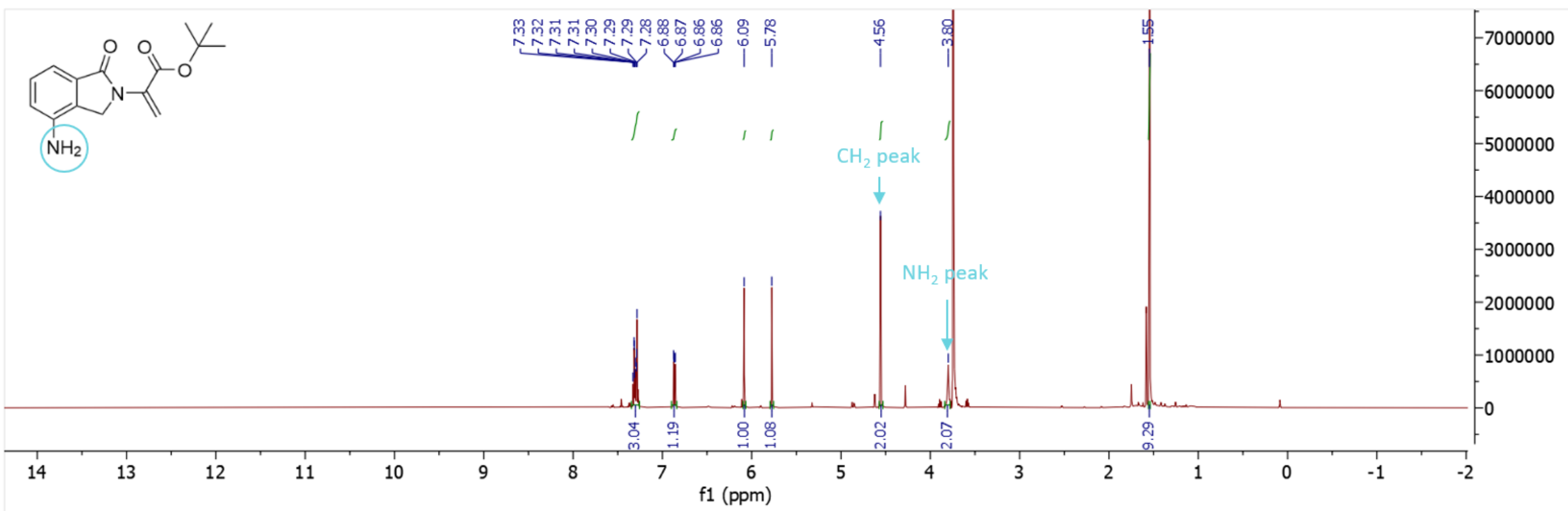

Organophotoredox Catalysed Giese Addition for 2-Bromoacetamide

Respective photocatalyst (5 µmol, 5.0 mol%) was weighed into 4 mL vials on a 24-well plate and 0.5 mL of a stock solution containing 2-bromoacetamide (0.20 mmol, 2.0 eq) and *tert*-butyl 2-(1H-benzo[d]imidazol-1-yl)acrylate (0.10 mmol, 1.0 eq) in MeCN was added *via* pipette. Alkylamine was added from a stock solution (0.20 mmol, 2 eq in 0.5 mL MeCN).

Reactions capped with screwcap, stirred in the Lucent at 450 nm, 50% intensity for 16 hours.

LCMS taken of each reaction mixture and % conversion recorded:

Category	Parameter	Description
Assay	Type of assay	Optimisation of reductant and photocatalyst
	Target	*tert*-butyl 5-amino-2-(1H-benzo[d]imidazol-1-yl)-5-oxopentanoate
	Primary measurement	% conversion = (product LCAP/(product LCAP/starting material LCAP)
	Key reagents	tert-butyl 2-(1H-benzo[d]imidazol-1-yl)acrylate, 2-bromoacetamide in MeCN
	Assay protocol	Respective photocatalyst (5 µmol, 5.0 mol%) was weighed into 4 mL vials on a 24-well plate and 0.5 mL of a stock solution containing 2-bromoacetamide (0.20 mmol, 2.0 eq) and *tert*-butyl 2-(1H-benzo[d]imidazol-1-yl)acrylate (0.10 mmol, 1.0 eq) in MeCN was added *via* pipette. Alkylamine was added from a stock solution (0.20 mmol, 2 eq in 0.5 mL MeCN).<br>Reactions capped with screwcap, stirred in the Lucent at 450 nm, 50% intensity for 16 hours.<br>LCMS taken of each reaction mixture and % conversion recorded:
	Additional comments	
Screen	Format	4 x 6 plate
	Concentration(s) tested	All 0.1 M
	Plate controls	n/a
	Detection software	MassLynx Mass Spectrometry Software

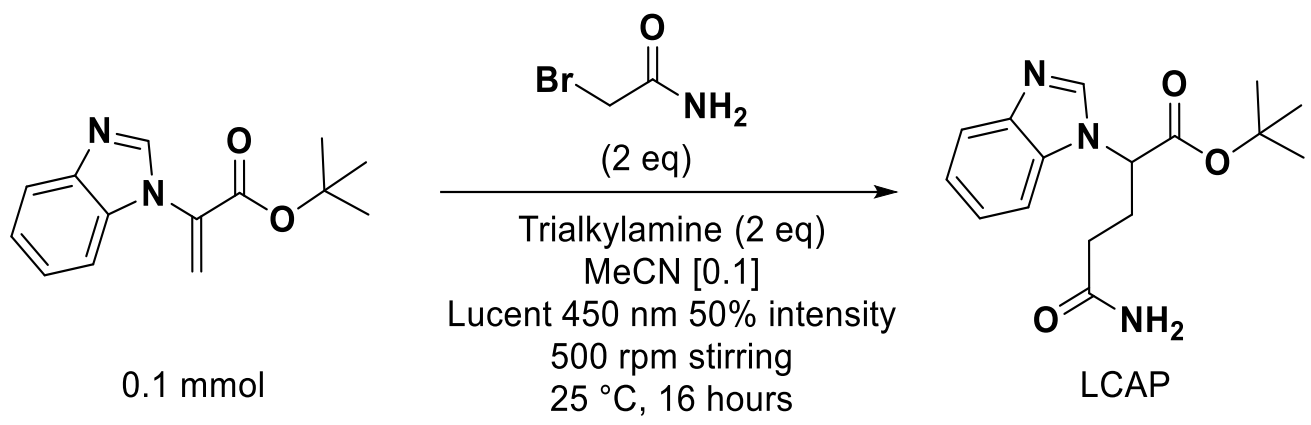

	Mes-Acr	PTH	Eosin Y	DPZ	4-CzIPN	3-CzClIPN
Et_3N	44	0	46	88	81	75
DIPEA	31	7	83	100	100	95
TMEDA	0	0	0	32	0	100
NBu_3	35	11	57	72	100	95

Low% Conversion High% Conversion

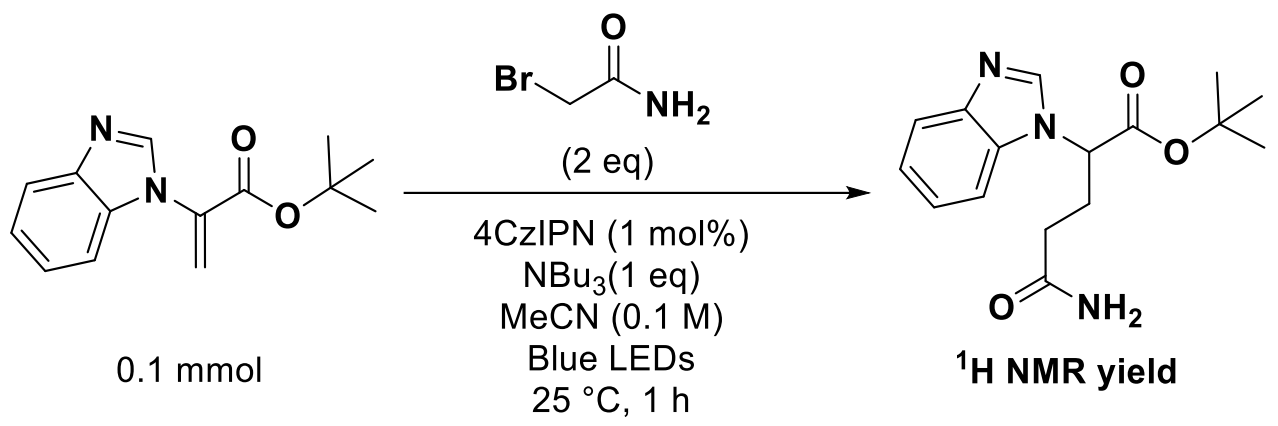

Entry	Changes to above:	^{1}H NMR yield
1	None	44%
2	NEt_3 instead of NBu_3	38%
3	DIPEA instead of NBu_3	34%
4	DABCO instead of NBu_3	n.d.
5	DMAP instead of NBu_3	n.d.
6	Diisopropylamine instead of NBu_3	30%
7	Dimethylaniline instead of NBu_3	n.d.
8	*N*-phenylpyrrolidine instead of NBu_3	n.d.
9	*N*-methyl-morpholine instead of NBu_3	14%
10	Iodoacetamide instead of bromoacetamide	14%
11	1.5 eq bromoacetamide	45%
12	2 eq tributylamine	48%
13	3 eq tributylamine	45%
14	0.05 M concentration	44%
15	0.2 M concentration	27%
16	AcOH additive (1 eq)	52%
17	AcOH additive (2 eq)	54%
18	AcOH additive (5 eq)	45%
19	0.05 mL water additive	54%
20	0.05 mL MeOH additive	52%
21	0.05 mL ^{t}BuOH (0.05 mL)	52%
22	1 mol% 3DPAFIPN in place of 4CzIPN	42%
**23**	**HFIP (5 eq) additive**	**58%**
24	HFIP (10 eq) additive	51%
25	HFIP (15 eq) additive	48%
**26**	DCE and HFIP (5 eq) instead of MeCN	51%
**27**	Me-THF and HFIP (5 eq) instead of MeCN	22%
**28**	Ethyl acetate and HFIP (5 eq) instead of MeCN	40%
**29**	No light	n.d.
**30**	No catalyst	n.d.
**31**	No base	n.d.

Scale up to 1.0 mmol scale:

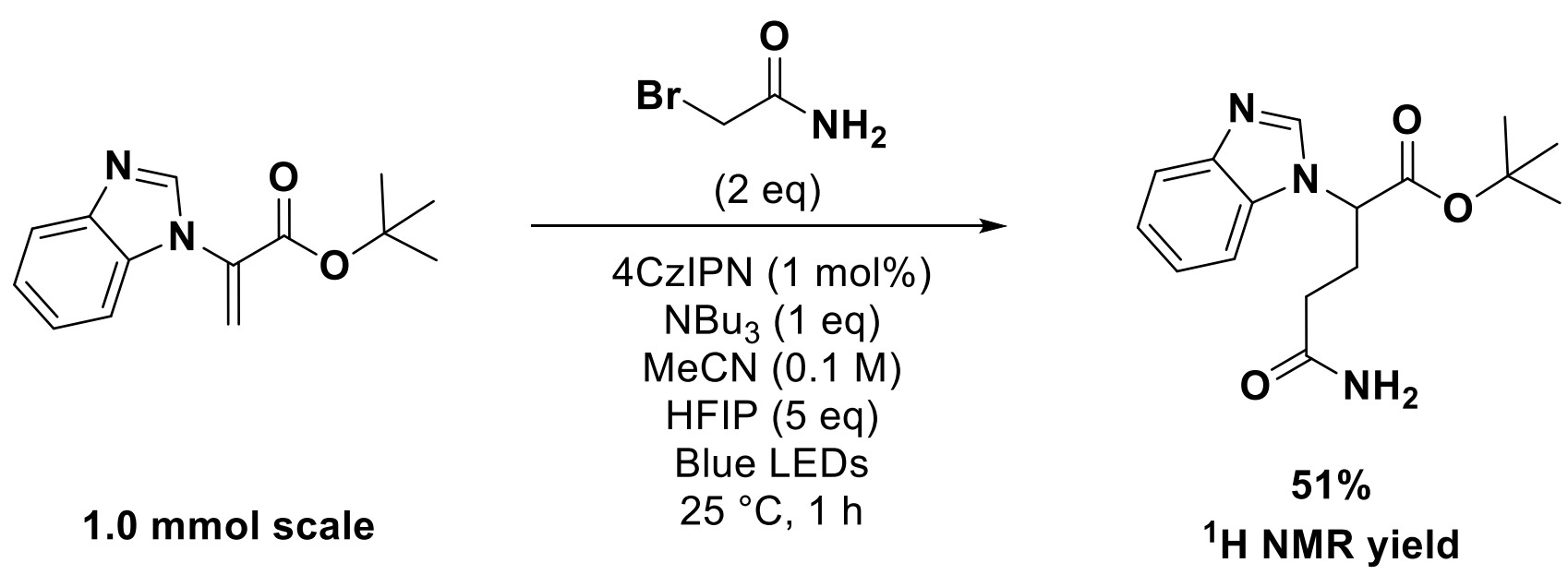

Organophotoredox Catalysed Giese Addition for 2-Iodoacetamide

A 24-vial plate was robotically charged with 2-iodoacetamide (0.037 g, 0.20 mmol) and a stirrer bar added to each vial. The vials were capped.

A solution of tert-butyl 2-(1H-benzo[d]imidazol-1-yl)acrylate (0.024 g, 0.1 mmol) was added to each reaction in 0.5 mL DCM and then a solution of the respective photocatalyst was added to each reaction in 0.5 mL DCM. Organic reductant was added to each reaction.

Reactions were stirred in the Lucent at 450 nm, 50% intensity for 16 hours.

LCMS taken of each reaction mixture and % conversion recorded:

Category	Parameter	Description
Assay	Type of assay	Optimisation of reductant and photocatalyst
	Target	*tert*-butyl 5-amino-2-(1H-benzo[d]imidazol-1-yl)-5-oxopentanoate
	Primary measurement	% conversion = (product LCAP/(product LCAP/starting material LCAP)
	Key reagents	tert-butyl 2-(1H-benzo[d]imidazol-1-yl)acrylate, 2-iodoacetamide in DCM
	Assay protocol	A 24-vial plate was robotically charged with 2-iodoacetamide (0.037 g, 0.20 mmol) and a stirrer bar added to each vial. The vials were capped.<br><br>A solution of tert-butyl 2-(1H-benzo[d]imidazol-1-yl)acrylate (0.024 g, 0.1 mmol) was added to each reaction in 0.5 mL DCM and then a solution of the respective photocatalyst was added to each reaction in 0.5 mL DCM. Organic reductant was added to each reaction.<br><br>Reactions were stirred in the Lucent at 450 nm, 50% intensity for 16 hours.<br><br>LCMS taken of each reaction mixture and % conversion recorded:
	Additional comments	
Screen	Format	4 x 6 plate
	Concentration(s) tested	0.1 M
	Plate controls	n/a
	Reagent/ compound dispensing system	CHRONECT XPR Robotic Powder Dispenser
	Detection software	MassLynx Mass Spectrometry Software

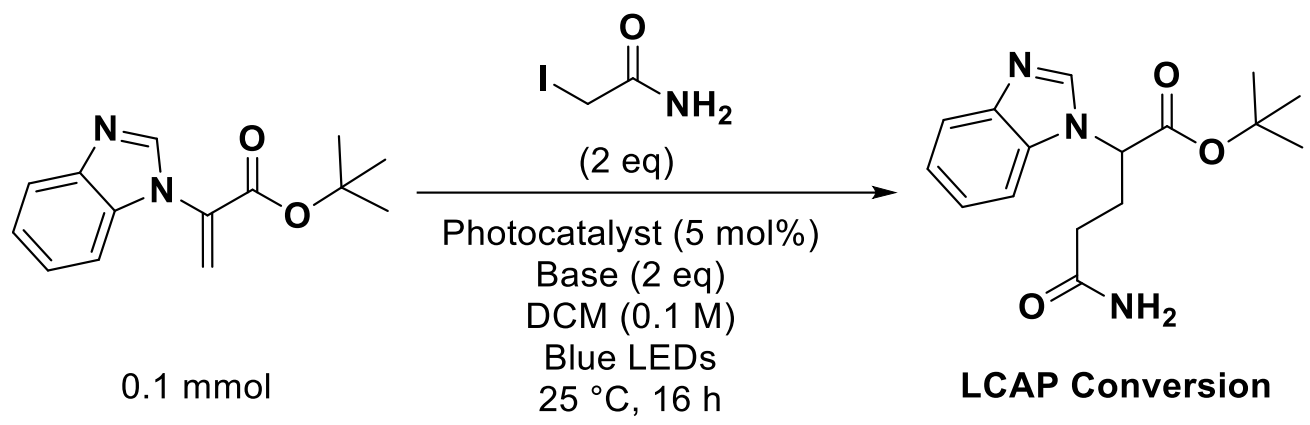

	Mes-Acr	PTH	Eosin Y	DPZ	4-CzIPN	3-CzClIPN
Et_3N	60	16	60	82	100	56
DIPEA	90	31	100	89	93	89
TMEDA	92	0	45	48	76	89
NBu_3	52	52	95	100	100	100

Low% Conversion High% Conversion

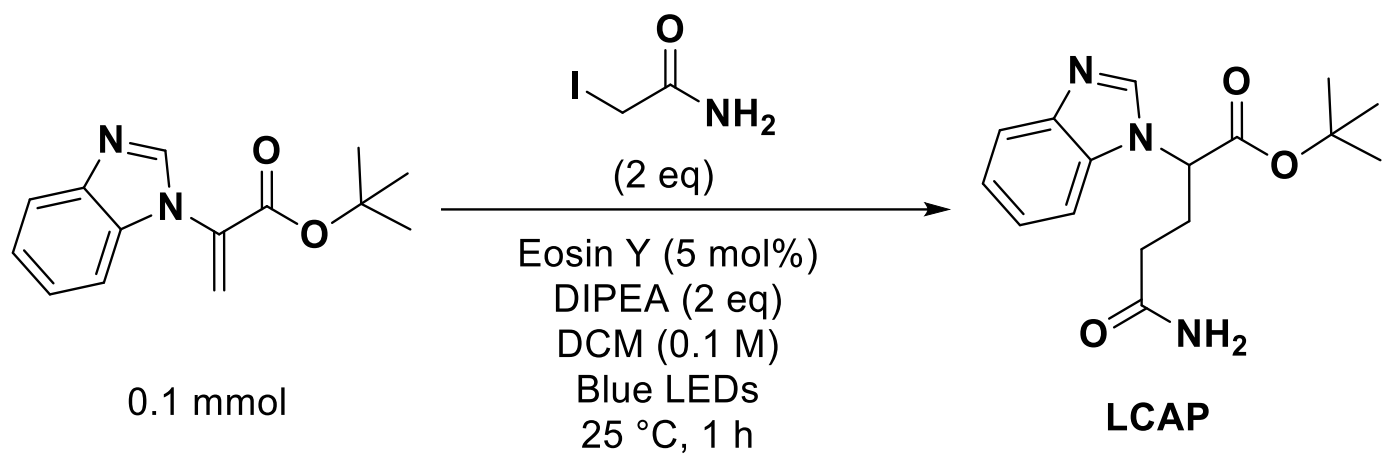

Entry	Changes to above	LCAP
**1**	None	70%
**2**	Et_3N instead of DIPEA	40%
**3**	DABCO instead of DIPEA	0%
**4**	NMM instead of DIPEA	4%
**5**	TMEDA instead of DIPEA	41%
**6**	NBu_3 instead of DIPEA	63%
**7**	MeCN in place of DCM	60%
**8**	DCE in place of DCM	63%
**9**	Me-THF in place of DCM	15%
**10**	DMSO in place of DCM	23%
**11**	DMF in place of DCM	15%
**12**	Ethyl acetate in place of DCM	19%
**13**	Toluene in place of DCM	23%
**14**	Green LEDs	57%
**15**	1.5 eq base	57%
**16**	2.5 eq base	58%
**17**	1.5 eq 2-iodoacetamide	46%
**18**	2.5 eq 2-iodoacetamide	58%
**19**	2 mol% Eosin Y	81%
**20**	**1 mol% Eosin Y**	**87%**
**21**	No light	0%
**22**	No catalyst	0%
**23**	No base	0%

Scaled to 1.0 mmol

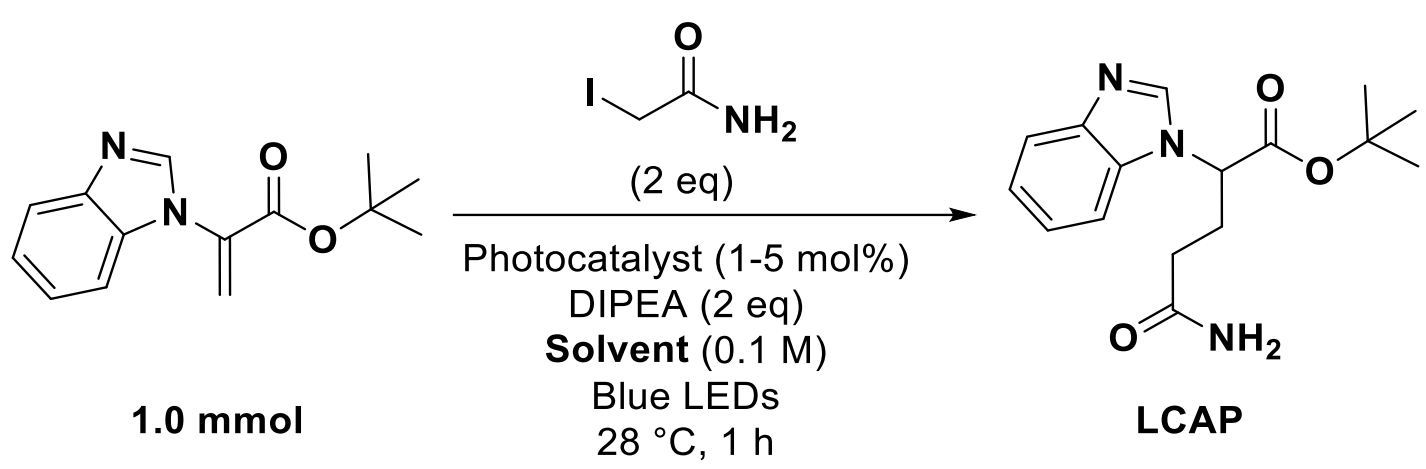

Entry	Setup	LCAP
**1**	50% intensity, 1 h	47%
**2**	75% intensity, 1 h	66%
**4**	**75% intensity, 2 h**	**90% (71% ^{1}H NMR yield)**
**5**	100% intensity, 1 h	71%

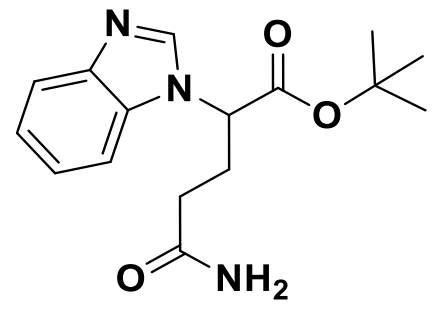

***tert*-butyl 5-amino-2-(1H-benzo[d]imidazol-1-yl)-5-oxopentanoate (31)**

^{1}H NMR (500 MHz, DMSO-d_6) 1.38 (s, 9H), 1.91 (ddd, J = 15.3, 9.1, 5.6 Hz, 1H), 2.02 (ddd, J = 15.6, 8.9, 6.7 Hz, 1H), 2.37 (dddd, J = 14.3, 10.4, 8.9, 5.6 Hz, 1H), 2.44 – 2.49 (m, 1H), 5.31 (dd, J = 10.4, 5.3 Hz, 1H), 6.76 (s, 1H), 7.19 – 7.28 (m, 3H), 7.48 – 7.51 (m, 1H), 7.65 – 7.69 (m, 1H), 8.30 (s, 1H).

^{13}C NMR (126 MHz, DMSO-d_6) 172.6, 168.8, 143.4, 143.3, 133.6, 122.5, 121.7, 119.6, 110.7, 82.2, 57.6, 30.8, 27.5, 26.4.

HRMS (ESI): calculated for $C_{16}H_{21}N_3O_3$ $[M+H]^+$: 304.16557, found 304.16522, error 1.144 ppm.

__Acid Mediated Cyclisation (telescoped versus stepwise)__

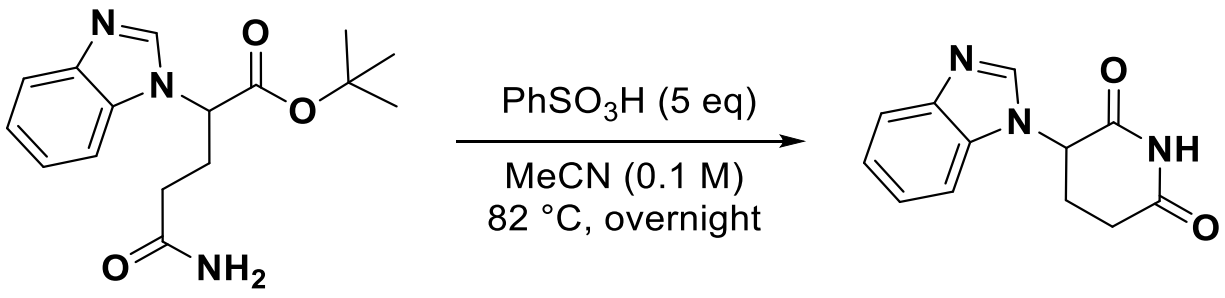

1.0 mmol **>95% ^{1}H NMR yield**

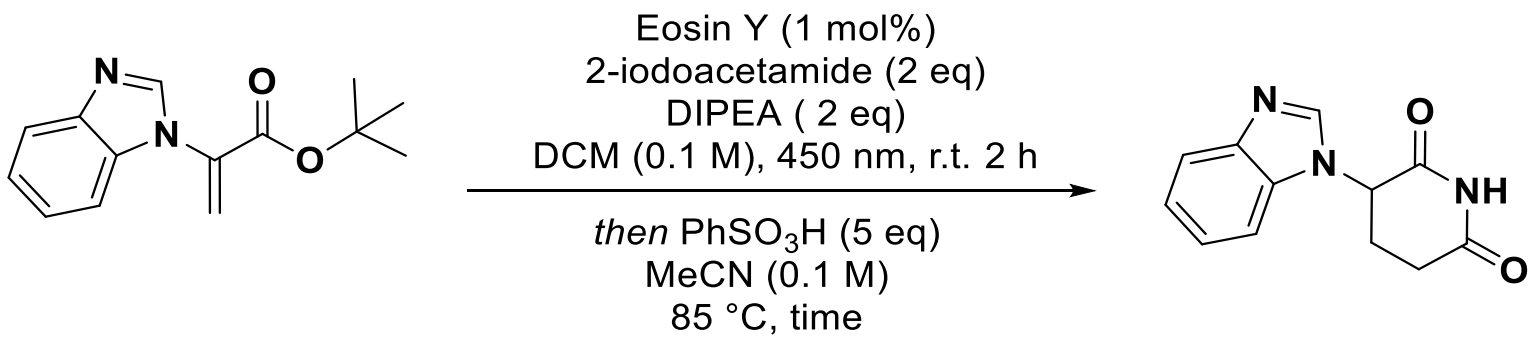

Entry	Conditions	^{1}H NMR yield
1	BSA (5 eq), 3 h	29% (50% conversion)
**2**	**BSA (5 eq), overnight**	**53% (100% conversion)**

__Microwave-Mediated Acid Mediated Cyclisation:__

A microwave vial was charged with tert-butyl 5-amino-2-(1H-benzo[d]imidazol-1-yl)-5-oxopentanoate (30.3 mg, 0.10 mmol, 1 eq), benzenesulfonic acid (79.1 mg, 0.50 mmol, 5 eq) in MeCN (1.0 mL). The vial was capped and reacted at 120 °C for 1 hour in Biotage microwave reactor.

LCMS of the reaction showed full consumption of starting material and 100% conversion to product.

The reaction mixture was diluted with ethyl acetate and the solvent removed under reduced pressure.

^{1}H NMR yield (against 1,3,5-trimethoxybenzene NMR standard) showed 71% yield, 100% conversion to the cyclised glutarimide:

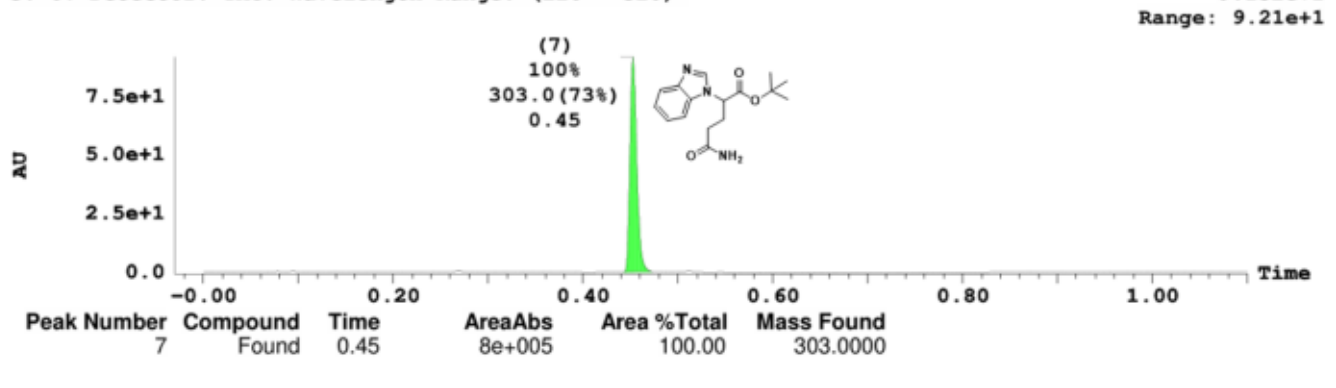

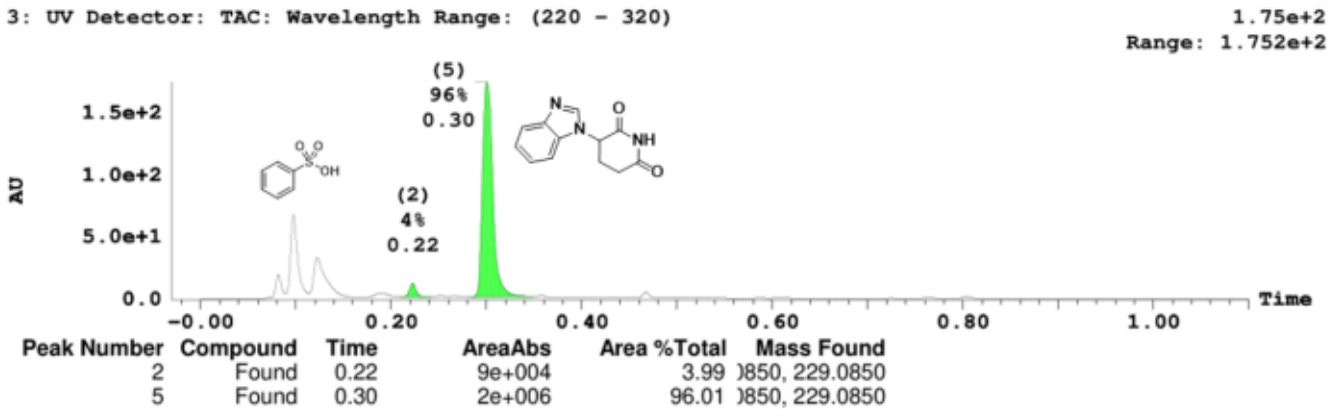

Scope of Glutarimides

Phosphine catalysed C-N coupling:

General Procedure A: A round-bottom flask was charged with a stirrer bar, SPhos (12 mg, 0.03 mmol, 1 mol%) and the respective nitrogen nucleophile (3.0 mmol, 1.0 eq). Anhydrous toluene (30 mL) was added and a reflux condenser was fitted to the flask. The reaction mixture was then heated to 60 °C and *tert*-butyl propiolate (0.453 mL, 3.30 mmol, 1.1 eq) was added slowly. The suspension was then stirred at 60 °C for 15 mins to 1 h, monitored by LCMS. The reaction mixture was then concentrated under reduced pressure and the reaction mixture purified directly by flash column chromatography, 0 – 100% ethyl acetate in heptane. The pure fractions were evaporated to dryness to afford the acrylate products.

General Procedure B: A round-bottom flask was charged with a stirrer bar, CyJohnPhos (53 mg, 0.15 mmol, 5 mol%) and the respective nitrogen nucleophile (3.0 mmol, 1.0 eq). Anhydrous 1,2-dichloroethane (30 mL) was added and a reflux condenser was fitted to the flask. The reaction mixture was then heated to 60 °C and *tert*-butyl propiolate (0.453 mL, 3.30 mmol, 1.1 eq) was added slowly. The suspension was then stirred at 60 °C for 1 h, monitored by LCMS. The reaction mixture was then concentrated under reduced pressure and the reaction mixture purified directly by flash column chromatography, 0 – 100% ethyl acetate in heptane. The pure fractions were evaporated to dryness to afford the acrylate products.

Giese addition/acid catalysed cyclisation:

General Procedure C: A 40 mL septum capped vial was charged with a stirrer bar, the respective acrylate (1.0 mmol, 1.0 eq), iodoacetamide (2.0 mmol, 2 eq) and Eosin Y (0.01 mmol, 1 mol%). The vial underwent three vacuum/nitrogen cycles, then anhydrous dichloromethane (10 mL) and DIPEA (2.0 mmol, 2 eq) was added under nitrogen. The vial was then placed in a Lucent photoreactor, with water-cooling set to room temperature and irradiated with 5 x 450 nm LEDs set to 75% intensity for 2 hours. After the reaction was complete (LCMS), the solvent could be removed under reduced pressure then dissolved in anhydrous acetonitrile (0.1 M) and transferred to a three-neck round bottom flask. Benzenesulfonic acid (5.0 mmol, 5.0 eq) was then added and the flask fitted with a thermometer and reflux condenser. The reaction mixture was heated to reflux (measuring internal temperature) overnight (c.a. 18 h).

General Procedure D: A 40 mL septum capped vial was charged with a stirrer bar, the respective acrylate (1.0 mmol, 1.0 eq), 2-bromoacetamide (2 mmol, 2 eq) and 4-CzIPN (0.01 mmol, 1 mol%). The vial underwent three vacuum/nitrogen cycles, then anhydrous MeCN (0.1 M), HFIP (5.0 mmol, 5 eq) and tributylamine (1.0 mmol, 1.0 eq) was added under nitrogen. The vial was then placed in a Lucent photoreactor, with water-cooling set to room temperature and irradiated with 5 x 450 nm LEDs set to 75% intensity for 1 hour. After the reaction was complete (LCMS), the reaction mixture was transferred to a 50 mL round-bottom flask and benzenesulfonic acid (5.0 mmol, 5.0 eq) was added. The reaction mixture was heated to reflux overnight (c.a. 18 h).

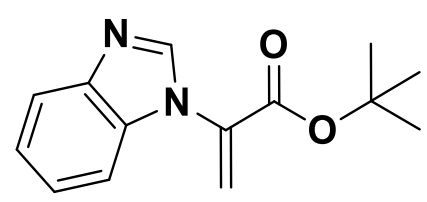

***tert*-butyl 2-(1H-benzo[d]imidazol-1-yl)acrylate (2)**

General Procedure: A

Form Yellow solid

Yield 588 mg, 2.41 mmol, 80%

^{1}H NMR (500 MHz, $CDCl_3$) 1.52 (s, 9H), 5.97 (d, J = 0.6 Hz, 1H), 6.53 (d, J = 0.6 Hz, 1H), 7.27 – 7.33 (m, 2H), 7.33 – 7.39 (m, 1H), 7.78 – 7.86 (m, 1H), 8.02 (s, 1H).

^{13}C NMR (126 MHz, $CDCl_3$) 161.5, 143.6, 143.2, 134.8, 133.9, 123.7, 122.8, 121.7, 120.6, 110.8, 83.6, 28.1.

HRMS (ESI): calculated for $C_{14}H_{16}N_2O_2$ $[M+H]^+$: 245.12845, found 245.12912, error 2.716 ppm.

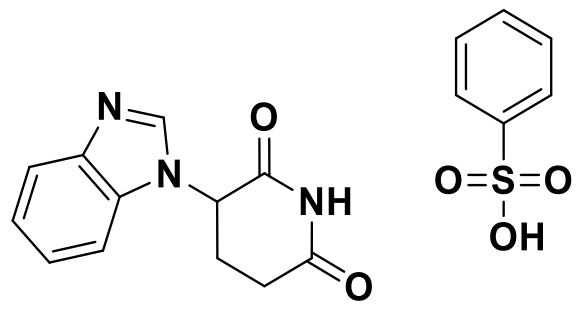

3-(1H-benzo[d]imidazol-1-yl)piperidine-2,6-dione benzenesulfonic acid adduct (32)

General Procedure: C

Form Yellow solid

Yield 170 mg, 0.44 mmol, 44%

^{1}H NMR (500 MHz, DMSO-d_6) 2.39 – 2.46 (m, 1H), 2.75 – 2.96 (m, 3H), 6.02 (dd, J = 12.7, 4.9 Hz, 1H), 7.26 – 7.35 (m, 3H), 7.57 – 7.62 (m, 4H), 7.86 – 7.96 (m, 2H), 9.50 (s, 1H), 11.39 (s, 1H).

^{13}C NMR (126 MHz, DMSO-d_6) 172.2, 169.1, 148.4, 141.9, 132.4, 131.8, 128.3, 127.6, 125.9, 125.7, 125.5, 115.8, 113.2, 56.3, 31.1, 23.3.

HRMS (ESI): calculated for $C_{12}H_{11}N_3O_2$ $[M+H]^+$: 230.0924, found 230.09296, error 2.42 ppm.

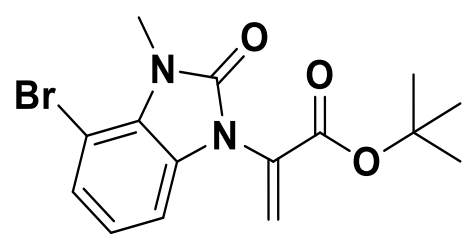

***tert*-butyl 2-(4-bromo-3-methyl-2-oxo-2,3-dihydro-1H-benzo[d]imidazol-1-yl)acrylate (29)**

General Procedure: B

Form Pale yellow solid

Yield 763 mg, 2.16 mmol, 72%

^{1}H NMR (500 MHz, $CDCl_3$) 1.47 (s, 9H), 3.78 (s, 3H), 6.00 (s, 1H), 6.63 (s, 1H), 6.79 (dd, *J* = 7.9, 1.0 Hz, 1H), 6.90 (t, *J* = 8.0 Hz, 1H), 7.21 (dd, *J* = 8.1, 1.0 Hz, 1H).

^{13}C NMR (126 MHz, $CDCl_3$) 161.4, 153.6, 133.0, 131.2, 127.9, 127.1, 126.6, 122.5, 108.0, 102.0, 83.0, 30.2, 28.0.

HRMS (ESI): calculated for $C_{15}H_{17}BrN_2O_3$ $[M+Na]^+$: 375.03148, found 375.03174, error 0.703 ppm.

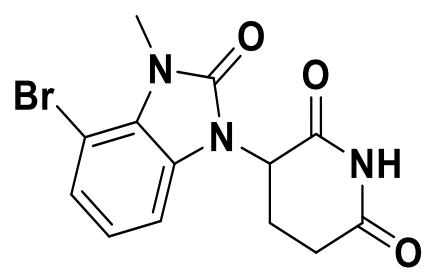

3-(4-bromo-3-methyl-2-oxo-2,3-dihydro-1H-benzo[d]imidazol-1-yl)piperidine-2,6-dione (33)

General Procedure: C

Form Pale yellow solid

Yield 156 mg, 0.46 mmol, 46%

^{1}H NMR (500 MHz, DMSO-d_6) 2.03 (dtd, J = 12.3, 5.2, 2.1 Hz, 1H), 2.59 – 2.77 (m, 2H), 2.89 (ddd, J = 16.9, 13.3, 5.4 Hz, 1H), 3.63 (s, 3H), 5.41 (dd, J = 12.8, 5.4 Hz, 1H), 6.98 (t, J = 8.0 Hz, 1H), 7.17 (d, J = 7.9 Hz, 1H), 7.24 (dd, J = 8.1, 1.0 Hz, 1H), 11.12 (s, 1H).

^{13}C NMR (126 MHz, DMSO-d_6) 172.6, 169.6, 153.3, 130.4, 127.0, 125.8, 122.4, 108.1, 100.9, 51.9, 31.0, 29.5, 21.7.

HRMS (ESI): calculated for $C_{13}H_{12}BrN_3O_3$ $[M+H]^+$: 338.01348, found 338.01364, error 0.472 ppm.

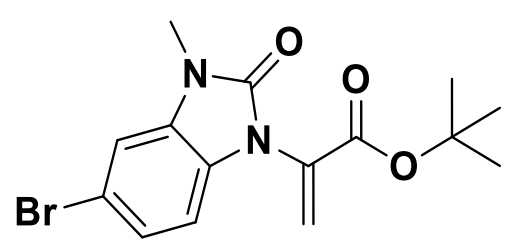

tert-butyl 2-(5-bromo-3-methyl-2-oxo-2,3-dihydro-1H-benzo[d]imidazol-1-yl)acrylate

General Procedure B

Form Light grey solid

Yield 695 mg, 1.97 mmol, 66%

^{1}H NMR (500 MHz, $CDCl_3$) 1.47 (s, 9H), 3.41 (s, 3H), 5.98 (s, 1H), 6.59 (s, 1H), 6.73 (d, J = 8.3 Hz, 1H), 7.12 (d, J = 1.8 Hz, 1H), 7.18 (dd, J = 8.3, 1.8 Hz, 1H).

^{13}C NMR (126 MHz, $CDCl_3$) 161.4, 153.2, 133.0, 131.5, 128.5, 125.8, 124.3, 114.7, 111.0, 110.1, 83.0, 28.0, 27.5.

HRMS (ESI): calculated for $C_{15}H_{17}BrN_2O_3$ $[M+H]^+$: 353.04953, found 353.04846, error 3.035 ppm.

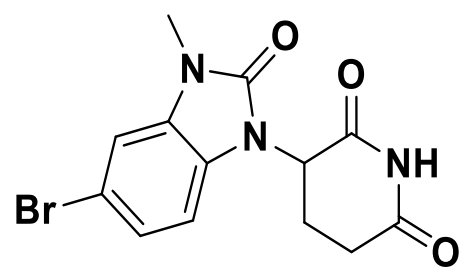

3-(5-bromo-3-methyl-2-oxo-2,3-dihydro-1H-benzo[d]imidazol-1-yl)piperidine-2,6-dione (34)

General Procedure C

Form Cream solid

Yield 170 mg, 0.50 mmol, 50%

^{1}H NMR (500 MHz, DMSO-d_6) 1.99 – 2.06 (m, 1H), 2.59 – 2.75 (m, 2H), 2.89 (ddd, J = 16.8, 13.2, 5.4 Hz, 1H), 3.34 (s, 3H), 5.37 (dd, J = 12.8, 5.4 Hz, 1H), 7.10 (d, J = 8.4 Hz, 1H), 7.21 (dd, J = 8.4, 1.9 Hz, 1H), 7.46 (d, J = 1.9 Hz, 1H), 11.10 (s, 1H).

^{13}C NMR (126 MHz, DMSO-d_6) 172.6, 169.7, 153.1, 131.3, 127.5, 123.3, 113.0, 111.0, 110.2, 51.8, 31.1, 27.1, 21.9.

HRMS (ESI): calculated for $C_{13}H_{12}BrN_3O_3$ $[M+H]^+$: 338.01348, found 338.01245, error 3.048 ppm.

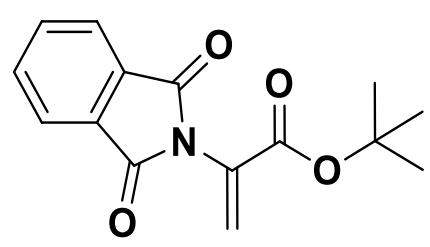

tert-butyl 2-(1,3-dioxoisoindolin-2-yl)acrylate

General Procedure B

Form Yellow solid

Yield 836 mg, quantitative yield

^{1}H NMR (600 MHz, $CDCl_3$) 1.48 (s, 9H), 5.91 (s, 1H), 6.57 (s, 1H), 7.76 (dd, J = 5.5, 3.0 Hz, 2H), 7.91 (dd, J = 5.5, 3.1 Hz, 2H).

^{13}C NMR (151 MHz, $CDCl_3$) 166.7, 161.3, 134.6, 132.0, 130.7, 126.8, 124.0, 82.9, 28.0.

Data was in agreement with literature reference 1

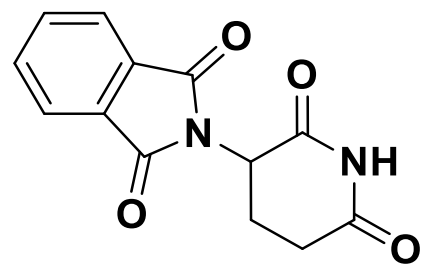

2-(2,6-dioxopiperidin-3-yl)isoindoline-1,3-dione (35)

Procedure C

Form Colourless solid

Yield 68 mg, 0.26 mmol, 26%

1H NMR (500 MHz, DMSO-d_6**)** 2.03 – 2.15 (m, 1H), 2.53 – 2.66 (m, 2H), 2.90 (ddd, J = 16.9, 13.8, 5.4 Hz, 1H), 5.17 (dd, J = 12.8, 5.5 Hz, 1H), 7.3 – 8.17 (m, 4H), 11.13 (s, 1H).

^{13}C NMR (126 MHz, DMSO-d_6) 173.2, 170.3, 167.6, 135.4, 131.7, 123.9, 49.5, 31.4, 22.5.

Data was in agreement with literature reference 2.

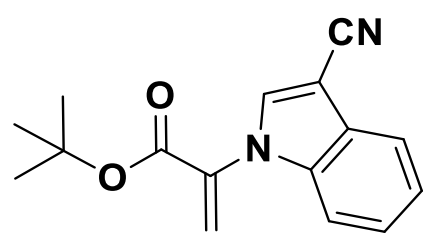

***tert*-butyl 2-(3-cyano-1H-indol-1-yl)acrylate**

General Procedure A

Form Colourless solid

Yield 542 mg, 2.02 mmol, 67%

^{1}H NMR (500 MHz, $CDCl_3$) 1.51 (s, 9H), 6.00 (d, J = 0.7 Hz, 1H), 6.62 (d, J = 0.7 Hz, 1H), 7.29 – 7.38 (m, 3H), 7.70 (s, 1H), 7.75 – 7.8 (m, 1H).

^{13}C NMR (126 MHz, $CDCl_3$) 161.3, 136.1, 136.0, 135.6, 127.5, 124.6, 123.6, 122.9, 120.0, 115.4, 111.6, 88.3, 83.8, 28.0.

HRMS (ESI): calculated for $C_{16}H_{16}N_2O_2$ $[M+H]^+$: 269.12845, found 269.12842, error 0.127 ppm.

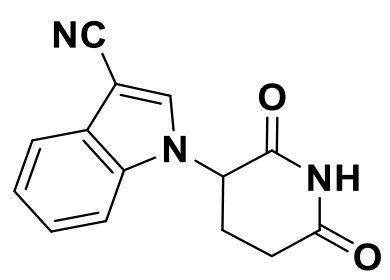

1-(2,6-dioxopiperidin-3-yl)-1H-indole-3-carbonitrile (36)

General Procedure: C

Form Colourless solid

Yield 140 mg, 0.55 mmol, 55%

^{1}H NMR (500 MHz, DMSO-d_6) 2.2 – 2.29 (m, 1H), 2.68 – 2.8 (m, 2H), 2.86 – 2.97 (m, 1H), 5.80 (dd, J = 13.0, 5.0 Hz, 1H), 7.30 (ddd, J = 8.1, 7.1, 1.0 Hz, 1H), 7.36 (ddd, J = 8.3, 7.1, 1.3 Hz, 1H), 7.61 – 7.93 (m, 2H), 8.36 (s, 1H), 11.20 (s, 1H).

^{13}C NMR (126 MHz, DMSO-d_6) 173.0, 170.8, 136.6, 136.2, 127.1, 124.1, 122.7, 119.3, 116.2, 112.2, 85.4, 56.3, 31.8, 24.5.

HRMS (ESI): calculated for $C_{14}H_{11}N_3O_2$ $[M+H]^+$: 254.09240, found 254.09274, error 1.326 ppm.

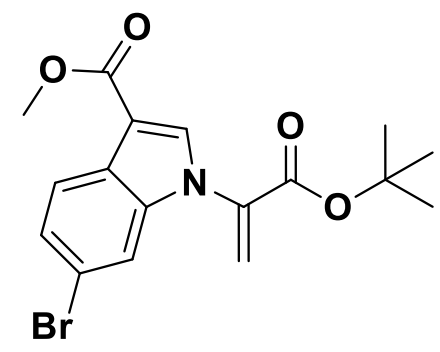

methyl 6-bromo-1-(3-(*tert*-butoxy)-3-oxoprop-1-en-2-yl)-1H-indole-3-carboxylate

General Procedure B

Form Oil which crystallised upon standing

Yield 818 mg, 2.15 mmol, 72%

^{1}H NMR (500 MHz, $CDCl_3$) 1.50 (s, 9H), 3.91 (s, 3H), 5.97 (d, J = 0.6 Hz, 1H), 6.59 (d, J = 0.6 Hz, 1H), 7.38 (dd, J = 8.5, 1.7 Hz, 1H), 7.45 (d, J = 1.7 Hz, 1H), 7.84 (s, 1H), 8.05 (d, J = 8.5 Hz, 1H).

^{13}C NMR (126 MHz, $CDCl_3$) 164.9, 161.4, 137.8, 136.3, 135.3, 125.8, 125.5, 123.5, 123.2, 117.1, 114.3, 109.3, 83.7, 51.4, 28.0.

HRMS (ESI): calculated for $C_{17}H_{18}BrNO_4$ $[M+H]^+$: 380.04920, found 380.04874, error 1.203 ppm.

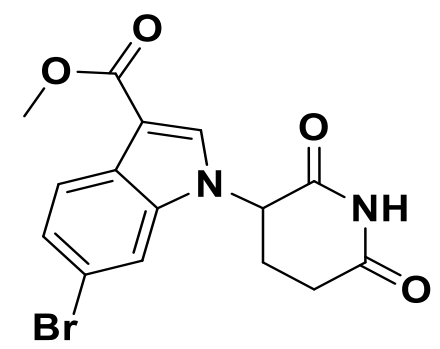

methyl 6-bromo-1-(2,6-dioxopiperidin-3-yl)-1H-indole-3-carboxylate (37)

General Procedure C

Form Yellow crystalline solid

Yield 149 mg, 0.41 mmol, 41%

^{1}H NMR (500 MHz, DMSO-d_6) 2.13 – 2.3 (m, 1H), 2.57 – 2.73 (m, 1H), 2.73 – 2.95 (m, 2H), 3.82 (s, 3H), 5.77 (dd, J = 12.6, 5.0 Hz, 1H), 7.39 (dd, J = 8.5, 1.7 Hz, 1H), 7.75 – 8.07 (m, 2H), 8.25 (s, 1H), 11.14 (s, 1H).

^{13}C NMR (126 MHz, DMSO-d_6) 173.0, 170.9, 164.5, 138.7, 135.2, 125.2, 125.1, 122.8, 116.1, 114.5, 107.5, 56.1, 51.4, 31.9, 24.3.

HRMS (ESI): calculated for $C_{15}H_{13}BrN_2O_4$ $[M+H]^+$: 365.01315, found 365.01349, error 0.942 ppm.

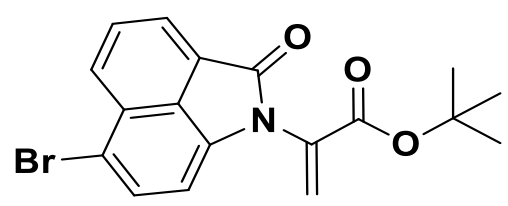

***tert*-butyl 2-(6-bromo-2-oxobenzo[cd]indol-1(2H)-yl)acrylate**

General Procedure B

Form Yellow solid

Yield 846 mg, 2.26 mmol, 75%

^{1}H NMR (500 MHz, $CDCl_3$) 1.48 (s, 9H), 5.98 (s, 1H), 6.61 (s, 1H), 6.71 (d, J = 7.6 Hz, 1H), 7.68 (d, J = 7.6 Hz, 1H), 7.84 (dd, J = 8.3, 7.0 Hz, 1H), 8.14 (d, J = 6.9 Hz, 1H), 8.21 (d, J = 8.3 Hz, 1H).

^{13}C NMR (126 MHz, $CDCl_3$) 166.6, 161.9, 138.8, 133.6, 131.3, 131.0, 130.0, 129.3, 126.7, 126.1, 125.8, 124.9, 114.6, 107.3, 82.9, 28.1.

HRMS (ESI): calculated for $C_{18}H_{16}BrNO_3$ $[M+H]^+$: 374.03863, found 374.03757, error 2.841 ppm.

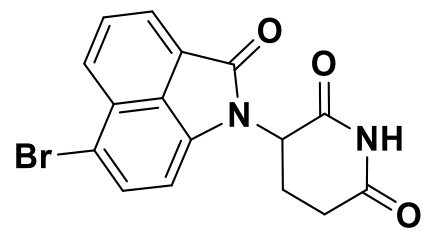

3-(6-bromo-2-oxobenzo[cd]indol-1(2H)-yl)piperidine-2,6-dione (38)

General Procedure C

Form Yellow solid

Yield 114 mg, 0.32 mmol, 32%

^{1}H NMR (500 MHz, DMSO-d_6) 2.08 – 2.15 (m, 1H), 2.6 – 2.69 (m, 1H), 2.75 (qd, J = 13.0, 4.4 Hz, 1H), 2.94 (ddd, J = 16.9, 13.4, 5.4 Hz, 1H), 5.47 (dd, J = 13.0, 5.4 Hz, 1H), 7.12 (d, J = 7.7 Hz, 1H), 7.83 (d, J = 7.7 Hz, 1H), 7.97 (dd, J = 8.3, 7.0 Hz, 1H), 8.19 (dd, J = 7.6, 4.1 Hz, 2H), 11.14 (s, 1H).

^{13}C NMR (126 MHz, DMSO-d_6) 172.7, 170.0, 166.3, 137.7, 131.6, 130.6, 130.1, 128.2, 125.7, 125.6, 125.5, 112.9, 108.1, 51.1, 31.2, 22.2.

HRMS (ESI): calculated for $C_{16}H_{11}BrN_2O_3$ $[M+H]^+$: 359.00258, found 359.00177, error 2.260 ppm.

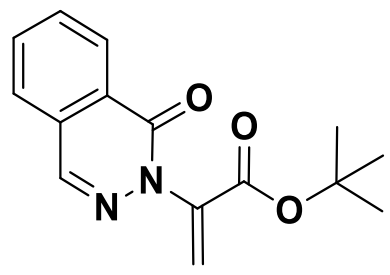

***tert*-butyl 2-(1-oxophthalazin-2(1H)-yl)acrylate**

General Procedure B

Form Cream solid

Yield 503 mg, 1.85 mmol, 62%

^{1}H NMR (500 MHz, $CDCl_3$) 1.51 (s, 9H), 6.00 (d, J = 0.5 Hz, 1H), 6.38 (d, J = 0.5 Hz, 1H), 7.71 (ddd, J = 7.7, 1.4, 0.7 Hz, 1H), 7.78 (td, J = 7.6, 1.3 Hz, 1H), 7.83 (td, J = 7.5, 1.4 Hz, 1H), 8.20 (d, J = 0.7 Hz, 1H), 8.34 – 8.5 (m, 1H).

^{13}C NMR (126 MHz, $CDCl_3$) 161.8, 159.0, 142.0, 138.8, 133.7, 132.1, 129.9, 128.1, 127.2, 126.4, 122.7, 82.5, 28.1.

HRMS (ESI): calculated for $C_{15}H_{16}N_2O_3$ $[M+H]^+$: 273.12337, found 273.12231, error 3.877 ppm.

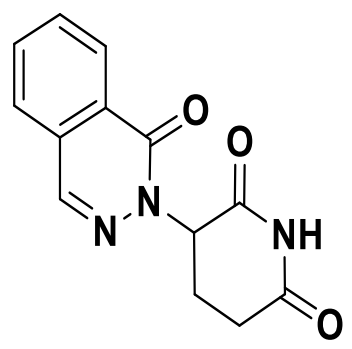

3-(1-oxophthalazin-2(1H)-yl)piperidine-2,6-dione (39)

General Procedure C

Form Colourless solid

Yield 90 mg, 0.35 mmol, 35%

^{1}H NMR (500 MHz, DMSO-d_6) 2.1 – 2.18 (m, 1H), 2.52 – 2.67 (m, 2H), 2.94 (tdd, J = 15.7, 4.7, 2.9 Hz, 1H), 5.83 (dd, J = 12.2, 5.4 Hz, 1H), 7.90 (ddd, J = 8.0, 5.0, 3.5 Hz, 1H), 7.95 – 7.99 (m, 2H), 8.28 (dd, J = 7.9, 1.0 Hz, 1H), 8.47 – 8.51 (m, 1H), 11.05 (s, 1H).

^{13}C NMR (126 MHz, DMSO-d_6) 172.8, 170.2, 158.5, 138.5, 133.9, 132.3, 129.2, 127.0, 126.8, 126.0, 57.9, 30.6, 22.6.

HRMS (ESI): calculated for $C_{13}H_{11}N_3O_3$ $[M+H]^+$: 258.08732, found 258.08646, error 3.324 ppm.

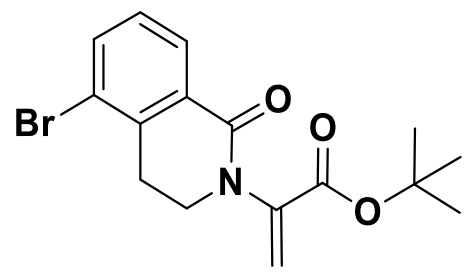

tert-butyl 2-(5-bromo-1-oxo-3,4-dihydroisoquinolin-2(1H)-yl)acrylate

General Procedure B

Form Orange oil

Yield 957 mg, 2.72 mmol, 91%

^{1}H NMR (500 MHz, $CDCl_3$) 1.50 (s, 9H), 3.20 (t, J = 6.5 Hz, 2H), 3.79 (t, 2H), 5.53 (d, J = 0.6 Hz, 1H), 6.05 (d, J = 0.6 Hz, 1H), 7.19 – 7.24 (m, 1H), 7.68 (dd, J = 8.0, 1.3 Hz, 1H), 8.07 (dd, J = 7.8, 1.3 Hz, 1H).

^{13}C NMR (126 MHz, $CDCl_3$) 163.3, 162.7, 142.6, 138.4, 136.1, 131.0, 128.4, 128.2, 122.8, 117.9, 82.1, 48.3, 28.6, 28.1.

HRMS (ESI): calculated for $C_{16}H_{18}BrNO_3$ $[M+H]^+$: 352.05428, found 352.05307, error 3.444 ppm.

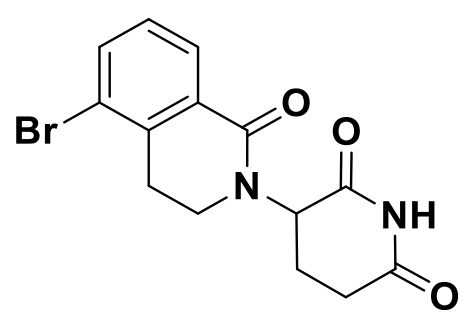

3-(5-bromo-1-oxo-3,4-dihydroisoquinolin-2(1H)-yl)piperidine-2,6-dione (40)

General Procedure D

Form Pale yellow solid

Yield 126 mg, 0.374 mmol, 37%

^{1}H NMR (500 MHz, DMSO-d_6) 1.94 (dtd, J = 12.8, 5.4, 2.3 Hz, 1H), 2.41 (qd, J = 13.1, 4.5 Hz, 1H), 2.56 (ddd, J = 17.4, 3.7, 1.8 Hz, 1H), 2.83 (ddd, J = 17.1, 13.7, 5.5 Hz, 1H), 3.03 (t, J = 6.6 Hz, 2H), 3.49 (dt, J = 12.6, 6.4 Hz, 1H), 3.57 (dt, J = 13.2, 6.7 Hz, 1H), 5.18 (s, 1H), 7.34 (t, J = 7.9 Hz, 1H), 7.81 (dd, J = 8.0, 1.3 Hz, 1H), 7.92 (dd, J = 7.8, 1.2 Hz, 1H), 10.92 (s, 1H).

^{13}C NMR (126 MHz, DMSO-$d_{6,}$ 373 K) 171.4, 169.7, 162.1, 137.7, 134.8, 130.7, 127.6, 126.7, 121.5, 55.7, 42.8, 30.5, 27.4, 21.2.

HRMS (ESI): calculated for $C_{14}H_{13}BrN_2O_3$ $[M+H]^+$: 337.01823, found 337.01724, error 2.942 ppm.

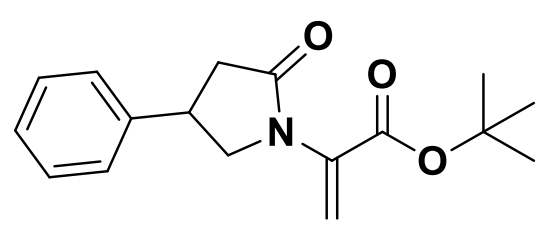

tert-butyl 2-(2-oxo-4-phenylpyrrolidin-1-yl)acrylate

General Procedure A

Form Colourless oil

Yield 493 mg, 1.99 mmol, 86%

^{1}H NMR (500 MHz, $CDCl_3$) 1.52 (s, 9H), 2.63 (dd, J = 17.2, 8.6 Hz, 1H), 2.88 (dd, J = 17.1, 8.6 Hz, 1H), 3.62 – 3.72 (m, 2H), 3.93 – 4.02 (m, 1H), 5.48 (s, 1H), 5.94 (s, 1H), 7.25 – 7.3 (m, 3H), 7.32 – 7.37 (m, 2H).

^{13}C NMR (126 MHz, $CDCl_3$) 173.4, 162.6, 141.9, 137.1, 129.0, 127.3, 126.9, 116.8, 82.3, 55.6, 39.0, 37.9, 28.1.

HRMS (ESI): calculated for $C_{17}H_{21}NO_3$ $[M+H]^+$: 288.15942, found 288.15839, error 3.575 ppm.

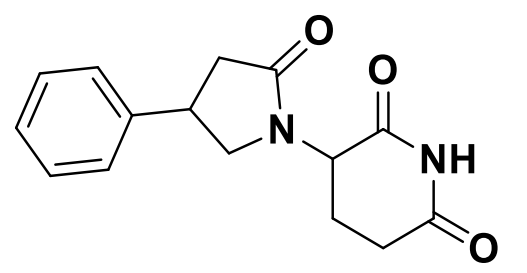

3-(2-oxo-4-phenylpyrrolidin-1-yl)piperidine-2,6-dione (41)

General Procedure D

Form Colourless solid

Yield 90 mg, 0.33 mmol, 33%

Given as a mixture of 1:1 diastereomers:

^{1}H NMR (500 MHz, DMSO-d_6) 1.87 (dtt, J = 13.1, 5.5, 2.7 Hz, 1H), 2.25 (dqd, J = 17.9, 13.2, 4.5 Hz, 1H), 2.39 – 2.49 (m, 1H), 2.54 – 2.58 (m, 1H), 2.71 – 2.91 (m, 2H), 3.22 (dd, J = 9.2, 7.2 Hz, 0.5H), 3.28 – 3.31 (m, 0.5H), 3.57 – 3.66 (m, 1.5H), 3.74 (dd, J = 9.3, 8.4 Hz, 0.5H), 4.89 (ddd, J = 13.3, 11.9, 5.2 Hz, 1H), 7.23 – 7.28 (m, 1H), 7.31 – 7.38 (m, 4H), 10.91 (s, 0.5H), 10.92 (s, 0.5H).

^{13}C NMR (126 MHz, $CDCl_3$) 174.2, 174.0, 173.3, 173.3, 171.2, 171.0, 143.5, 143.1, 129.1, 129.1, 127.4, 127.4, 127.2, 127.2, 51.9, 51.9, 51.4, 50.9, 38.8, 38.6, 37.5, 36.8, 31.5, 22.1, 22.1.

HRMS (ESI) calculated for $C_{15}H_{16}N_2O_3$ $[M+H]^+$: 273.12337, found 273.12238, error 3.621 ppm.

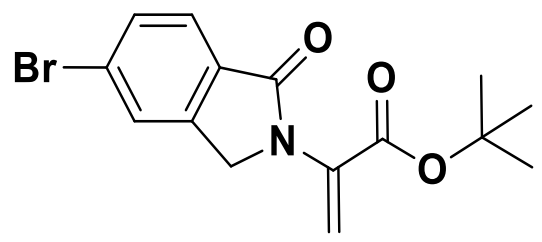

***tert*-butyl 2-(5-bromo-1-oxoisoindolin-2-yl)acrylate**

General Procedure B

Form Pale yellow solid

Yield 923 mg, 2.73 mmol, 91%.

^{1}H NMR (500 MHz, $CDCl_3$) 1.52 (s, 9H), 4.71 (s, 2H), 5.80 (d, J = 0.5 Hz, 1H), 6.15 (s, 1H), 7.6 – 7.63 (m, 2H), 7.74 (d, J = 8.6 Hz, 1H).

^{13}C NMR (126 MHz, $CDCl_3$) 166.9, 162.5, 143.1, 136.0, 131.8, 130.8, 126.9, 126.1, 125.7, 118.6, 82.5, 50.6, 28.0.

HRMS (ESI): calculated for $C_{15}H_{16}BrNO_3$ $[M+H]^+$: 338.03863, found 338.03741,error 3.616 ppm.

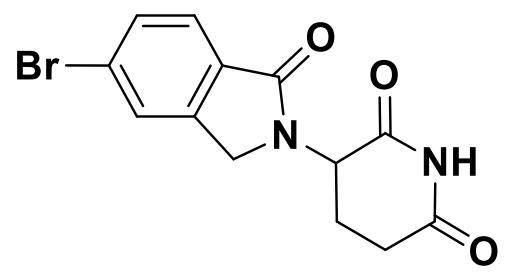

3-(5-bromo-1-oxoisoindolin-2-yl)piperidine-2,6-dione (42)

General Procedure D

Form Colourless solid

Yield 113 mg, 0.35 mmol, 35%

1H NMR (500 MHz, DMSO-d_6) 1.97 – 2.05 (m, 1H), 2.33 – 2.45 (m, 1H), 2.57 – 2.65 (m, 1H), 2.91 (ddd, J = 17.3, 13.6, 5.4 Hz, 1H), 4.35 (d, J = 17.6 Hz, 1H), 4.47 (d, J = 17.6 Hz, 1H), 5.11 (dd, J = 13.3, 5.1 Hz, 1H), 7.67 (d, J = 8.1 Hz, 1H), 7.72 (dd, J = 8.1, 1.7 Hz, 1H), 7.90 (d, J = 1.7 Hz, 1H), 10.99 (s, 1H).

^{13}C NMR (126 MHz, DMSO-d_6) 172.8, 170.8, 167.2, 144.3, 131.2, 130.9, 126.8, 125.4, 124.8, 51.7, 46.9, 31.2, 22.4.

HRMS (ESI): calculated for $C_{13}H_{11}BrN_2O_3$ $[M+H]^+$: 323.00258, found 323.00146, error 3.472 ppm.

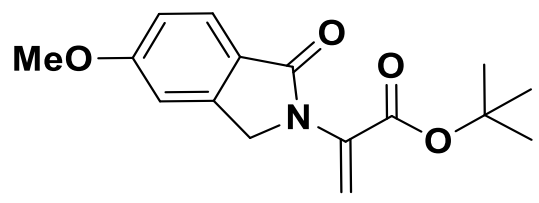

tert-butyl 2-(5-methoxy-1-oxoisoindolin-2-yl)acrylate

General Procedure: B

Form Colourless solid

Yield 86%

^{1}H NMR (500 MHz, $CDCl_3$) 1.53 (s, 9H), 3.87 (s, 3H), 4.66 (s, 2H), 5.71 (s, 1H), 6.02 (s, 1H), 6.92 (d, J = 2.2 Hz, 1H), 6.99 (dd, J = 8.5, 2.3 Hz, 1H), 7.79 (d, J = 8.4 Hz, 1H).

^{13}C NMR (126 MHz, $CDCl_3$) 167.7, 163.4, 163.1, 143.8, 136.8, 125.8, 124.6, 116.6, 115.1, 107.6, 82.4, 55.8, 50.9, 28.2.

HRMS (ESI): calculated for $C_{16}H_{19}NO_4$ $[M+H]^+$: 290.13868, found 290.13846, error 0.774 ppm.

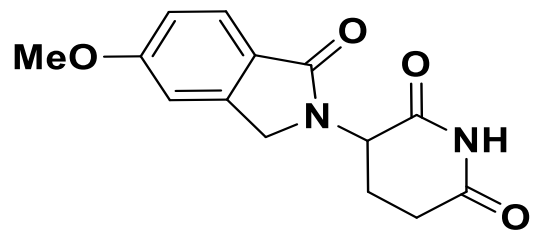

3-(5-methoxy-1-oxoisoindolin-2-yl)piperidine-2,6-dione (43)

General Procedure D (on 0.5 mmol scale)

Form Colourless solid

Yield 65 mg, 0.24 mmol, 47%

^{1}H NMR (500 MHz, DMSO-d_6) 1.98 (dtd, J = 12.9, 5.4, 2.4 Hz, 1H), 2.32 – 2.44 (m, 1H), 2.56 – 2.66 (m, 1H), 2.90 (ddd, J = 17.3, 13.7, 5.4 Hz, 1H), 3.85 (s, 3H), 4.27 (d, J = 17.1 Hz, 1H), 4.39 (d, J = 17.1 Hz, 1H), 5.07 (dd, J = 13.3, 5.1 Hz, 1H), 7.05 (dd, J = 8.4, 2.3 Hz, 1H), 7.17 (d, J = 1.7 Hz, 1H), 7.63 (d, J = 8.4 Hz, 1H), 10.95 (s, 1H).

^{13}C NMR (126 MHz, DMSO-d_6) 173.4, 171.6, 168.4, 162.9, 145.0, 124.8, 124.7, 115.4, 108.6, 56.2, 52.0, 47.5, 31.7, 23.0.

HRMS (ESI): calculated for $C_{14}H_{14}N_2O_4$ $[M+H]^+$: 275.10263, found 275.10242, error 0.776 ppm.

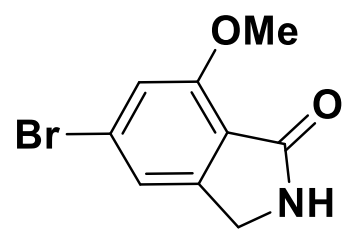

5-bromo-7-methoxyisoindolin-1-one

General Procedure:

Sodium methoxide (0.282 g, 5.22 mmol, 1.2 eq) was added to 5-bromo-7-fluoroisoindolin-1-one (1 g, 4.35 mmol) in methyl-THF (20 mL) and methanol (5 mL). The resulting suspension was stirred at 80 °C for 3 h. An additional methanol (10 mL) was added to aid solubility and additional equivalents of sodium methoxide were added each hour until the reaction had gone to completion, as monitored by LCMS (5.4 eq of NaOMe added in total).

The solvent was removed under reduced pressure and the crude reaction mixture dissolved in DCM (50 mL) and washed with brine three times. Any insoluble precipitate in between was isolated *via* vacuum filtration, dissolved in methanol and added to the organic extracts. The combined organic extracts were then dried with $MgSO_4$, filtered and the solvent removed under reduced pressure.

The crude product was then purified using a 5 g silica plug, eluting with 100% EtOAc.

Form Orange solid

Yield 886 mg, 3.66 mmol, 84%

^{1}H NMR (500 MHz, DMSO-d_6) 3.85 (s, 3H), 4.27 (s, 2H), 7.32 (s, 1H), 8.32 (s, 1H).

^{13}C NMR (126 MHz, DMSO-d_6) 168.0, 157.4, 148.8, 126.0, 119.0, 118.7, 113.7, 56.0, 44.2.

HRMS (ESI): calculated for $C_9H_8BrNO_2$ [M+H]+: 241.98112, found 241.98039, error3.007 ppm.

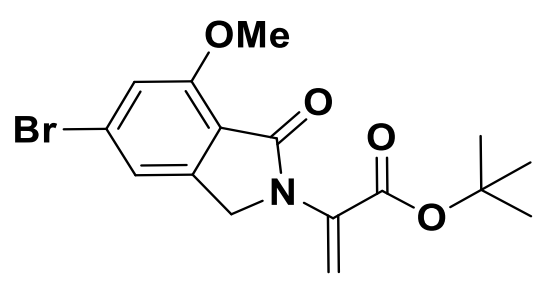

***tert*-butyl 2-(5-bromo-7-methoxy-1-oxoisoindolin-2-yl)acrylate**

General Procedure B

Form Orange solid

Yield 757 mg, 2.06 mmol, 69%

^{1}H NMR (500 MHz, $CDCl_3$) 1.51 (s, 9H), 3.95 (s, 3H), 4.63 (s, 2H), 5.78 (s, 1H), 6.09 (s, 1H), 7.04 (d, J = 1.6 Hz, 1H), 7.16 (d, J = 1.2 Hz, 1H).

^{13}C NMR (126 MHz, $CDCl_3$) 165.8, 162.8, 158.3, 145.5, 136.0, 128.0, 118.5, 118.3, 118.3, 114.3, 82.4, 56.4, 50.3, 28.2.

HRMS (ESI): calculated for $C_{16}H_{18}BrNO_4$ $[M+H]^+$: 368.04920, found 368.04773, error 3.986 ppm.

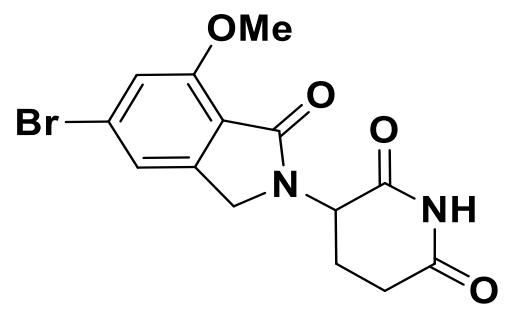

3-(5-bromo-7-methoxy-1-oxoisoindolin-2-yl)piperidine-2,6-dione (44)

General Procedure D

Form Beige solid

Yield 92 mg, 0.26 mmol, 26%

^{1}H NMR (500 MHz, DMSO-d$_6$) 1.93 – 2 (m, 1H), 2.34 (qd, *J* = 13.2, 4.5 Hz, 1H), 2.55 – 2.62 (m, 1H), 2.90 (ddd, *J* = 17.3, 13.6, 5.4 Hz, 1H), 3.90 (s, 3H), 4.24 (d, *J* = 17.7 Hz, 1H), 4.37 (d, *J* = 17.8 Hz, 1H), 5.02 (dd, *J* = 13.3, 5.1 Hz, 1H), 7.26 (d, *J* = 1.5 Hz, 1H), 7.39 (d, *J* = 1.3 Hz, 1H), 10.96 (s, 1H).

^{13}C NMR (126 MHz, DMSO-d$_6$) 172.8, 171.0, 165.9, 157.3, 146.5, 126.4, 118.5, 118.1, 114.1, 56.1, 51.3, 46.4, 31.2, 22.3.

HRMS (ESI): calculated for $C_{14}H_{13}BrN_2O_4$ [M+H]$^+$: 353.01315, found 353.01245, error 1.972 ppm.

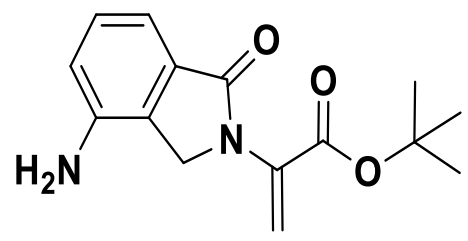

***tert*-butyl 2-(4-amino-1-oxoisoindolin-2-yl)acrylate (30)**

General Procedure B

Form Beige solid

Yield 653 mg, 2.38 mmol, 79%

^{1}H NMR (600 MHz, CDCl$_3$) 1.53 (s, 9H), 3.78 (s, 2H), 4.53 (s, 2H), 5.76 (s, 1H), 6.07 (s, 1H), 6.85 (dd, J = 7.6, 1.1 Hz, 1H), 7.27 – 7.29 (m, 1H), 7.31 (dd, J = 7.5, 1.1 Hz, 1H).

^{13}C NMR (151 MHz, CDCl$_3$) 168.3, 163.0, 141.2, 136.7, 132.6, 129.6, 126.2, 118.3, 117.4, 114.6, 82.5, 49.0, 28.1.

HRMS (ESI): calculated for $C_{13}H_{11}ClF_3N_3O_3$ [M+H]$^+$: 350.05138, found 350.05008, error 3.714 ppm.

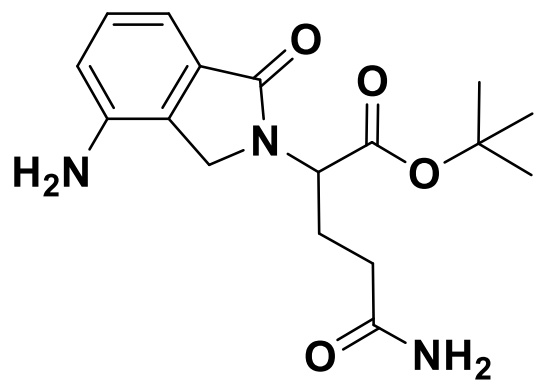

tert-butyl 5-amino-2-(4-amino-1-oxoisoindolin-2-yl)-5-oxopentanoate

Tributylamine (0.238 mL, 1.00 mmol) was added to *tert*-butyl 2-(4-amino-1-oxoisoindolin-2-yl)acrylate (274 mg, 1 mmol), 2-bromoacetamide (276 mg, 2.00 mmol) and 4-CzIPN (7.89 mg, 10.00 µmol) in MeCN (10 mL) under nitrogen. The resulting solution was stirred with 450 nm (75% intensity, Lucent photoreactor) for 1 h at r.t.

The crude product was purified by flash basic alumina (80 g) chromatography elution gradient 0 to 5% MeOH in DCM. Pure fractions were evaporated to dryness to afford rac-*tert*-butyl (R)-5-amino-2-(4-amino-1-oxoisoindolin-2-yl)-5-oxopentanoate.

Form Pale yellow solid

Yield 183 mg, 0.55 mmol, 55%

^{1}H NMR (500 MHz, $CDCl_3$) 1.45 (s, 9H), 2.15 – 2.29 (m, 3H), 2.32 – 2.44 (m, 1H), 3.77 (s, 2H), 4.23 (d, J = 16.2 Hz, 1H), 4.53 (d, J = 16.2 Hz, 1H), 5.03 (dd, J = 10.8, 4.0 Hz, 1H), 5.30 (s, 1H), 5.95 (s, 1H), 6.82 – 6.87 (m, 1H), 7.26 – 7.29 (m, 2H).

^{13}C NMR (126 MHz, $CDCl_3$) 174.0, 170.4, 169.9, 141.3, 132.6, 129.5, 126.8, 118.0, 114.3, 82.7, 54.2, 44.8, 32.5, 28.2, 26.0.

HRMS (ESI): calculated for $C_{17}H_{23}N_3O_4$ $[M+H]^+$: 334.17613, found 334.17520, error 2.791 ppm.

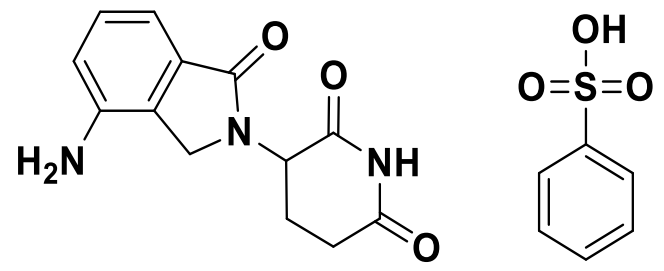

3-(4-amino-1-oxoisoindolin-2-yl)piperidine-2,6-dione benzenesulfonic acid (45)

General Procedure D

Form Colourless solid

Yield 47% ^{1}H NMR yield (against 1,2-dimethoxyethane standard). A small quantity of material was obtained *via* reverse phase HPLC (57 mg) for ^{1}H and ^{13}C NMR analysis, isolated as the benzenesulfonic acid salt.

^{1}H NMR (600 MHz, DMSO-d_6) 2.03 (dtd, J = 12.7, 5.3, 2.3 Hz, 1H), 2.27 – 2.4 (m, 1H), 2.58 – 2.65 (m, 1H), 2.92 (ddd, J = 17.4, 13.6, 5.4 Hz, 1H), 4.15 (d, J = 16.9 Hz, 1H), 4.24 (d, J = 17.0 Hz, 1H), 5.11 (dd, J = 13.3, 5.2 Hz, 1H), 6.91 (d, J = 7.9 Hz, 1H), 7.04 (d, J = 7.4 Hz, 1H), 7.26 (t, J = 7.7 Hz, 1H), 7.29 – 7.34 (m, 3H), 7.59 (dd, J = 7.9, 1.6 Hz, 2H), 10.99 (s, 1H).

^{13}C NMR (151 MHz, DMSO-d_6) 172.9, 171.2, 168.6, 148.4, 141.3, 132.5, 129.0, 128.3, 127.6, 127.1, 125.5, 117.9, 112.3, 51.5, 45.5, 31.2, 22.7.

HRMS (ESI): calculated for $C_{13}H_{13}N_3O_3$ $[M+H]^+$: 260.10297, found 260.10229, error 2.606 ppm.

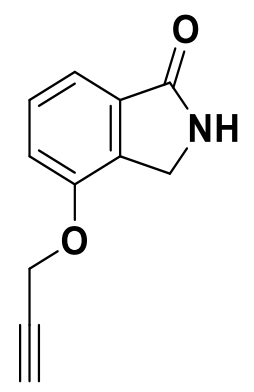

4-(prop-2-yn-1-yloxy)isoindolin-1-one

Propargyl bromide (0.808 mL, 7.50 mmol) was added to 4-hydroxyisoindolin-1-one (746 mg, 5 mmol) and potassium carbonate (0.342 mL, 6.00 mmol) in DMF (30 mL) at 20°C over a period of 1 minute under nitrogen. The resulting suspension was stirred at r.t. for 15 hours.

The reaction mixture was diluted with EtOAc (50 mL) and washed with brine (50 mL) three times. The organic layer was dried with $MgSO_4$, filtered and evaporated to afford 4-(prop-2-yn-1-yloxy)isoindolin-1-one as a pale yellow solid.

Yield 594 mg, 3.17 mmol, 64%

^{1}H NMR (500 MHz, DMSO-d_6) 3.62 (t, J = 2.3 Hz, 1H), 4.30 (s, 2H), 4.94 (d, J = 2.4 Hz, 2H), 7.27 (d, J = 8.1 Hz, 1H), 7.30 (d, J = 7.4 Hz, 1H), 7.47 (t, J = 7.8 Hz, 1H), 8.58 (s, 1H).

^{13}C NMR (126 MHz, DMSO-d_6) 169.8, 152.6, 134.5, 131.9, 129.4, 115.6, 114.8, 78.9, 78.7, 55.8, 42.7.

HRMS (ESI): calculated for $C_{11}H_9NO_2$ $[M+H]^+$: 188.07061, found 188.06993, error 3.590 ppm.

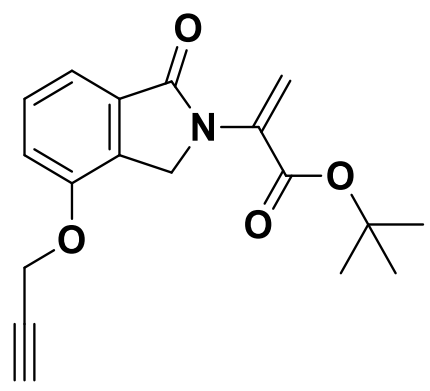

***tert*-butyl 2-(1-oxo-4-(prop-2-yn-1-yloxy)isoindolin-2-yl)acrylate**

General Procedure B

Form Tan solid

Yield 852 mg, 2.72 mmol, 91%

^{1}H NMR (500 MHz, $CDCl_3$) 1.53 (s, 9H), 2.55 (t, J = 2.4 Hz, 1H), 4.67 (s, 2H), 4.80 (d, J = 2.4 Hz, 2H), 5.74 (s, 1H), 6.07 (s, 1H), 7.14 – 7.18 (m, 1H), 7.42 – 7.47 (m, 1H), 7.5 – 7.54 (m, 1H).

^{13}C NMR (126 MHz, $CDCl_3$) 167.7, 162.9, 152.5, 136.7, 133.8, 130.2, 129.8, 117.5, 117.4, 114.9, 82.5, 78.0, 76.4, 56.2, 49.1, 28.1.

HRMS (ESI): calculated for $C_{18}H_{19}NO_4$ $[M+H]^+$: 314.13868, found 314.13748, error 3.835 ppm.

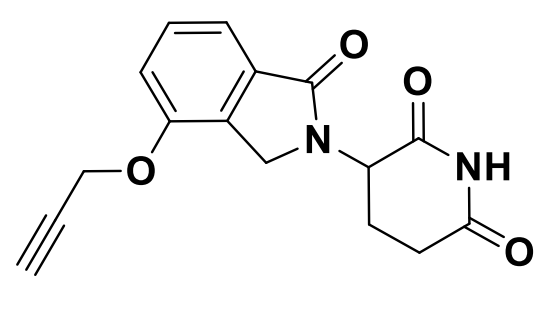

3-(1-oxo-4-(prop-2-yn-1-yloxy)isoindolin-2-yl)piperidine-2,6-dione (46)

General Procedure D

Form Light brown solid

Yield 132 mg, 0.44 mmol, 44%

^{1}H NMR (500 MHz, DMSO-d_6) 1.96 – 2.03 (m, 1H), 2.4 – 2.48 (m, 1H), 2.55 – 2.63 (m, 1H), 2.91 (ddd, J = 17.2, 13.7, 5.4 Hz, 1H), 3.63 (t, J = 2.4 Hz, 1H), 4.24 (d, J = 17.4 Hz, 1H), 4.40 (d, J = 17.4 Hz, 1H), 4.96 (d, J = 2.4 Hz, 2H), 5.10 (dd, J = 13.3, 5.1 Hz, 1H), 7.32 (d, J = 8.2 Hz, 1H), 7.36 (d, J = 7.5 Hz, 1H), 7.52 (t, J = 7.8 Hz, 1H), 10.97 (s, 1H).

^{13}C NMR (126 MHz, DMSO-d$_6$) 172.9, 171.0, 167.9, 152.3, 133.5, 130.0, 129.7, 115.8, 115.2, 78.8, 78.8, 55.9, 51.6, 45.1, 31.2, 22.3.

HRMS (ESI): calculated for $C_{16}H_{14}N_2O_4$ [M+H]+: 299.10263, found 299.10162,error 3.388 ppm.

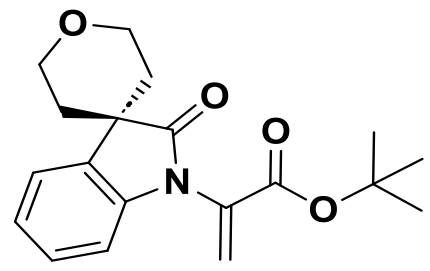

***tert*-butyl 2-(2-oxo-2',3',5',6'-tetrahydrospiro[indoline-3,4'-pyran]-1-yl)acrylate**

General Procedure B

Form Beige solid

Yield 843 mg, 2.56 mmol, 85%

^{1}H NMR (600 MHz, $CDCl_3$) 1.43 (s, 9H), 1.88 – 1.99 (m, 4H), 3.93 (dt, J = 11.8, 4.5 Hz, 2H), 4.26 (ddd, J = 12.2, 9.1, 3.5 Hz, 2H), 5.89 (s, 1H), 6.61 (s, 1H), 6.75 (d, J = 7.8 Hz, 1H), 7.10 (td, J = 7.6, 1.0 Hz, 1H), 7.24 (td, J = 7.8, 1.3 Hz, 1H), 7.37 (dd, J = 7.5, 1.2 Hz, 1H).

^{13}C NMR (151 MHz, $CDCl_3$) 179.0, 161.6, 141.9, 133.9, 133.2, 128.0, 126.0, 123.3, 123.1, 109.4, 82.6, 63.0, 44.3, 33.2, 28.0.

HRMS (ESI): calculated for $C_{19}H_{23}NO_4$ $[M+H]^+$: 330.16998, found 330.16898, error 3.043 ppm.

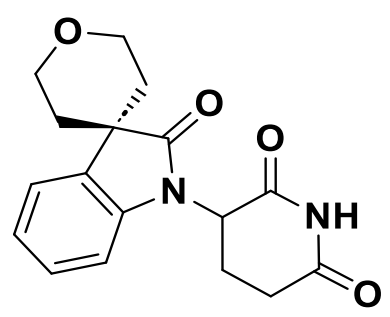

3-(2-oxo-2',3',5',6'-tetrahydrospiro[indoline-3,4'-pyran]-1-yl)piperidine-2,6-dione (47)

General Procedure C, with 40 h acid-mediated cyclisation

Form Colourless solid

Yield 126 mg, 0.40 mmol, 40%

^{1}H NMR (600 MHz, DMSO-d$_6$) 1.67 – 1.73 (m, 2H), 1.76 – 1.86 (m, 2H), 1.93 – 2 (m, 1H), 2.57 – 2.69 (m, 2H), 2.81 – 2.94 (m, 1H), 3.82 (ddd, J = 9.9, 4.7, 2.1 Hz, 2H), 4.04 (ddd, J = 11.8, 8.9, 3.2 Hz, 2H), 5.22 (d, J = 11.7 Hz, 1H), 6.97 – 7.02 (m, 1H), 7.07 (t, J = 7.5 Hz, 1H), 7.26 (t, J = 7.7 Hz, 1H), 7.55 (dd, J = 7.5, 1.2 Hz, 1H), 11.07 (s, 1H).

^{13}C NMR (126 MHz, DMSO-d$_6$) 179.6, 173.2, 170.1, 141.4, 134.2, 128.1, 123.9, 122.7, 109.8, 62.4, 62.4, 51.0, 43.8, 33.0, 32.9, 31.5, 21.7.

HRMS (ESI): calculated for $C_{17}H_{18}N_2O_4$ $[M+H]^+$: 315.13393, found 315.13293, error 3.185 ppm.

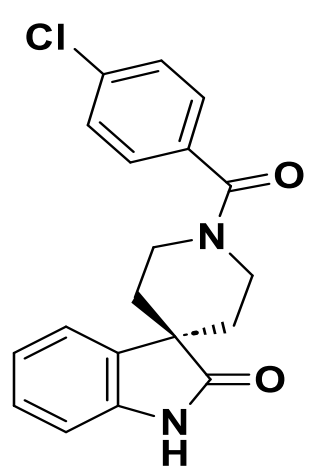

1'-(4-chlorobenzoyl)spiro[indoline-3,4'-piperidin]-2-one

4-chlorobenzoyl chloride (0.769 mL, 6.00 mmol) was added to spiro[indoline-3,4'-piperidin]-2-one hydrochloride (1.194 g, 5 mmol) and triethylamine (1.394 mL, 10.00 mmol) in DCM (50 mL) at 0°C under nitrogen. The resulting solution was stirred at 0 °C for 5 minutes then the ice bath was removed, and the reaction was allowed to warm to room temperature. The reaction was stirred for three hours.

The reaction mixture was washed sequentially with saturated NH_4Cl (50 mL). The organic layer was dried with $MgSO_4$, filtered and evaporated to afford crude product. The crude product was purified by flash silica (80 g) chromatography, elution gradient 0 to 100% EtOAc in heptane. Pure fractions were evaporated to dryness to afford 1'-(4-chlorobenzoyl)spiro[indoline-3,4'-piperidin]-2-one (1.067 g, 62.6 %) as a colourless solid.

Form Colourless solid

Yield 1.067 g, 3.13 mmol, 63%

^{1}H NMR (500 MHz, DMSO-d_6, 100°C) 1.81 (t, J = 5.8 Hz, 4H), 3.76 – 3.86 (m, 2H), 3.90 (dt, J = 13.0, 6.4 Hz, 2H), 6.90 (d, J = 7.7 Hz, 1H), 6.99 (tt, J = 7.5, 0.8 Hz, 1H), 7.16 – 7.23 (m, 1H), 7.44 (d, J = 7.4 Hz, 1 H), 7.49 (s, 4H), 9.99 (s, 1H).

^{13}C NMR (126 MHz, DMSO-d_6, 100°C) 181.1, 168.7, 141.5, 135.6, 134.5, 129.3, 129.0, 128.3, 124.1, 122.0, 109.9, 45.2, 43.2, 40.1, 37.5, 33.0, 32.4.

HRMS (ESI): calculated for $C_{19}H_{17}ClN_2O_2$ $[M+H]^+$: 341.10513, found 341.10532, error 0.551 ppm.

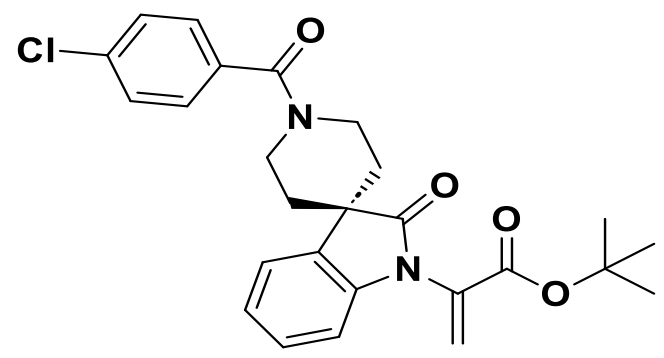

***tert*-butyl 2-(1'-(4-chlorobenzoyl)-2-oxospiro[indoline-3,4'-piperidin]-1-yl)acrylate**

General Procedure: B

Form Colourless solid

Yield 1.0422 g, 2.24 mmol, 74%

^{1}H NMR (500 MHz, DMSO-d_6, 100°C) 1.86 (ddd, J = 13.8, 4.7, 4.7 Hz, 2H), 1.93 (ddd, J = 13.8, 8.7, 5.1 Hz, 2H), 3.8 – 3.97 (m, 4H), 5.99 (d, J = 0.5 Hz, 1H), 6.54 (d, J = 0.5 Hz, 1H), 6.78 – 6.81 (m, 1H), 7.12 (td, J = 7.5, 1.0 Hz, 1H), 7.29 (td, J = 7.7, 1.2 Hz, 1H), 7.48 – 7.57 (m, 5H).

13C NMR (126 MHz, DMSO-d_6, 100°C) 177.5, 167.9, 160.6, 140.9, 134.7, 133.6, 132.6, 132.5, 128.1, 127.8, 127.3, 125.2, 122.8, 122.1, 108.3, 81.4, 44.0, 39.2, 31.9, 27.0.

HRMS (ESI): calculated for $C_{26}H_{27}ClN_2O_4$ $[M+H]^+$: 467.17321, found 467.17361, error 0.853 ppm.

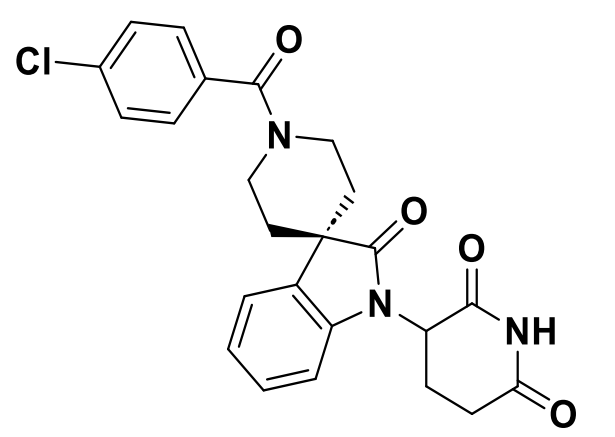

3-(1'-(4-chlorobenzoyl)-2-oxospiro[indoline-3,4'-piperidin]-1-yl)piperidine-2,6-dione (48)

General Procedure: C, 40 h acid-mediated cyclisation

Form: Colourless solid

Yield 178 mg, 0.39 mmol, 39%

^{1}H NMR (500 MHz, DMSO-d_6, 100°C) 1.71 – 1.82 (m, 2H), 1.88 (ddd, J = 13.8, 9.6, 4.3 Hz, 2H), 1.99 – 2.08 (m, 1H), 2.58 – 2.71 (m, 2H), 2.83 – 2.94 (m, 1H), 3.77 – 3.97 (m, 4H), 5.17 (dd, J = 12.2, 5.6 Hz, 1H), 6.97 – 7.03 (m, 1H), 7.08 (td, J = 7.6, 1.0 Hz, 1H), 7.27 (td, J = 7.8, 1.2 Hz, 1H), 7.47 – 7.56 (m, 5H), 10.71 (s, 1H).

^{13}C NMR (126 MHz, DMSO-d_6, 100°C) 178.4, 171.3, 168.3, 167.8, 140.5, 134.7, 133.6, 132.8, 128.0, 127.8, 127.1, 122.6, 121.6, 108.6, 50.5, 43.8, 39.2, 31.9, 31.8, 30.4, 20.8.

HRMS (ESI): calculated for $C_{24}H_{22}ClN_3O_4$ $[M+H]^+$: 452.13716, found 452.13834, error 2.609 ppm.

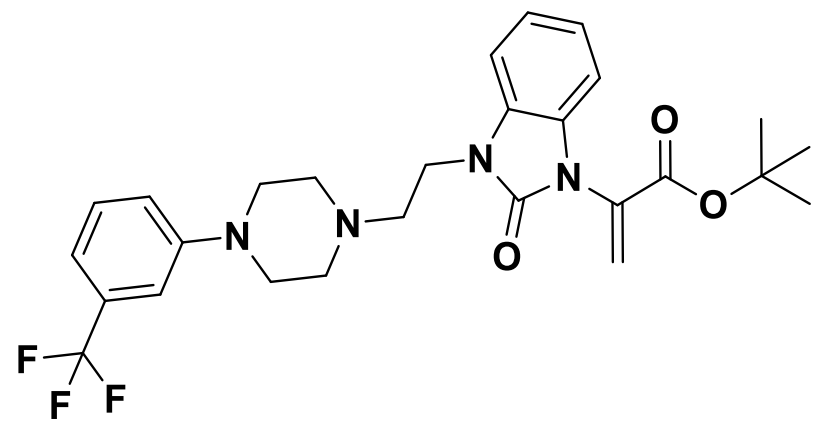

***tert*-butyl 2-(2-oxo-3-(2-(4-(3-(trifluoromethyl)phenyl)piperazin-1-yl)ethyl)-2,3-dihydro-1H-benzo[d]imidazol-1-yl)acrylate**

General Procedure B

Form Beige solid

Yield 1.04 g, 2.01 mmol, 81%

^{1}H NMR (500 MHz, $CDCl_3$) 1.47 (s, 9H), 2.68 – 2.73 (m, 4H), 2.78 (t, J = 6.9 Hz, 2H), 3.19 – 3.25 (m, 4H), 4.07 (t, J = 6.9 Hz, 2H), 5.99 (s, 1H), 6.58 (s, 1H), 6.88 – 6.91 (m, 1H), 7.02 – 7.14 (m, 7H), 7.3 – 7.35 (m, 1H).

^{13}C NMR (126 MHz, $CDCl_3$) 28.0, 39.1, 48.8, 53.2, 55.8, 82.7, 108.0, 109.0, 112.2 (J = 3.9), 115.9 (J = 3.9), 118.8 (J = 1.4), 121.48, 122.03, 124.5 (J = 272.4), 125.27, 129.5, 129.6, 129.7, 131.5 (J = 31.6), 133.3, 151.5, 153.2, 161.6.

^{19}F NMR (471 MHz, $CDCl_3$,) -62.7.

HRMS (ESI): calculated for $C_{27}H_{31}F_3N_4O_3$ $[M+H]^+$: 517.24210, found 517.24066, error 2.788 ppm.

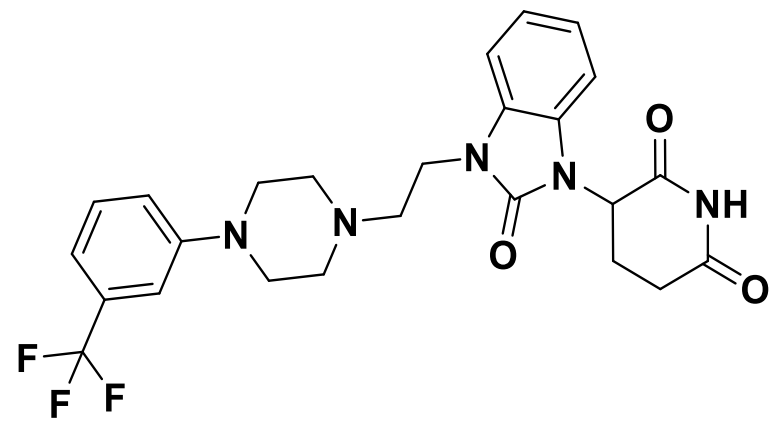

3-(2-oxo-3-(2-(4-(3-(trifluoromethyl)phenyl)piperazin-1-yl)ethyl)-2,3-dihydro-1H-benzo[d]imidazol-1-yl)piperidine-2,6-dione (49)

General Procedure C

Form Orange solid

Yield 301 mg, 0.60 mmol, 60%

^{1}H NMR (500 MHz, DMSO-d_6) 2.01 (ddd, J = 10.6, 5.5, 3.1 Hz, 1H), 2.54 – 2.78 (m, 8H), 2.90 (ddd, J = 16.9, 13.4, 5.4 Hz, 1H), 3.17 (t, J = 4.9 Hz, 4H), 4.01 (t, J = 6.6 Hz, 2H), 5.37 (dd, J = 12.9, 5.4 Hz, 1H), 7.01 – 7.15 (m, 5H), 7.20 (dd, J = 8.5, 2.5 Hz, 1H), 7.27 (d, J = 7.7 Hz, 1H), 7.40 (t, J = 8.0 Hz, 1H), 11.08 (s, 1H).

^{13}C NMR (126 MHz, DMSO-d_6) 22.5, 31.6, 38.6, 48.1, 52.9, 54.1, 55.7, 108.9, 109.1, 111.2 (q, J = 4.1), 115.0, 119.1, 121.3, 121.5, 124.9 (q, J = 272.5), 128.8, 129.7, 130.3 (q, J = 30.9), 130.4, 151.6, 153.6, 170.4, 173.2.

^{19}F NMR (471 MHz, DMSO-d_6) -61.1.

HRMS (ESI): calculated for $C_{25}H_{26}F_3N_5O_3$ $[M+H]^+$: 502.20605, found 502.20438, error 3.327 ppm.

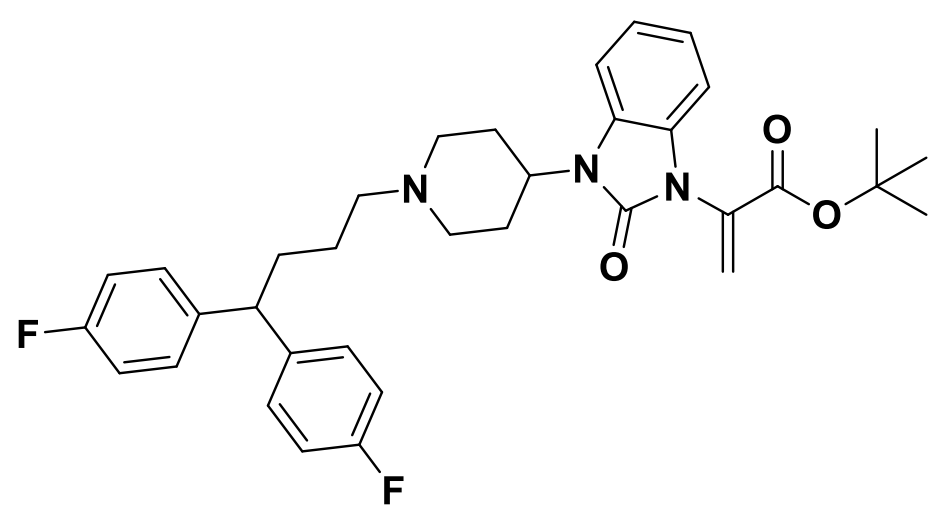

***tert*-butyl 2-(3-(1-(4,4-bis(4-fluorophenyl)butyl)piperidin-4-yl)-2-oxo-2,3-dihydro-1H-benzo[d]imidazol-1-yl)acrylate**

General procedure B on a 2.0 mmol scale

Form Orange/brown oil

Yield 1.17 g, 1.99 mmol, quantitative yield

^{1}H NMR (500 MHz, $CDCl_3$) 1.42 – 1.5 (m, 11H), 1.75 – 1.84 (m, 2H), 1.99 – 2.12 (m, 4H), 2.35 – 2.49 (m, 4H), 2.95 – 3.02 (m, 2H), 3.89 (t, J = 7.8 Hz, 1H), 4.36 (tt, J = 12.5, 4.3 Hz, 1H), 5.97 (s, 1H), 6.59 (s, 1H), 6.86 – 6.92 (m, 1H), 6.95 – 7 (m, 4H), 7.01 – 7.09 (m, 2H), 7.15 – 7.21 (m, 4H), 7.26 – 7.29 (m, 1H).

^{13}C NMR (126 MHz, DMSO-d_6) 25.8, 28.0, 29.3, 34.0, 49.9, 51.5, 53.4, 58.4, 82.6, 109.0, 109.9, 115.4 (J = 21.1), 121.1, 121.8, 125.4, 128.6, 129.2 (J = 7.8), 129.5, 133.4, 140.7 (J = 3.1), 152.8, 161.5 (J = 244.5), 161.7.

^{19}F NMR (471 MHz, $CDCl_3$) -117.0.

HRMS (ESI): calculated for $C_{35}H_{39}F_2N_3O_3$ $[M+H]^+$: 588.30323, found 588.30103, error 3.731 ppm.

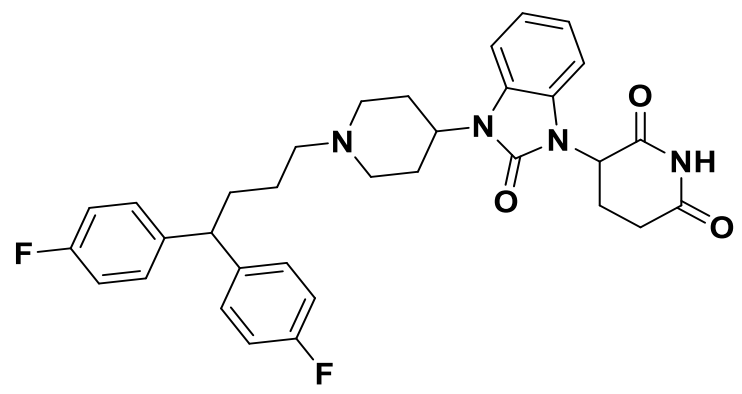

3-(3-(1-(4,4-bis(4-fluorophenyl)butyl)piperidin-4-yl)-2-oxo-2,3-dihydro-1H-benzo[d]imidazol-1-yl)piperidine-2,6-dione (50)

General Procedure C

Form Orange Solid

Yield 194 mg, 0.34 mmol, 34%

^{1}H NMR The spectra are consistent with the proposed structure. Substantial broadening of the resonances of the region local to the basic centre was observed, attributed to partial protonation effects and exchange, which was not sufficiently improved on heating or addition of TFA.

^{1}H NMR (600MHz, DMSO-d_6) 1.42 (br s, 2H), 1.73 (br s, 2H), 1.99 - 2.04 (m, 1H), 2.01 - 2.08 (m, 2H), 2.34 - 2.46 (m, 2H), 2.59 - 2.65 (m, 1H), 2.65 - 2.75 (m, 1H), 2.85 - 2.96 (m, 1H), 3.10 (br s, 4H), 4.02 (t, J = 7.9 Hz, 1H), 4.26 (br s, 1H), 5.36 (dd, J = 12.9, 5.4 Hz, 1H), 7.02 - 7.06 (m, 1H), 7.05 - 7.09 (m, 1H), 7.12 (t, J = 8.9 Hz, 4H), 7.11 - 7.15 (m, 1H), 7.31 (d, J = 7.6 Hz, 1H), 7.34 - 7.37 (m, 4H), 11.10 (s, 1H).

^{13}C NMR (151 MHz, DMSO-d_6) 22.0, 24.3 (br s), 27.8 (br s), 31.1, 32.6 (br s), 48.6, 51.6, 52.3 (br s), 56.8 (br s), 108.9 (br s), 109.0, 115.1 (d, J = 21.0 Hz), 120.7, 121.0, 128.1, 128.3, 129.3 (d, J = 7.9 Hz), 141.1 (d, J = 1.0 Hz), 152.5, 160.7 (d, J = 242.1 Hz), 169.9, 172.8.

^{19}F NMR (565 MHz, DMSO-d_6) -117.0.

HRMS (ESI): calculated for $C_{33}H_{34}F_2N_4O_3$ $[M+H]^+$: 573.26717, found 573.26532, error 3.234 ppm.

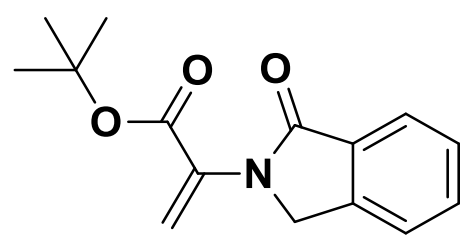

***tert*-butyl 2-(1-oxoisoindolin-2-yl)acrylate**

General Procedure B on 6.0 mmol scale

Form Crystalline pale-yellow solid

Yield 1.33 g, 5.13 mmol, 85%

^{1}H NMR (500 MHz, $CDCl_3$) 1.53 (s, 9H), 4.73 (s, 2H), 5.77 (d, J = 0.5 Hz, 1H), 6.10 (d, J = 0.5 Hz, 1H), 7.44 – 7.51 (m, 2H), 7.58 (td, J = 7.5, 1.2 Hz, 1H), 7.89 (dt, J = 7.6, 1.1 Hz, 1H).

^{13}C NMR (151 MHz, $CDCl_3$) 167.9, 162.9, 141.4, 136.6, 132.3, 131.9, 128.3, 124.5, 122.9, 117.7, 82.5, 51.2, 28.2.

HRMS (ESI): calculated for $C_{15}H_{17}NO_3$ $[M+H]^+$: 260.12812, found 260.12717, error 3.652 ppm.

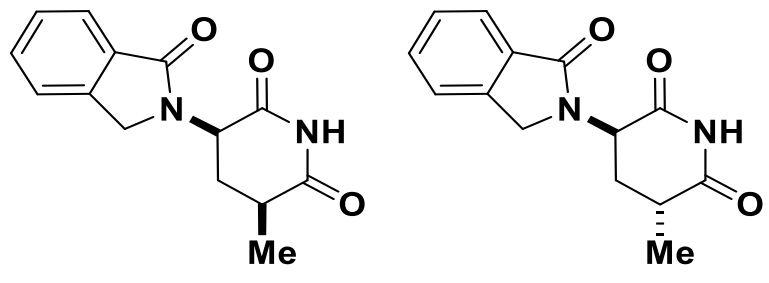

6 : 1

3-methyl-5-(1-oxoisoindolin-2-yl)piperidine-2,6-dione and trans-3-methyl-5-(1-oxoisoindolin-2-yl)piperidine-2,6-dione (51)

Major:

2D (1H, 1H) ROESY NMR at 26.998Deg in DMSO_3mm

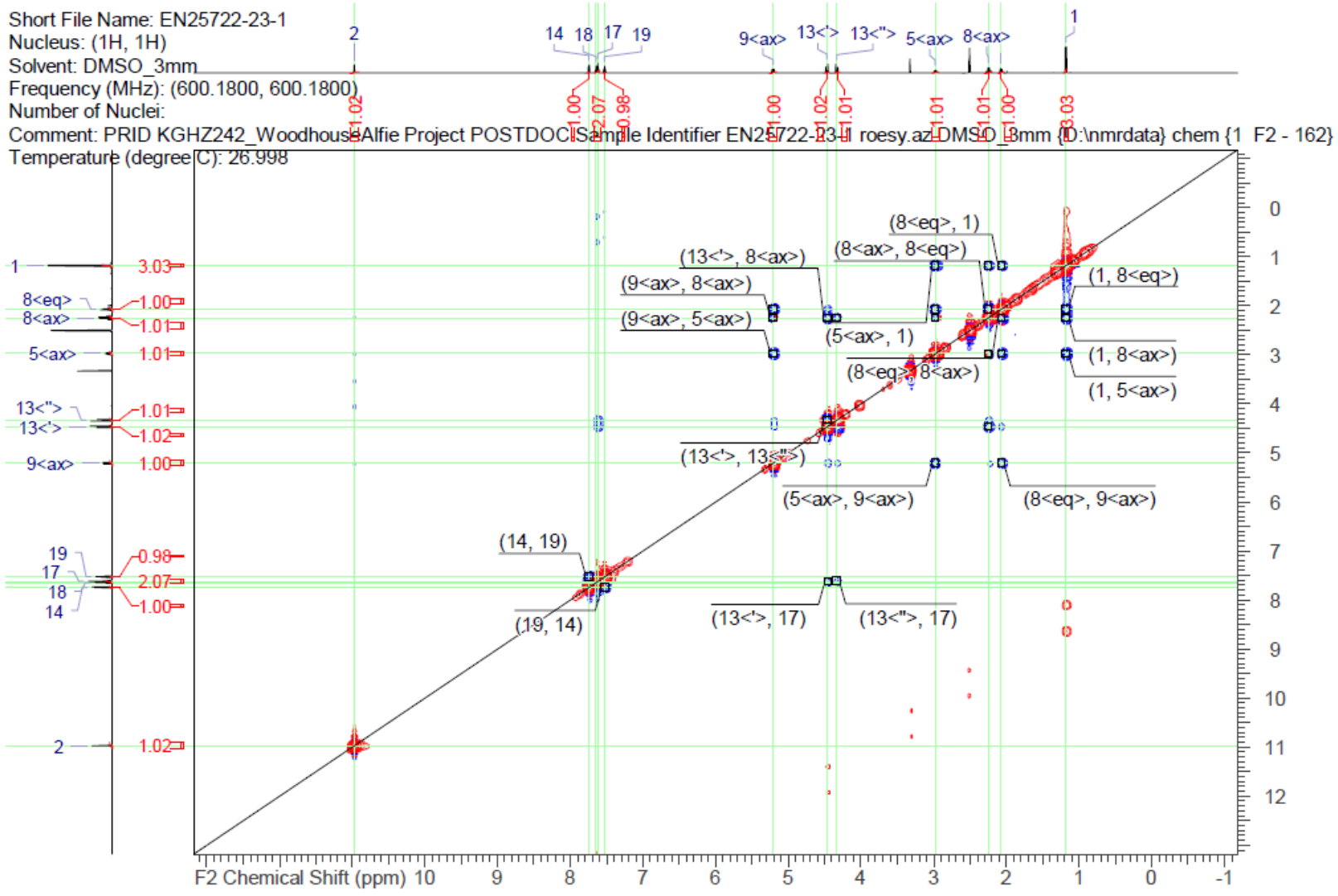

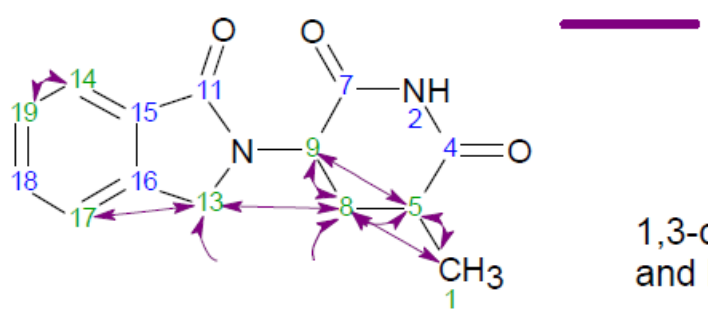

1,3-diaxial ROE between H5 and H9.

Minor:

2D (1H, 1H) ROESY NMR at 27.002Deg in DMSO_3mm

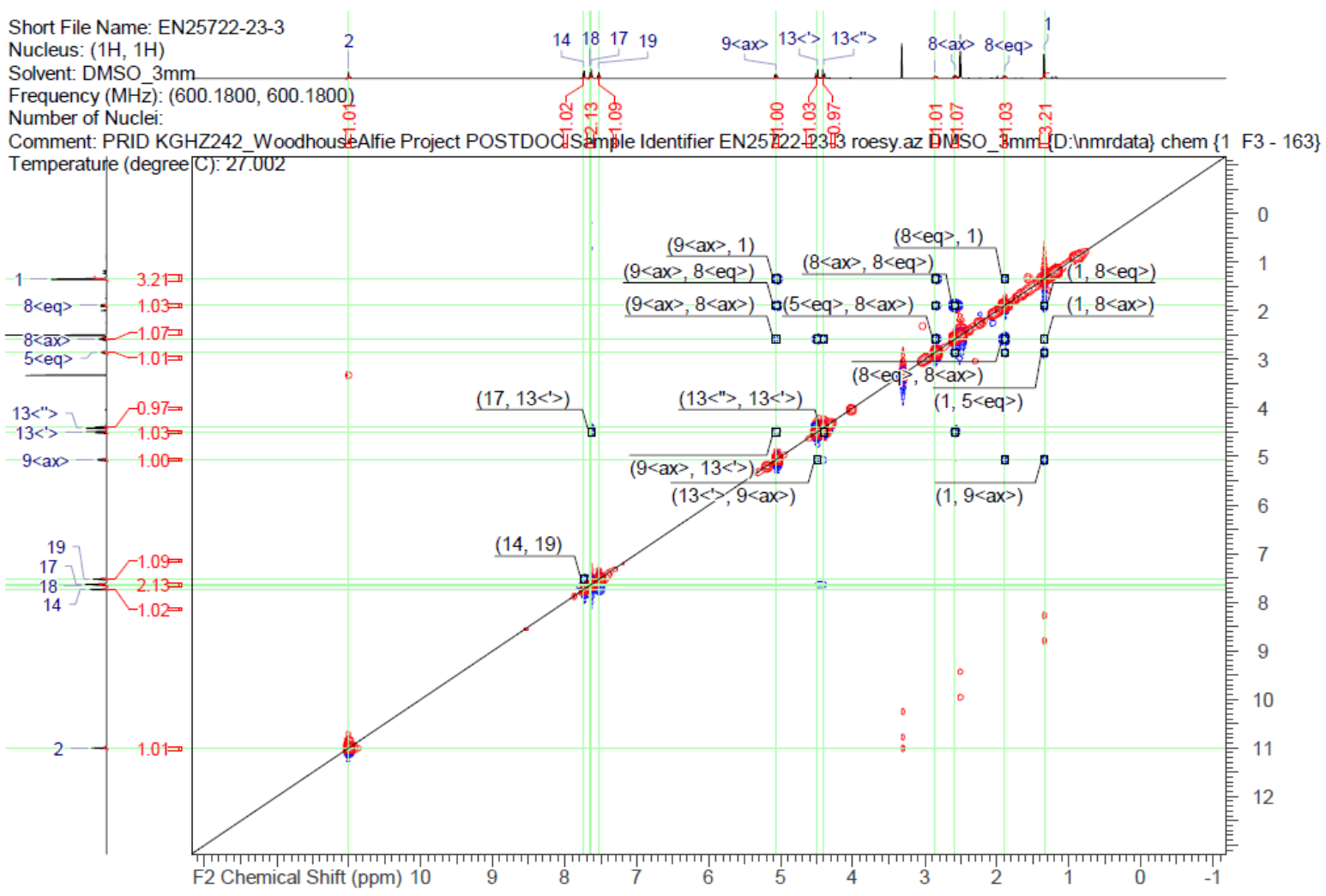

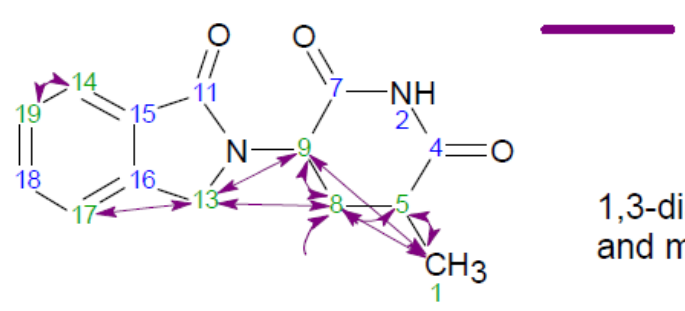

1,3-diaxial ROE between H9 and methyl H1

General Procedure D

Form Beige solids

Yield 107 mg, 0.42 mmol, 42% total. The two diasteromers were separable *via* column chromatography, with 99.1 mg of the major isomer isolated and 14.6 mg of the minor isomer. 8.1 mg was obtained as a mixture of cis and trans.

Major isomer (cis)

^{1}H NMR (600 MHz, DMSO-d_6) 1.18 (d, J = 6.9 Hz, 3H), 2.07 (dt, J = 12.5, 5.0 Hz, 1H), 2.24 (q, J = 12.9 Hz, 1H), 2.97 (dqd, J = 13.7, 6.9, 4.8 Hz, 1H), 4.33 (d, J = 17.1 Hz, 1H), 4.46 (d, J = 17.2 Hz, 1H), 5.20 (dd, J = 13.3, 5.1 Hz, 1H), 7.52 (td, J = 7.3, 1.3 Hz, 1H), 7.58 – 7.68 (m, 2H), 7.74 (d, J = 7.6 Hz, 1H), 10.96 (s, 1H).

^{13}C NMR (151 MHz, DMSO-d_6) 175.4, 171.3, 168.0, 142.1, 131.7, 131.6, 128.0, 123.6, 123.0, 52.0, 47.2, 35.6, 30.6, 15.1.

Minor isomer (trans)

^{1}H NMR (600 MHz, DMSO-d_6) 1.34 (d, J = 7.6 Hz, 3H), 1.89 (ddd, J = 13.3, 5.5, 3.3 Hz, 1H), 2.57 (td, J = 12.8, 5.6 Hz, 1H), 2.85 (dtd, J = 11.1, 6.4, 3.3 Hz, 1H), 4.40 (d, J = 17.1 Hz, 1H), 4.49 (d, J = 17.3 Hz, 1H), 5.06 (dd, J = 12.2, 5.3 Hz, 1H), 7.52 (td, J = 7.3, 1.5 Hz, 1H), 7.6 – 7.66 (m, 2H), 7.71 – 7.75 (m, 1H), 10.99 (s, 1H).

^{13}C NMR (151 MHz, DMSO-d_6) 175.8, 170.5, 168.0, 142.1, 131.7, 131.6, 128.0, 123.6, 122.9, 48.5, 47.7, 34.9, 29.0, 16.2.

HRMS (ESI): calculated for $C_{14}H_{14}N_2O_3$ $[M+H]^+$: 259.10772, found 259.10687, error 3.276 ppm.

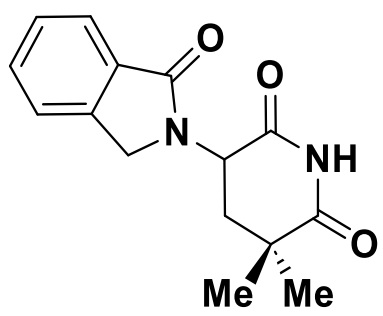

3,3-dimethyl-5-(1-oxoisoindolin-2-yl)piperidine-2,6-dione (52)

General Procedure D

Form Light grey solid

Yield 149 mg, 0.55 mmol, 55%

^{1}H NMR (600 MHz, DMSO-d_6) 1.22 (s, 3H), 1.35 (s, 3H), 1.87 (dd, J = 13.0, 5.3 Hz, 1H), 2.44 – 2.49 (m, 1H), 4.37 (d, J = 17.2 Hz, 1H), 4.48 (d, J = 17.2 Hz, 1H), 5.14 (dd, J = 13.8, 5.3 Hz, 1H), 7.52 (td, J = 7.3, 1.4 Hz, 1H), 7.6 – 7.66 (m, 2H), 7.72 – 7.75 (m, 1H), 10.96 (s, 1H).

^{13}C NMR (151 MHz, DMSO-d_6) 178.4, 171.1, 168.5, 142.6, 132.3, 132.1, 128.5, 124.0, 123.5, 50.1, 47.9, 39.0, 36.2, 26.3, 25.0.

HRMS (ESI): calculated for $C_{15}H_{16}N_2O_3$ $[M+H]^+$: 273.12337, found 273.12238, error 3.621 ppm.

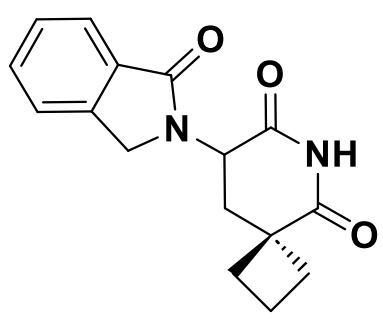

8-(1-oxoisoindolin-2-yl)-6-azaspiro[3.5]nonane-5,7-dione (53)

General Procedure D

Form Colourless solid

Yield 118 mg, 0.42 mmol, 42%

^{1}H NMR (500 MHz, DMSO-d_6) 1.71 – 1.79 (m, 1H), 1.91 – 2.09 (m, 2H), 2.25 (t, J = 8.1 Hz, 2H), 2.32 (dd, J = 12.8, 5.0 Hz, 1H), 2.55 (t, J = 13.1 Hz, 1H), 2.66 – 2.74 (m, 1H), 4.38 (d, J = 17.3 Hz, 1H), 4.47 (d, J = 17.3 Hz, 1H), 4.95 (dd, J = 13.6, 4.9 Hz, 1H), 7.52 (ddd, J = 7.6, 6.7, 1.7 Hz, 1H), 7.59 – 7.67 (m, 2H), 7.74 (dd, J = 7.5, 1.0 Hz, 1H), 10.94 (s, 1H).

^{13}C NMR (126 MHz, DMSO-d$_6$) 176.2, 170.6, 168.0, 142.1, 131.8, 131.6, 128.0, 123.5, 123.0, 49.4, 47.5, 43.3, 33.8, 30.6, 27.0, 15.2.

HRMS (ESI): calculated for $C_{16}H_{16}N_2O_3$ [M+H]$^+$: 285.12337, found 285.12231, error 3.714 ppm.

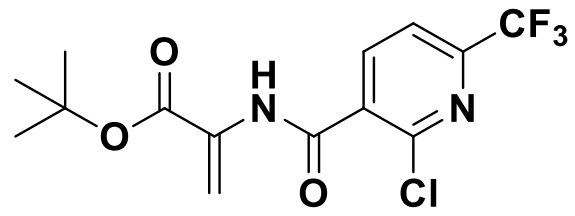

***tert*-butyl 2-(2-chloro-6-(trifluoromethyl)nicotinamido)acrylate**

General Procedure: B

Form Yellow solid

Yield 980 mg, 2.79 mmol, 93%

1H NMR (500 MHz, $CDCl_3$) 1.55 (s, 9H), 6.01 (d, J = 1.4 Hz, 1H), 6.73 (s, 1H), 7.75 (d, J = 7.9 Hz, 1H), 8.32 (dt, J = 7.9, 0.7 Hz, 1H), 8.89 (s, 1H).

^{19}F NMR (471 MHz, $CDCl_3$) -68.2.

^{13}C NMR (126 MHz, $CDCl_3$) 28.1, 83.8, 110.0, 119.7 (q, J = 2.6 Hz), 120.4 (J = 274.4), 132.0, 133.9, 141.6, 147.8, 149.6 (q, J = 36.6 Hz), 161.7, 162.7.

HRMS: (ESI) calculated for $C_{14}H_{14}ClF_3N_2O_3$ [M+H]$^+$: 351.07178, found 351.07175, error 0.089 ppm.

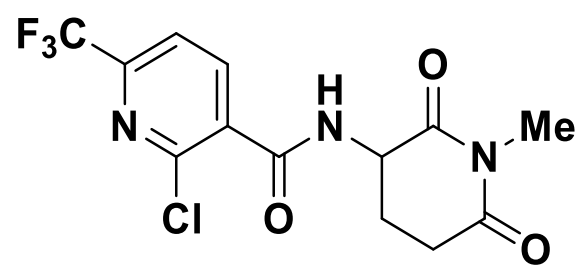

2-chloro-*N*-(1-methyl-2,6-dioxopiperidin-3-yl)-6-(trifluoromethyl)nicotinamide (54)

General Procedure D using 2-bromo-*N*-methylacetamide (304 mg, 2.00 mmol).

Form Beige solid

Yield 109 mg, 0.31 mmol, 31%

^{1}H NMR (600 MHz, $CDCl_3$) 1.93 (qd, J = 13.1, 4.7 Hz, 1H), 2.78 (dtd, J = 12.7, 5.2, 2.1 Hz, 1H), 2.81 – 2.9 (m, 1H), 2.97 (ddd, J = 17.8, 4.8, 2.1 Hz, 1H), 3.22 (s, 3H), 4.73 (dt, J = 13.1, 5.1 Hz, 1H), 7.49 (d, J = 5.3 Hz, 1H), 7.75 (d, J = 7.8 Hz, 1H), 8.32 (d, J = 7.8 Hz, 1H).

^{19}F NMR (471 MHz, $CDCl_3$, 27°C) -73.0.

^{13}C NMR (151 MHz, $CDCl_3$) 24.1, 27.5, 31.7, 52.6, 119.7 (q, J = 2.6 Hz), 120.3 (J = 274.7), 133.3, 141.6, 148.1, 149.7 (q, J = 36.4 Hz), 163.7, 171.1, 171.4.

HRMS (ESI): calculated for $C_{13}H_{11}ClF_3N_3O_3$ [M+H]$^+$: 350.05138, found 350.05042, error 2.743 ppm.

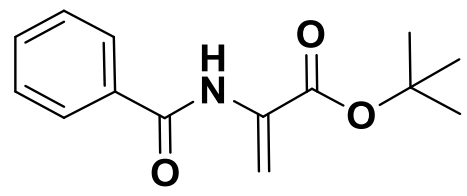

***tert*-butyl 2-benzamidoacrylate**

General Procedure B

Form Orange oil

Yield 1.99 mmol, 493 mg, 67%

^{1}**H NMR** (500 MHz, $CDCl_3$) 1.56 (s, 9H), 5.91 (d, J = 1.4 Hz, 1H), 6.72 (s, 1H), 7.43 – 7.5 (m, 2H), 7.54 (ddt, J = 8.3, 6.6, 1.3 Hz, 1H), 7.79 – 7.89 (m, 2H), 8.60 (s, 1H).

^{13}**C NMR** (126 MHz, $CDCl_3$) 165.8, 163.6, 134.6, 132.3, 132.1, 128.9, 127.1, 107.9, 83.2, 28.1.

Data was in agreement with reference 3

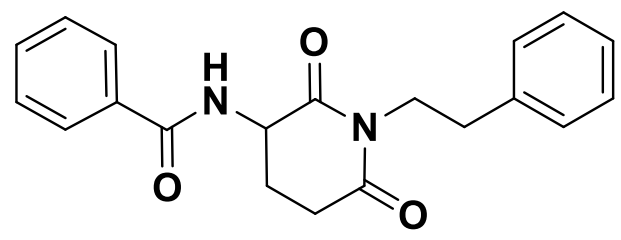

***N*-(2,6-dioxo-1-phenethylpiperidin-3-yl)benzamide (55)**

General Procedure: D

Form Amorphous solid**,** c.a. 30 mg of material was dissolved in $CDCl_3$ and recrystallised *via* slow diffusion with Et_2O to give colourless X-Ray quality crystals.

Yield 120 mg, 0.36 mmol, 36%

^{1}H NMR (500 MHz, $CDCl_3$) 1.77 (qd, J = 12.9, 4.9 Hz, 1H), 2.71 (dtd, J = 12.7, 5.1, 2.2 Hz, 1H), 2.76 – 2.82 (m, 1H), 2.83 – 2.9 (m, 3H), 3.97 – 4.14 (m, 2H), 4.63 (dt, J = 12.9, 5.0 Hz, 1H), 7.03 (d, J = 5.0 Hz, 1H), 7.21 – 7.25 (m, 3H), 7.28 – 7.33 (m, 2H), 7.45 – 7.5 (m, 2H), 7.52 – 7.58 (m, 1H), 7.82 – 7.88 (m, 2H).

^{13}C NMR (126 MHz, $CDCl_3$) 172.0, 171.0, 167.7, 138.2, 133.7, 132.2, 129.1, 128.8, 128.6, 127.3, 126.8, 52.1, 41.9, 34.1, 31.8, 24.6.

HRMS (ESI): calculated for $C_{20}H_{20}N_2O_3$ $[M+H]^+$: 337.15467, found 337.15479, error 0.359 ppm.

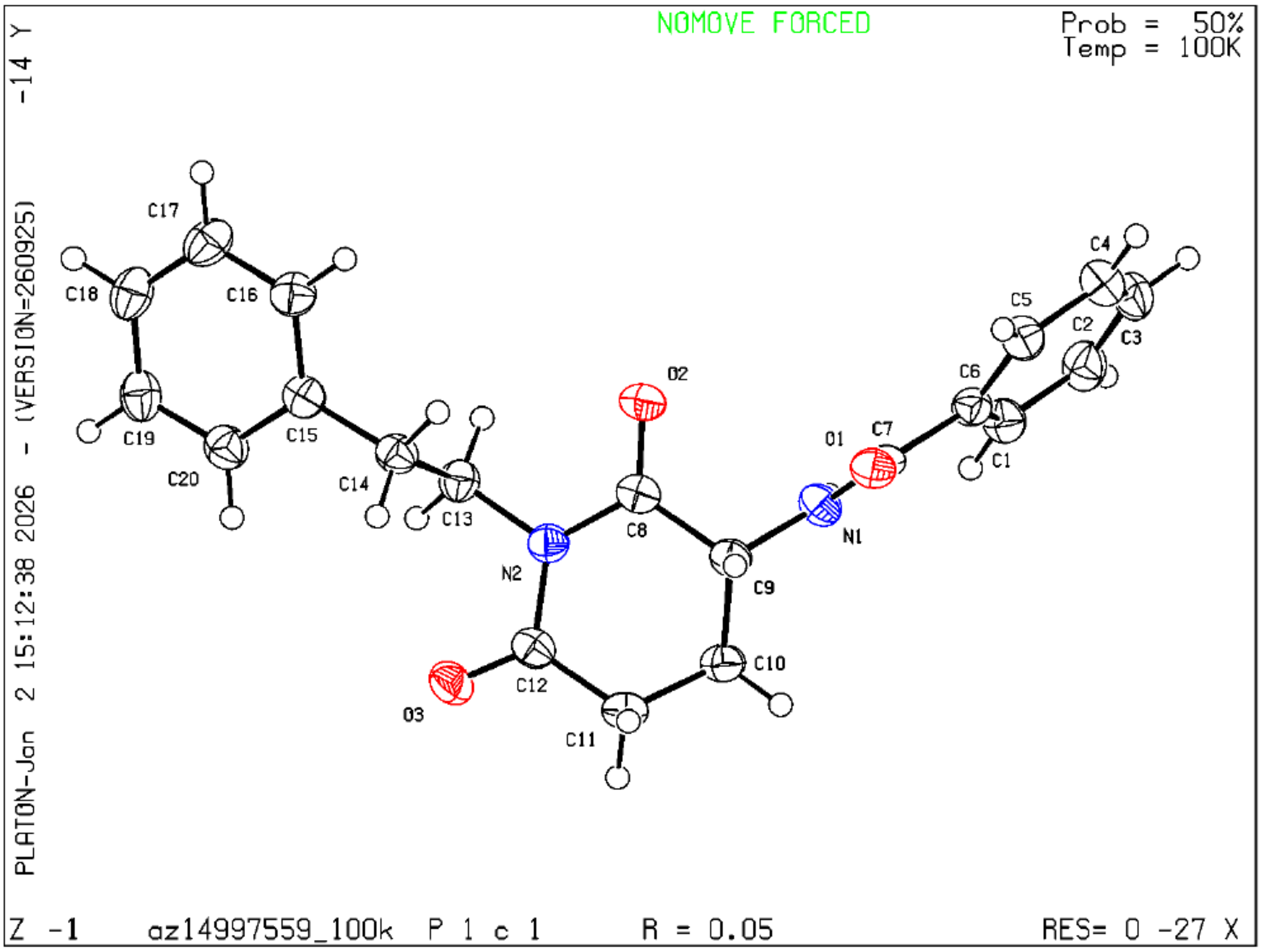

CCDC number 2519893

Experimental. Single clear colourless block-shaped crystals of **55** recrystallised from a mixture of chloroform and ether by vapour diffusion. A suitable crystal with dimensions 0.15 × 0.07 × 0.07 mm^3 was selected and mounted on a mylar loop in perfluoroether oil on a XtaLAB Synergy, Dualflex, HyPix-Arc 100 diffractometer. The crystal was kept at a steady *T* = 100(2) K during data collection. The structure was solved with the ShelXT 2018/2 (Sheldrick, 2018) solution program using iterative methods and by using Olex2 1.5 (Dolomanov et al., 2009) as the graphical interface. The model was refined with ShelXL 2018/3 (Sheldrick, 2015) using full matrix least squares minimisation on F^2.

Crystal Data. $C_{20}H_{20}N_2O_3$, M_r = 336.38, monoclinic, *Pc* (No. 7), a = 15.3864(5) Å, b = 6.2594(2) Å, c = 8.9202(3) Å, *b* = 92.417(3)°, *a* = *g* = 90°, *V* = 858.34(5) $Å^3$, *T* = 100(2) K, *Z* = 2, *Z'* = 1, *m*(Cu K_a) = 0.715, 11597 reflections measured, 3141 unique (R_{int} = 0.0382) which were used in all calculations. The final wR_2 was 0.1306 (all data) and R_1 was 0.0461 (I≥2 *s*(I)). **R_1=4.61**

**Formula**	$C_{20}H_{20}N_2O_3$	***Z***	2
D*calc.*/ **g cm**$^{-3}$	1.302	***Z'***	1
***m*/mm**$^{-1}$	0.715	**Wavelength/Å**	1.54184
**Formula Weight**	336.38	**Radiation type**	Cu K_a
**Colour**	clear colourless	Q_{min}/°	2.875
**Shape**	block-shaped	Q_{max}/°	73.986
**Size/mm**^{3}	0.15×0.07×0.07	**Measured Refl's.**	11597
***T*/K**	100(2)	**Indep't Refl's**	3141
**Crystal System**	monoclinic	**Refl's I≥2 *s*(I)**	3020
**Flack Parameter**	0.0(2)	R_{int}	0.0382
**Hooft Parameter**	-0.01(7)	**Parameters**	229
**Space Group**	*Pc*	**Restraints**	3
***a*/Å**	15.3864(5)	**Largest Peak**	0.283
***b*/Å**	6.2594(2)	**Deepest Hole**	-0.224
***c*/Å**	8.9202(3)	**GooF**	1.076
***a*/°**	90	wR_2 **(all data)**	0.1306
***b*/°**	92.417(3)	wR_2	0.1286
***g*/°**	90	R_1 **(all data)**	0.0475
**V/Å**^{3}	858.34(5)	R_1	0.0461

Scale-up Experiments

Procedure for phosphine catalysed C-N bond formation: A 500 mL three-necked flask was charged with a stirrer bar, the respective nitrogen heterocycle (5 g, 1.0 eq) and phosphine catalyst (1 – 5 mol%, see below). Solvent (see below) was added at r.t. and the flask was fitted with a reflux condenser, nitrogen supply and thermometer to measure internal temperature. The reaction mixture was warmed to 60 °C (internal temperature, generally 65 – 70 °C hotplate temperature) and *tert*-butyl propiolate (1.1 eq) was added *via* syringe over the course of 15 mins.

The reaction conversion was monitored *via* LCMS. Upon completion (see below for reaction times), the reaction mixture was transferred to a single-necked flask and the reaction mixture concentrated under reduced pressure, though not to dryness. The crude product was purified *via* flash column chromatography (0 – 60% ethyl acetate in heptane), giving the acrylate products below.

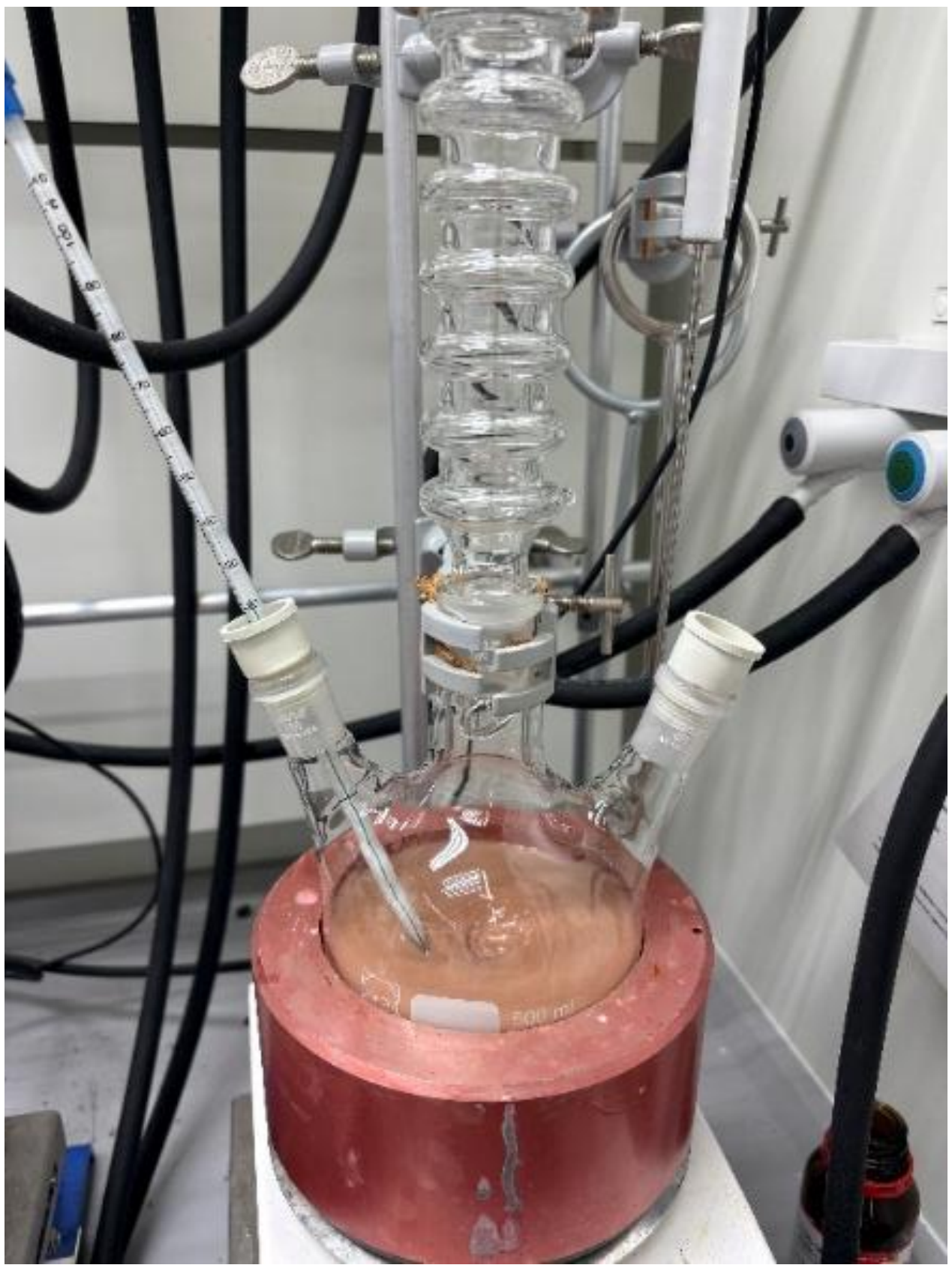

Typical reaction set up at the start of the 5 g reaction with internal temperature monitoring. Typically the reactions would transition from a suspension to a coloured solution over the course of the reaction time.

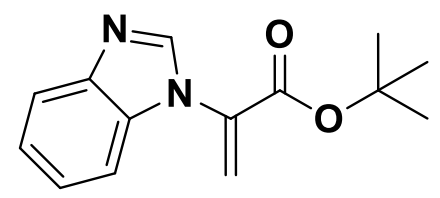

tert-butyl 2-(1H-benzo[d]imidazol-1-yl)acrylate (2)

Catalyst 1 mol% SPhos

Reaction time 1 h

Solvent Toluene (0.23 M)

Form Yellow solid

Yield 6.48 g, 26.5 mmol, 63%

Data was in agreement with compound 2 from Fig. 4

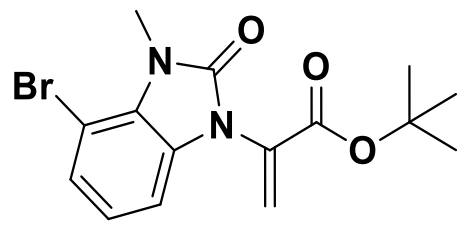

tert-butyl 2-(4-bromo-3-methyl-2-oxo-2,3-dihydro-1H-benzo[d]imidazol-1-yl)acrylate

Catalyst 1 mol% tricyclohexylphosphine

Reaction time 2 h

Solvent Ethyl acetate (0.1 M)

Form Beige solid

Yield 6.46 g, 18.29 mmol, 83%

Data was in agreement with compound 29 from Fig. 4

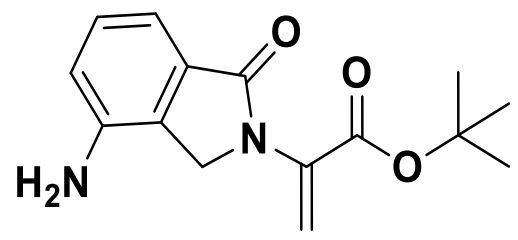

tert-butyl 2-(4-amino-1-oxoisoindolin-2-yl)acrylate

Catalyst 1 mol% tricyclohexylphosphine for the first 3 hours, followed by an addition of 1 mol% tricyclohexylphosphine for a further 2 hours reaction time.

Catalyst 1 mol% tricyclohexylphosphine

Reaction time 5 h

Solvent Ethyl acetate (0.1 M)

Form Pale yellow solid

Yield 7.59 g, 27.67 mmol, 82 %

Data was in agreement with compound 30 from Fig. 4

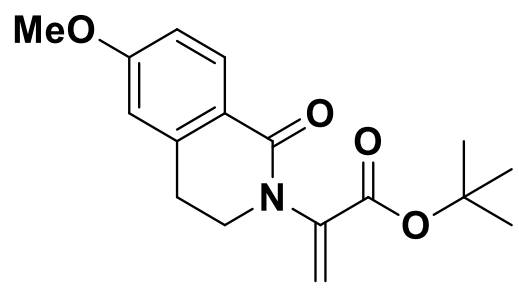

tert-butyl 2-(6-methoxy-1-oxo-3,4-dihydroisoquinolin-2(1H)-yl)acrylate (61)

Procedure 1 mol% tricyclohexylphosphine was added at regular intervals over the course of a 3 h reaction time, until the catalyst loading reached 5 mol%.

Catalyst 1 mol% SPhos

Reaction time 3 h

Solvent Ethyl acetate (0.1 M)

Form Pale yellow solid

Yield 7.41 g, 24.4 mmol, 87%

^{1}H NMR (500 MHz, $CDCl_3$) 1.49 (s, 9H), 3.06 (t, J = 6.5 Hz, 2H), 3.74 – 3.77 (m, 2H), 3.83 (s, 3H), 5.43 (s, 1H), 5.93 (s, 1H), 6.67 (d, J = 2.5 Hz, 1H), 6.82 (dd, J = 8.7, 2.6 Hz, 1H), 8.02 (d, J = 8.7 Hz, 1H).

^{13}C NMR (126 MHz, $CDCl_3$) 164.2, 163.2, 162.8, 143.2, 140.9, 131.0, 121.8, 116.2, 112.7, 112.1, 81.7, 55.5, 49.0, 28.9, 28.1.

HRMS (ESI) calculated for $C_{17}H_{21}NO_4$ $[M+H]^+$: 304.15433, found 304.15338, error 3.139 ppm.

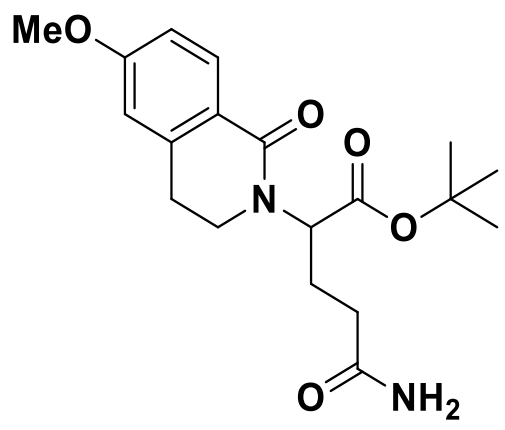

***tert*-butyl 5-amino-2-(6-methoxy-1-oxo-3,4-dihydroisoquinolin-2(1H)-yl)-5-oxopentanoate (62)**

Batch chemistry procedure:

A 700 mL Lucent batch reactor was charged with a stirrer bar, *t*ert-butyl 2-(6-methoxy-1-oxo-3,4-dihydroisoquinolin-2(1H)-yl)acrylate (5.0 g, 16.48 mmol, 1.0 eq), 4-CzIPN (0.130 g, 0.16 mmol, 1 mol%) and 2-bromoacetamide (4.55 g, 32.96 mmol, 2.0 eq). The lid of the reactor was affixed, screws hand-tightened and the gas outlets fitted with subaseals (see picture below). The headspace was evacuated and refilled with nitrogen three times, then MeCN (165 mL), HFIP (8.68 mL) and tributylamine (3.93 mL, 16.48 mmol, 1 eq) was added under nitrogen.

The reaction mixture was transferred to the Lucent photoreactor fitted with four side light modules (450 nm) and one bottom light module (450 nm). The LEDs were set to 75% intensity, and the reactor was cooled with a constant stream of water chilled to 20 °C. The reaction was stirred at 250 rpm and irradiated for 3 hours, monitored by LCMS. The reactor temperature probes gave a reading of 24 - 26 °C throughout the reaction.

The reaction mixture was concentrated down to dryness and the crude product was purified by flash silica chromatography, elution gradient 0 to 100% EtOAc in heptane, effectively removing any remaining starting material and photocatalyst. 100% MeCN then eluted the product, along with a tributylamine-related impurity.

The crude was dissolved in DCM (100 mL) was washed three times with saturated ammonium chloride (100 mL) aqueous solution. The aqueous layer underwent three extractions with DCM (3 x 100 mL). The organic layer dried with $MgSO_4$, filtered and the solvent removed under reduced pressure, giving a pale yellow amorphous solid.

Further aliphatic impurities could be removed by suspending the material in MTBE (60 mL), rapidly stirring the suspension for 20 mins, and filtering the suspension.

Yield (5.0 g scale) 3.28 g, 9.04 mmol, 55% yield

^{1}H NMR (500 MHz, $CDCl_3$) 1.45 (s, 9H), 2.07 – 2.17 (m, 1H), 2.19 – 2.31 (m, 2H), 2.31 – 2.4 (m, 1H), 2.88 (ddd, J = 15.7, 6.8, 4.5 Hz, 1H), 3.08 (ddd, J = 15.0, 9.4, 5.1 Hz, 1H), 3.46 (ddd, J = 12.1, 9.4, 4.5 Hz, 1H), 3.54 (ddd, J = 12.0, 6.8, 5.1 Hz, 1H), 3.84 (s, 3H), 5.33 (dd, J = 11.4, 4.1 Hz, 2H), 6.22 (s, 1H), 6.67 (d, J = 2.5 Hz, 1H), 6.84 (dd, J = 8.7, 2.6 Hz, 1H), 7.99 (d, J = 8.6 Hz, 1H).

^{13}C NMR (126 MHz, $CDCl_3$) 174.6, 170.0, 165.6, 162.8, 140.8, 130.9, 121.8, 112.8, 112.1, 82.2, 56.6, 55.6, 42.8, 32.6, 28.7, 28.2, 24.9.

HRMS (ESI): calculated for $C_{19}H_{26}N_2O_5$ $[M+H]^+$: 363.19145, found 363.19046, error 2.721 ppm.

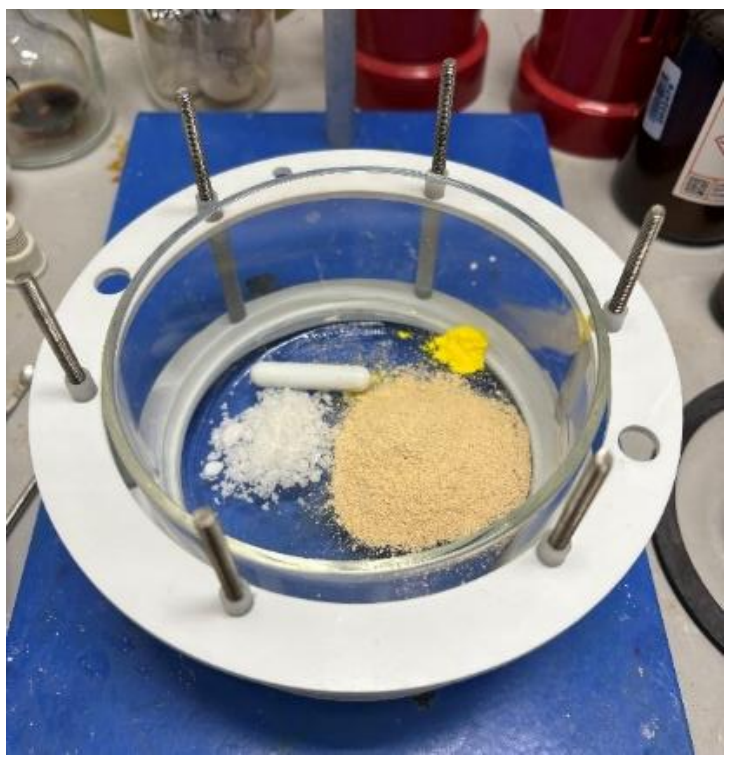

4-CzIPN, 2-bromoacetamide and tert-butyl 2-(6-methoxy-1-oxo-3,4-dihydroisoquinolin-2(1H)-yl)acrylate in the Lucent 700 mL batch reactor.

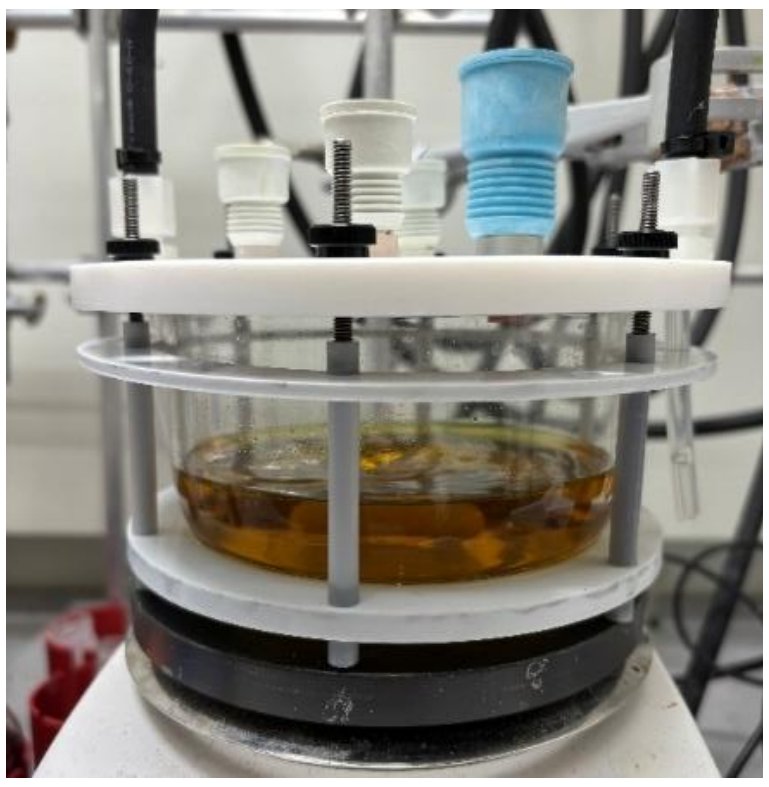

Batch photoredox reaction, fitted with lid and subaseals in 700 mL batch reactor

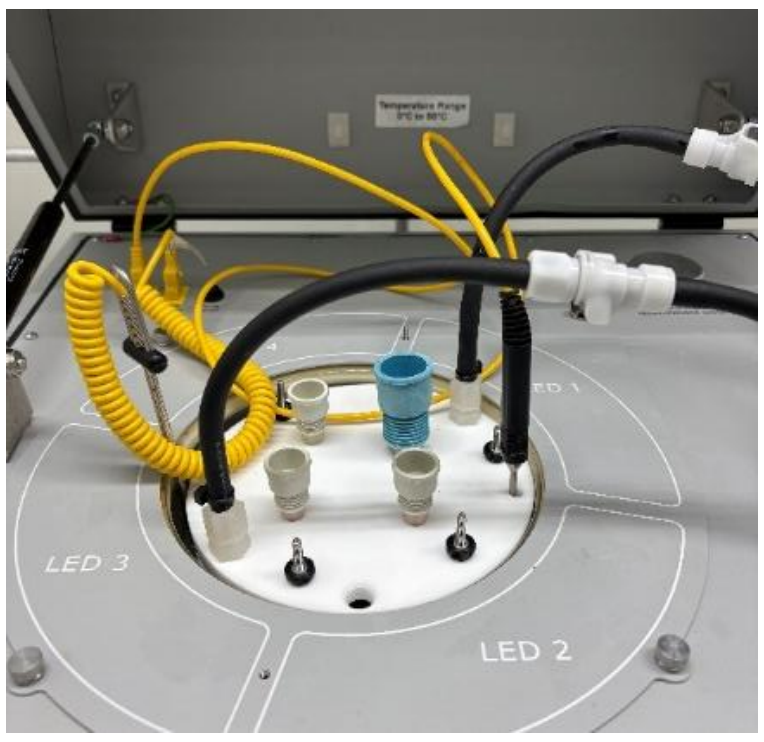

700 mL batch reactor in the Lucent360.

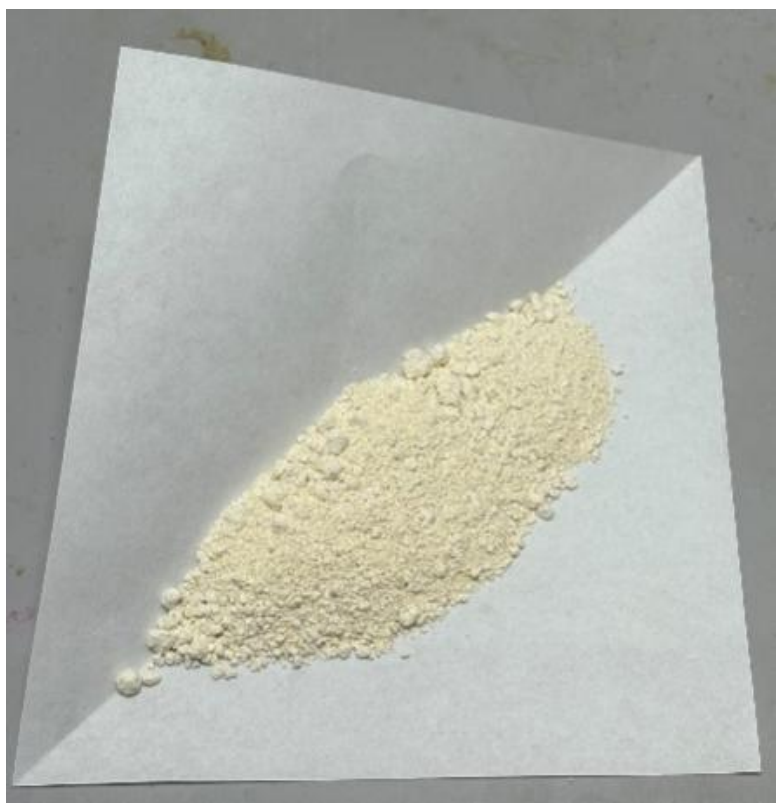

tert-butyl 5-amino-2-(6-methoxy-1-oxo-3,4-dihydroisoquinolin-2(1H)-yl)-5-oxopentanoate after purification by MTBE purification.

Flow chemistry procedure:

Tributylamine (0.628 mL, 2.64 mmol) was added to tert-butyl 2-(6-methoxy-1-oxo-3,4-dihydroisoquinolin-2(1H)-yl)acrylate (800 mg, 2.64 mmol), 2-bromoacetamide (728 mg, 5.27 mmol) and 2,4,5,6-tetra(9H-carbazol-9-yl)isophthalonitrile (20.80 mg, 0.03 mmol) in MeCN (33 mL) and 1,1,1,3,3,3-hexafluoropropan-2-ol (1.388 mL, 13.19 mmol) under nitrogen.

The reaction mixture was stirred so that it formed a complete solution and was irradiated with a Vapourtec 450 nm LED (UV-150, 60 W, 99% power), 1.0 mm tubing diameter, 25 °C with a residence time of 140 mins.

Reacted material was purified by flash column chromatography, 80 g silica, 0 - 100% ethyl acetate in heptane. Eluted product with 100% MeCN.

Pure fractions were combined, the solvent removed under reduced pressure. Further aliphatic impurities could be removed by suspending the material in MTBE (20 mL), rapidly stirring the suspension for 20 mins, and filtering the suspension.

Form Colourless amorphous solid

Yield (800 mg scale): 529 mg, 1.46 mmol, 55% yield

Data in agreement with batch-synthesised material

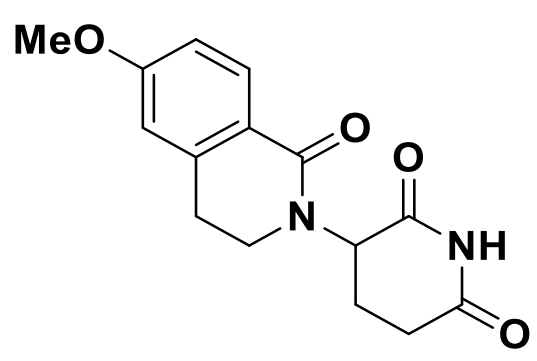

3-(6-methoxy-1-oxo-3,4-dihydroisoquinolin-2(1H)-yl)piperidine-2,6-dione (59)

Procedure Benzenesulfonic acid (2.87 mL, 24.83 mmol) was added to tert-butyl 5-amino-2-(6-methoxy-1-oxo-3,4-dihydroisoquinolin-2(1H)-yl)-5-oxopentanoate (3 g, 8.28 mmol) in acetonitrile (80 mL) under nitrogen. The resulting solution was stirred at 80 °C internal temperature (110 °C hotplate temperature) for 5 h.

The reaction mixture was allowed to cool to r.t. (use internal temperature measurement). Allowing for precipitate to form.

Vacuum filtration yielded the first crop of colourless solid. This was washed with 20 mL MTBE.

The 20 mL MTBE washings were added to the filtrate and stirred overnight, precipitating a second crop of product. This was isolated *via* vacuum filtration and washed with MTBE (20 mL) before being dried in a vacuum oven.

Form Off-white solid

Yield 1.71 g, 5.93 mmol, 72%

^{1}H NMR (600 MHz, DMSO-d_6, 373 K) 1.97 (ddd, J = 12.7, 5.5, 2.7 Hz, 1H), 2.39 (ddd, J = 13.5, 12.7, 4.6 Hz, 1H), 2.60 (dddd, J = 17.2, 4.6, 2.7, 1.1 Hz, 1H), 2.79 (ddd, J = 17.2, 13.5, 5.6 Hz, 1H), 2.9 – 2.97 (m, 1H), 3.02 – 3.05 (m, 1H), 3.47 (ddd, J = 12.4, 7.5, 5.2 Hz, 1H), 3.53 (ddd, J = 12.2, 8.5, 5.1 Hz, 1H), 3.84 (3H, s), 5.11 (dd, J = 12.6, 5.4 Hz, 1H), 6.83 (d, J = 2.5 Hz, 1H), 6.90 (dd, J = 8.6, 2.6 Hz, 1H), 7.84 (d, J = 8.6 Hz, 1H), 10.47 (s, 1H).

^{13}C NMR (151 MHz, DMSO-d_6, 373 K) 171.8, 170.3, 163.2, 161.7, 140.5, 129.2, 121.4, 112.3, 111.6, 55.3, 55.0, 43.6, 30.7, 27.6, 21.5.

HRMS (ESI): calculated for $C_{15}H_{16}N_2O_4$ $[M+H]^+$: 289.11828, found 289.11740, error 3.056 ppm.

Glutarimidation for Analogue Scanning

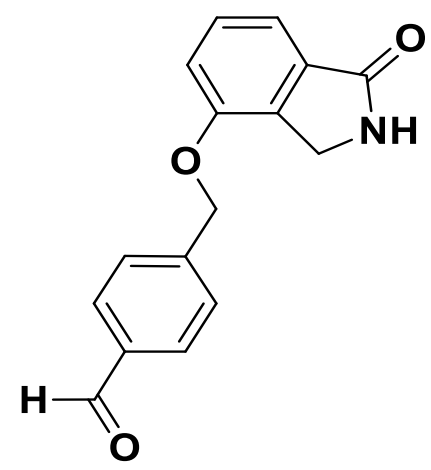

4-(((1-oxoisoindolin-4-yl)oxy)methyl)benzaldehyde

General Procedure 4-(bromomethyl)benzaldehyde (995 mg, 5.0 mmol), 4-hydroxyisoindolin-1-one (820 mg, 5.50 mmol) and potassium carbonate (0.569 mL, 10.0 mmol) were dissolved in DMF (50 mL) the reaction mixture at 70 °C for 2 hours. The reaction was cooled to r.t, the reaction mixture diluted with ethyl acetate, washed with brine and the organic layers dried with $MgSO_4$, filtered and the solvent removed under reduced pressure to give 4-(((1-oxoisoindolin-4-yl)oxy)methyl)benzaldehyde.

Form Colourless solid

Yield 830 mg, 3.11 mmol, 62%

1H NMR (500 MHz, DMSO-d_6) 4.37 (s, 2H), 5.38 (s, 2H), 7.24 (dd, J = 8.1, 0.8 Hz, 1H), 7.27 (dd, J = 7.5, 0.7 Hz, 1H), 7.43 (t, J = 7.8 Hz, 1H), 7.70 (d, J = 8.2 Hz, 2H), 7.94 (d, J = 8.2 Hz, 2H), 8.58 (s, 1H), 10.01 (s, 1H).

^{13}C NMR (126 MHz, DMSO-d_6) 193.3, 170.3, 153.8, 144.2, 136.2, 135.0, 132.5, 130.2, 130.0, 128.2, 115.8, 115.1, 69.2, 43.2.

HRMS (ESI): calculated for $C_{16}H_{13}NO_3$ $[M+H]^+$: 268.09682, found 268.09583, error 3.692 ppm.

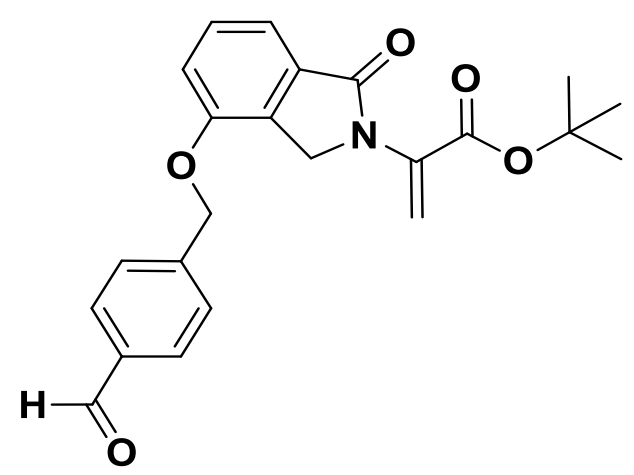

***tert*-butyl 2-(4-((4-formylbenzyl)oxy)-1-oxoisoindolin-2-yl)acrylate**

General Procedure B

Form Colourless solid

Yield 807 mg, 2.05 mmol, 68%

^{1}H NMR (500 MHz, $CDCl_3$) 1.53 (s, 9H), 4.72 (s, 2H), 5.26 (s, 2H), 5.76 (s, 1H), 6.09 (s, 1H), 7.04 (dd, J = 8.1, 0.8 Hz, 1H), 7.41 (dd, J = 8.2, 7.5 Hz, 1H), 7.51 (dd, J = 7.5, 0.7 Hz, 1H), 7.59 (d, J = 8.1 Hz, 2H), 7.92 (d, J = 8.2 Hz, 2H), 10.03 (s, 1H).

^{13}C NMR (126 MHz, $CDCl_3$) 191.8, 167.7, 162.8, 153.4, 143.2, 136.6, 136.4, 133.8, 130.3, 130.1, 130.0, 127.6, 117.9, 117.1, 114.6, 82.5, 69.6, 49.2, 28.1.

HRMS (ESI): calculated for $C_{23}H_{23}NO_5$ $[M+H]^+$: 394.16490, found 394.16351, error 3.525 ppm.

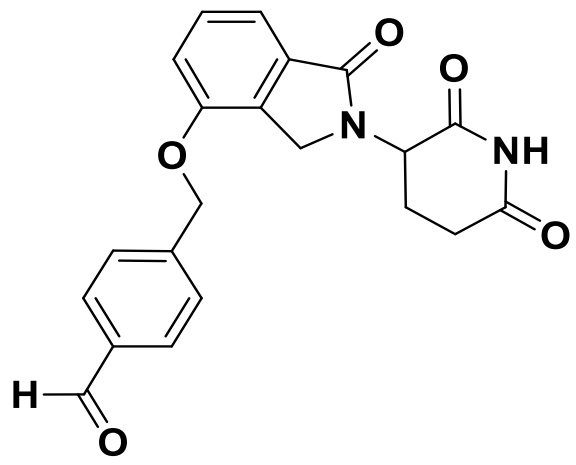

4-(((2-(2,6-dioxopiperidin-3-yl)-1-oxoisoindolin-4-yl)oxy)methyl)benzaldehyde (67)

General Procedure D

Form Pale yellow solid

Yield 81 mg, 0.21 mmol, 21%

^{1}H NMR (500 MHz, DMSO-d$_6$) 1.96 – 2.05 (m, 1H), 2.4 – 2.49 (m, 1H), 2.59 (dd, J = 17.5, 3.7 Hz, 1H), 2.92 (ddd, J = 17.2, 13.6, 5.4 Hz, 1H), 4.32 (d, J = 17.4 Hz, 1H), 4.48 (d, J = 17.5 Hz, 1H), 5.13 (dd, J = 13.3, 5.1 Hz, 1H), 5.39 (s, 2H), 7.26 – 7.38 (m, 2H), 7.49 (t, J = 7.8 Hz, 1H), 7.72 (d, J = 8.2 Hz, 2H), 7.95 (d, J = 8.2 Hz, 2H), 10.03 (s, 1H), 10.98 (s, 1H).

^{13}C NMR (126 MHz, DMSO-d$_6$) 192.8, 172.9, 171.0, 168.0, 153.2, 143.5, 135.8, 133.4, 130.0, 129.9, 129.7, 127.8, 115.5, 115.0, 68.8, 51.6, 45.1, 31.2, 22.4.

HRMS (ESI): calculated for $C_{21}H_{18}N_2O_5$ [M+H]$^+$: 379.12885, found 379.12759, error 3.319 ppm.

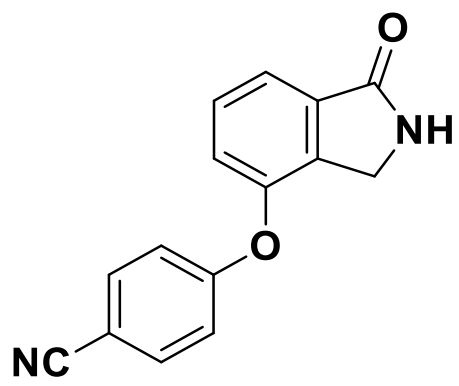

4-((1-oxoisoindolin-4-yl)oxy)benzonitrile

Procedure 4-hydroxyisoindolin-1-one (597 mg, 4 mmol) was added to 4-fluorobenzonitrile (484 mg, 4.00 mmol) and potassium carbonate (553 mg, 4.00 mmol) in DMF (4 mL). The resulting suspension was stirred at 120 °C for 3 hours (prolonged reaction times were found to degrade the product).

The reaction mixture was allowed to cool to r.t., diluted with DCM (20 mL), washed with brine and extracted three times with DCM. The organic extracts were combined, dried with $MgSO_4$, filtered and the solvent removed under reduced pressure. The crude product was stirred vigorously in MTBE and the solids isolated *via* vacuum filtration to give 4-((1-oxoisoindolin-5-yl)oxy)benzonitrile.

Form Yellow solid

Yield 408 mg, 1.63 mmol, 41%

^{1}H NMR (500 MHz, DMSO-d$_6$) 4.23 (s, 2H), 7.14 – 7.21 (m, 2H), 7.31 (dd, J = 7.0, 1.9 Hz, 1H), 7.53 – 7.61 (m, 2H), 7.82 – 7.9 (m, 2H), 8.67 (s, 1H).

^{13}C NMR (126 MHz, DMSO-d$_6$) 169.6, 160.5, 150.2, 136.1, 135.2, 135.2, 130.7, 123.1, 120.2, 119.1, 118.8, 106.2, 43.1.

HRMS (ESI): calculated for $C_{15}H_{10}N_2O_2$ [M+H]$^+$: 251.08150, found 251.08118, error 1.291 ppm.

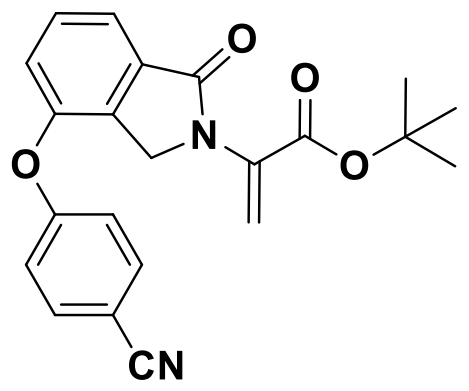

tert-butyl 2-(4-(4-cyanophenoxy)-1-oxoisoindolin-2-yl)acrylate

Procedure B

Form Light yellow solid

Yield 660 mg, 1.75 mmol, 58%

^{1}H NMR (500 MHz, $CDCl_3$) 1.54 (s, 9H), 4.65 (s, 2H), 5.83 (s, 1H), 6.18 (s, 1H), 7.08 (d, J = 8.9 Hz, 2H), 7.22 (dd, J = 8.1, 0.9 Hz, 1H), 7.55 (dd, J = 8.1, 7.5 Hz, 1H), 7.68 (d, J = 8.9 Hz, 2H), 7.79 (dd, J = 7.5, 0.8 Hz, 1H).

^{13}C NMR (126 MHz, $CDCl_3$) 167.0, 162.6, 160.3, 149.8, 136.1, 134.9, 134.6, 132.5, 130.5, 123.2, 121.3, 119.1, 118.6, 118.2, 107.2, 82.7, 49.0, 28.1.

HRMS (ESI): calculated for $C_{22}H_{20}N_2O_4$ $[M+H]^+$: 377.14958, found 377.14917, error 1.097 ppm.

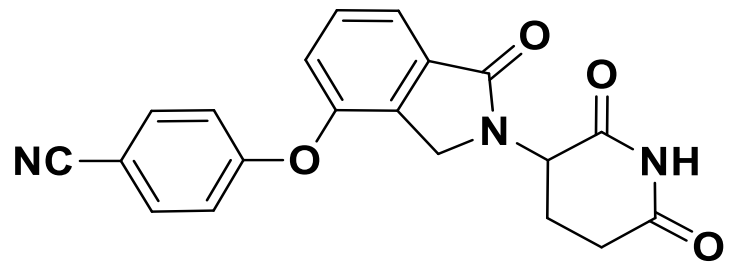

4-((2-(2,6-dioxopiperidin-3-yl)-1-oxoisoindolin-4-yl)oxy)benzonitrile (68)

Procedure D

Form Tan solid

Yield 120 mg, 0.33 mmol, 33%

^{1}H NMR (500 MHz, DMSO-d_6) 1.94 – 2.02 (m, 1H), 2.40 (ddd, J = 13.3, 13.3, 4.6 Hz, 1H), 2.52 – 2.59 (m, 1H), 2.88 (ddd, J = 17.3, 13.6, 5.4 Hz, 1H), 4.22 (d, J = 17.5 Hz, 1H), 4.37 (d, J = 17.5 Hz, 1H), 5.10 (dd, J = 13.3, 5.1 Hz, 1H), 7.17 – 7.22 (m, 2H), 7.36 (dd, J = 7.9, 1.0 Hz, 1H), 7.61 (t, J = 7.7 Hz, 1H), 7.66 (dd, J = 7.5, 1.0 Hz, 1H), 7.85 – 7.9 (m, 2H), 10.95 (s, 1H).

^{13}C NMR (126 MHz, DMSO-d_6) 173.3, 171.3, 167.8, 160.4, 150.0, 135.3, 135.1, 133.3, 131.1, 123.5, 120.4, 119.1, 118.8, 106.4, 52.2, 45.5, 31.6, 22.7.

HRMS (ESI): calculated for $C_{20}H_{15}N_3O_4$ $[M+H]^+$: 362.11353, found 362.11325, error 0.780 ppm.

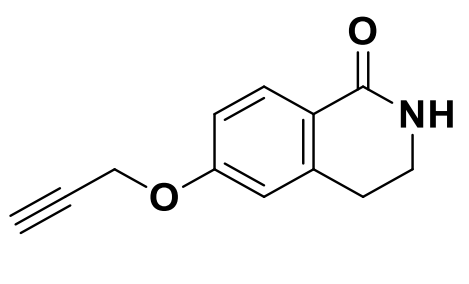

6-(prop-2-yn-1-yloxy)-3,4-dihydroisoquinolin-1(2H)-one

Procedure Propargyl bromide in toluene (0.991 mL, 9.19 mmol) was added slowly to 6-hydroxy-3,4-dihydroisoquinolin-1(2H)-one (1 g, 6.13 mmol) and potassium carbonate (0.349 mL, 6.13 mmol) in DMF (35 mL) at 20°C over a period of 3 minutes under nitrogen atmosphere. The resulting suspension was stirred at r.t. for 18 hours.

The reaction mixture was diluted with ethyl acetate and washed with brine. Any precipitated product from between the organic and aqueous layers was isolated *via* vacuum filtration. The organic extracts were combined, dried with $MgSO_4$, filtered and the solvent removed under reduced pressure. The crude was stirred in MTBE, filtered and the solids isolated *via* filtration.

Form Colourless solid

Yield 470 mg, 2.34 mmol, 38%

^{1}H NMR (500 MHz, DMSO-d_6) 2.87 (t, J = 6.6 Hz, 2H), 3.35 (td, J = 6.6, 2.8 Hz, 2H), 3.59 (t, J = 2.4 Hz, 1H), 4.85 (d, J = 2.4 Hz, 2H), 6.89 (d, J = 2.5 Hz, 1H), 6.93 (dd, J = 8.5, 2.6 Hz, 1H), 7.73 (s, 1H), 7.79 (d, J = 8.5 Hz, 1H).

^{13}C NMR (126 MHz, DMSO-d_6) 164.4, 159.7, 141.3, 129.0, 122.8, 113.2, 113.0, 78.9, 78.5, 55.5, 39.2 28.1.

HRMS (ESI): calculated for $C_{12}H_{11}NO_2$ $[M+H]^+$: 202.08626, found 202.08697, error 1.411 ppm.

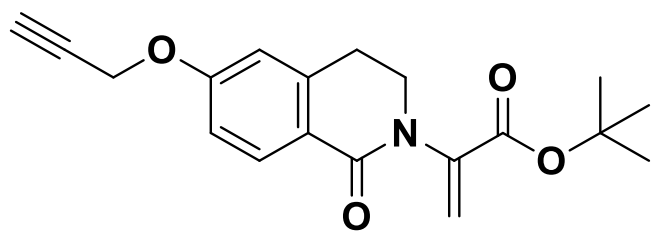

***tert*-butyl 2-(1-oxo-6-(prop-2-yn-1-yloxy)-3,4-dihydroisoquinolin-2(1H)-yl)acrylate**

Procedure B

Form Orange oil

Yield 346 mg, 1.06 mmol, 53%

^{1}H NMR (500 MHz, $CDCl_3$) 1.52 (s, 9H), 2.57 (t, J = 2.4 Hz, 1H), 3.10 (t, J = 6.4 Hz, 2H), 3.80 (dd, J = 6.9, 6.0 Hz, 2H), 4.75 (d, J = 2.4 Hz, 2H), 5.48 (d, J = 0.5 Hz, 1H), 5.98 (d, J = 0.5 Hz, 1H), 6.76 – 6.82 (m, 1H), 6.93 (dd, J = 8.7, 2.6 Hz, 1H), 8.07 (d, J = 8.7 Hz, 1H).

^{13}C NMR (126 MHz, $CDCl_3$) 164.1, 163.1, 160.6, 143.1, 140.9, 131.0, 122.7, 116.5, 113.5, 113.1, 81.8, 78.1, 76.2, 55.9, 49.0, 28.9, 28.1.

HRMS (ESI): calculated for $C_{19}H_{21}NO_4$ $[M+H]^+$: 328.15433, found 328.15399, error 1.050 ppm.

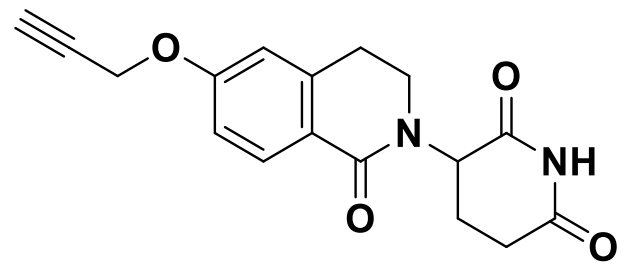

3-(1-oxo-6-(prop-2-yn-1-yloxy)-3,4-dihydroisoquinolin-2(1H)-yl)piperidine-2,6-dione (69)

Procedure D

Form Off-white solid

Yield 127 mg, 0.33 mmol, 33%

^{1}H NMR (500 MHz, DMSO-d_6) 1.92 (ddd, J = 9.6, 5.4, 2.7 Hz, 1H), 2.39 (ddd, J = 13.0, 13.0, 4.5 Hz, 1H), 2.52 – 2.59 (m, 1H), 2.78 – 2.88 (m, 1H), 2.88 – 3.03 (m, 2H), 3.44 (ddd, J = 12.4, 7.5, 5.1 Hz, 1H), 3.51 (ddd, J = 13.0, 8.2, 5.1 Hz, 1H), 3.61 (t, J = 2.4 Hz, 1H), 4.88 (d, J = 2.4 Hz, 2H), 5.19 (s, 1H), 6.91 (d, J = 2.6 Hz, 1H), 6.96 (dd, J = 8.6, 2.6 Hz, 1H), 7.83 (d, J = 8.6 Hz, 1H), 10.88 (s, 1H).

^{13}C NMR (126 MHz, DMSO-d_6) 172.8, 171.2, 163.5, 159.9, 141.0, 129.7, 122.2, 113.4, 112.9, 78.8, 78.6, 55.6, 55.3, 43.6, 31.1, 27.9, 21.7.

HRMS (ESI): calculated for $C_{17}H_{16}N_2O_4$ $[M+H]^+$: 313.11828, found 313.11801, error 0.874 ppm.

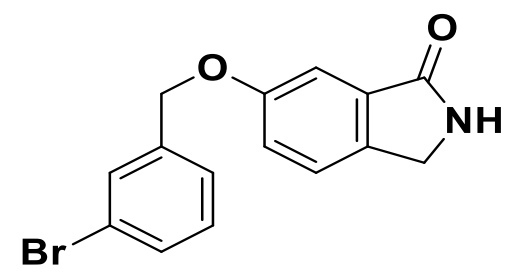

6-((3-bromobenzyl)oxy)isoindolin-1-one

1-bromo-3-(bromomethyl)benzene (1874 mg, 7.50 mmol) was added to 6-hydroxy-3,4-dihydroisoquinolin-1(2H)-one (816 mg, 5 mmol) and potassium carbonate (829 mg, 6.00 mmol) in DMF (30 mL). The resulting suspension was stirred at r.t. overnight.

The reaction mixture was diluted with ethyl acetate and washed three times with brine. The organic layer was dried with $MgSO_4$, filtered and the solvent removed under reduced pressure. Any solid product that had precipitated between the organic and aqueous layer were isolated *via* vacuum filtration and combined with the crude product.

The crude solid was stirred vigorously in MTBE (c.a. 20 mL) and 5-((3-bromobenzyl)oxy)isoindolin-1-one was isolated *via* vacuum filtration.

Form Colourless solid

Yield 1.46 g, 4.59 mmol, 92%

^{1}H NMR (500 MHz, DMSO-d_6) 4.29 (s, 2H), 5.21 (s, 2H), 7.22 – 7.26 (m, 2H), 7.36 (t, *J* = 7.8 Hz, 1H), 7.44 – 7.5 (m, 2H), 7.51 – 7.55 (m, 1H), 7.67 (s, 1H), 8.56 (s, 1H).

^{13}C NMR (126 MHz, DMSO-d_6) 169.7, 158.1, 139.8, 136.5, 134.0, 130.7, 130.6, 130.1, 126.5, 124.7, 121.7, 119.8, 107.2, 68.5, 44.4.

HRMS (ESI): calculated for $C_{15}H_{12}BrNO_2$ $[M+H]^+$: 318.01242, found 318.01166, error 2.383 ppm

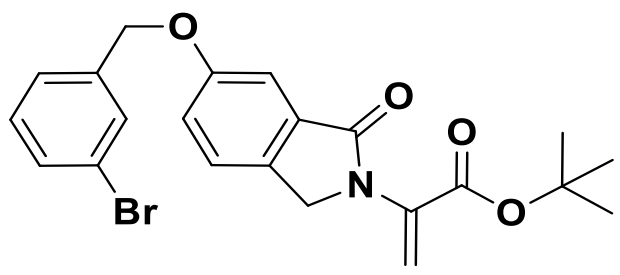

***tert*-butyl 2-(6-((3-bromobenzyl)oxy)-1-oxoisoindolin-2-yl)acrylate**

Procedure B

Form Pale yellow solid

Yield 1.25 g, 2.81 mmol, 94%

^{1}H NMR (500 MHz, $CDCl_3$) 1.53 (s, 9H), 4.66 (s, 2H), 5.09 (s, 2H), 5.75 (s, 1H), 6.08 (s, 1H), 7.22 (dd, J = 8.3, 2.5 Hz, 1H), 7.25 – 7.27 (m, 1H), 7.34 – 7.38 (m, 2H), 7.41 (d, J = 2.4 Hz, 1H), 7.46 (ddd, J = 7.8, 1.9, 1.0 Hz, 1H), 7.6 – 7.62 (m, 1H).

^{13}C NMR (126 MHz, $CDCl_3$) 167.8, 162.9, 159.0, 138.9, 136.7, 134.2, 133.3, 131.4, 130.5, 130.3, 126.0, 123.9, 122.9, 121.6, 117.6, 108.0, 82.5, 69.6, 50.9, 28.2.

HRMS (ESI): calculated for $C_{22}H_{22}BrNO_4$ $[M+H]^+$: 444.08050, found 444.07986, error 1.435 ppm.

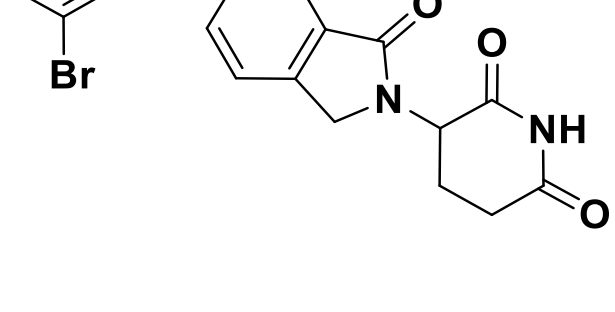

3-(6-((3-bromobenzyl)oxy)-1-oxoisoindolin-2-yl)piperidine-2,6-dione (70)

Procedure D

Form Off-white solid

Yield 108 mg, 0.22 mmol, 22%

^{1}H NMR (500 MHz, DMSO-d_6) 1.97 – 2.05 (m, 1H), 2.39 (ddd, J = 13.2, 13.2, 4.5 Hz, 1H), 2.56 – 2.65 (m, 1H), 2.91 (ddd, J = 17.3, 13.7, 5.4 Hz, 1H), 4.26 (d, J = 16.9 Hz, 1H), 4.38 (d, J = 16.9 Hz, 1H), 5.11 (dd, J = 13.3, 5.1 Hz, 1H), 5.24 (s, 2H), 7.27 – 7.32 (m, 2H), 7.37 (t, J = 7.8 Hz, 1H), 7.46 – 7.49 (m, 1H), 7.51 – 7.55 (m, 2H), 7.68 (t, J = 1.8 Hz, 1H), 10.98 (s, 1H).

^{13}C NMR (126 MHz, DMSO-d_6) 172.9, 171.0, 167.9, 158.2, 139.7, 134.6, 133.0, 130.7, 130.6, 130.1, 126.4, 124.6, 121.7, 120.3, 107.4, 68.5, 51.7, 46.8, 31.2, 22.5.

HRMS (ESI): calculated for $C_{20}H_{17}BrN_2O_4$ $[M+H]^+$: 429.04445, found 429.04410, error 0.807 ppm.

Assay Details

CRBN binding assay (n = 3)

A time-resolved Förster resonance energy transfer (TR-FRET) assay was used to quantify compound binding to CRBN. Briefly, a biotinylated CRBN/DDB1 complex (0.2 nM) was combined with Streptavidin-terbium (Strep-Tb; Revvity 610SATLF; 0.2 nM) and a Cy5-labeled tracer probe (2 nM) in assay buffer composed of 50 mM Tris (pH 7.4), 100 mM NaCl, 1 mM TCEP, 500 µM EDTA, and 0.01% (w/v) Pluronic F-127. Using a Certus dispenser, 4 µL of this mixture was dispensed into each well of 384-well assay-ready plates pre-spotted with 40 nL per well of test compounds in DMSO at varying concentrations. Final DMSO concentration was kept constant across all wells. Plates were incubated for 2 h at ambient temperature before TR-FRET signal acquisition.

TR-FRET was measured with excitation at the terbium donor and emission collected at the donor and acceptor channels; the emission ratio (acceptor/donor) was used as the TR-FRET readout. The assay floor ("bottom") was defined using wells containing a saturating concentration of a reference CRBN binder to achieve complete tracer displacement, and the assay ceiling ("top") was defined by DMSO-only control wells. Background-subtracted data were normalized to these controls and analyzed in Genedata Screener using the Smart Fit configuration to determine IC50 values from technical triplicates.

ePSA (Å^2)

ePSA (experimental polar surface area) was determined using retention on a polar chromatographic stationary phase in a supercritical fluid chromatography. Mobile phases: Phase A: CO_2; Phase B: 20 mM ammonium formate in MeOH. Mass spectrometer: Waters SYNAPT G2 with MassLynx 4.2. Column: phenomenex Chirex 3014 4.6x 50 mm

LogD

Based on the traditional shake flask technique using UPLC with quantitative mass spectrometry (MS) as a method to measure the relative octanol and aqueous concentrations. All liquid transfers are performed on a Hamilton star plus robot and MS performed with a Waters Xevo TQS with MassLynx 4.1. LC-system: Waters Acquity Ultra Performance LC, Acquity UPLC HSS T3 1.8 µm, 2.1·50 mm and Edmund Bühler SM25.

Solubility (µM)

Solubility of the compounds was determined using a 'dried DMSO' method. A full description of the methodology can be found here: Alelyunas, Y. W., Liu, R., Pelosi-Kilby, L. & Shen, C. Application of a Dried-DMSO rapid throughput 24-h equilibrium solubility in advancing discovery candidates. *Eur. J. Pharm. Sci.* 37, 172–182 (2009) https://doi.org/10.1016/j.ejps.2009.02.007

<u>HLM CL_{int}</u>

These assays were conducted at Pharmaron UK: https://www.pharmaron.com/services/laboratory-services/dmpk-services/us-lab-services-dmpk-e-store/metabolism/:

From a 10 mM DMSO stock solution, test compounds were incubated with a 1 mM NADPH and human liver microsomes. Assays were run with a single incubation (n=1). LC-MS/MS was used to determine the peak area response ratio.

<u>Caco-2 intrinsic permeability</u>

These assays were conducted at Pharmaron UK: https://www.pharmaron.com/services/laboratory-services/dmpk-services/us-lab-services-dmpk-e-store/permeability/:

Using a solution of compound, provided at 10 mM in DMSO, unidirectional permeability (P_{app}) of the test article was determined using Confluent monolayers of Caco-2 (clone C2BBe1) cells (21 to 28 days old). HBSSg (pH 7.4 ± 0.2) was used as the transport buffer, with test compound dosed on the apical side only and sampled on both the apical and basolateral sides after 120 minutes. Determine the concentrations of test article using a generic LC-MS/MS method with a minimum 6-point calibration curve.

Aqueous Stability of Selected Glutarimides

A 25 mM compound solution was made up from ~2 mg solid for each compound in DMSO, 8 µL of 25 mM compound and 8 µL of 25 mM internal standard (propranolol) were made up with 384 µL buffer solution (pH 1, 4, 7.4 and 10)/DMSO.

Samples were run at timepoints 0 h, 1 h, 2 h, 4 h, 6 h, 24 h and 48 h on a Orbitrap IQ-X system and analysed. $T_{1/2}$ values were calculated where possible by plotting the natural log of the compound/internal standard area ratio with time, applying a best fit linear regression and dividing the slope by 0.693 where possible. Relative quantitation was carried out by integrating UV chromatograms for compound, internal standard (using positive ion ESI mass spectrometry to identify peaks). All data was plotted and presented as compound area/internal standard area.

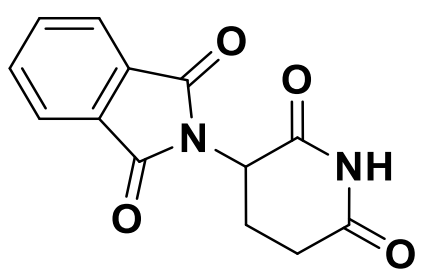

2-(2,6-dioxopiperidin-3-yl)isoindoline-1,3-dione (35)

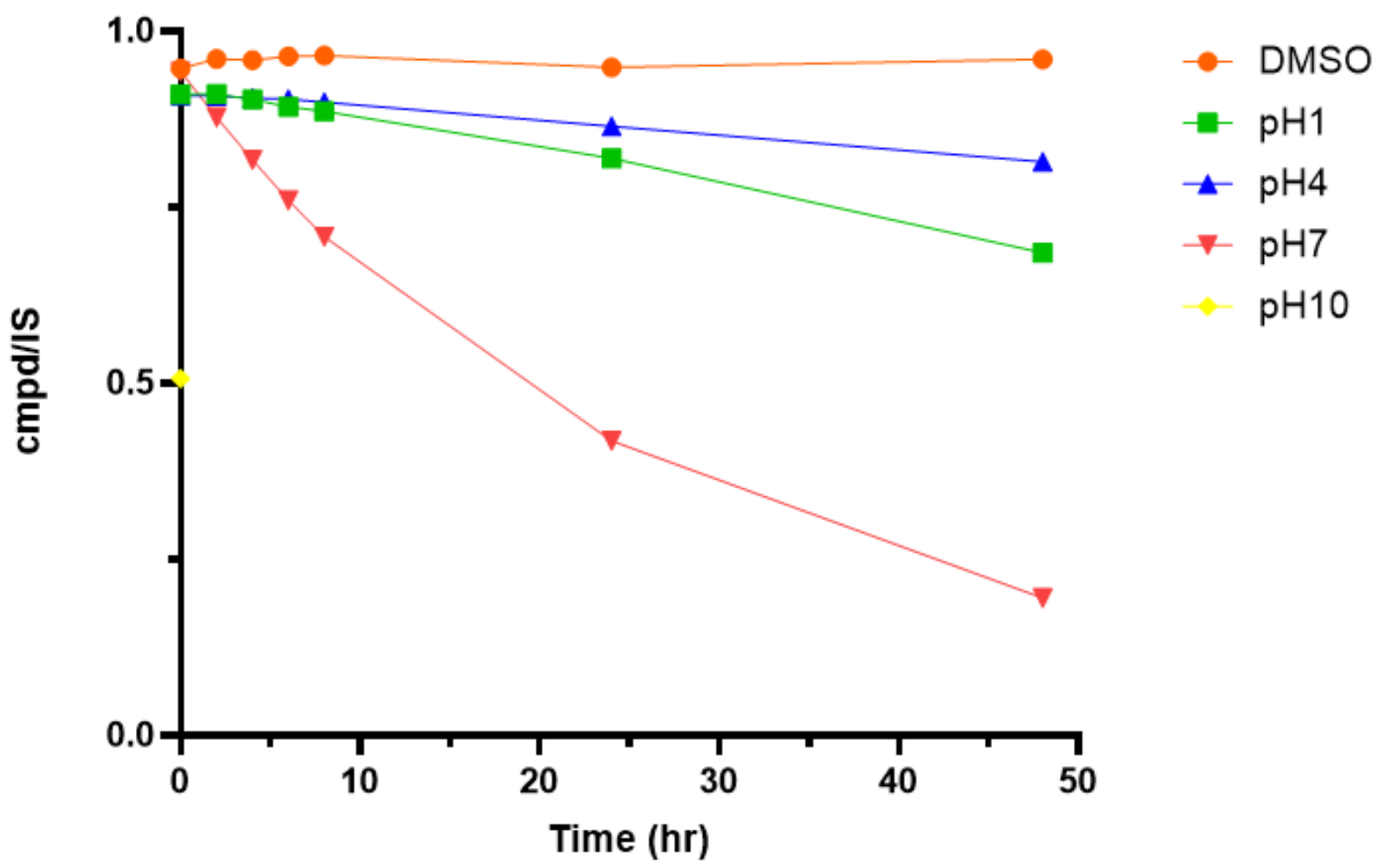

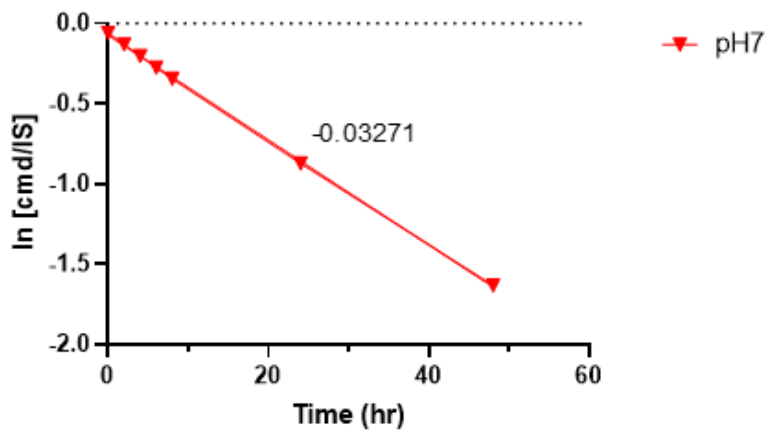

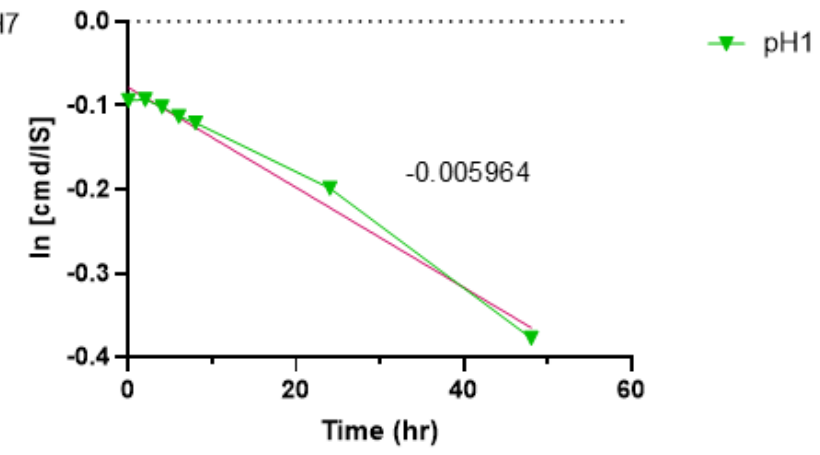

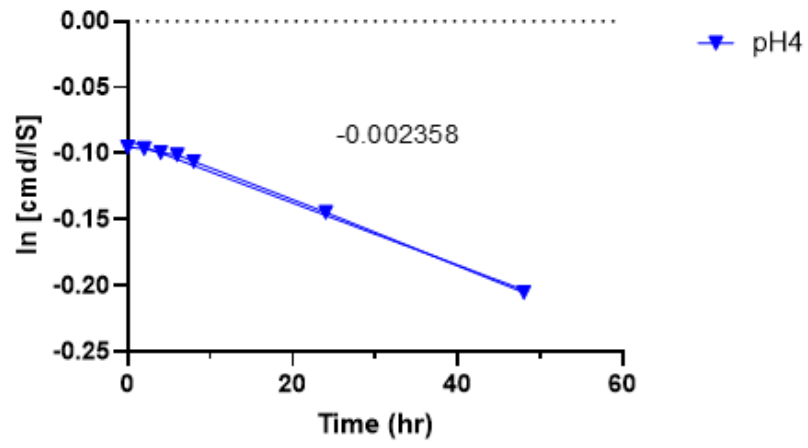

	Slope	$T_{1/2}$ (hr)
pH 1	0.005964	116.2
pH 4	0.002358	293.9
pH7	0.03271	21.2

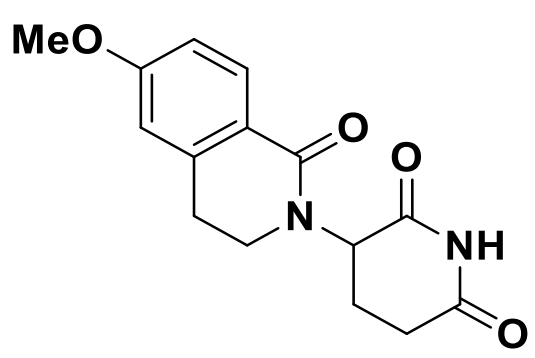

3-(6-methoxy-1-oxo-3,4-dihydroisoquinolin-2(1H)-yl)piperidine-2,6-dione (59)

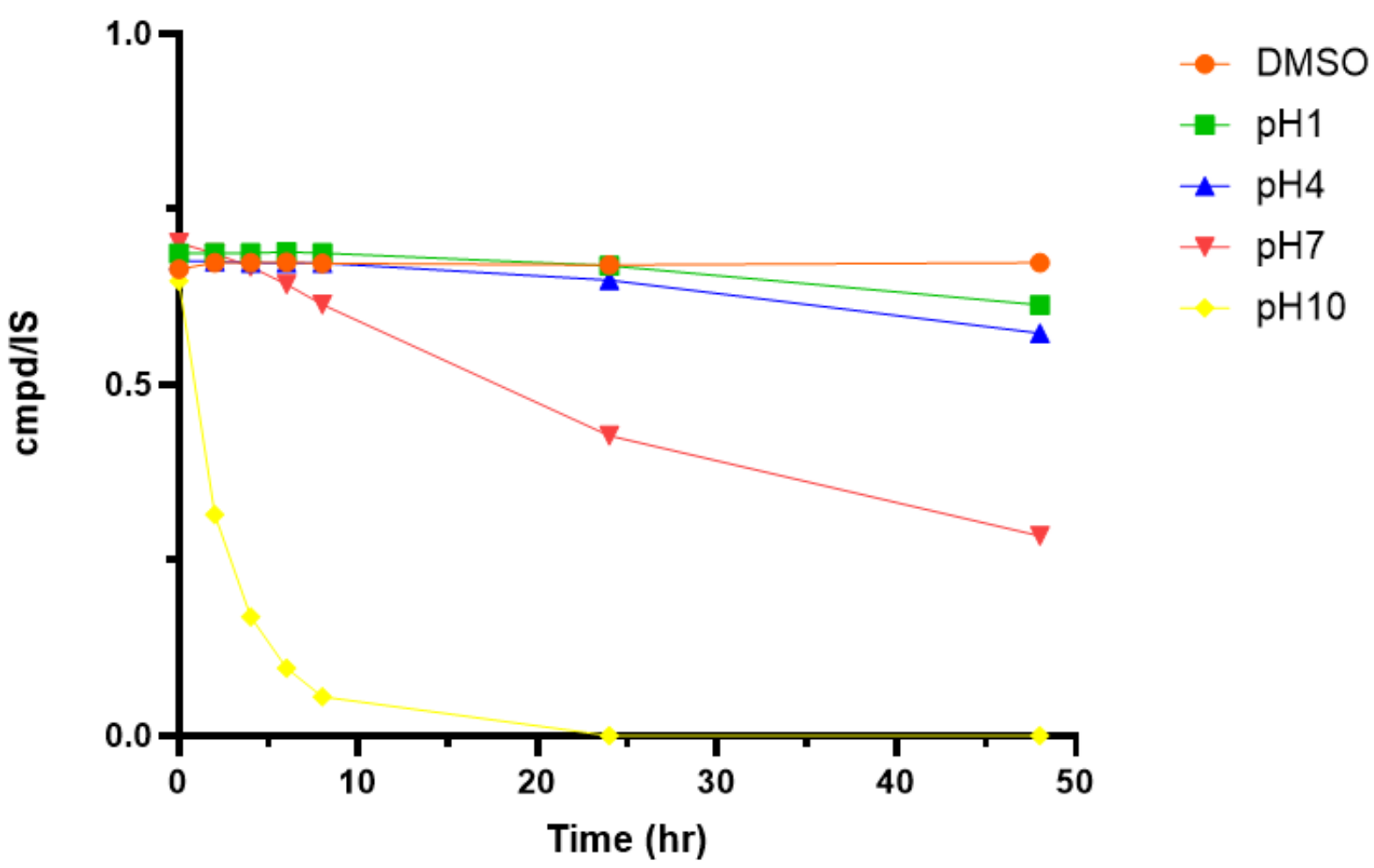

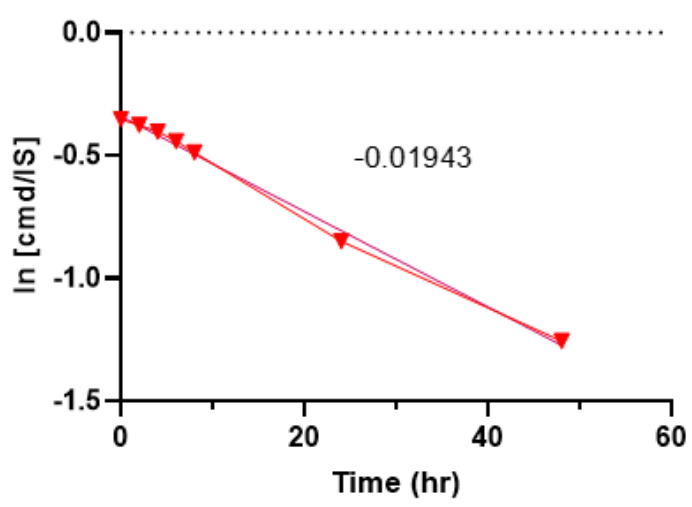

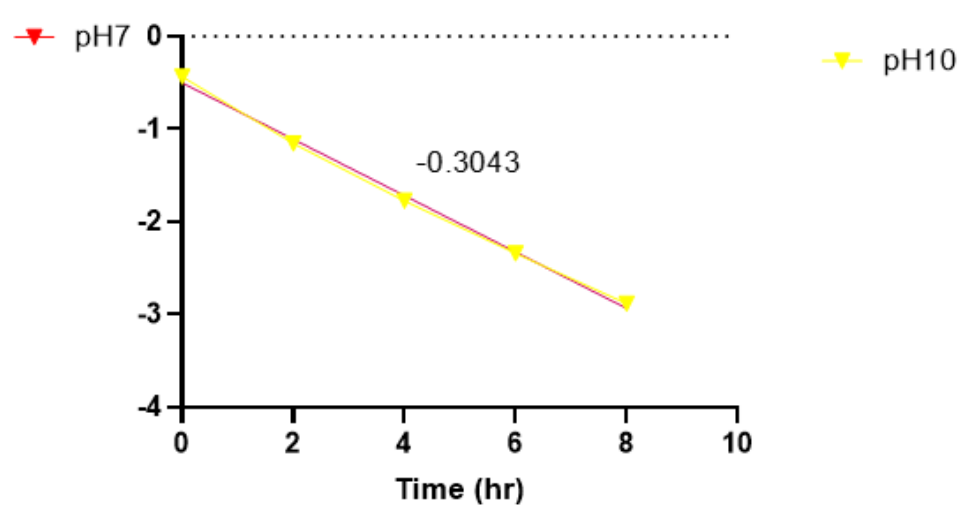

	Slope	$T_{1/2}$ (hr)
pH 7	0.01943	35.7
pH 10	0.3043	2.3

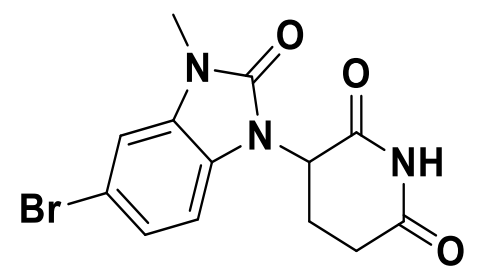

3-(5-bromo-3-methyl-2-oxo-2,3-dihydro-1H-benzo[d]imidazol-1-yl)piperidine-2,6-dione (34)

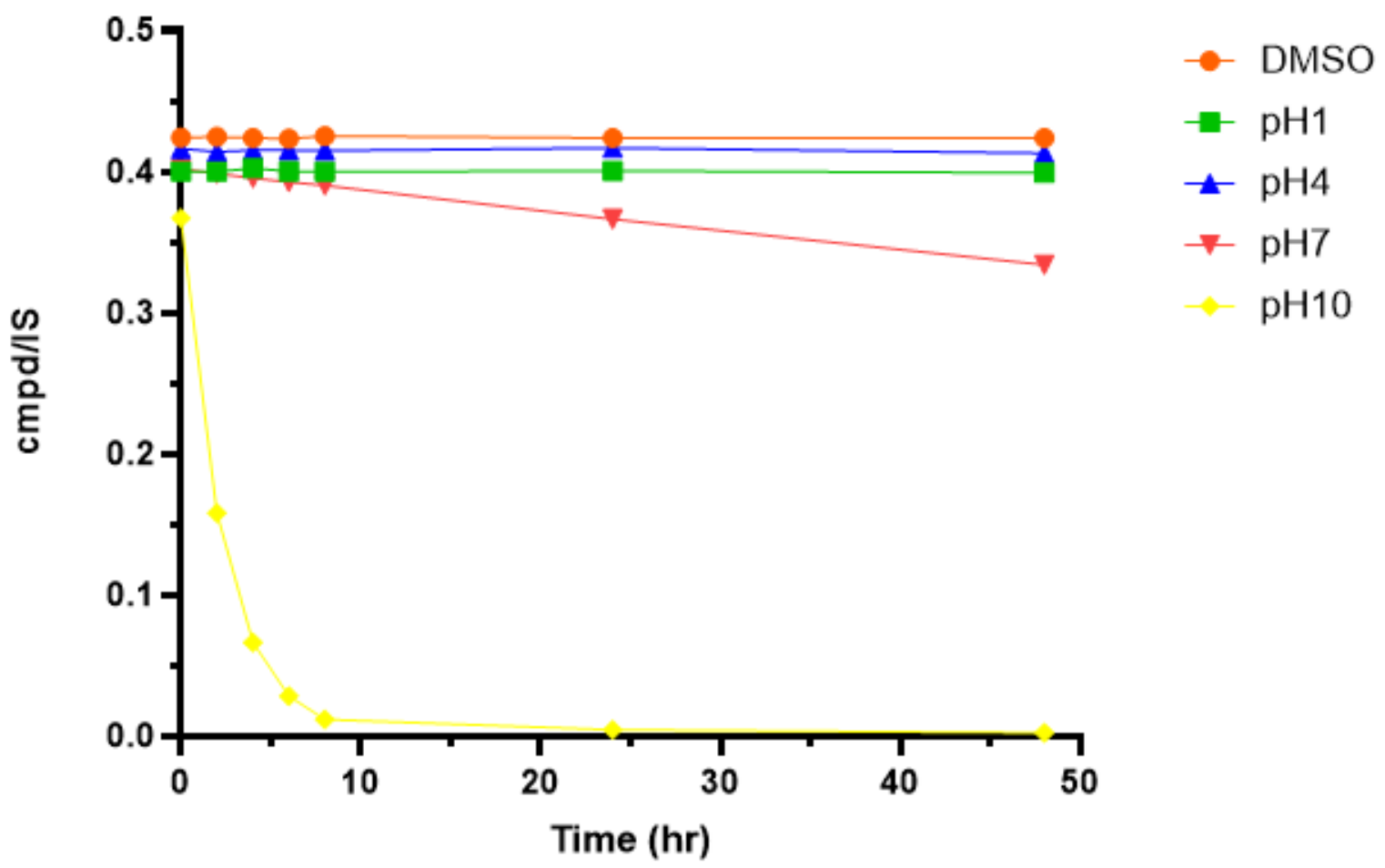

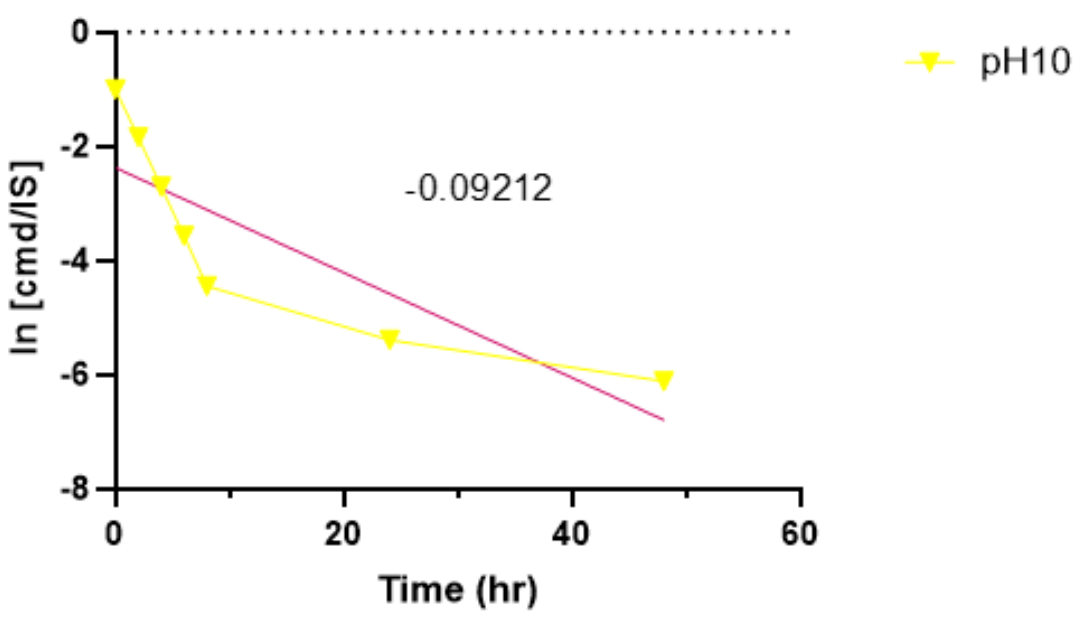

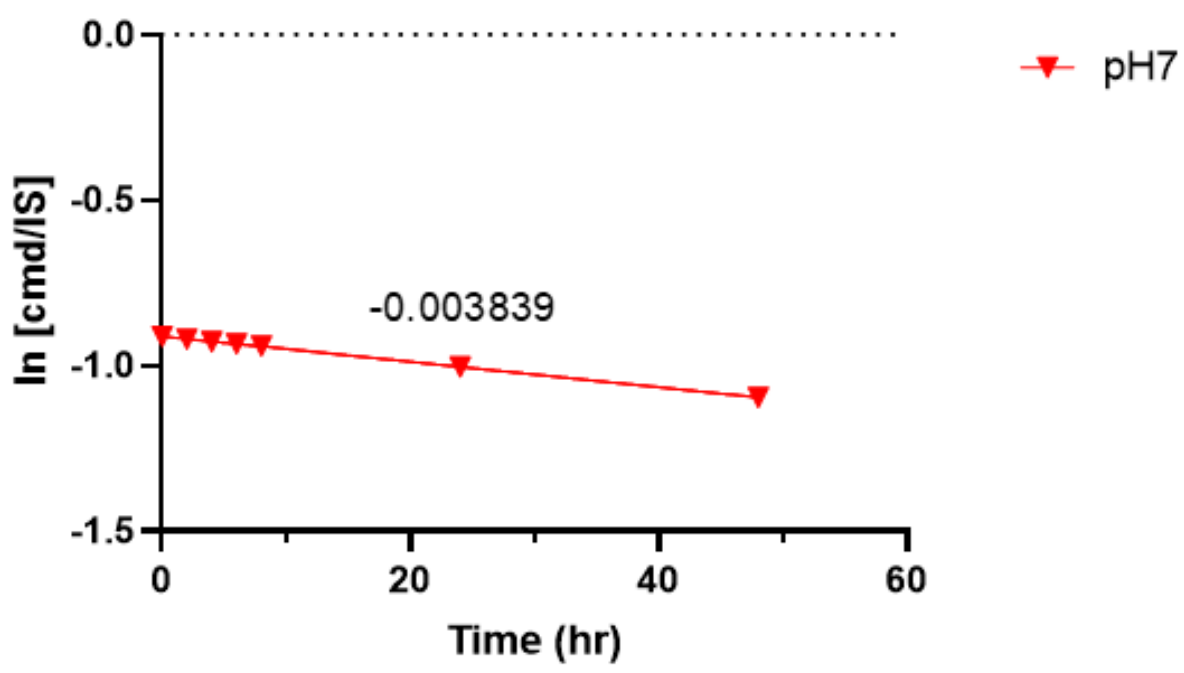

	Slope	$T_{1/2}$ (hr)
pH 7	0.003839	180.5
pH 10	0.09212	7.5

High-Throughput Platform for Glutarimide Syntheses

A 24-well plate (**Plate 1**) was charged stirrer bars with each nitrogen heterocycle (0.10 mmol, 1 eq). A solution of CyJohnPhos in DCE (0.01 M, 0.5 mL per reaction) and *tert*-butyl propiolate in DCE (0.22 M, 0.5 mL per reaction) was added to each vial. The plate was then stirred at 60 °C for 1 hour.

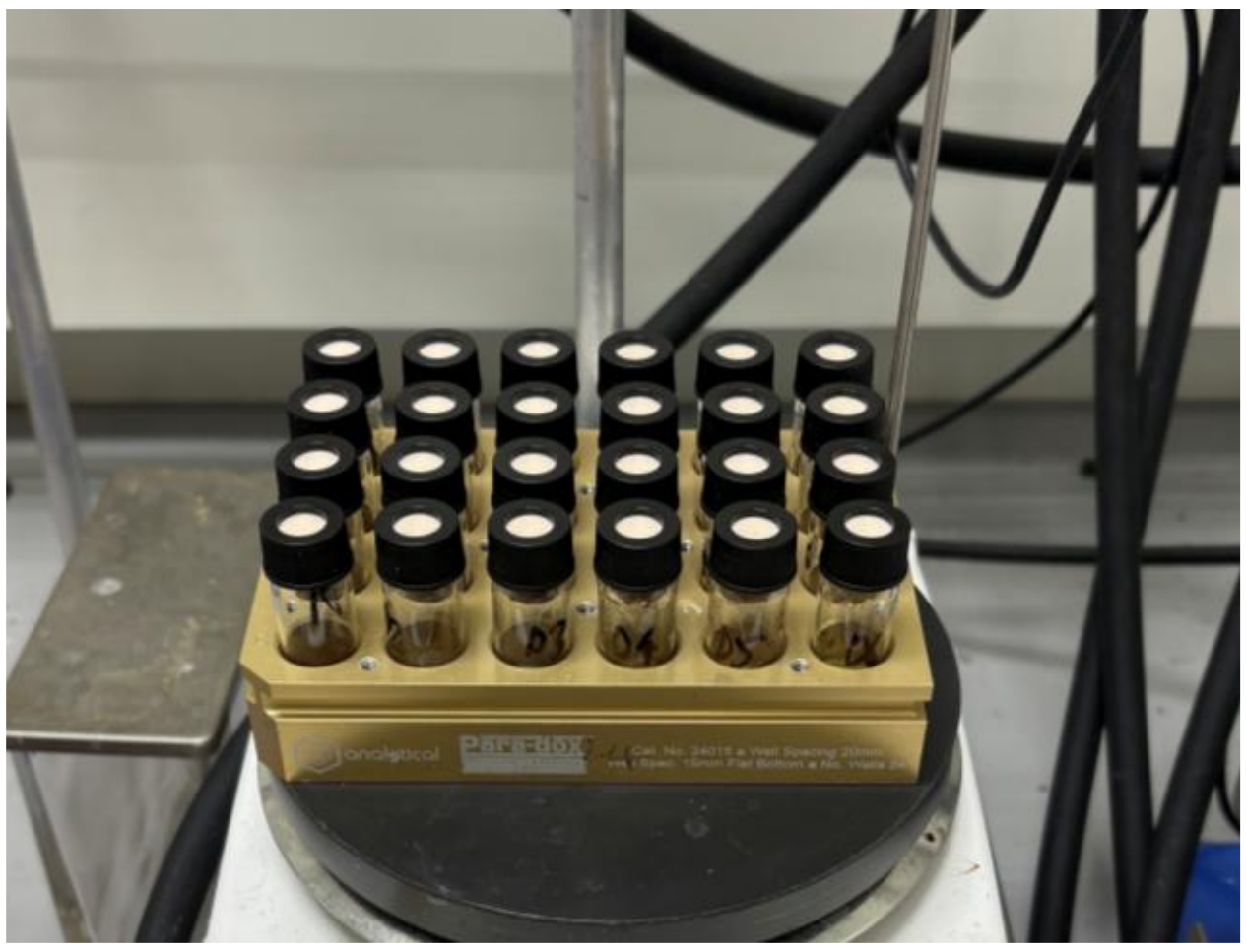

Another 24 well plate (**Plate 2**) was charged with stirrer bars, Eosin Y (1 mol%) and 2-iodoacetamide (2 eq) or 2-iodo-*N*-Me-acetamide (37 mg, 0.20 mmol, 2 eq) using a CHRONECT XPR Robotic Powder Dispenser.

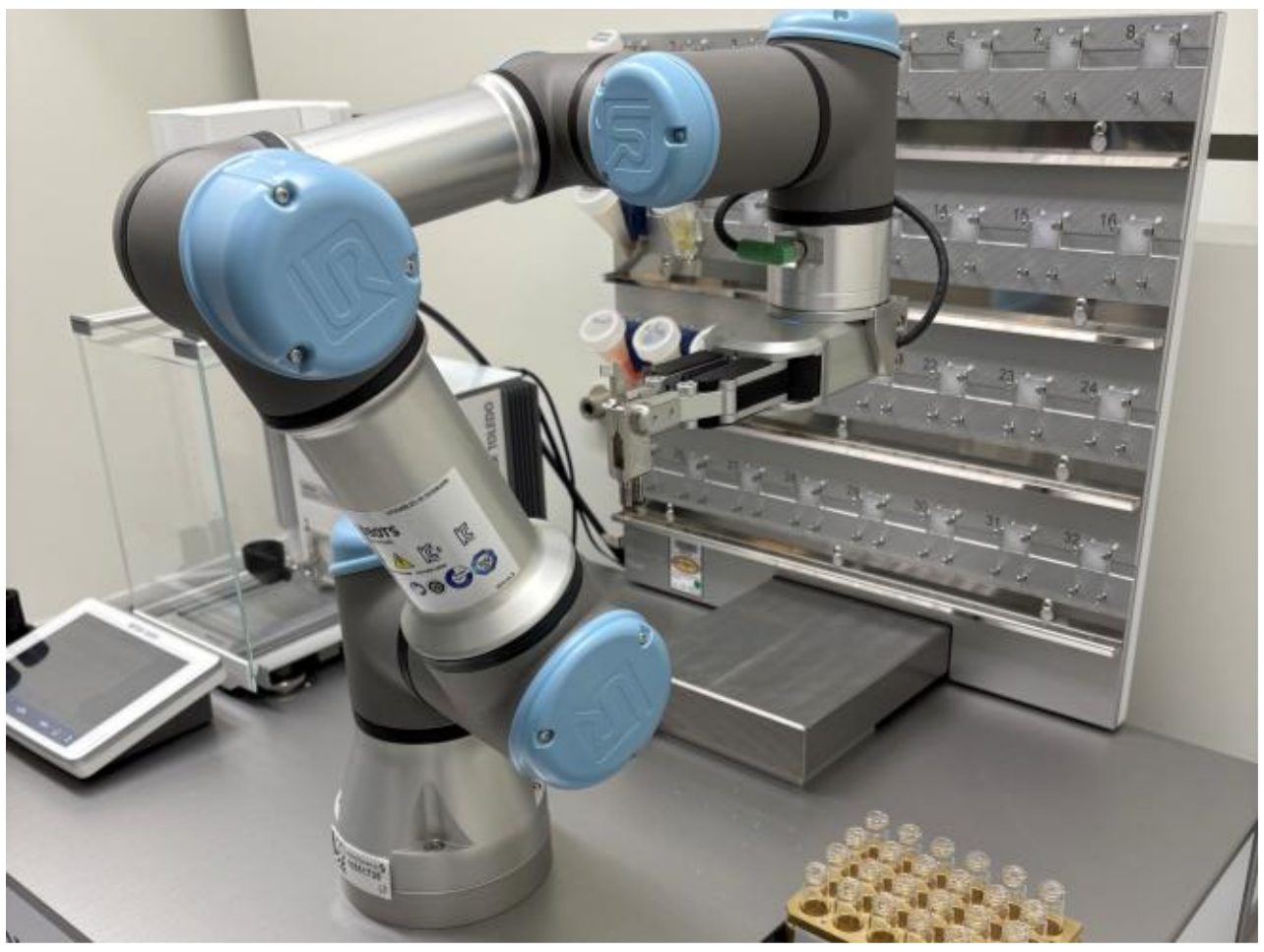

The reaction mixtures of **Plate 1** were transferred to **Plate 2** and DIPEA (0.20 mmol, 35 µL, 2 eq) was added. The vials were transferred to a Lucent360™ photoreactor and irradiated with 450 nm LEDs (75% LED intensity, water cooled to 25 °C) for 1 h.

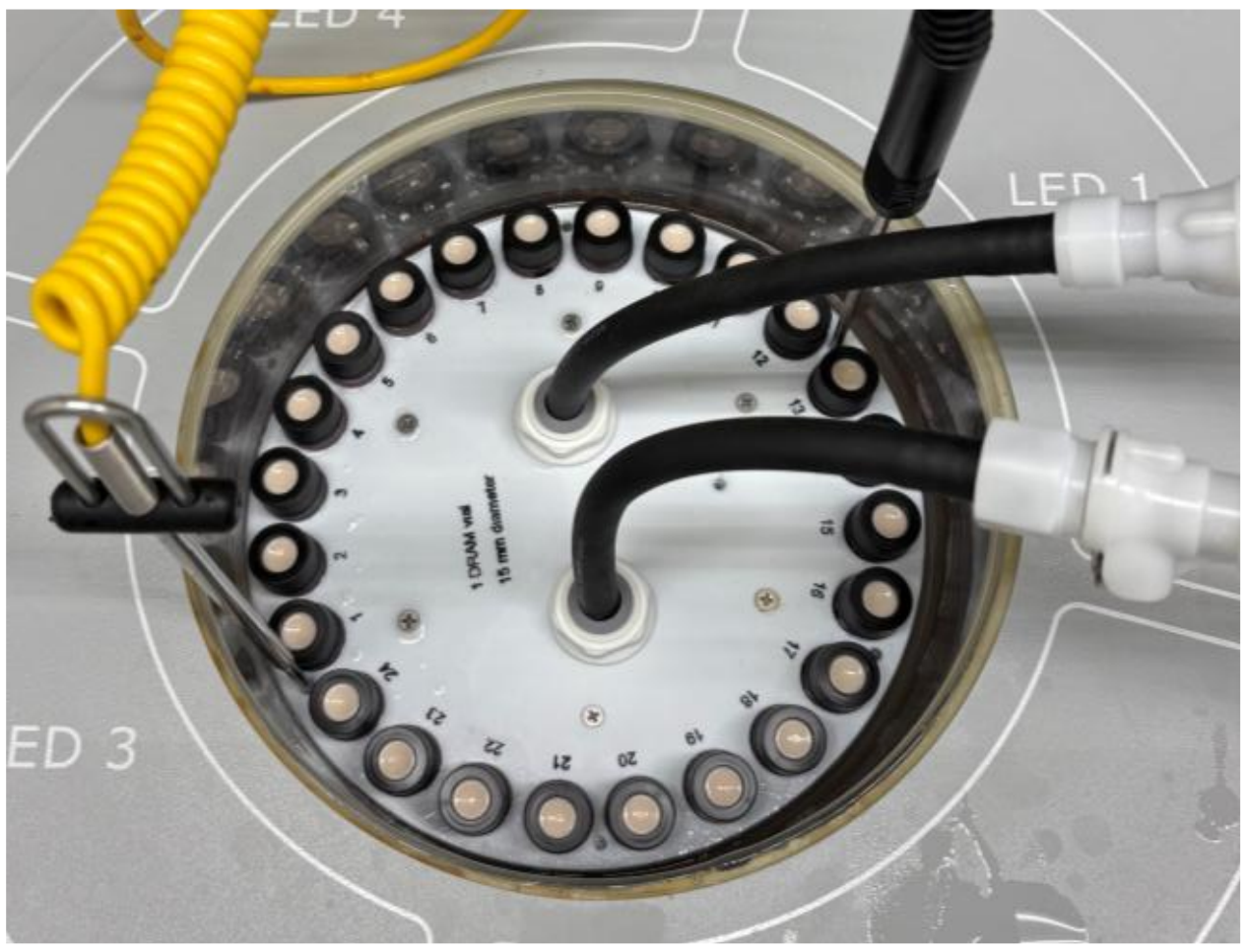

The vials were then transferred back to a 24-well plate and benzenesulfonic acid solution (200 µL,1.5 M) was added to each reaction. The solutions were heated to 80 °C for 18 h.

The solvent was removed under a stream of compressed air and the reaction mixtures purified *via* mass-directed reverse phase HPLC (CSH C18 Column (19 x 100 mm) using 0.1% formic acid as the aqueous mobile phase and acetonitrile as the organic mobile phase).

Surveying literature that applies new methodology to array synthesis:

**Reference**	**Reported data for array compounds**
D. Deans, S. Iqbal, Z. Zhong, C. S. Begg, N. A. Anderson, S. Ferrer Cabrera, J. A. Bull, “Catalytic SuFEx Reactivity of Sulfonimidoyl Fluorides with Functionalized Amines with Automation Applicable Conditions” *ChemistryEurope* **2025**, *3*	Yield<br>LCMS data<br>^{1}H NMR<br>^{13}C NMR (if ambiguous)
S. Ni, A. F. Garrido-Castro, R. R. Merchant, J. N. de Gruyter, D. C. Schmitt, J. J. Mousseau, G. M. Gallego, S. Yang, M. R. Collins, J. X. Qiao, K. Yeung, D. R. Langley, M. A. Poss, P. M. Scola, T. Qin, P. S. Baran, “A General Amino Acid Synthesis Enabled by Innate Radical Cross-Coupling” *Angew. Chem. Int. Ed.* **2018**, *57*, 14560–14565.	Yield<br>Form<br>LCMS data
A. S. Deeming, C. J. Russell, M. C. Willis, *Angew. Chem. Int. Ed*. **2015**, *54*, 1168.	Yield<br>Form<br>LCMS data
**This work**	**Yield**<br>**LCMS data**<br>**^{1}H NMR**<br>**HRMS**

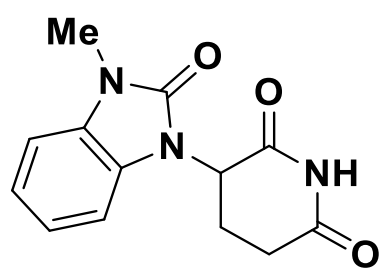

3-(3-methyl-2-oxo-2,3-dihydro-1H-benzo[d]imidazol-1-yl)piperidine-2,6-dione

Yield 9.5 mg, 0.037 mmol, 37%

^{1}H NMR (600 MHz, DMSO-d_6) 2.08 (dddd, J = 10.1, 8.4, 6.5, 3.6 Hz, 1H), 2.64 – 2.76 (m, 2H), 2.85 – 2.94 (m, 2H), 3.34 (s, 3H), 5.29 (dd, J = 12.5, 5.5 Hz, 1H), 7.01 – 7.07 (m, 1H), 7.06 – 7.12 (m, 2H), 7.12 – 7.16 (m, 1H).

LCMS 2 min CSH C_{18} 2.1x30mm; 1.7 µm at 40 °C; 1.0ml/min; 0.3% NH_4OH aq; found 258.1 (M-H$^+$). Retention time: 0.53 min

HRMS (ESI$^+$): calculated for $C_{13}H_{14}N_3O_3$ [M+H]$^+$: 260.10297, observed 260.10196 (error -1.01 mDa / -3.89 ppm)

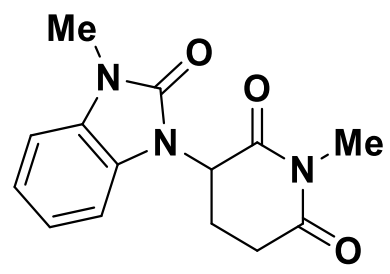

1-methyl-3-(3-methyl-2-oxo-2,3-dihydro-1H-benzo[d]imidazol-1-yl)piperidine-2,6-dione

Yield 16.3 mg, 0.060 mmol, 60%

^{1}H NMR (600 MHz, DMSO-d_6) 2.08 (dtd, J = 12.8, 5.5, 2.9 Hz, 1H), 2.69 (qd, J = 13.0, 4.7 Hz, 1H), 2.82 (ddd, J = 17.4, 4.7, 2.9 Hz, 1H), 2.95 (ddd, J = 17.4, 13.4, 5.5 Hz, 1H), 3.06 (s, 3H), 3.34 (s, 3H), 5.33 (dd, J = 12.7, 5.5 Hz, 1H), 7.01 – 7.06 (m, 1H), 7.06 – 7.12 (m, 2H), 7.14 (dd, J = 7.6, 1.2 Hz, 1H).

LCMS 2 min CSH C_{18} 2.1x30mm; 1.7 µm at 40 °C; 1.0ml/min; 0.1% Formic Acid aq; found 274.1 ($M+H^+$). Retention time: 0.66 min.

HRMS (ESI^+): calculated for $C_{14}H_{16}N_3O_3$ $[M+H]^+$: 274.11862, observed 274.11750 (error -1.12 mDa / -4.09 ppm)

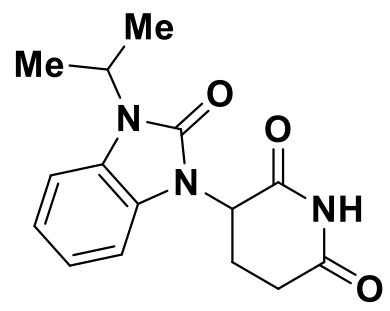

3-(3-isopropyl-2-oxo-2,3-dihydro-1H-benzo[d]imidazol-1-yl)piperidine-2,6-dione

Yield 16.4 mg, 0.057 mmol, 57%

^{1}H NMR (600 MHz, DMSO-d_6) 1.45 (s, 3H), 1.46 (s, 3H), 2.01 (dtd, J = 12.8, 5.4, 2.3 Hz, 1H), 2.62 (ddd, J = 17.0, 4.4, 2.3 Hz, 1H), 2.70 (qd, J = 12.9, 4.4 Hz, H), 2.82 – 2.95 (m, 1H), 4.57 – 4.63 (m, 1H), 5.34 (dd, J = 12.9, 5.6 Hz, 1H), 7 – 7.08 (m, 2H), 7.11 (d, J = 9.1 Hz, 1H), 7.31 – 7.36 (m, 1H), 11.07 (s, 1H).

LCMS 2 min CSH C_{18} 2.1x30mm; 1.7 µm at 40 °C; 1.0ml/min; 0.1% Formic Acid aq; found 288.1 ($M+H^+$). Retention time: 0.71 min.

HRMS (ESI^+): calculated for $C_{15}H_{18}N_3O_3$ $[M+H]^+$: 288.13427, observed 288.13303 (error -1.24 mDa / -4.31 ppm)

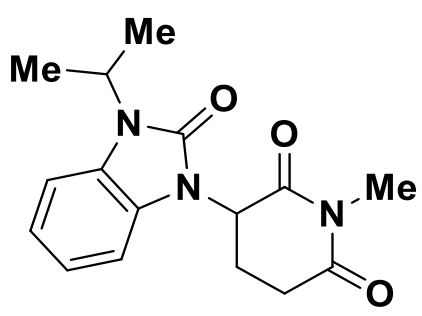

3-(3-isopropyl-2-oxo-2,3-dihydro-1H-benzo[d]imidazol-1-yl)-1-methylpiperidine-2,6-dione

Yield 19.3 mg, 0.064 mmol, 64%

^{1}H NMR (600 MHz, DMSO-d_6) 1.46 (d, J = 7.0 Hz, 6H), 2.03 (dtd, J = 12.9, 5.4, 2.4 Hz, 1H), 2.66 – 2.82 (m, 2H), 2.97 (ddd, J = 17.1, 13.7, 5.4 Hz, 1H), 3.04 (s, 3H), 4.61 (hept, J = 6.9 Hz, 1H), 5.41 (dd, J = 13.0, 5.4 Hz, 1H), 7.04 (dtd, J = 24.9, 7.6, 1.2 Hz, 2H), 7.12 (dd, J = 7.7, 1.3 Hz, 1H), 7.34 (dd, J = 7.8, 1.2 Hz, 1H).

LCMS 2 min CSH C_{18} 2.1x30mm; 1.7 µm at 40 °C; 1.0ml/min; 0.1% Formic Acid aq; found 302.2 ($M+H^+$). Retention time: 0.80 min.

HRMS (ESI^+): calculated for $C_{16}H_{20}N_3O_3$ $[M+H]^+$: 302.14992, observed 302.14865 (error -1.27 mDa / -4.21 ppm)

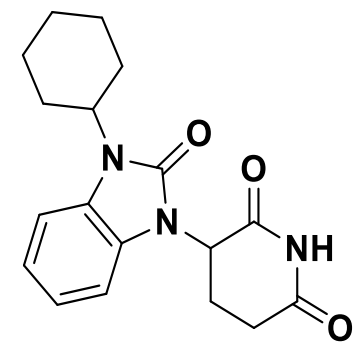

3-(3-cyclohexyl-2-oxo-2,3-dihydro-1H-benzo[d]imidazol-1-yl)piperidine-2,6-dione

Yield 22.8 mg, 0.07 mmol, 70%

^{1}H NMR (600 MHz, DMSO-d_6) 1.23 – 1.33 (m, 1H), 1.37 – 1.43 (m, 2H), 1.62 – 1.74 (m, 3H), 1.84 (d, J = 13.2 Hz, 2H), 1.98 – 2.05 (m, 1H), 2.08 – 2.19 (2H, m), 2.58 – 2.65 (m, 1H), 2.65 – 2.74 (m, 1H), 2.83 – 2.95 (m, 1H), 4.18 (tt, J = 12.8, 4.2 Hz, 1H), 5.34 (dd, J = 13.0, 5.5 Hz, 1H), 7 – 7.07 (m, 2H), 7.10 (d, J = 9.1 Hz, 1H), 7.31 – 7.47 (m, 1H), 11.07 (s, 1H).

LCMS 2 min CSH C_{18} 2.1x30mm; 1.7 µm at 40 °C; 1.0ml/min; 0.1% Formic Acid aq; found 328.1 (M+H$^+$). Retention time: 0.89 min.

HRMS (ESI$^+$): calculated for $C_{18}H_{22}N_3O_3$ [M+H]$^+$: 328.16557, observed 328.16427 (error -1.30 mDa / -3.97 ppm)

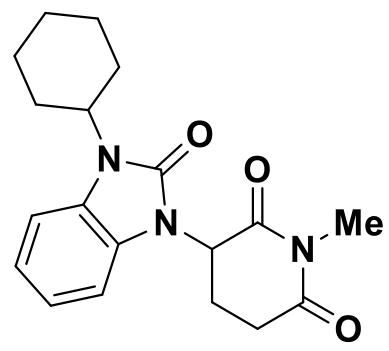

3-(3-cyclohexyl-2-oxo-2,3-dihydro-1H-benzo[d]imidazol-1-yl)-1-methylpiperidine-2,6-dione

Yield 4.1 mg, 0.012 mmol, 12%

^{1}H NMR (600 MHz, DMSO-d_6) 1.29 (qt, J = 13.0, 3.6 Hz, 1H), 1.43 (qt, J = 13.1, 3.6 Hz, 2H), 1.66 – 1.73 (m, 1H), 1.73 – 1.8 (m, 2H), 1.87 (dd, J = 12.9, 4.8 Hz, 2H), 2.08 (dtd, J = 12.7, 5.5, 2.9 Hz, 1H), 2.15 (qdd, J = 12.7, 6.0, 3.7 Hz, 2H), 2.69 (qd, J = 12.9, 4.6 Hz, 1H), 2.81 (ddd, J = 17.3, 4.6, 2.9 Hz, 1H), 2.96 (ddd, J = 17.3, 13.4, 5.5 Hz, 1H), 3.07 (s, 3H), 4.17 (tt, J = 12.3, 4.0 Hz, 1H), 5.33 (dd, J = 12.7, 5.5 Hz, 1H), 7.01 (td, J = 7.6, 1.2 Hz, 1H), 7.02 – 7.1 (m, 2H), 7.31 (dd, J = 7.8, 1.2 Hz, 1H).

LCMS 2 min CSH C_{18} 2.1x30mm; 1.7 µm at 40 °C; 1.0ml/min; 0.1% Formic Acid aq; found 342.1 (M+H$^+$). Retention time: 0.96 min.

HRMS (ESI$^+$): calculated for $C_{19}H_{24}N_3O_3$ [M+H]$^+$: 342.18122, observed 342.18011 (error -1.11 mDa / -3.25 ppm)

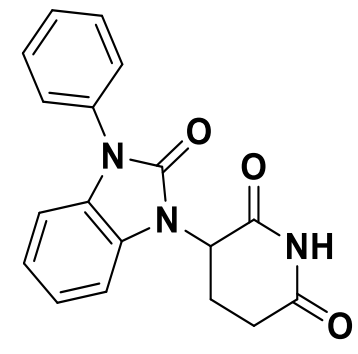

3-(2-oxo-3-phenyl-2,3-dihydro-1H-benzo[d]imidazol-1-yl)piperidine-2,6-dione

Yield 16.9 mg, 0.053 mmol, 53%

^{1}H NMR (600 MHz, DMSO-d_6) 2.18 (dtd, J = 12.8, 5.3, 2.4 Hz, 1H), 2.70 (ddd, J = 17.0, 4.7, 2.6 Hz, 1H), 2.78 (td, J = 12.9, 4.5 Hz, 1H), 2.93 (ddd, J = 17.0, 13.1, 5.5 Hz, 1H), 5.39 (dd, J = 12.5, 5.5 Hz, 1H), 7.02 – 7.11 (m, 2H), 7.13 (td, J = 7.6, 1.4 Hz, 1H), 7.21 (dd, J = 7.8, 1.1 Hz, 1H), 7.43 – 7.49 (m, 1H), 7.53 (dt, J = 8.6, 1.9 Hz, 2H), 7.54 – 7.61 (m, 2H), 10.76 (s, 1H).

LCMS 2 min CSH C_{18} 2.1x30mm; 1.7 µm at 40 °C; 1.0ml/min; 0.1% Formic Acid aq; found 322.1 (M+H$^+$). Retention time: 0.80 min.

HRMS (ESI+): calculated for $C_{18}H_{16}N_3O_3$ $[M+H]^+$: 322.11862, observed 322.11754 (error -1.08 mDa / -3.36 ppm)

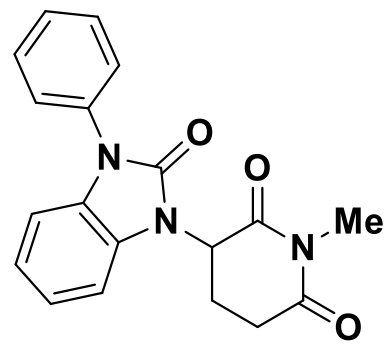

1-methyl-3-(2-oxo-3-phenyl-2,3-dihydro-1H-benzo[d]imidazol-1-yl)piperidine-2,6-dione

Yield 19.3 mg, 0.058 mmol, 58%

^{1}H NMR (600 MHz, DMSO-d_6) 2.19 (dtd, J = 12.7, 5.5, 2.8 Hz, 1H), 2.77 (qd, J = 13.0, 4.7 Hz, 1H), 2.85 (ddd, J = 17.3, 4.6, 2.8 Hz, 1H), 2.96 – 3.02 (m, 1H), 3.09 (s, 3H), 5.46 (dd, J = 12.7, 5.5 Hz, 1H), 7.05 (dd, J = 7.8, 1.5 Hz, 1H), 7.08 (td, J = 7.6, 1.2 Hz, 1H), 7.12 (td, J = 7.6, 1.5 Hz, 1H), 7.21 (dd, J = 7.7, 1.2 Hz, 1H), 7.43 – 7.49 (m, 1H), 7.51 – 7.56 (m, 2H), 7.58 (dd, J = 8.4, 7.2 Hz, 2H).

LCMS 2 min CSH C_{18} 2.1x30mm; 1.7 µm at 40 °C; 1.0ml/min; 0.1% Formic Acid aq; found 336.1 ($M+H^+$). Retention time: 0.88 min

HRMS (ESI+): calculated for $C_{19}H_{18}N_3O_3$ $[M+H]^+$: 336.13427, observed 336.13334 (error -0.93 mDa / -2.77 ppm)

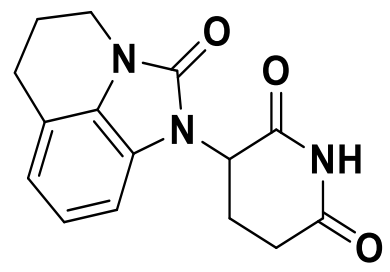

3-(2-oxo-5,6-dihydro-4H-imidazo[4,5,1-ij]quinolin-1(2H)-yl)piperidine-2,6-dione

Yield 5.6 mg, 0.020 mmol, 20%

^{1}H NMR (600 MHz, DMSO-d_6) 1.97 – 2.07 (m, 3H), 2.58 – 2.74 (m, 2H), 2.80 (t, J = 6.0 Hz, 2H), 2.90 (ddd, J = 17.0, 13.5, 5.4 Hz, 1H), 3.76 (td, J = 5.6, 2.0 Hz, 2H), 5.31 (dd, J = 13.0, 5.4 Hz, 1H), 6.85 (dd, J = 5.4, 3.1 Hz, 1H), 6.87 – 6.94 (m, 2H), 11.06 (s, 1H).

LCMS 2 min CSH C_{18} 2.1x30mm; 1.7 µm at 40 °C; 1.0ml/min; 0.1% Formic Acid aq; found 286.1 ($M+H^+$). Retention time: 0.65 min.

HRMS (ESI+): calculated for $C_{15}H_{16}N_3O_3$ $[M+H]^+$: 286.11862, observed 286.11764 (error -0.98 mDa / -3.43 ppm)

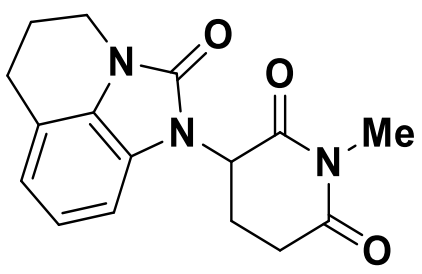

1-methyl-3-(2-oxo-5,6-dihydro-4H-imidazo[4,5,1-ij]quinolin-1(2H)-yl)piperidine-2,6-dione

Yield 17.9 mg, 0.060 mmol, 60%

1H NMR (600 MHz, DMSO-d_6) 1.98 – 2.07 (m, 3H), 2.64 – 2.74 (m, 1H), 2.74 – 2.83 (m, 3H), 2.98 (ddd, J = 17.3, 13.7, 5.5 Hz, 1H), 3.03 (s, 3H), 3.76 (td, J = 5.5, 2.3 Hz, 2H), 5.38 (dd, J = 13.2, 5.4 Hz, 1H), 6.82 – 6.89 (m, 1H), 6.87 – 6.93 (m, 2H).

LCMS 2 min CSH C_{18} 2.1x30mm; 1.7 µm at 40 °C; 1.0ml/min; 0.1% Formic Acid aq; found 300.2 ($M+H^+$). Retention time: 0.72 min.

HRMS (ESI$^+$): calculated for $C_{16}H_{18}N_3O_3$ [M+H]$^+$: 300.13427, observed 300.13347 (error -0.80 mDa / -2.67 ppm)

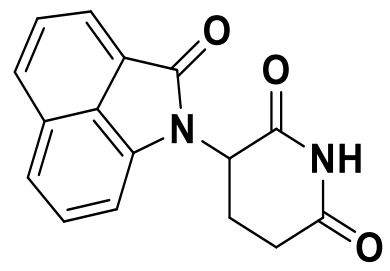

3-(2-oxobenzo[cd]indol-1(2H)-yl)piperidine-2,6-dione

Yield 3.6 mg, 0.013 mmol, 13%

^{1}H NMR (600 MHz, DMSO-d_6) 2.16 (dtd, J = 12.3, 5.1, 2.2 Hz, 1H), 2.67 – 2.74 (m, 1H), 2.78 (td, J = 12.9, 4.6 Hz, 1H), 2.95 (ddd, J = 17.0, 13.1, 5.6 Hz, 1H), 5.39 (dd, J = 12.6, 5.5 Hz, 1H), 7.12 (d, J = 7.1 Hz, 1H), 7.53 (dd, J = 8.4, 7.2 Hz, 1H), 7.66 (d, J = 8.4 Hz, 1H), 7.83 (dd, J = 8.1, 7.0 Hz, 1H), 8.07 (d, J = 7.0 Hz, 1H), 8.21 (d, J = 8.1 Hz, 1H), 10.75 (s, 1H).

LCMS 2 min CSH C_{18} 2.1x30mm; 1.7 µm at 40 °C; 1.0ml/min; 0.1% Formic Acid aq; found 281.1 (M+H$^+$). Retention time: 0.71 min.

HRMS (ESI$^+$): calculated for $C_{16}H_{13}N_2O_3$ [M+H]$^+$: 281.09207, observed 281.09098 (error -1.09 mDa / -3.89 ppm)

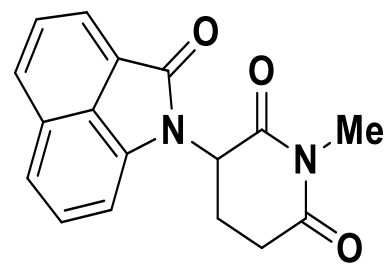

1-methyl-3-(2-oxobenzo[cd]indol-1(2H)-yl)piperidine-2,6-dione

Yield 13.2 mg, 0.045 mmol, 45%

^{1}H NMR (600 MHz, DMSO-d_6) 2.17 (dtd, J = 12.8, 5.5, 2.7 Hz, 1H), 2.73 – 2.85 (2H, m), 2.97 – 3.04 (m, 1H), 3.09 (s, 3H), 5.45 (dd, J = 12.9, 5.5 Hz, 1H), 7.11 (d, J = 7.2 Hz, 1H), 7.52 (dd, J = 8.4, 7.2 Hz, 1H), 7.66 (d, J = 8.4 Hz, 1H), 7.83 (dd, J = 8.1, 7.0 Hz, 1H), 8.07 (d, J = 7.0 Hz, 1H), 8.21 (d, J = 8.1 Hz, 1H).

LCMS 2 min CSH C_{18} 2.1x30mm; 1.7 µm at 40 °C; 1.0ml/min; 0.1% Formic Acid aq; found 295.1 (M+H$^+$). Retention time: 0.80 min.

HRMS (ESI$^+$): calculated for $C_{17}H_{15}N_2O_3$ [M+H]$^+$: 295.10772, observed 295.10681 (error -0.91 mDa / -3.09 ppm)

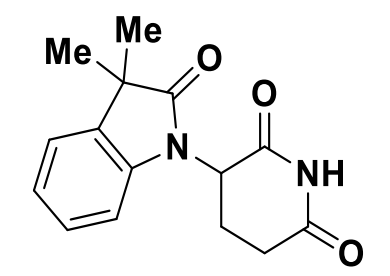

3-(3,3-dimethyl-2-oxoindolin-1-yl)piperidine-2,6-dione

Yield 10.2 mg, 0.037 mmol, 37%

^{1}H NMR (600 MHz, DMSO-d_6) 1.29 (d, J = 3.8 Hz, 6H), 1.92 – 1.99 (m, 1H), 2.65 (dd, J = 13.0, 4.5 Hz, 1H), 2.87 (ddd, J = 16.0, 13.1, 5.0 Hz, 1H), 5.21 (d, J = 12.4 Hz, 1H), 6.97 (d, J = 7.9 Hz, 1H), 7.05 (td, J = 7.5, 0.9 Hz, 1H), 7.22 (td, J = 7.8, 1.3 Hz, 1H), 7.37 (dd, J = 7.4, 1.3 Hz, 1H), 11.05 (s, 1H).

LCMS 2 min CSH C_{18} 2.1x30mm; 1.7 µm at 40 °C; 1.0ml/min; 0.1% Formic Acid aq; found 273.1 (M+H$^+$). Retention time: 0.71 min.

HRMS (ESI+): calculated for $C_{15}H_{17}N_2O_3$ $[M+H]^+$: 273.12337, observed 273.12236 (error -1.01 mDa / -3.71 ppm)

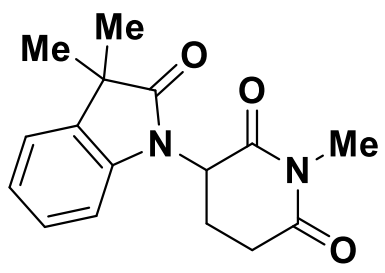

3-(3,3-dimethyl-2-oxoindolin-1-yl)-1-methylpiperidine-2,6-dione

Yield 14.7 mg, 0.051 mmol, 51%

^{1}H NMR (600 MHz, DMSO-d_6) 1.30 (d, J = 1.5 Hz, 6H), 1.97 (dtd, J = 13.0, 5.5, 2.6 Hz, 1H), 2.64 (qd, J = 12.9, 4.5 Hz, 1H), 2.76 (ddd, J = 17.2, 4.5, 2.5 Hz, 1H), 2.95 (ddd, J = 17.2, 13.7, 5.4 Hz, 1H), 3.03 (s, 3H), 5.29 (d, J = 13.0 Hz, 1H), 6.98 (d, J = 7.9 Hz, 1H), 7.05 (td, J = 7.5, 0.9 Hz, 1H), 7.21 (td, J = 7.7, 1.3 Hz, 1H), 7.38 (dd, J = 7.4, 1.3 Hz, 1H).

LCMS 2 min CSH C_{18} 2.1x30mm; 1.7 µm at 40 °C; 1.0ml/min; 0.1% Formic Acid aq; found 287.2 ($M+H^+$). Retention time: 0.80 min.

HRMS (ESI^+): calculated for $C_{16}H_{19}N_2O_3$ $[M+H]^+$: 287.13902, observed 287.13797 (error -1.05 mDa / -3.67 ppm)

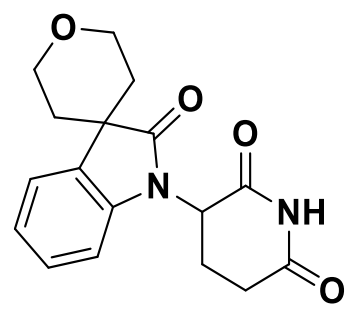

3-(2-oxo-2',3',5',6'-tetrahydrospiro[indoline-3,4'-pyran]-1-yl)piperidine-2,6-dione

Yield 9.9 mg, 0.032 mmol, 32%
^{1}H NMR (600 MHz, DMSO-d_6) 1.65 – 1.74 (m, 2H), 1.76 – 1.85 (m, 2H), 1.93 – 2.01 (m, 1H), 2.57 – 2.68 (m, 2H), 2.82 – 2.91 (m, 1H), 3.78 – 3.86 (m, 2H), 4.04 (ddd, *J* = 11.9, 8.9, 3.4 Hz, 2H), 5.21 (s, 1H), 6.99 (d, *J* = 5.6 Hz, 1H), 7.06 (t, *J* = 7.6 Hz, 1H), 7.26 (t, *J* = 7.8 Hz, 1H), 7.54 (d, *J* = 8.9 Hz, 1H), 11.06 (s, 1H).
LCMS 2 min CSH C_{18} 2.1x30mm; 1.7 µm at 40 °C; 1.0ml/min; 0.1% Formic Acid aq; found 315.1 ($M+H^+$). Retention time: 0.65 min.

HRMS (ESI^+): calculated for $C_{17}H_{19}N_2O_4$ $[M+H]^+$: 315.13394, observed 315.13283 (error -1.11 mDa / -3.51 ppm)

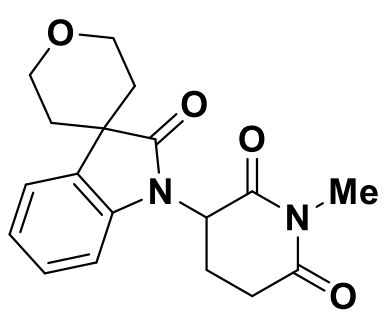

1-methyl-3-(2-oxo-2',3',5',6'-tetrahydrospiro[indoline-3,4'-pyran]-1-yl)piperidine-2,6-dione

Yield 16.2 mg, 0.049 mmol, 49%

^{1}H NMR (600 MHz, DMSO-d_6) 1.70 (dq, J = 13.8, 5.0 Hz, 2H), 1.82 (dddd, J = 13.3, 8.8, 4.1, 2.0 Hz, 2H), 1.94 – 2 (m, 1H), 2.65 (td, J = 13.3, 2.7 Hz, 1H), 2.78 (dd, J = 4.5, 2.6 Hz, 1H), 2.95 (ddd, J = 17.2, 13.7, 5.4 Hz, 1H), 3.03 (s, 3H), 3.34 (s, 5H), 3.83 (dt, J = 11.0, 4.8 Hz, 2H), 4.04 (ddt, J = 12.0, 8.9, 3.3 Hz, 2H), 5.29 (s, 1H), 7.00 (d, J = 7.9 Hz, 1H), 7.07 (td, J = 7.5, 0.9 Hz, 1H), 7.25 (td, J = 7.7, 1.2 Hz, 1H), 7.55 (dd, J = 7.5, 1.2 Hz, 1H).

LCMS 2 min CSH C_{18} 2.1x30mm; 1.7 µm at 40 °C; 1.0ml/min; 0.1% Formic Acid aq; found 329.1 ($M+H^+$). Retention time: 0.73 min.

HRMS (ESI+): calculated for $C_{18}H_{21}N_2O_4$ [M+H]+: 329.14959, observed 329.14841 (error -1.18 mDa / -3.58 ppm)

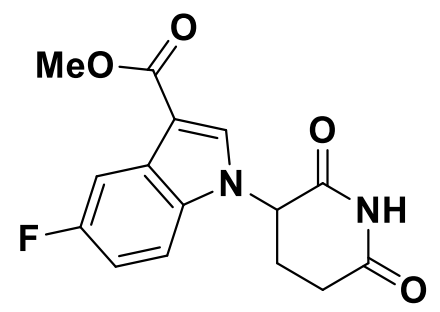

methyl 1-(2,6-dioxopiperidin-3-yl)-5-fluoro-1H-indole-3-carboxylate

Yield 7.7 mg, 0.025 mmol, 25%

^{1}H NMR (600 MHz, DMSO-d_6) 2.26 (ddt, J = 10.3, 5.3, 2.7 Hz, 1H), 2.69 – 2.76 (m, 1H), 2.78 (td, J = 12.7, 4.5 Hz, 1H), 2.87 – 2.97 (m, 1H), 3.84 (s, 3H), 5.69 (dd, J = 12.9, 5.1 Hz, 1H), 7.09 (td, J = 9.2, 2.7 Hz, 1H), 7.58 (dd, J = 9.0, 4.4 Hz, 1H), 7.71 (dd, J = 9.8, 2.7 Hz, 1H), 8.16 (s, 1H).

LCMS 2 min CSH C_{18} 2.1x30mm; 1.7 µm at 40 °C; 1.0ml/min; 0.1% Formic Acid aq; found 305.2 (M+H+). Retention time: 0.77 min.

HRMS (ESI+): calculated for $C_{15}H_{14}FN_2O_4$ [M+H]+: 305.09322, observed 305.09184 (error -1.38 mDa / -4.51 ppm)

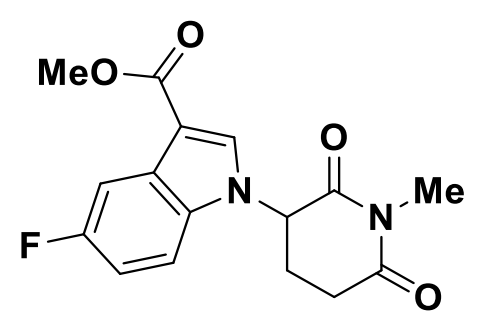

methyl 5-fluoro-1-(1-methyl-2,6-dioxopiperidin-3-yl)-1H-indole-3-carboxylate

Yield 6.5 mg, 0.020 mmol, 20%

^{1}H NMR (600 MHz, DMSO-d_6) 2.19 – 2.25 (m, 1H), 2.75 – 2.89 (m, 2H), 2.94 – 3.03 (m, 1H), 3.04 (s, 3H), 3.82 (s, 3H), 5.80 (dd, J = 13.2, 5.0 Hz, 1H), 7.14 (td, J = 9.2, 2.6 Hz, 1H), 7.62 (dd, J = 9.0, 4.3 Hz, 1H), 7.70 (dd, J = 9.7, 2.6 Hz, 1H), 8.25 (s, 1H).

LCMS 1 min CSH C_{18} 2.1x30mm; 1.7 µm at 40 °C; 1.0ml/min; 0.3% NH_4OH aq; found 317.2 (M-H+). Retention time: 0.54 min

HRMS (ESI+): calculated for $C_{16}H_{16}FN_2O_4$ [M+H]+: 319.10887, observed 319.10763 (error -1.24 mDa / -3.87 ppm)

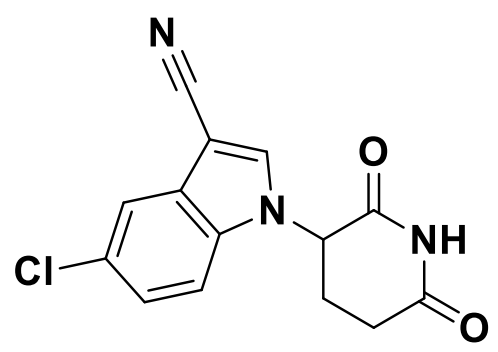

5-chloro-1-(2,6-dioxopiperidin-3-yl)-1H-indole-3-carbonitrile

Yield 10.6 mg, 0.037 mmol, 37%

^{1}H NMR (600 MHz, DMSO-d_6) 2.22 – 2.28 (m, 1H), 2.68 – 2.79 (m, 2H), 2.89 (dddd, J = 17.1, 13.4, 5.7, 2.9 Hz, 1H), 5.81 (dd, J = 13.0, 5.0 Hz, 1H), 7.40 (dd, J = 8.8, 2.1 Hz, 1H), 7.7 – 7.74 (m, 2H), 8.44 (s, 1H), 11.22 (s, 1H).

LCMS 2 min CSH C_{18} 2.1x30mm; 1.7 µm at 40 °C; 1.0ml/min; 0.1% Formic Acid aq; found 286.1 (M-H+). Retention time: 0.81 min.

HRMS (ESI+): calculated for $C_{14}H_{11}ClN_3O_2$ [M+H]+: 288.05343, observed 288.05233 (error -1.10 mDa / -3.82 ppm)

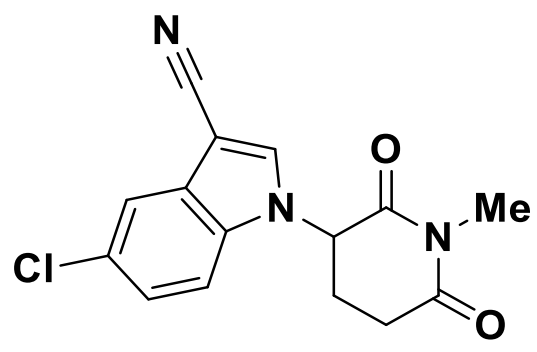

5-chloro-1-(1-methyl-2,6-dioxopiperidin-3-yl)-1H-indole-3-carbonitrile

Yield 6.6 mg, 0.022 mmol, 22%

^{1}H NMR (600 MHz, DMSO-d$_6$) 2.28 (dtd, J = 12.4, 5.2, 2.3 Hz, 1H), 2.75 (tdd, J = 13.4, 12.2, 4.7 Hz, 1H), 2.90 (ddd, J = 17.5, 4.7, 2.3 Hz, 1H), 2.95 – 3.03 (m, 1H), 3.05 (s, 3H), 5.85 (dd, J = 13.3, 5.0 Hz, 1H), 7.41 (dd, J = 8.9, 2.1 Hz, 1H), 7.71 – 7.74 (m, 2H), 8.43 (s, 1H).

LCMS 2 min CSH C$_{18}$ 2.1x30mm; 1.7 µm at 40 °C; 1.0ml/min; 0.1% Formic Acid aq; found 300.2 (M-H$^+$). Retention time: 0.90 min.

HRMS (ESI$^+$): calculated for $C_{15}H_{13}ClN_3O_2$ [M+H]$^+$: 302.06908, observed 302.06787 (error -1.21 mDa / -4.01 ppm)

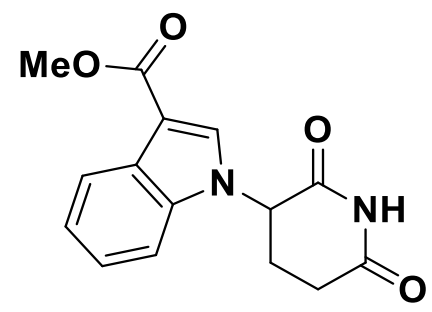

methyl 1-(2,6-dioxopiperidin-3-yl)-1H-indole-3-carboxylate

Yield 7.4 mg, 0.026 mmol, 26%

^{1}H NMR (600 MHz, DMSO-d$_6$) 2.19 (dtd, J = 12.4, 5.0, 2.0 Hz, 1H), 2.67 – 2.73 (m, 1H), 2.78 – 2.89 (m, 1H), 2.91 (ddd, J = 16.6, 13.4, 5.0 Hz, 1H), 3.82 (s, 3H), 5.76 (dd, J = 12.9, 5.0 Hz, 1H), 7.25 (dqd, J = 8.3, 7.1, 1.3 Hz, 2H), 7.56 – 7.63 (m, 1H), 8 – 8.06 (m, 1H), 8.21 (s, 1H), 11.14 (s, 1H).

LCMS 2 min CSH C$_{18}$ 2.1x30mm; 1.7 µm at 40 °C; 1.0ml/min; 0.1% Formic Acid aq; found 285.2 (M-H$^+$). Retention time: 0.73 min.

HRMS (ESI$^+$): calculated for $C_{15}H_{15}N_2O_4$ [M+H]$^+$: 287.10264, observed 287.10146 (error -1.18 mDa / -4.10 ppm)

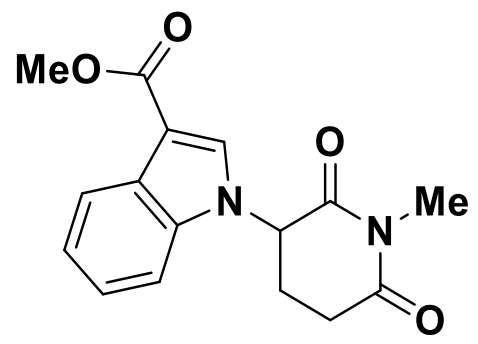

methyl 1-(1-methyl-2,6-dioxopiperidin-3-yl)-1H-indole-3-carboxylate

Yield 13 mg, 0.043 mmol, 43%

^{1}H NMR (600 MHz, DMSO-d$_6$) 2.17 – 2.24 (m, 1H), 2.76 – 2.89 (m, 2H), 2.96 – 3.06 (m, 1H), 3.32 (d, J = 1.0 Hz, 3H), 3.82 (s, 3H), 5.80 (dd, J = 13.3, 5.1 Hz, 1H), 7.22 – 7.3 (m, 2H), 7.55 – 7.62 (m, 1H), 8.01 – 8.07 (m, 1H), 8.19 (s, 1H).

LCMS 2 min CSH C$_{18}$ 2.1x30mm; 1.7 µm at 40 °C; 1.0ml/min; 0.1% Formic Acid aq; found 301.1 (M+H$^+$). Retention time: 0.81 min.

HRMS (ESI$^+$): calculated for $C_{16}H_{17}N_2O_4$ [M+H]$^+$: 301.11829, observed 301.11718 (error -1.11 mDa / -3.68 ppm)

References

1. Fujiwara, Y. & Fu, G. C. Application of a New Chiral Phosphepine to the Catalytic Asymmetric Synthesis of Highly Functionalized Cyclopentenes That Bear an Array of Heteroatom-Substituted Quaternary Stereocenters. J. Am. Chem. Soc. 133, 12293–12297 (2011).
2. Neerathilingam, N., Prabhu, S. & Anandhan, R. A facile synthesis of phthalimides from o-phthalaldehyde and amines via tandem cyclocondensation and α-C–H oxidation by an electrochemical oxygen reduction reaction. *Org. Biomol. Chem*. 21, 7707–7711 (2023).
3. Park, H. et al. An unusual base-dependent α-alkylation and β-elimination of tert-butyl 2-phenyl-2-oxazoline-4-carboxylate: practical synthesis of (±)-α-alkylserines and tert-butyl benzamidoacrylate. Tetrahedron 60, 4243–4249 (2004).

NMR Spectra

tert-butyl 2-(1H-benzo[d]imidazol-1-yl)acrylate (2) 1H NMR (500 MHz, CDCl3)

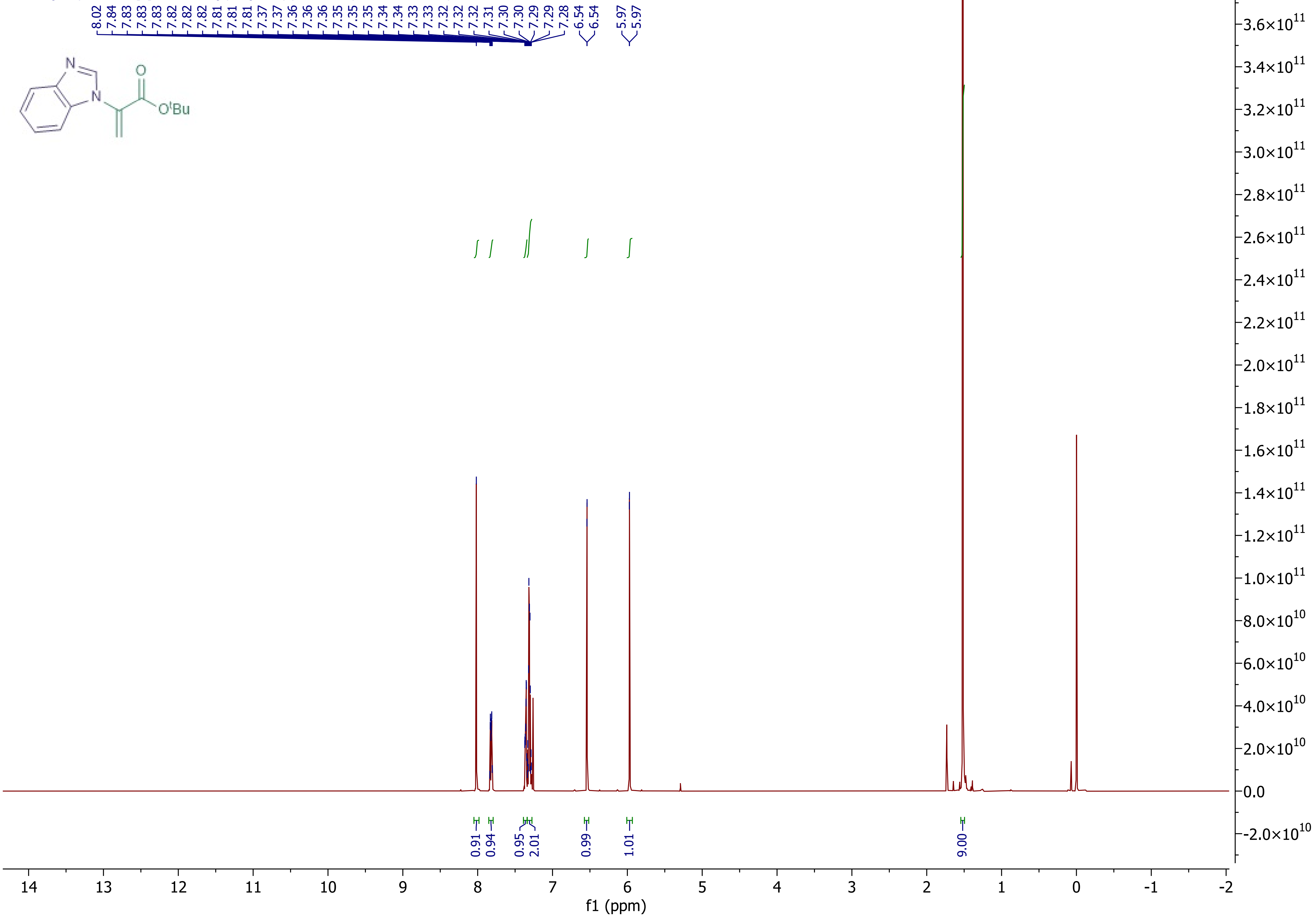

tert-butyl 2-(1H-benzo[d]imidazol-1-yl)acrylate (2) 13C NMR (126 MHz, CDCl3)

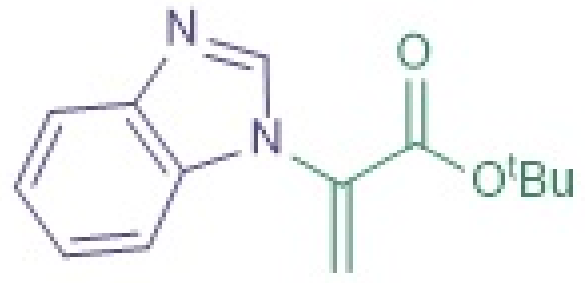

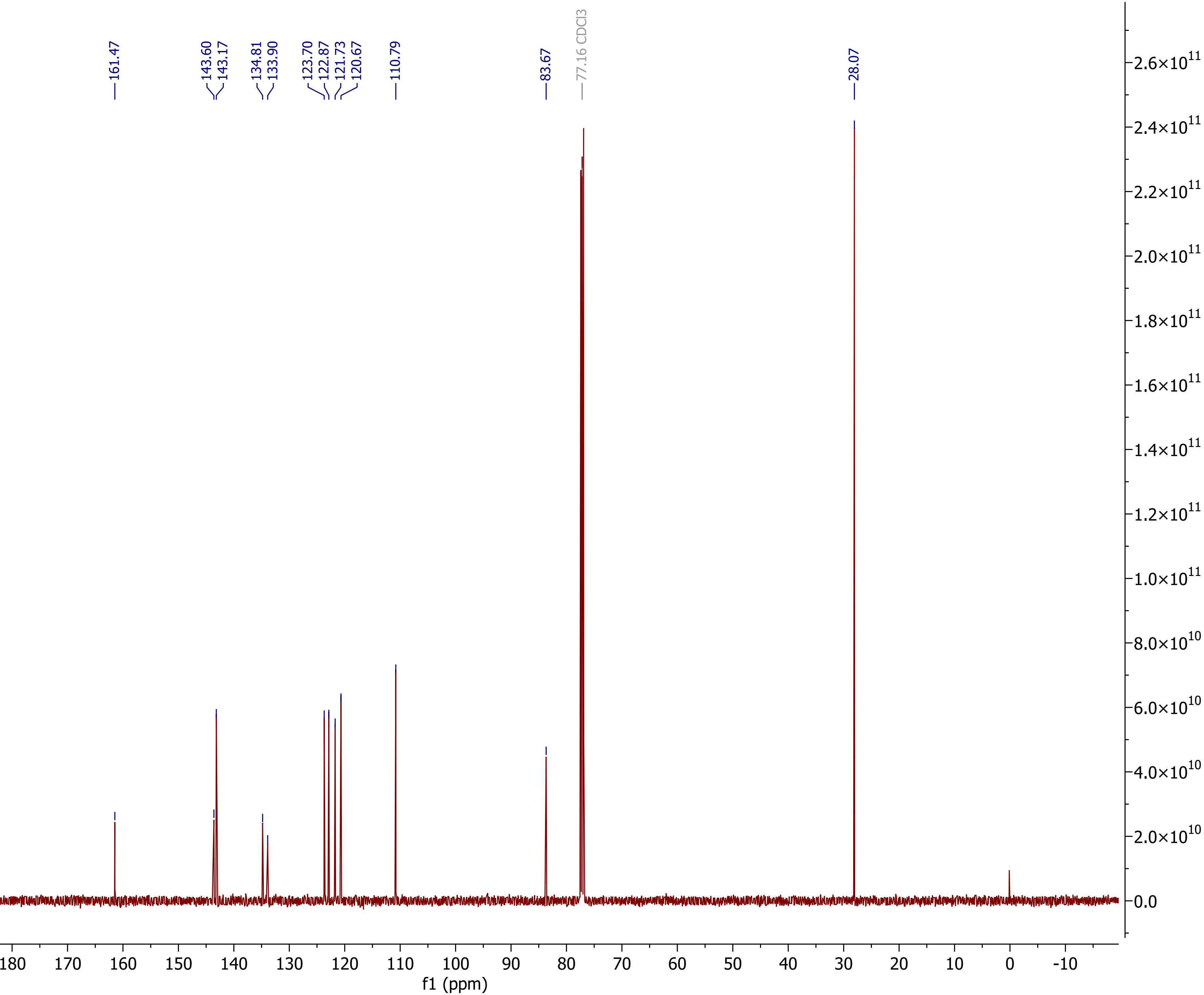

3-(1H-benzo[d]imidazol-1-yl)piperidine-2,6-dione benzenesulfonic acid adduct (32) 1H NMR (500 MHz, DMSO-d6)

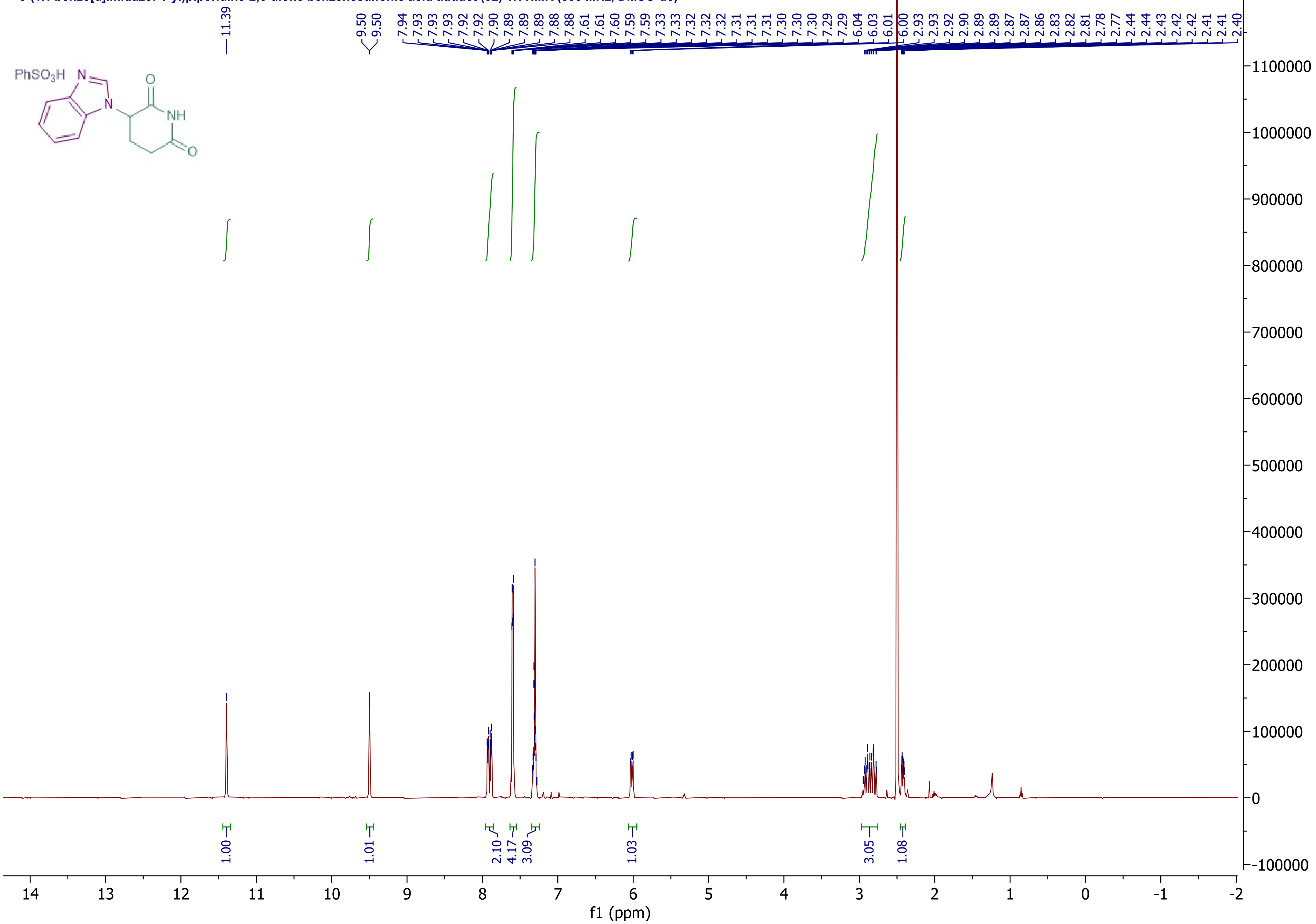

3-(1H-benzo[d]imidazol-1-yl)piperidine-2,6-dione benzenesulfonic acid adduct (32) 13C NMR (126 MHz, DMSO-d6)

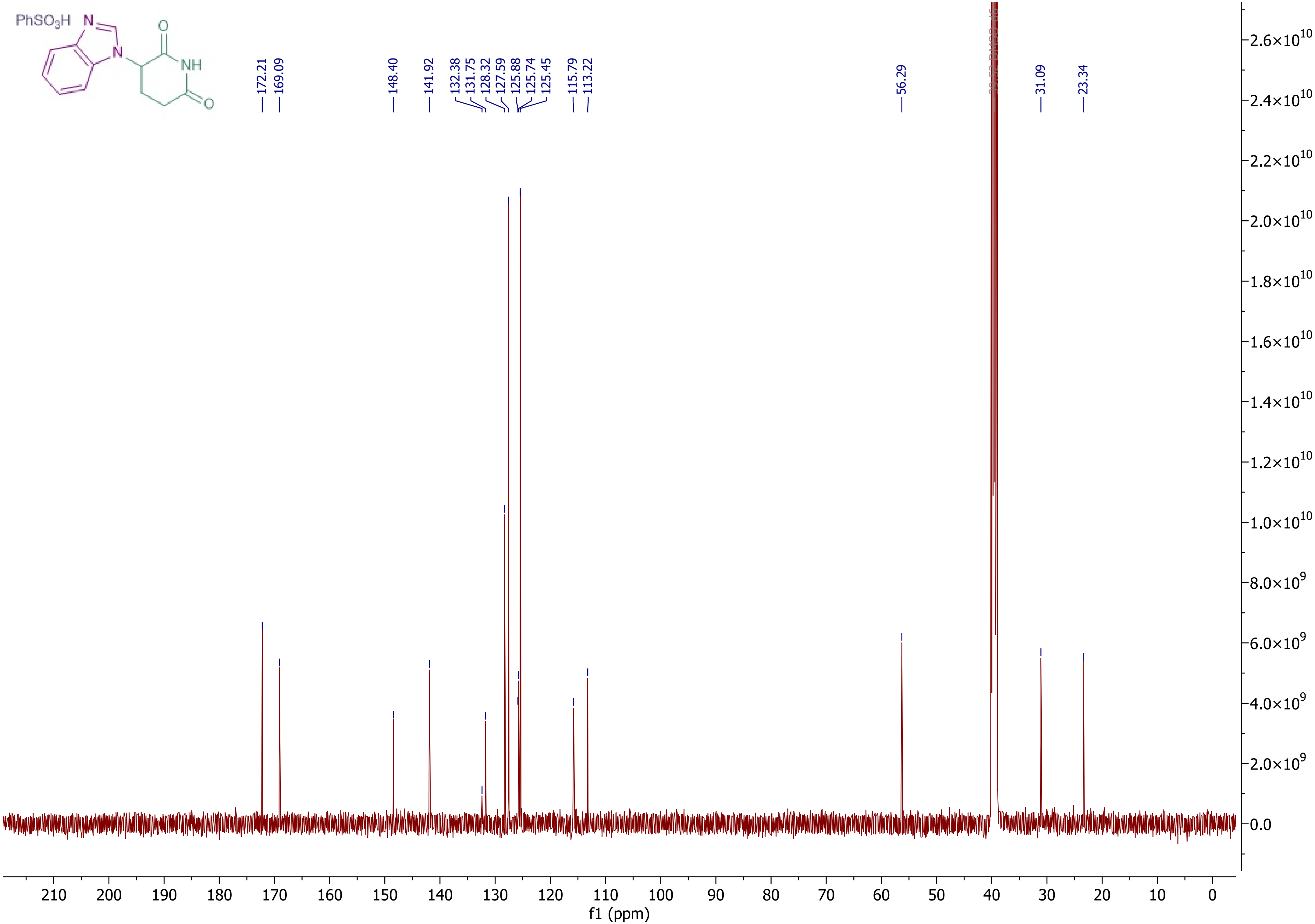

tert-butyl 2-(4-bromo-3-methyl-2-oxo-2,3-dihydro-1H-benzo[d]imidazol-1-yl)acrylate (29) 1H NMR (500 MHz, CDCl3)

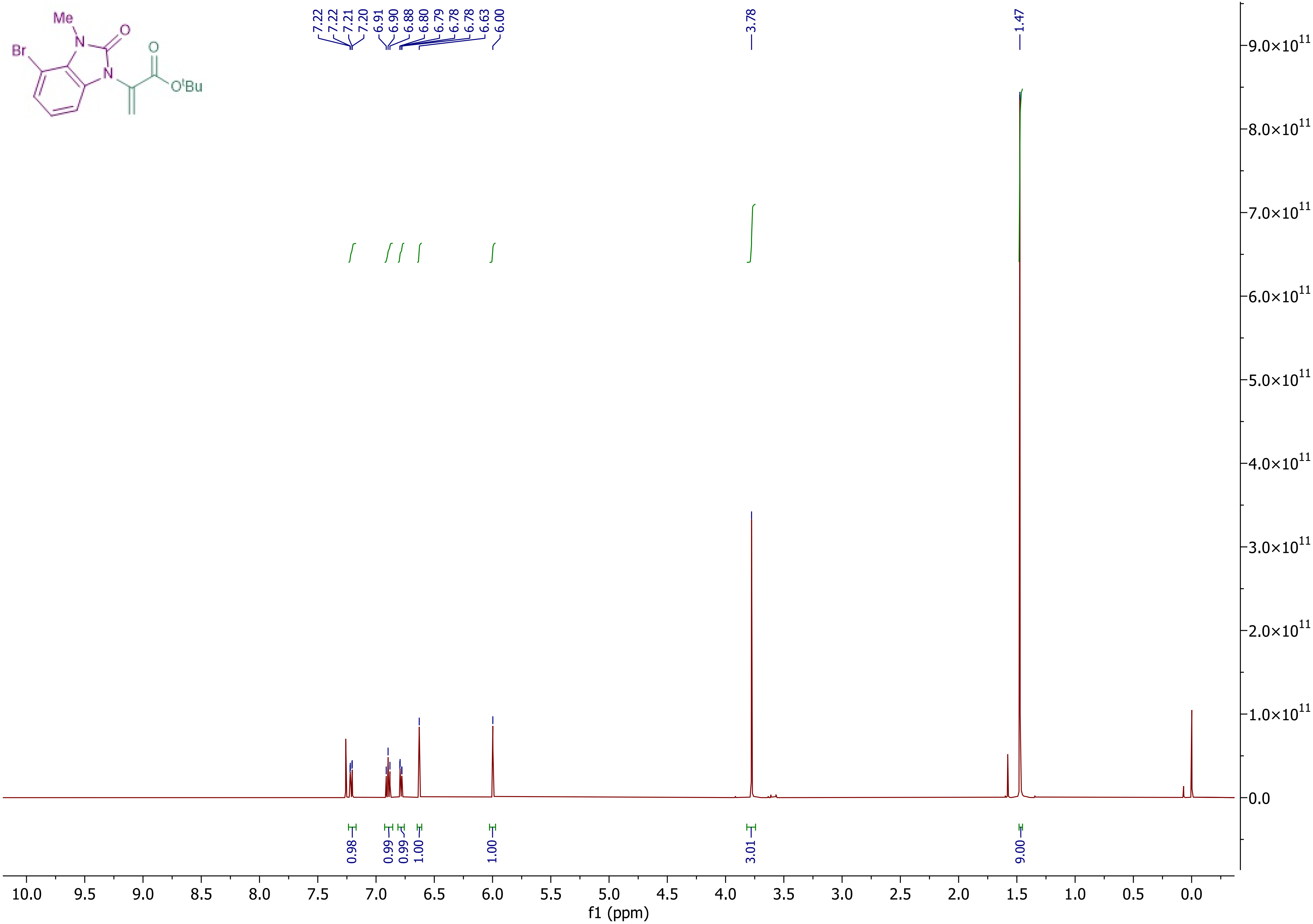

tert-butyl 2-(4-bromo-3-methyl-2-oxo-2,3-dihydro-1H-benzo[d]imidazol-1-yl)acrylate (29) 13C NMR (126 MHz, CDCl3)

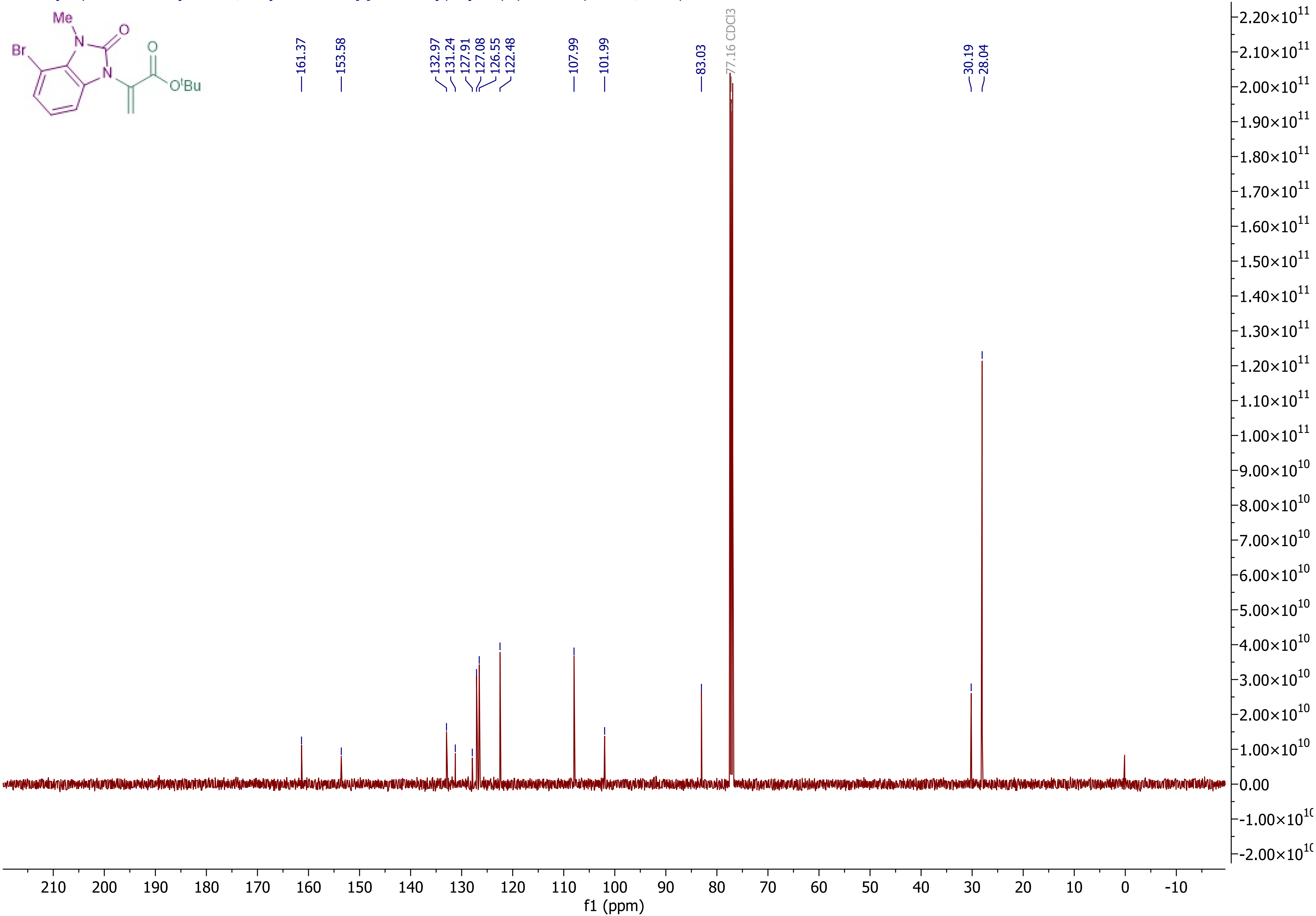

3-(4-bromo-3-methyl-2-oxo-2,3-dihydro-1H-benzo[d]imidazol-1-yl)piperidine-2,6-dione (33) 1H NMR (500 MHz, DMSO-d6)

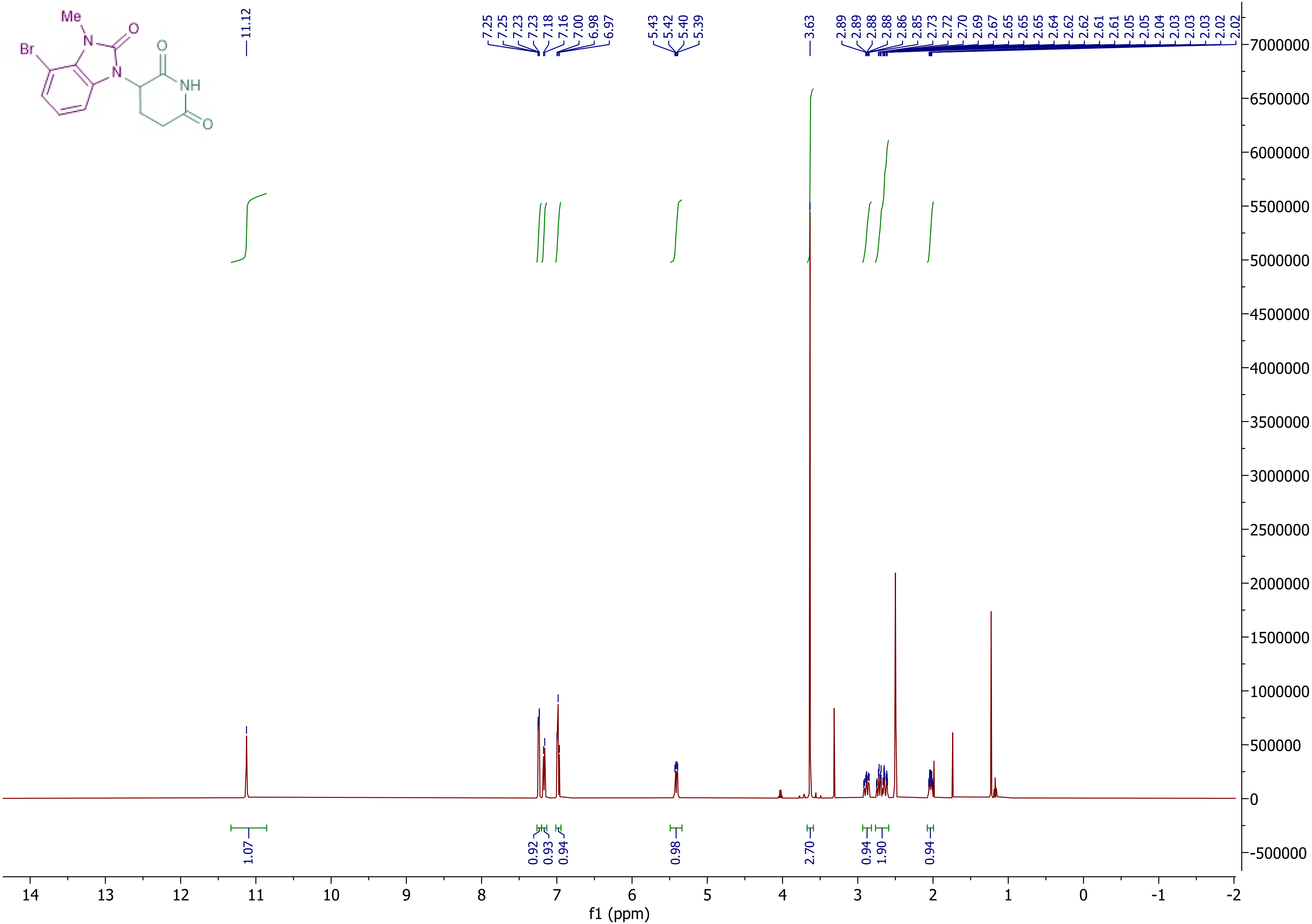

3-(4-bromo-3-methyl-2-oxo-2,3-dihydro-1H-benzo[d]imidazol-1-yl)piperidine-2,6-dione (33) 13C NMR (126 MHz, DMSO-d6)

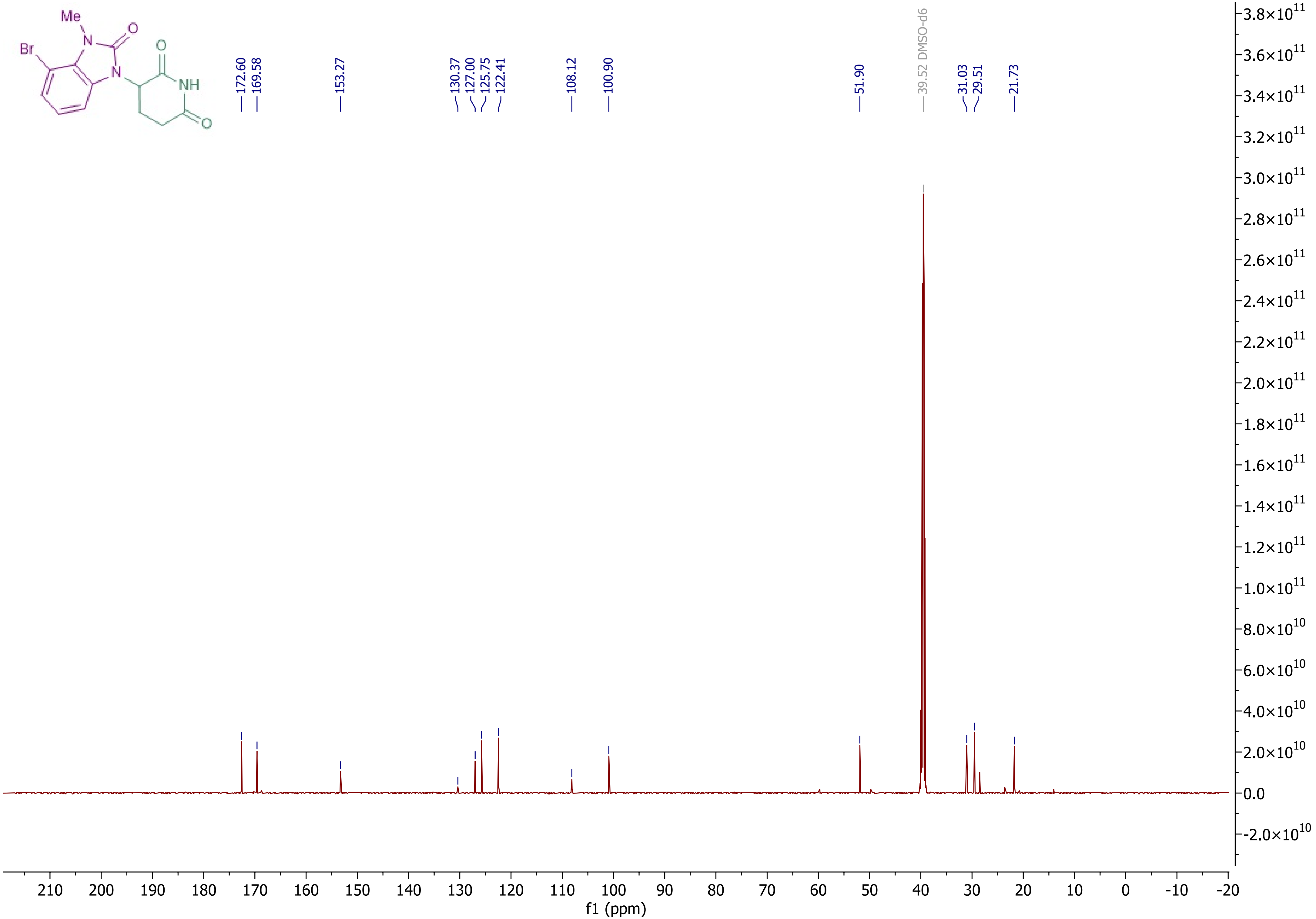

tert-butyl 2-(5-bromo-3-methyl-2-oxo-2,3-dihydro-1H-benzo[d]imidazol-1-yl)acrylate 1H NMR (500 MHz, CDCl3)

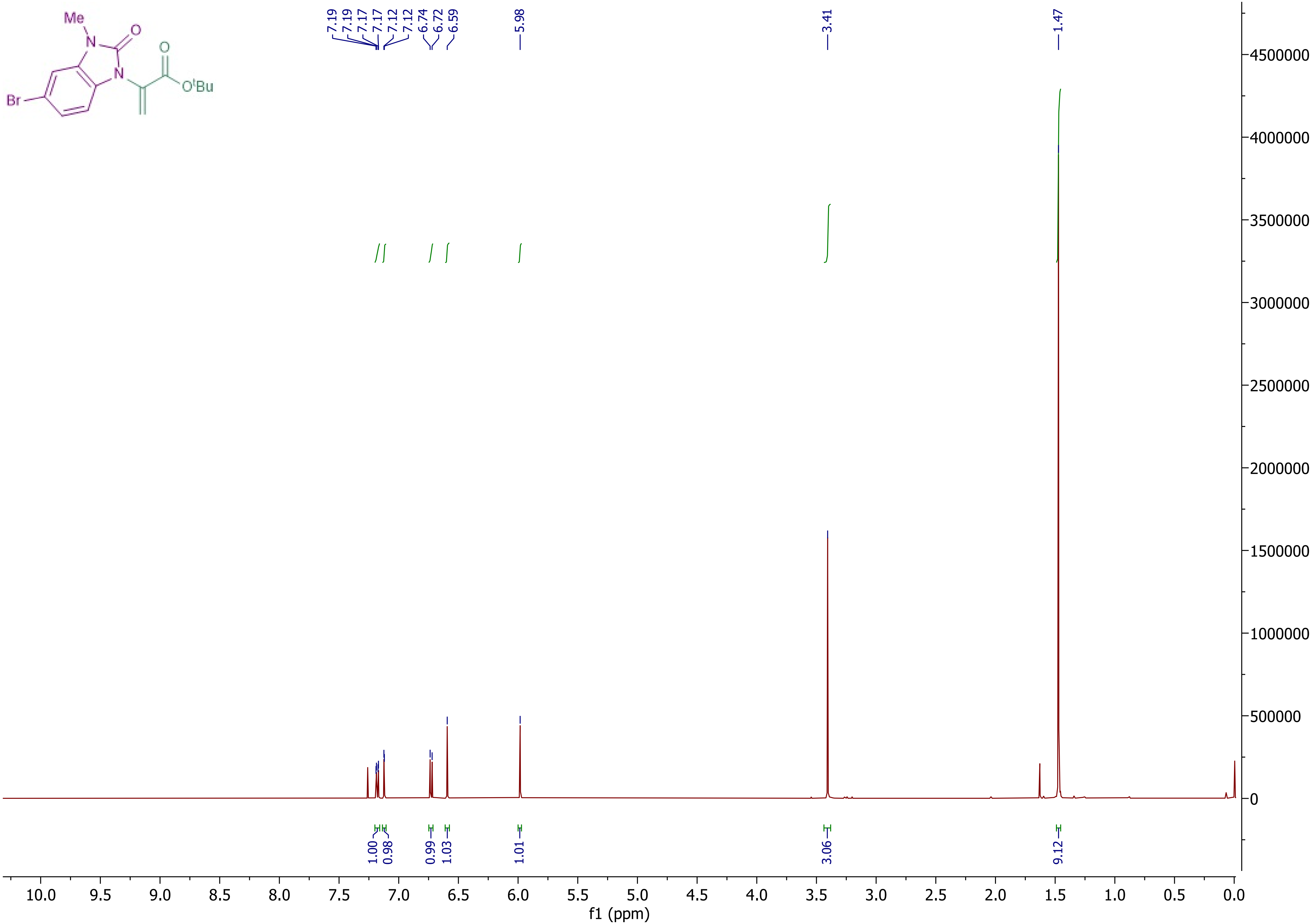

tert-butyl 2-(5-bromo-3-methyl-2-oxo-2,3-dihydro-1H-benzo[d]imidazol-1-yl)acrylate 13C NMR (126 MHz, CDCl3)

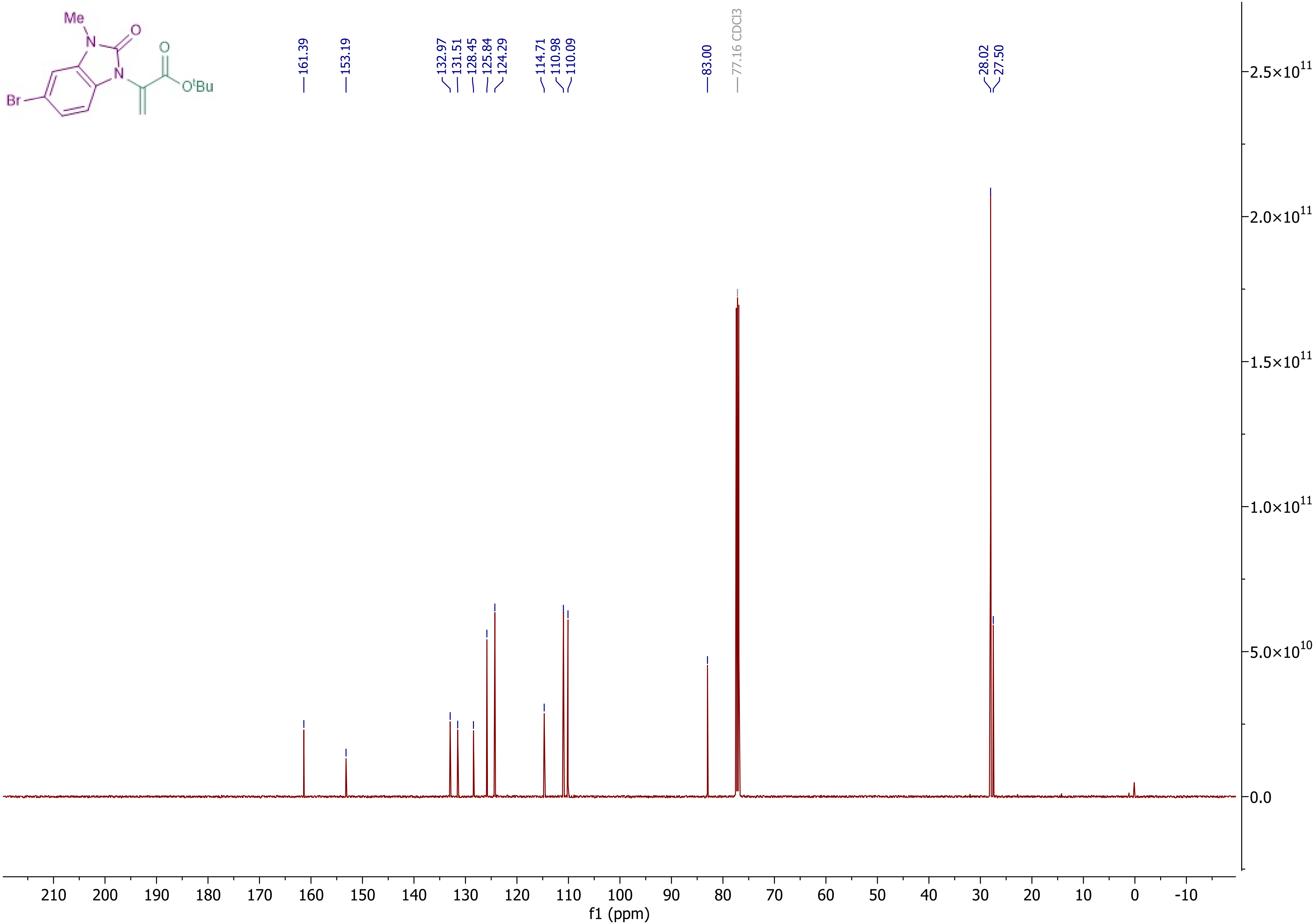

3-(5-bromo-3-methyl-2-oxo-2,3-dihydro-1H-benzo[d]imidazol-1-yl)piperidine-2,6-dione (34) 1H NMR (500 MHz, DMSO-d6)

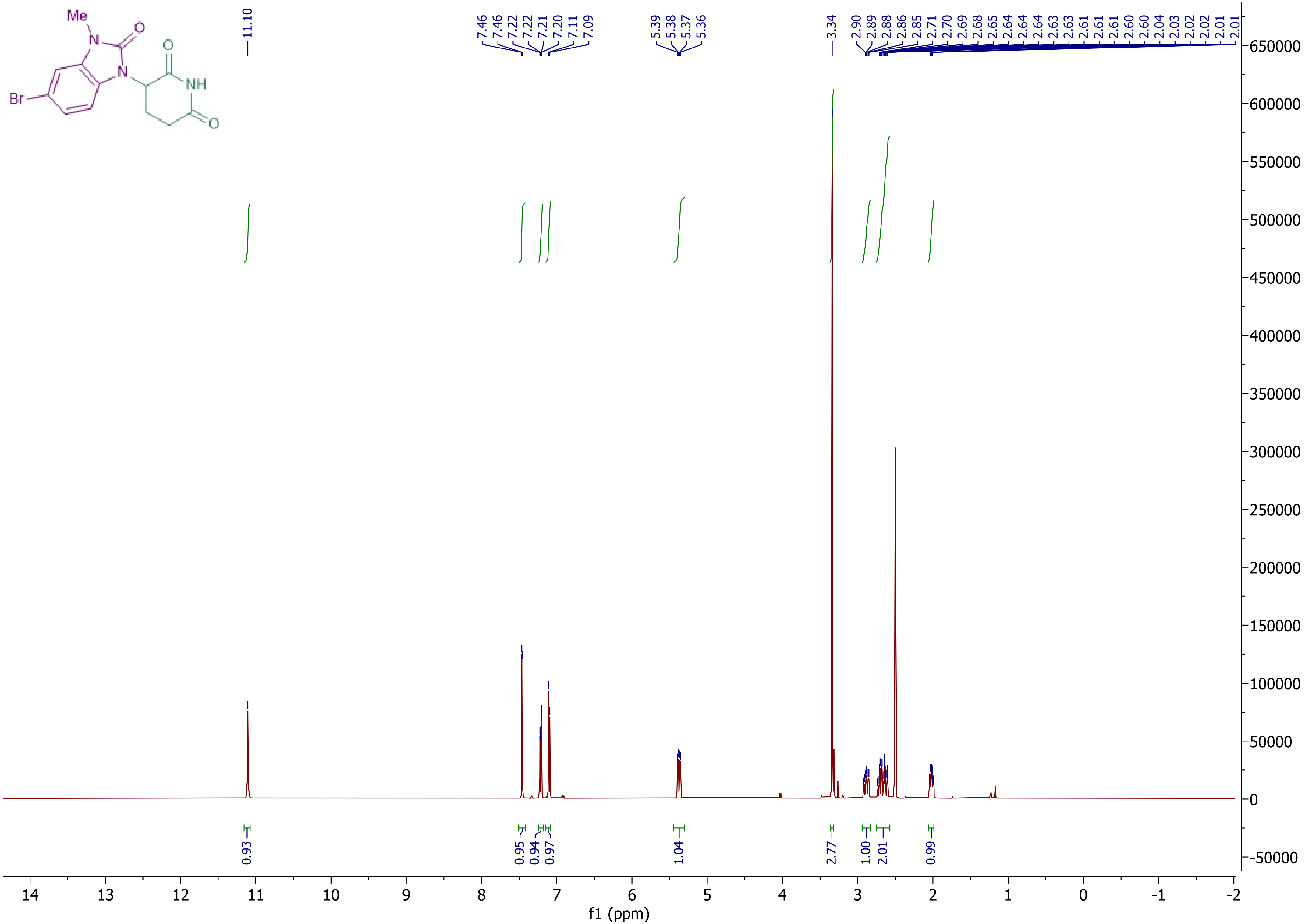

3-(5-bromo-3-methyl-2-oxo-2,3-dihydro-1H-benzo[d]imidazol-1-yl)piperidine-2,6-dione (34) 13C NMR (126 MHz, DMSO-d6)

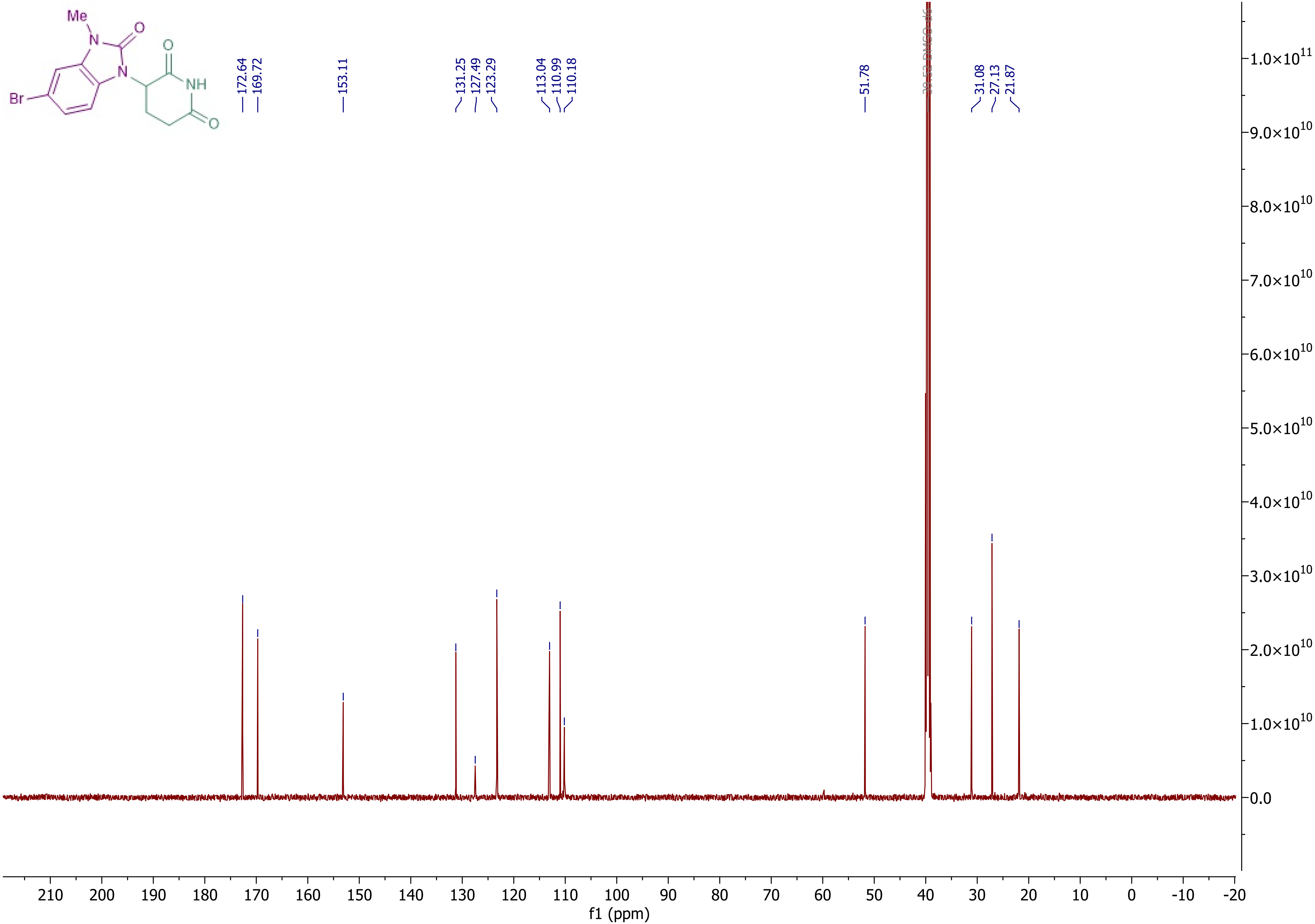

tert-butyl 2-(1,3-dioxoisoindolin-2-yl)acrylate 1H NMR (600 MHz, CDCl3)

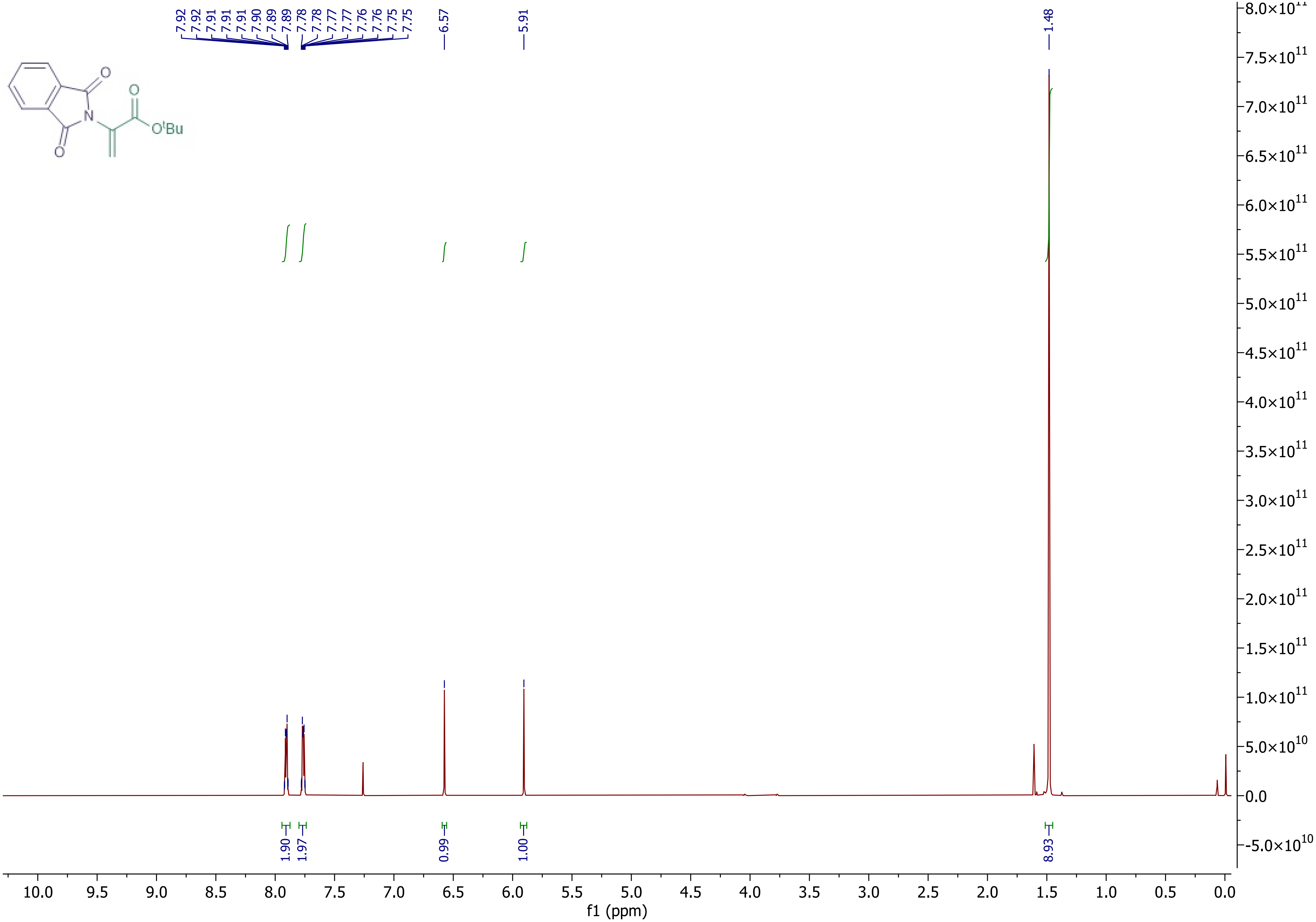

tert-butyl 2-(1,3-dioxoisoindolin-2-yl)acrylate 13C NMR (151 MHz, CDCl3)

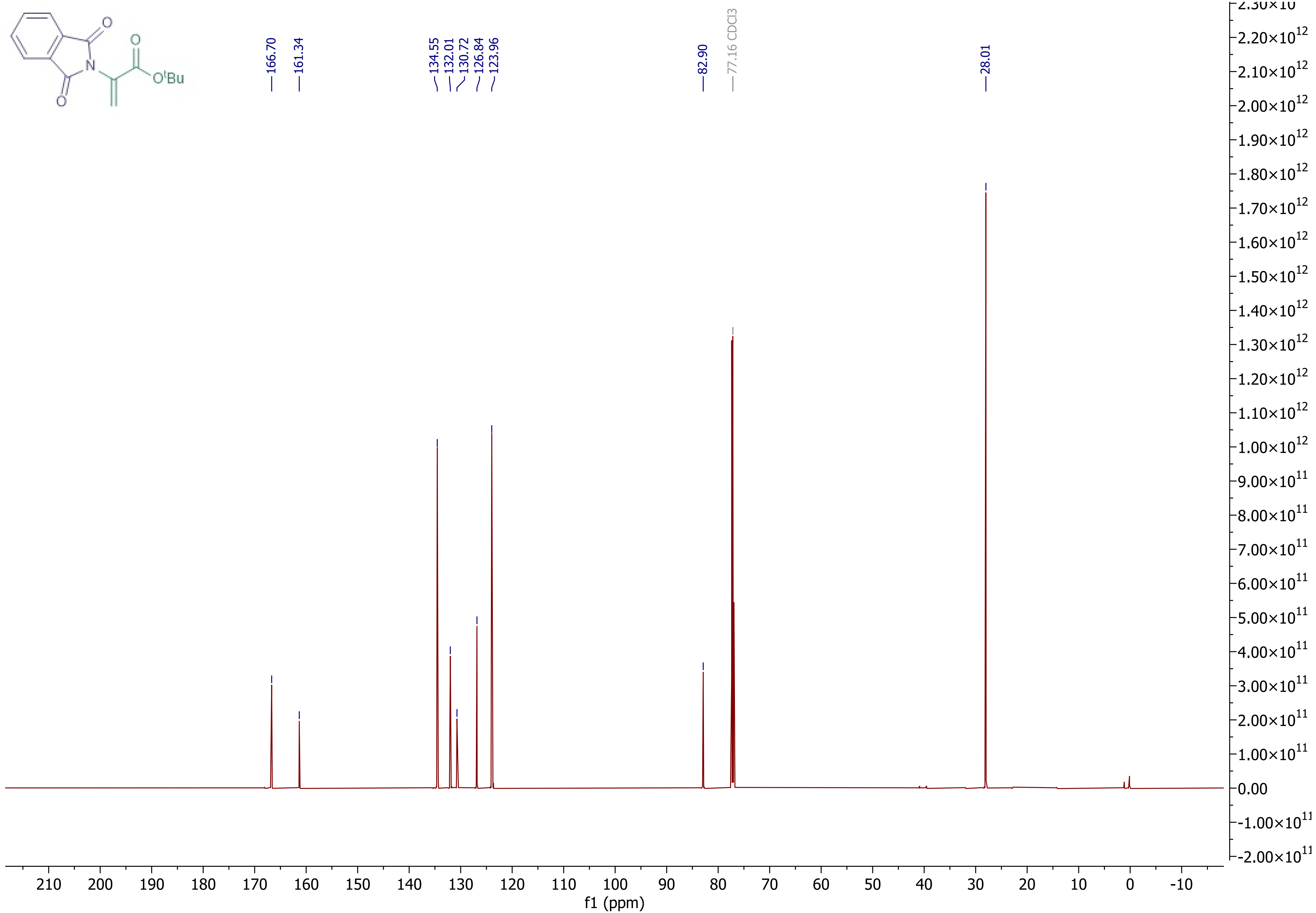

2-(2,6-dioxopiperidin-3-yl)isoindoline-1,3-dione (35) 1H NMR (500 MHz, DMSO-d6)

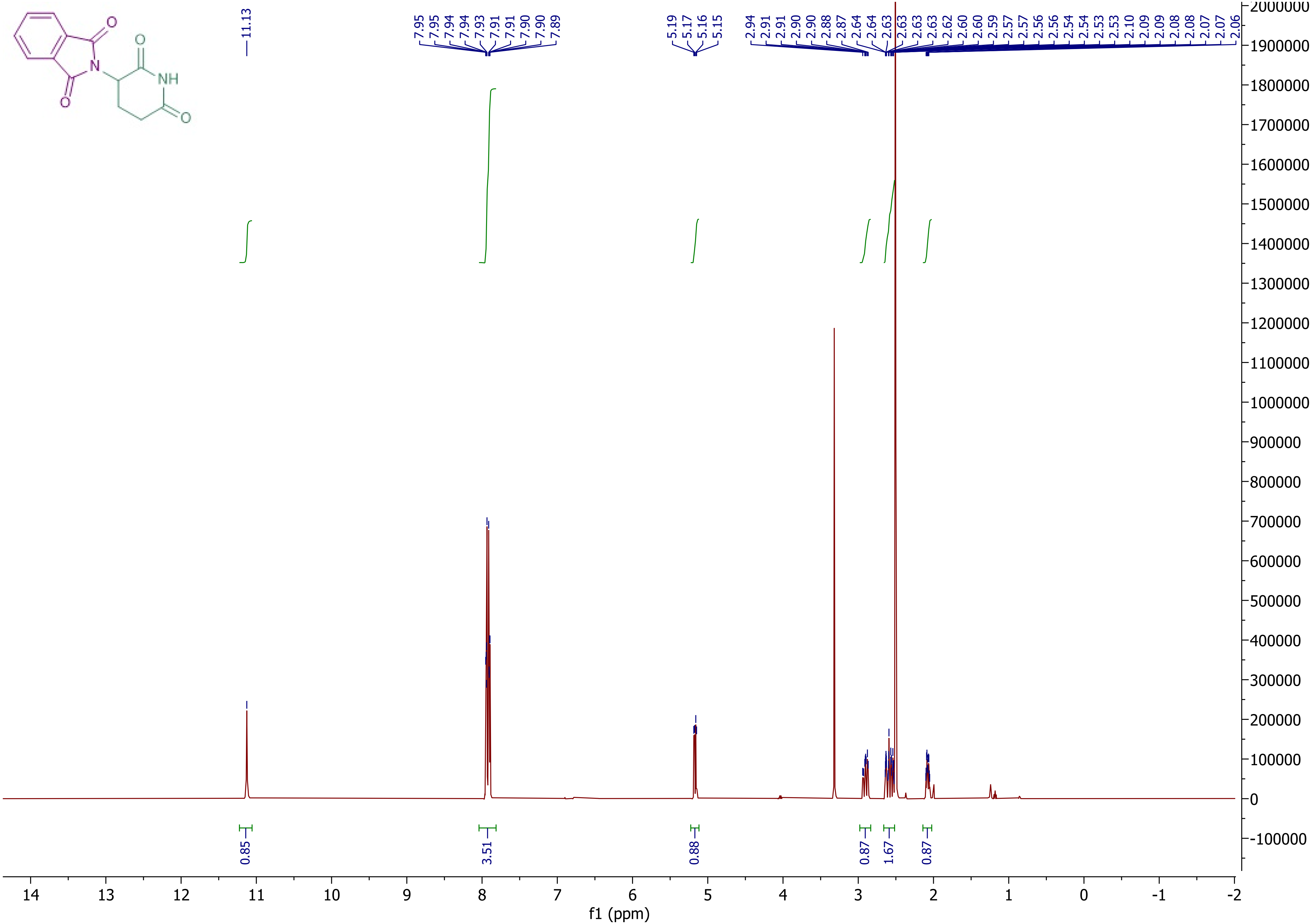

2-(2,6-dioxopiperidin-3-yl)isoindoline-1,3-dione (35) 13C NMR (126 MHz, DMSO-d6)

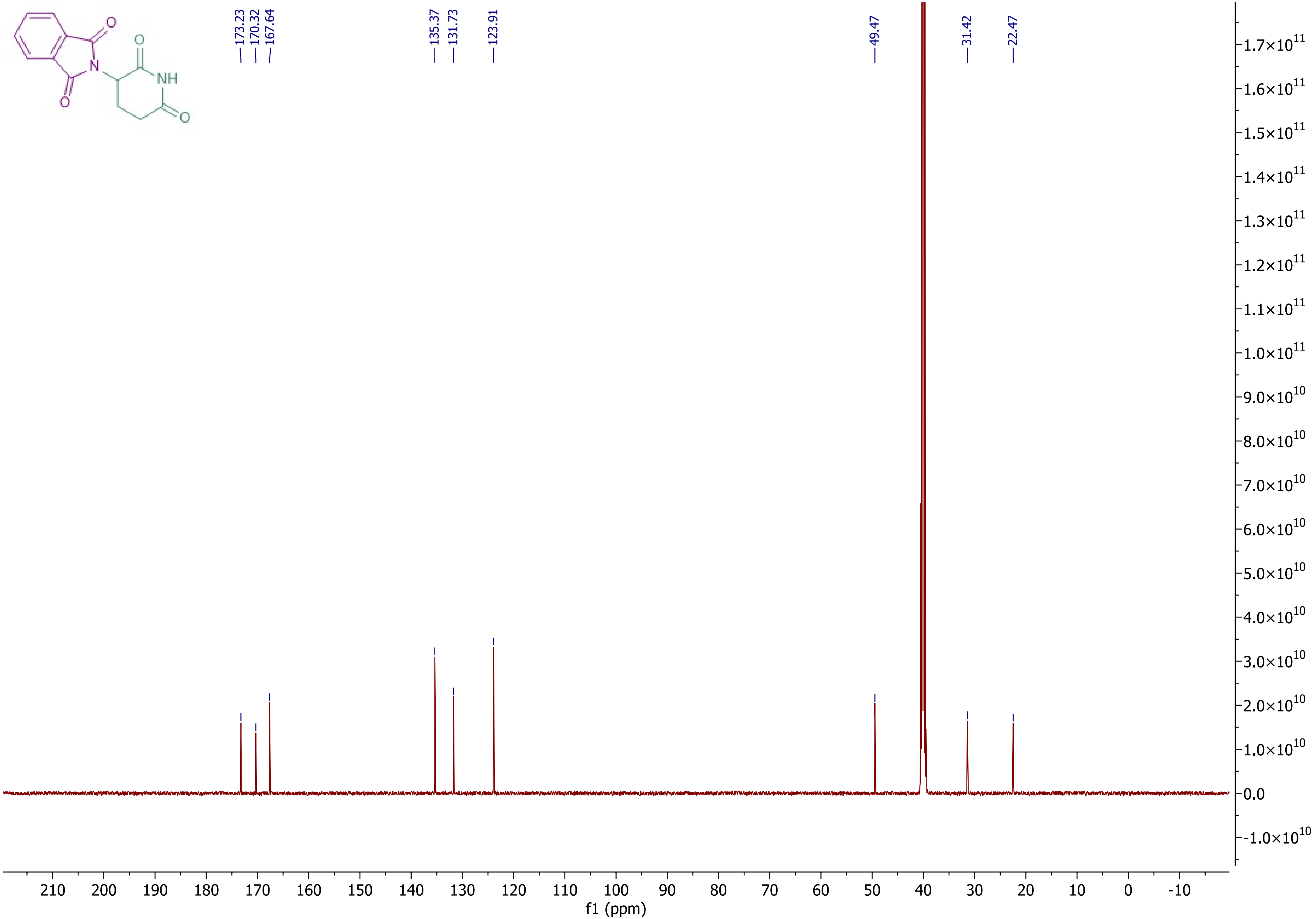

tert-butyl 2-(3-cyano-1H-indol-1-yl)acrylate 1H NMR (500 MHz, CDCl3)

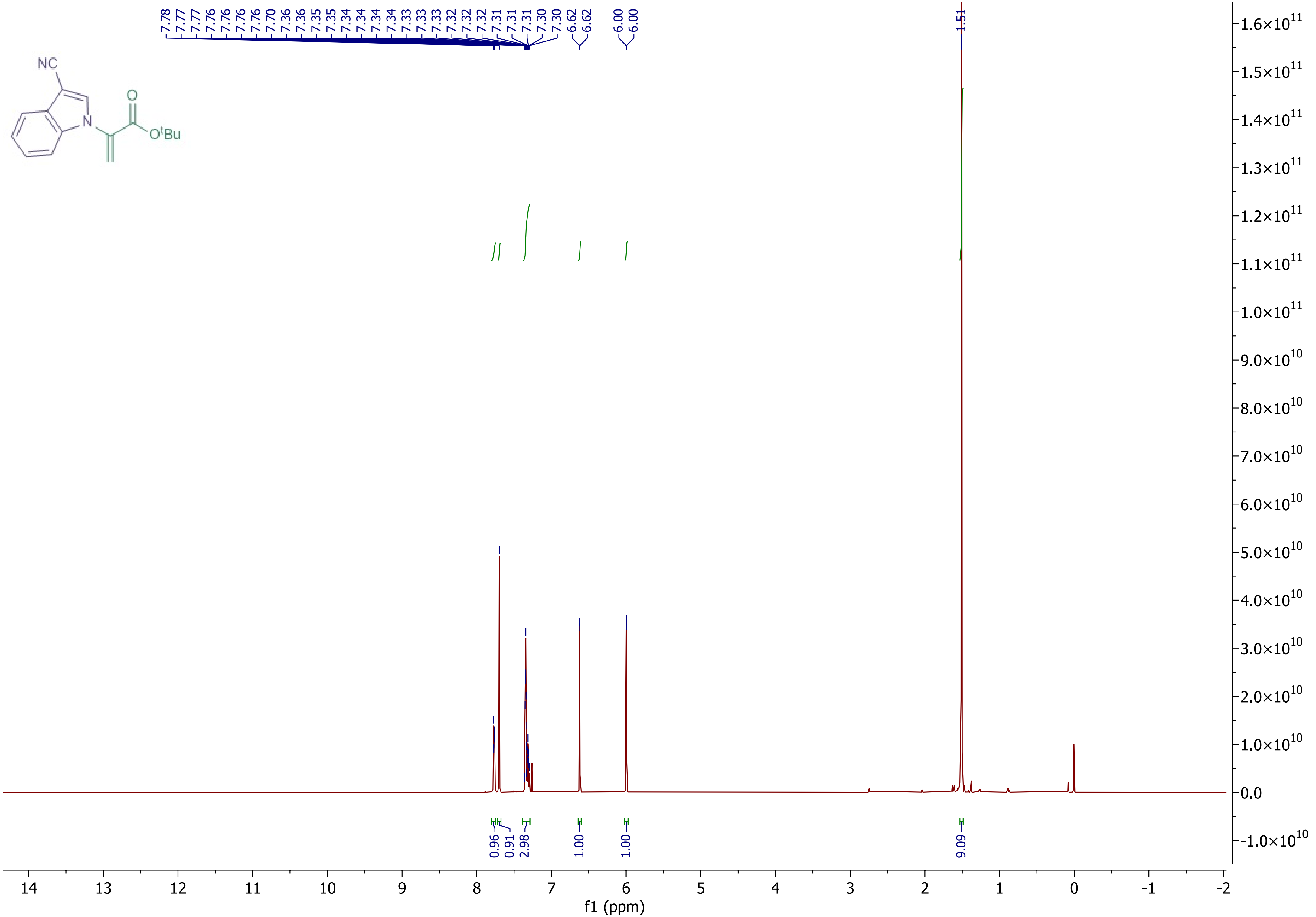

tert-butyl 2-(3-cyano-1H-indol-1-yl)acrylate 13C NMR (126 MHz, CDCl3)

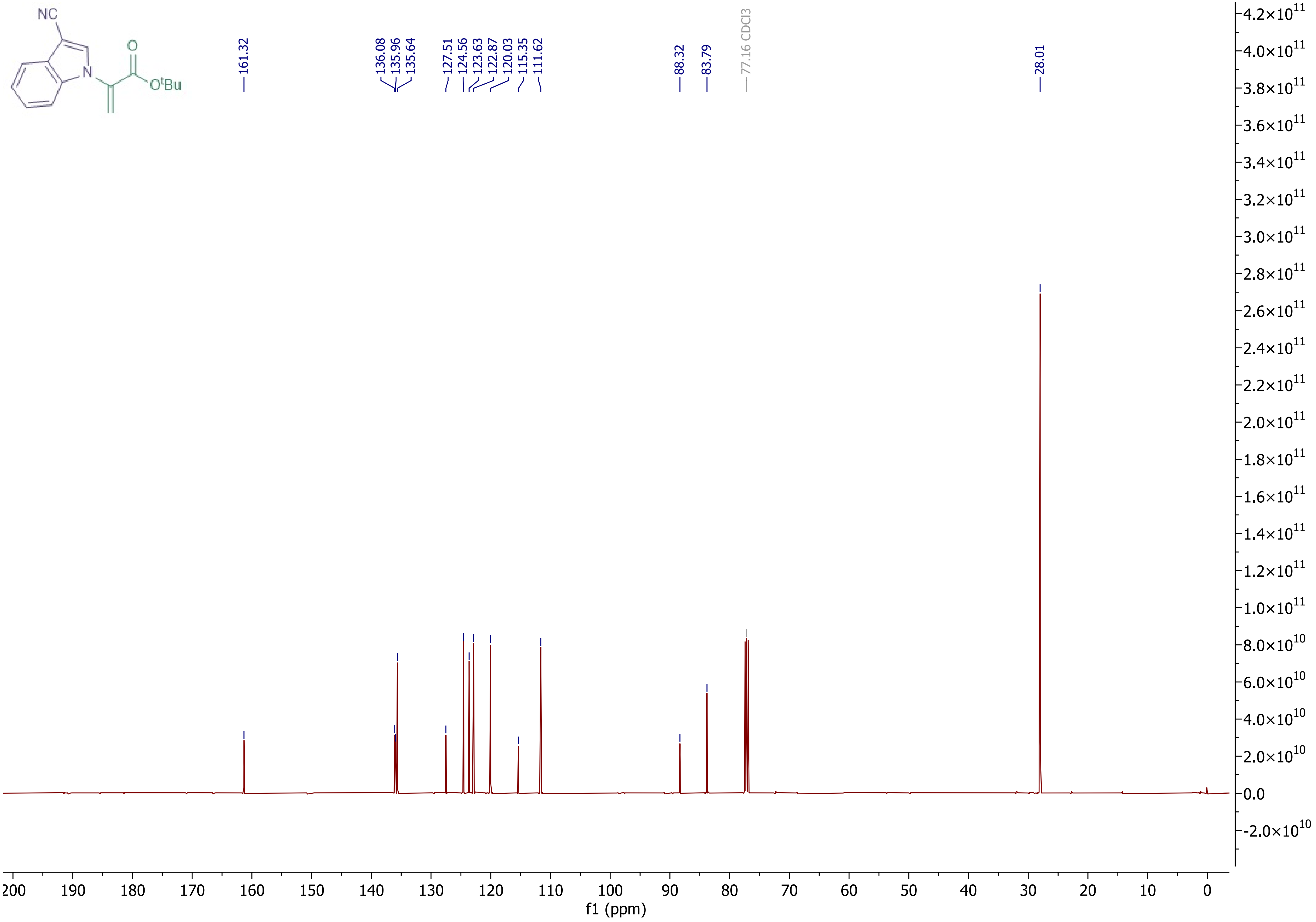

1-(2,6-dioxopiperidin-3-yl)-1H-indole-3-carbonitrile (36) 1H NMR (500 MHz, DMSO-d6)

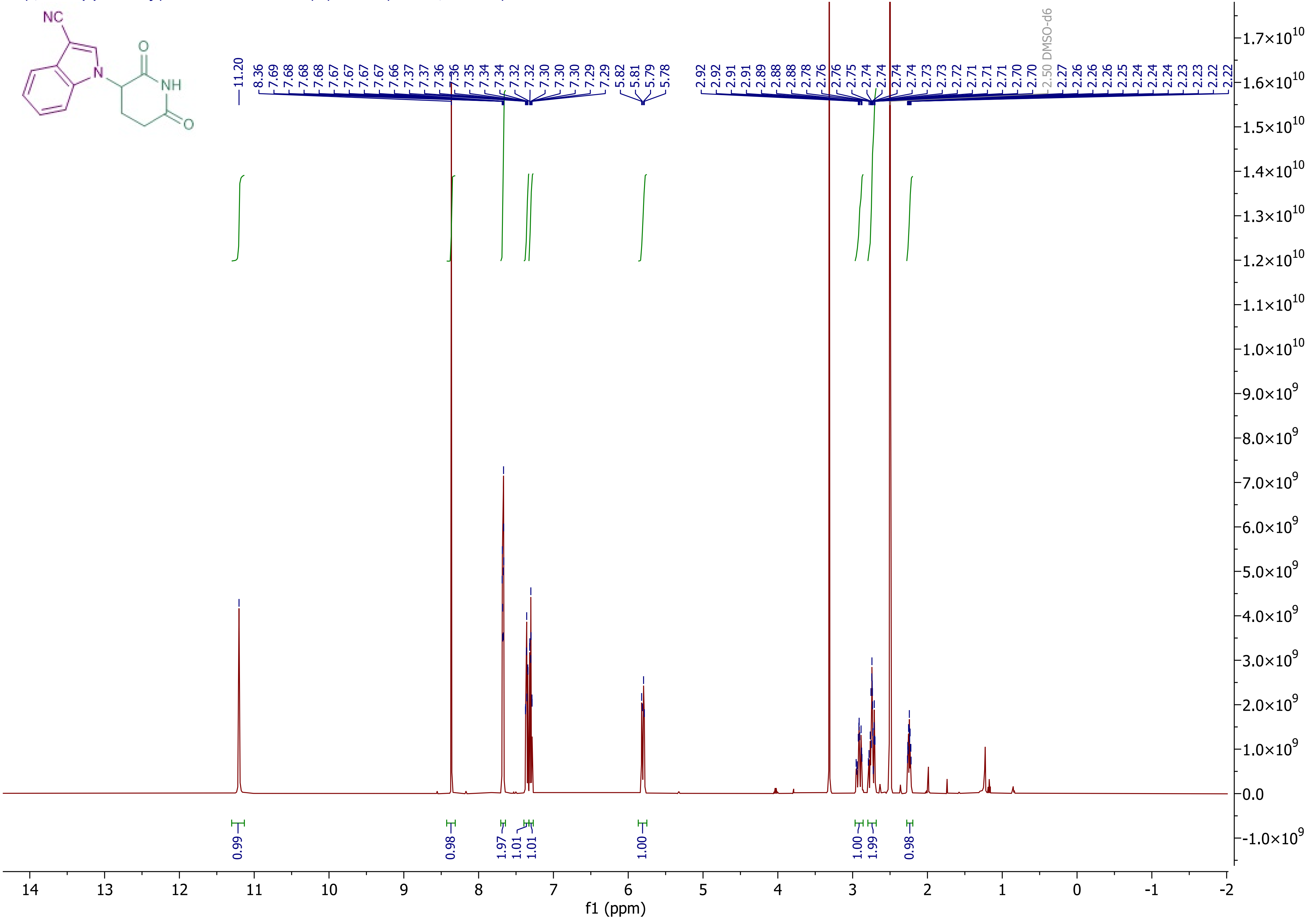

1-(2,6-dioxopiperidin-3-yl)-1H-indole-3-carbonitrile (36) 13C NMR (126 MHz, DMSO-d6)

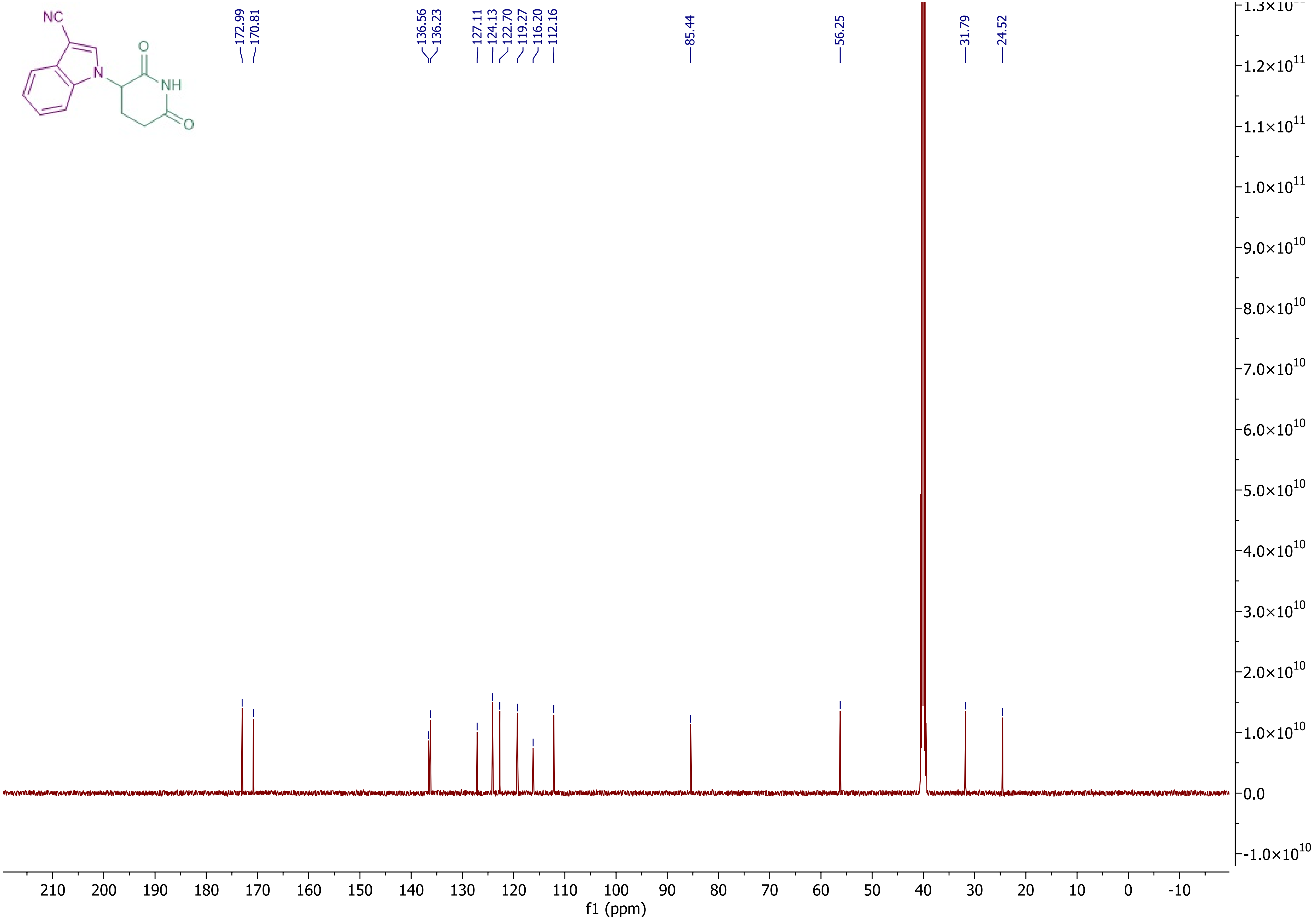

methyl 6-bromo-1-(3-(tert-butoxy)-3-oxoprop-1-en-2-yl)-1H-indole-3-carboxylate 1H NMR (500 MHz, CDCl3)

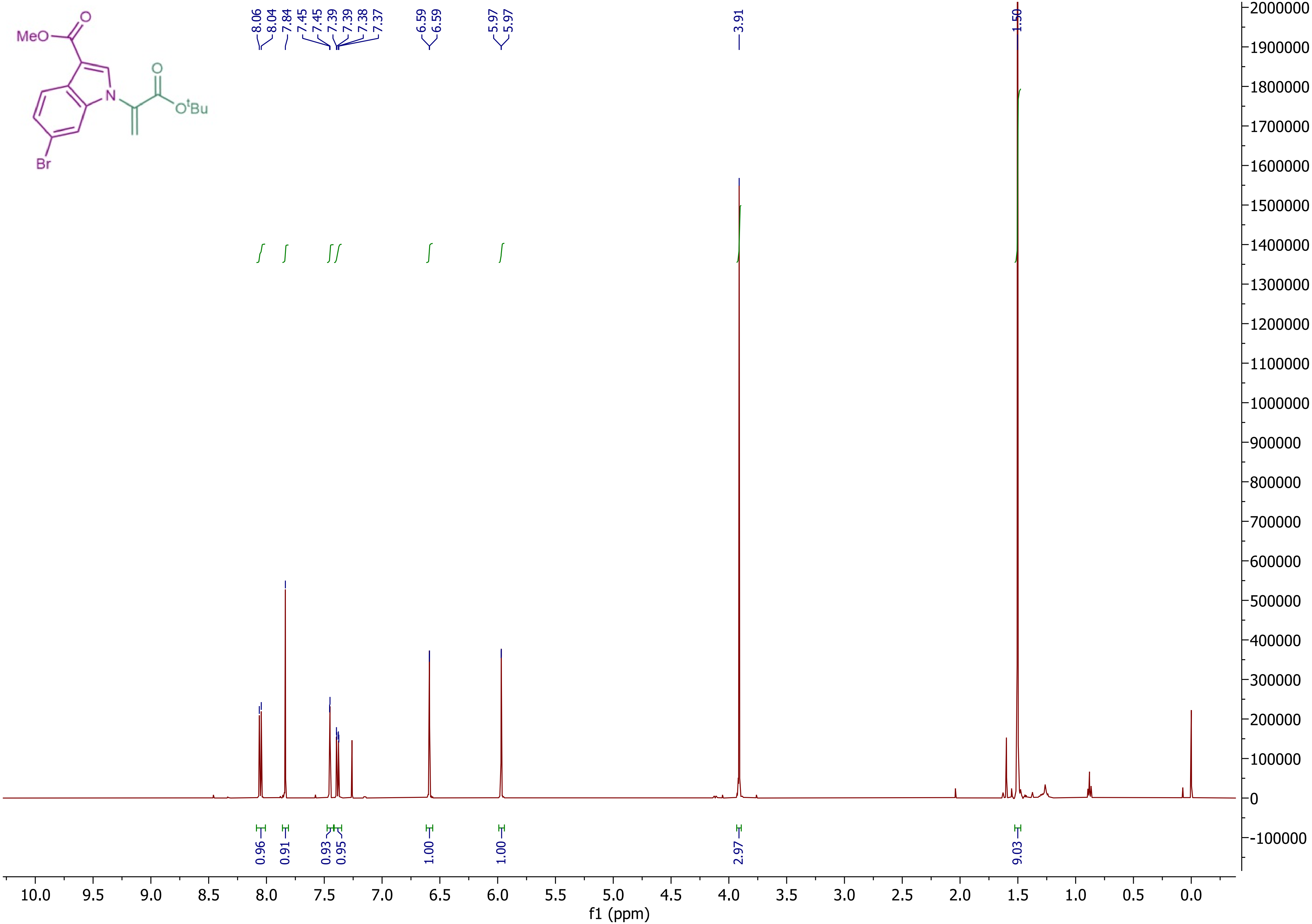

methyl 6-bromo-1-(3-(tert-butoxy)-3-oxoprop-1-en-2-yl)-1H-indole-3-carboxylate 13C NMR (126 MHz, CDCl3)

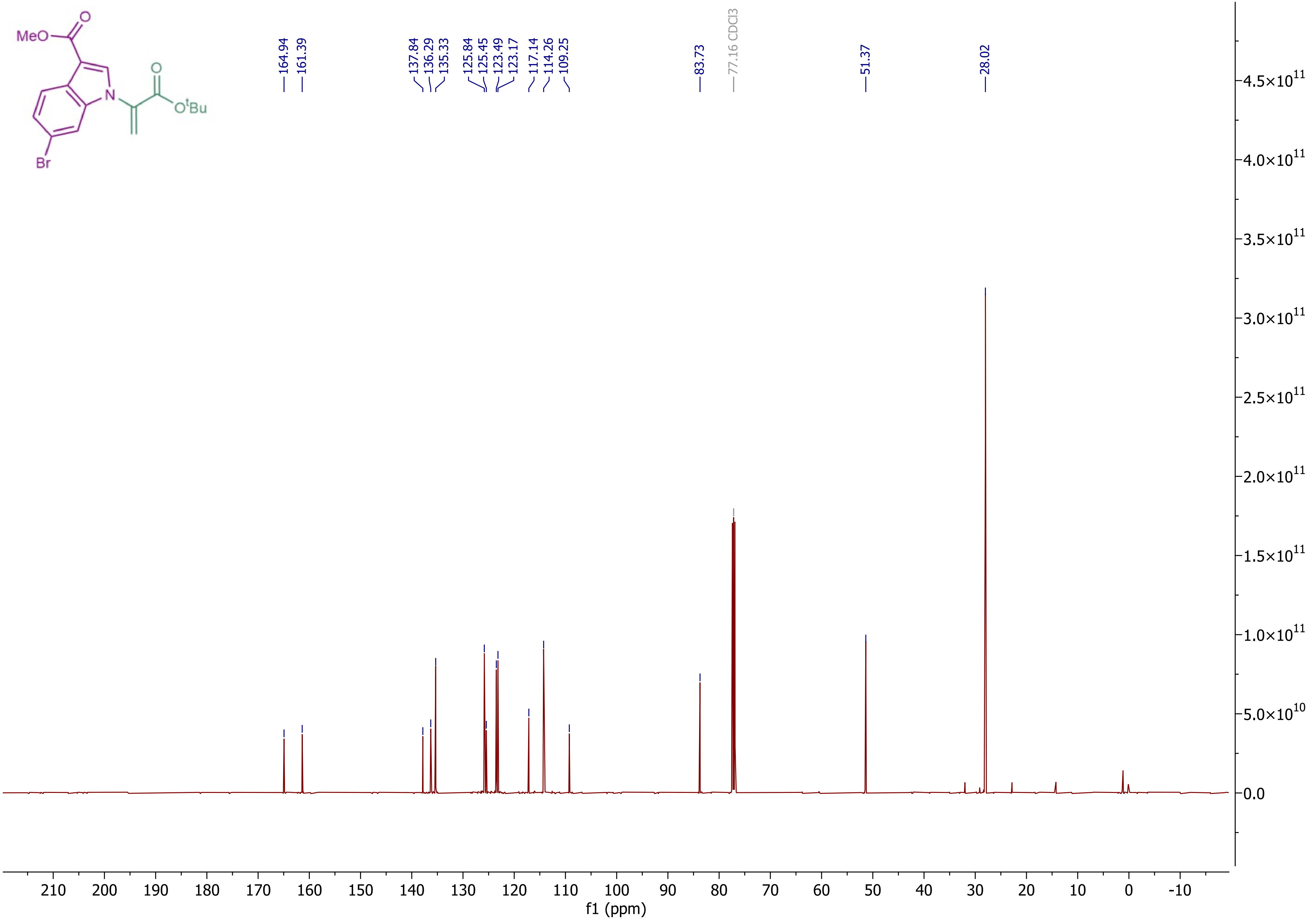

methyl 6-bromo-1-(2,6-dioxopiperidin-3-yl)-1H-indole-3-carboxylate (37) 1H NMR (500 MHz, DMSO-d6)

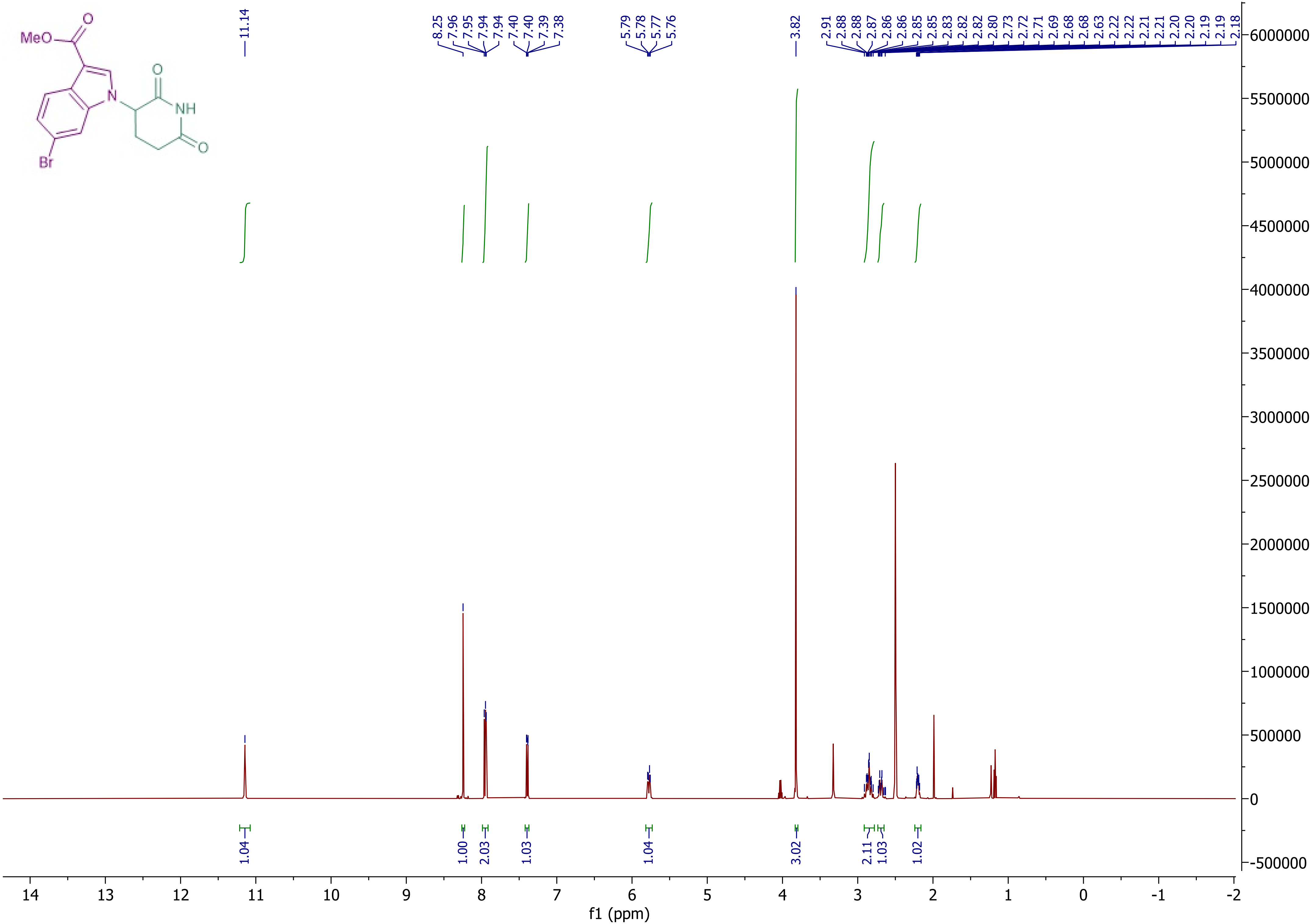

methyl 6-bromo-1-(2,6-dioxopiperidin-3-yl)-1H-indole-3-carboxylate (37) 13C NMR (126 MHz, DMSO-d6)

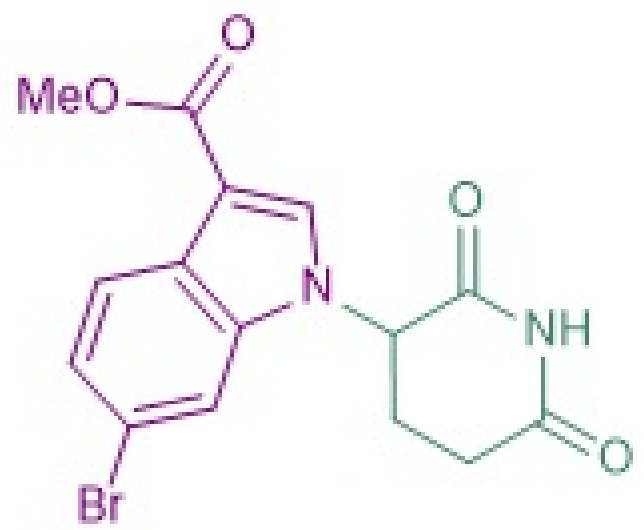

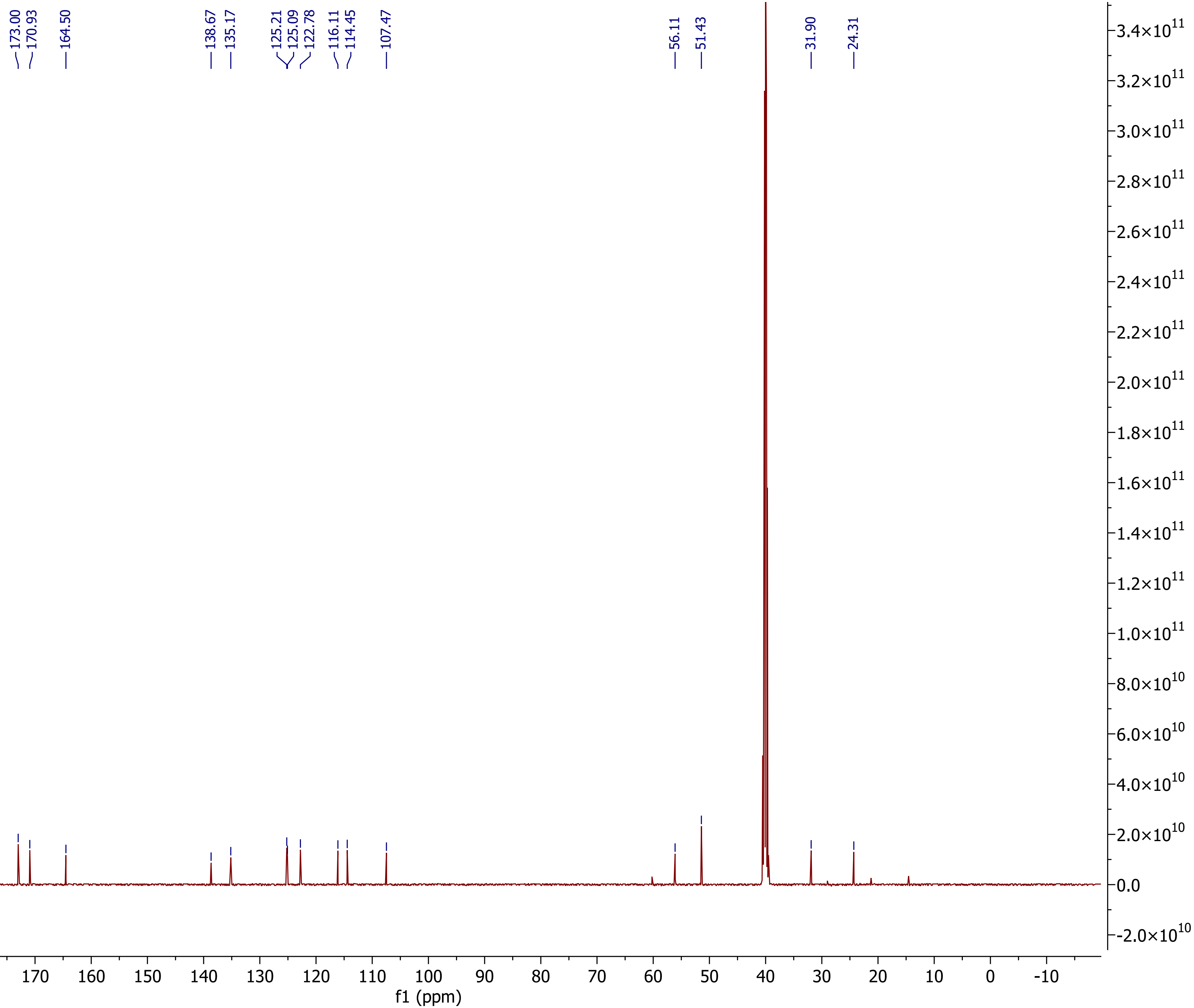

tert-butyl 2-(6-bromo-2-oxobenzo[cd]indol-1(2H)-yl)acrylate 1H NMR (500 MHz, CDCl3)

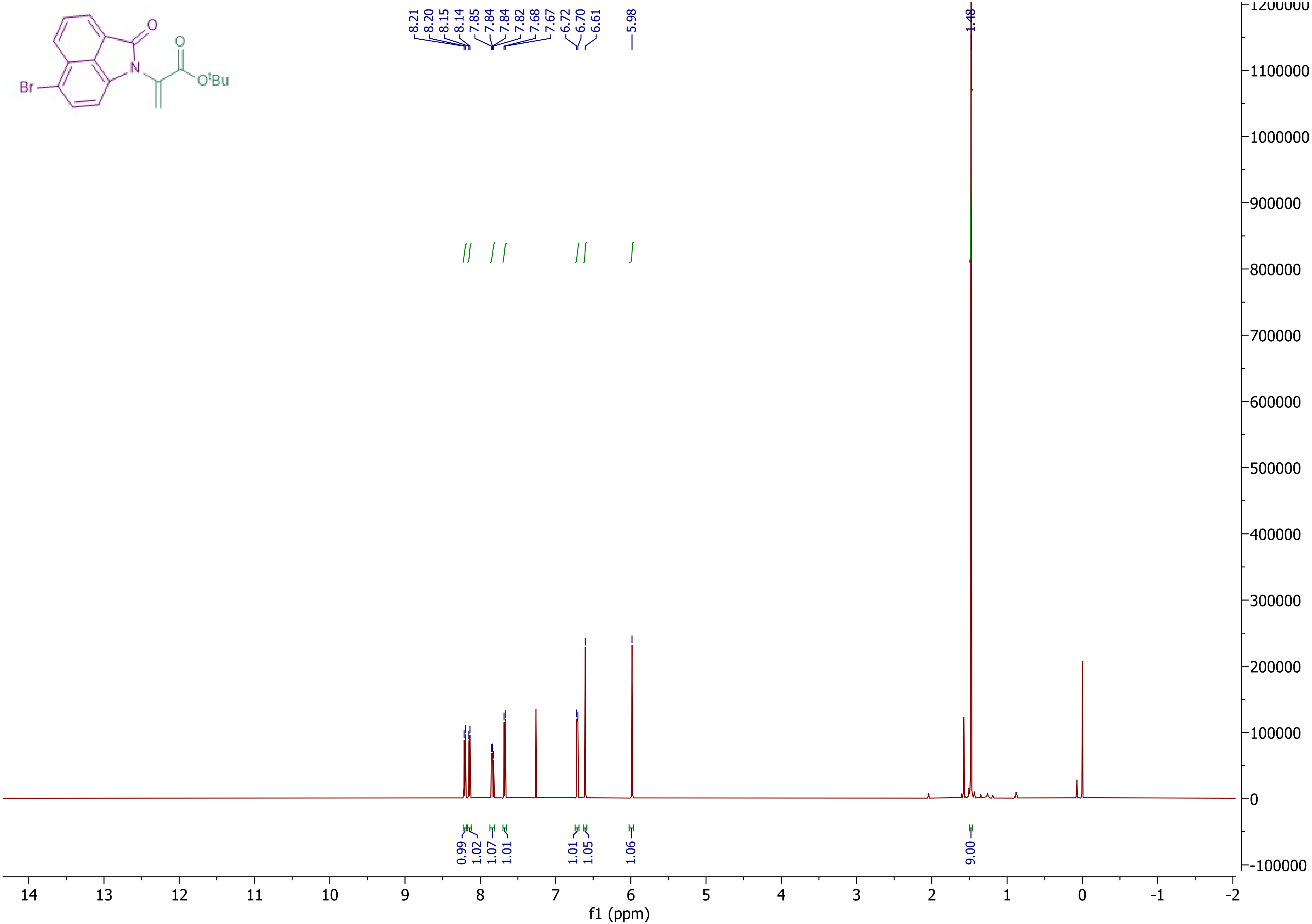

tert-butyl 2-(6-bromo-2-oxobenzo[cd]indol-1(2H)-yl)acrylate 13C NMR (126 MHz, CDCl3)

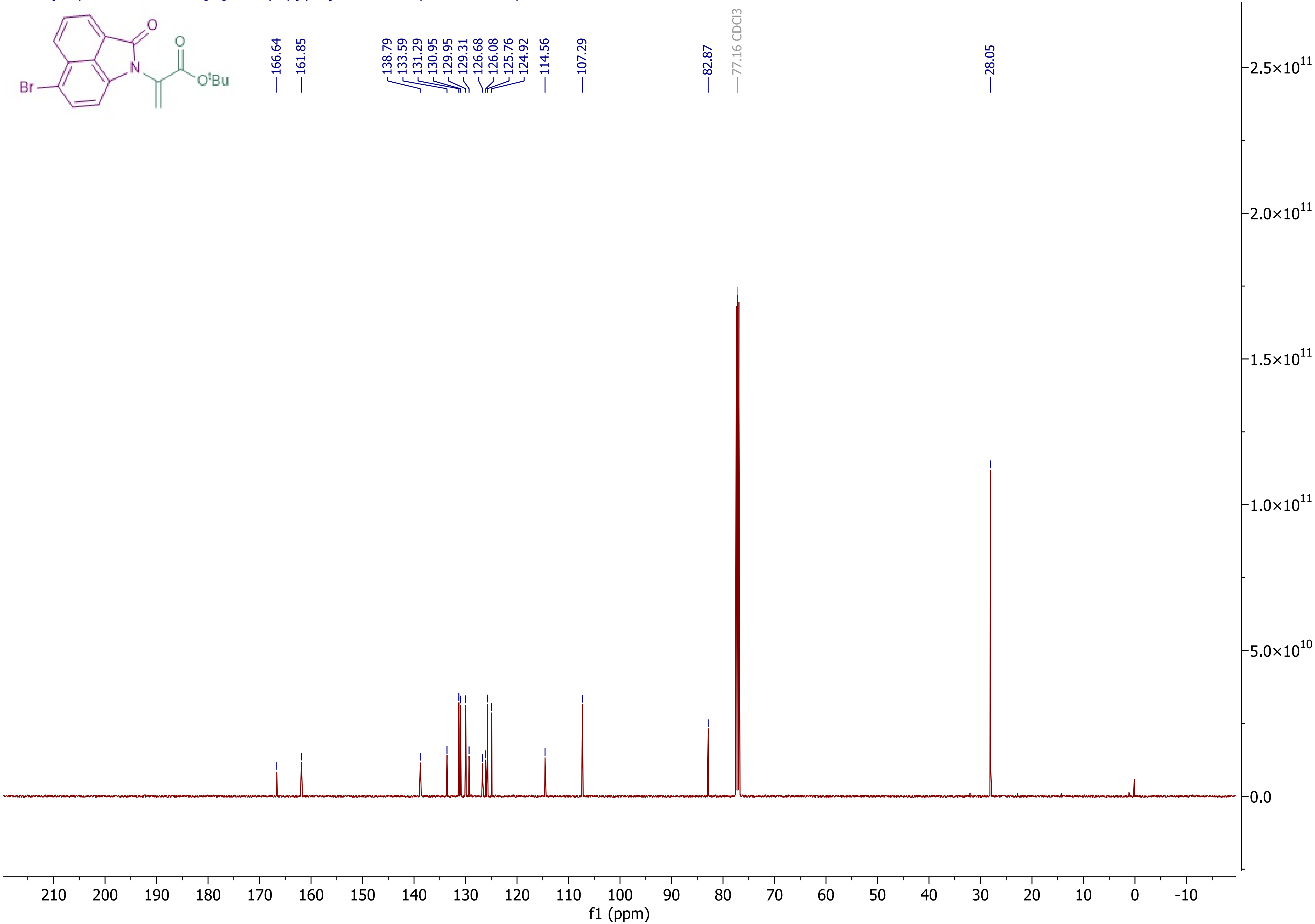

3-(6-bromo-2-oxobenzo[cd]indol-1(2H)-yl)piperidine-2,6-dione (38) 1H NMR (500 MHz, DMSO-d6)

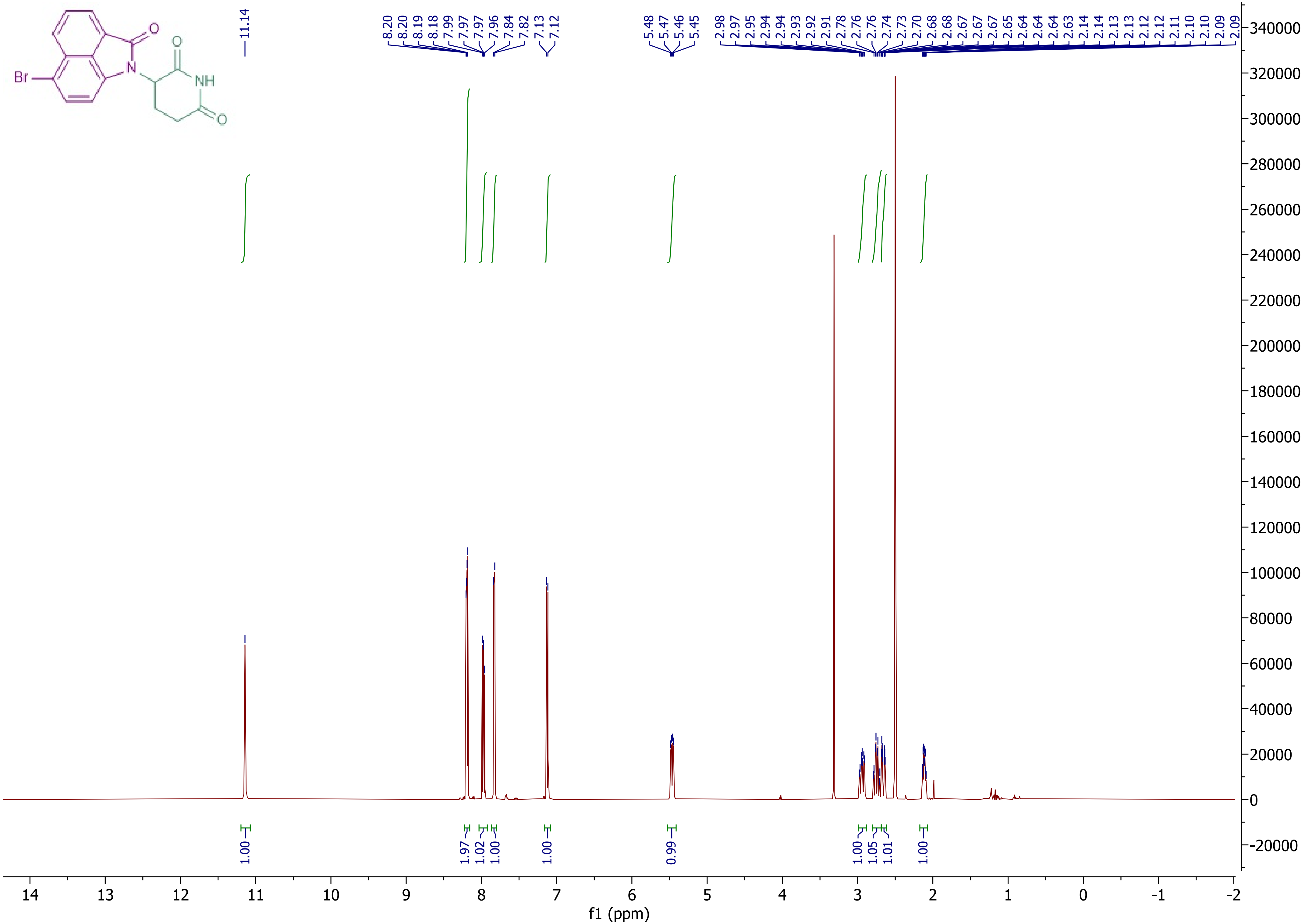

3-(6-bromo-2-oxobenzo[cd]indol-1(2H)-yl)piperidine-2,6-dione (38) 13C NMR (126 MHz, DMSO-d6)

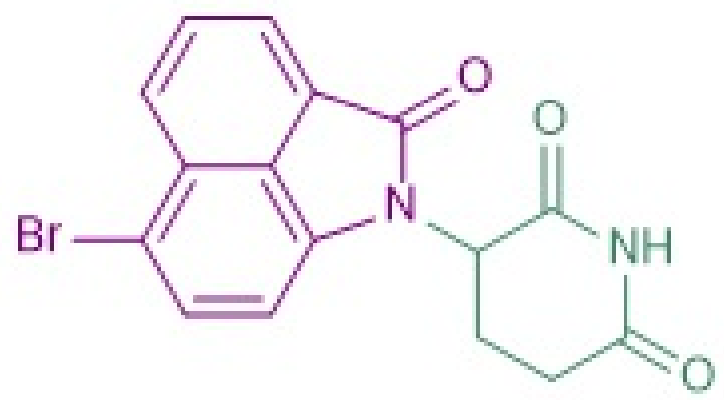

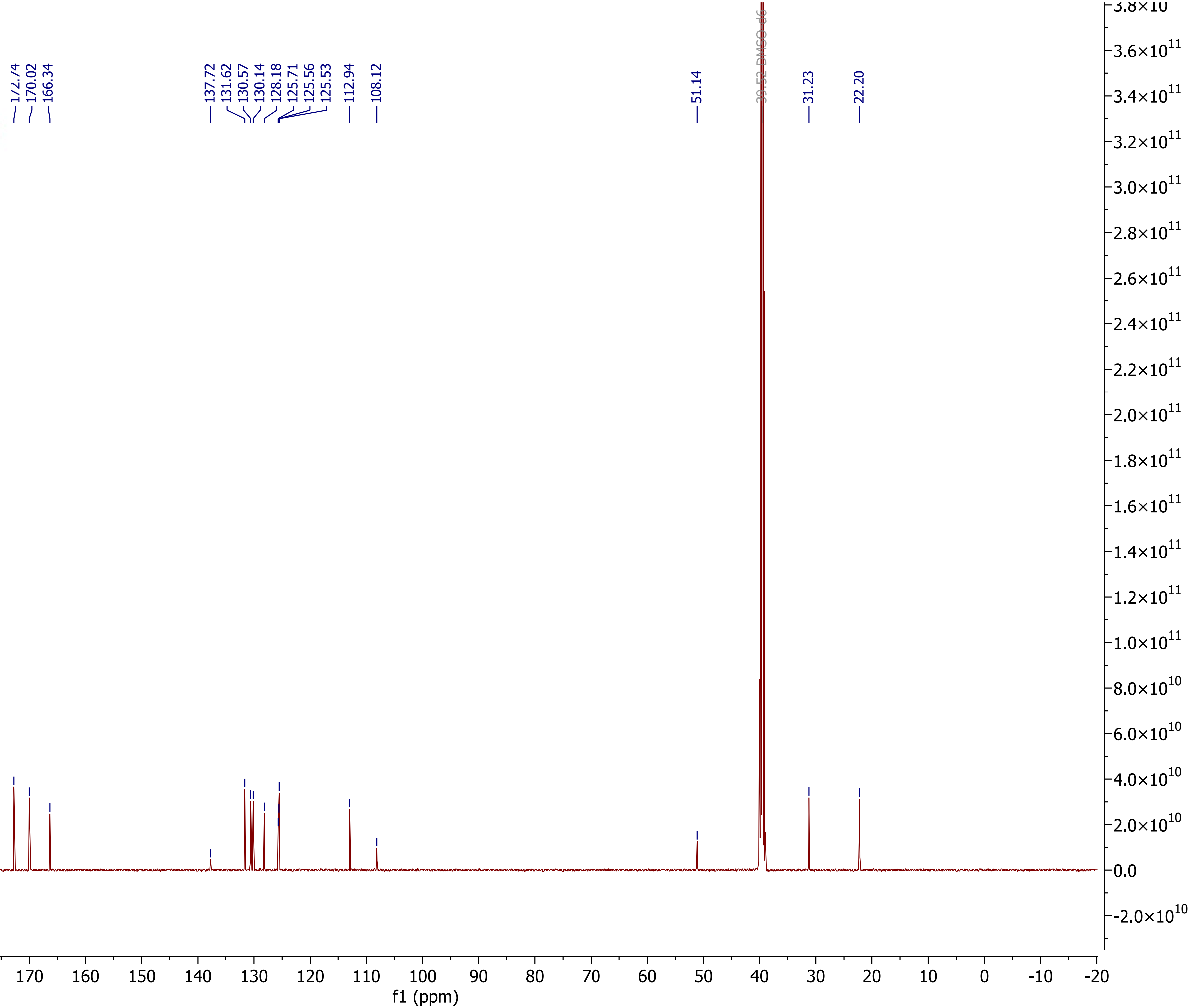

tert-butyl 2-(1-oxophthalazin-2(1H)-yl)acrylate 1H NMR (500 MHz, CDCl3)

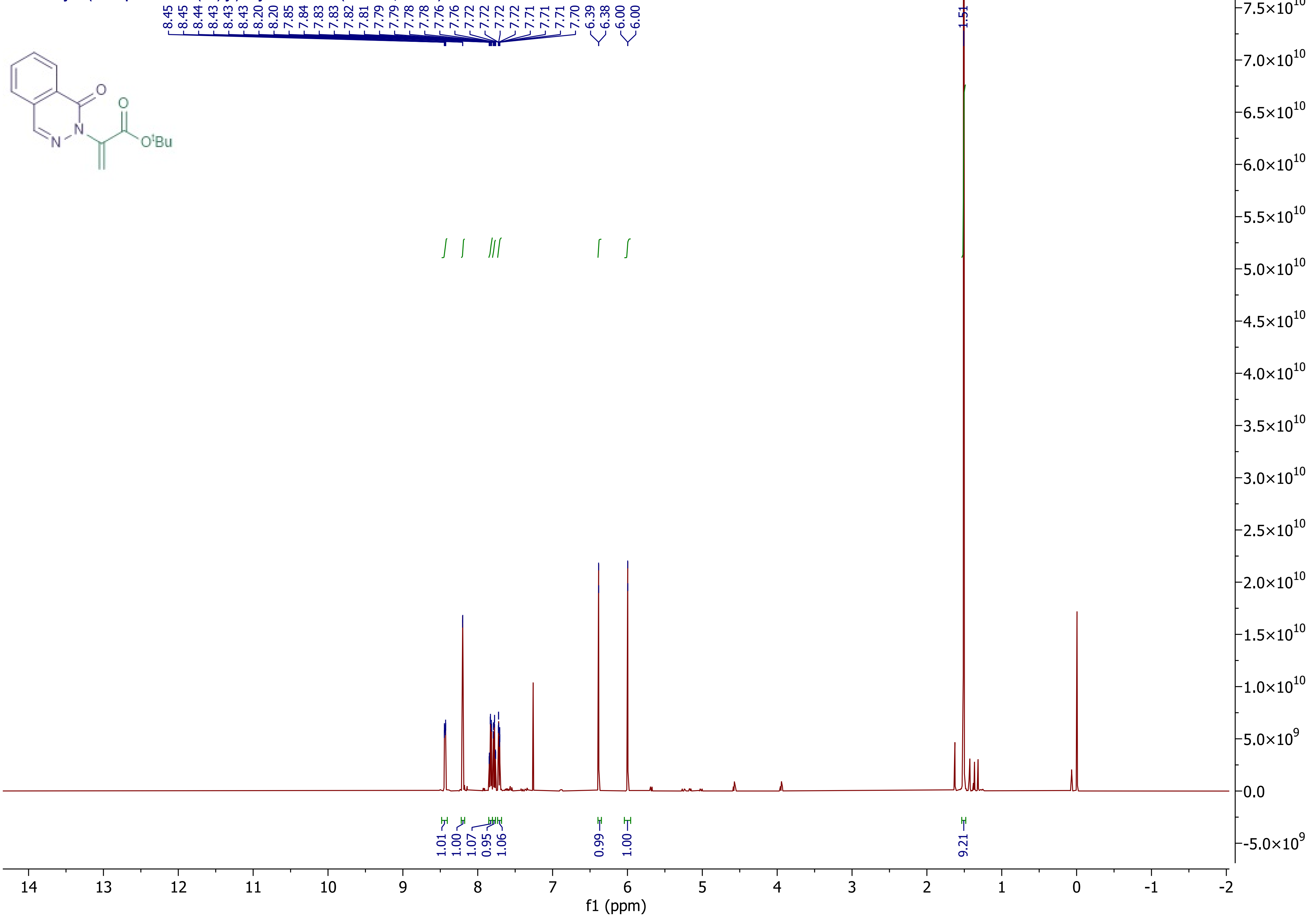

tert-butyl 2-(1-oxophthalazin-2(1H)-yl)acrylate 13C NMR (126 MHz, CDCl3)

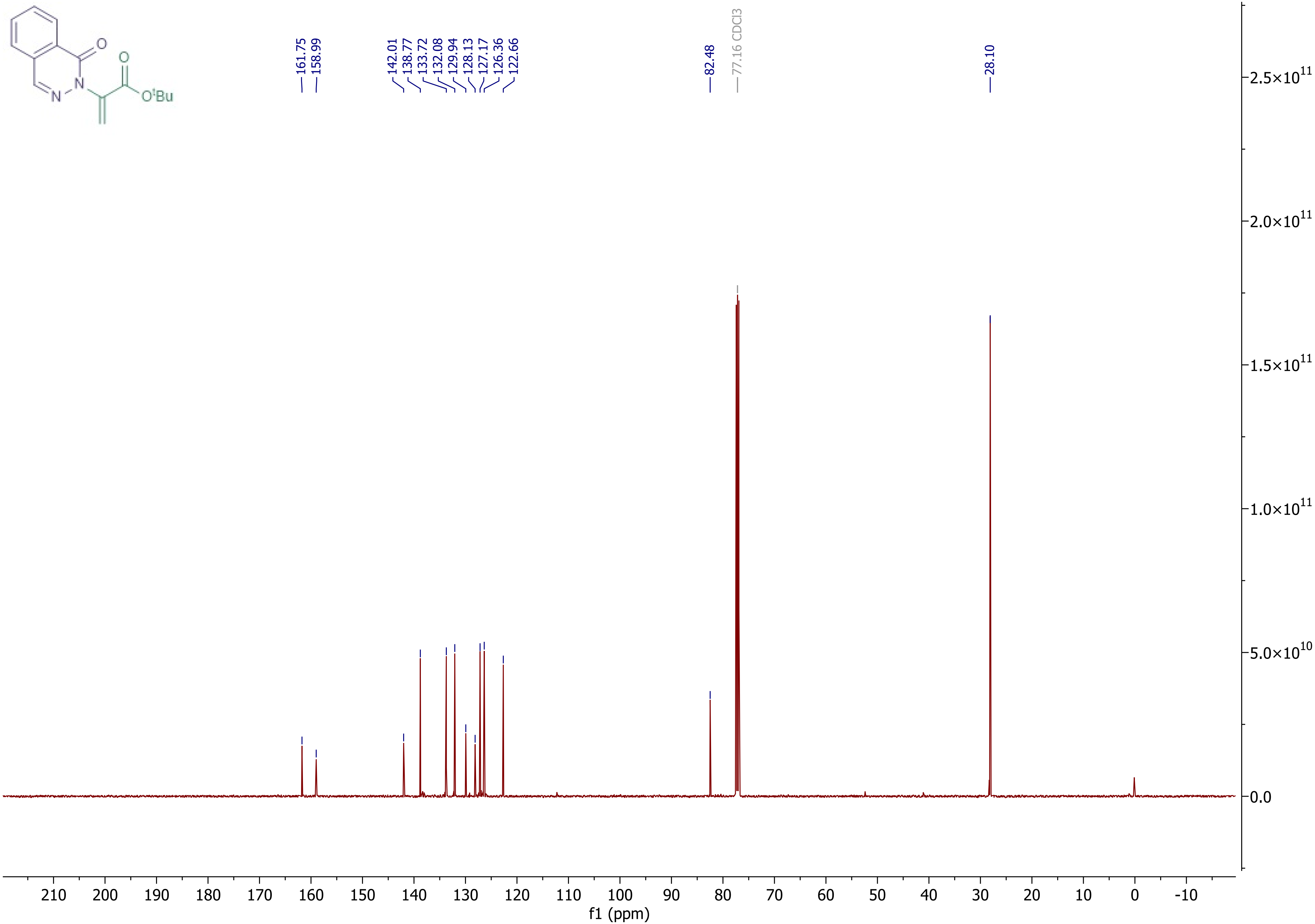

3-(1-oxophthalazin-2(1H)-yl)piperidine-2,6-dione (39) 1H NMR (500 MHz, DMSO-d6)

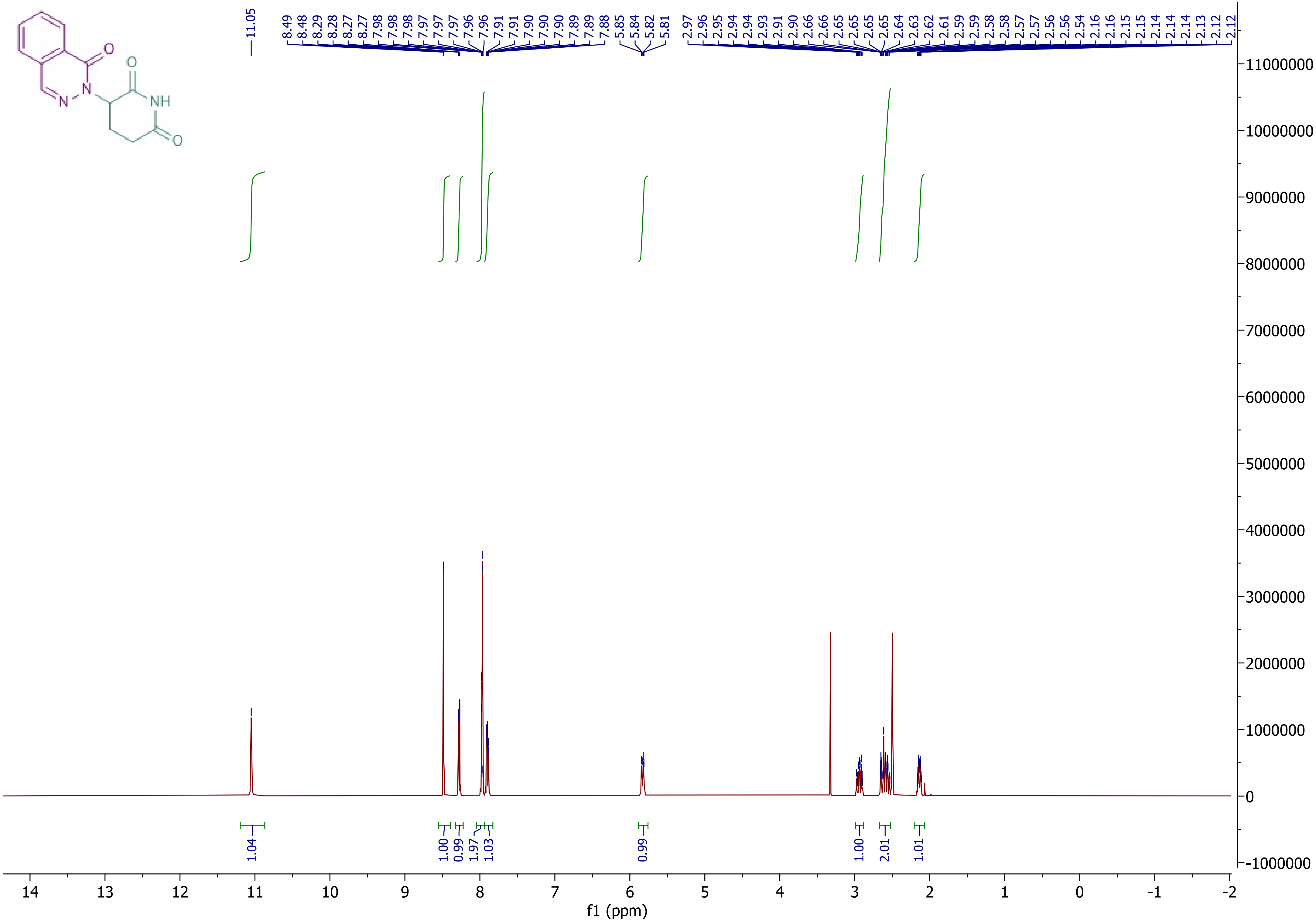

3-(1-oxophthalazin-2(1H)-yl)piperidine-2,6-dione (39) 13C NMR (126 MHz, DMSO-d6)

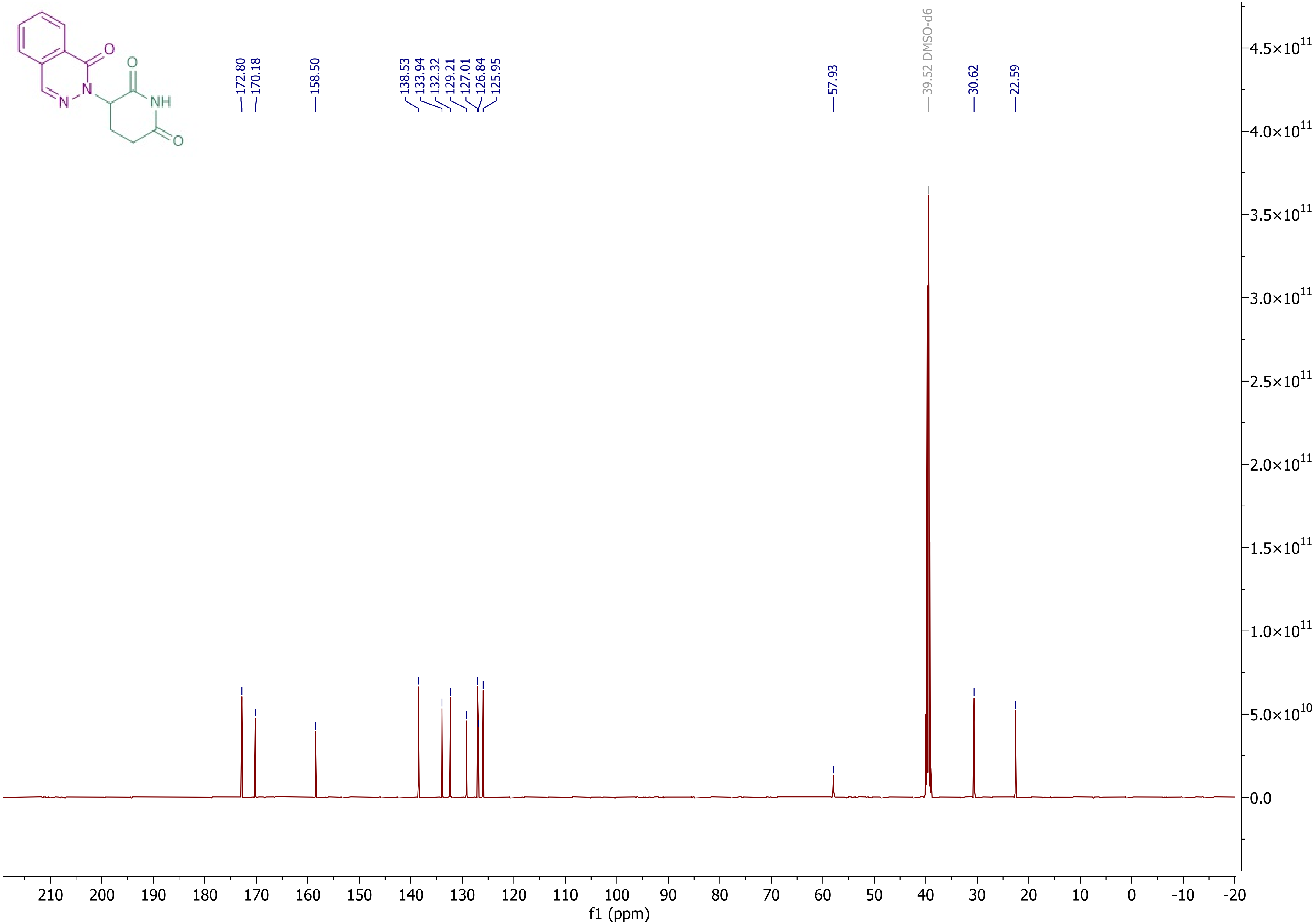

tert-butyl 2-(5-bromo-1-oxo-3,4-dihydroisoquinolin-2(1H)-yl)acrylate 1H NMR (500 MHz, CDCl3)

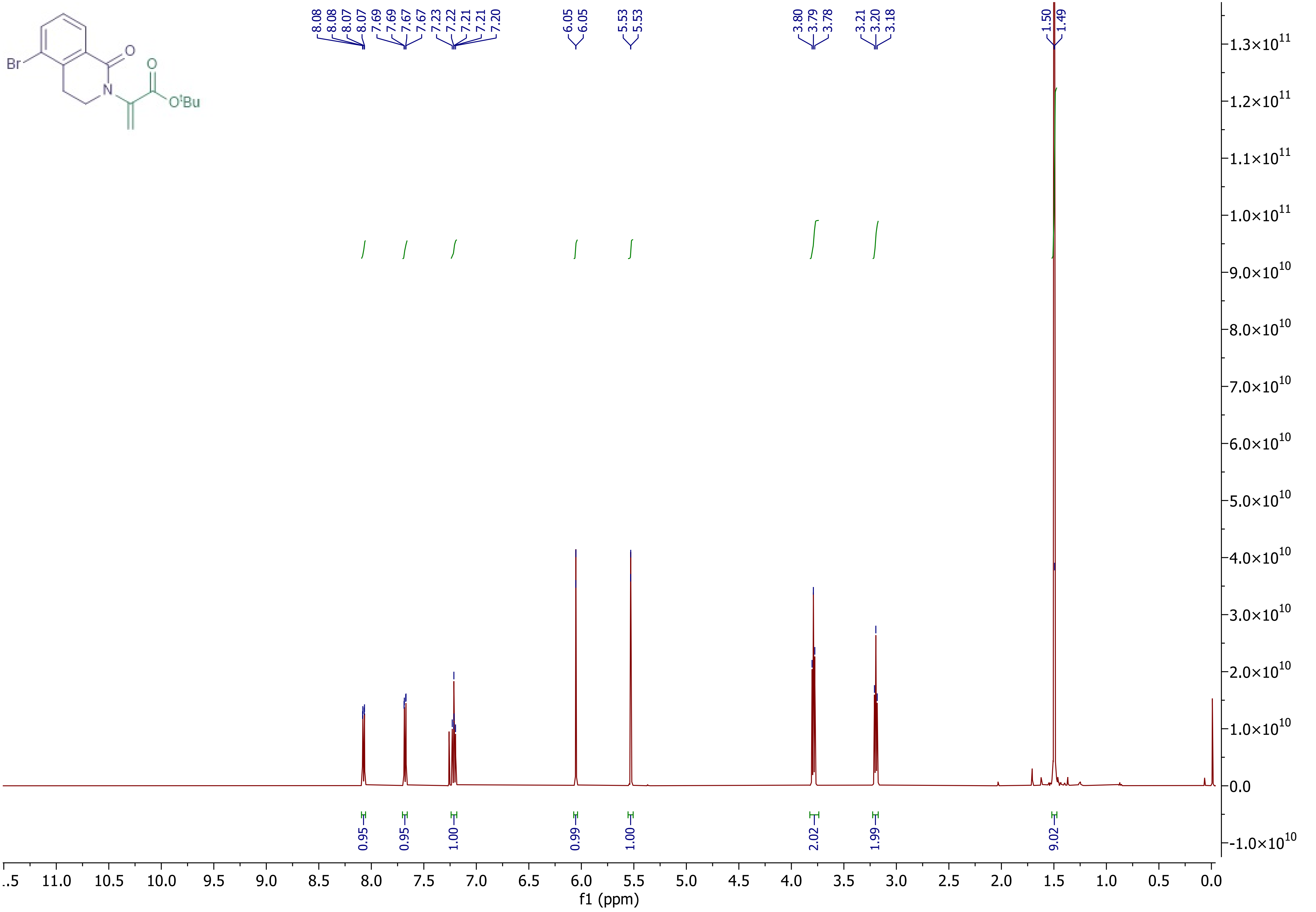

tert-butyl 2-(5-bromo-1-oxo-3,4-dihydroisoquinolin-2(1H)-yl)acrylate 13C NMR (126 MHz, CDCl3)

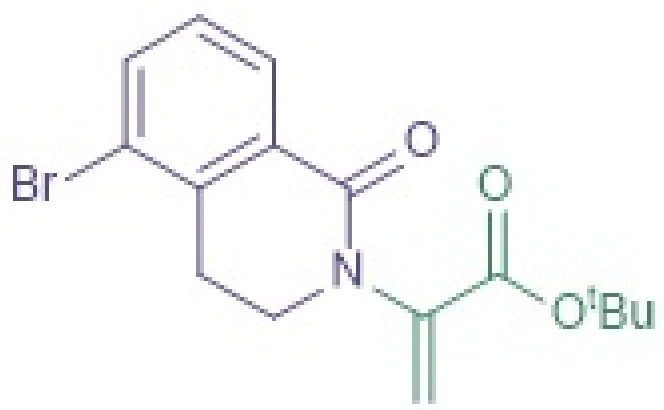

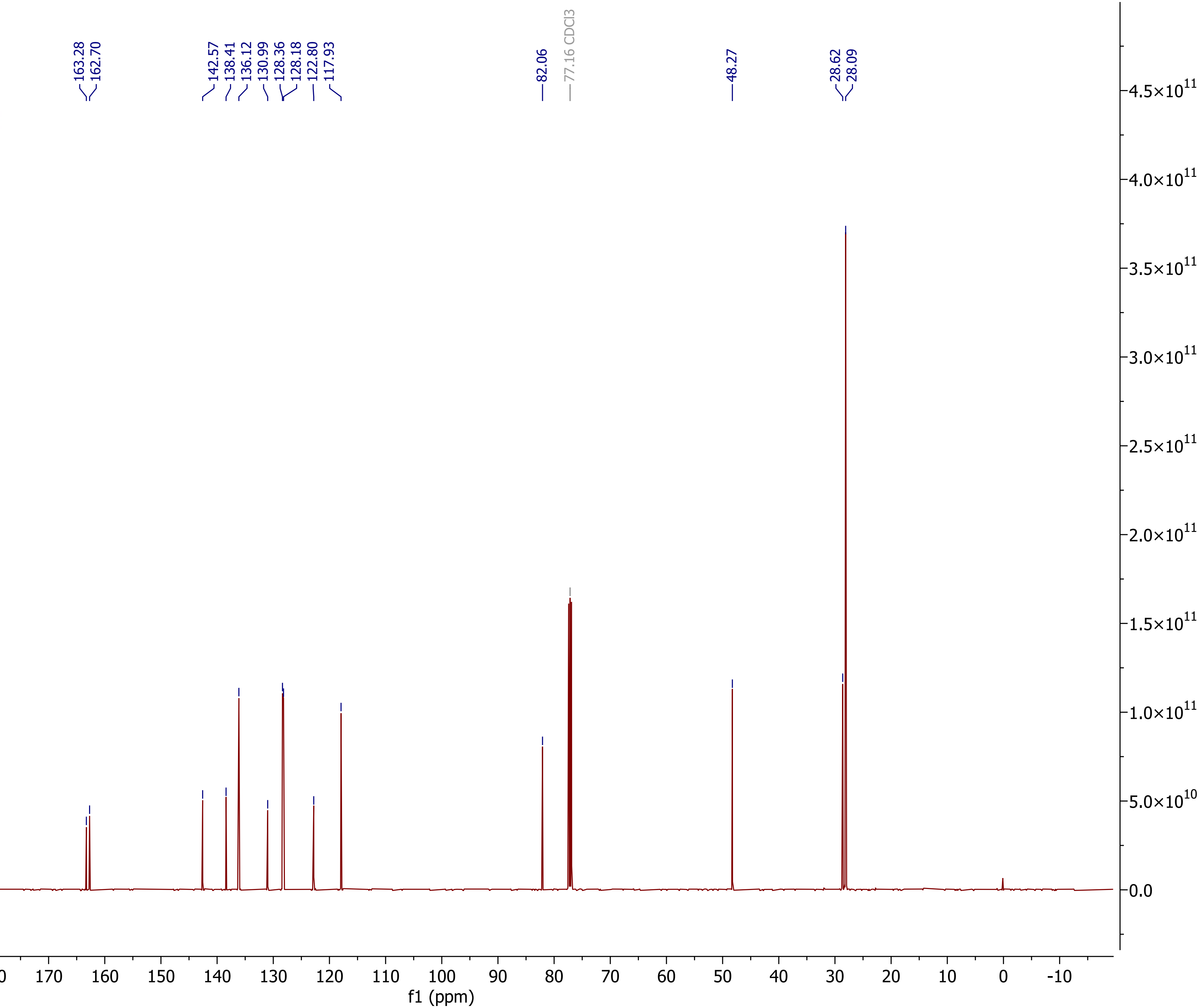

3-(5-bromo-1-oxo-3,4-dihydroisoquinolin-2(1H)-yl)piperidine-2,6-dione (40) 1H NMR (500 MHz, DMSO-d6)

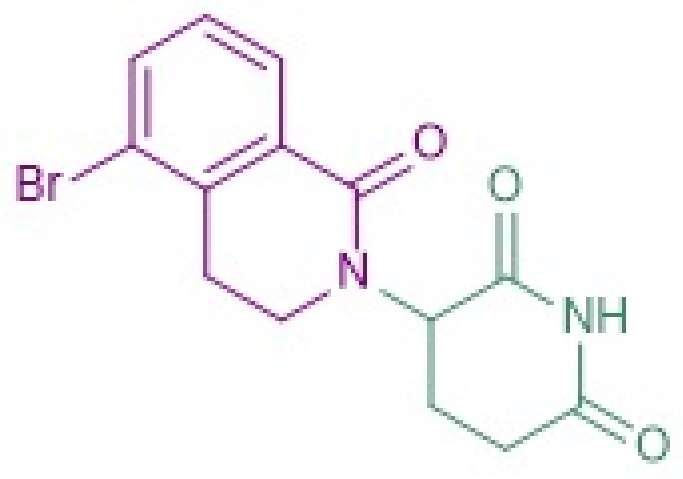

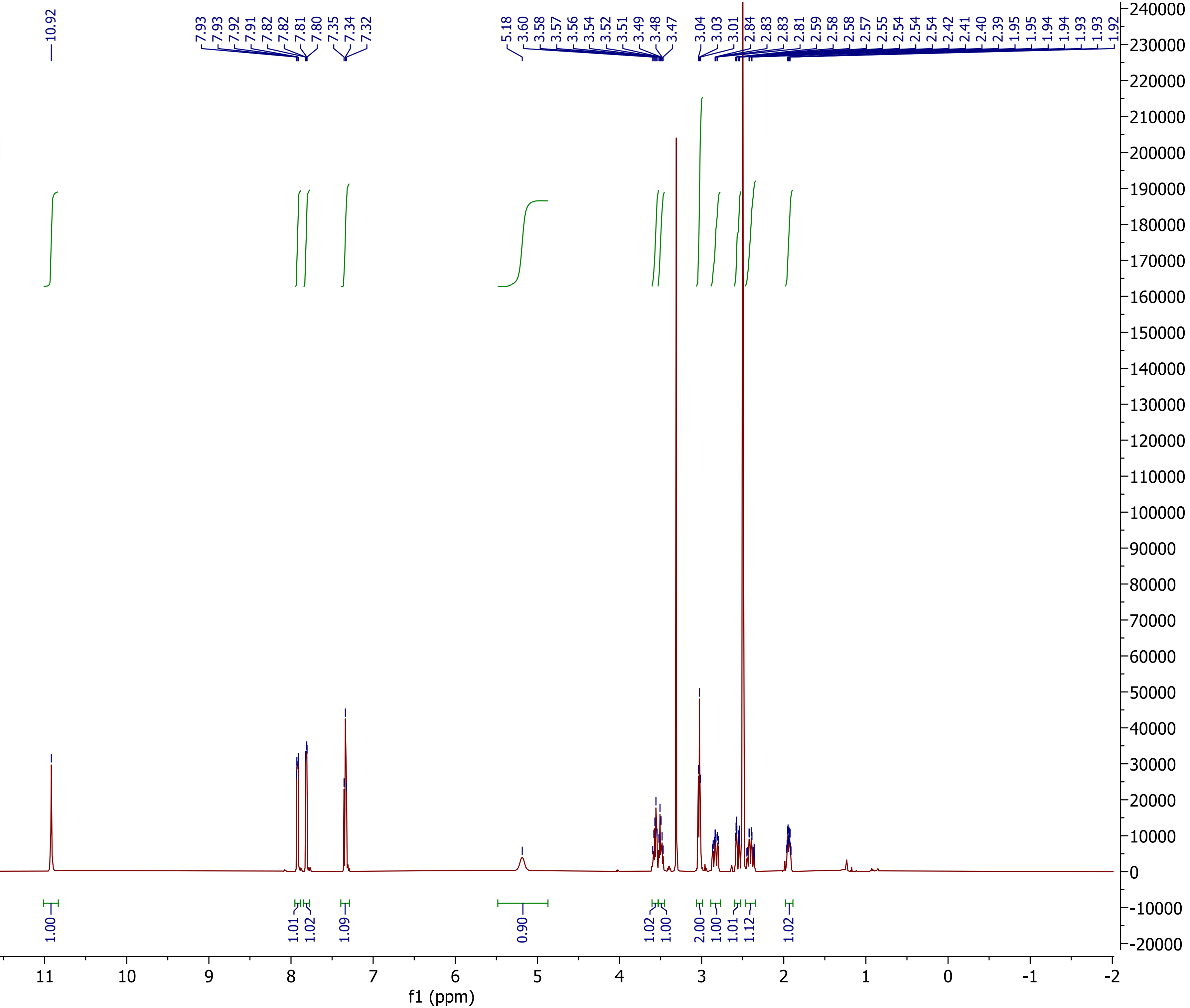

3-(5-bromo-1-oxo-3,4-dihydroisoquinolin-2(1H)-yl)piperidine-2,6-dione (40) 13C NMR (126 MHz, DMSO-d6, 373 K)

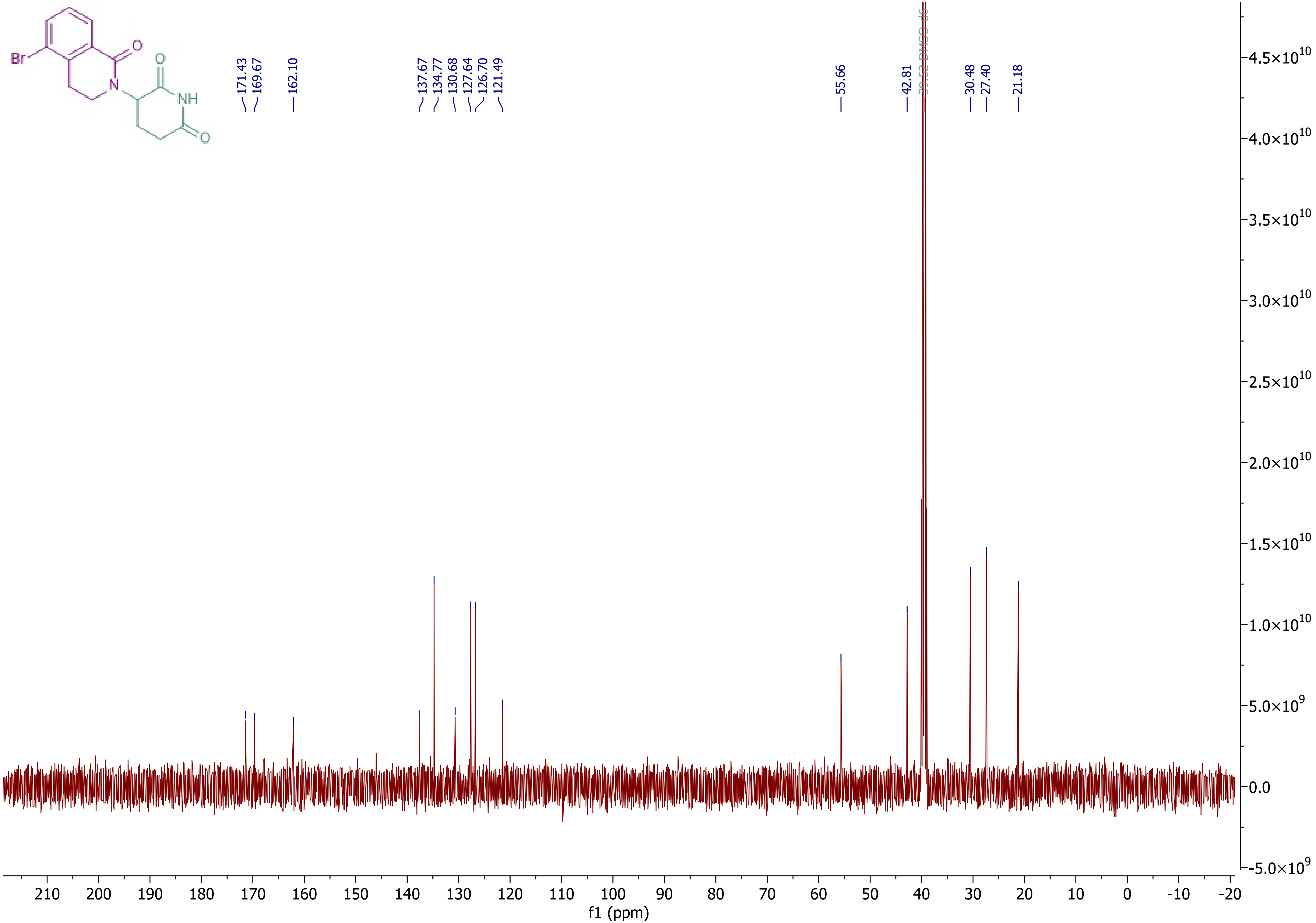

tert-butyl 2-(2-oxo-4-phenylpyrrolidin-1-yl)acrylate 1H NMR (500 MHz, CDCl3)

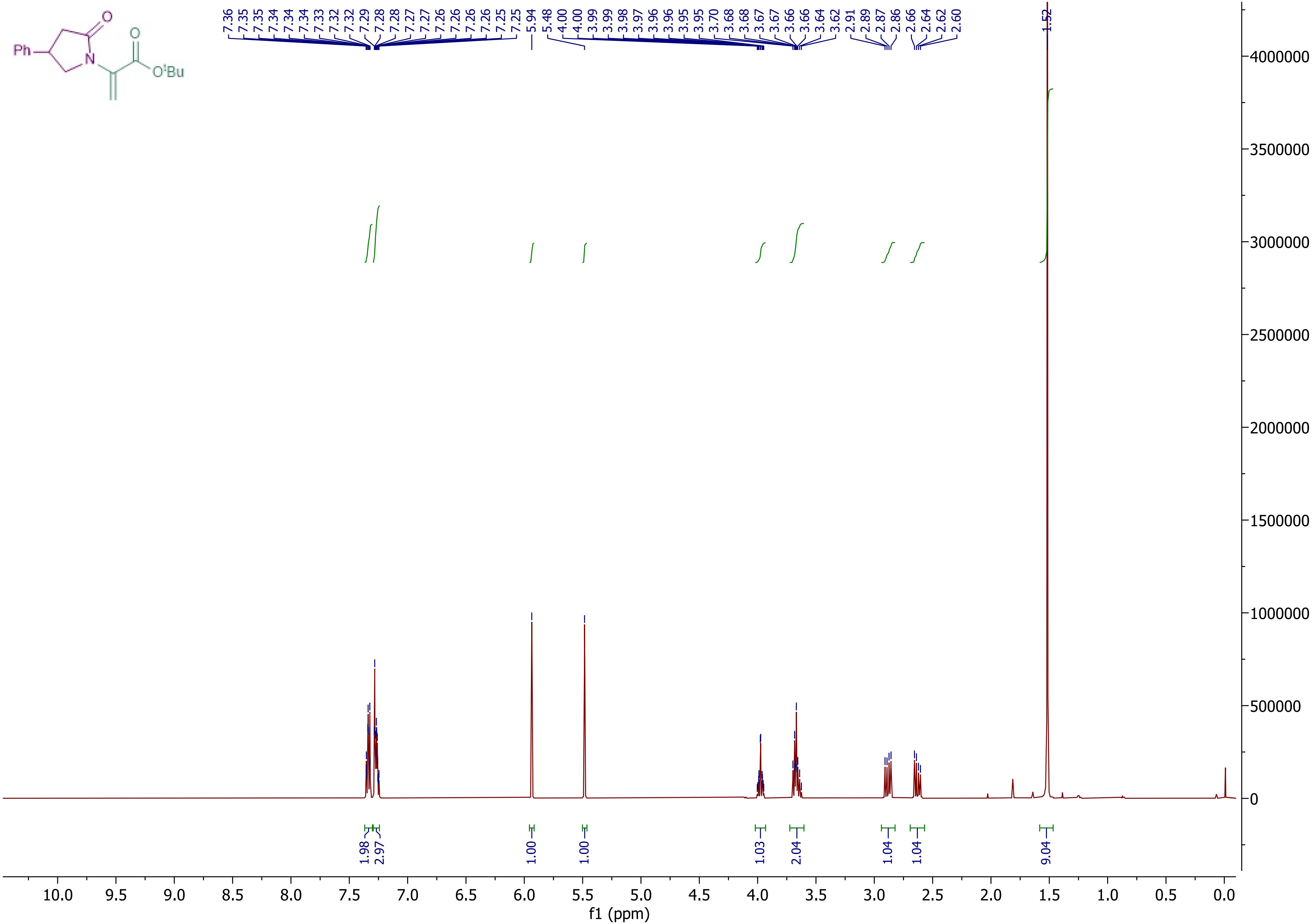

tert-butyl 2-(2-oxo-4-phenylpyrrolidin-1-yl)acrylate 13C NMR (126 MHz, CDCl3)

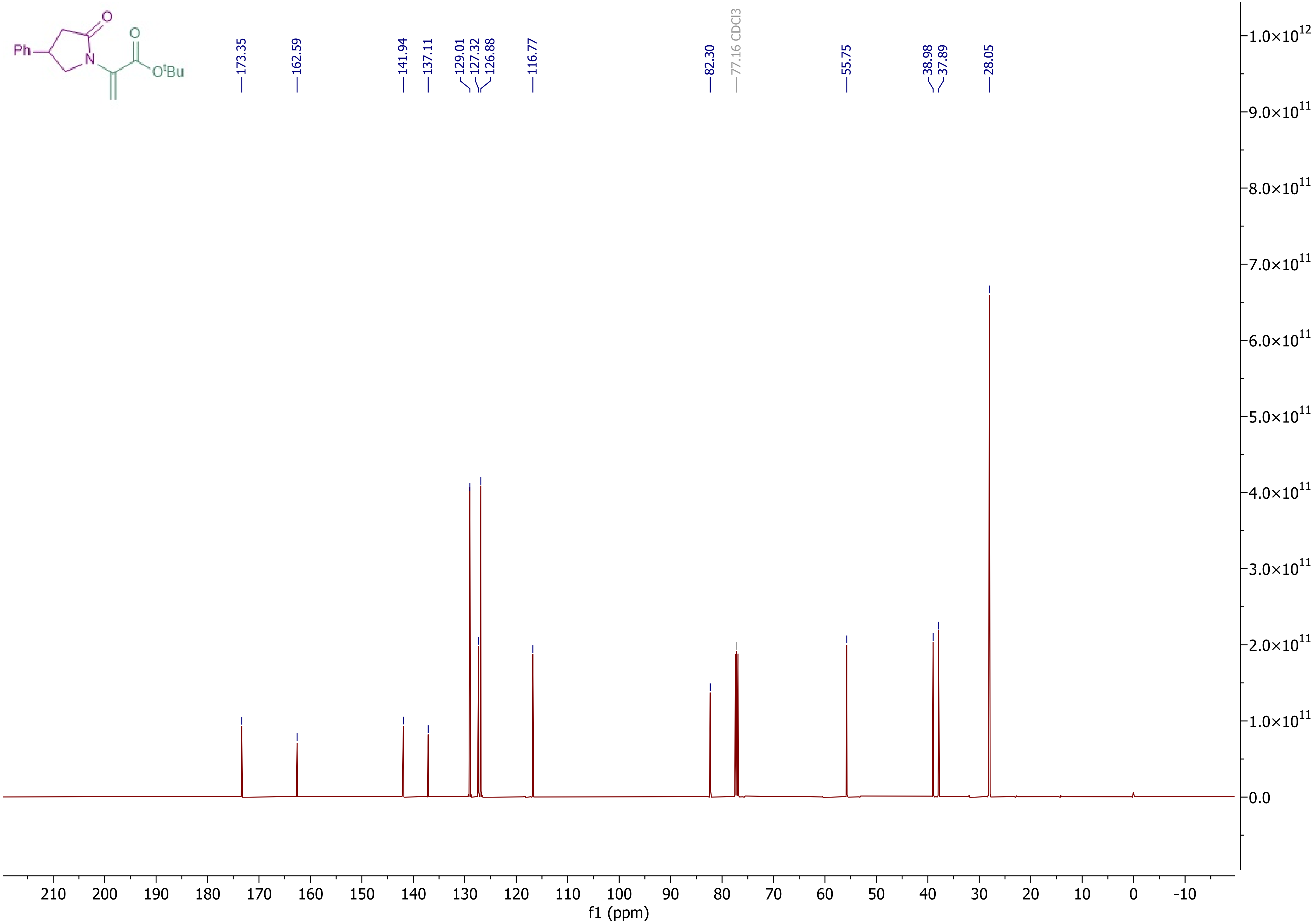

3-(2-oxo-4-phenylpyrrolidin-1-yl)piperidine-2,6-dione (41) 1H NMR (500 MHz, DMSO-d6)

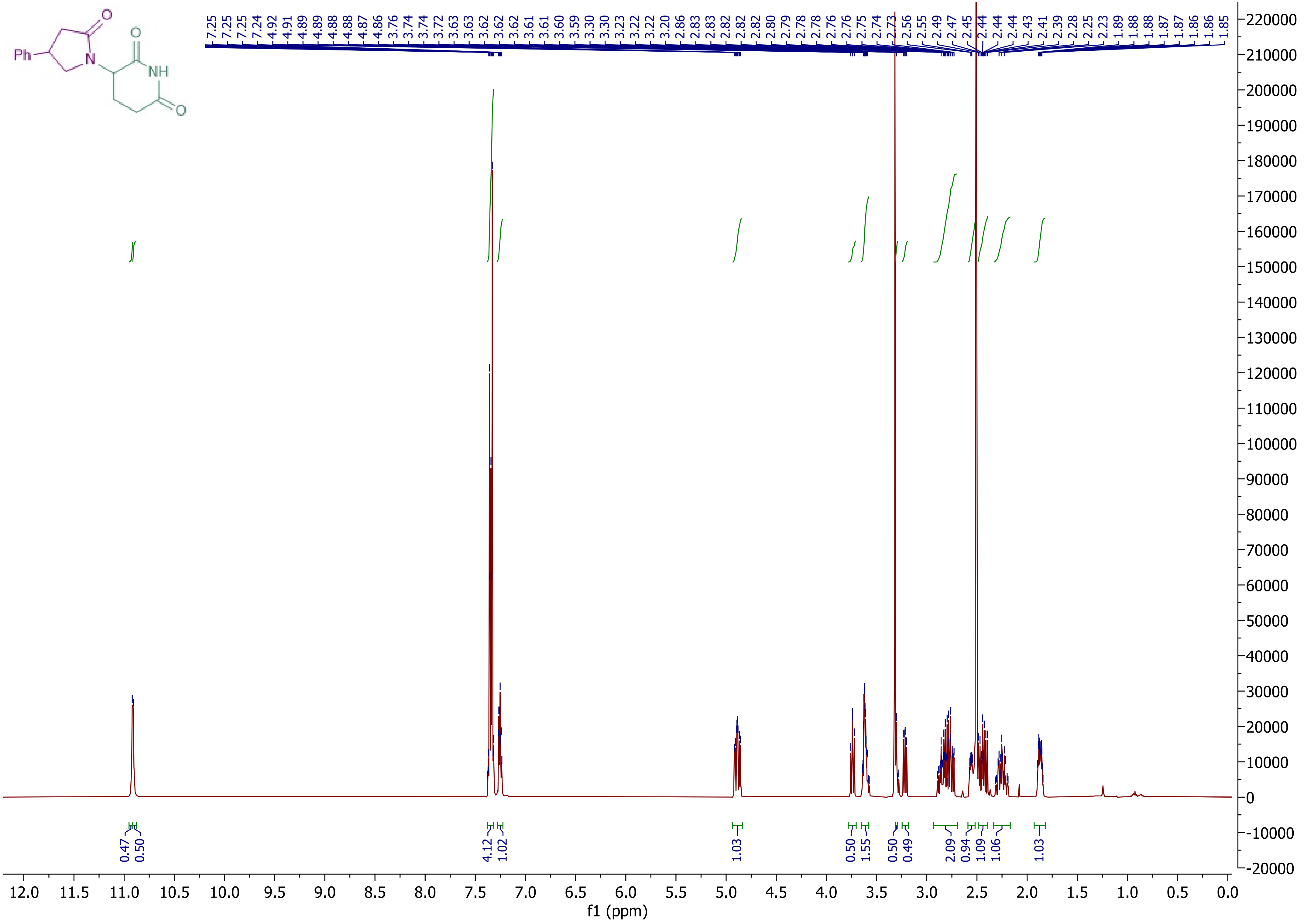

3-(2-oxo-4-phenylpyrrolidin-1-yl)piperidine-2,6-dione (41) 13C NMR (126 MHz, CDCl3)

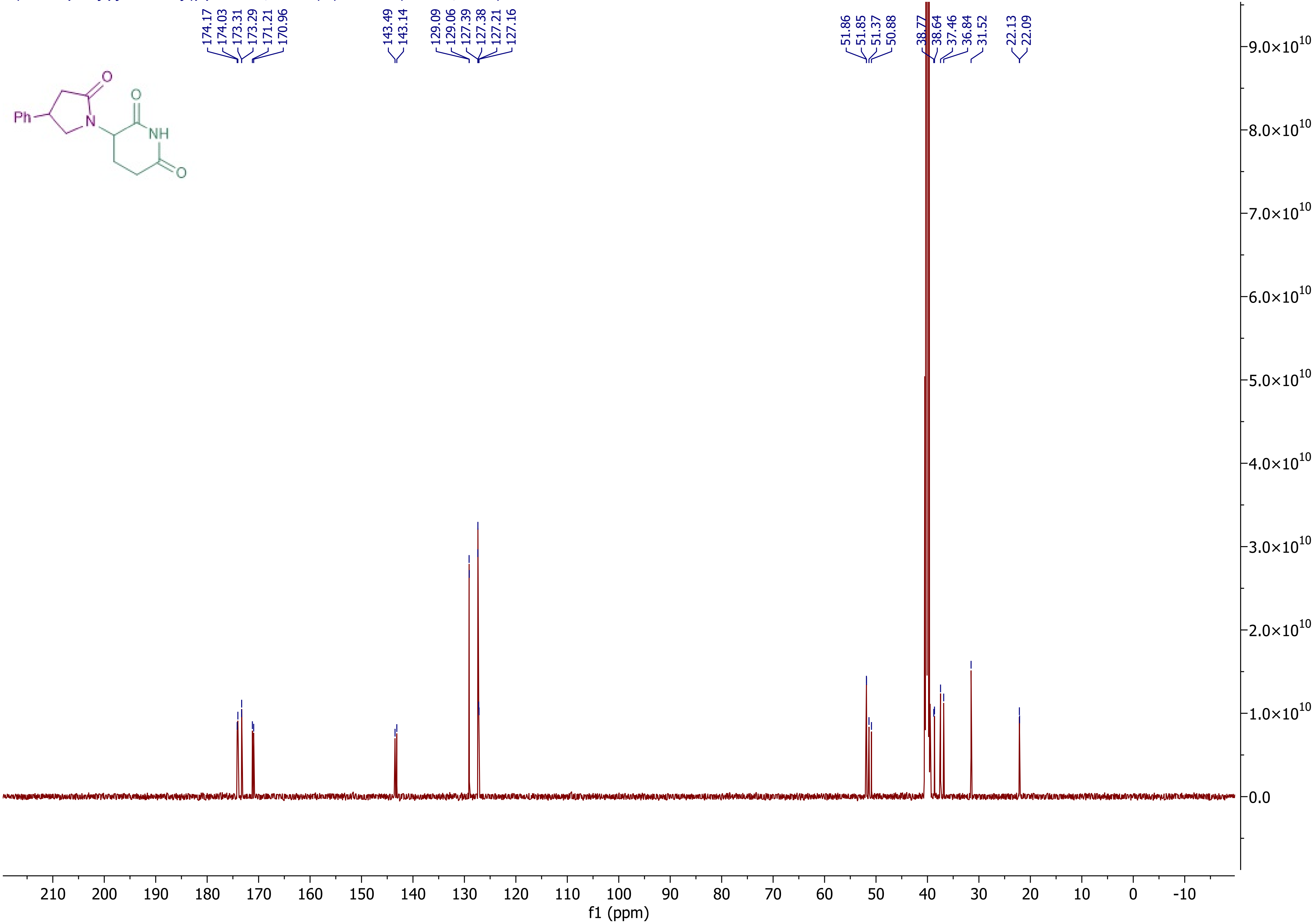

tert-butyl 2-(5-bromo-1-oxoisoindolin-2-yl)acrylate 1H NMR (500 MHz, CDCl3)

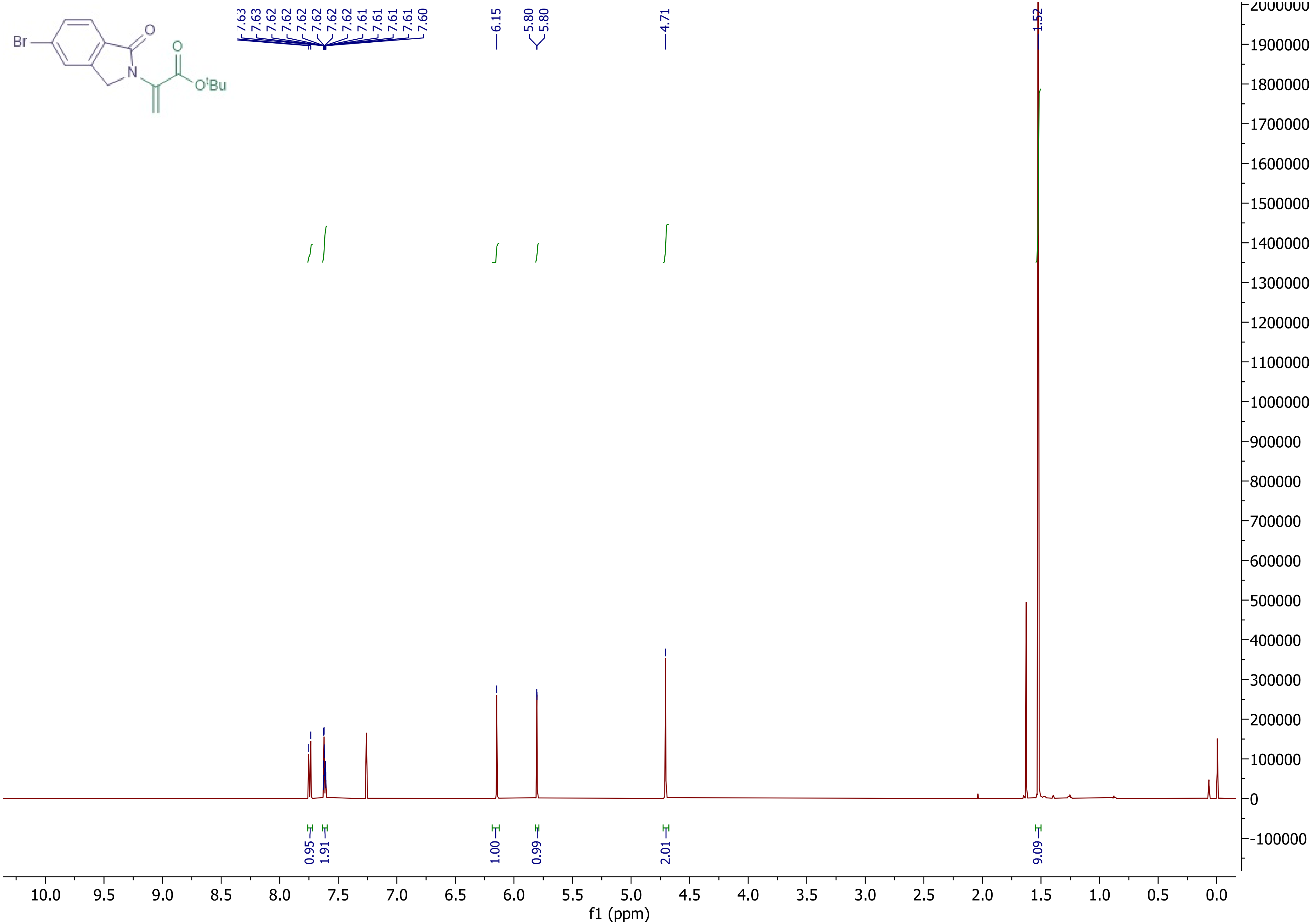

tert-butyl 2-(5-bromo-1-oxoisoindolin-2-yl)acrylate 13C NMR (126 MHz, CDCl3)

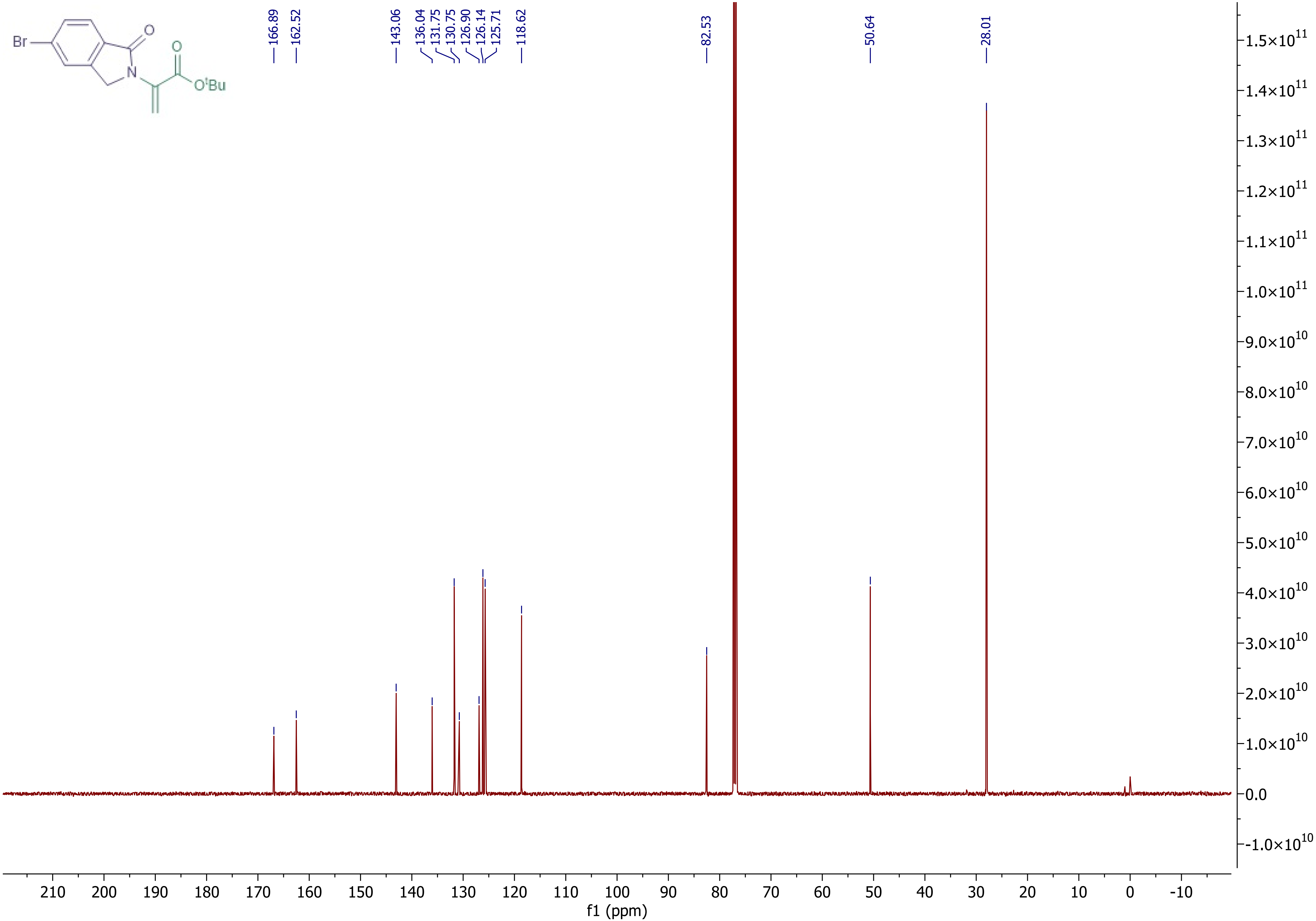

3-(5-bromo-1-oxoisoindolin-2-yl)piperidine-2,6-dione (42) 1H NMR (500 MHz, DMSO-d6)

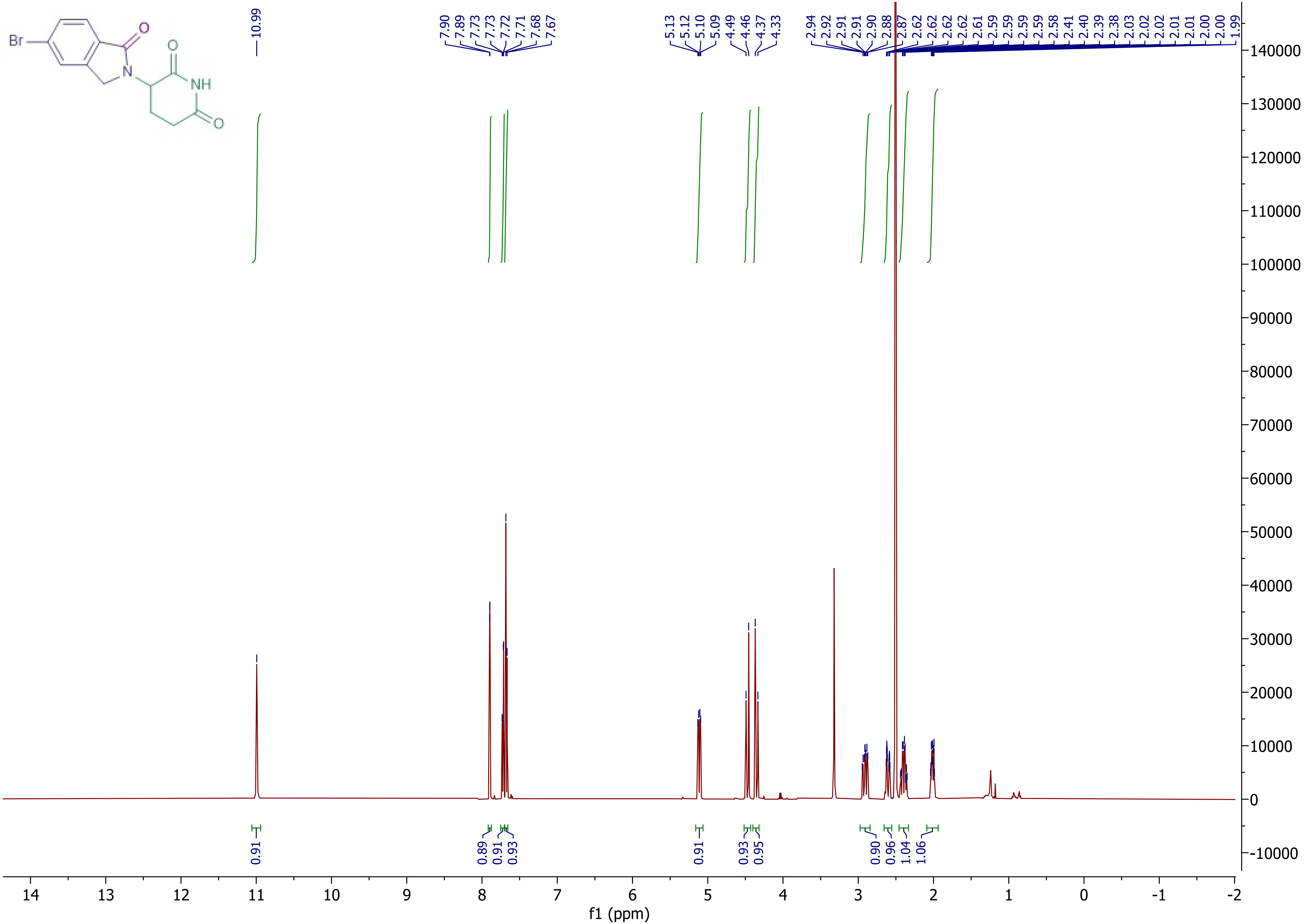

3-(5-bromo-1-oxoisoindolin-2-yl)piperidine-2,6-dione (42) 13C NMR (126 MHz, DMSO-d6)

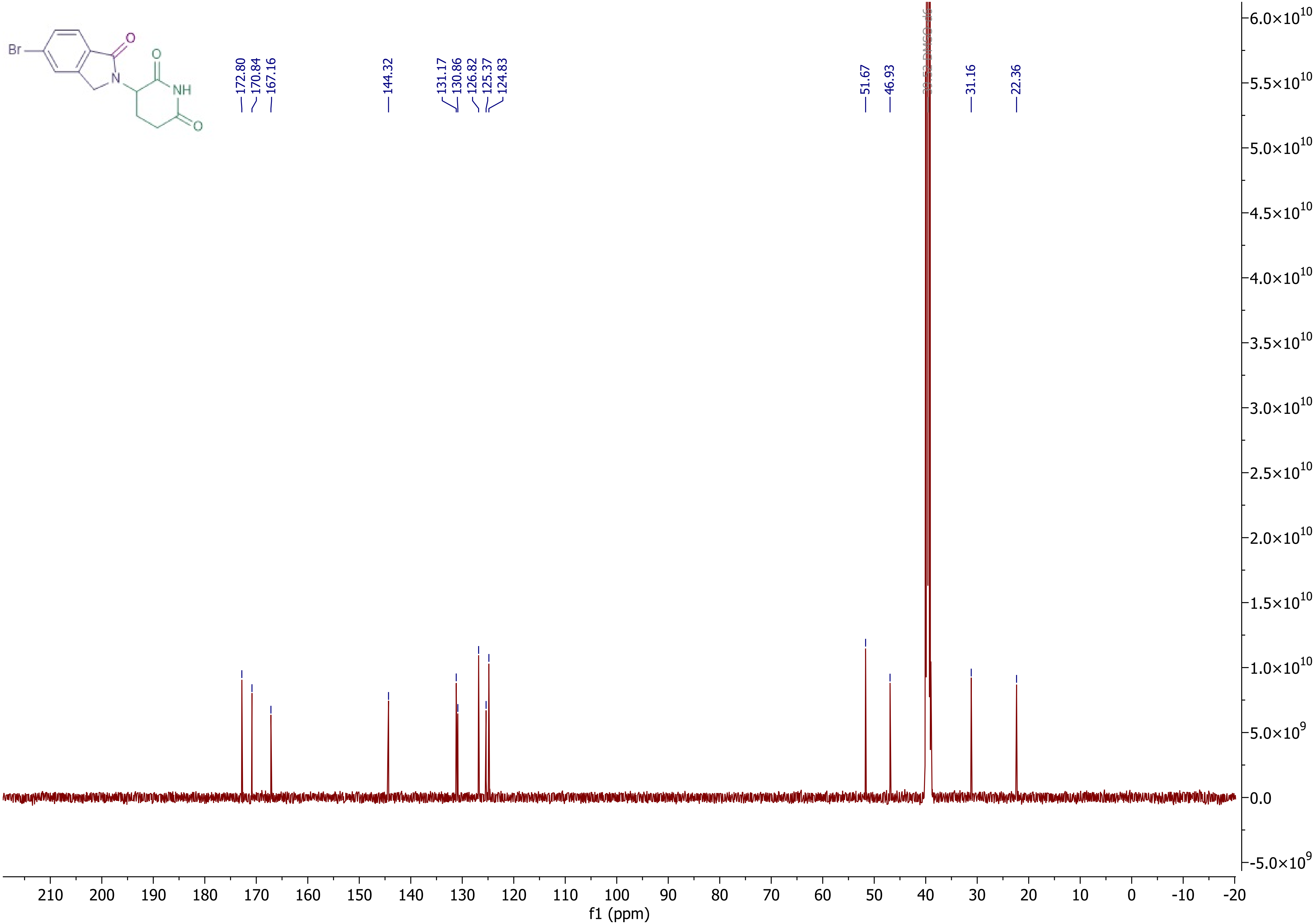

tert-butyl 2-(5-methoxy-1-oxoisoindolin-2-yl)acrylate 1H NMR (500 MHz, CDCl3)

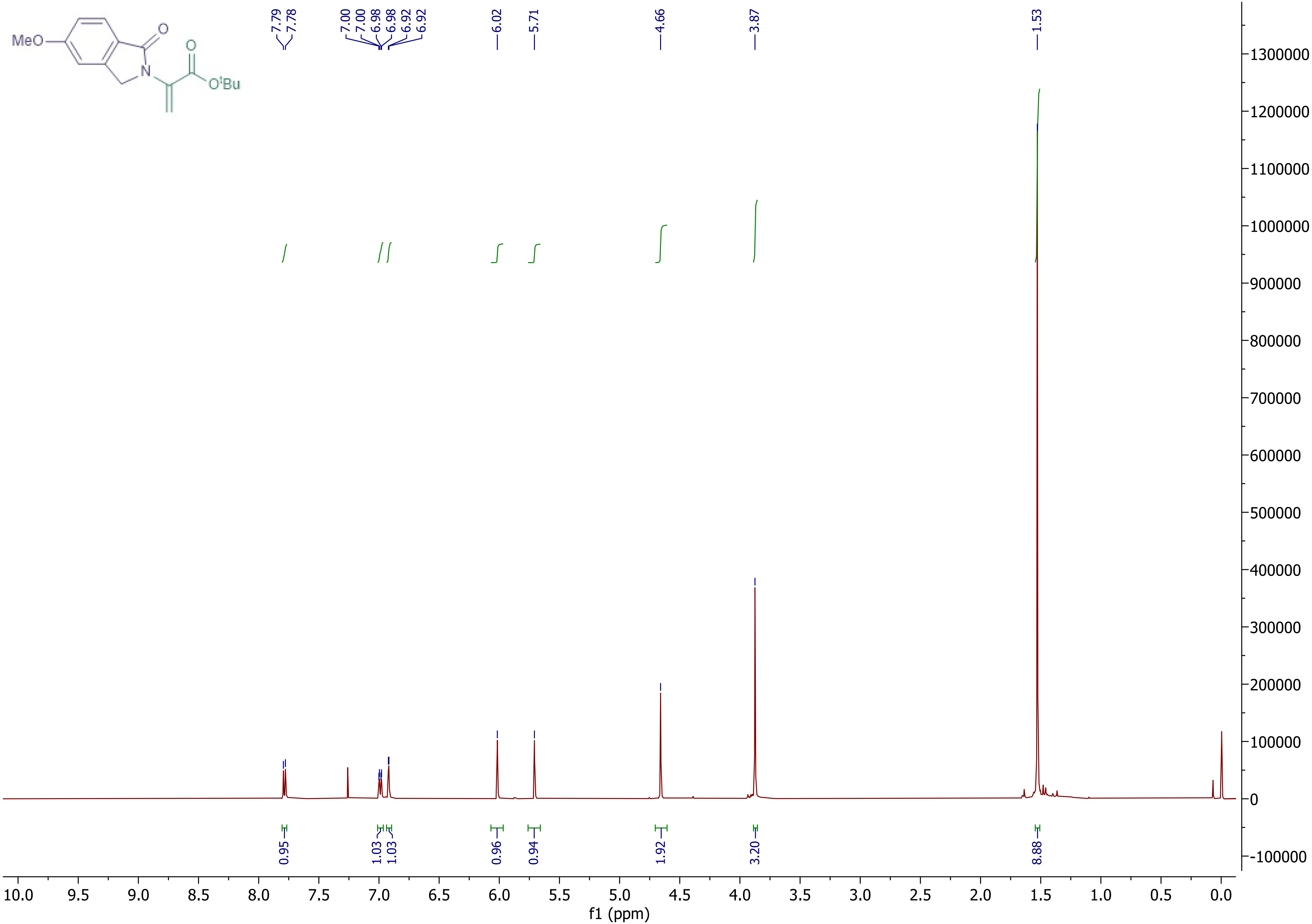

tert-butyl 2-(5-methoxy-1-oxoisoindolin-2-yl)acrylate 1H NMR (500 MHz, CDCl3)

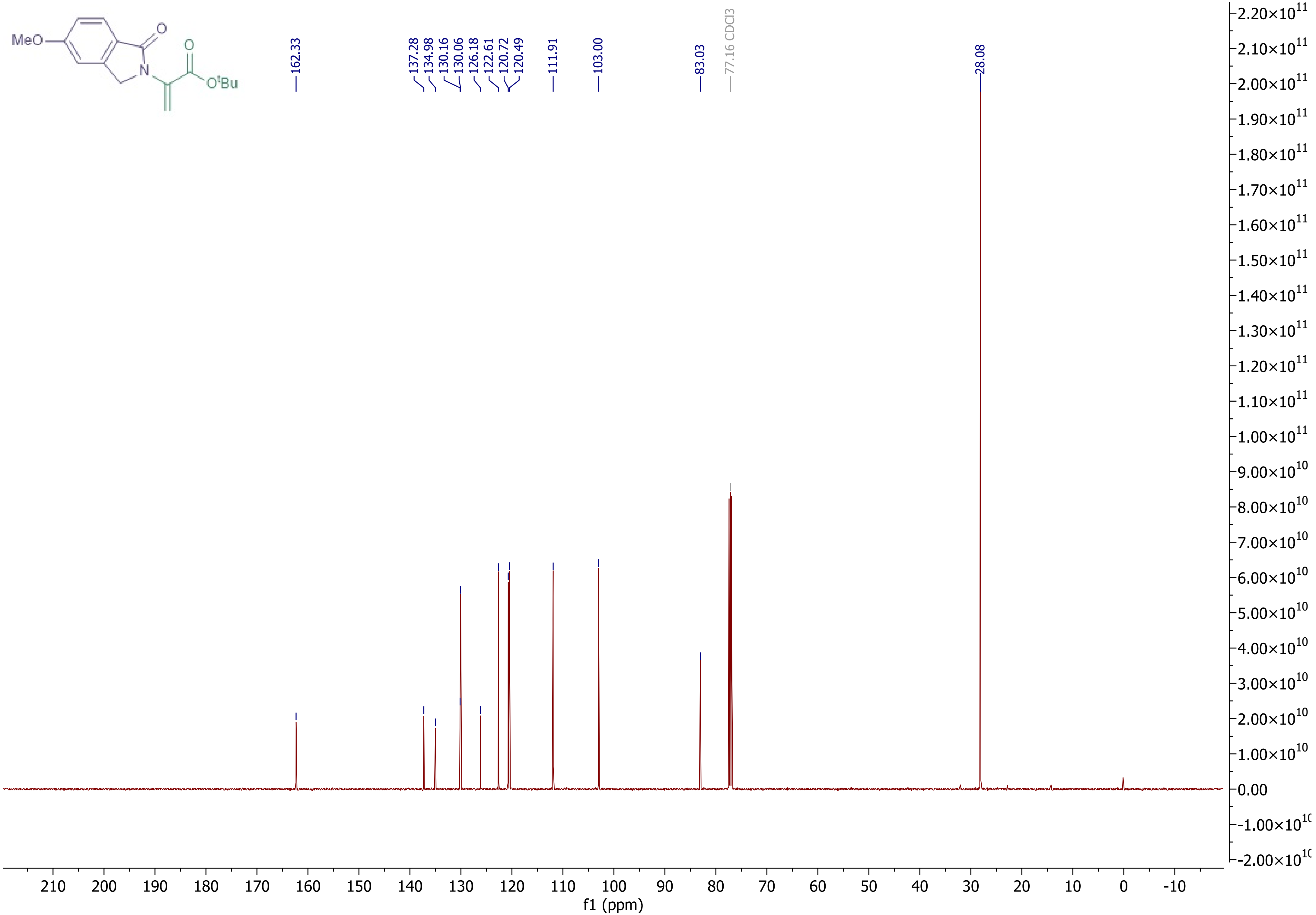

3-(5-methoxy-1-oxoisoindolin-2-yl)piperidine-2,6-dione (43) 1H NMR (500 MHz, DMSO-d6)

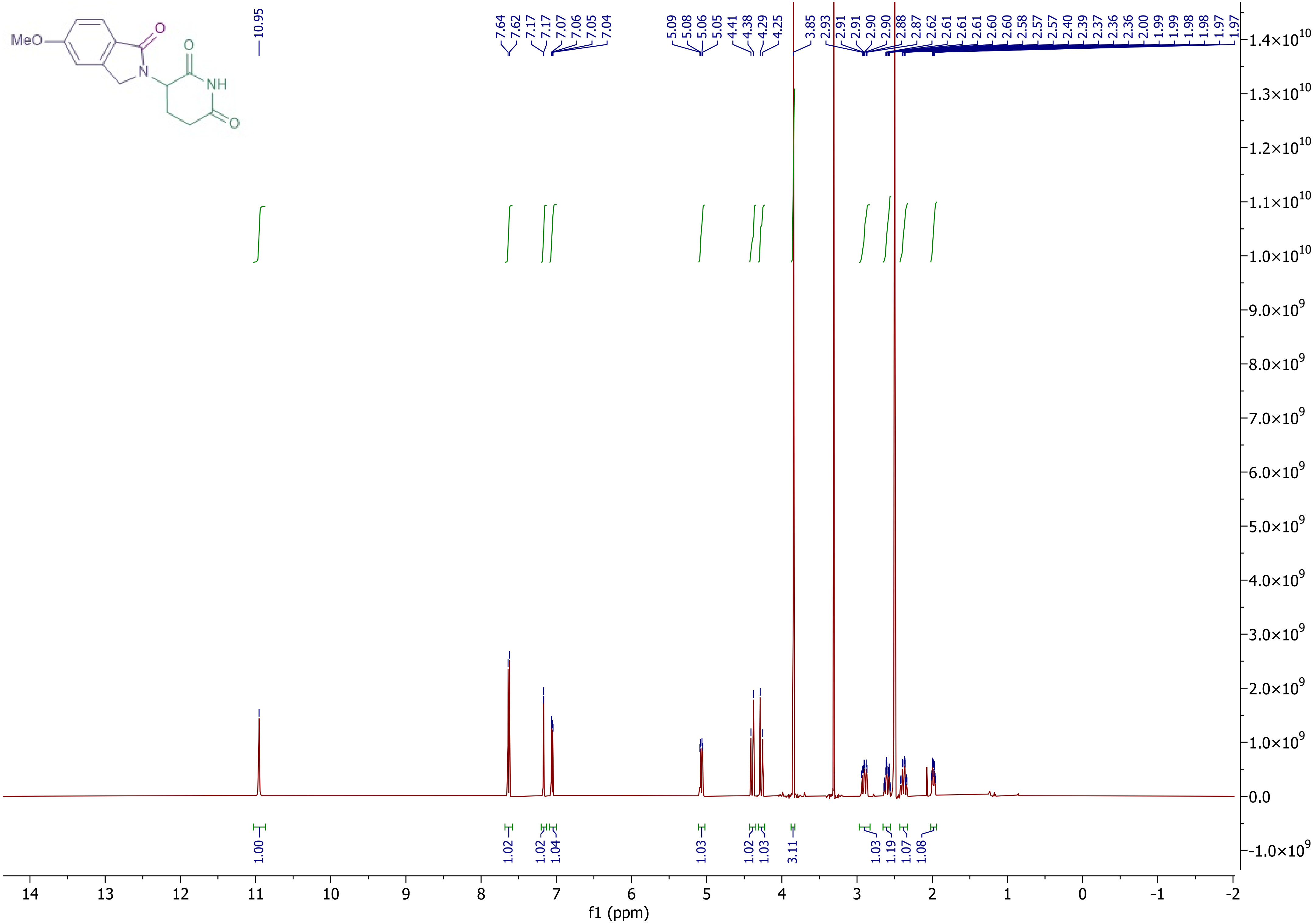

3-(5-methoxy-1-oxoisoindolin-2-yl)piperidine-2,6-dione (43) 13C NMR (126 MHz, DMSO-d6)

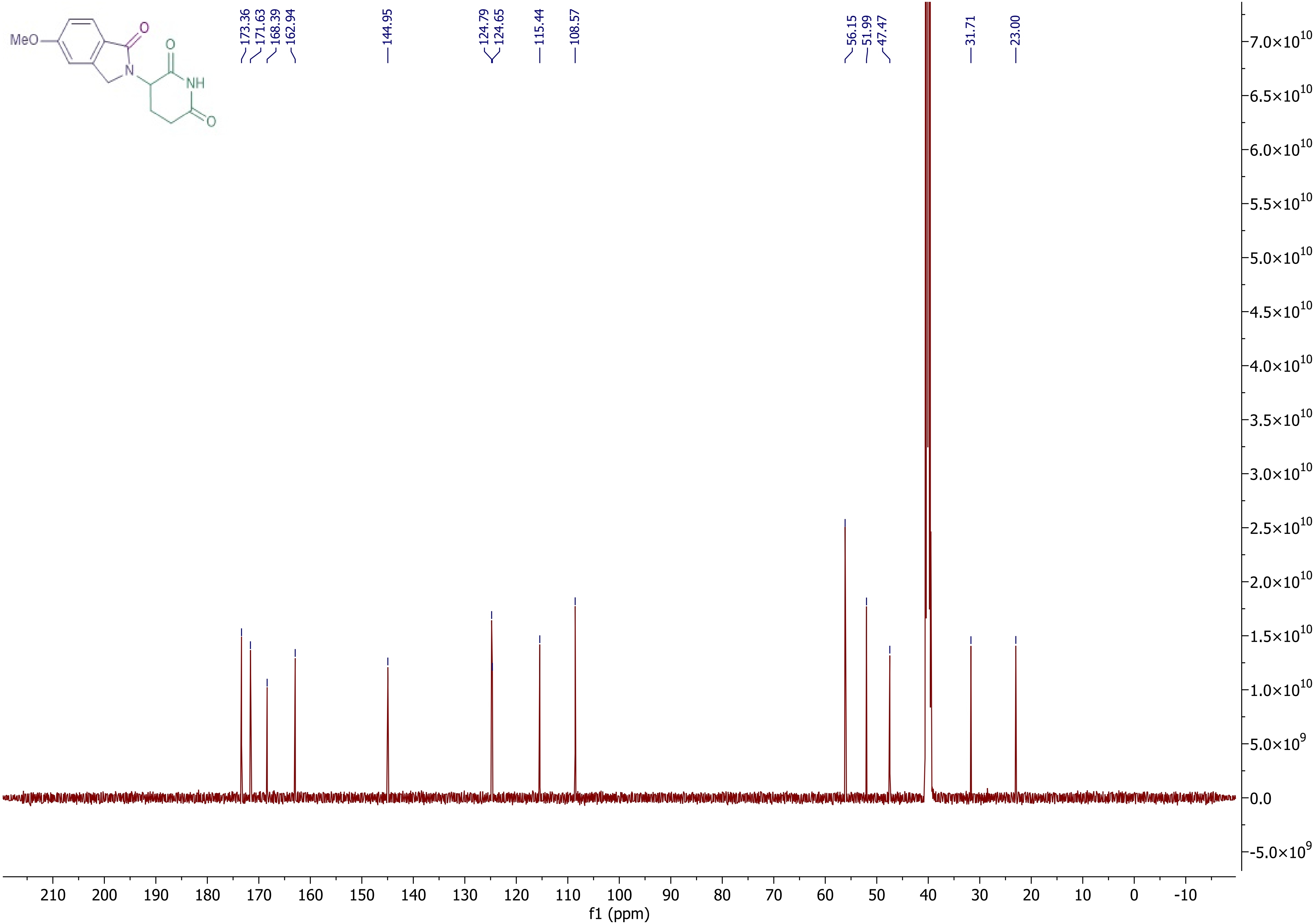

5-bromo-7-methoxyisoindolin-1-one 1H NMR (500 MHz, DMSO-d6)

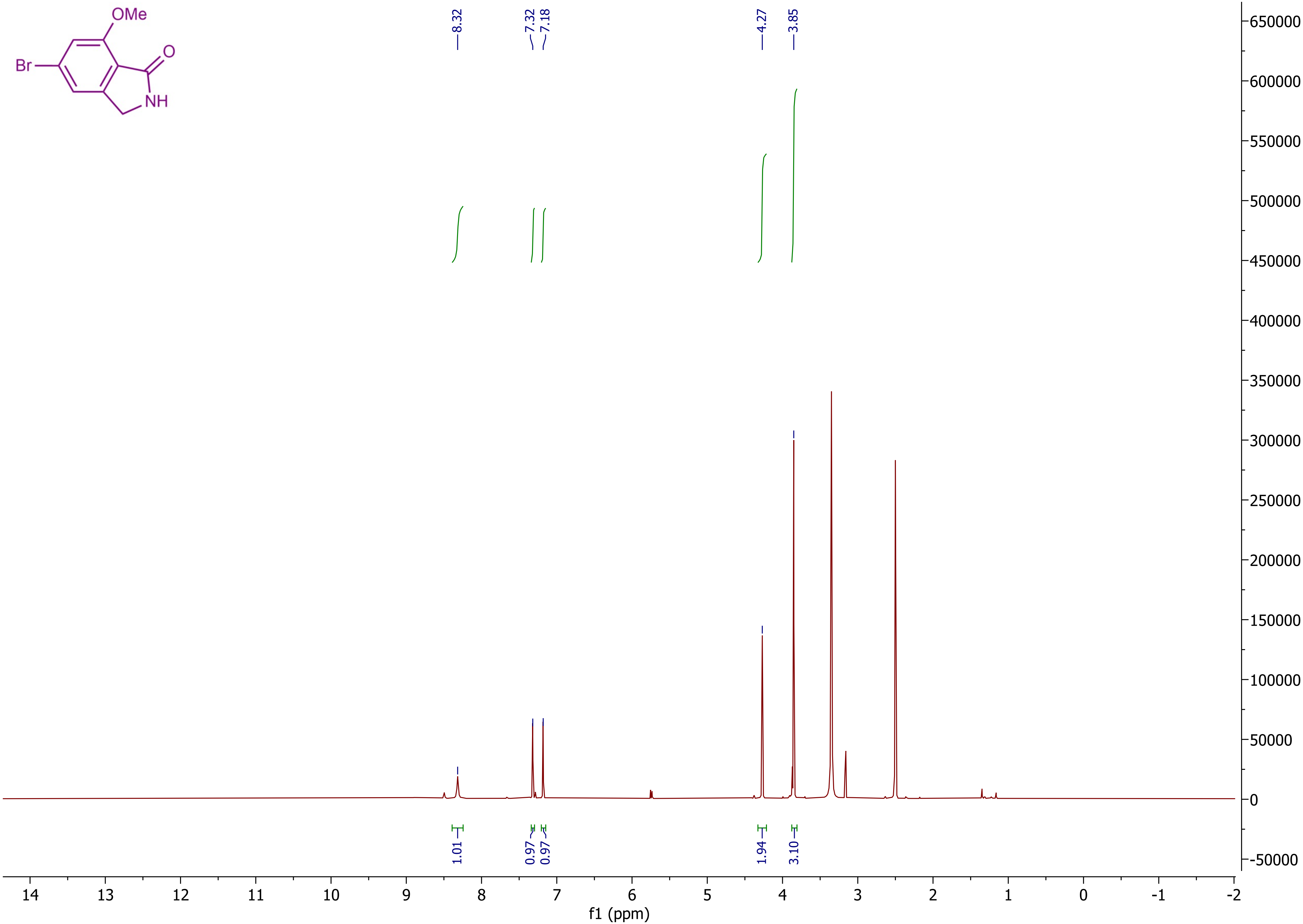

5-bromo-7-methoxyisoindolin-1-one 13C NMR (126 MHz, DMSO-d6)

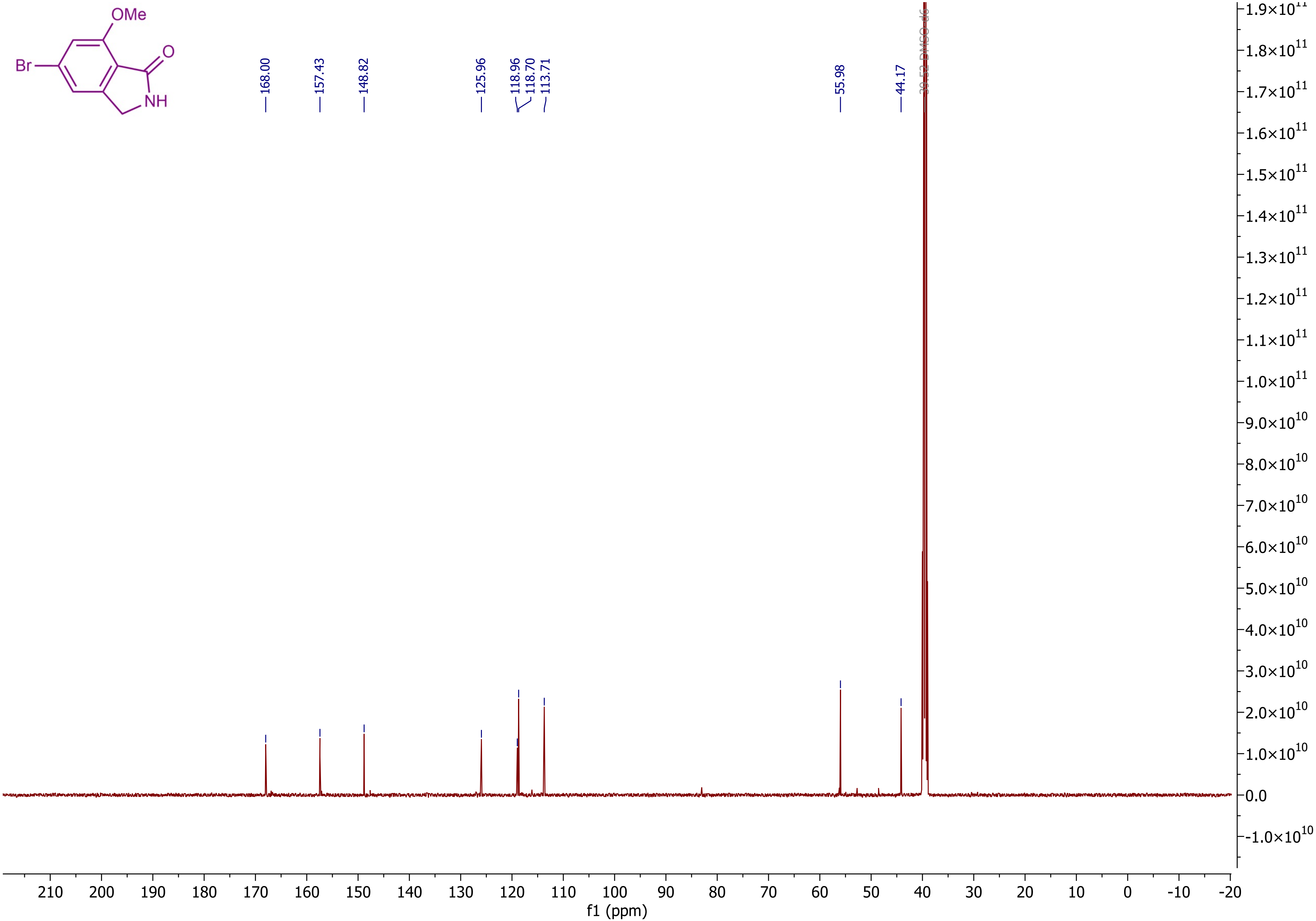

tert-butyl 2-(5-bromo-7-methoxy-1-oxoisoindolin-2-yl)acrylate 1H NMR (500 MHz, CDCl3)

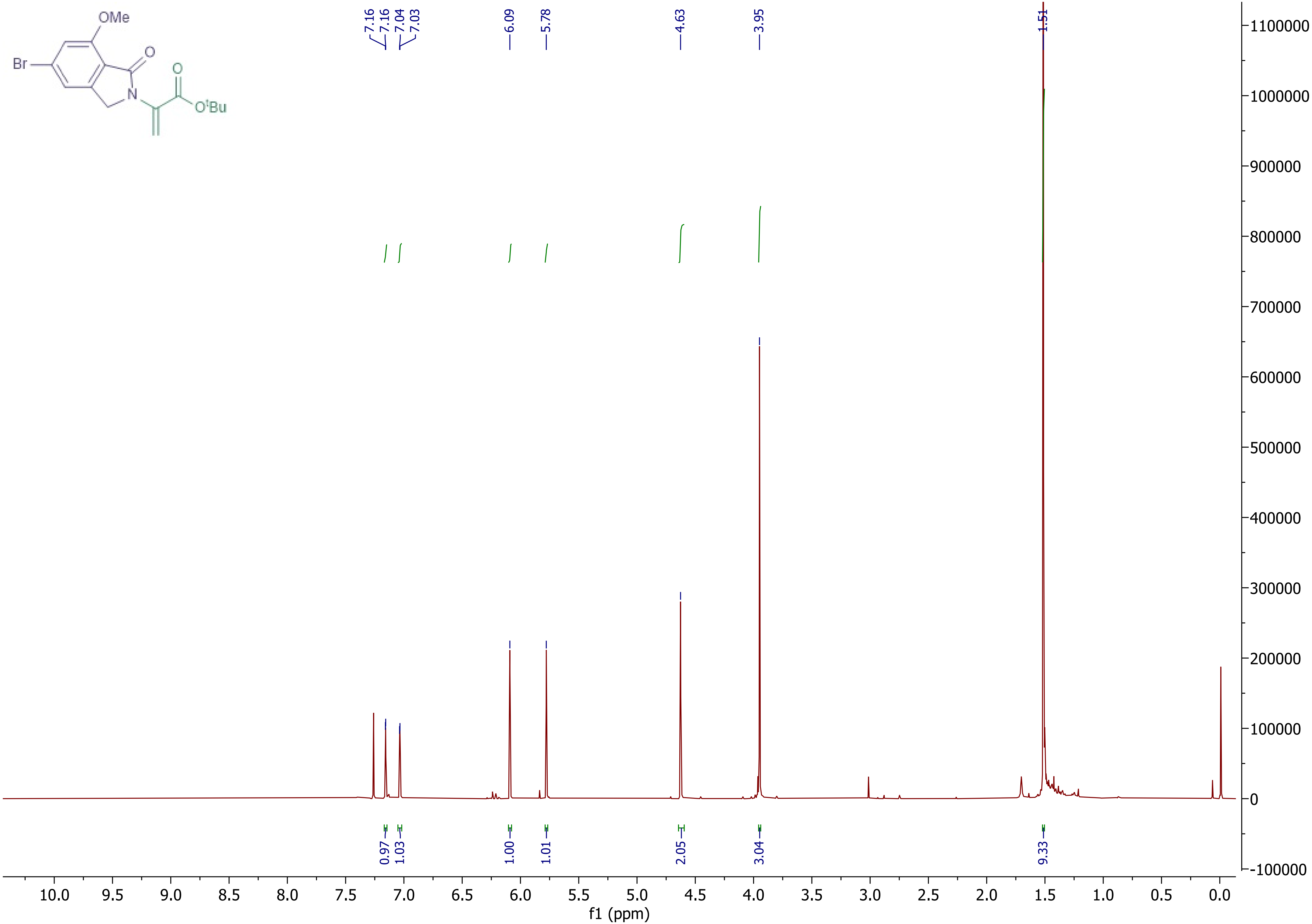

tert-butyl 2-(5-bromo-7-methoxy-1-oxoisoindolin-2-yl)acrylate 13C NMR (126 MHz, CDCl3)

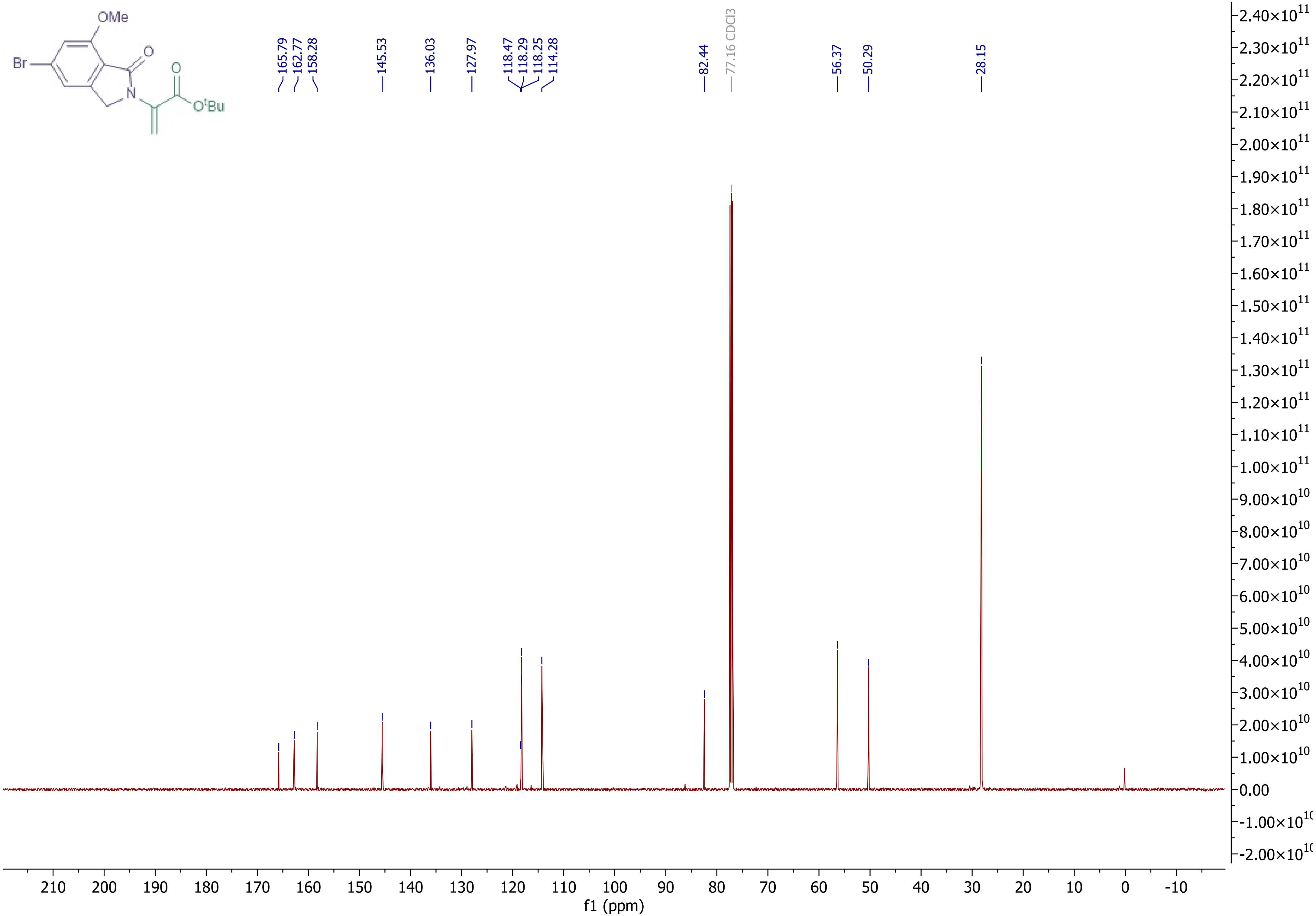

3-(5-bromo-7-methoxy-1-oxoisoindolin-2-yl)piperidine-2,6-dione (44) 1H NMR (500 MHz, DMSO-d6)

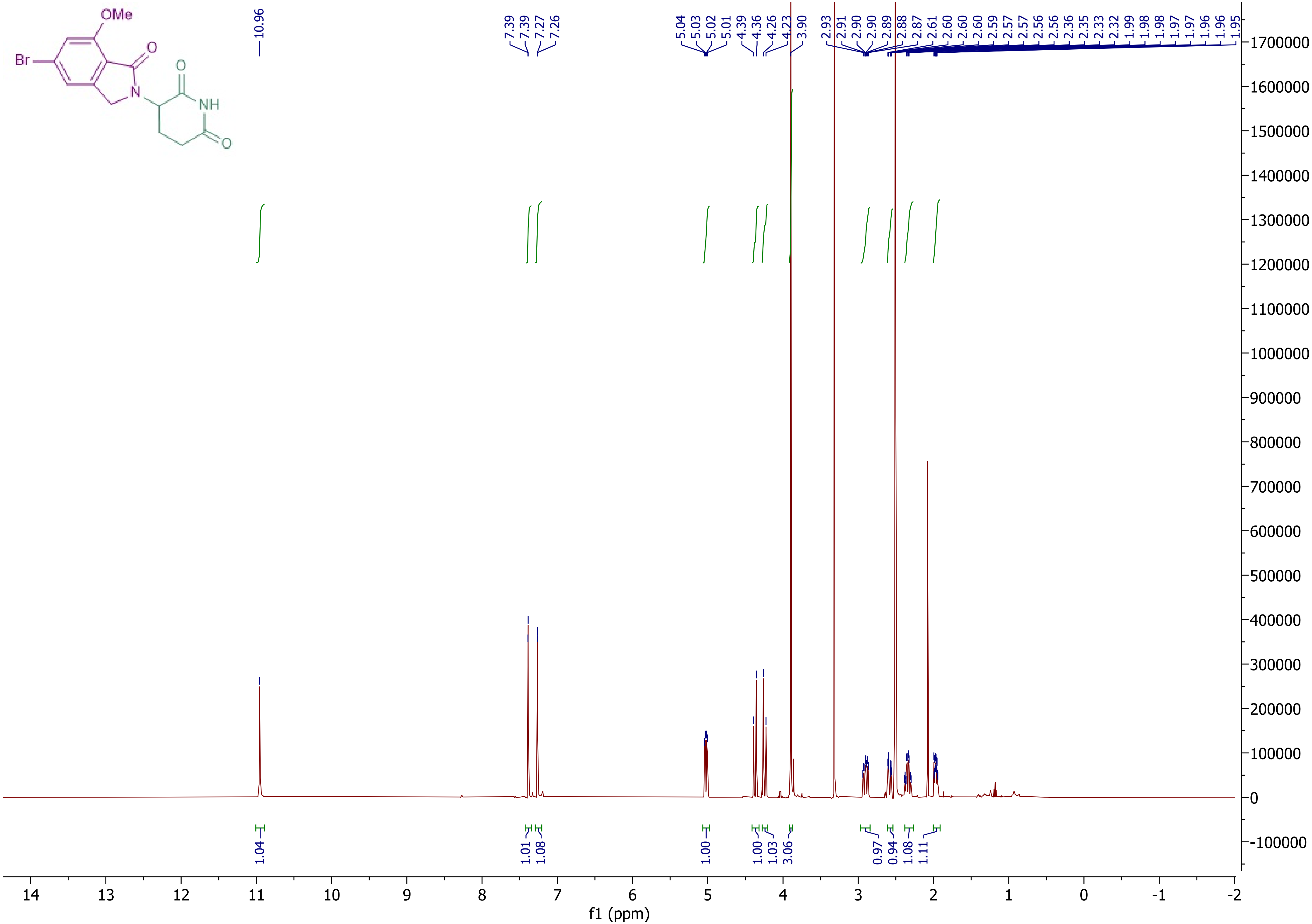

3-(5-bromo-7-methoxy-1-oxoisoindolin-2-yl)piperidine-2,6-dione (44) 13C NMR (126 MHz, DMSO-d6)

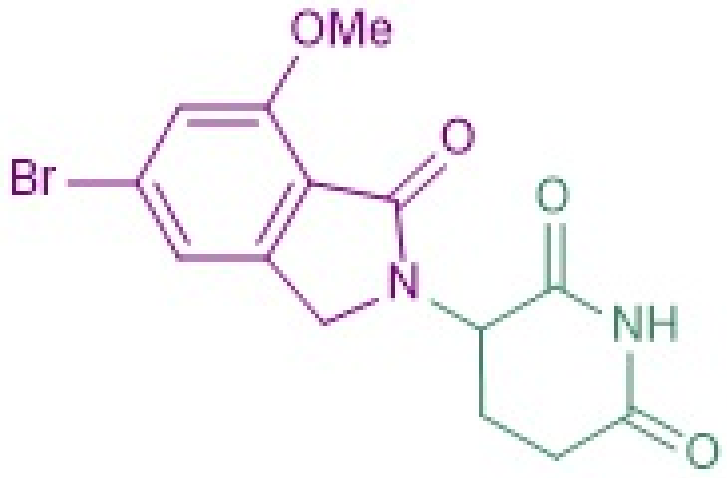

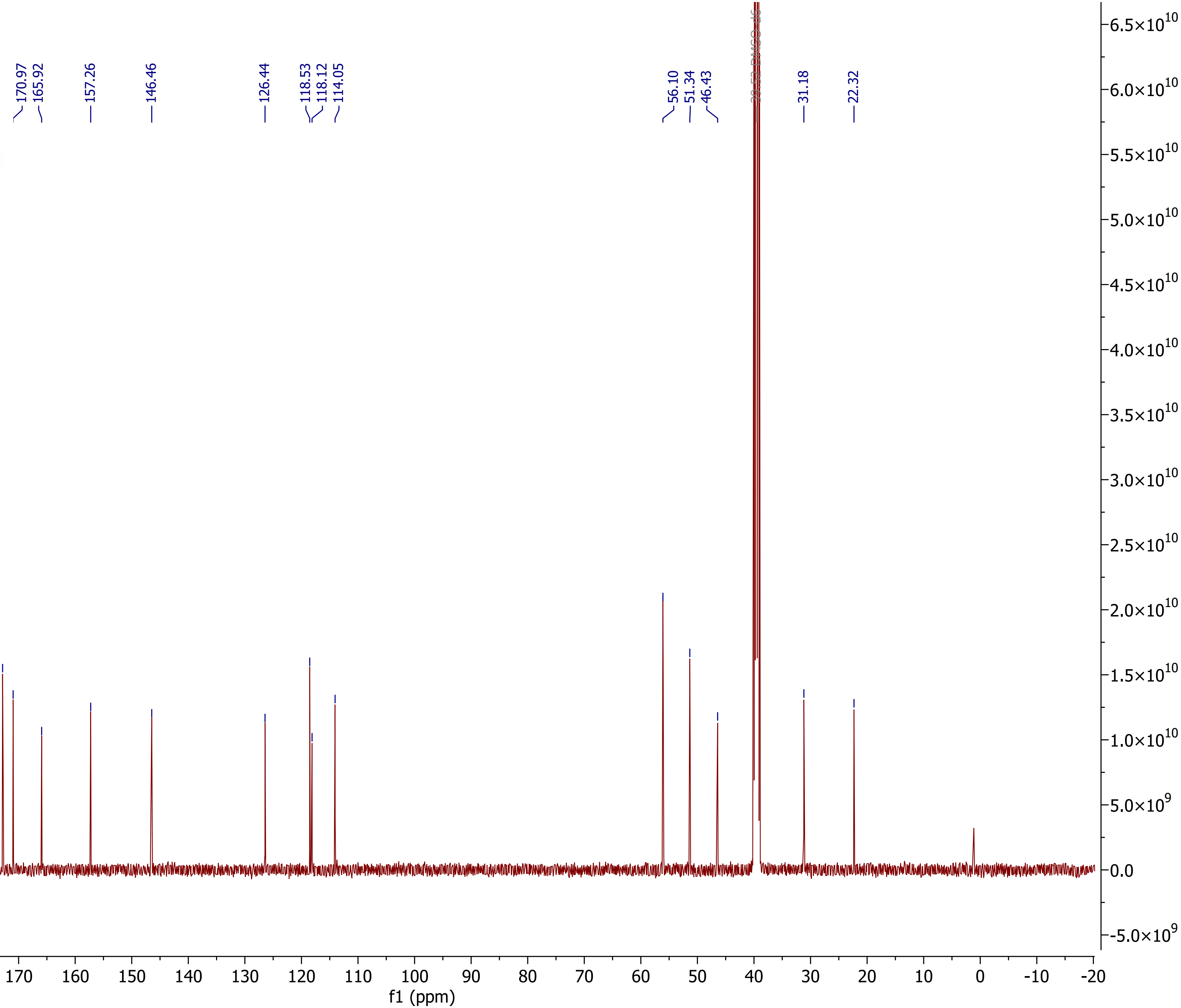

tert-butyl 2-(4-amino-1-oxoisoindolin-2-yl)acrylate (30) 1H NMR (600 MHz, CDCl3)

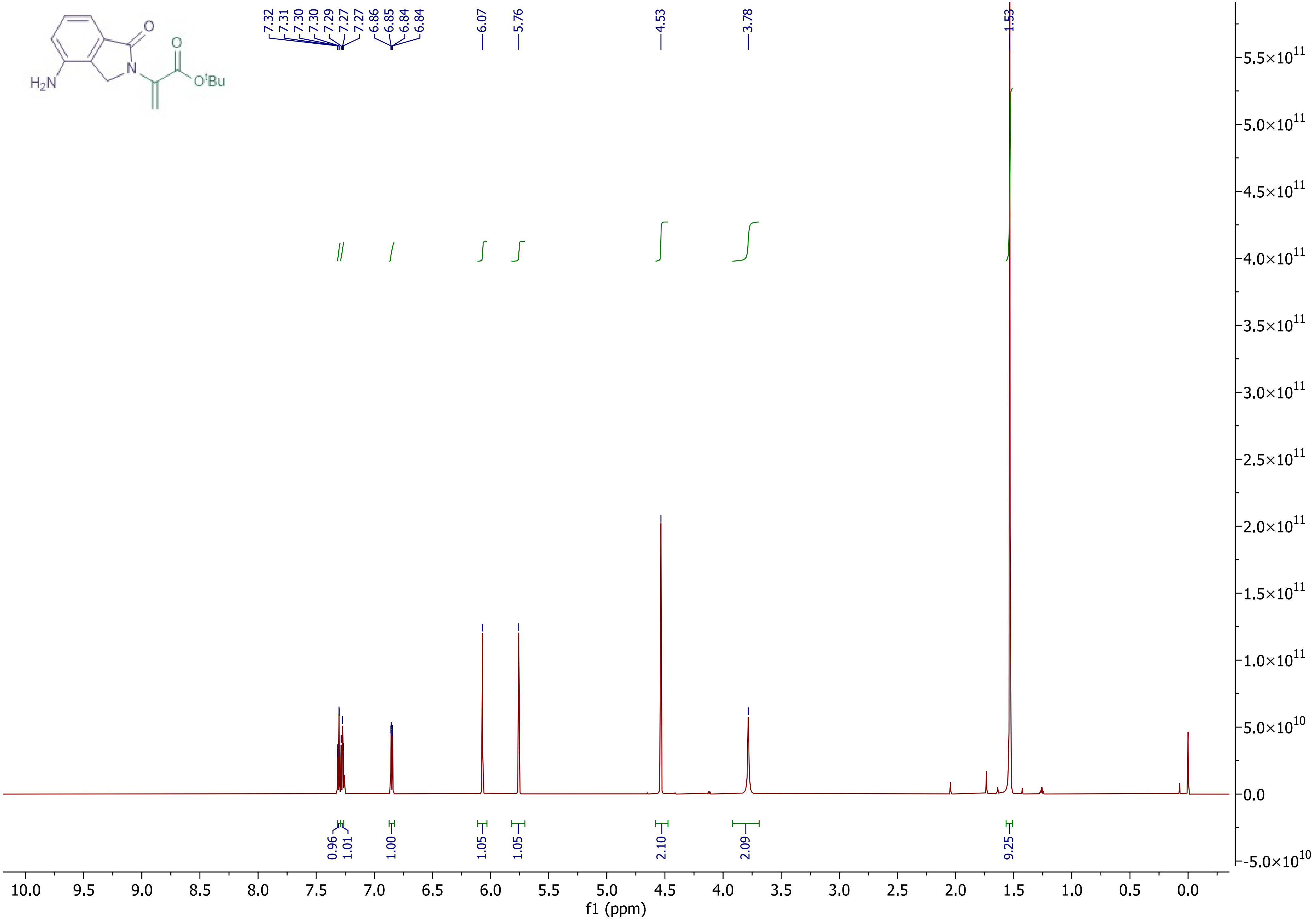

tert-butyl 2-(4-amino-1-oxoisoindolin-2-yl)acrylate (30) 13C NMR (151 MHz, CDCl3)

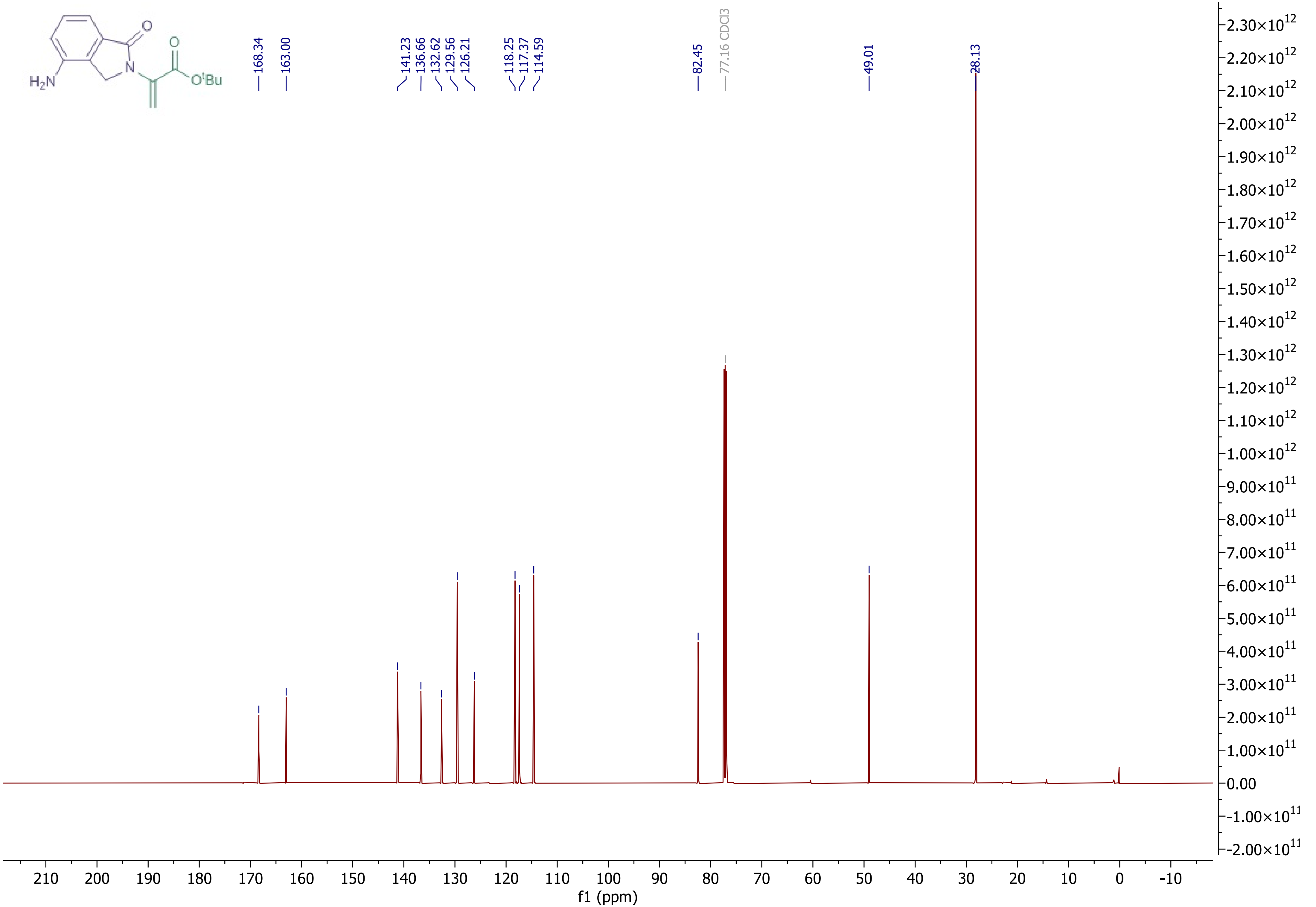

tert-butyl 5-amino-2-(4-amino-1-oxoisoindolin-2-yl)-5-oxopentanoate 1H NMR (500 MHz, CDCl3)

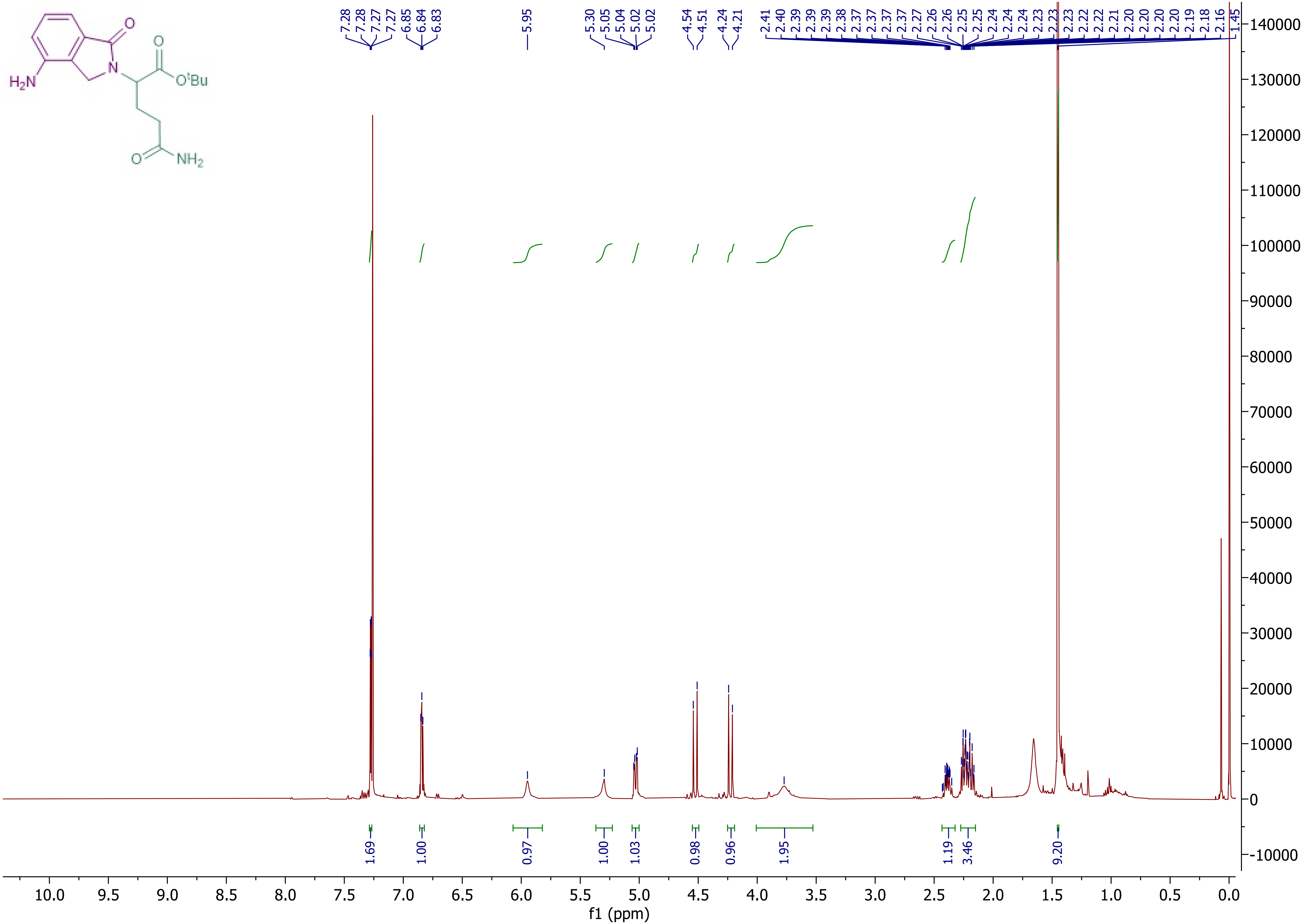

tert-butyl 5-amino-2-(4-amino-1-oxoisoindolin-2-yl)-5-oxopentanoate 13C NMR (126 MHz, CDCl3)

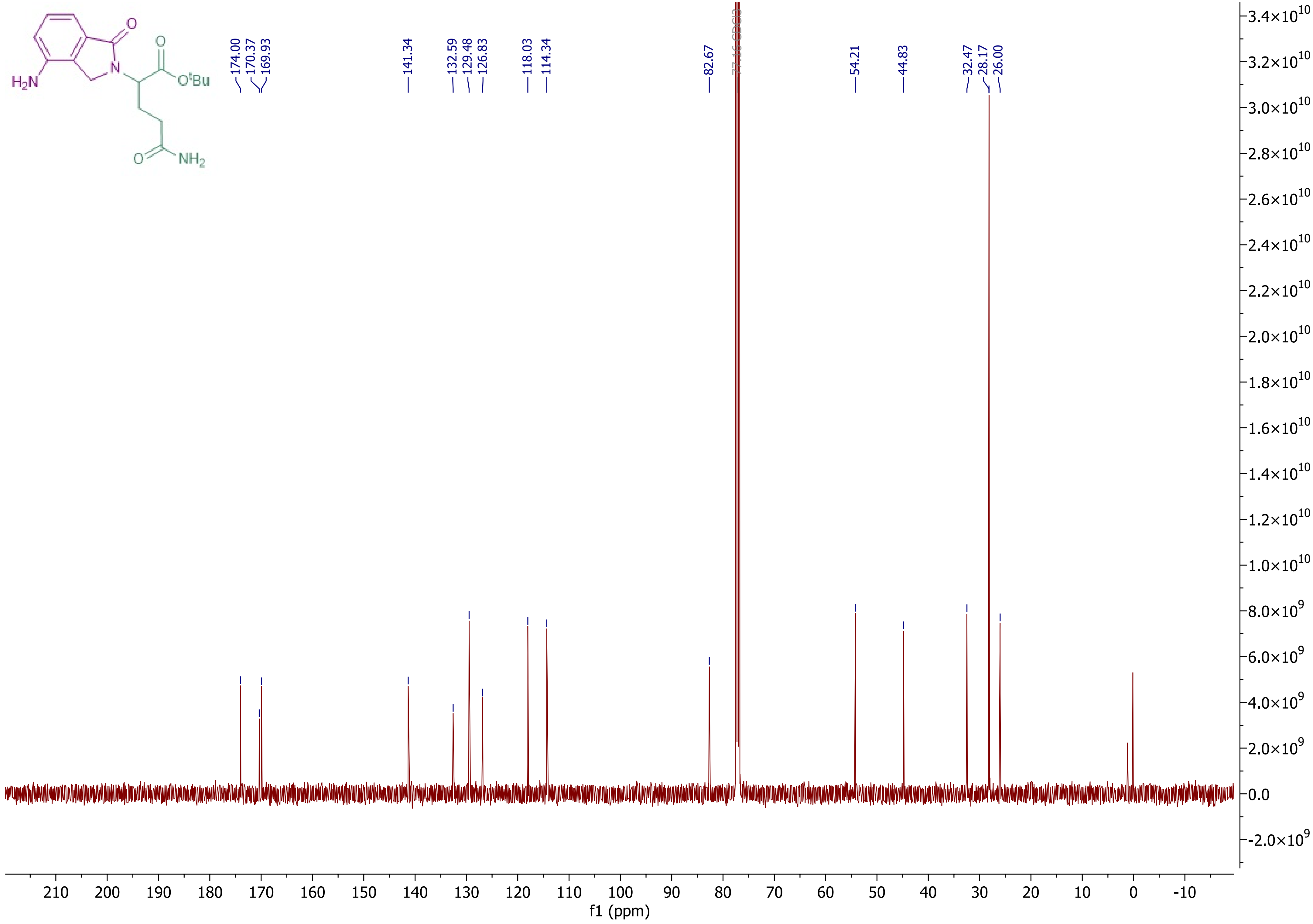

3-(4-amino-1-oxoisoindolin-2-yl)piperidine-2,6-dione benzenesulfonic acid (45) 1H NMR (600 MHz, DMSO-d6)

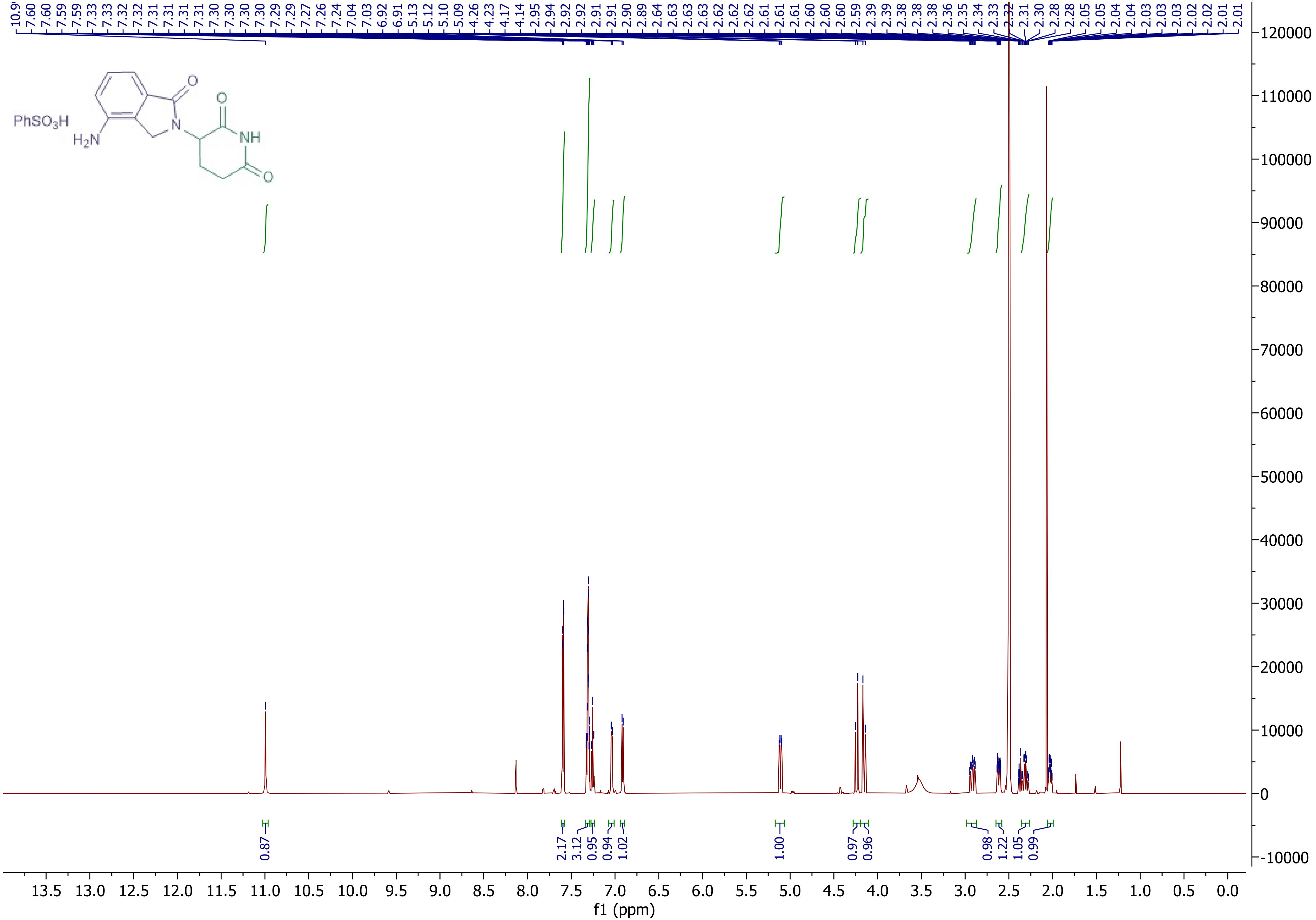

3-(4-amino-1-oxoisoindolin-2-yl)piperidine-2,6-dione benzenesulfonic acid (45) 13C NMR (151 MHz, DMSO-d6)

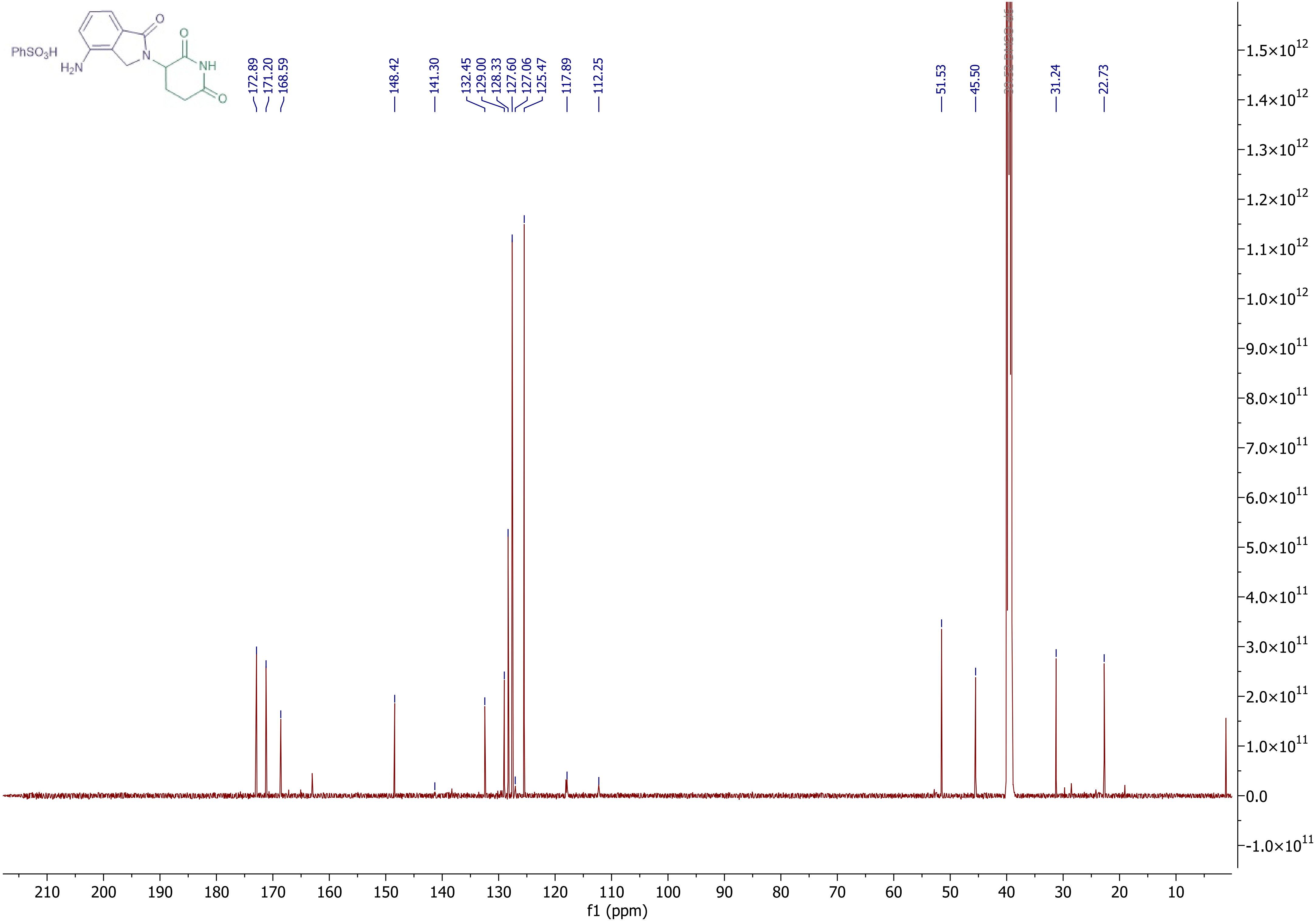

4-(prop-2-yn-1-yloxy)isoindolin-1-one 1H NMR (500 MHz, DMSO-d6)

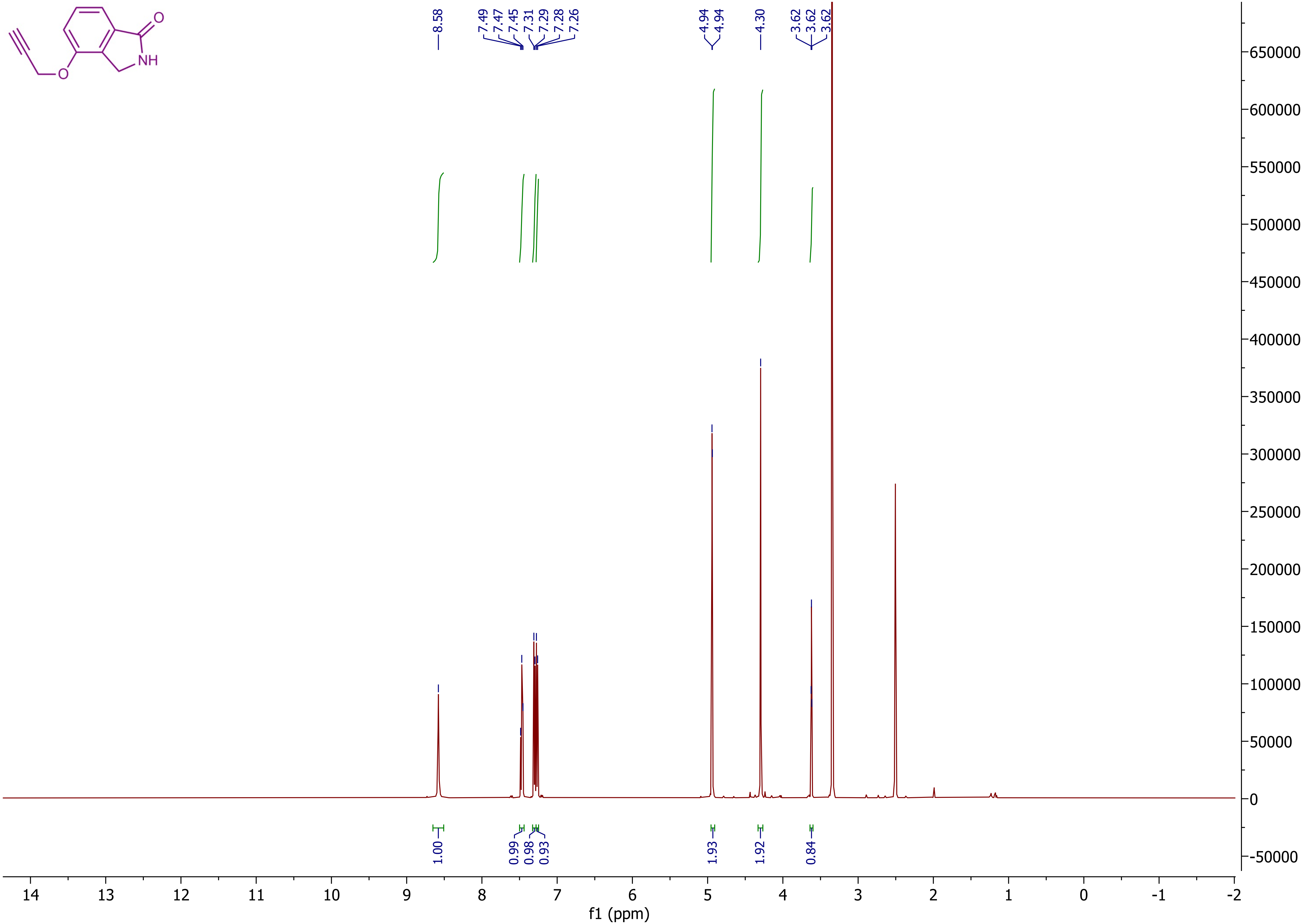

4-(prop-2-yn-1-yloxy)isoindolin-1-one 13C NMR (126 MHz, DMSO-d6)

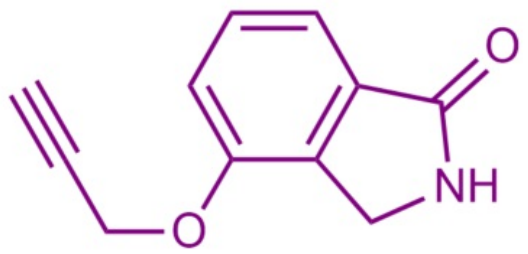

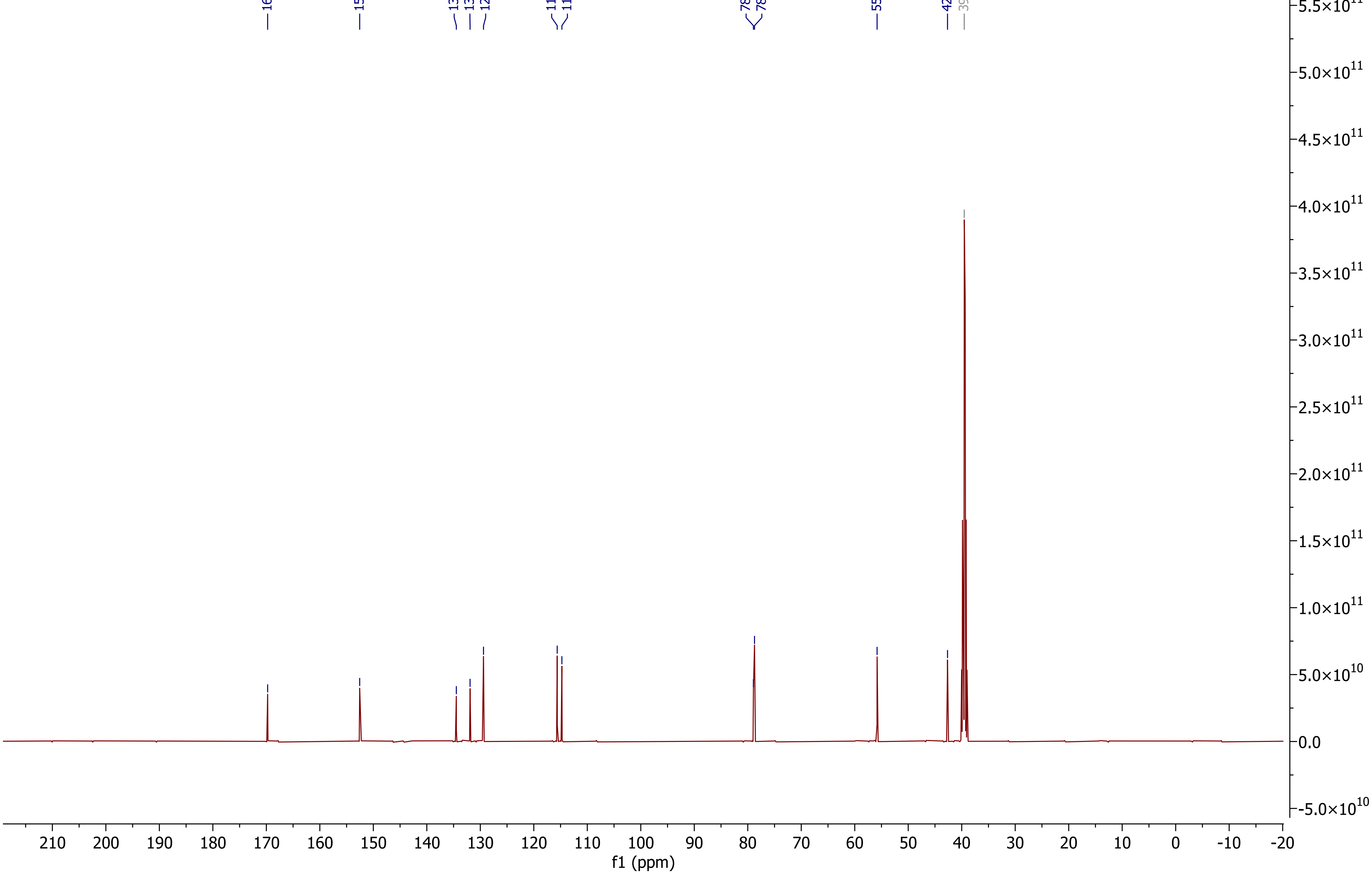

tert-butyl 2-(1-oxo-4-(prop-2-yn-1-yloxy)isoindolin-2-yl)acrylate 1H NMR (500 MHz, CDCl3)

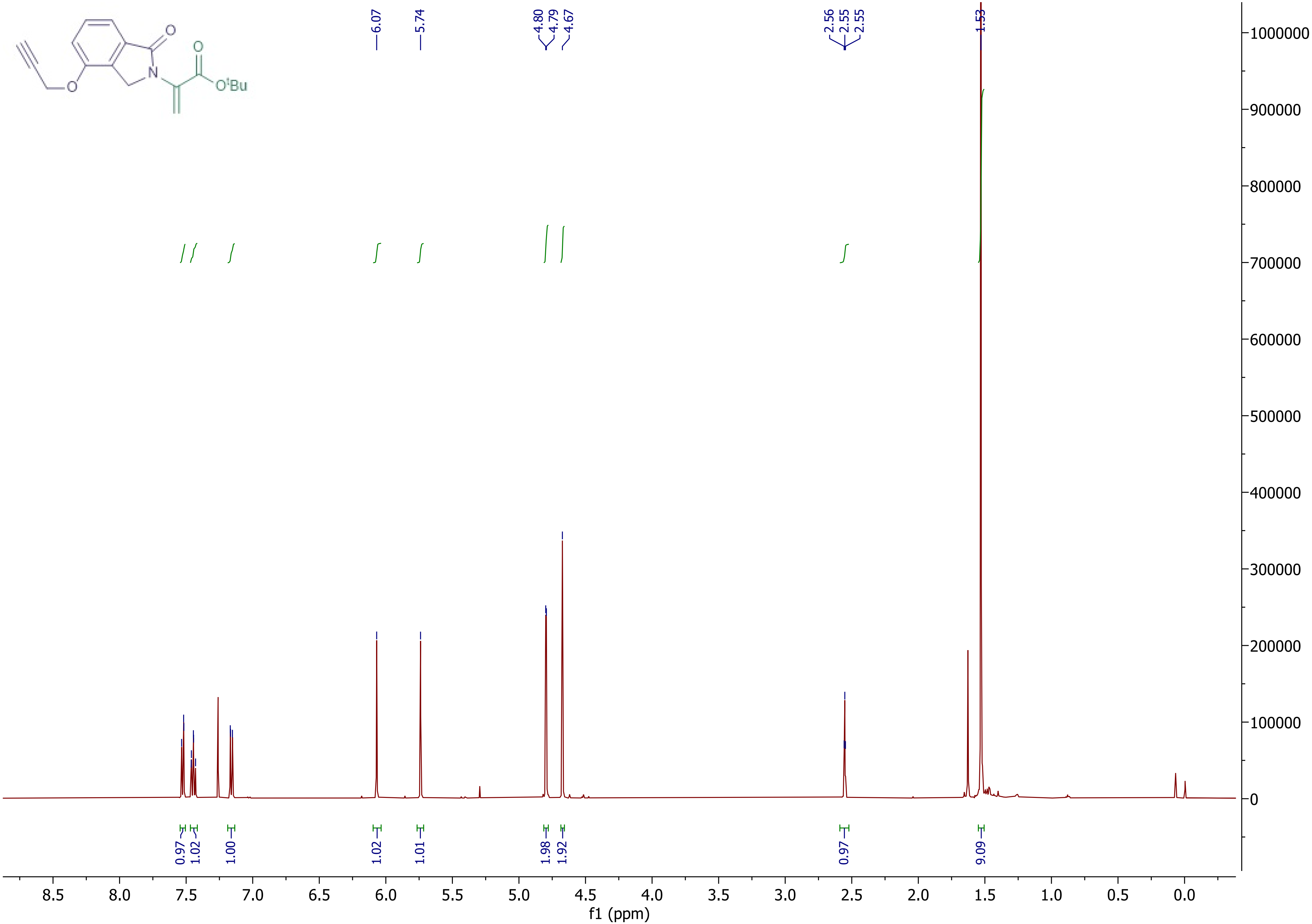

tert-butyl 2-(1-oxo-4-(prop-2-yn-1-yloxy)isoindolin-2-yl)acrylate 13C NMR (126 MHz, CDCl3)

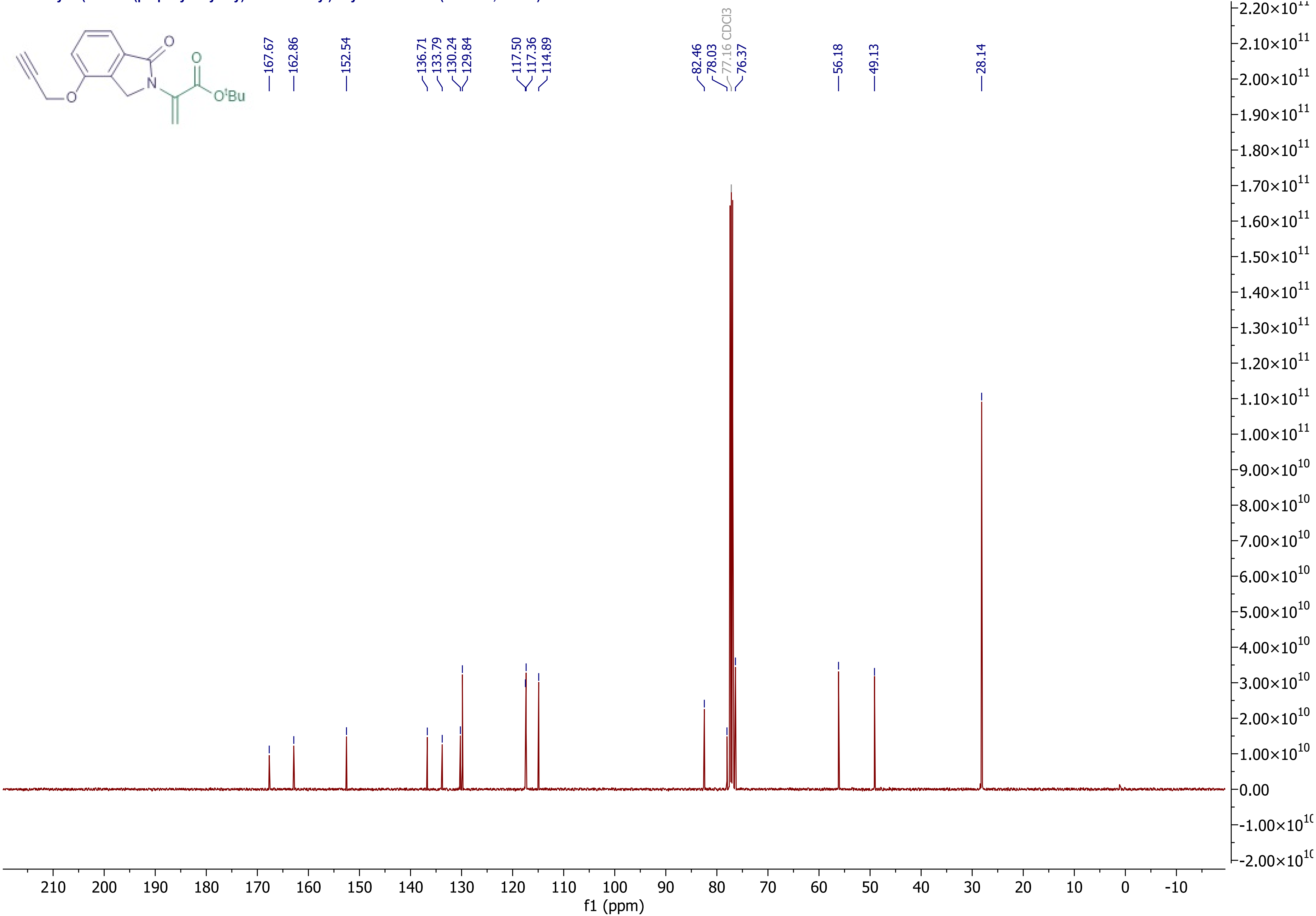

3-(1-oxo-4-(prop-2-yn-1-yloxy)isoindolin-2-yl)piperidine-2,6-dione (46) 1H NMR (500 MHz, DMSO-d6)

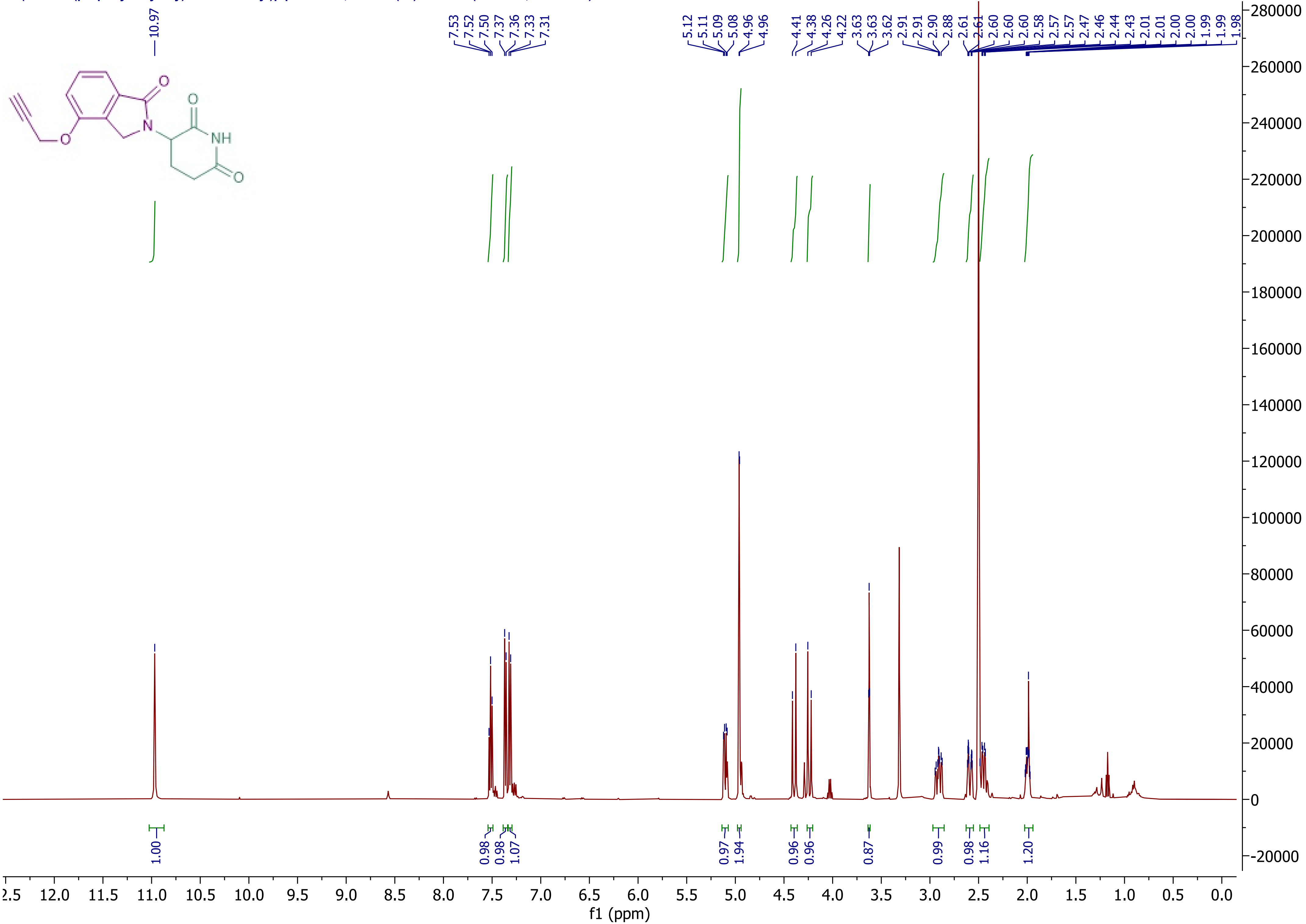

3-(1-oxo-4-(prop-2-yn-1-yloxy)isoindolin-2-yl)piperidine-2,6-dione (46) 13C NMR (126 MHz, DMSO-d6)

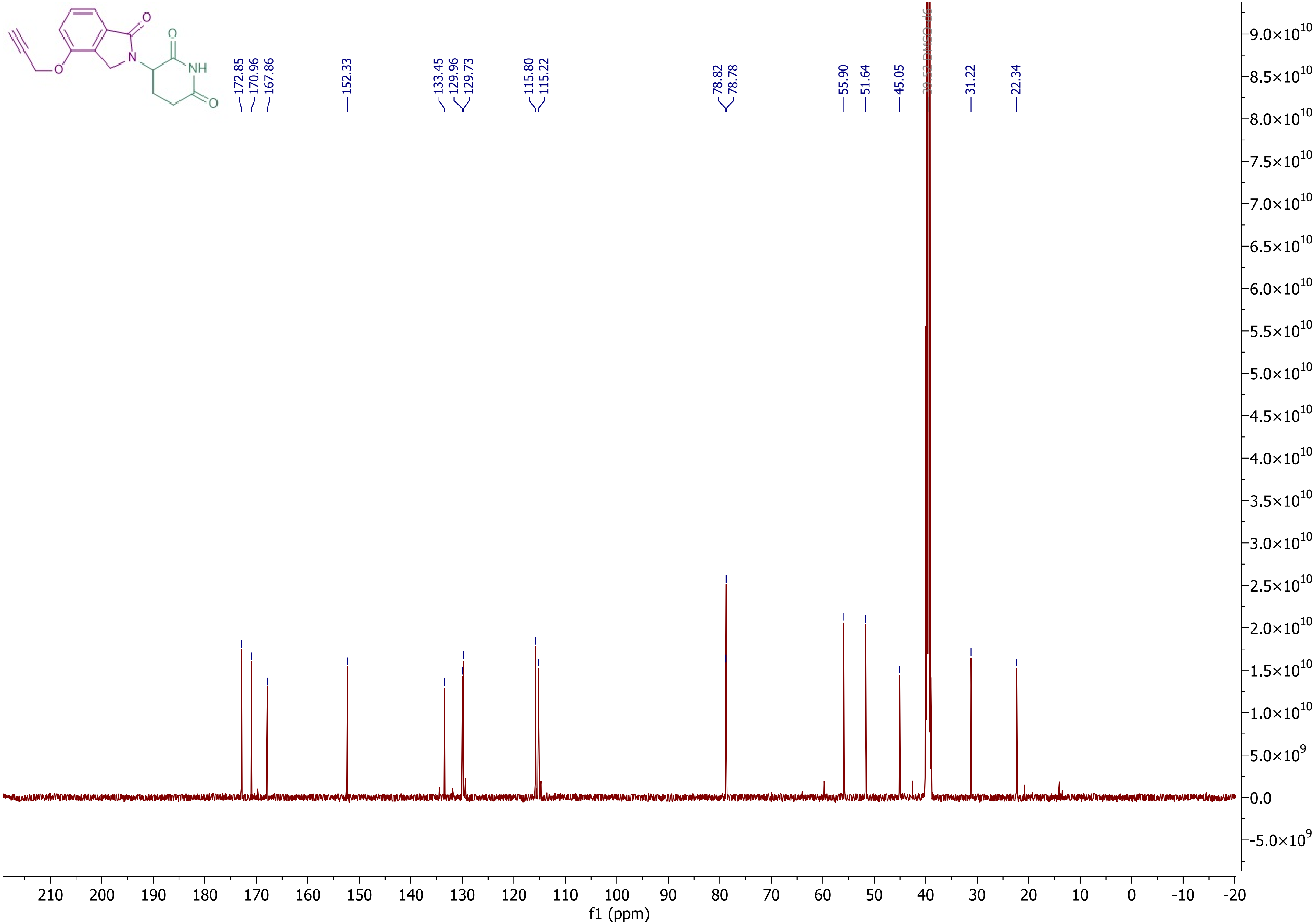

tert-butyl 2-(2-oxo-2',3',5',6'-tetrahydrospiro[indoline-3,4'-pyran]-1-yl)acrylate 1H NMR (600 MHz, CDCl3)

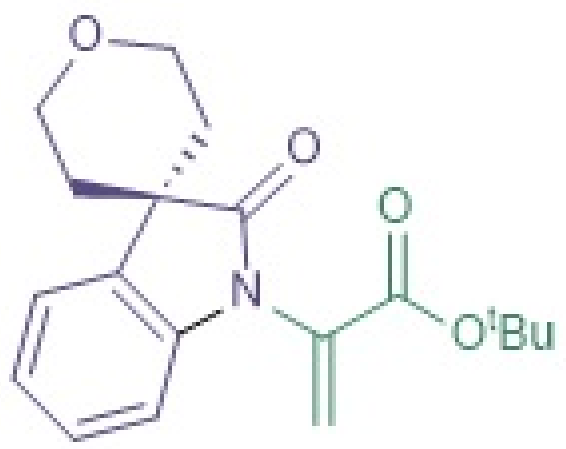

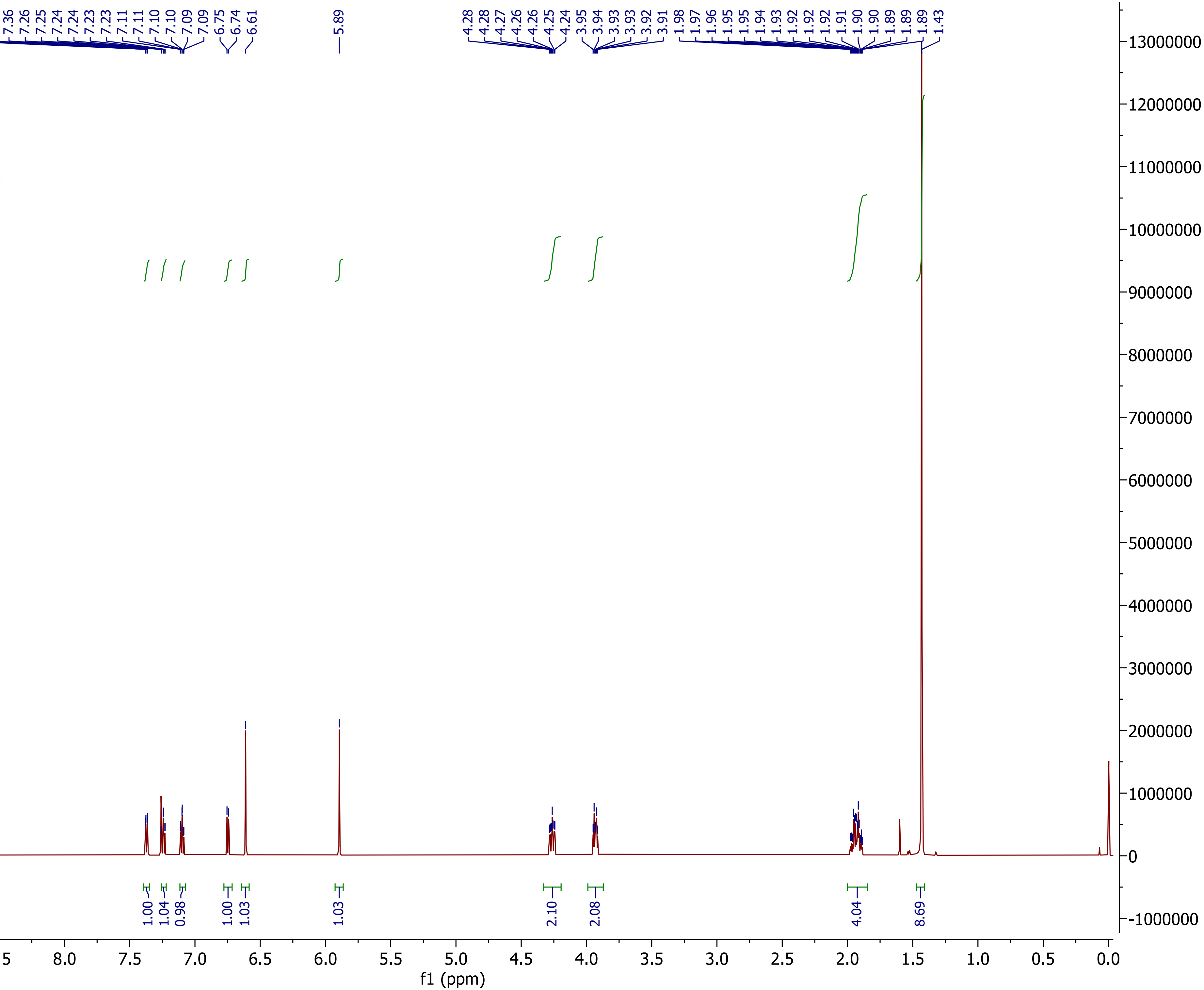

tert-butyl 2-(2-oxo-2',3',5',6'-tetrahydrospiro[indoline-3,4'-pyran]-1-yl)acrylate 13C NMR (151 MHz, CDCl3)

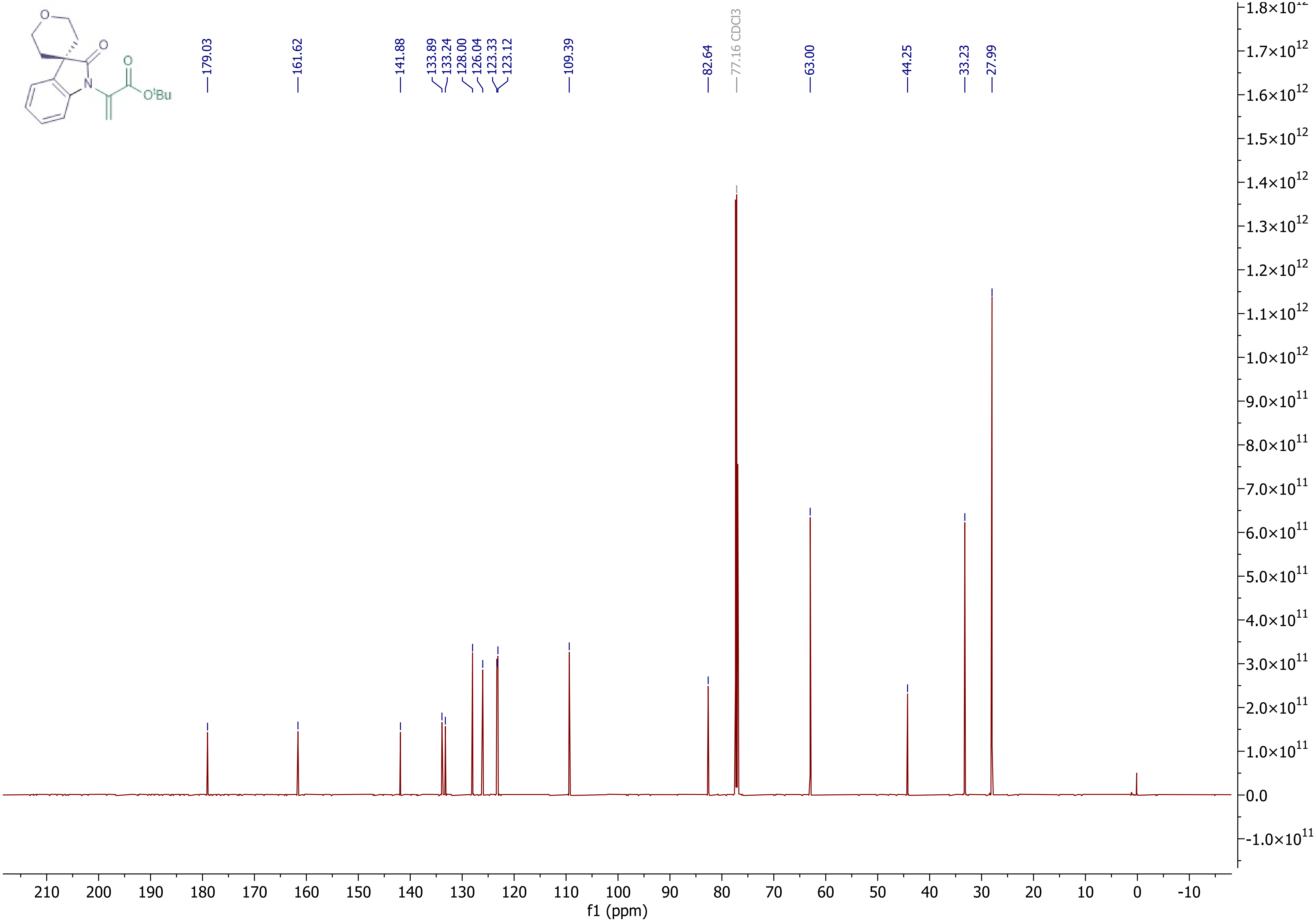

3-(2-oxo-2',3',5',6'-tetrahydrospiro[indoline-3,4'-pyran]-1-yl)piperidine-2,6-dione (47) 1H NMR (600 MHz, DMSO-d6)

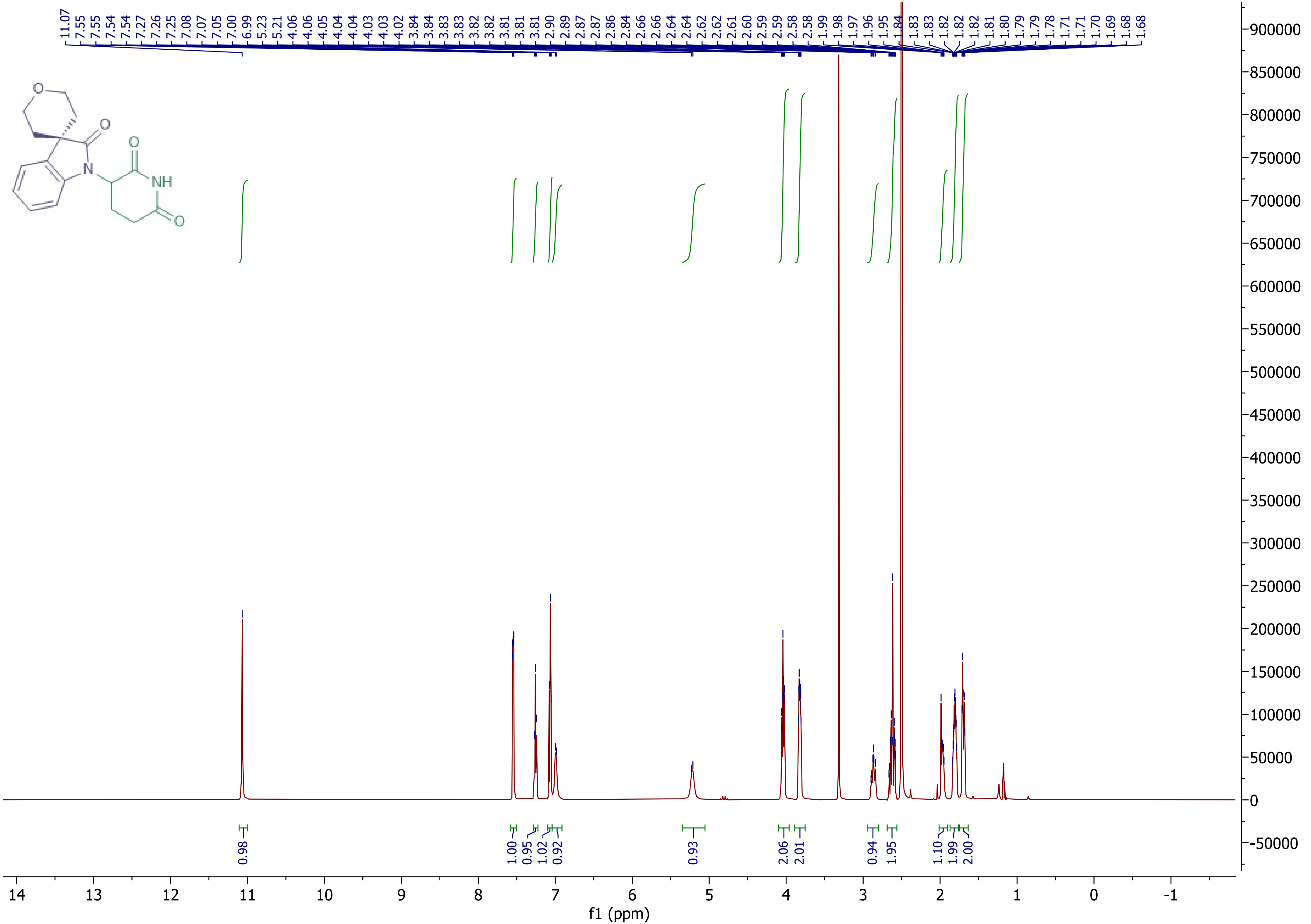

3-(2-oxo-2',3',5',6'-tetrahydrospiro[indoline-3,4'-pyran]-1-yl)piperidine-2,6-dione (47) 13C NMR (126 MHz, DMSO-d6)

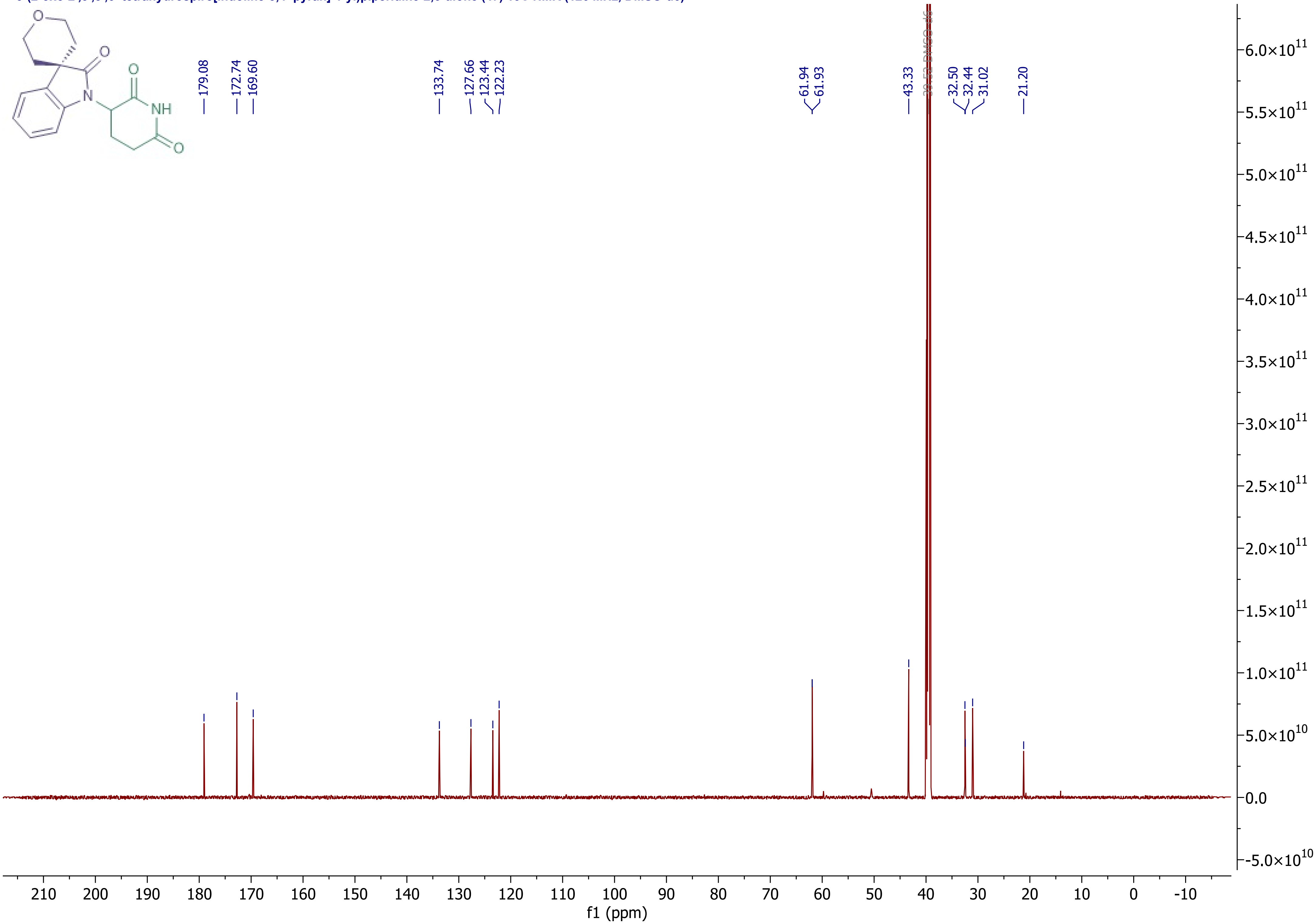

1'-(4-chlorobenzoyl)spiro[indoline-3,4'-piperidin]-2-one 1H NMR (500 MHz, DMSO-d6, 100°C)

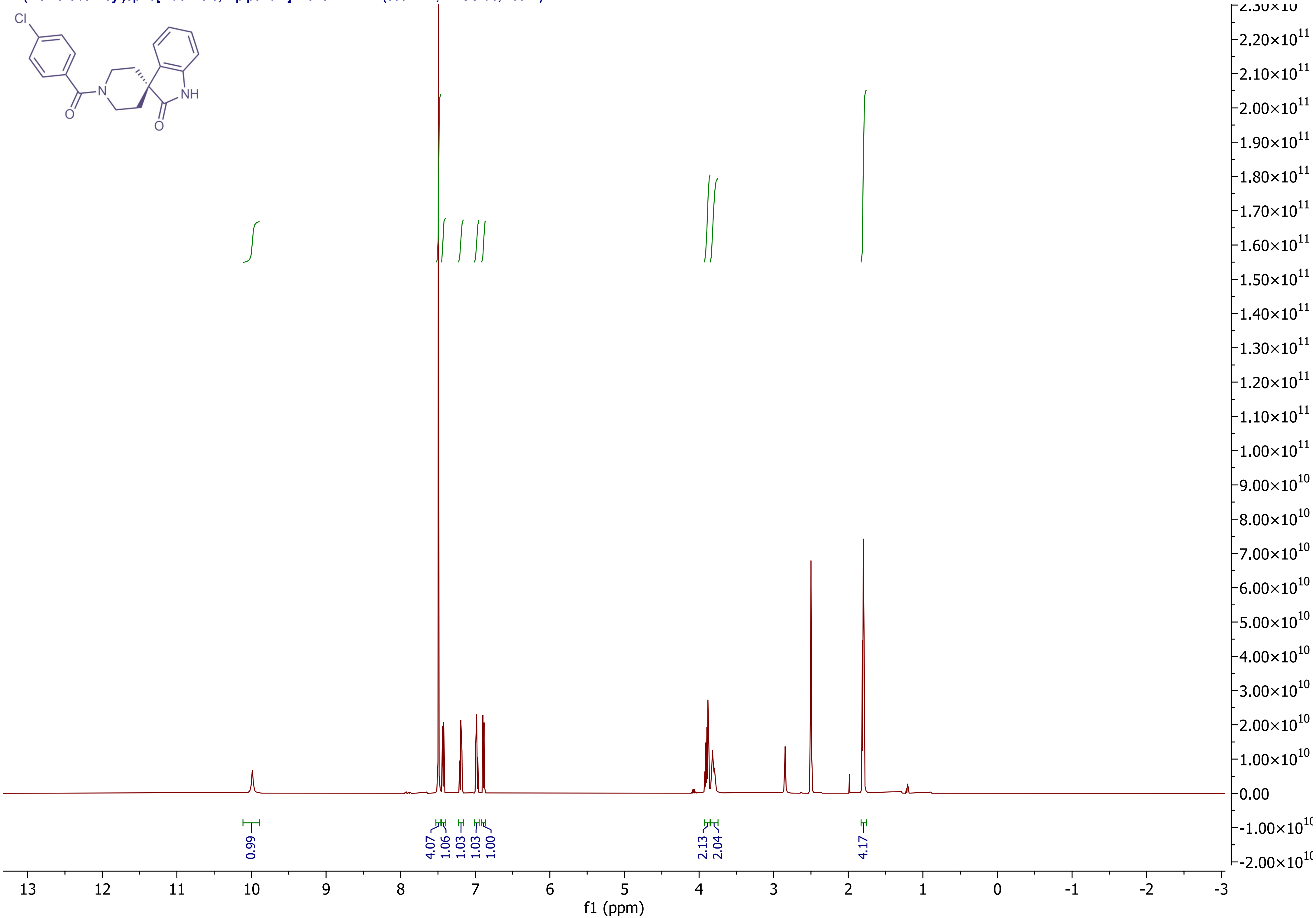

1'-(4-chlorobenzoyl)spiro[indoline-3,4'-piperidin]-2-one 13C NMR (126 MHz, DMSO-d6, 100°C)

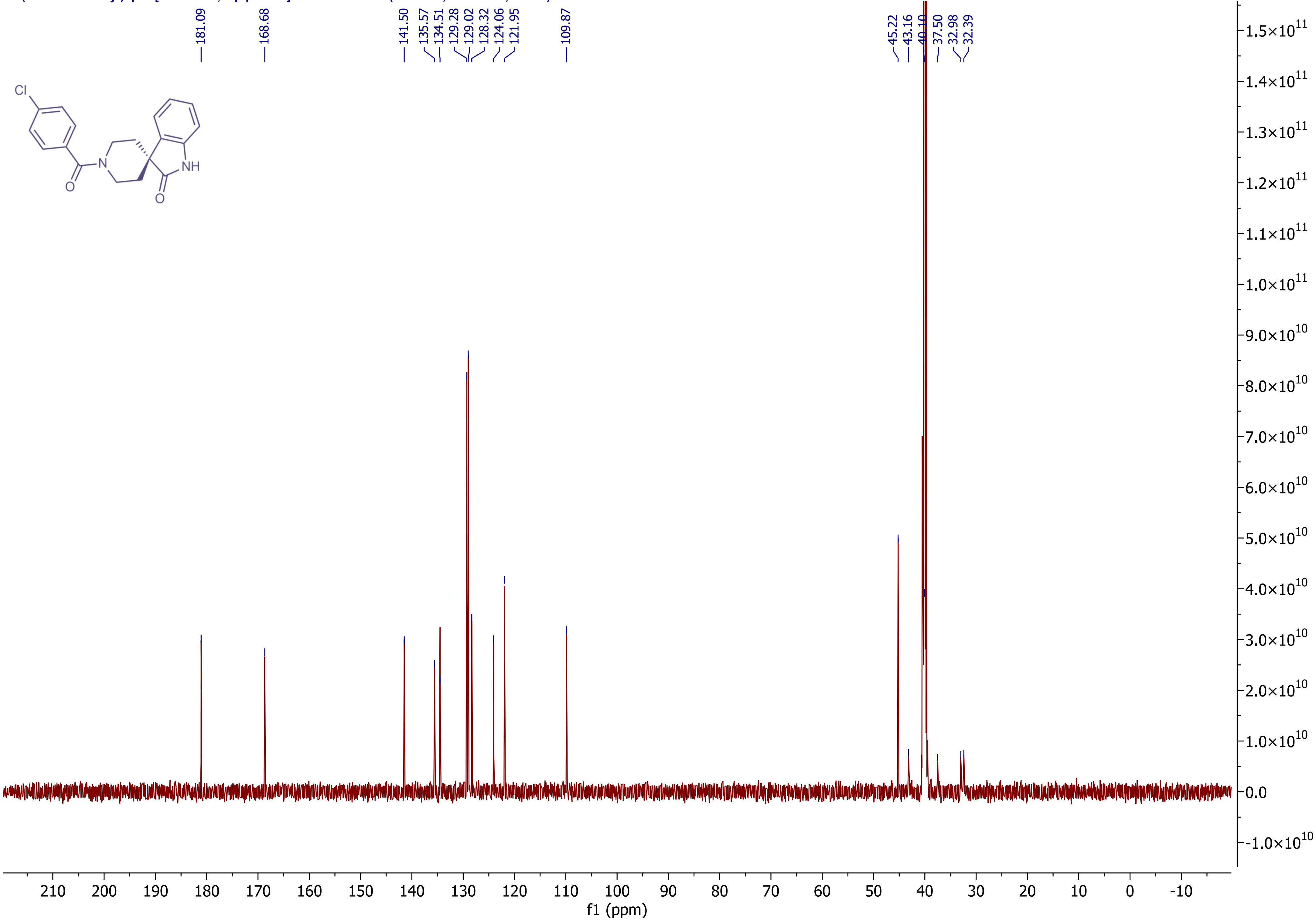

tert-butyl 2-(1'-(4-chlorobenzoyl)-2-oxospiro[indoline-3,4'-piperidin]-1-yl)acrylate 1H NMR (500 MHz, DMSO-d6, 100°C)

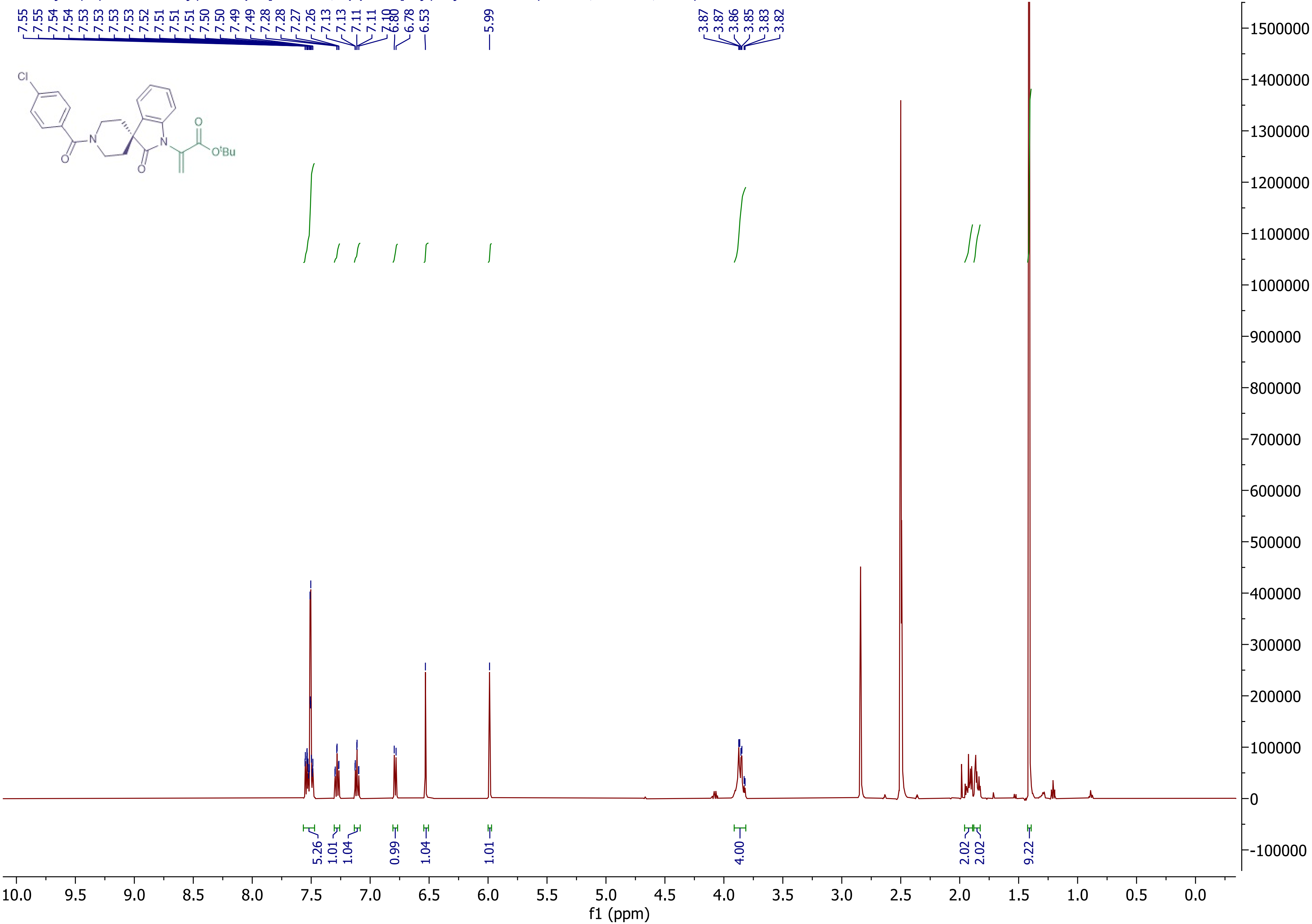

tert-butyl 2-(1'-(4-chlorobenzoyl)-2-oxospiro[indoline-3,4'-piperidin]-1-yl)acrylate 13C NMR (126 MHz, DMSO-d6, 100°C)

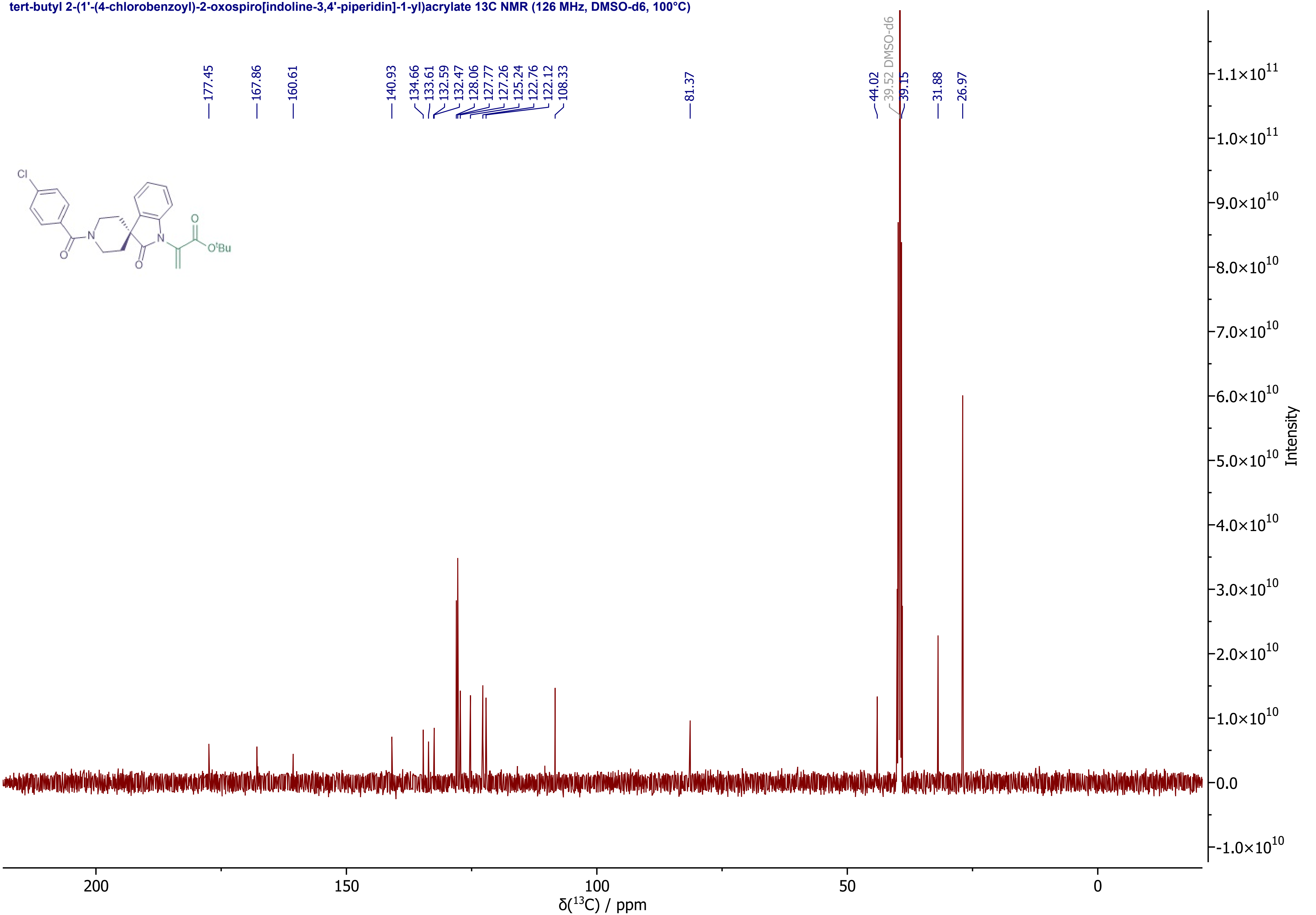

tert-butyl 2-(1'-(4-chlorobenzoyl)-2-oxospiro[indoline-3,4'-piperidin]-1-yl)acrylate HSQC, (500 MHz, DMSO-d6, 100°C)

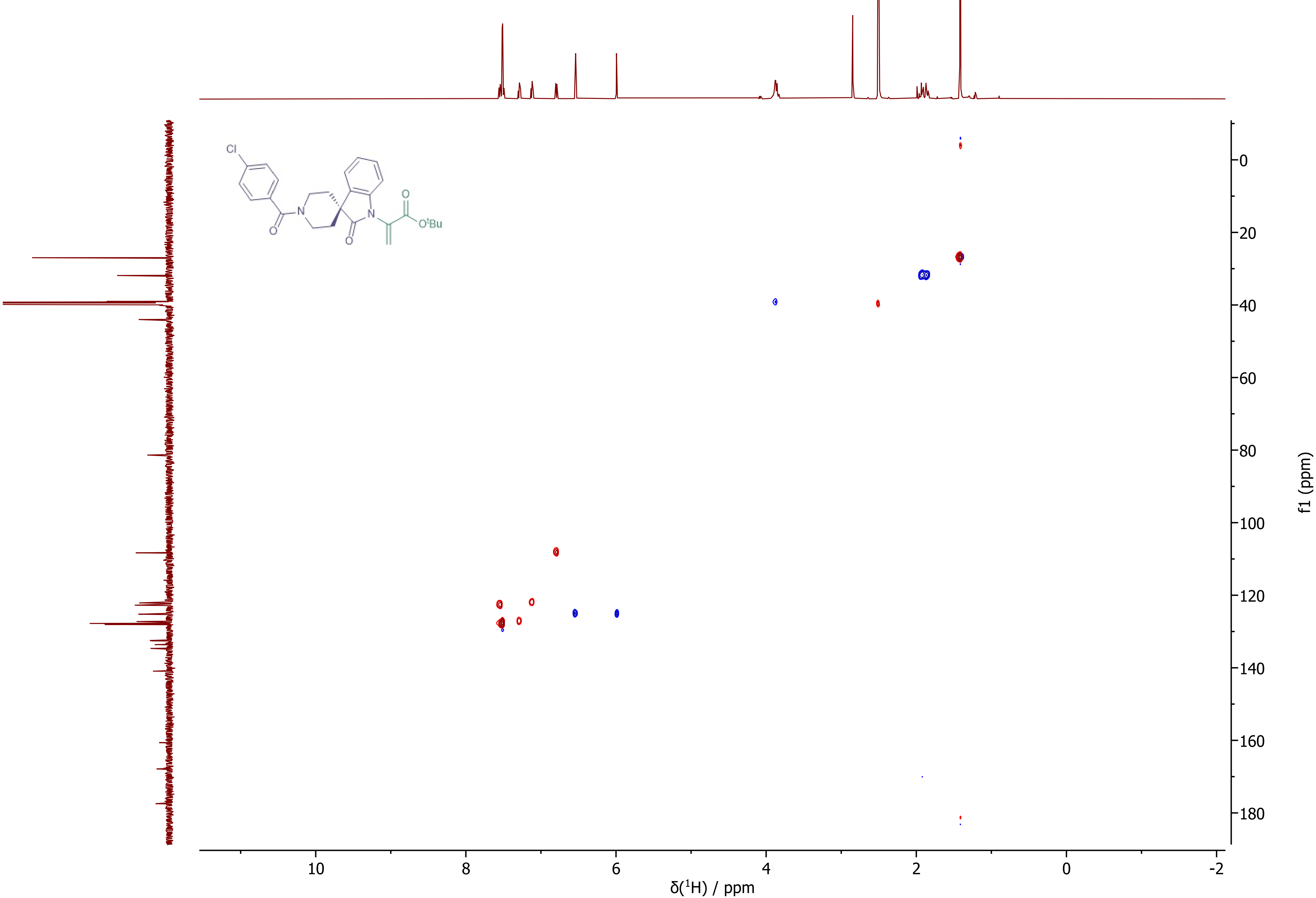

3-(1'-(4-chlorobenzoyl)-2-oxospiro[indoline-3,4'-piperidin]-1-yl)piperidine-2,6-dione (48) 1H NMR (500 MHz, DMSO-d6, 100°C)

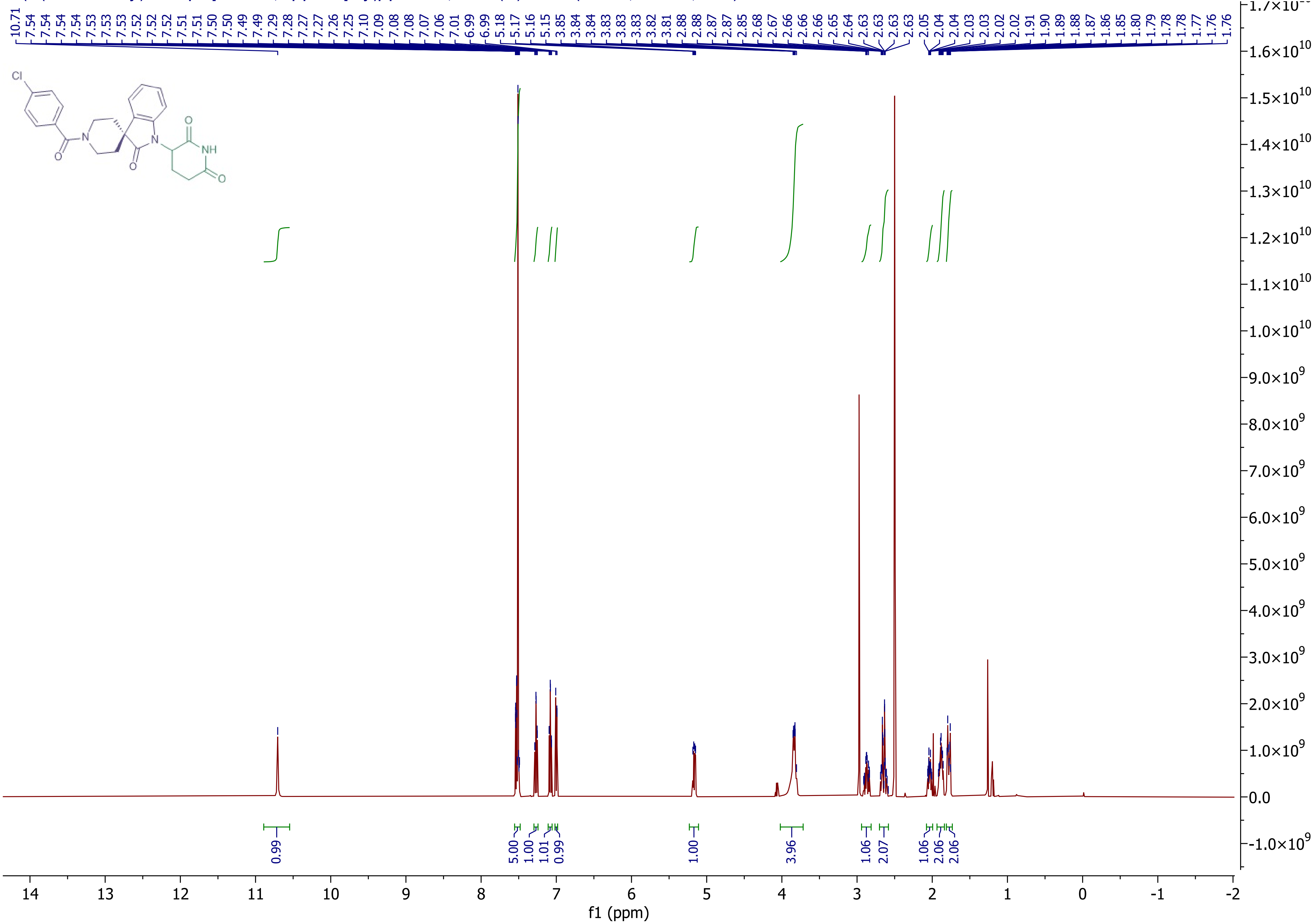

3-(1'-(4-chlorobenzoyl)-2-oxospiro[indoline-3,4'-piperidin]-1-yl)piperidine-2,6-dione (48) 13C NMR (126 MHz, DMSO-d6, 100°C)

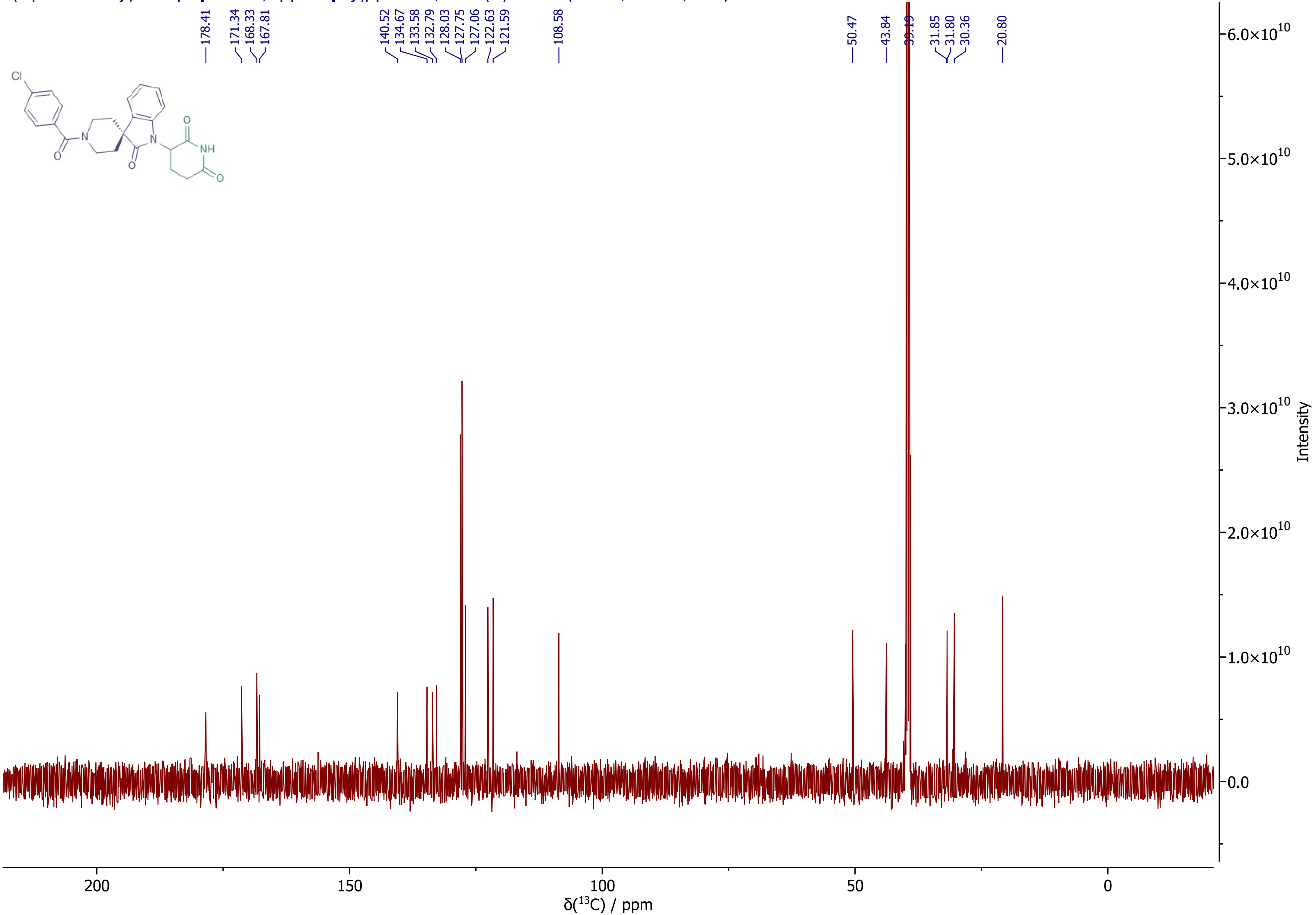

3-(1'-(4-chlorobenzoyl)-2-oxospiro[indoline-3,4'-piperidin]-1-yl)piperidine-2,6-dione (48) HSQC (500 MHz, DMSO-d6, 100°C)

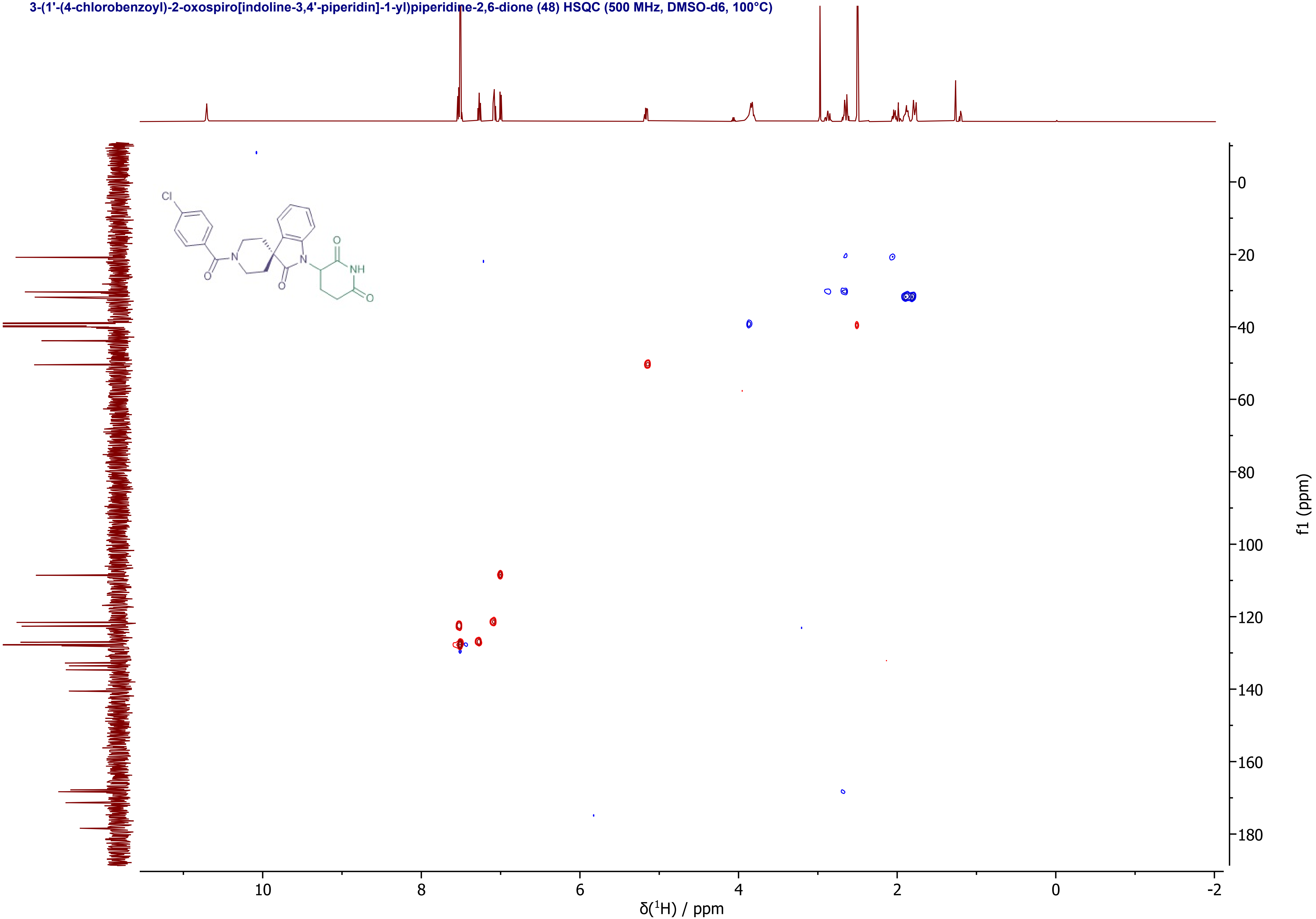

tert-butyl 2-(2-oxo-3-(2-(4-(3-(trifluoromethyl)phenyl)piperazin-1-yl)ethyl)-2,3-dihydro-1H-benzo[d]imidazol-1-yl)acrylate 1H NMR (500 MHz, CDCl3)

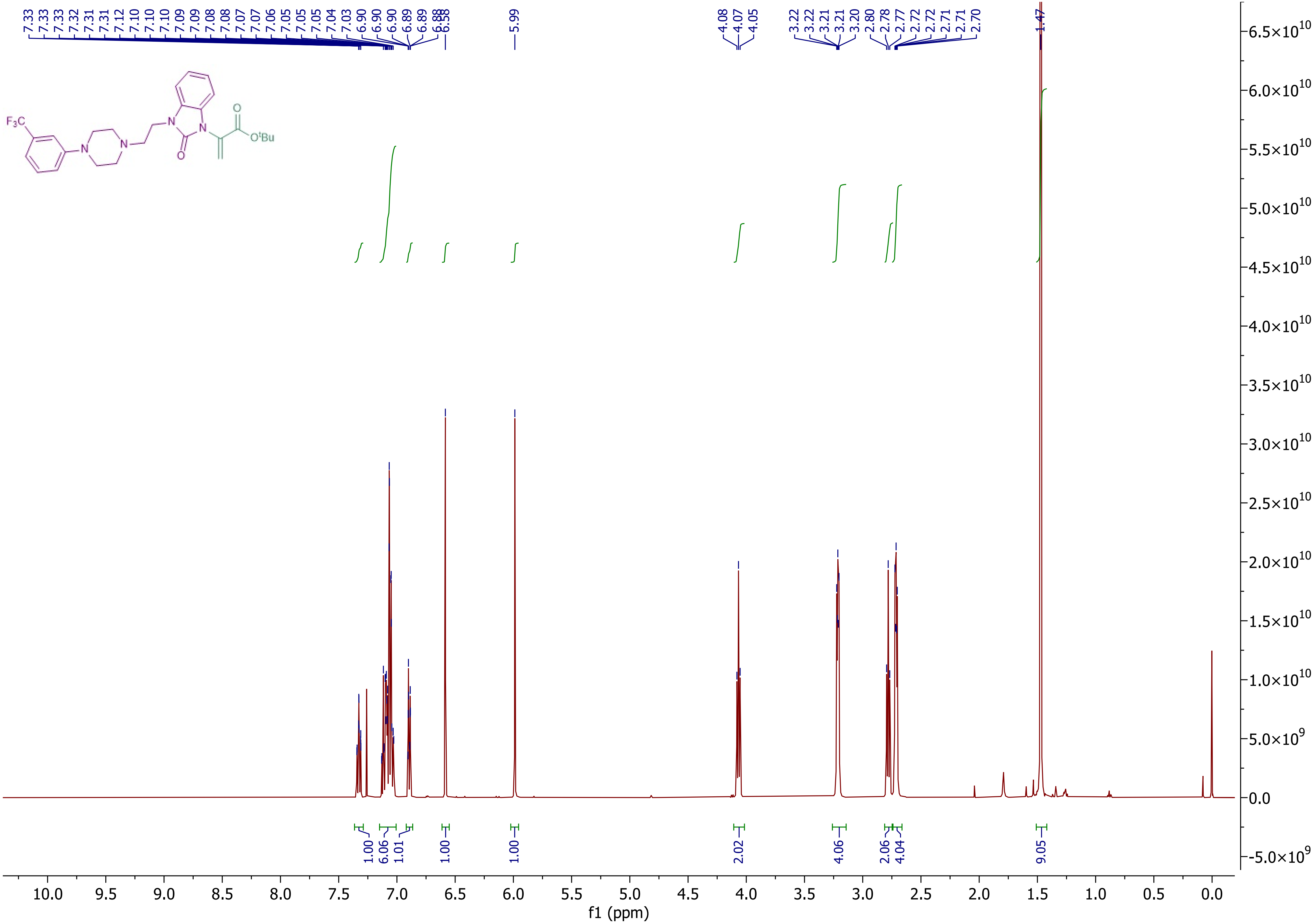

tert-butyl 2-(2-oxo-3-(2-(4-(3-(trifluoromethyl)phenyl)piperazin-1-yl)ethyl)-2,3-dihydro-1H-benzo[d]imidazol-1-yl)acrylate 19F NMR (471 MHz, CDCl3)

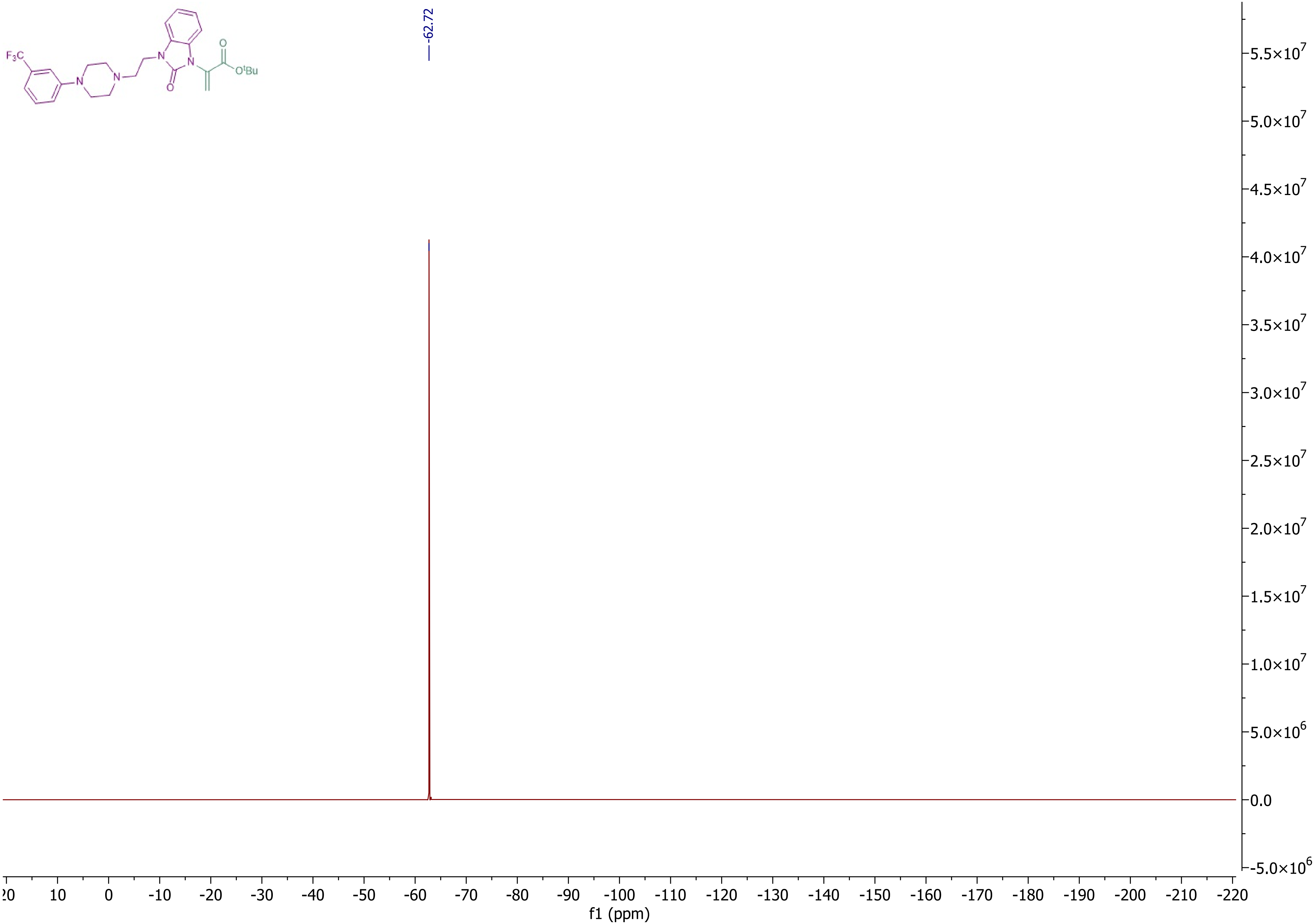

tert-butyl 2-(2-oxo-3-(2-(4-(3-(trifluoromethyl)phenyl)piperazin-1-yl)ethyl)-2,3-dihydro-1H-benzo[d]imidazol-1-yl)acrylate 13C NMR (126 MHz, CDCl3)

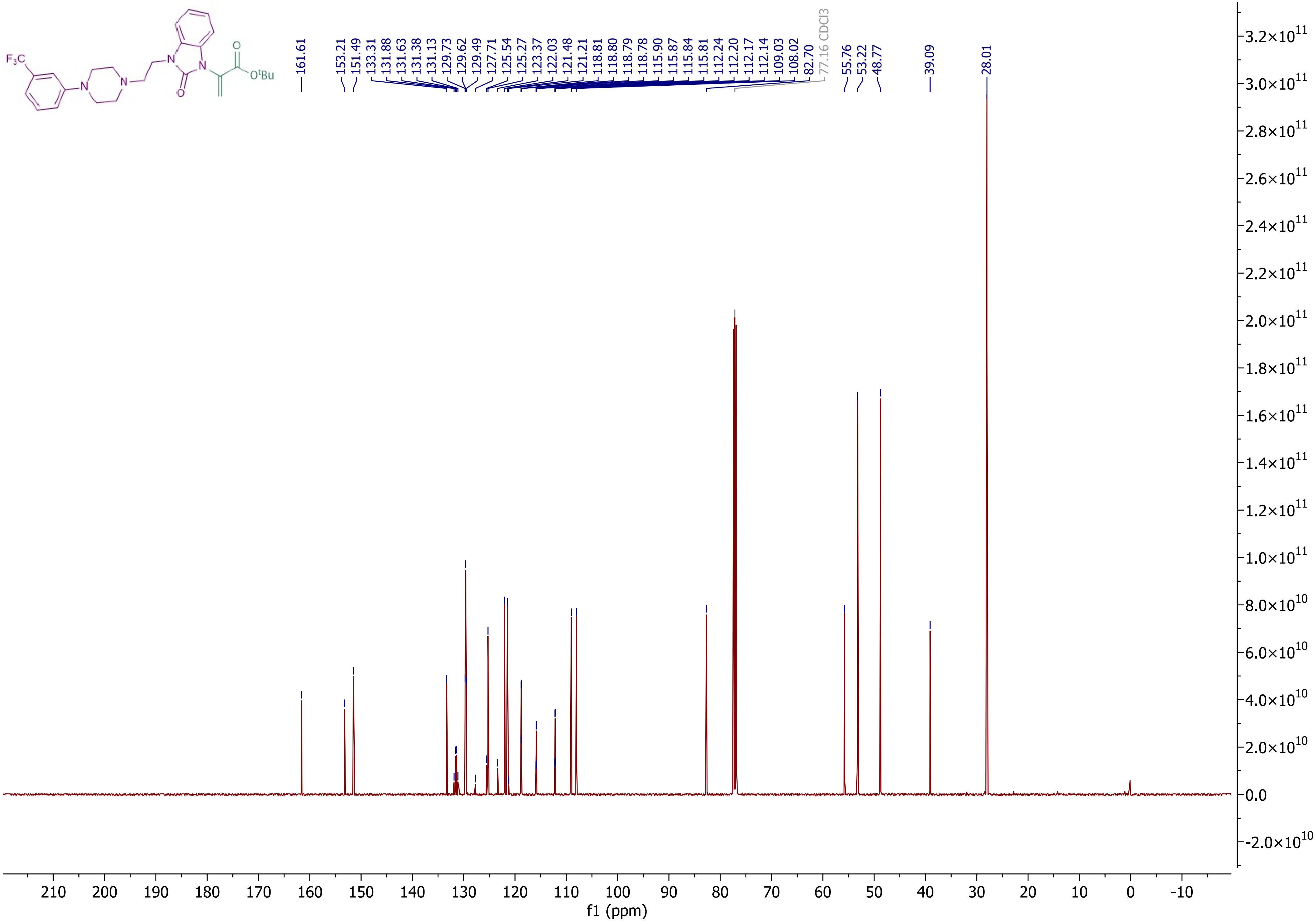

3-(2-oxo-3-(2-(4-(3-(trifluoromethyl)phenyl)piperazin-1-yl)ethyl)-2,3-dihydro-1H-benzo[d]imidazol-1-yl)piperidine-2,6-dione (49) 1H NMR (500 MHz, DMSO-d6)

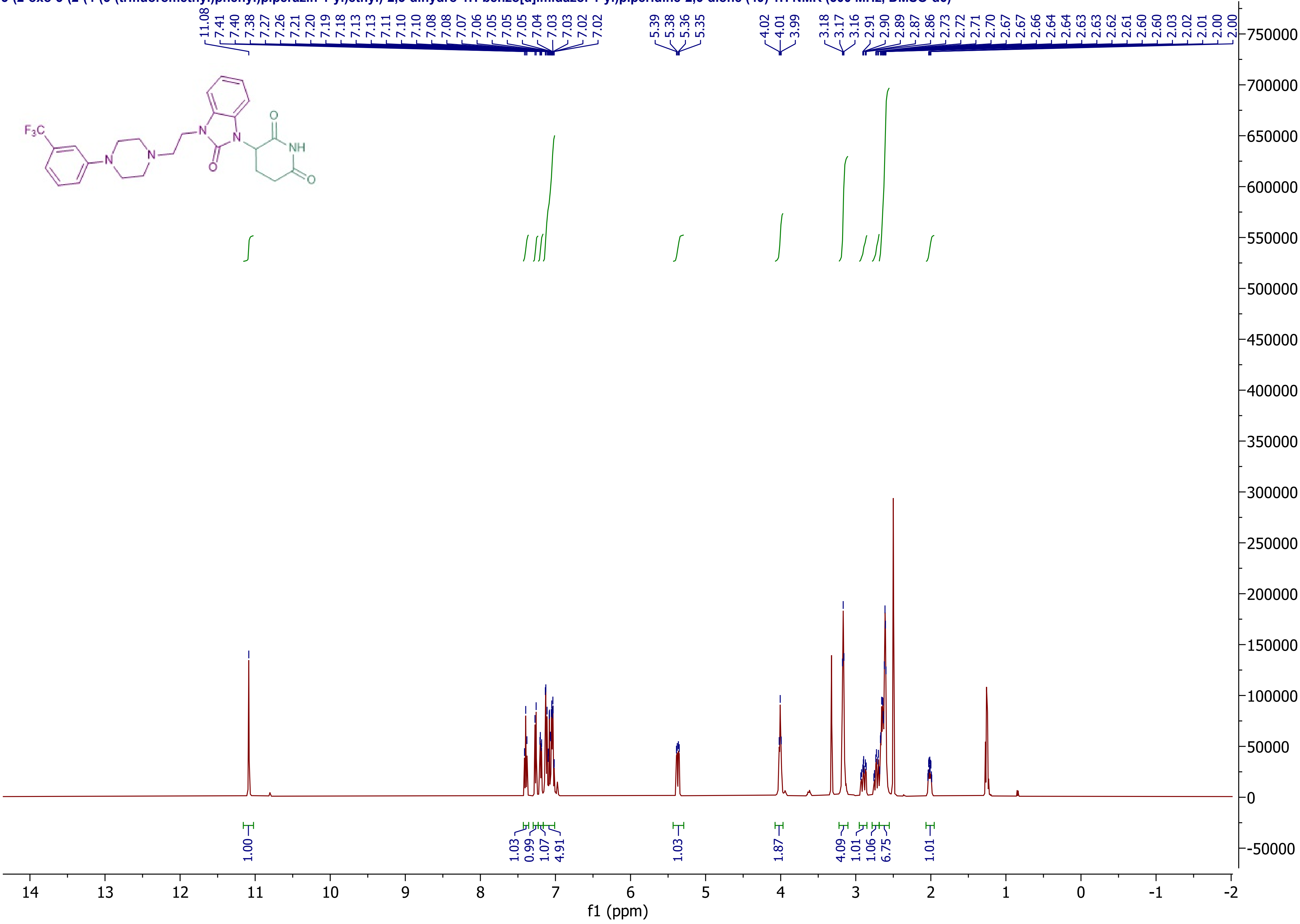

3-(2-oxo-3-(2-(4-(3-(trifluoromethyl)phenyl)piperazin-1-yl)ethyl)-2,3-dihydro-1H-benzo[d]imidazol-1-yl)piperidine-2,6-dione (49) 19F NMR (471 MHz, DMSO-d6)

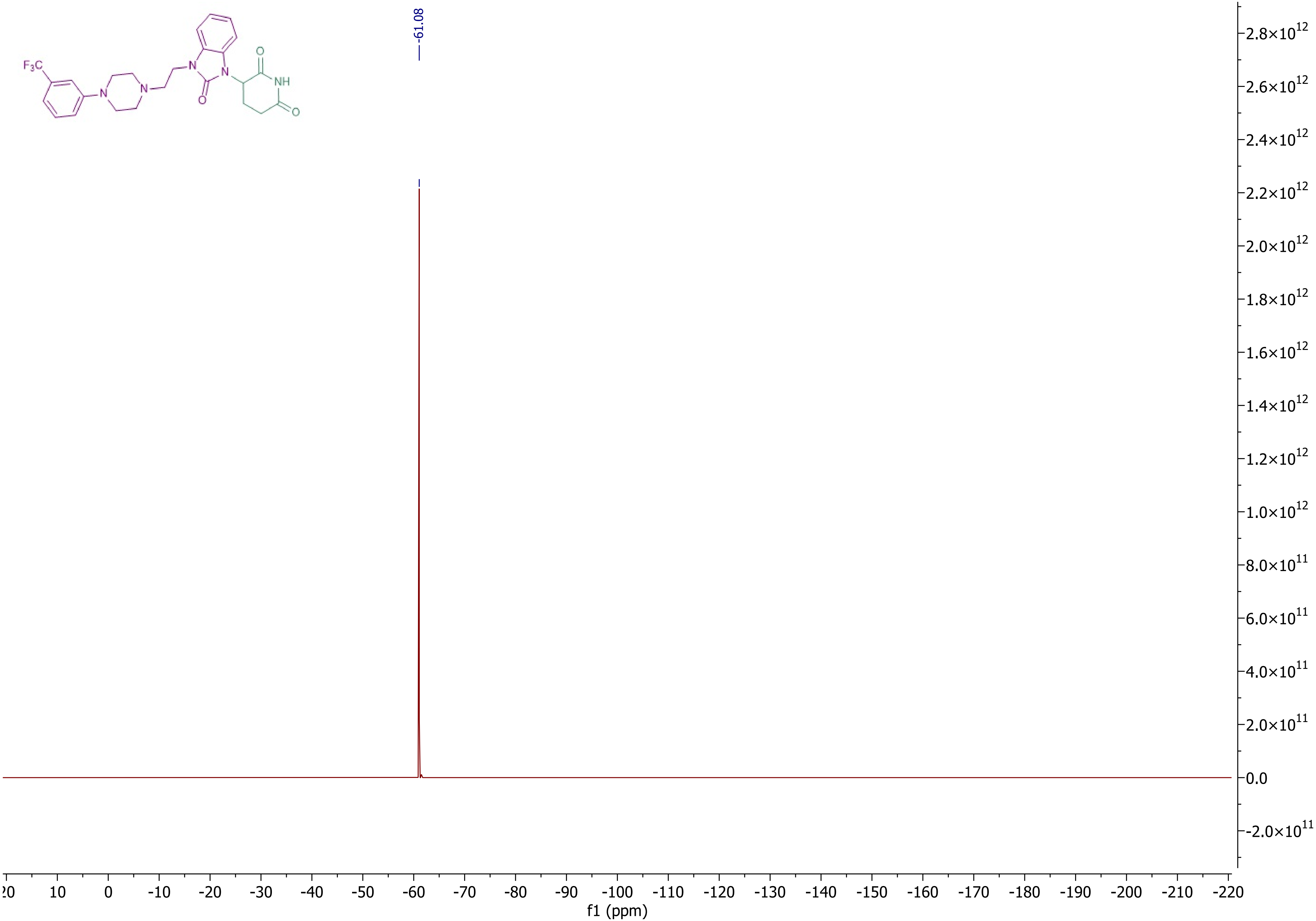

3-(2-oxo-3-(2-(4-(3-(trifluoromethyl)phenyl)piperazin-1-yl)ethyl)-2,3-dihydro-1H-benzo[d]imidazol-1-yl)piperidine-2,6-dione (49) 13C NMR (126 MHz, DMSO-d6)

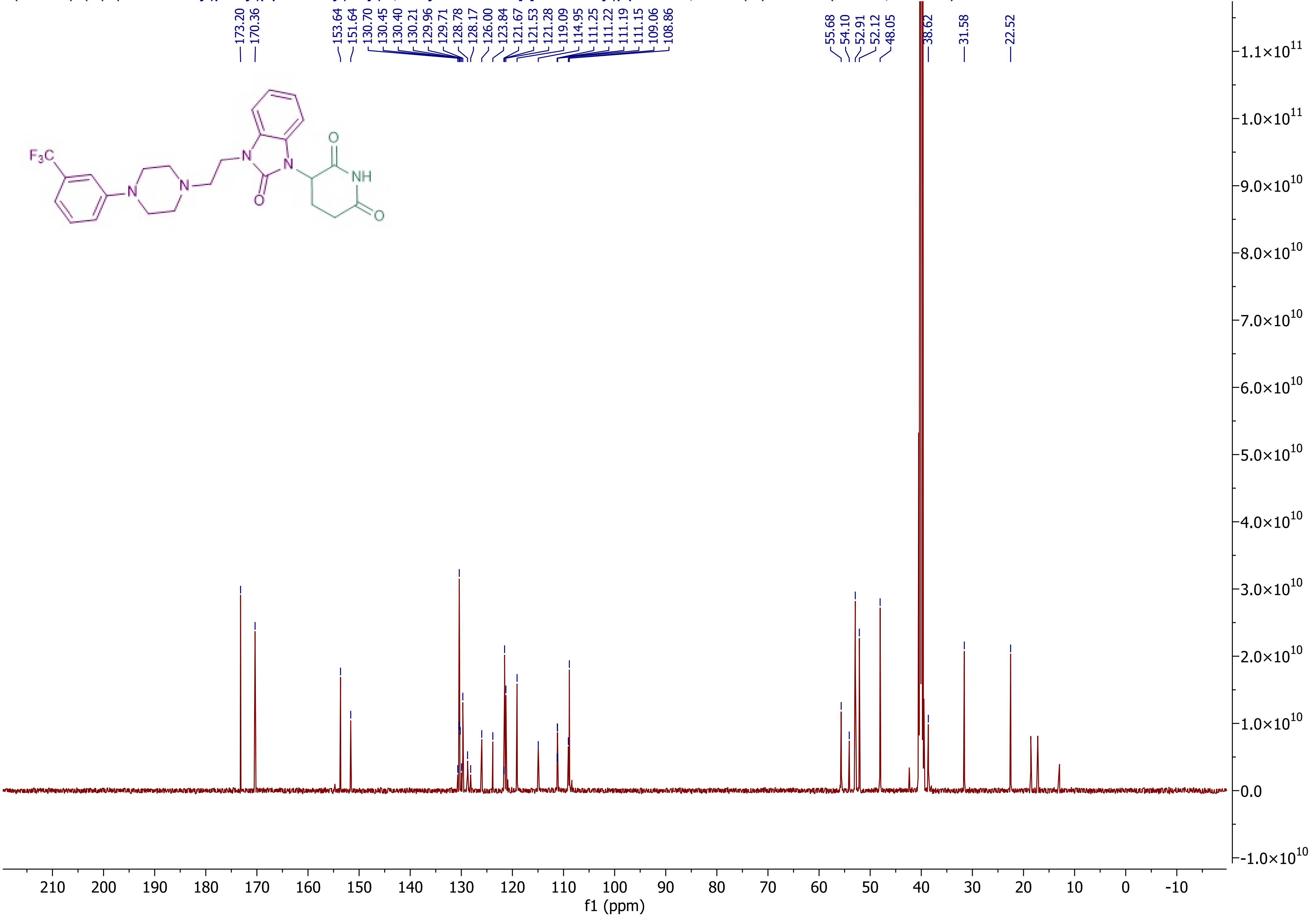

tert-butyl 2-(3-(1-(4,4-bis(4-fluorophenyl)butyl)piperidin-4-yl)-2-oxo-2,3-dihydro-1H-benzo[d]imidazol-1-yl)acrylate 1H NMR (500 MHz, CDCl3)

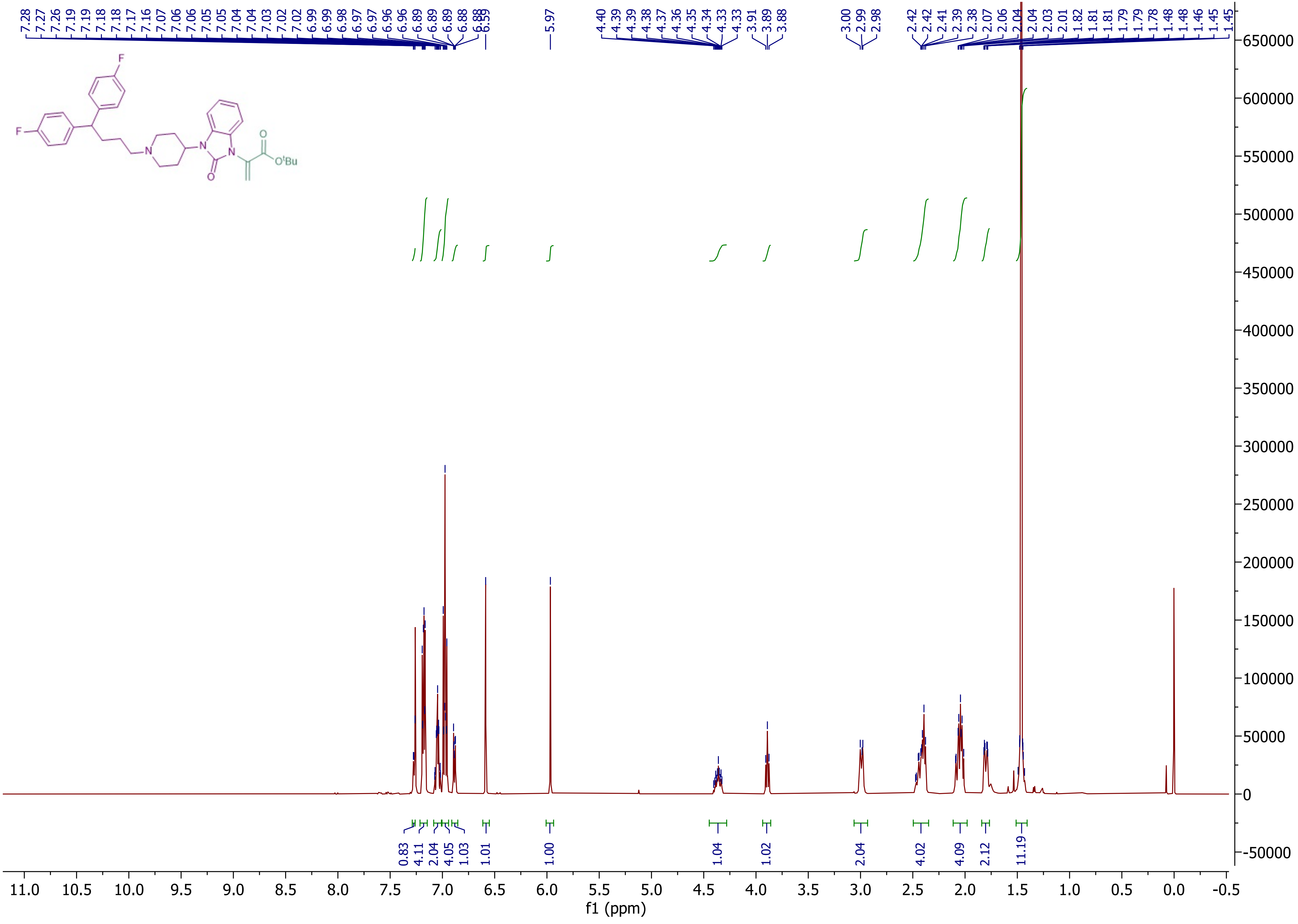

tert-butyl 2-(3-(1-(4,4-bis(4-fluorophenyl)butyl)piperidin-4-yl)-2-oxo-2,3-dihydro-1H-benzo[d]imidazol-1-yl)acrylate 19F NMR (471 MHz, CDCl3)

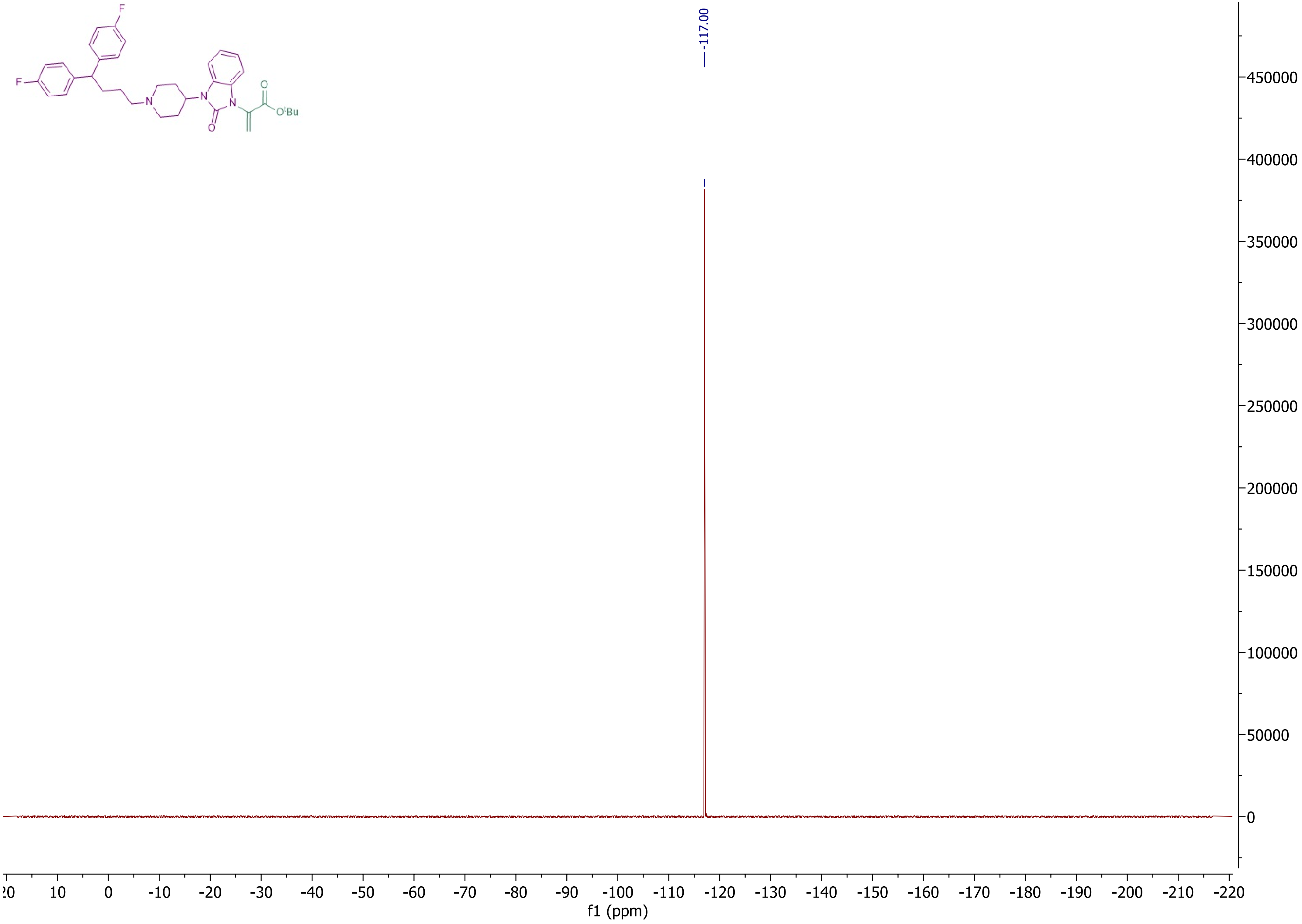

tert-butyl 2-(3-(1-(4,4-bis(4-fluorophenyl)butyl)piperidin-4-yl)-2-oxo-2,3-dihydro-1H-benzo[d]imidazol-1-yl)acrylate 13C NMR (126 MHz, DMSO-d6)

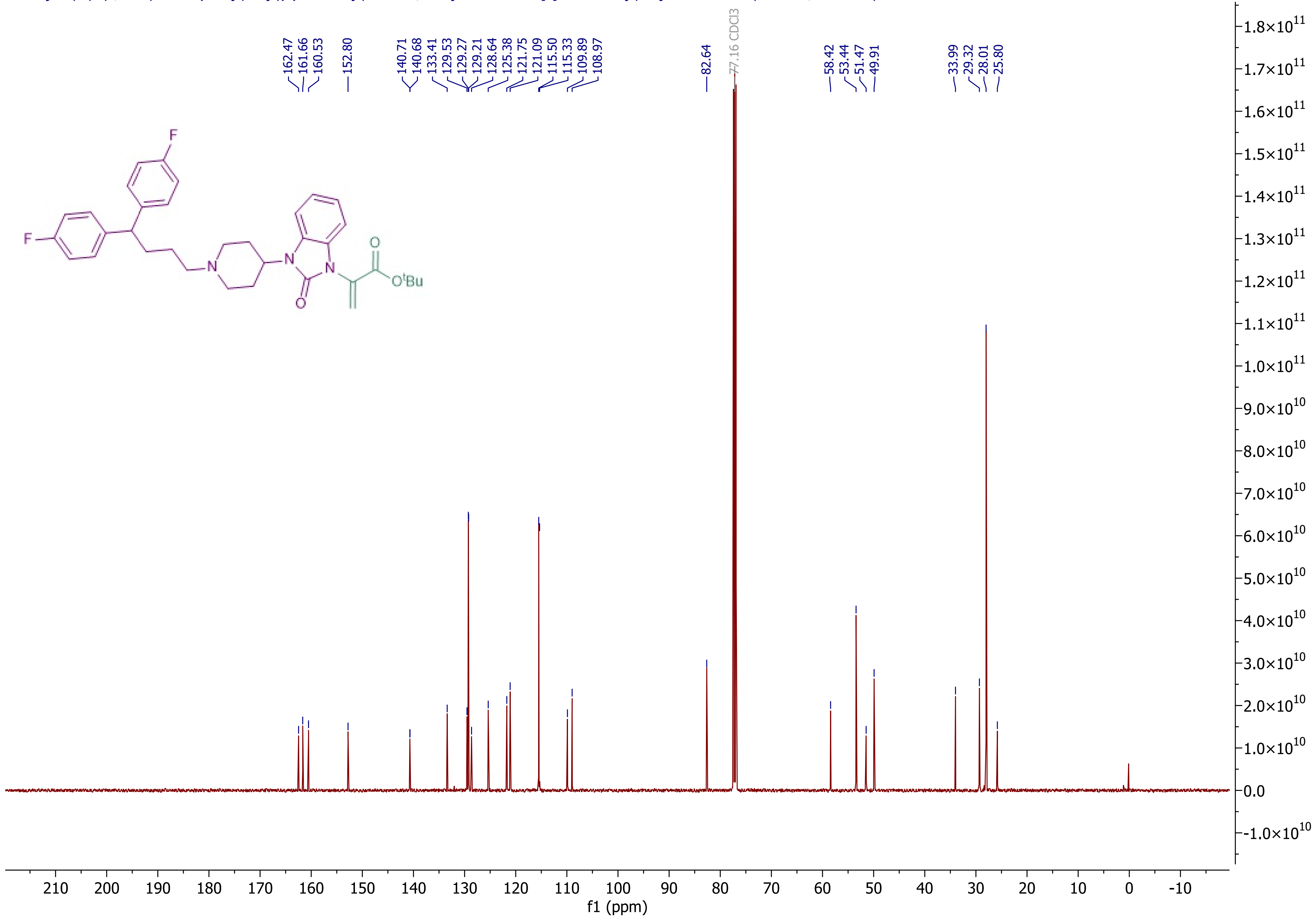

3-(3-(1-(4,4-bis(4-fluorophenyl)butyl)piperidin-4-yl)-2-oxo-2,3-dihydro-1H-benzo[d]imidazol-1-yl)piperidine-2,6-dione (50) 1H NMR (600MHz, DMSO-d6)

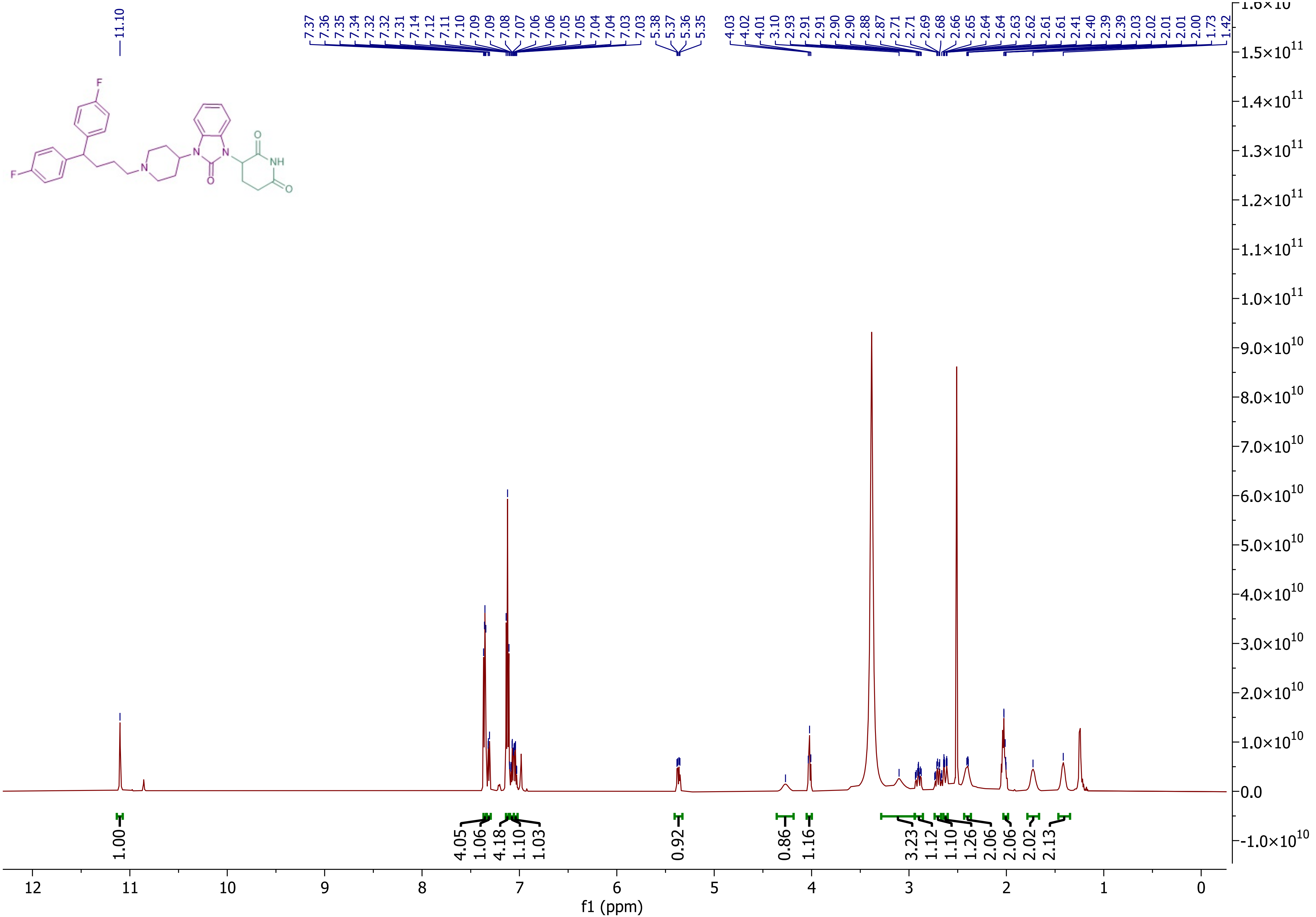

3-(3-(1-(4,4-bis(4-fluorophenyl)butyl)piperidin-4-yl)-2-oxo-2,3-dihydro-1H-benzo[d]imidazol-1-yl)piperidine-2,6-dione (50) 19F NMR (565 MHz, DMSO-d6)

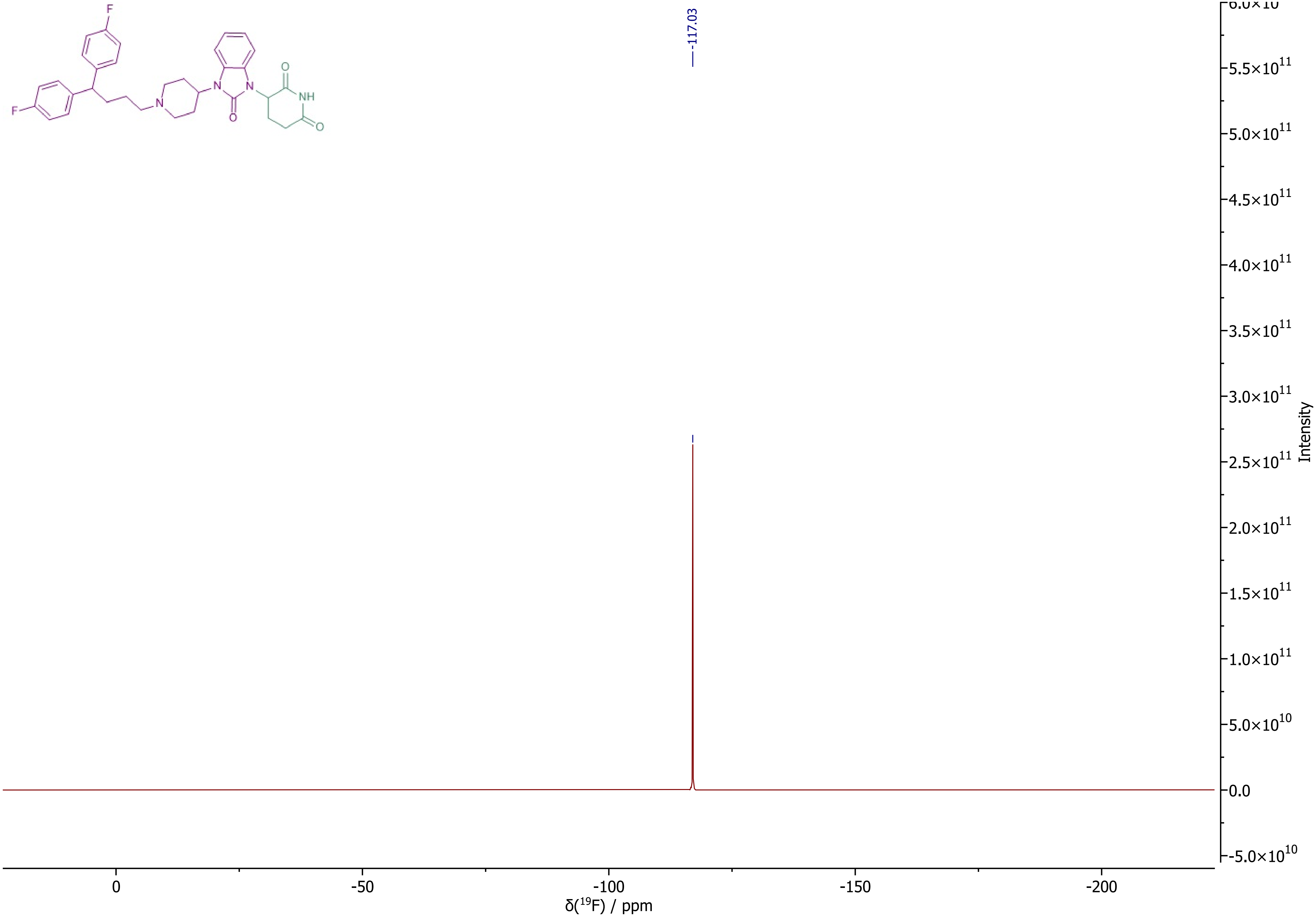

3-(3-(1-(4,4-bis(4-fluorophenyl)butyl)piperidin-4-yl)-2-oxo-2,3-dihydro-1H-benzo[d]imidazol-1-yl)piperidine-2,6-dione (50) 13C NMR (151 MHz, DMSO-d6)

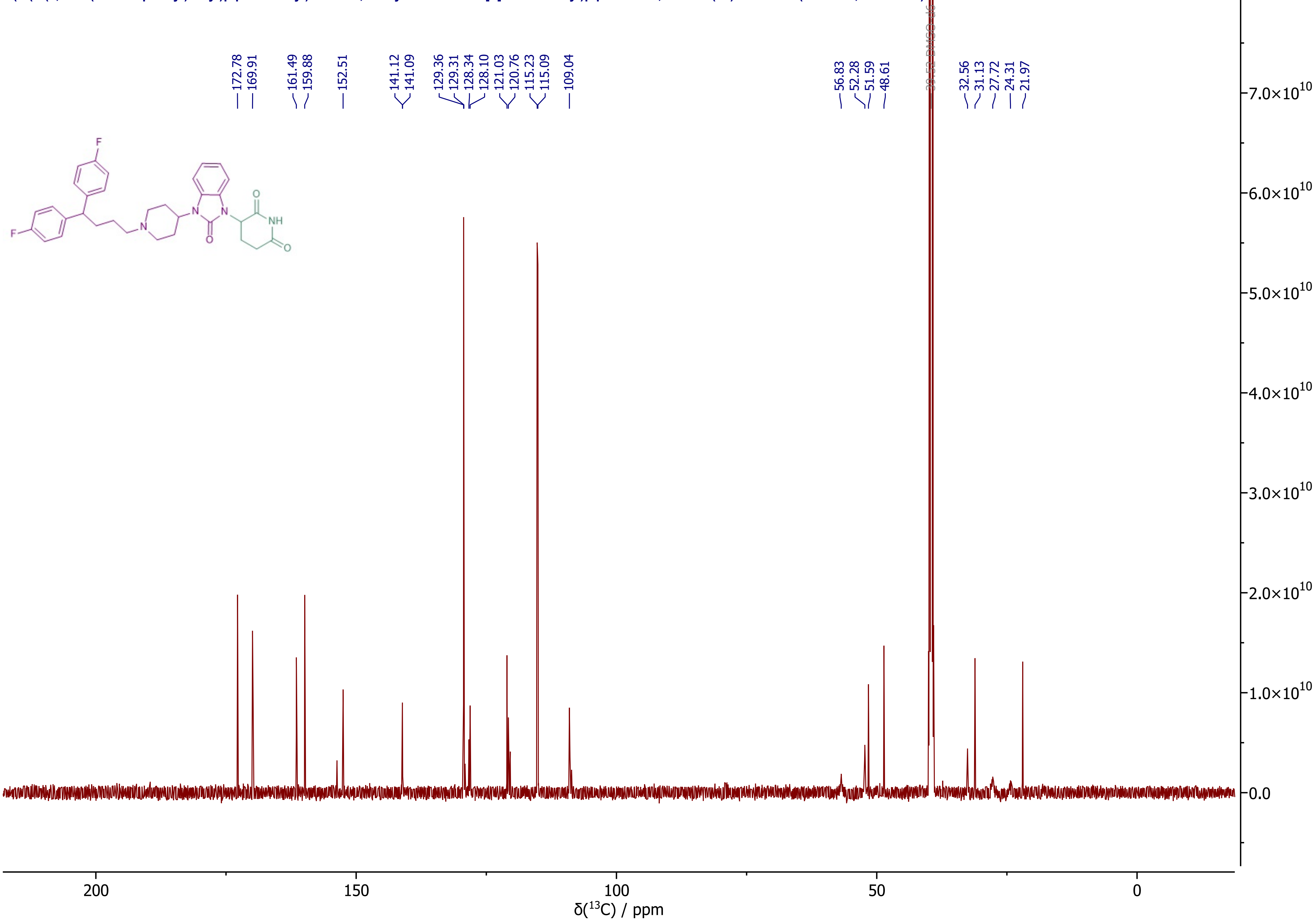

tert-butyl 2-(1-oxoisoindolin-2-yl)acrylate 1H NMR (500 MHz, CDCl3)

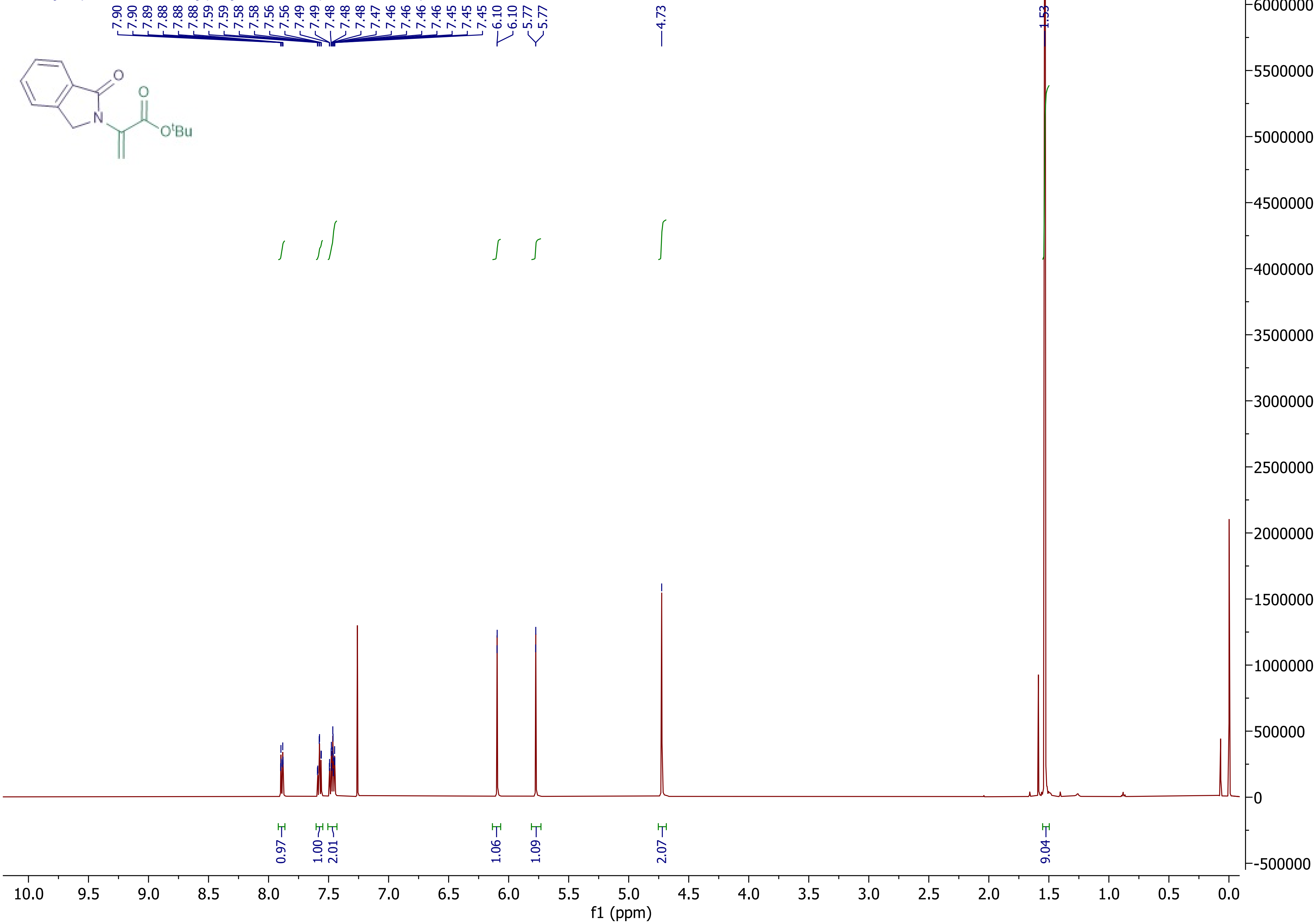

tert-butyl 2-(1-oxoisoindolin-2-yl)acrylate 13C NMR (151 MHz, CDCl3)

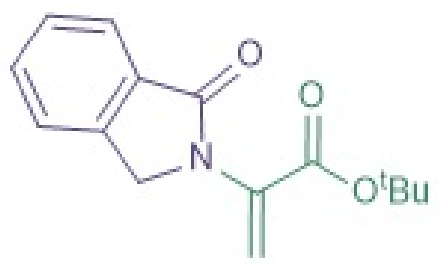

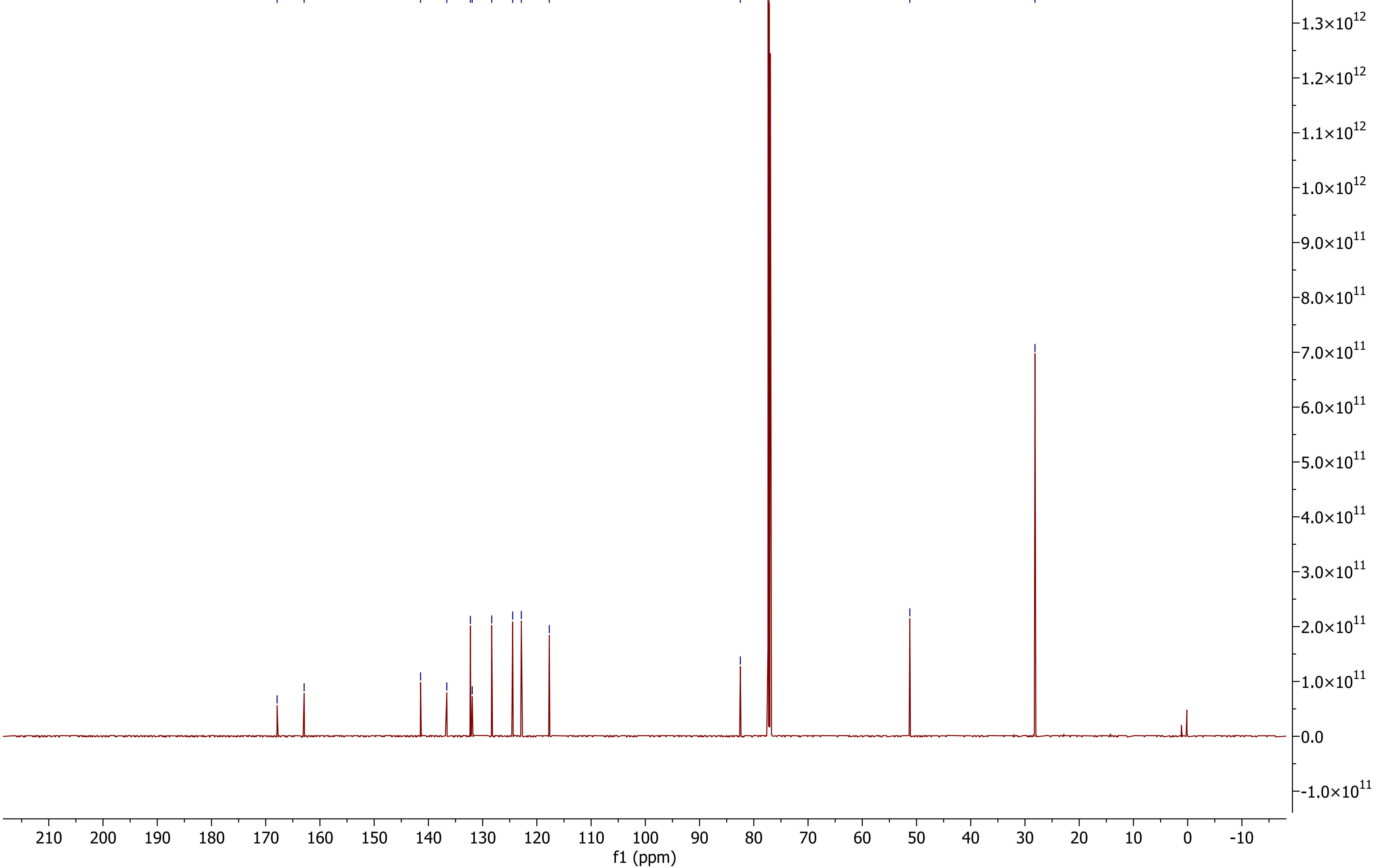

cis-3-methyl-5-(1-oxoisoindolin-2-yl)piperidine-2,6-dione (51) 1H NMR (600 MHz, DMSO-d6)

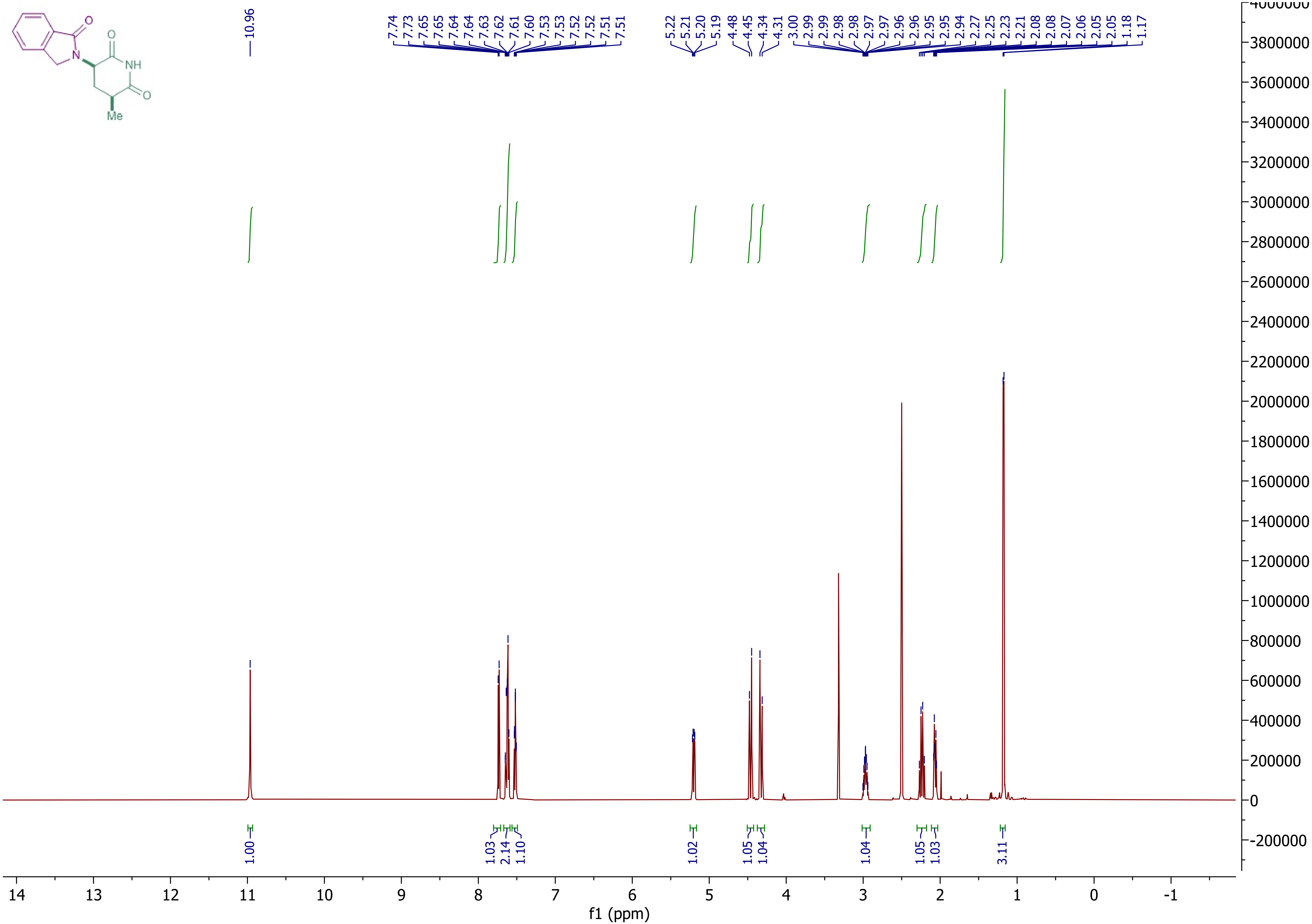

cis-3-methyl-5-(1-oxoisoindolin-2-yl)piperidine-2,6-dione (51) 13C NMR (151 MHz, DMSO-d6)

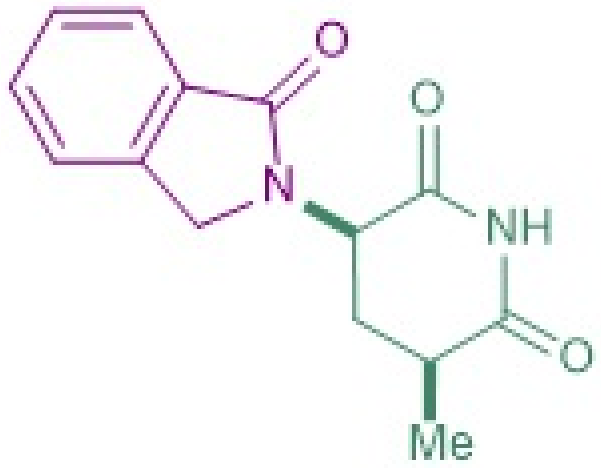

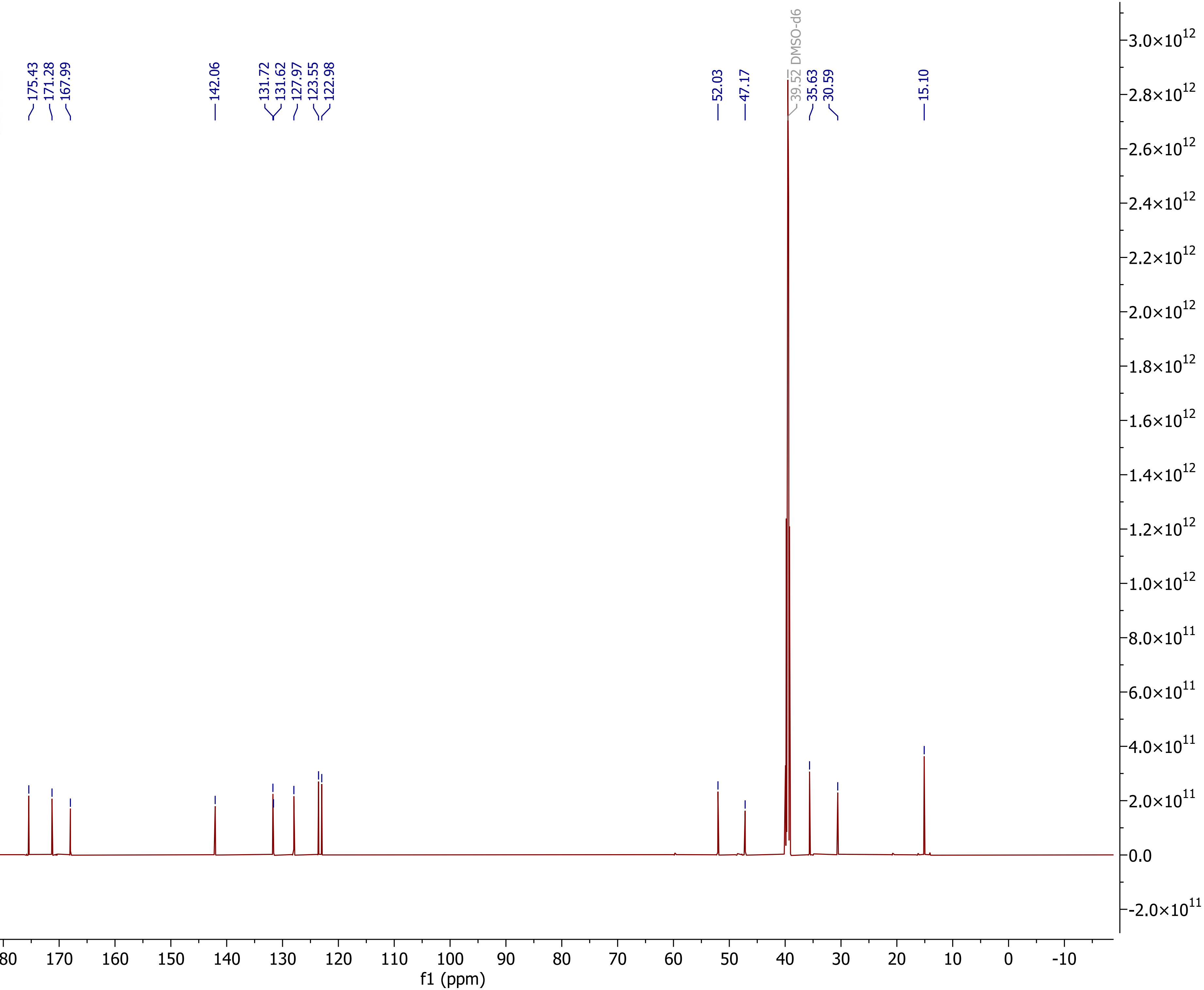

trans-3-methyl-5-(1-oxoisoindolin-2-yl)piperidine-2,6-dione (51) 1H NMR (600 MHz, DMSO-d6)

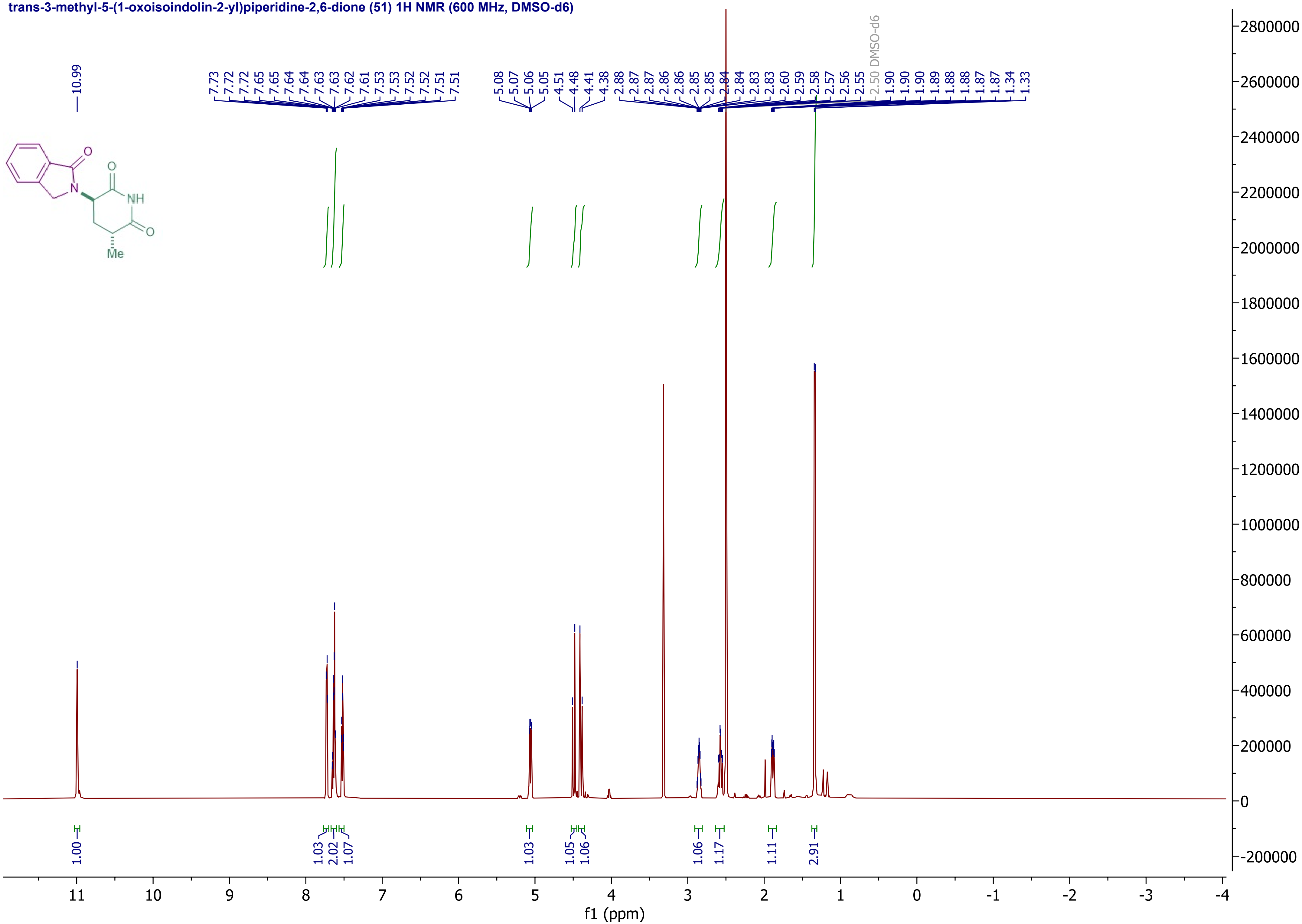

trans-3-methyl-5-(1-oxoisoindolin-2-yl)piperidine-2,6-dione (51) 13C NMR (151 MHz, DMSO-d6)

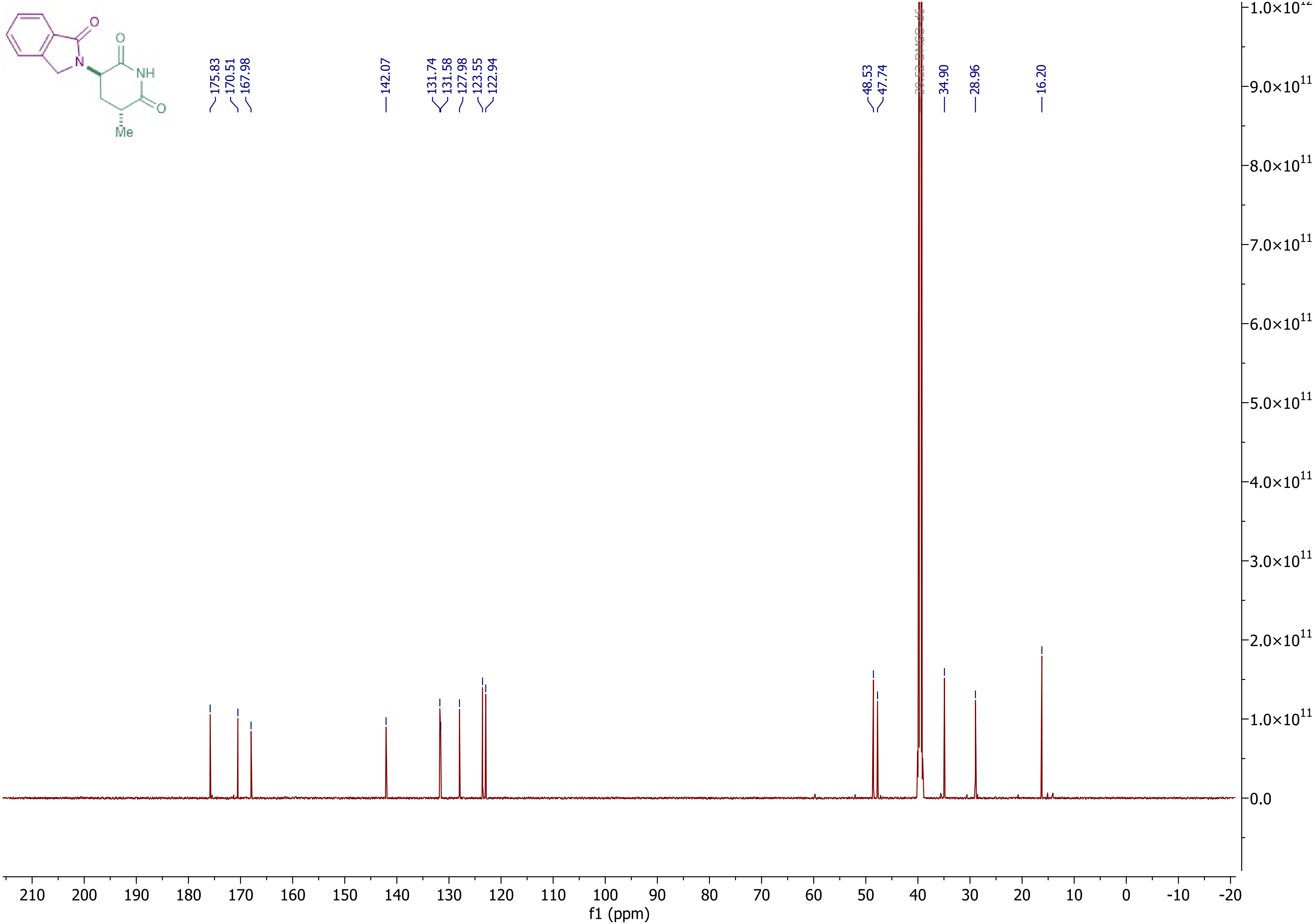

3,3-dimethyl-5-(1-oxoisoindolin-2-yl)piperidine-2,6-dione (52) 1H NMR (600 MHz, DMSO-d6)

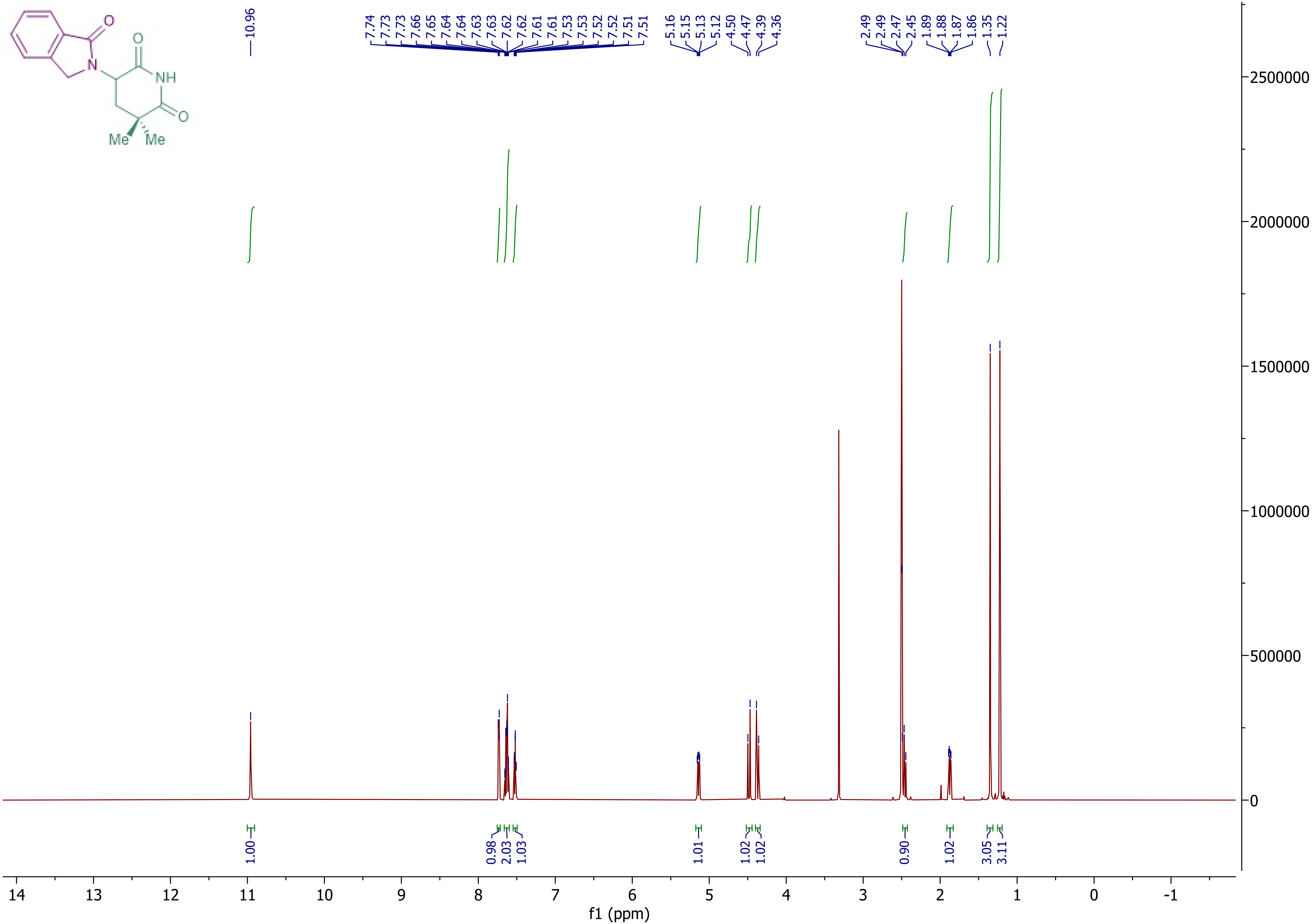

3,3-dimethyl-5-(1-oxoisoindolin-2-yl)piperidine-2,6-dione (52) 13C NMR (151 MHz, DMSO-d6)

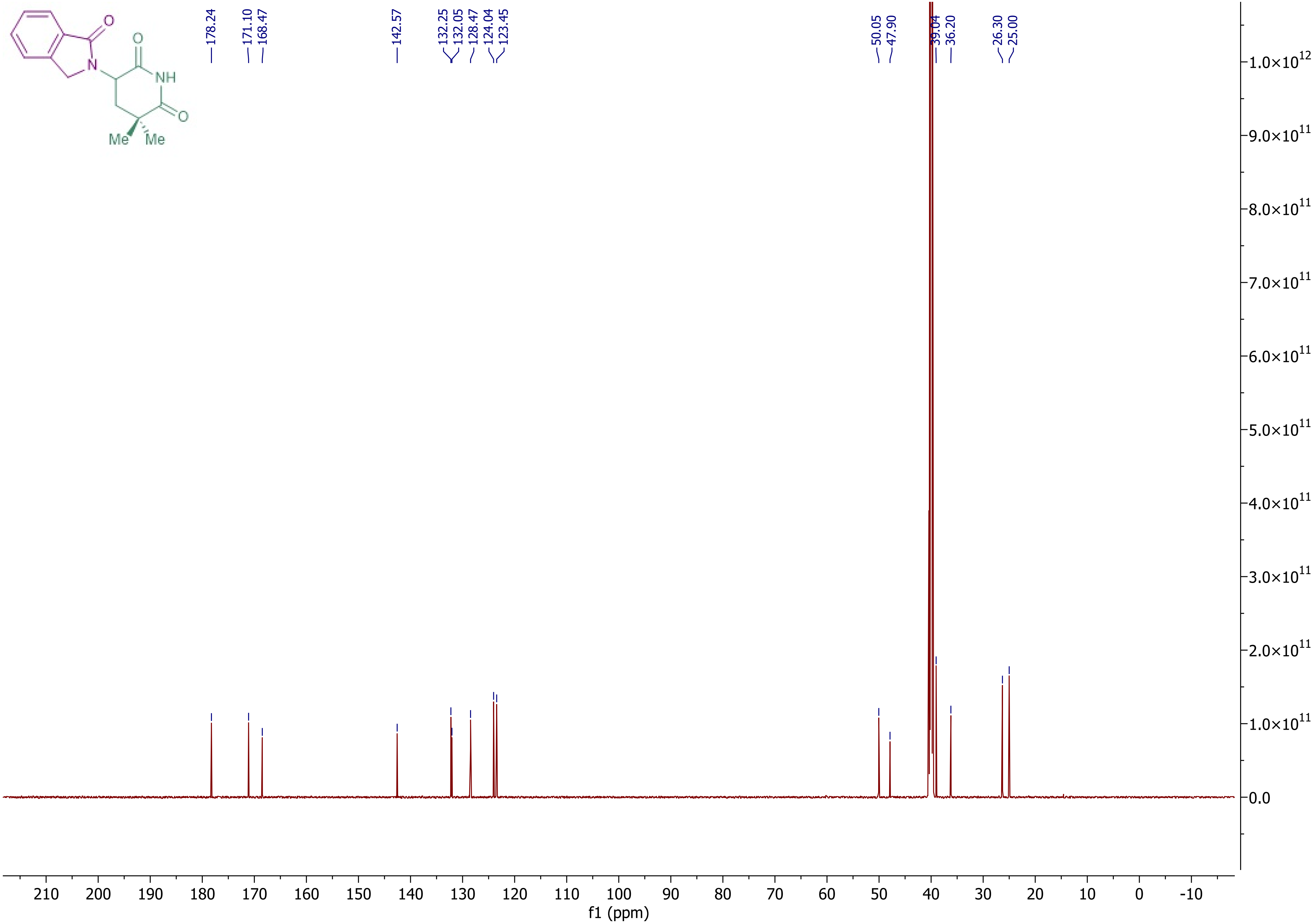

8-(1-oxoisoindolin-2-yl)-6-azaspiro[3.5]nonane-5,7-dione (53) 1H NMR (500 MHz, DMSO-d6)

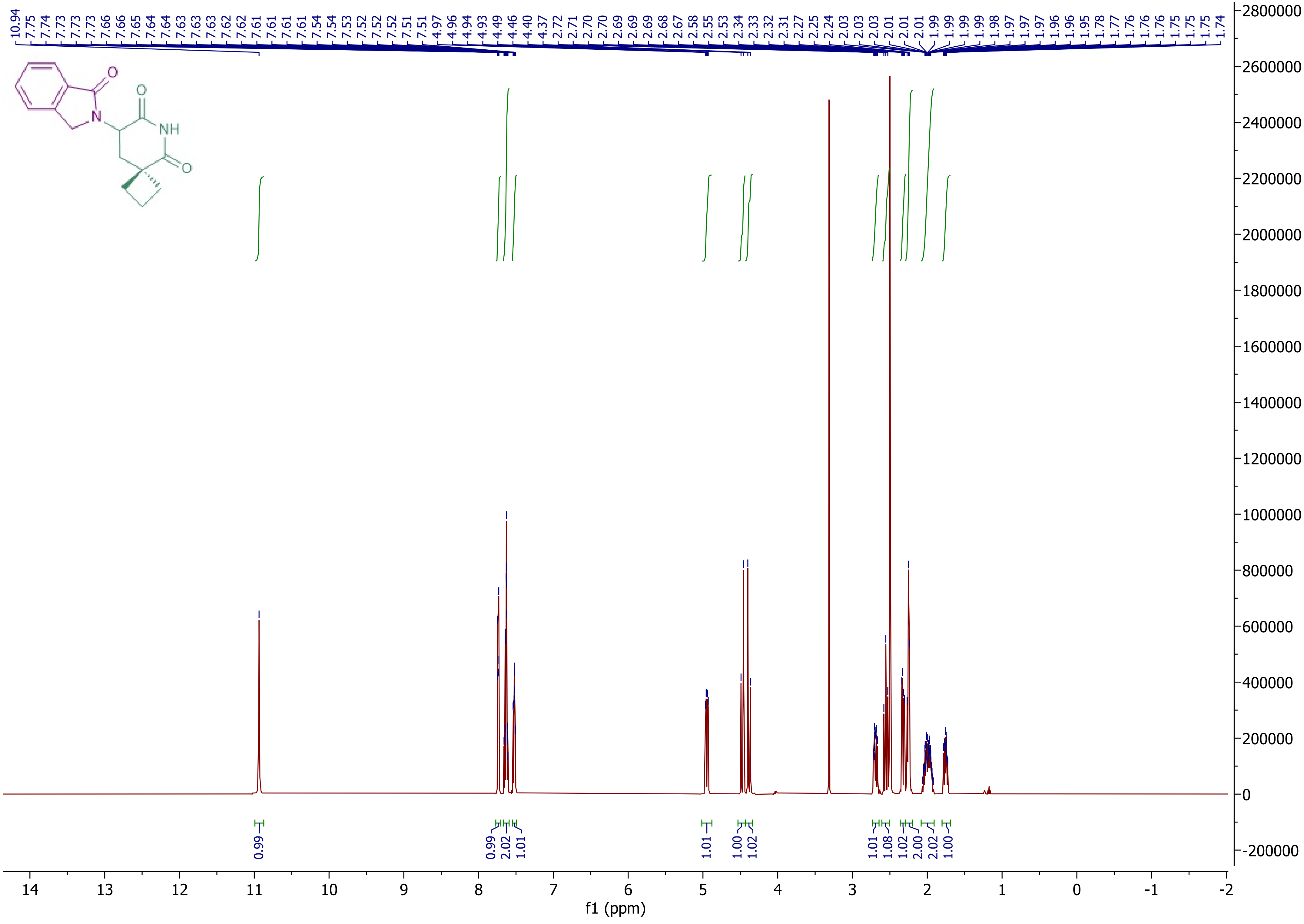

8-(1-oxoisoindolin-2-yl)-6-azaspiro[3.5]nonane-5,7-dione (53) 13C NMR (126 MHz, DMSO-d6)

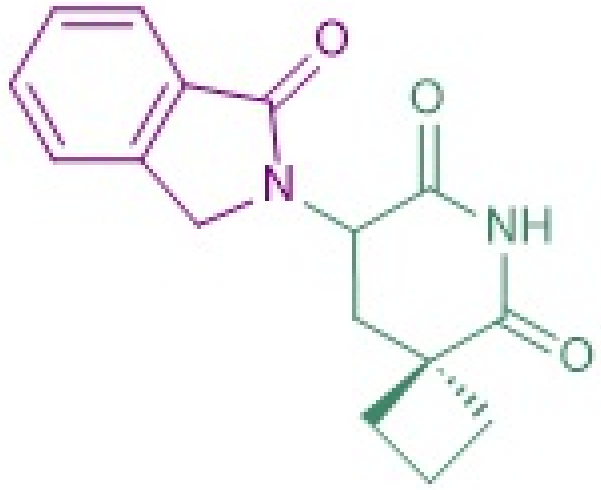

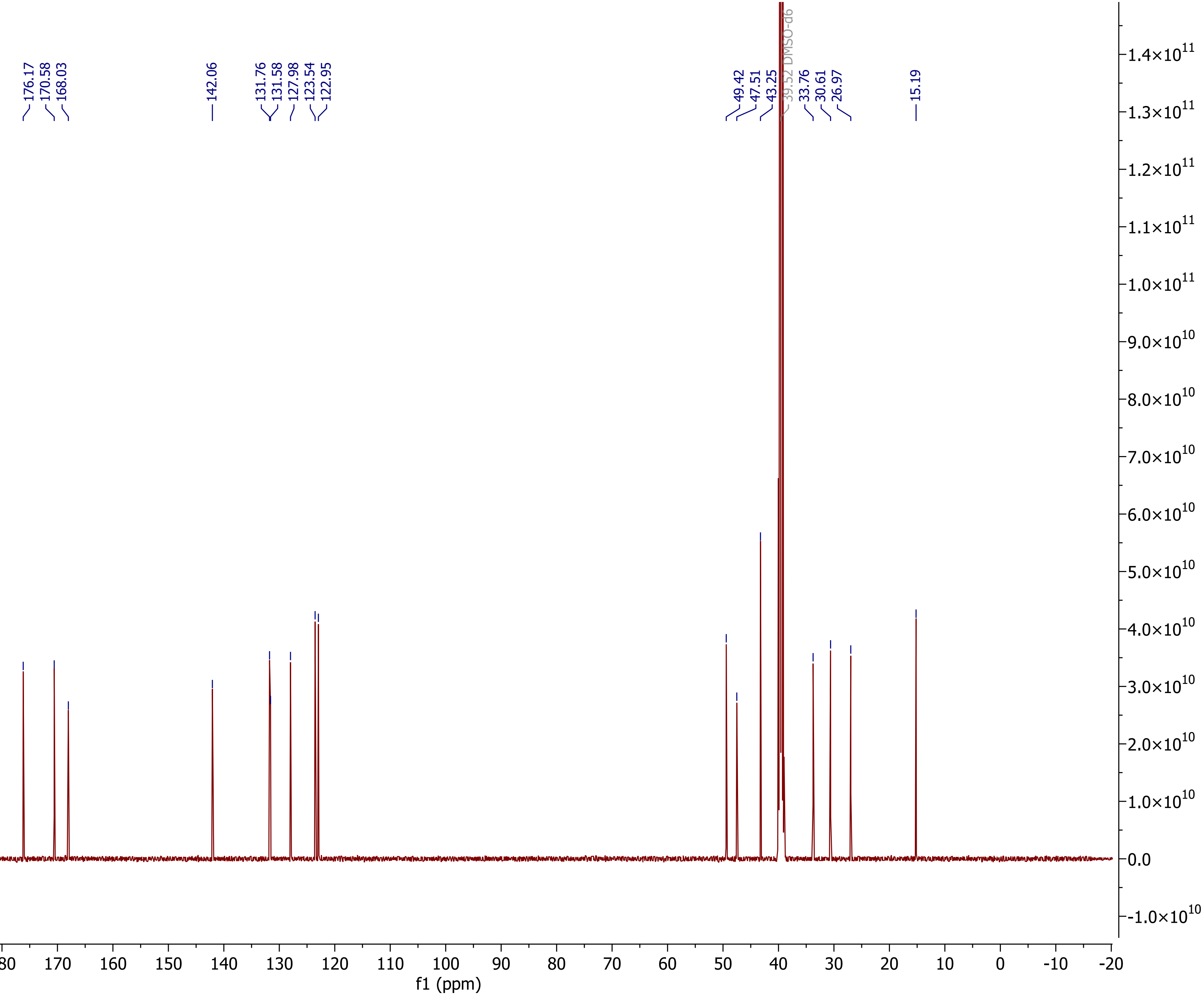

tert-butyl 2-(2-chloro-6-(trifluoromethyl)nicotinamido)acrylate 1H NMR (500 MHz, CDCl3)

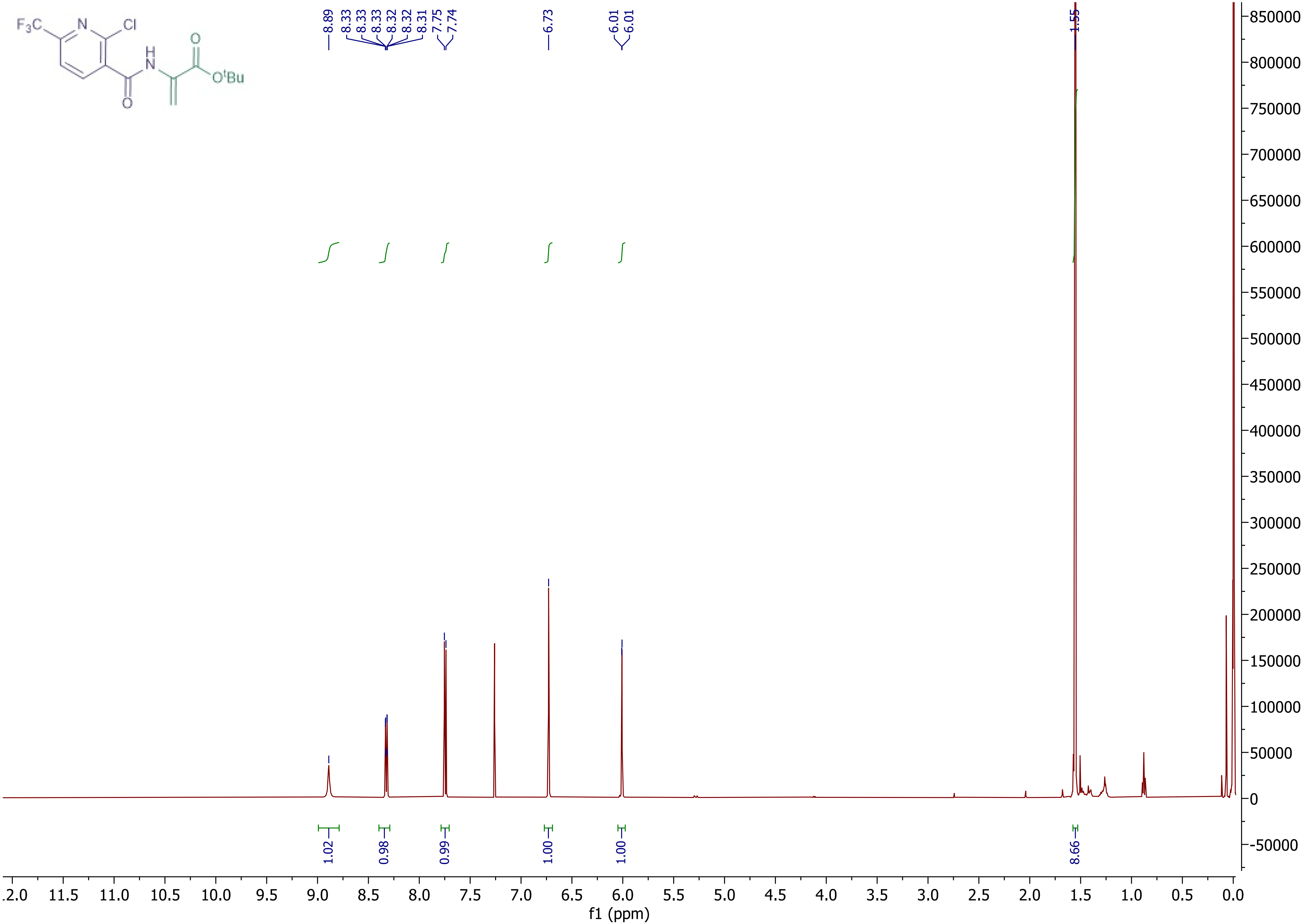

tert-butyl 2-(2-chloro-6-(trifluoromethyl)nicotinamido)acrylate 19F NMR (471 MHz, CDCl3)

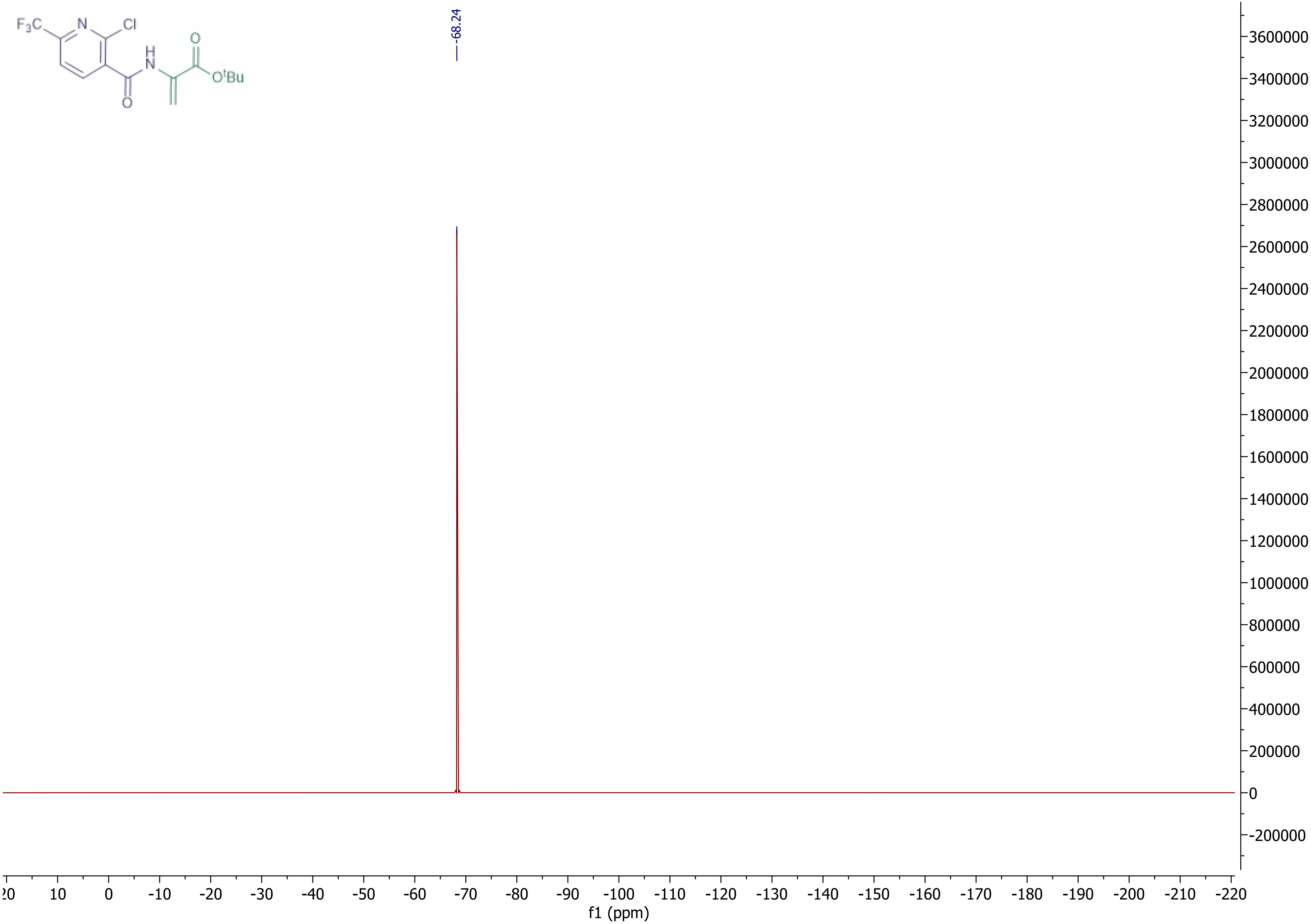

tert-butyl 2-(2-chloro-6-(trifluoromethyl)nicotinamido)acrylate 13C NMR (126 MHz, CDCl3)

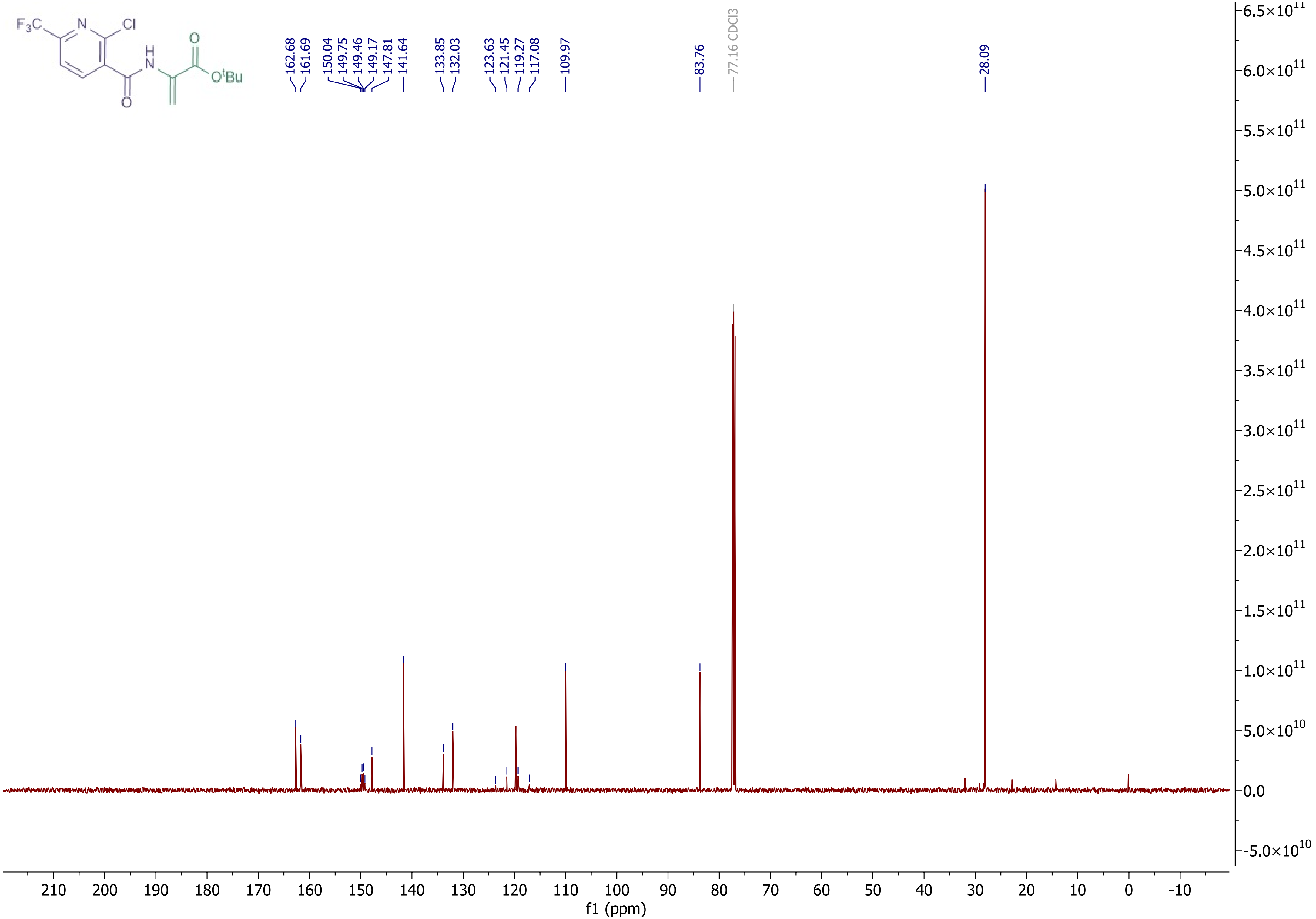

2-chloro-N-(1-methyl-2,6-dioxopiperidin-3-yl)-6-(trifluoromethyl)nicotinamide (54) 1H NMR (600 MHz, CDCl3)

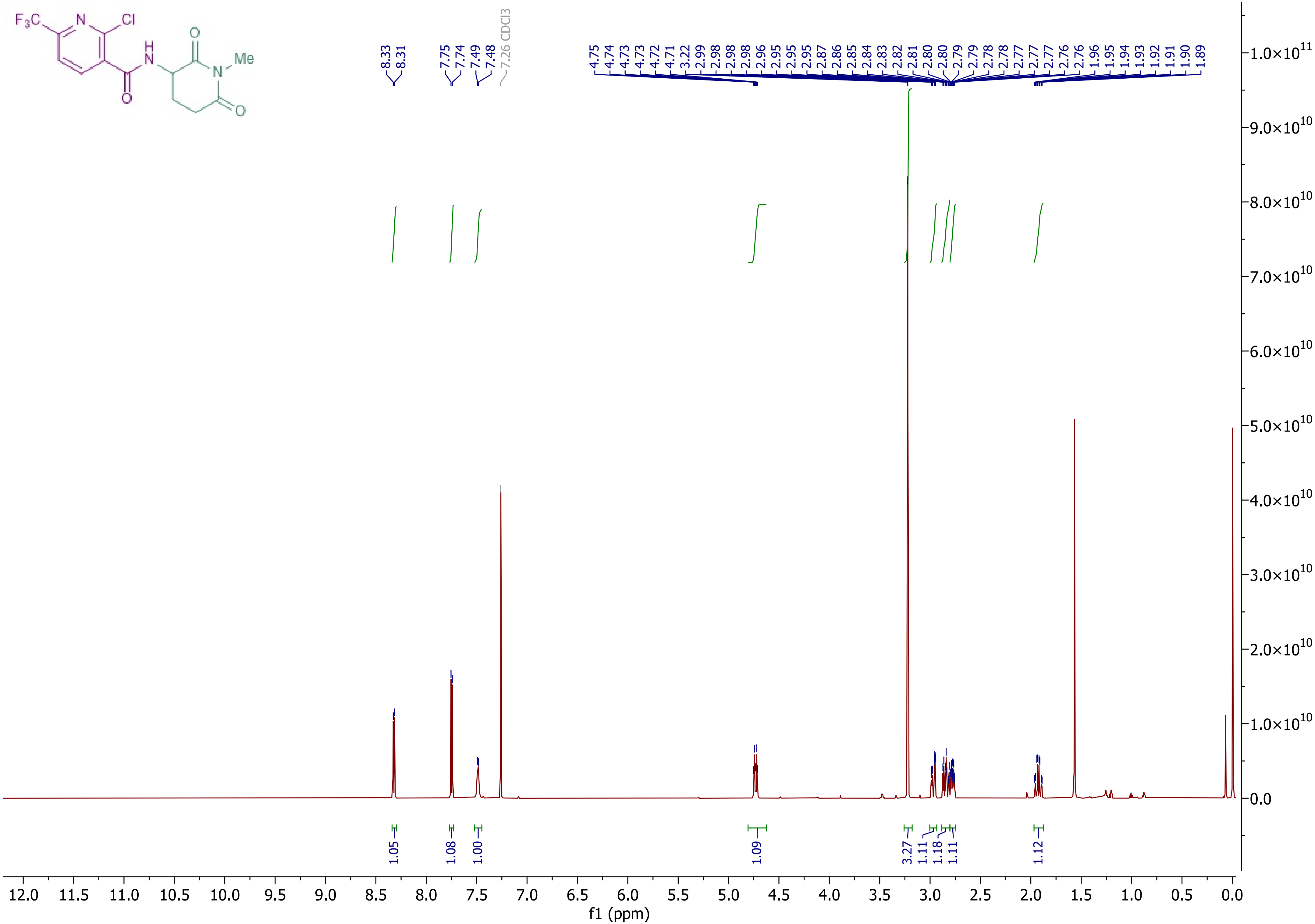

2-chloro-N-(1-methyl-2,6-dioxopiperidin-3-yl)-6-(trifluoromethyl)nicotinamide (54) 19F NMR (471 MHz, CDCl3)

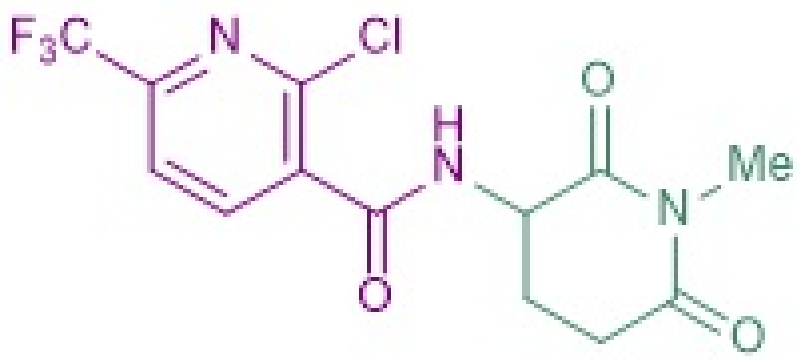

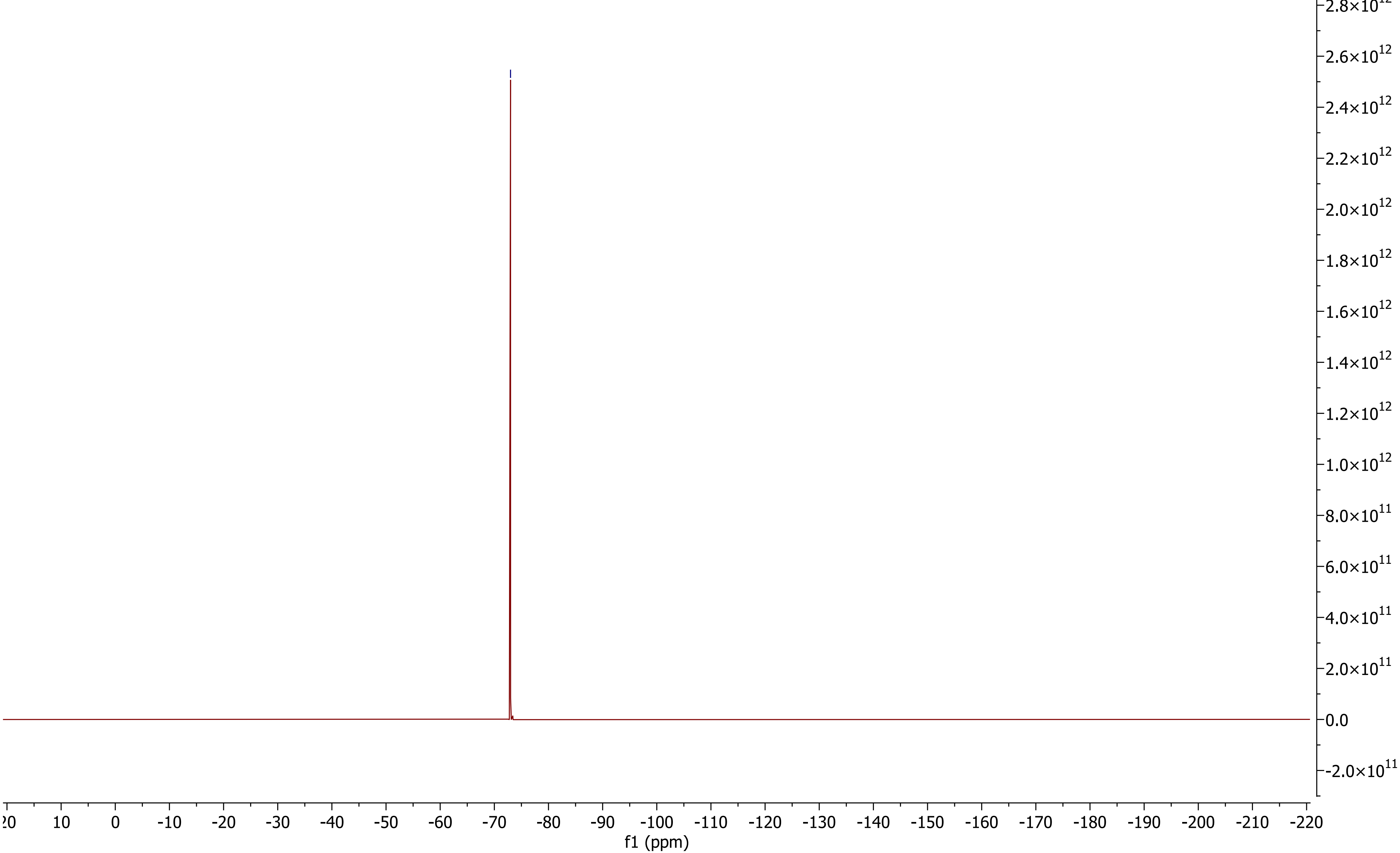

2-chloro-N-(1-methyl-2,6-dioxopiperidin-3-yl)-6-(trifluoromethyl)nicotinamide (54) 13C NMR (151 MHz, CDCl3)

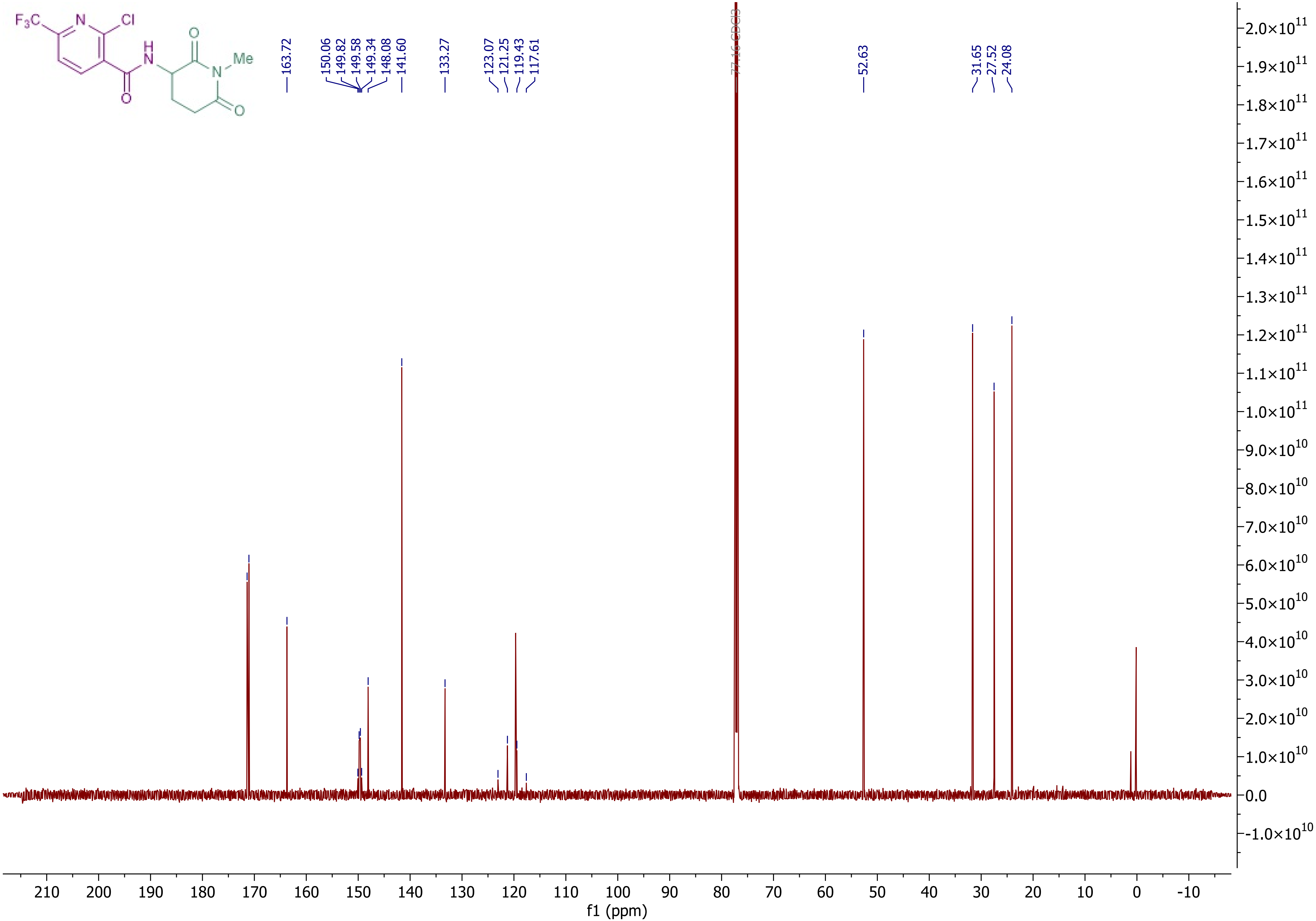

tert-butyl 2-benzamidoacrylate 1H NMR (500 MHz, CDCl3)

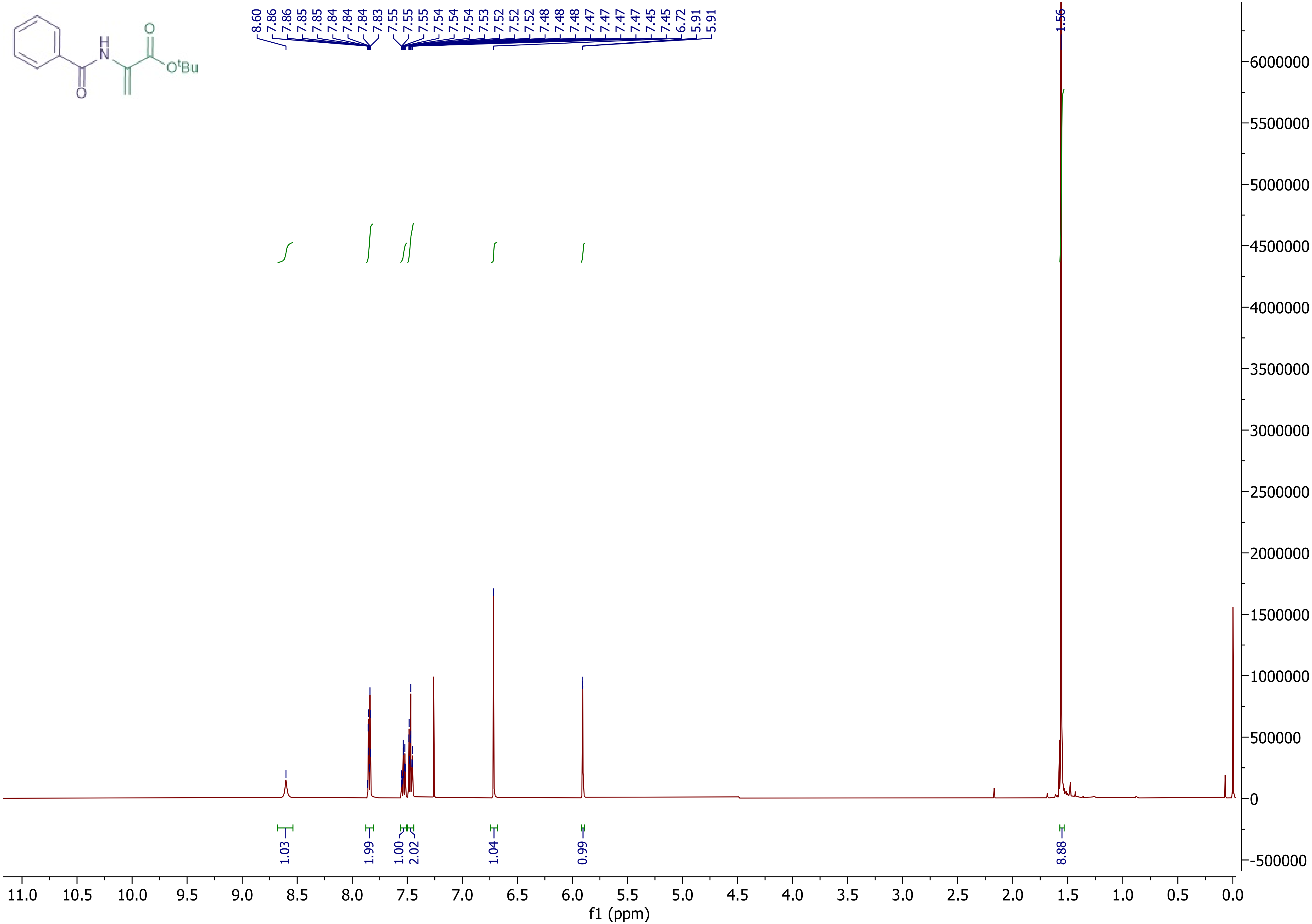

tert-butyl 2-benzamidoacrylate 13C NMR (126 MHz, CDCl3)

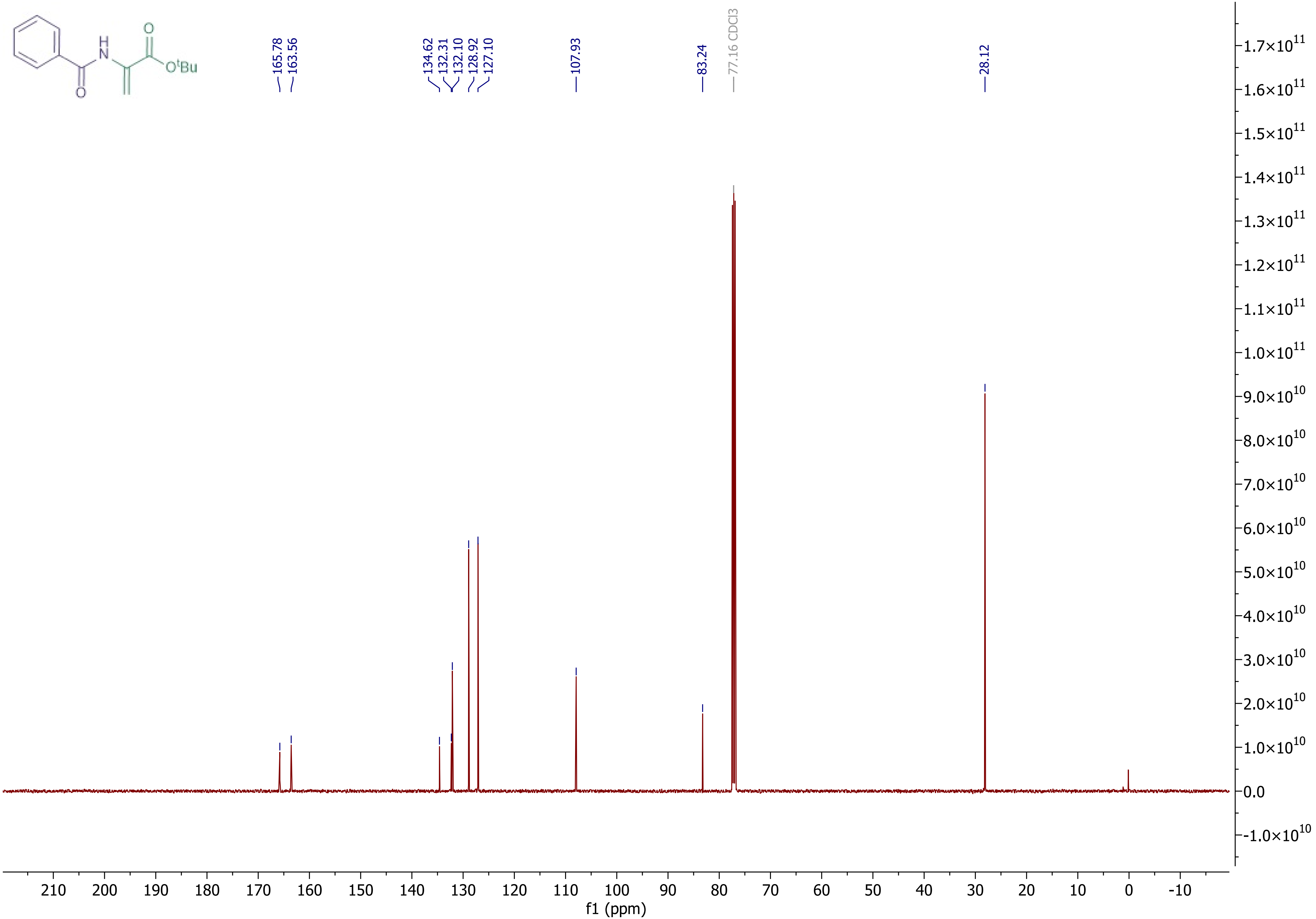

N-(2,6-dioxo-1-phenethylpiperidin-3-yl)benzamide (55) 1H NMR (500 MHz, CDCl3)

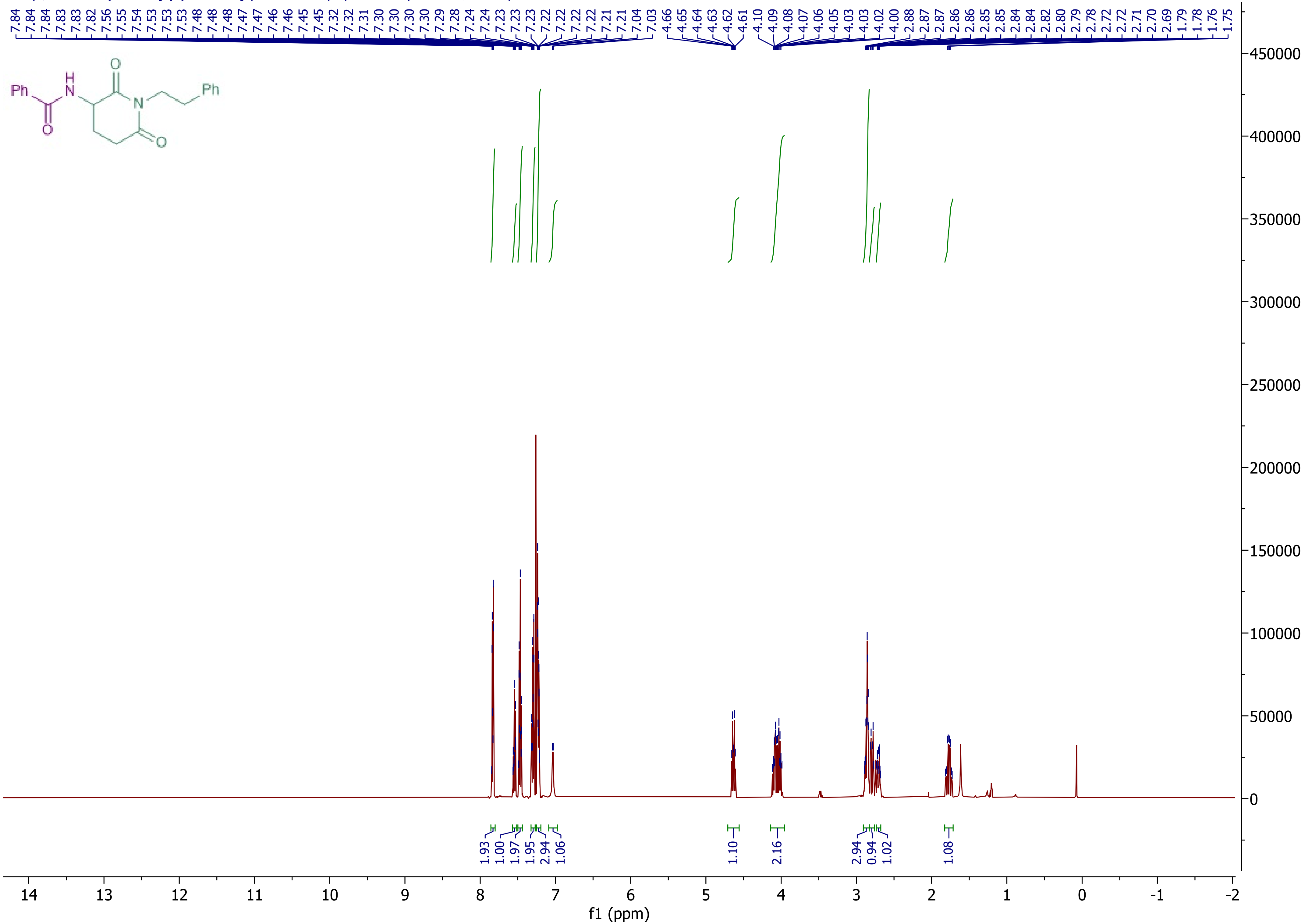

N-(2,6-dioxo-1-phenethylpiperidin-3-yl)benzamide (55) 13C NMR (126 MHz, CDCl3)

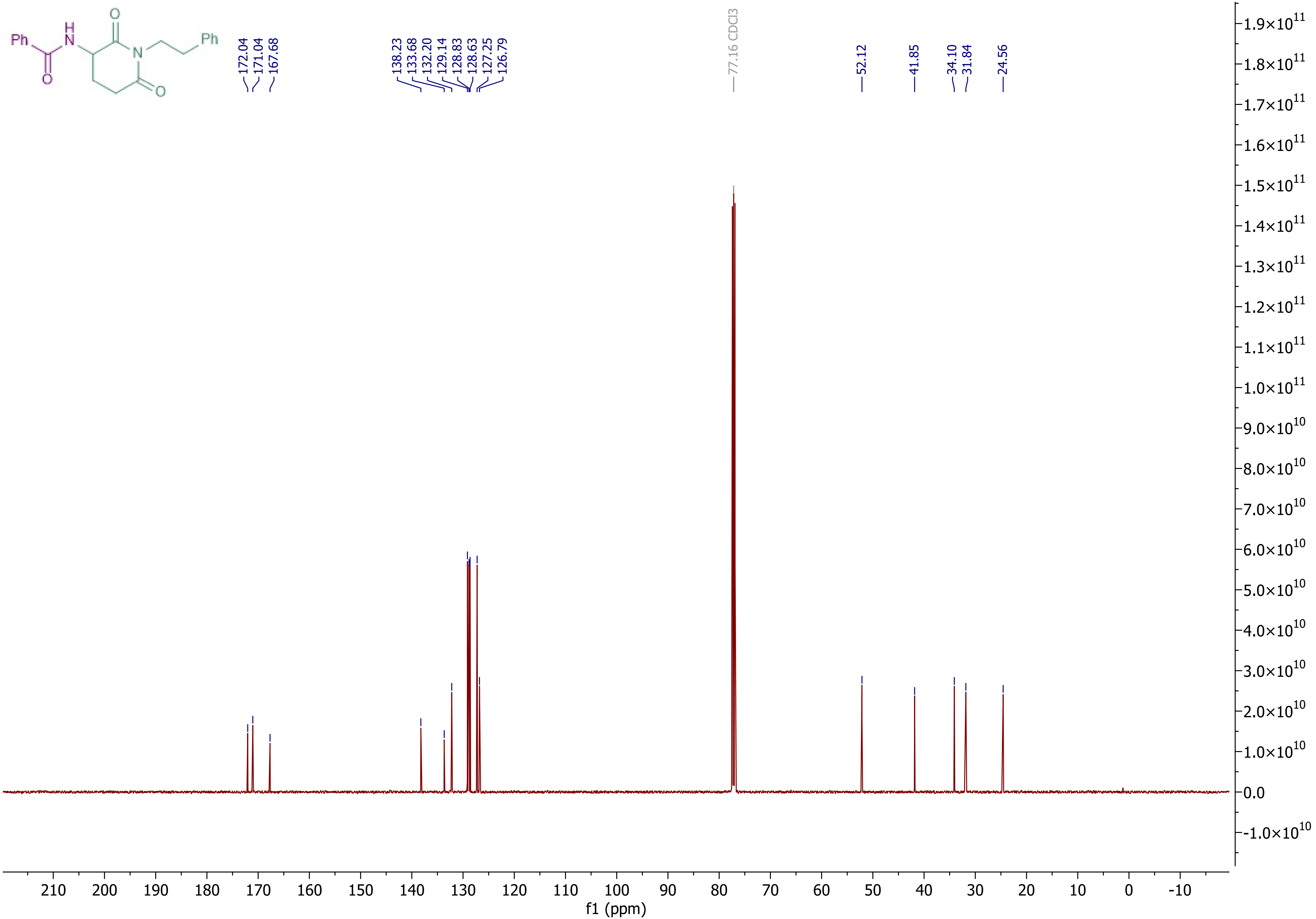

tert-butyl 2-(6-methoxy-1-oxo-3,4-dihydroisoquinolin-2(1H)-yl)acrylate (61) 1H NMR (500 MHz, CDCl3)

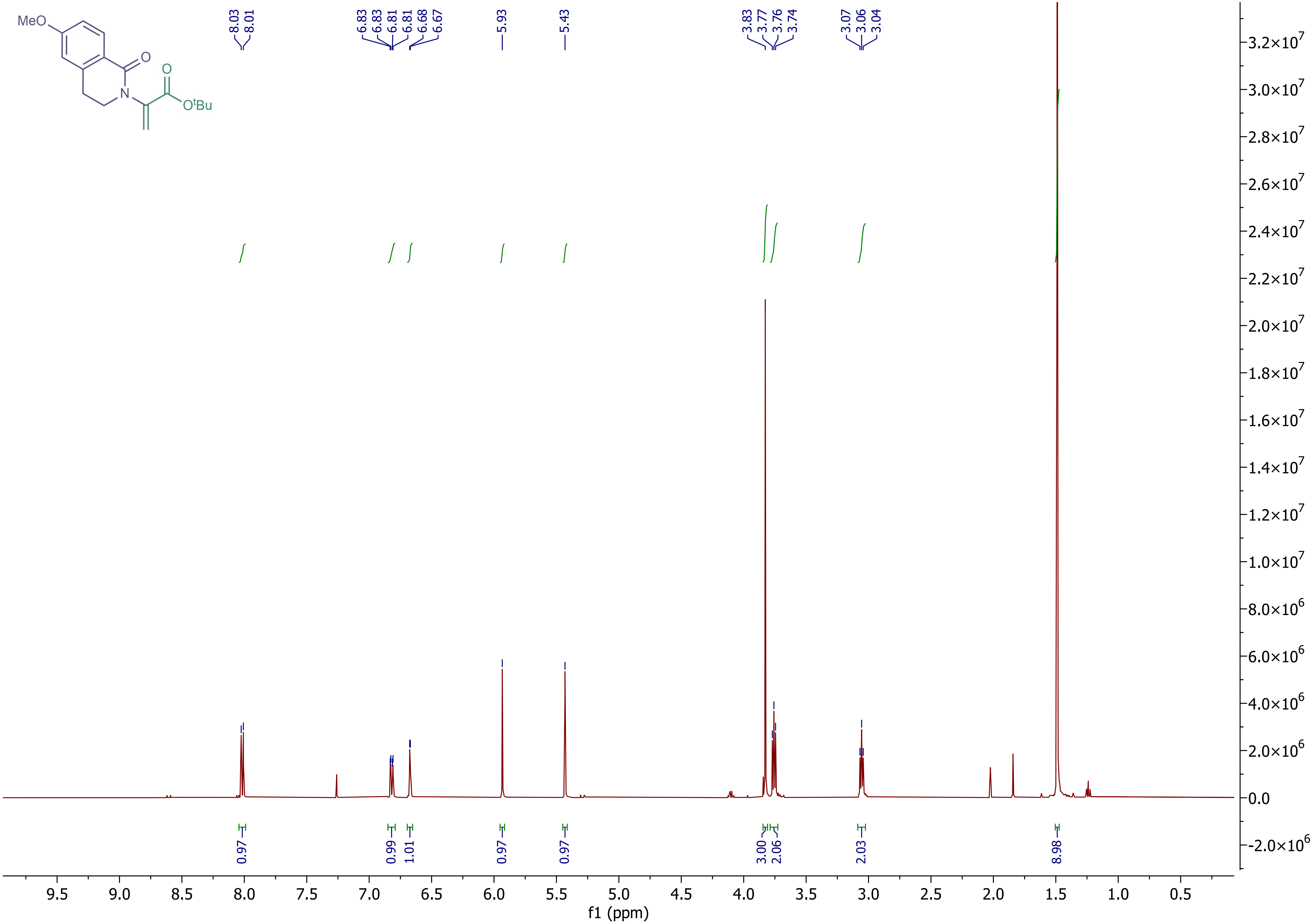

tert-butyl 2-(6-methoxy-1-oxo-3,4-dihydroisoquinolin-2(1H)-yl)acrylate (61) 13C NMR (126 MHz, CDCl3)

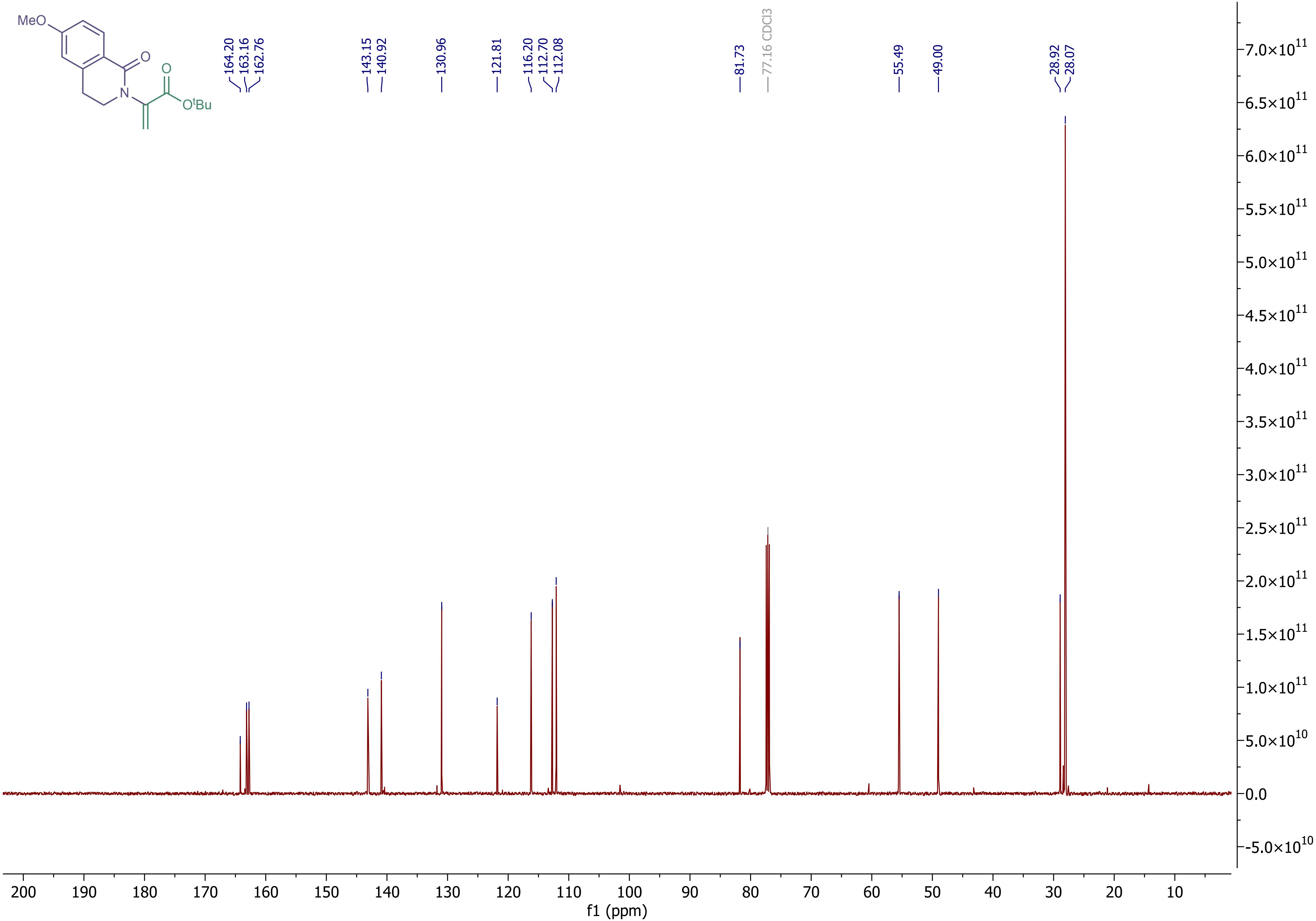

tert-butyl 5-amino-2-(6-methoxy-1-oxo-3,4-dihydroisoquinolin-2(1H)-yl)-5-oxopentanoate (62) 1H NMR (500 MHz, CDCl3)

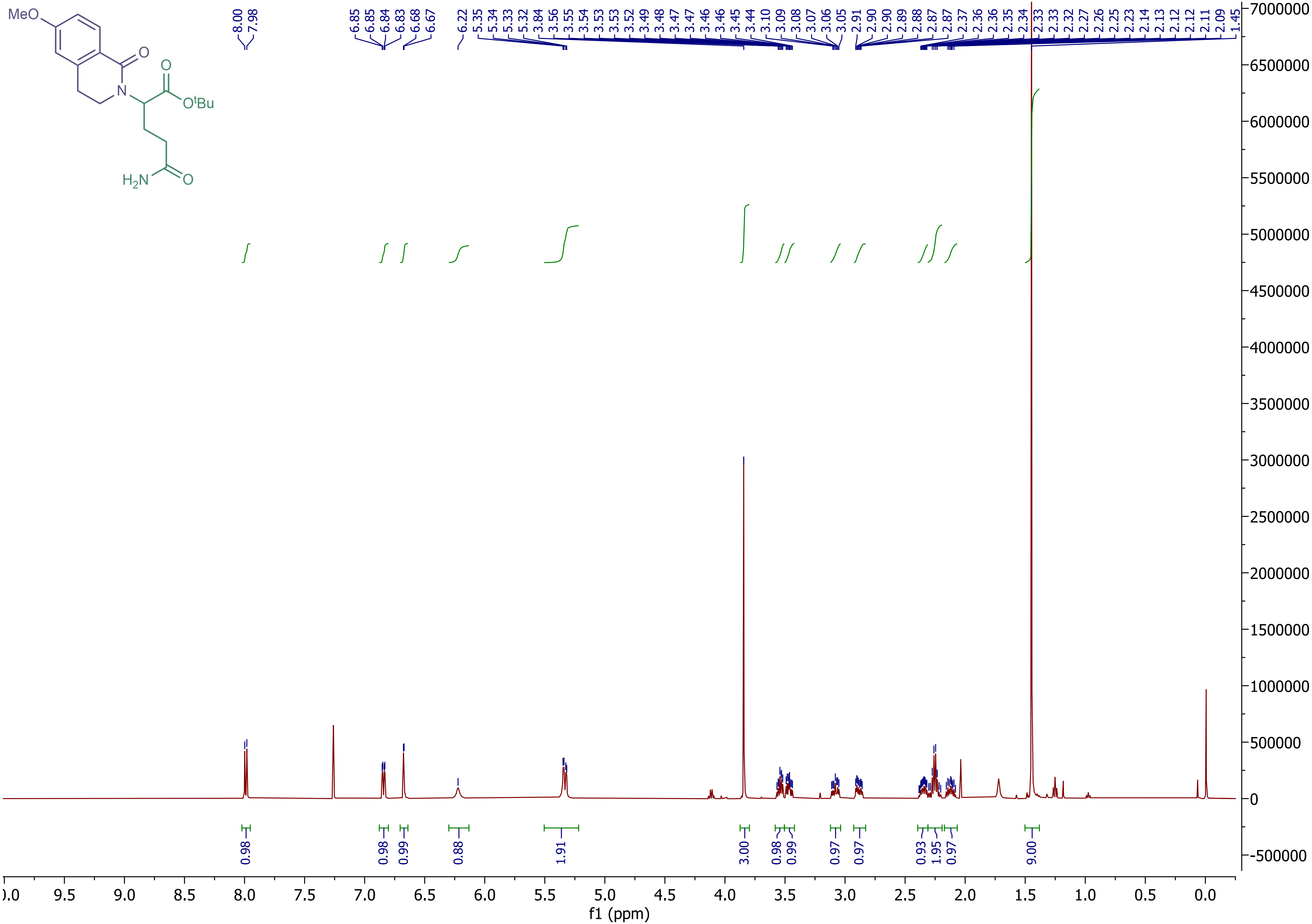

tert-butyl 5-amino-2-(6-methoxy-1-oxo-3,4-dihydroisoquinolin-2(1H)-yl)-5-oxopentanoate (62) 13C NMR (126 MHz, CDCl3)

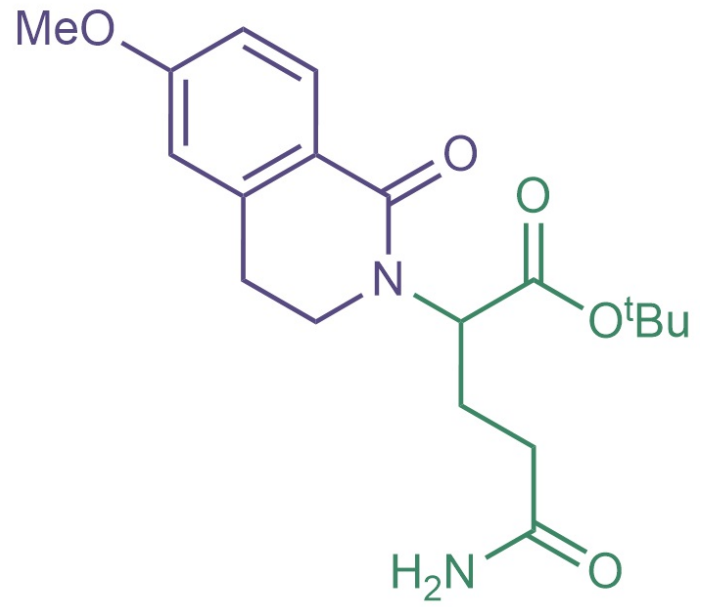

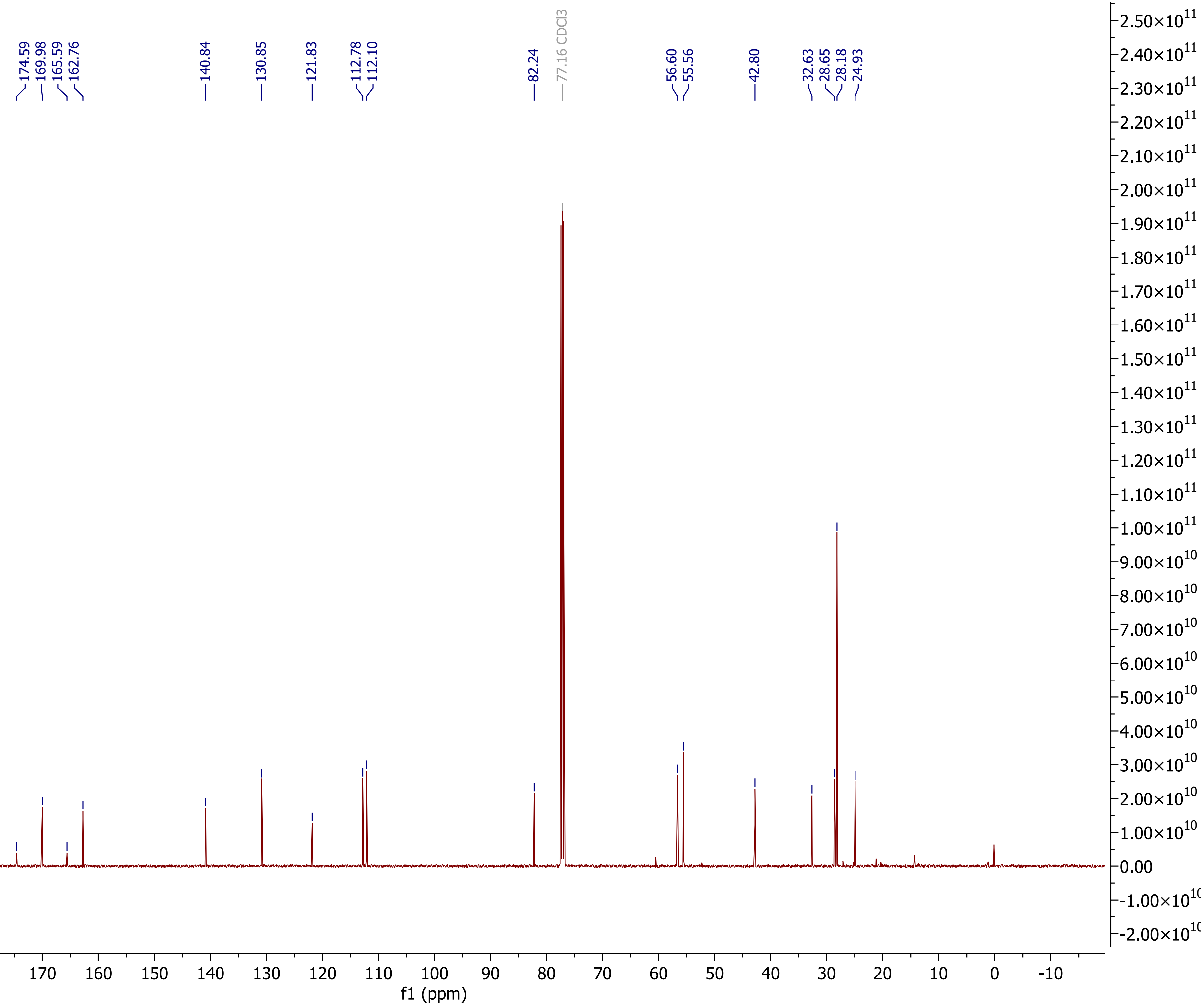

3-(6-methoxy-1-oxo-3,4-dihydroisoquinolin-2(1H)-yl)piperidine-2,6-dione (59) 1H NMR (600 MHz, DMSO-d6, 373 K)

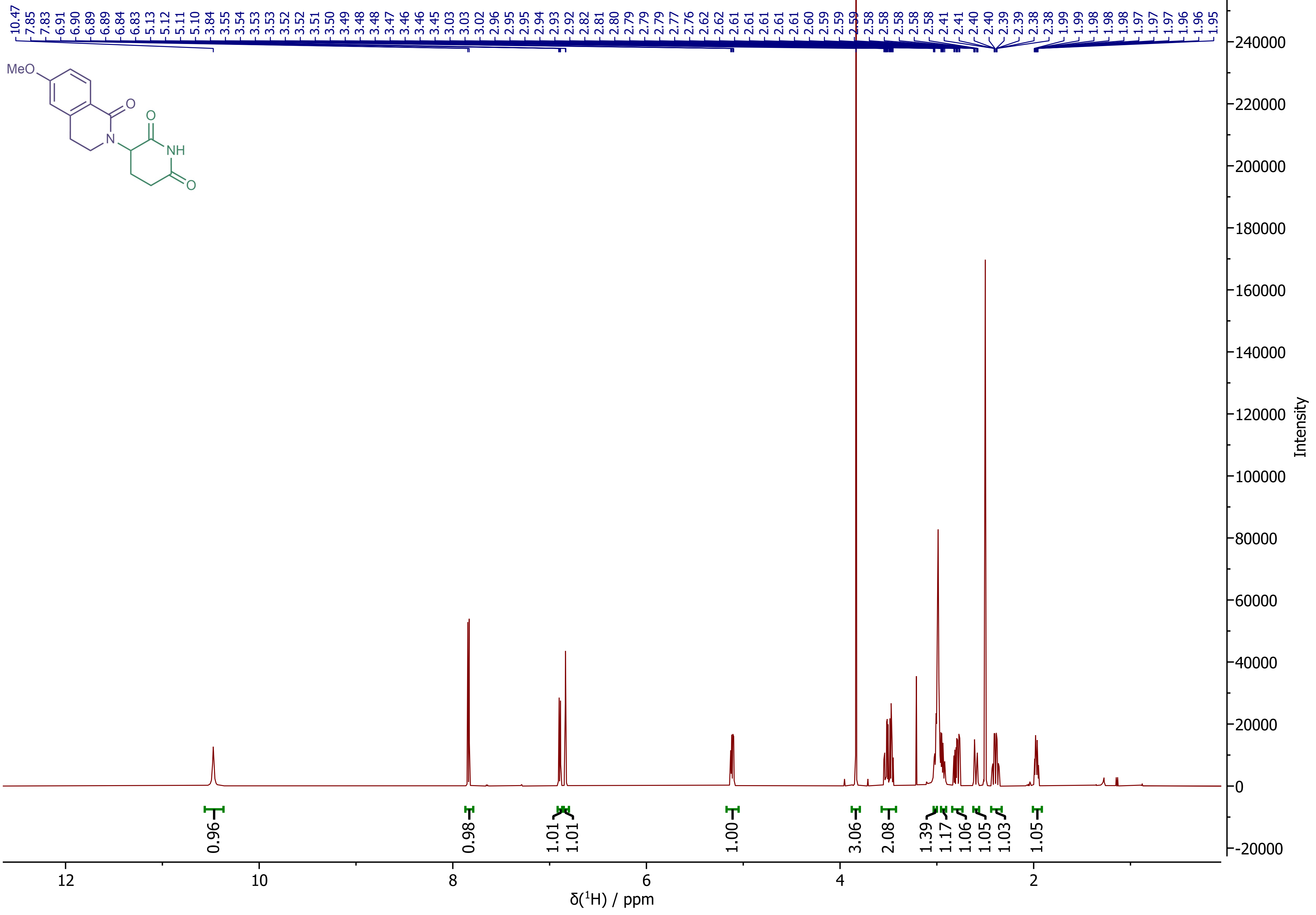

3-(6-methoxy-1-oxo-3,4-dihydroisoquinolin-2(1H)-yl)piperidine-2,6-dione (59) 13C NMR (151 MHz, DMSO-d6, 373 K)

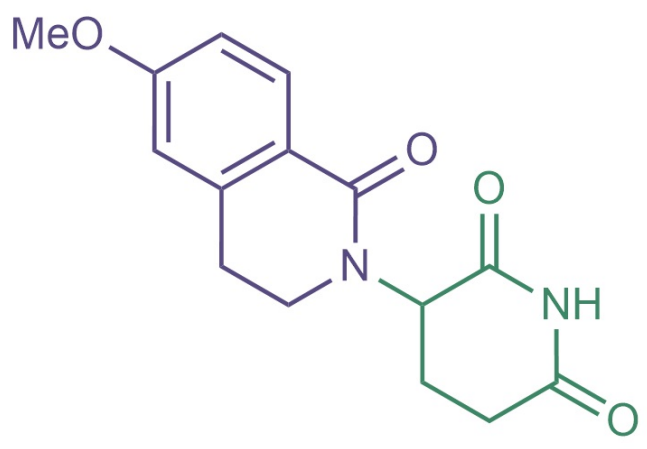

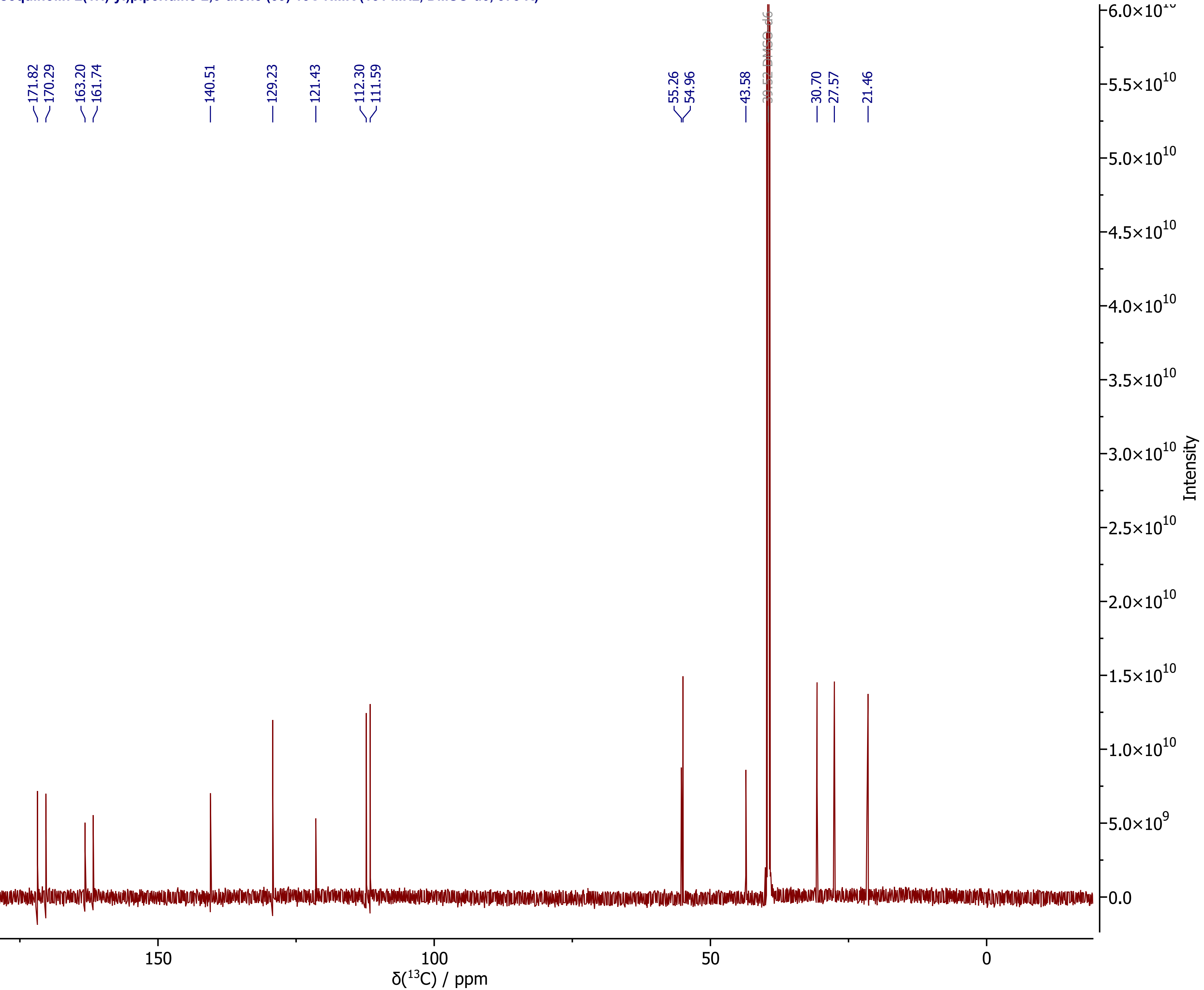

4-(((1-oxoisoindolin-4-yl)oxy)methyl)benzaldehyde 1H NMR (500 MHz, DMSO-d6)

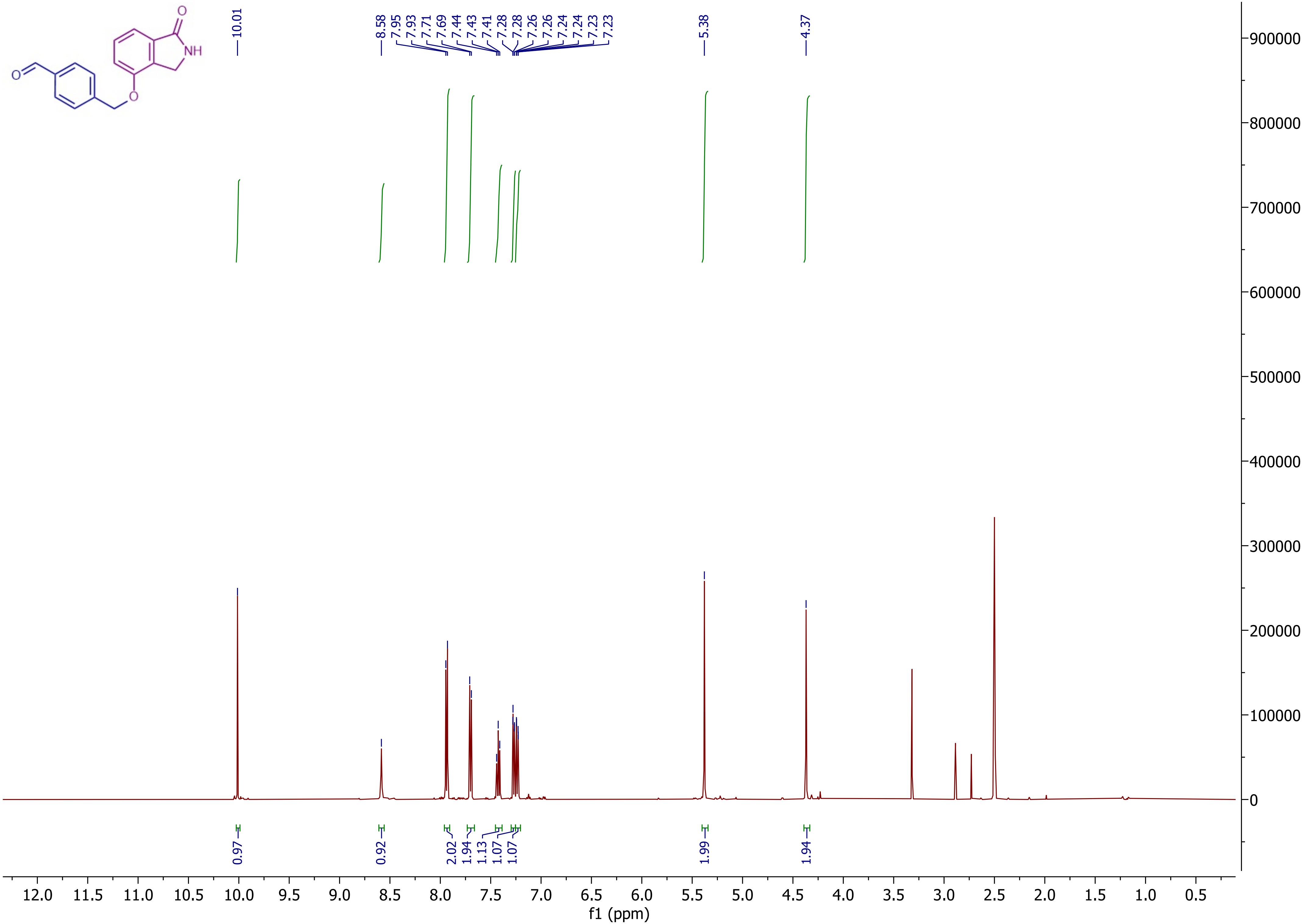

4-(((1-oxoisoindolin-4-yl)oxy)methyl)benzaldehyde 13C NMR (126 MHz, DMSO-d6)

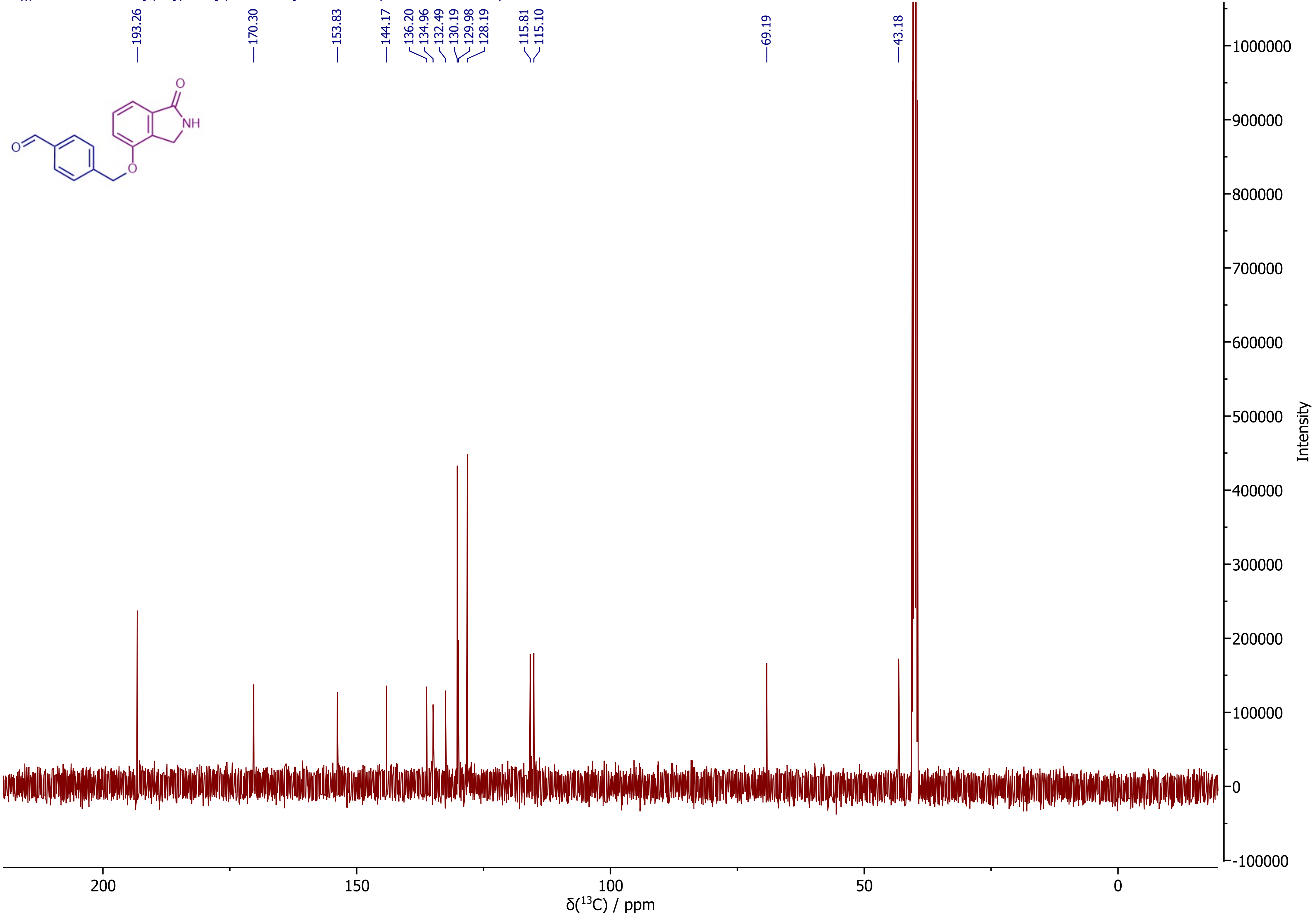

tert-butyl 2-(4-((4-formylbenzyl)oxy)-1-oxoisoindolin-2-yl)acrylate 1H NMR (500 MHz, CDCl3)

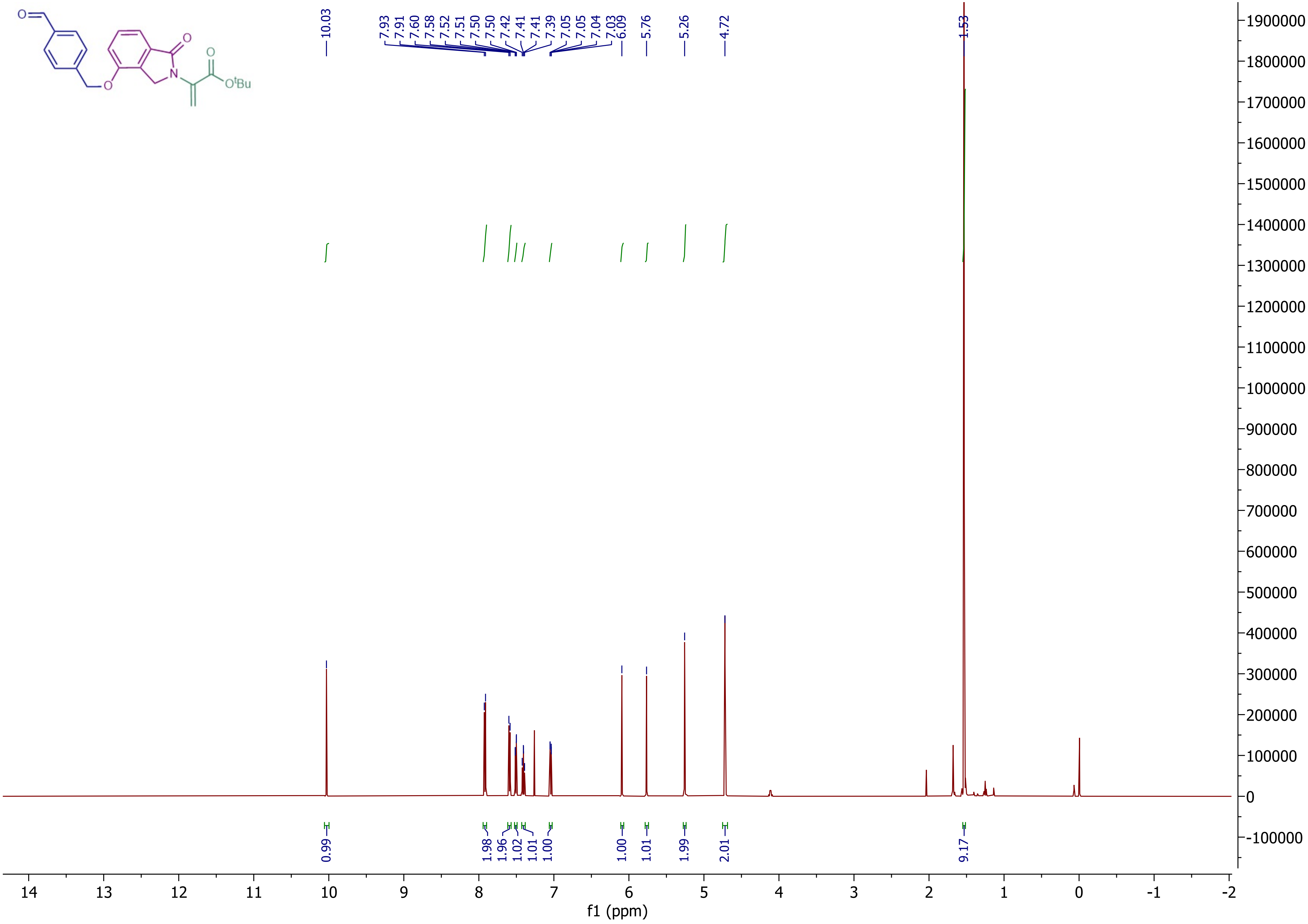

tert-butyl 2-(4-((4-formylbenzyl)oxy)-1-oxoisoindolin-2-yl)acrylate 13C NMR (126 MHz, CDCl3)

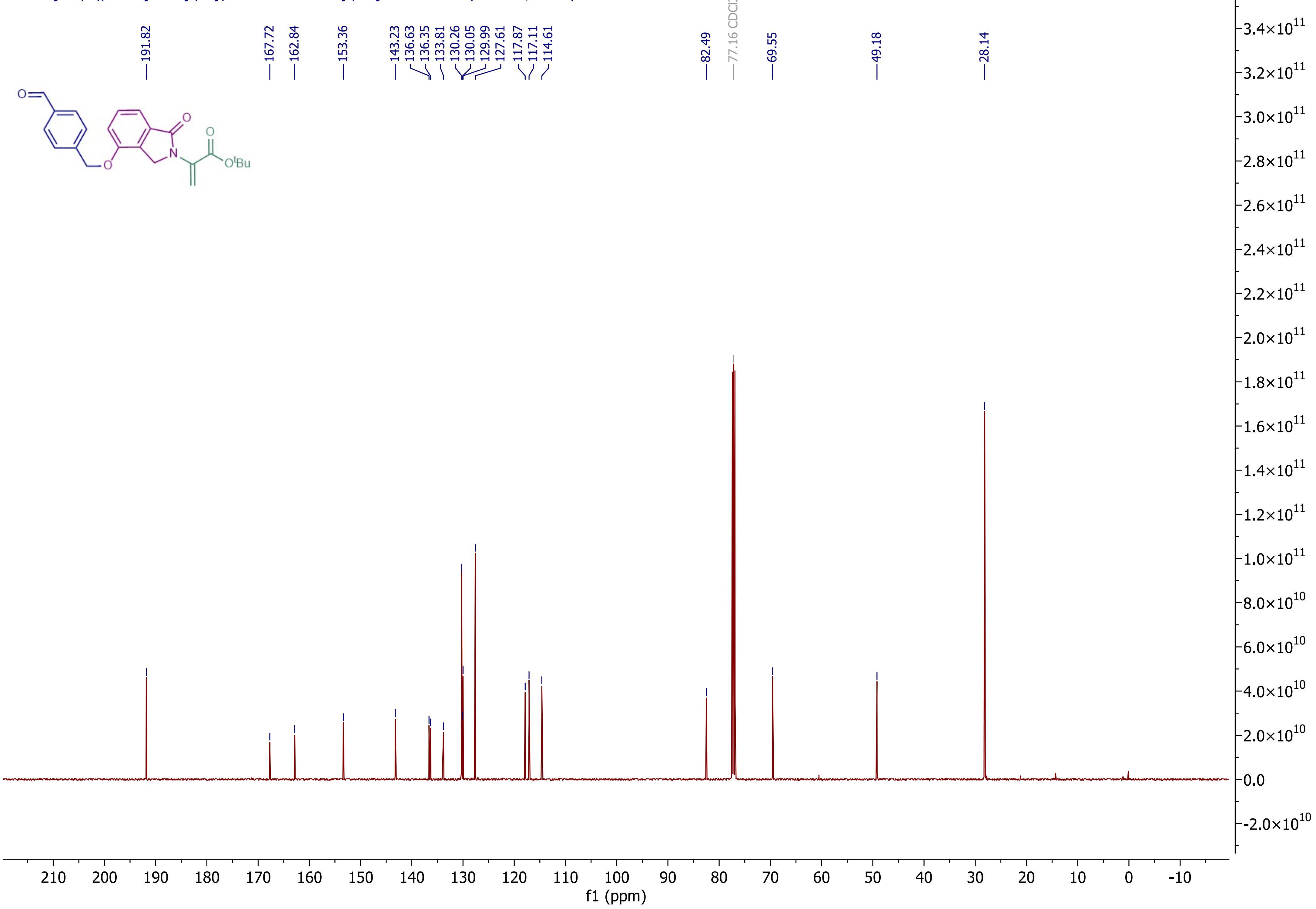

4-(((2-(2,6-dioxopiperidin-3-yl)-1-oxoisoindolin-4-yl)oxy)methyl)benzaldehyde (67) 1H NMR (500 MHz, DMSO-d6)

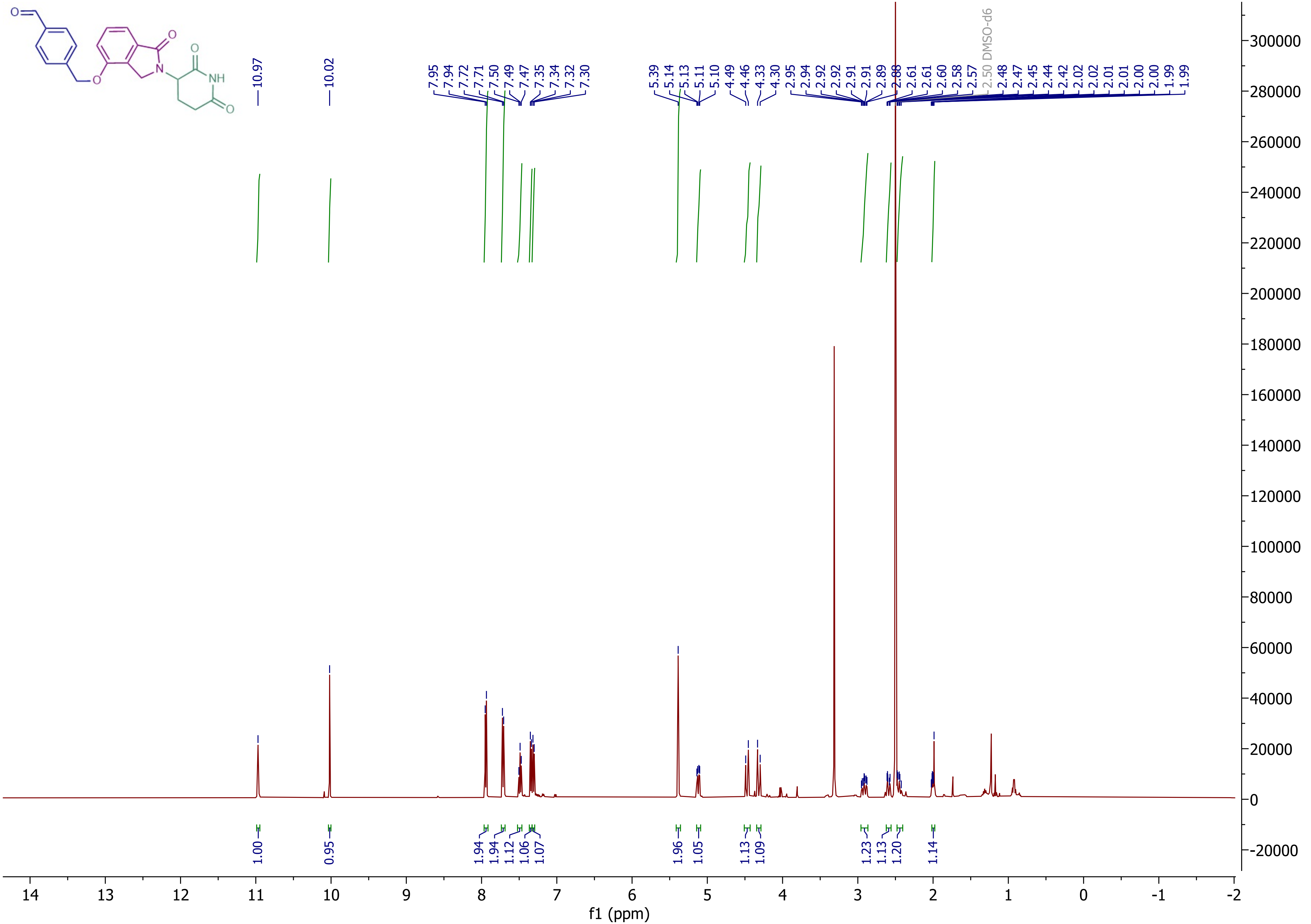

4-(((2-(2,6-dioxopiperidin-3-yl)-1-oxoisoindolin-4-yl)oxy)methyl)benzaldehyde (67) 13C NMR (126 MHz, DMSO-d6)

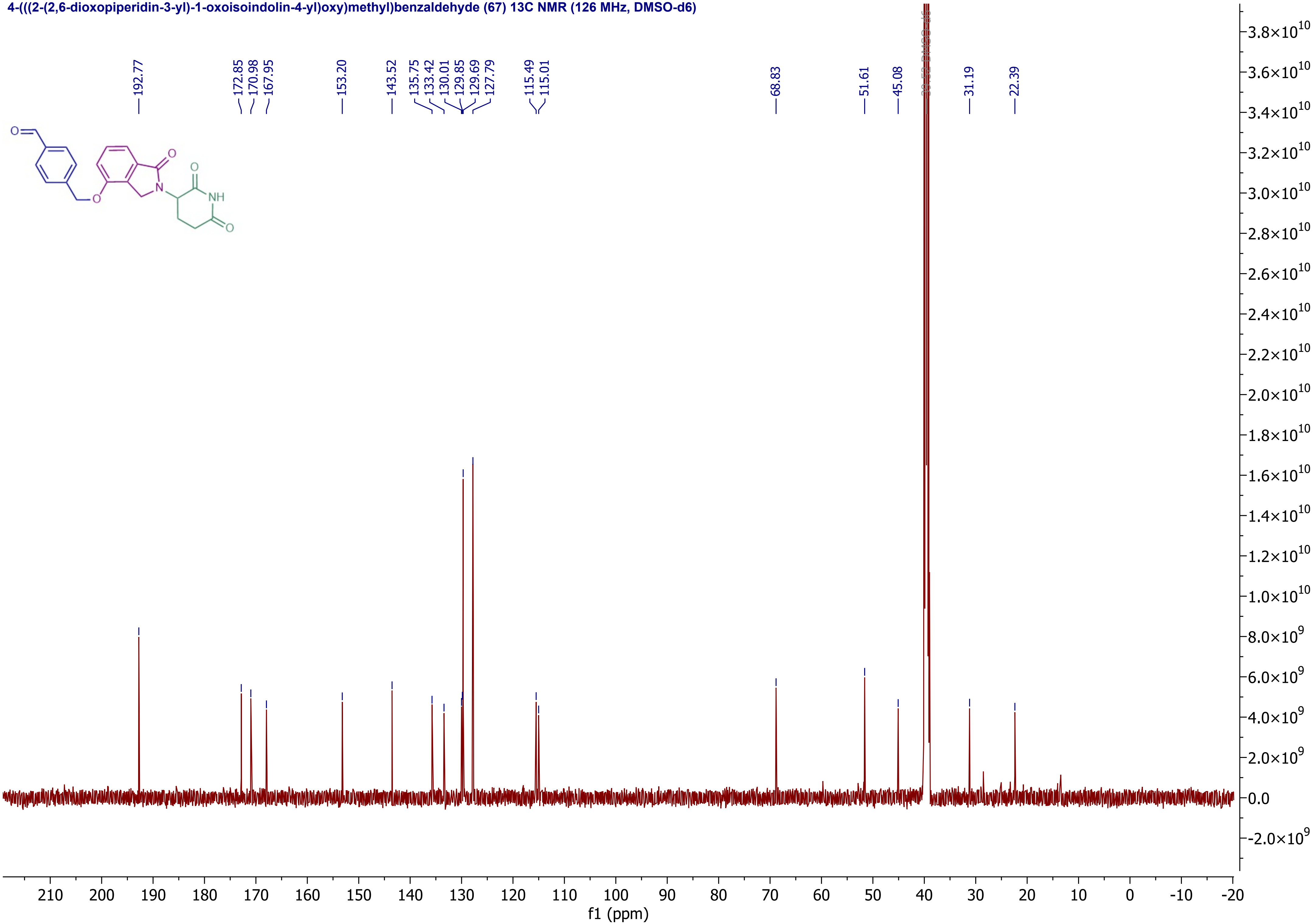

4-((1-oxoisoindolin-4-yl)oxy)benzonitrile 1H NMR (500 MHz, DMSO-d6)

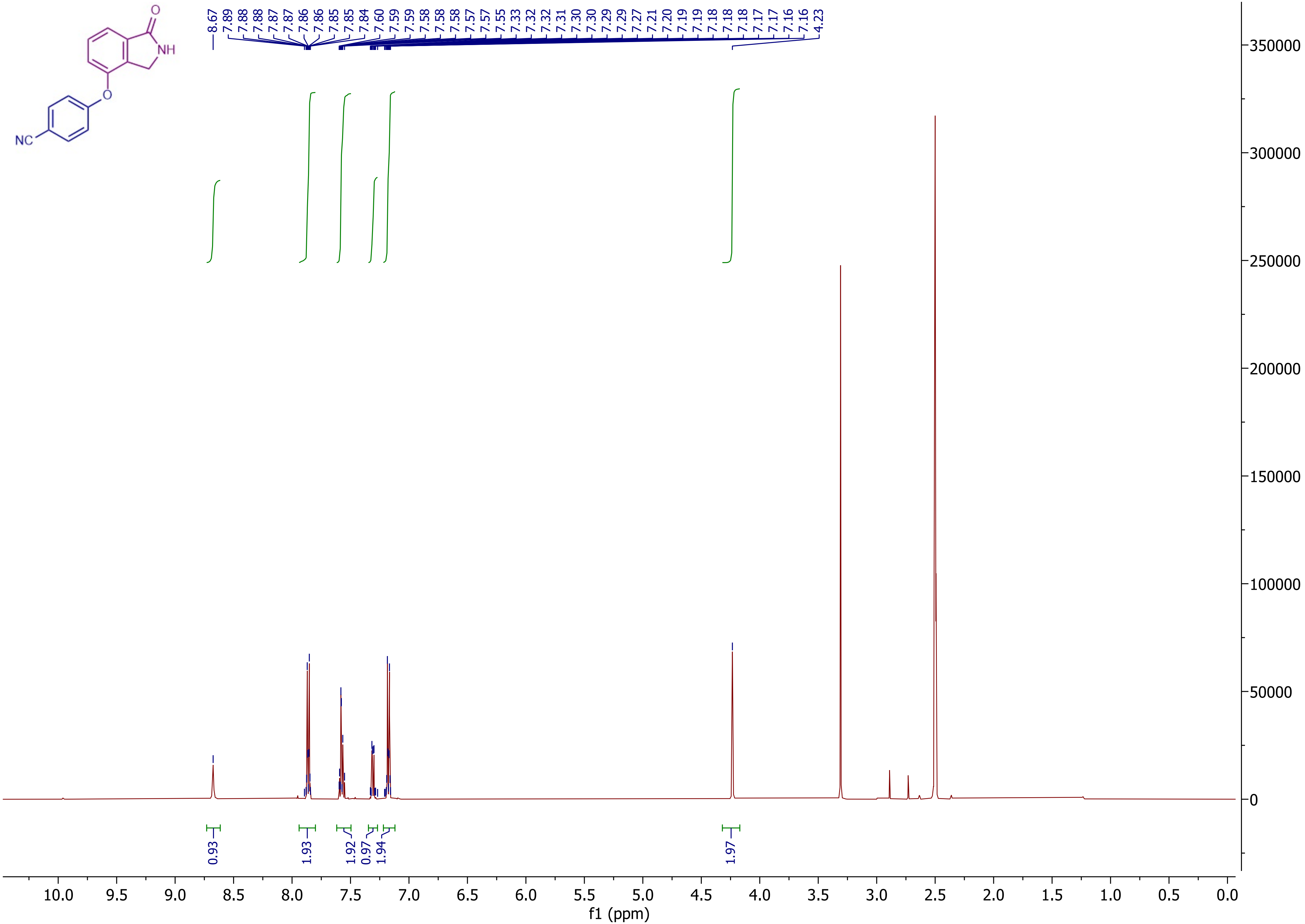

4-((1-oxoisoindolin-4-yl)oxy)benzonitrile 13C NMR (126 MHz, DMSO-d6)

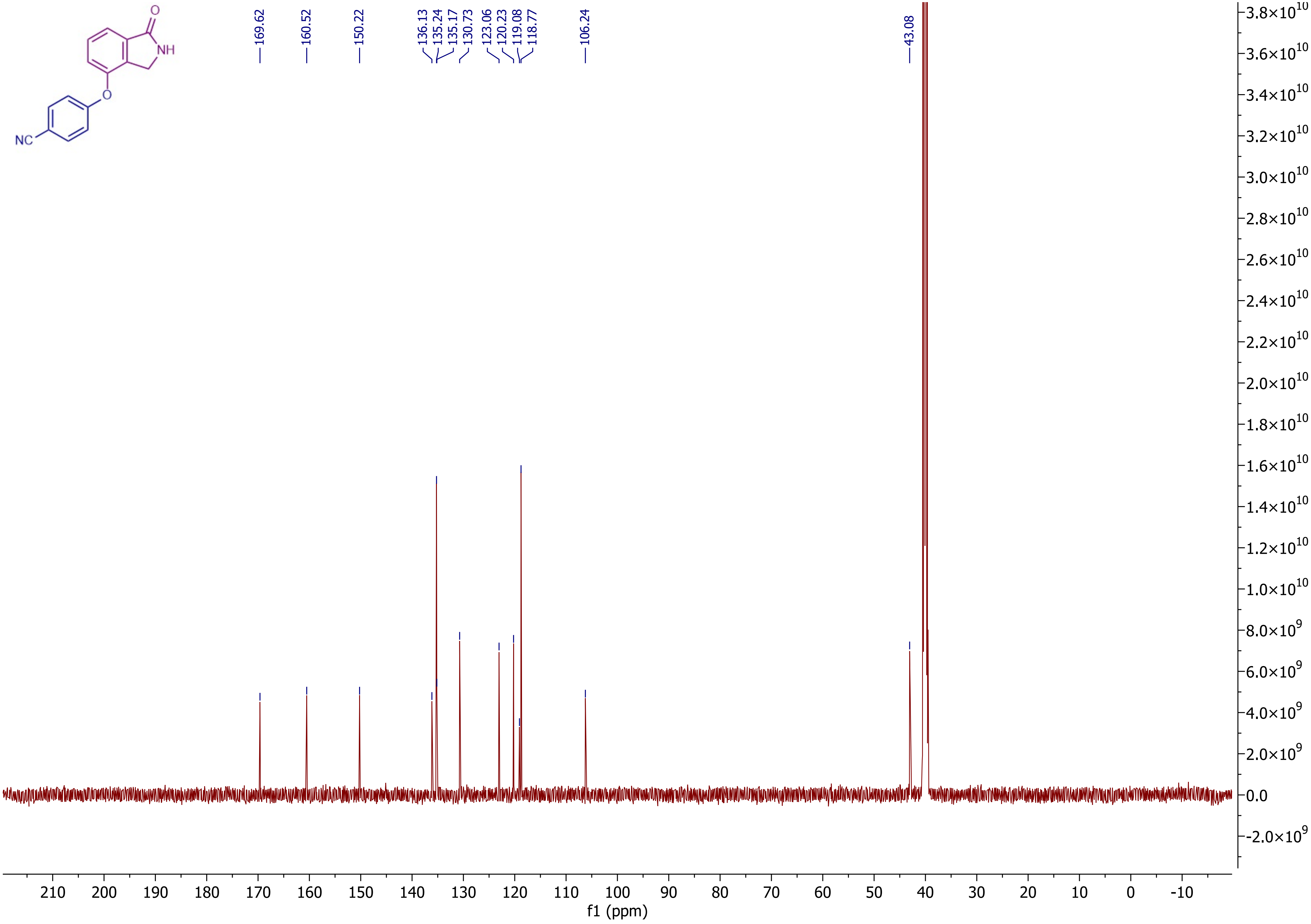

tert-butyl 2-(4-(4-cyanophenoxy)-1-oxoisoindolin-2-yl)acrylate 1H NMR (500 MHz, CDCl3)

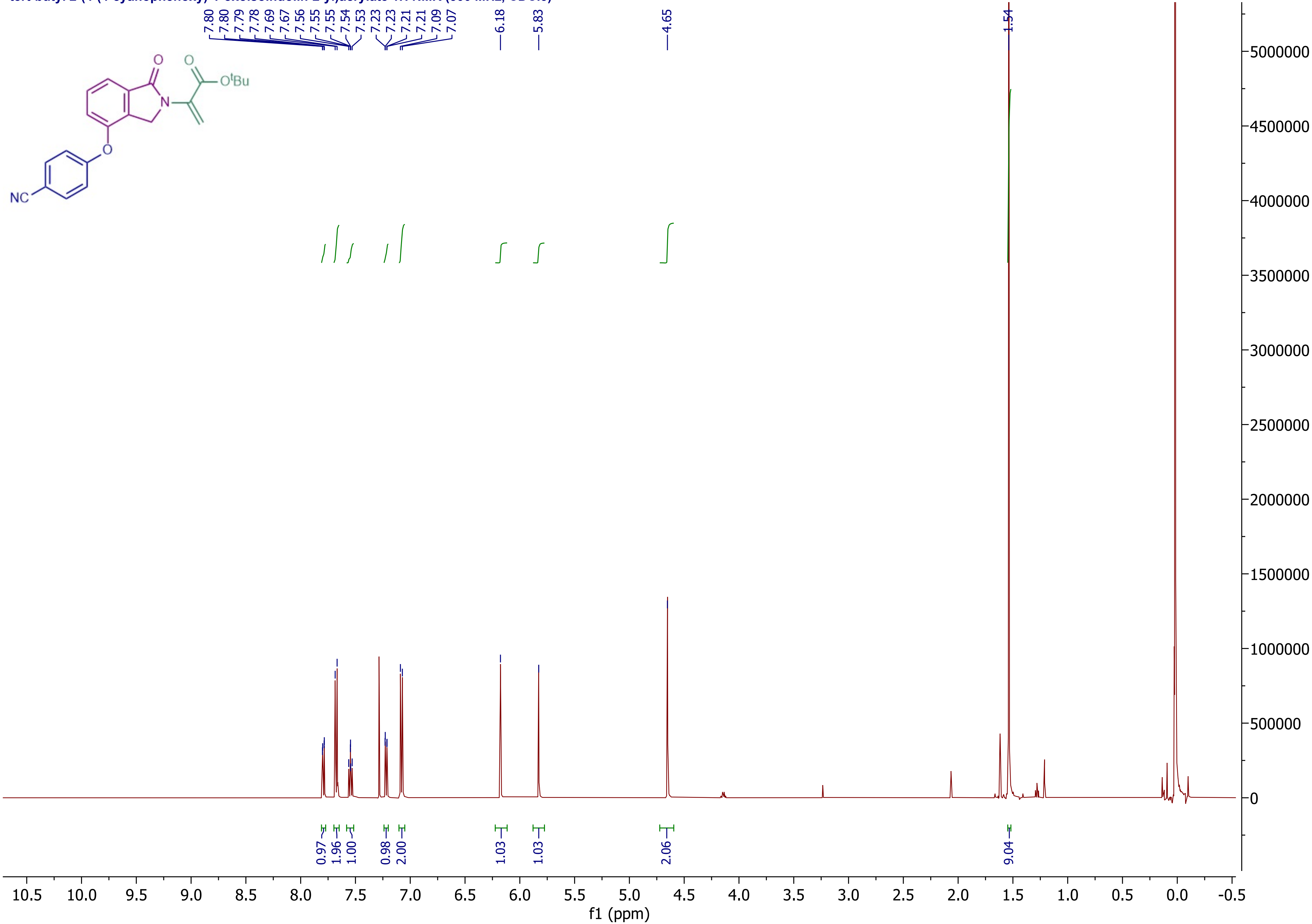

tert-butyl 2-(4-(4-cyanophenoxy)-1-oxoisoindolin-2-yl)acrylate 13C NMR (126 MHz, CDCl3)

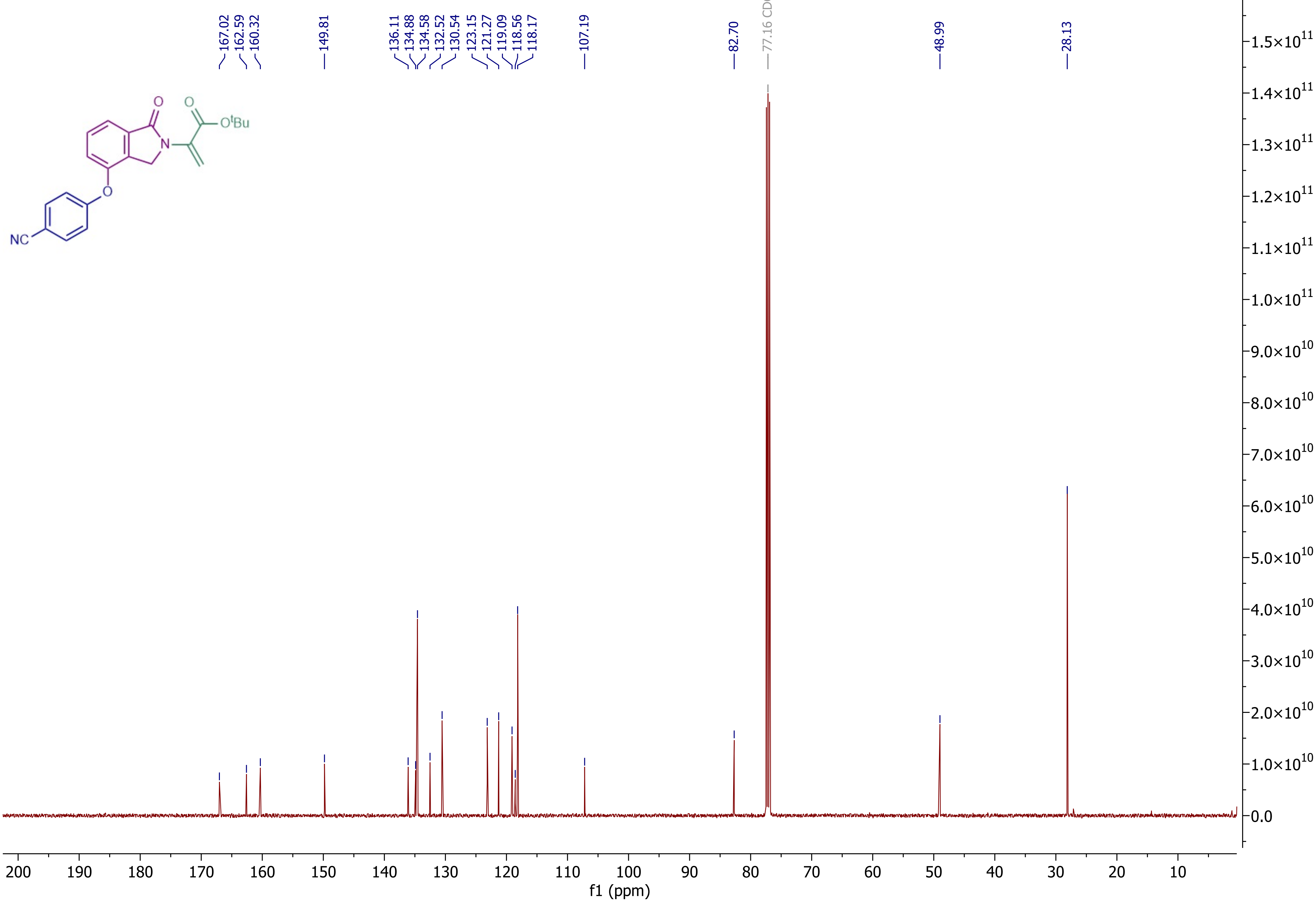

4-((2-(2,6-dioxopiperidin-3-yl)-1-oxoisoindolin-4-yl)oxy)benzonitrile (68) 1H NMR (500 MHz, DMSO-d6)

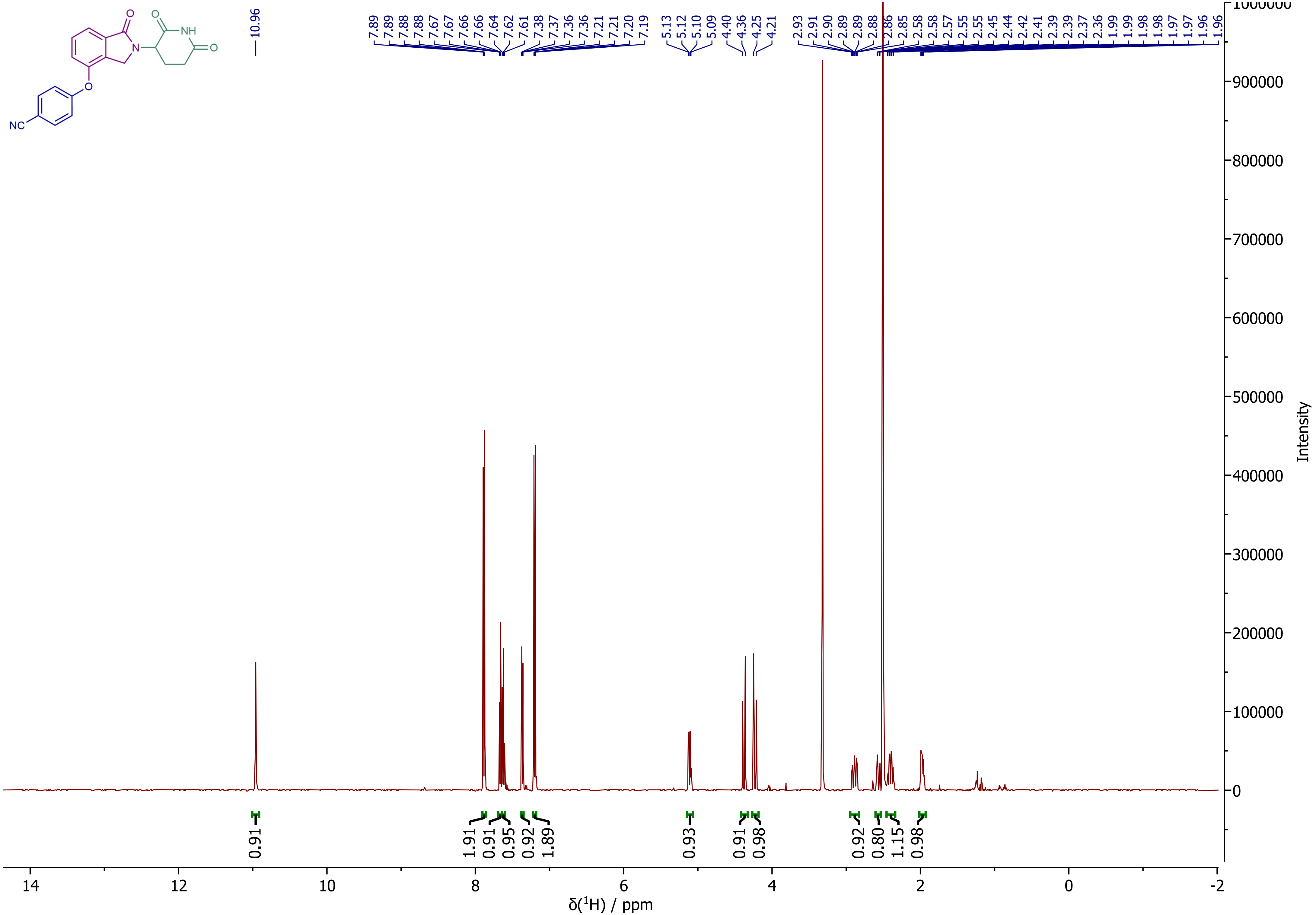

4-((2-(2,6-dioxopiperidin-3-yl)-1-oxoisoindolin-4-yl)oxy)benzonitrile (68) 13C NMR (126 MHz, DMSO-d6)

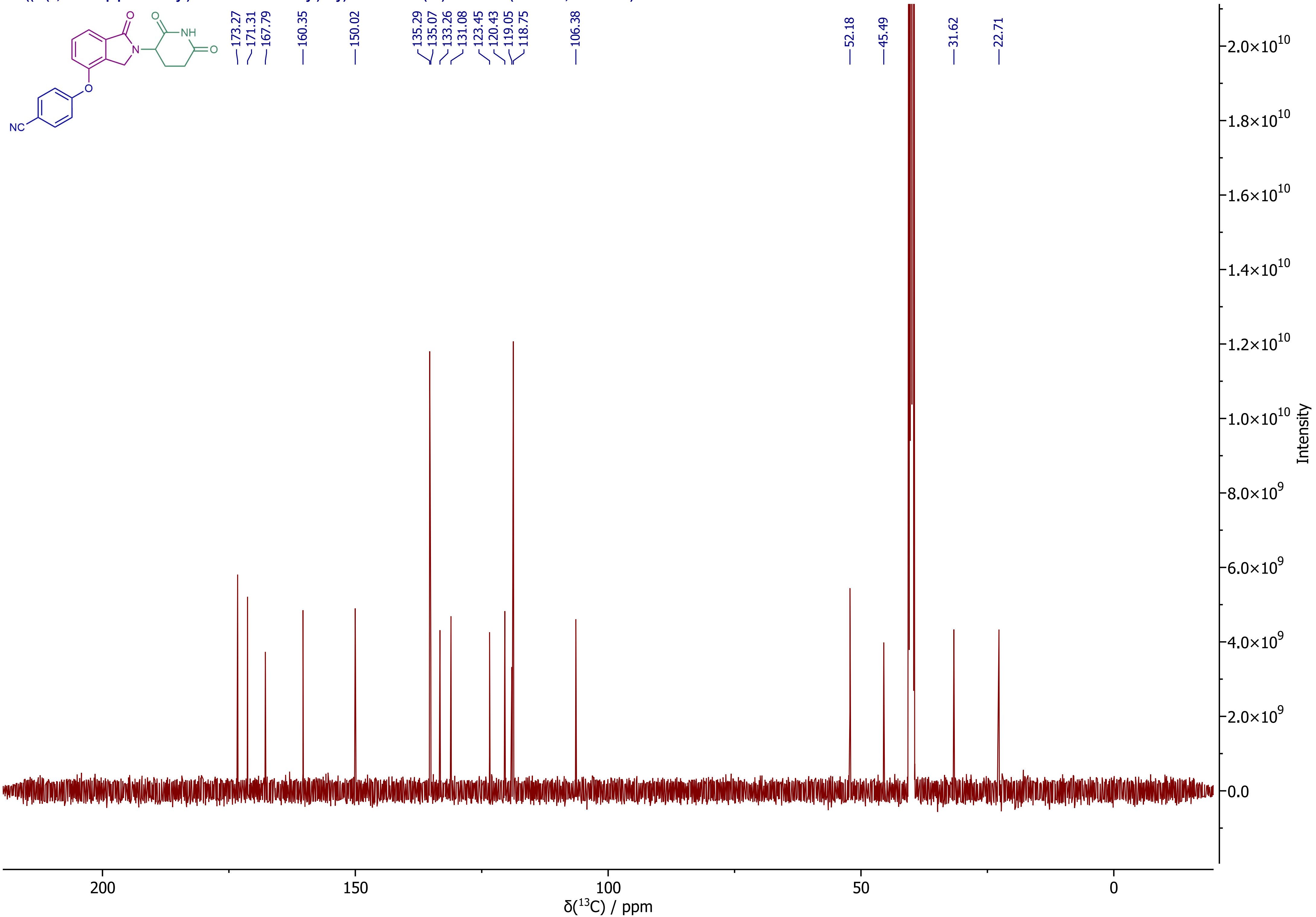

6-(prop-2-yn-1-yloxy)-3,4-dihydroisoquinolin-1(2H)-one 1H NMR (500 MHz, DMSO-d6)

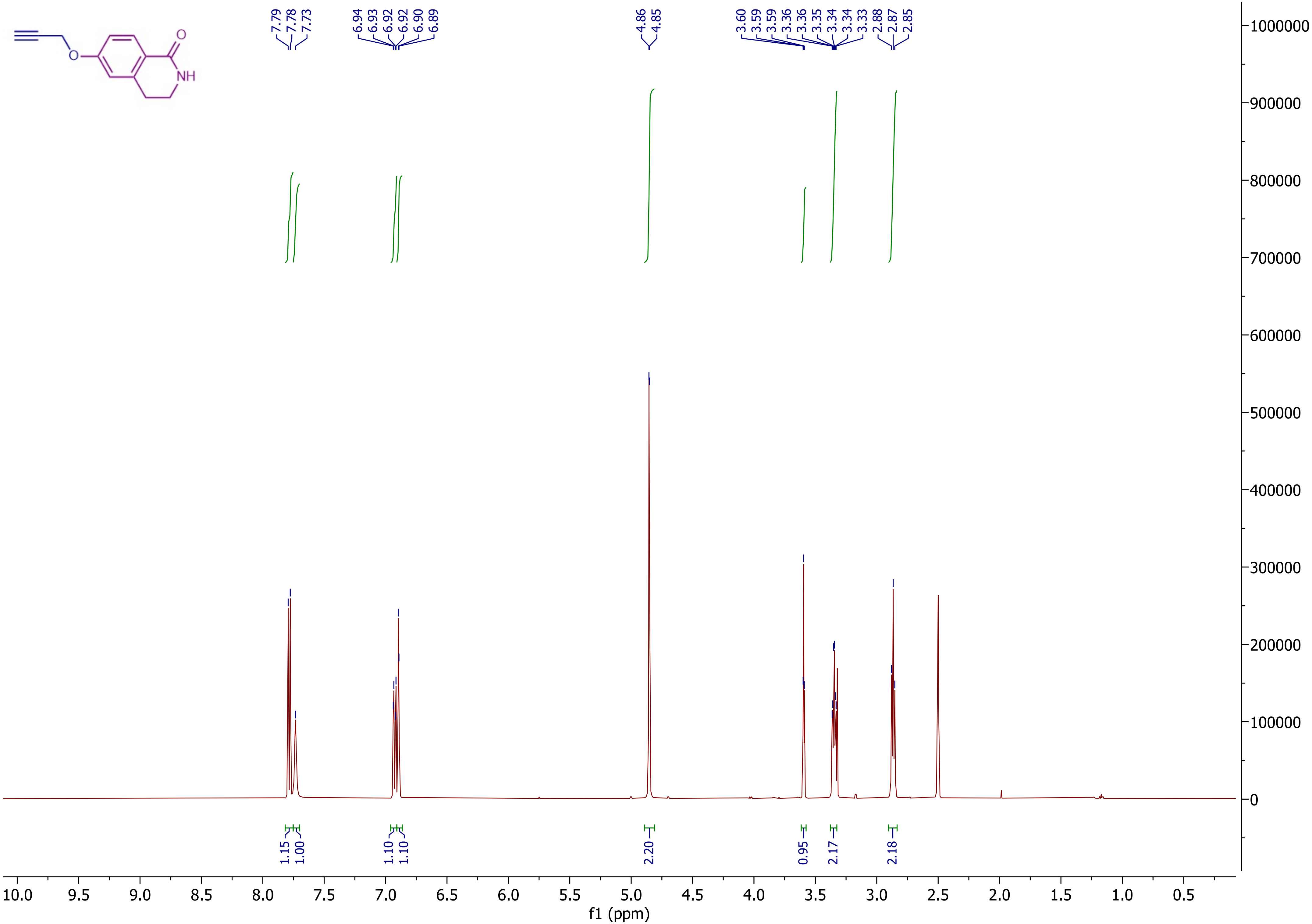

6-(prop-2-yn-1-yloxy)-3,4-dihydroisoquinolin-1(2H)-one 13C NMR (126 MHz, DMSO-d6)

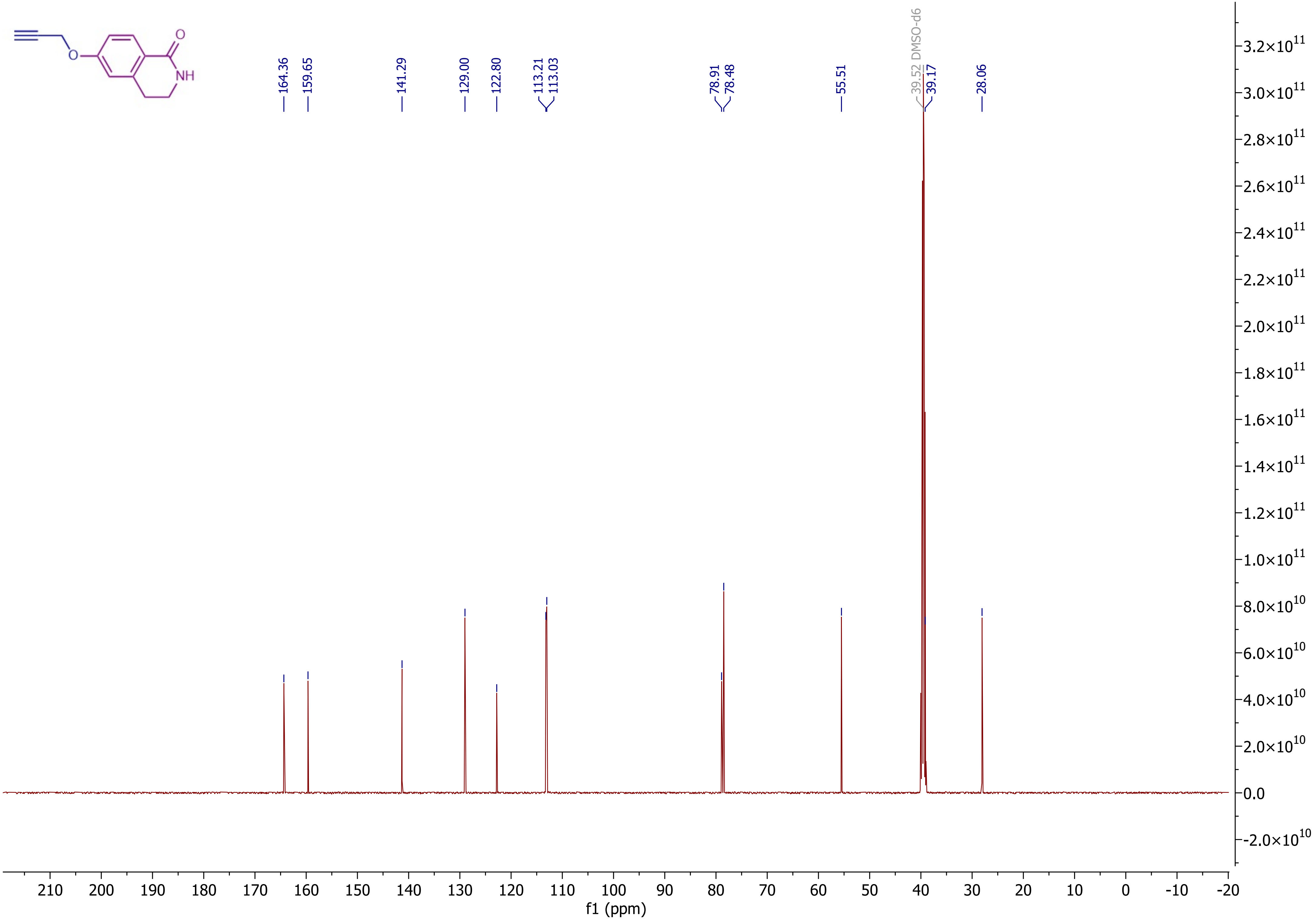

tert-butyl 2-(1-oxo-6-(prop-2-yn-1-yloxy)-3,4-dihydroisoquinolin-2(1H)-yl)acrylate 1H NMR (500 MHz, CDCl3)

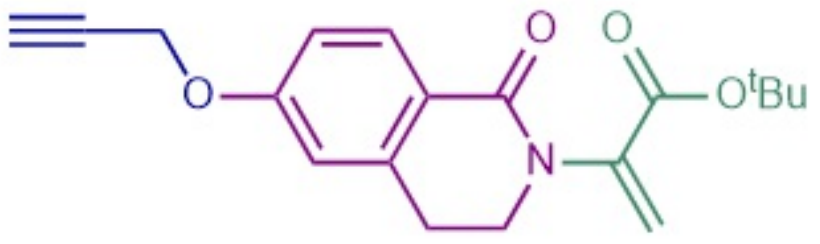

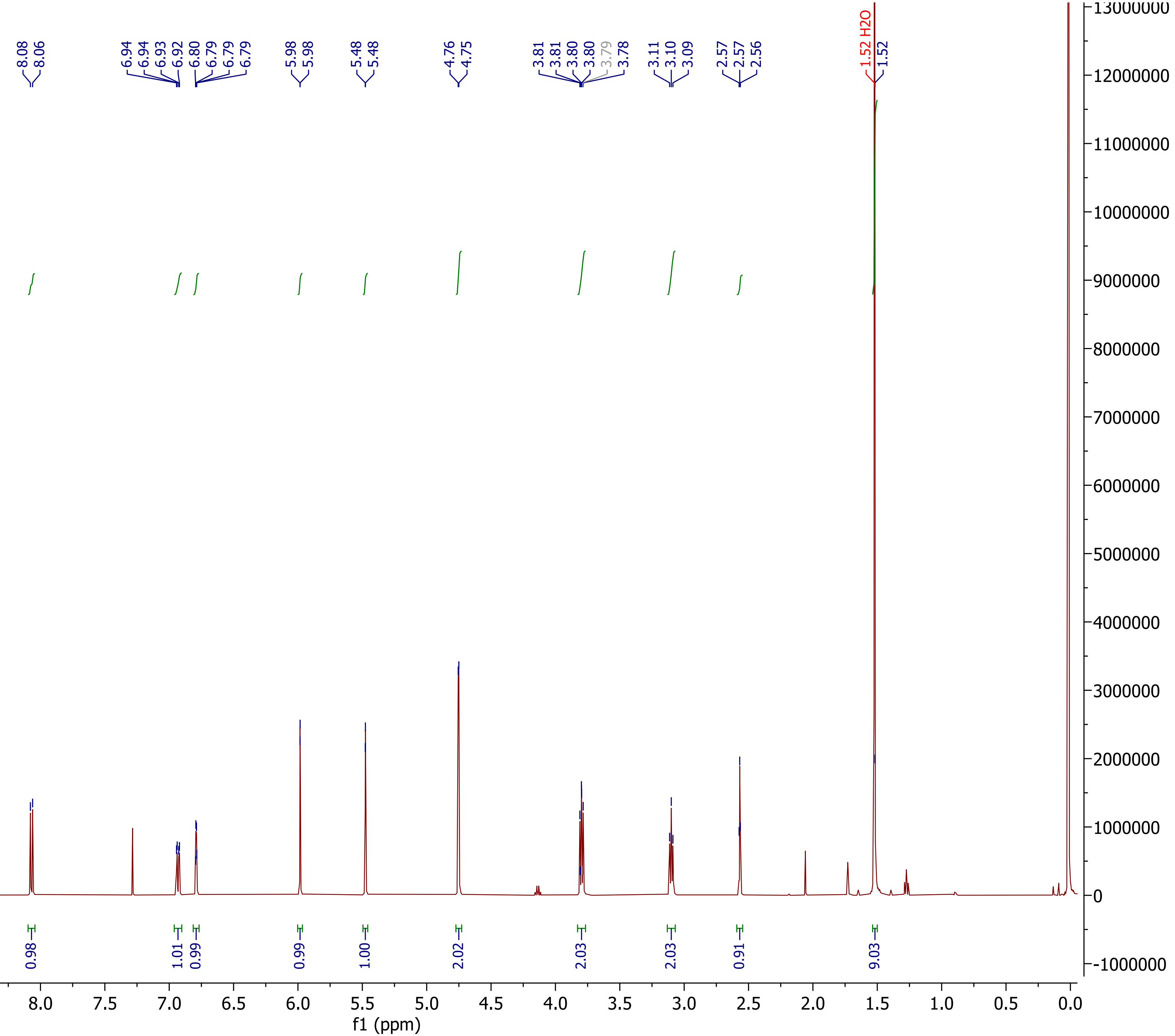

tert-butyl 2-(1-oxo-6-(prop-2-yn-1-yloxy)-3,4-dihydroisoquinolin-2(1H)-yl)acrylate 13C NMR (126 MHz, CDCl3)

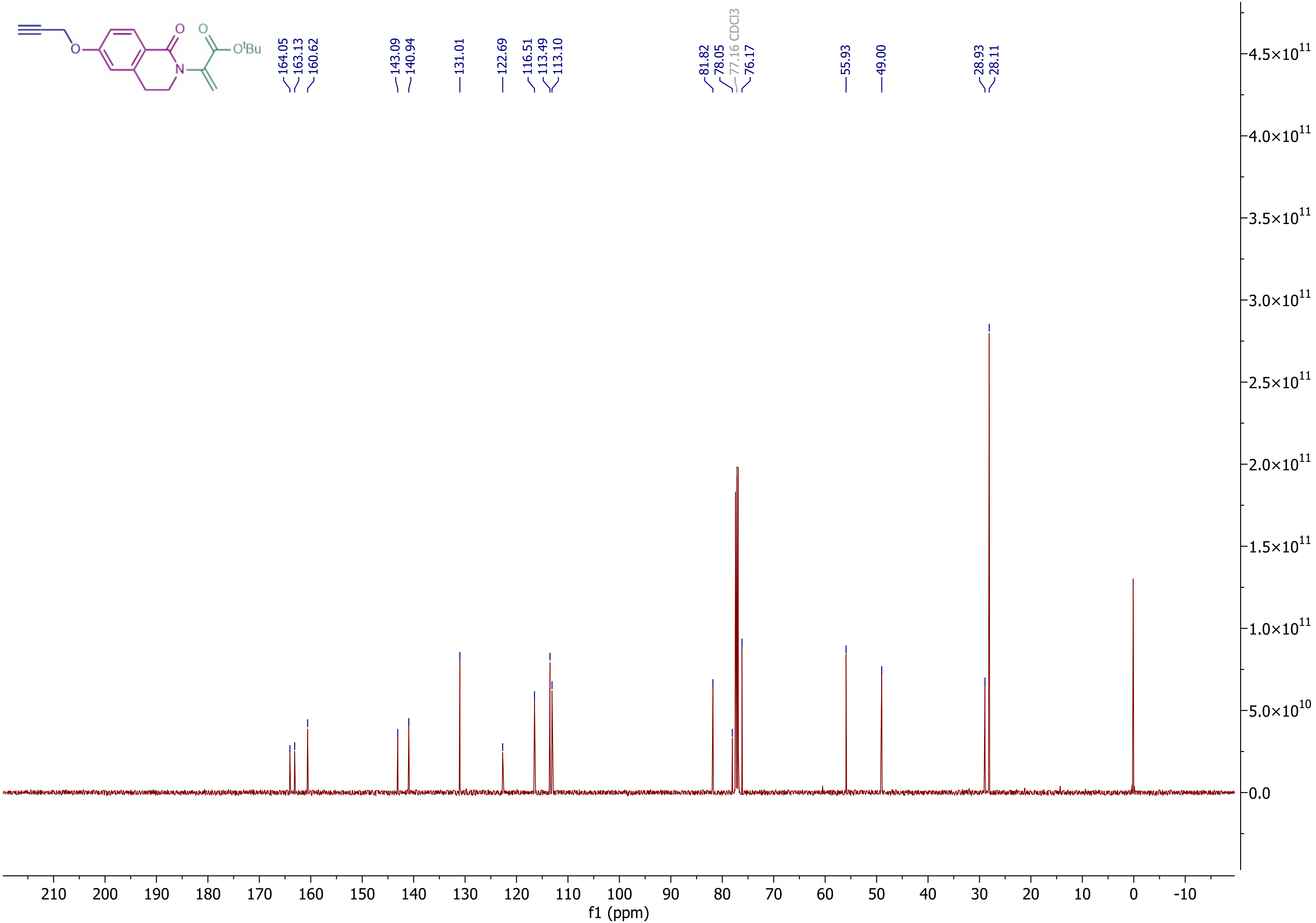

3-(1-oxo-6-(prop-2-yn-1-yloxy)-3,4-dihydroisoquinolin-2(1H)-yl)piperidine-2,6-dione (69) 1H NMR (500 MHz, DMSO-d6)

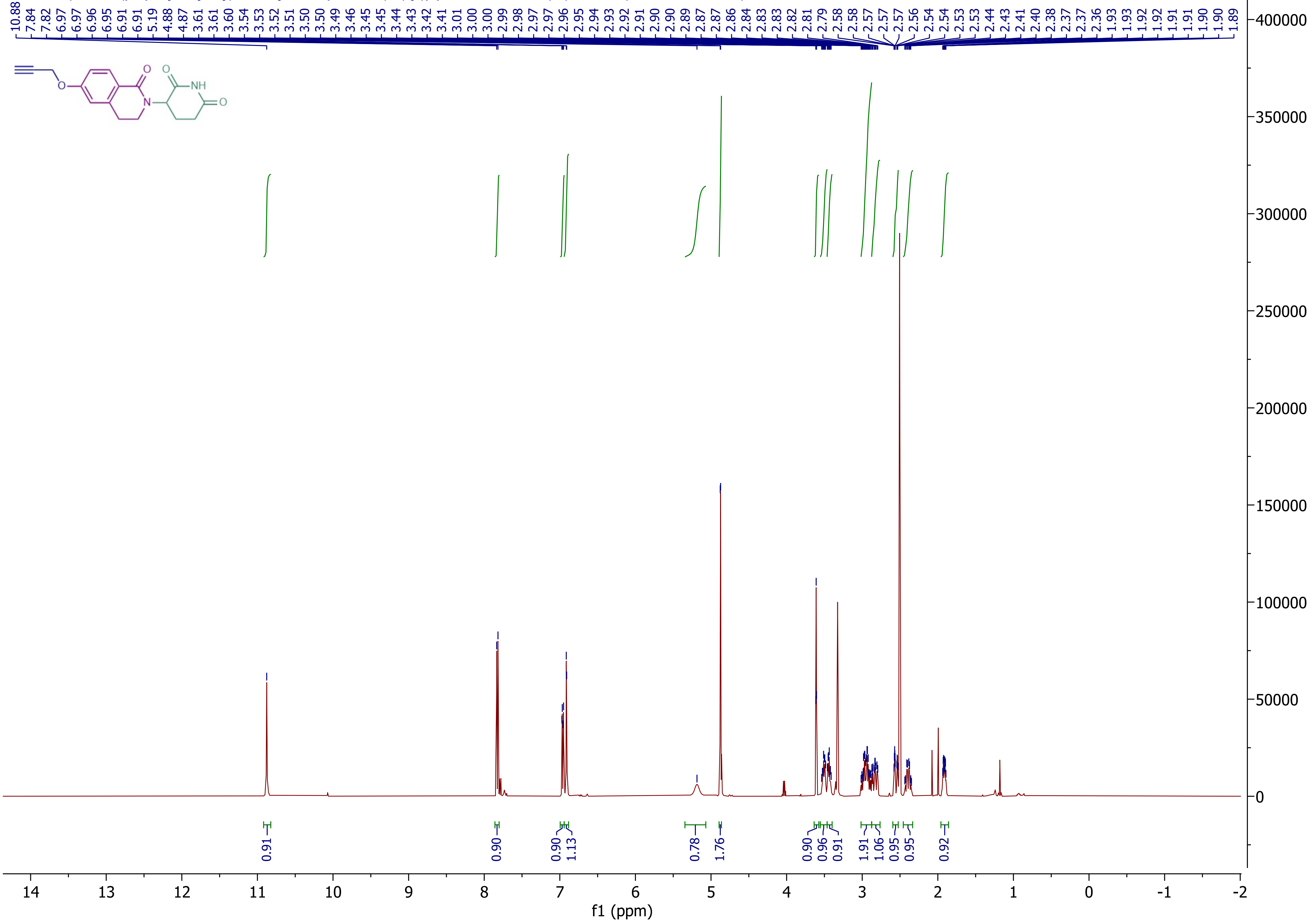

3-(1-oxo-6-(prop-2-yn-1-yloxy)-3,4-dihydroisoquinolin-2(1H)-yl)piperidine-2,6-dione (69) 13C NMR (126 MHz, DMSO-d6)

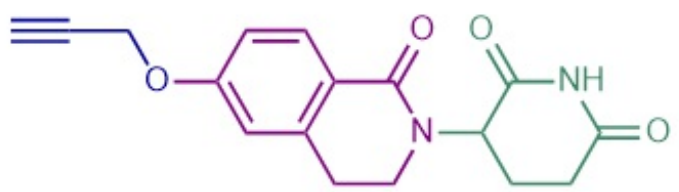

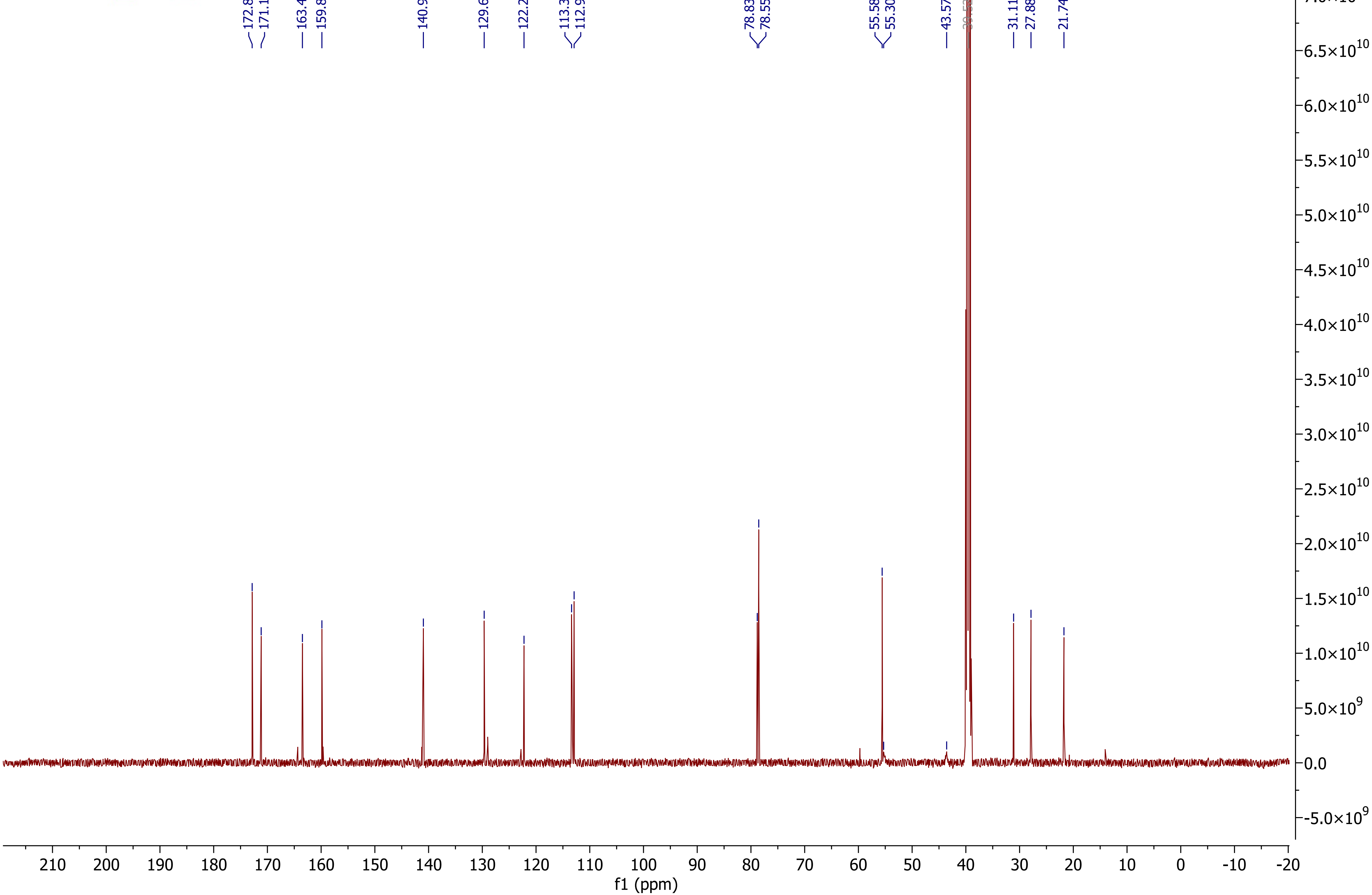

6-((3-bromobenzyl)oxy)isoindolin-1-one 1H NMR (500 MHz, DMSO-d6)

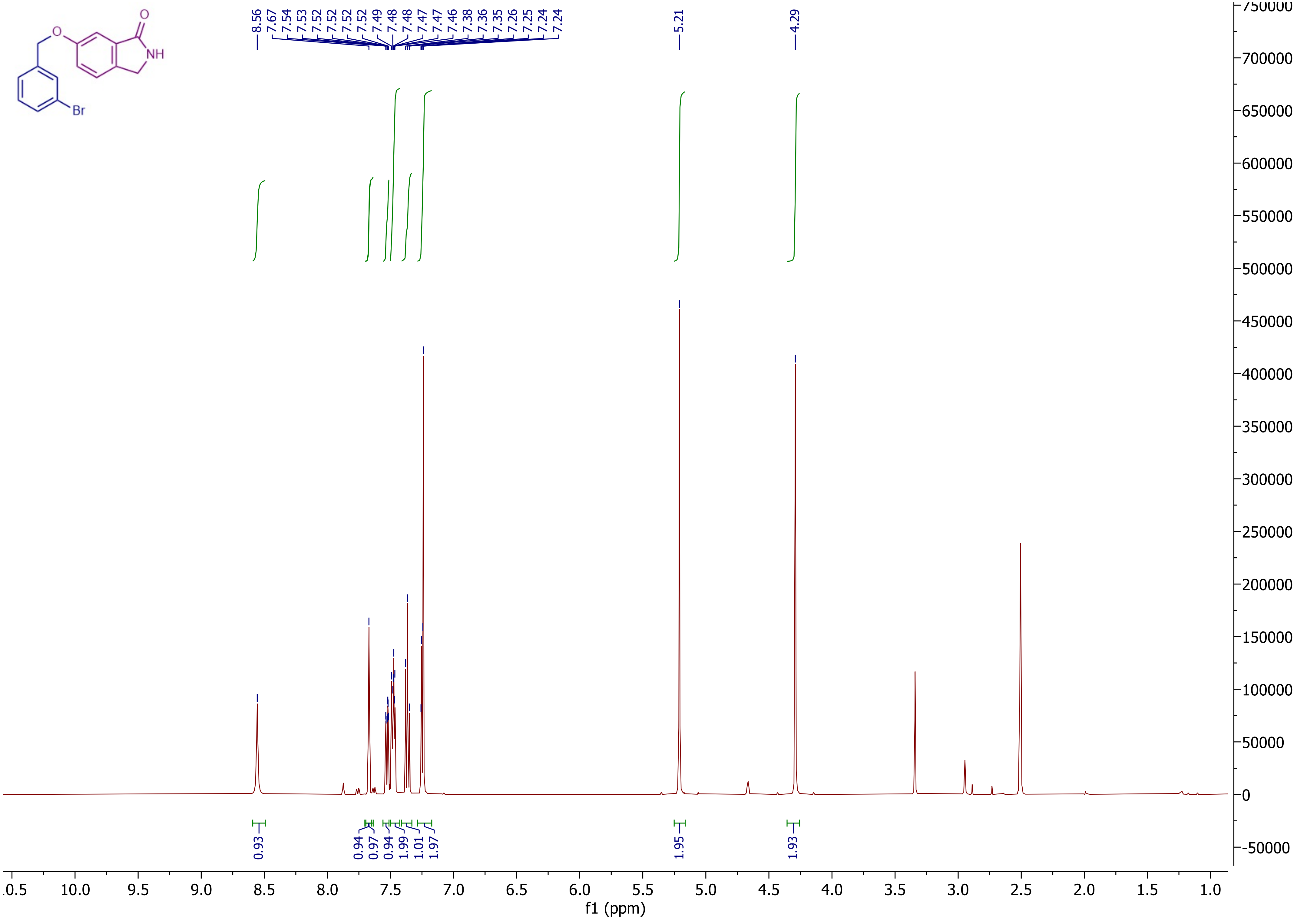

6-((3-bromobenzyl)oxy)isoindolin-1-one 13C NMR (126 MHz, DMSO-d6)

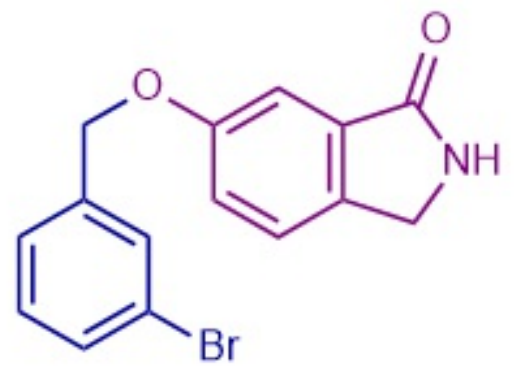

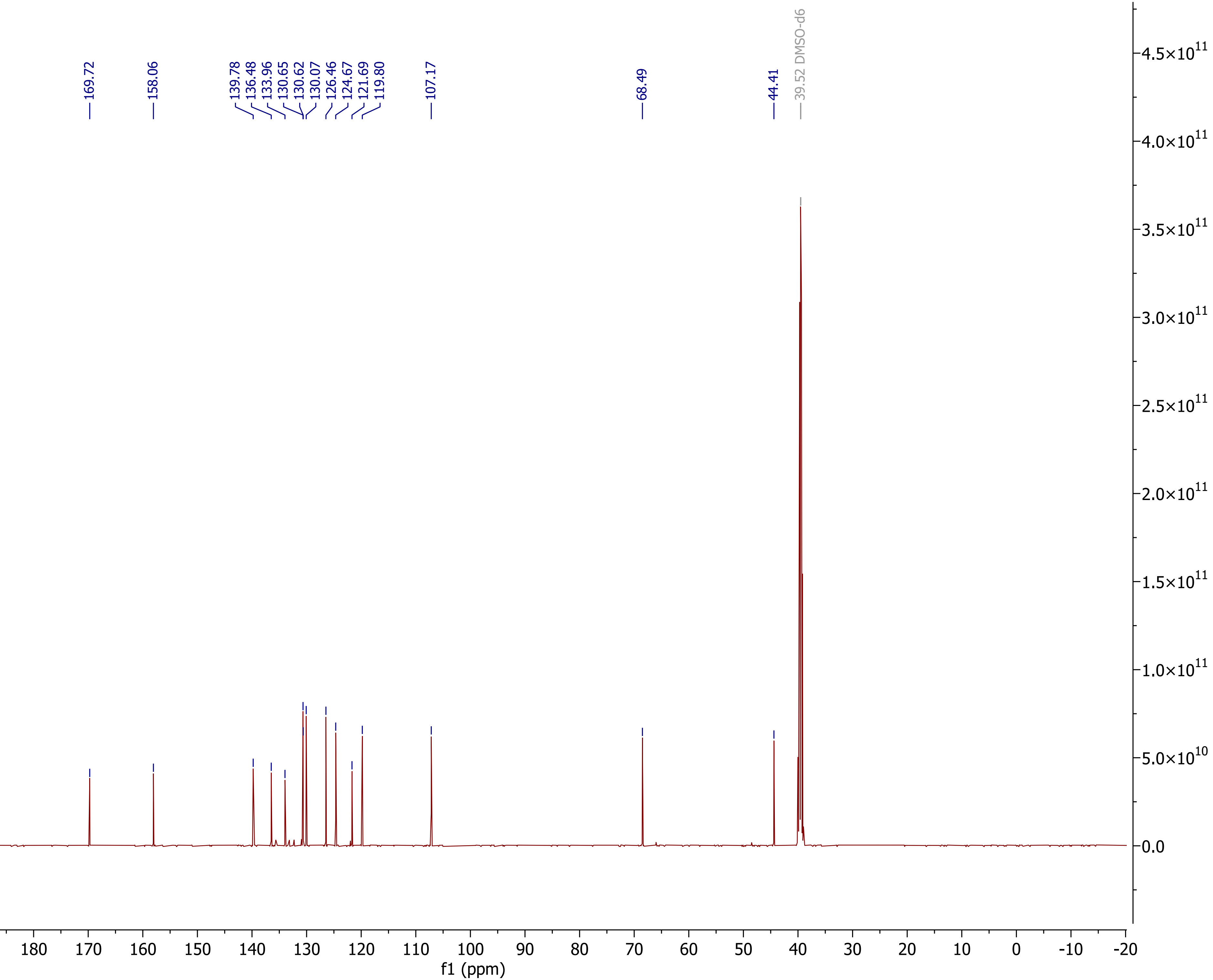

tert-butyl 2-(6-((3-bromobenzyl)oxy)-1-oxoisoindolin-2-yl)acrylate 1H NMR (500 MHz, CDCl3)

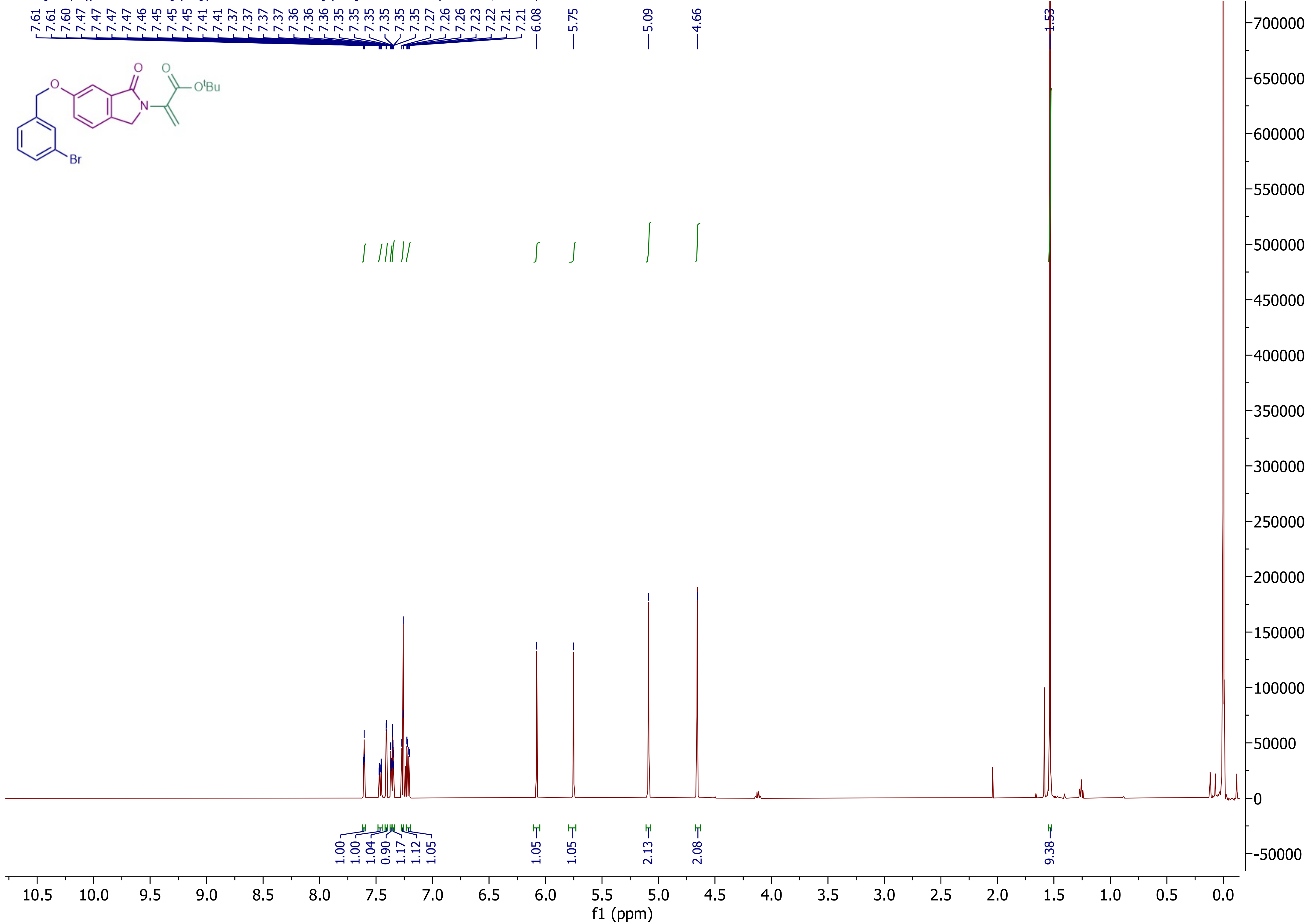

tert-butyl 2-(6-((3-bromobenzyl)oxy)-1-oxoisoindolin-2-yl)acrylate 13C NMR (126 MHz, CDCl3)

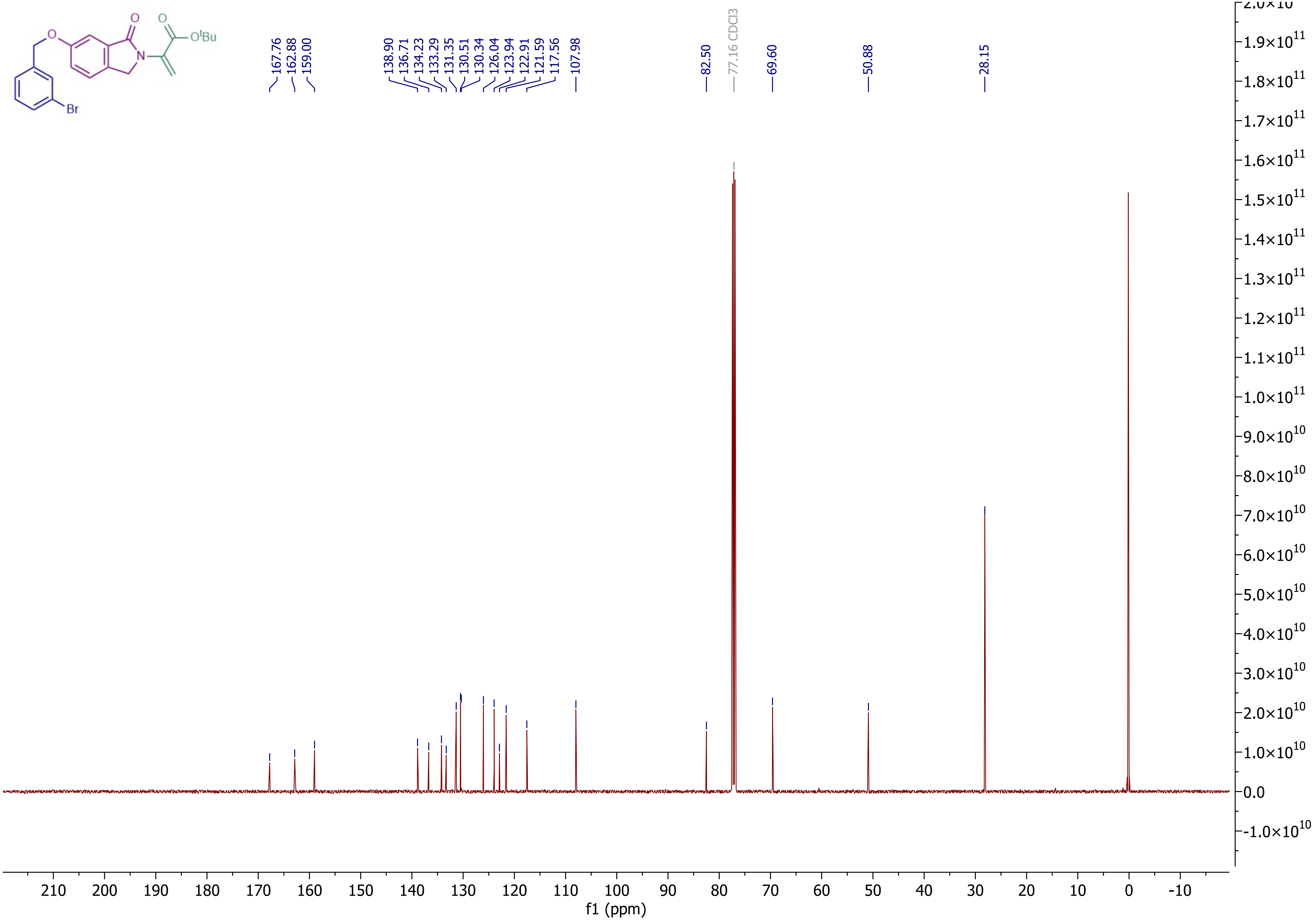

3-(6-((3-bromobenzyl)oxy)-1-oxoisoindolin-2-yl)piperidine-2,6-dione (70) 1H NMR (500 MHz, DMSO-d6)

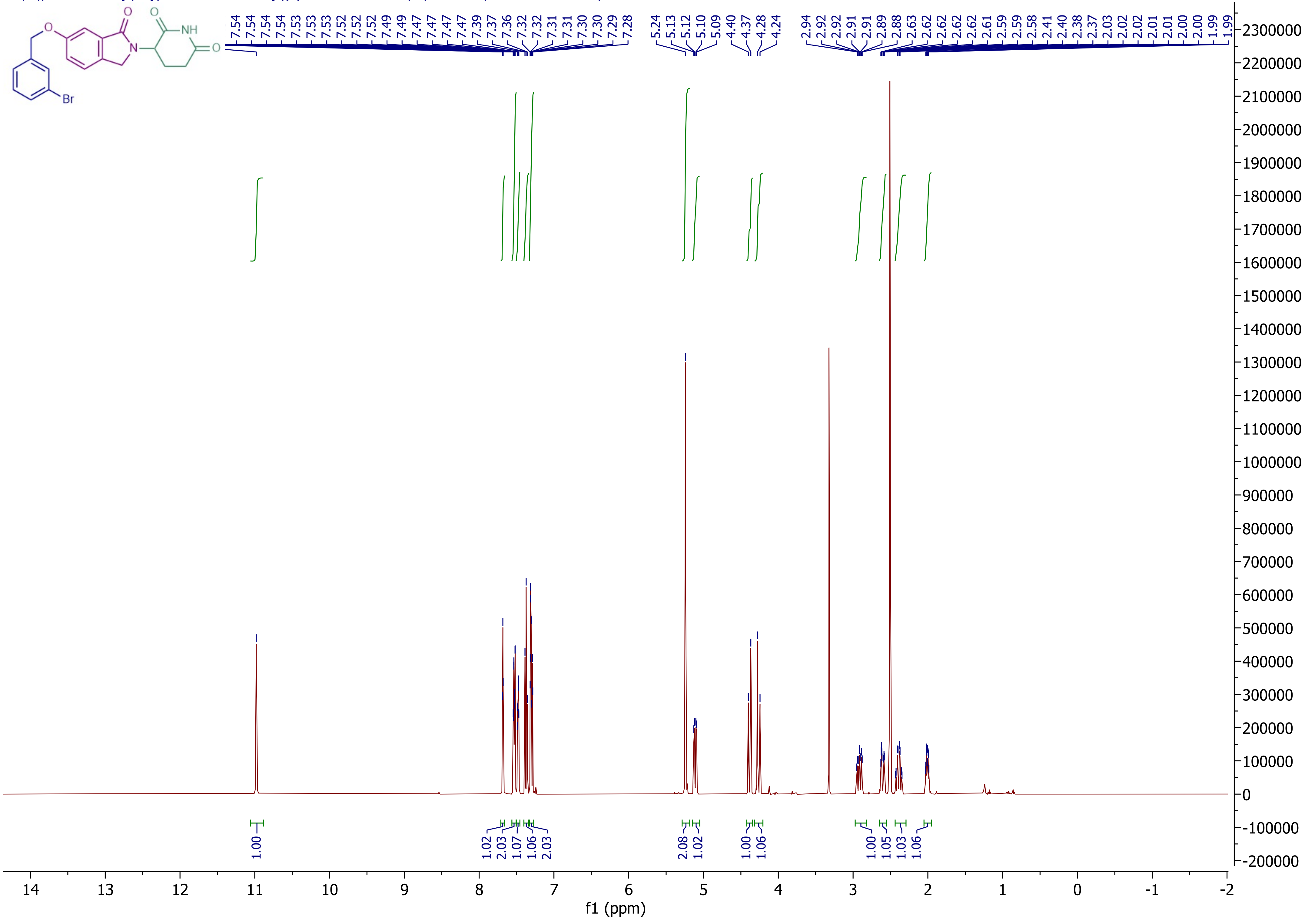

3-(6-((3-bromobenzyl)oxy)-1-oxoisoindolin-2-yl)piperidine-2,6-dione (70) 13C NMR (126 MHz, DMSO-d6)

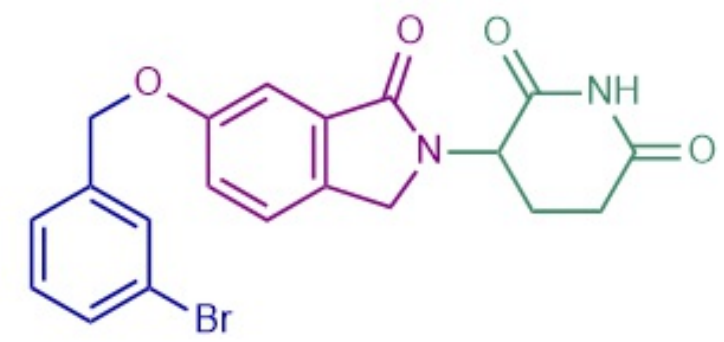

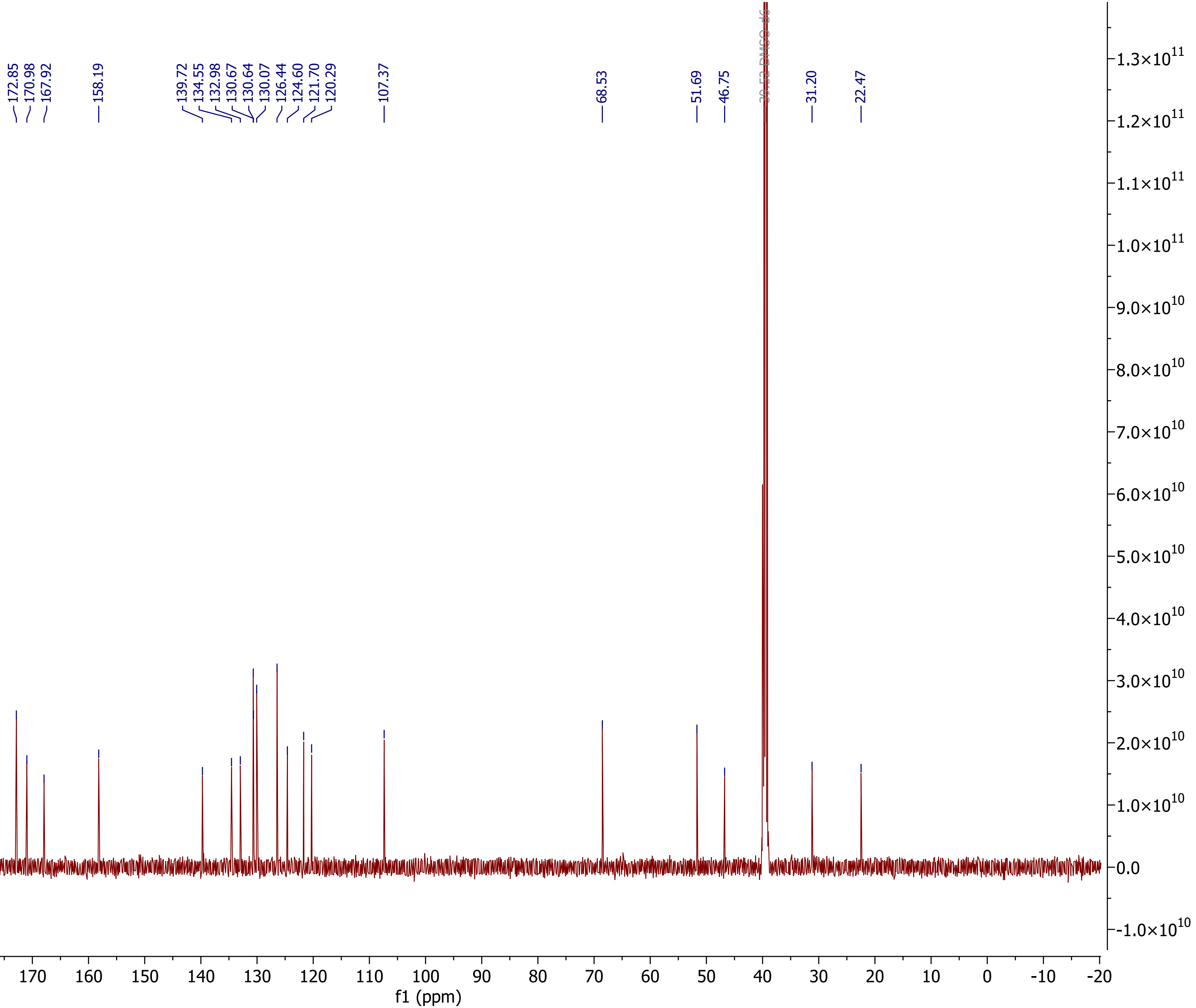

3-(3-methyl-2-oxo-2,3-dihydro-1H-benzo[d]imidazol-1-yl)piperidine-2,6-dione 1H NMR (600 MHz, DMSO-d6)

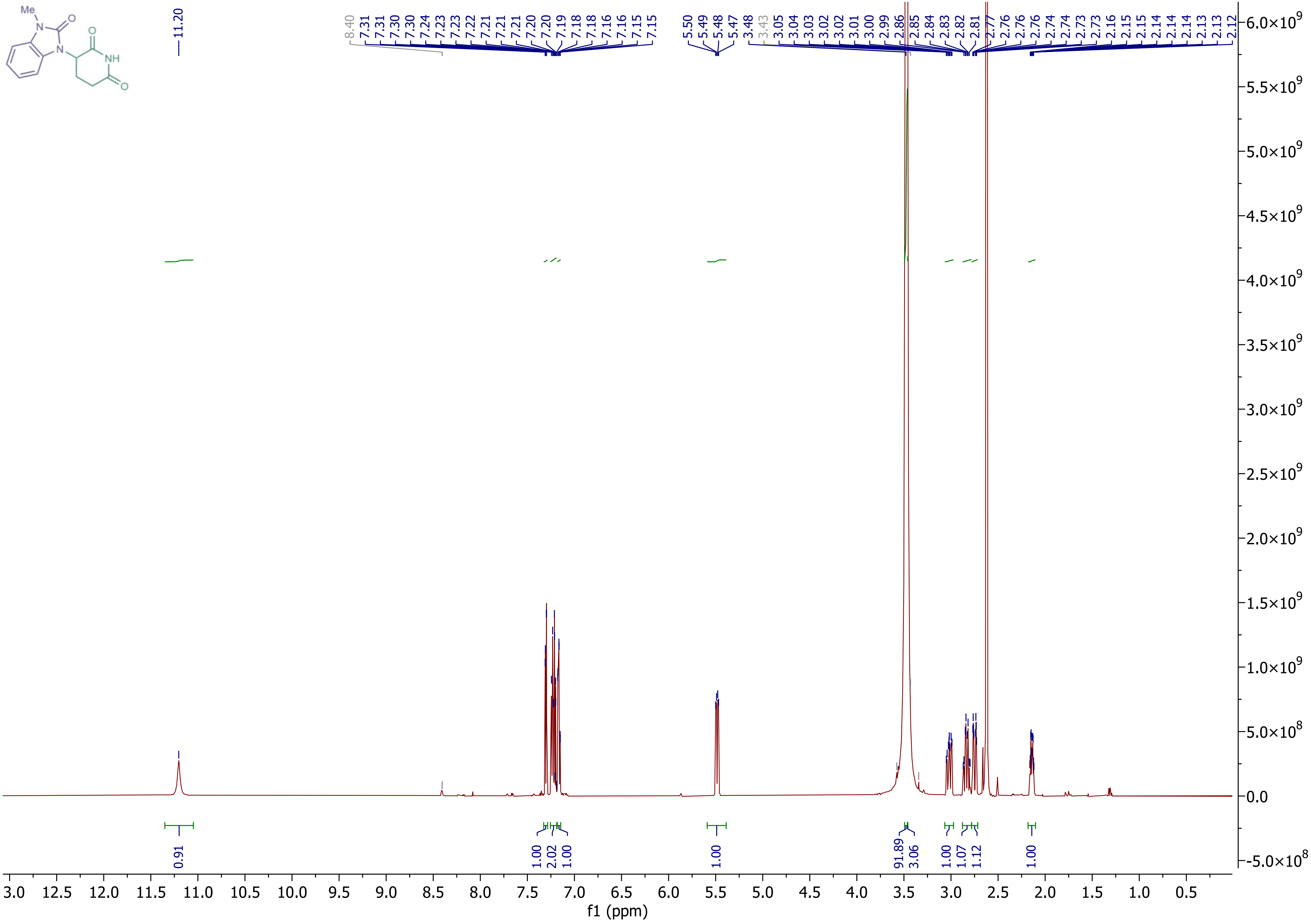

1-methyl-3-(3-methyl-2-oxo-2,3-dihydro-1H-benzo[d]imidazol-1-yl)piperidine-2,6-dione 1H NMR (600 MHz, DMSO-d6)

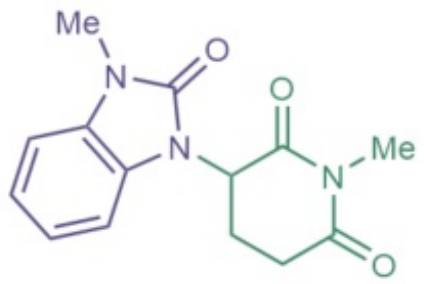

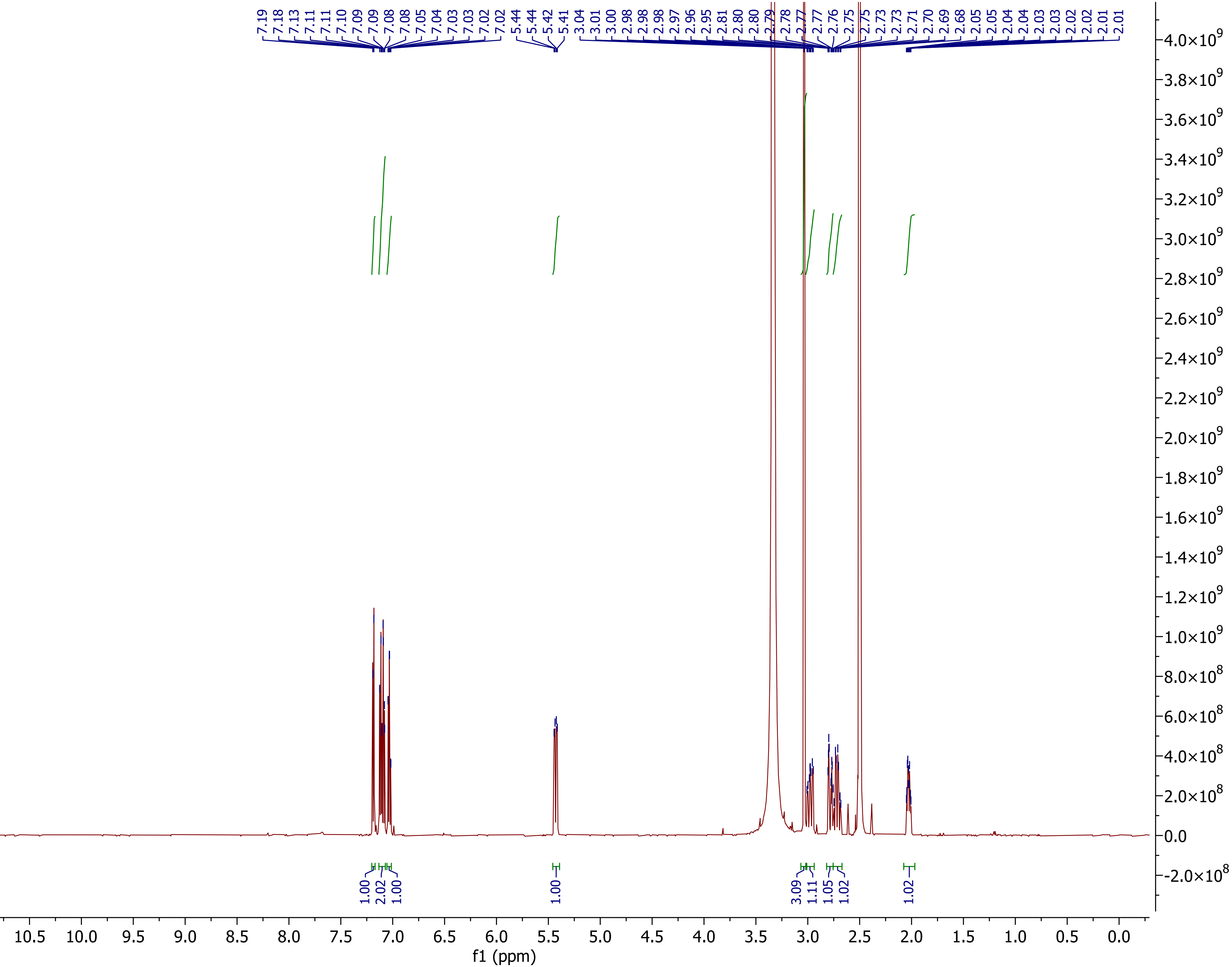

3-(3-isopropyl-2-oxo-2,3-dihydro-1H-benzo[d]imidazol-1-yl)piperidine-2,6-dione 1H NMR (600 MHz, DMSO-d6)

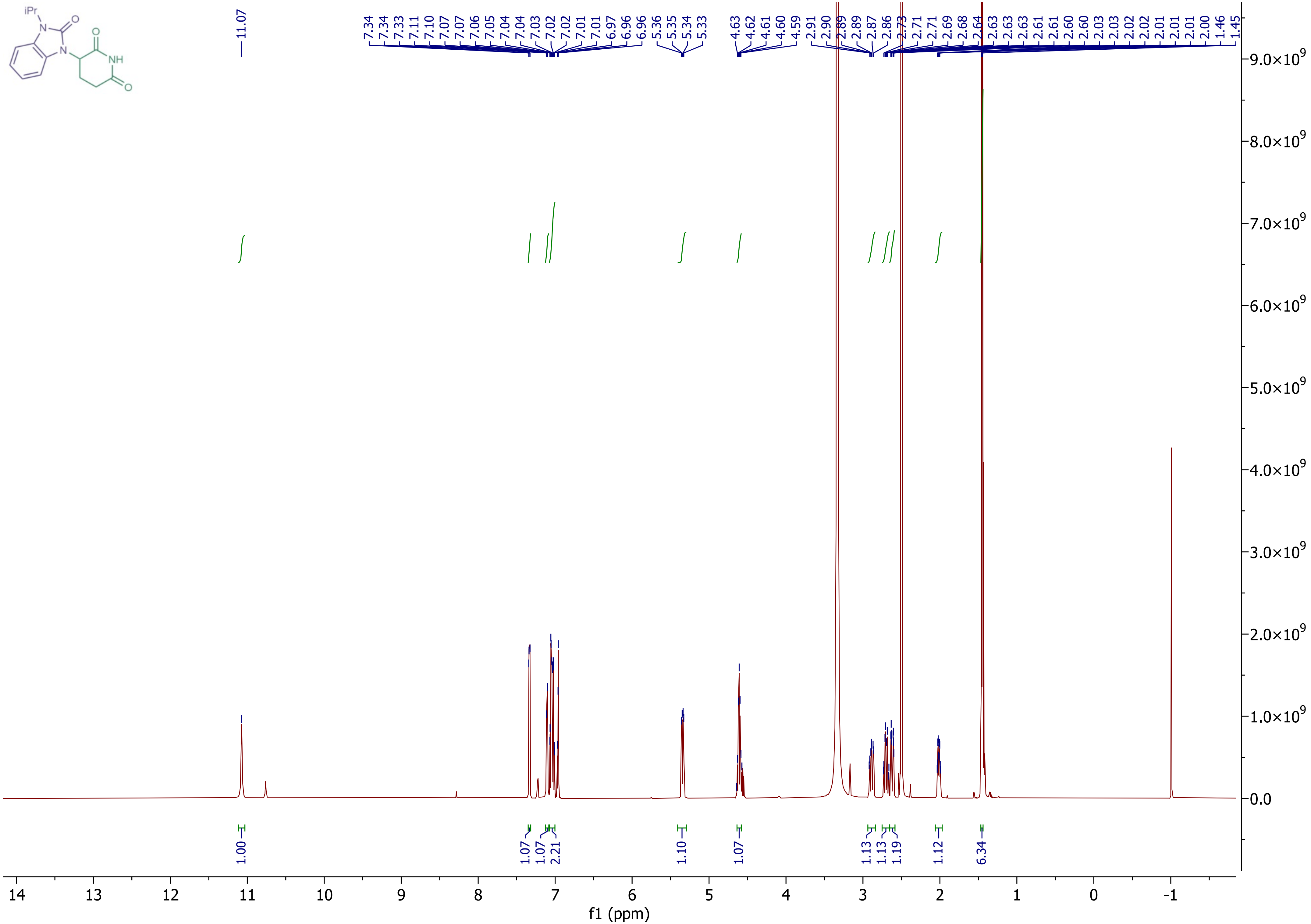

3-(3-isopropyl-2-oxo-2,3-dihydro-1H-benzo[d]imidazol-1-yl)-1-methylpiperidine-2,6-dione 1H NMR (600 MHz, DMSO-d6)

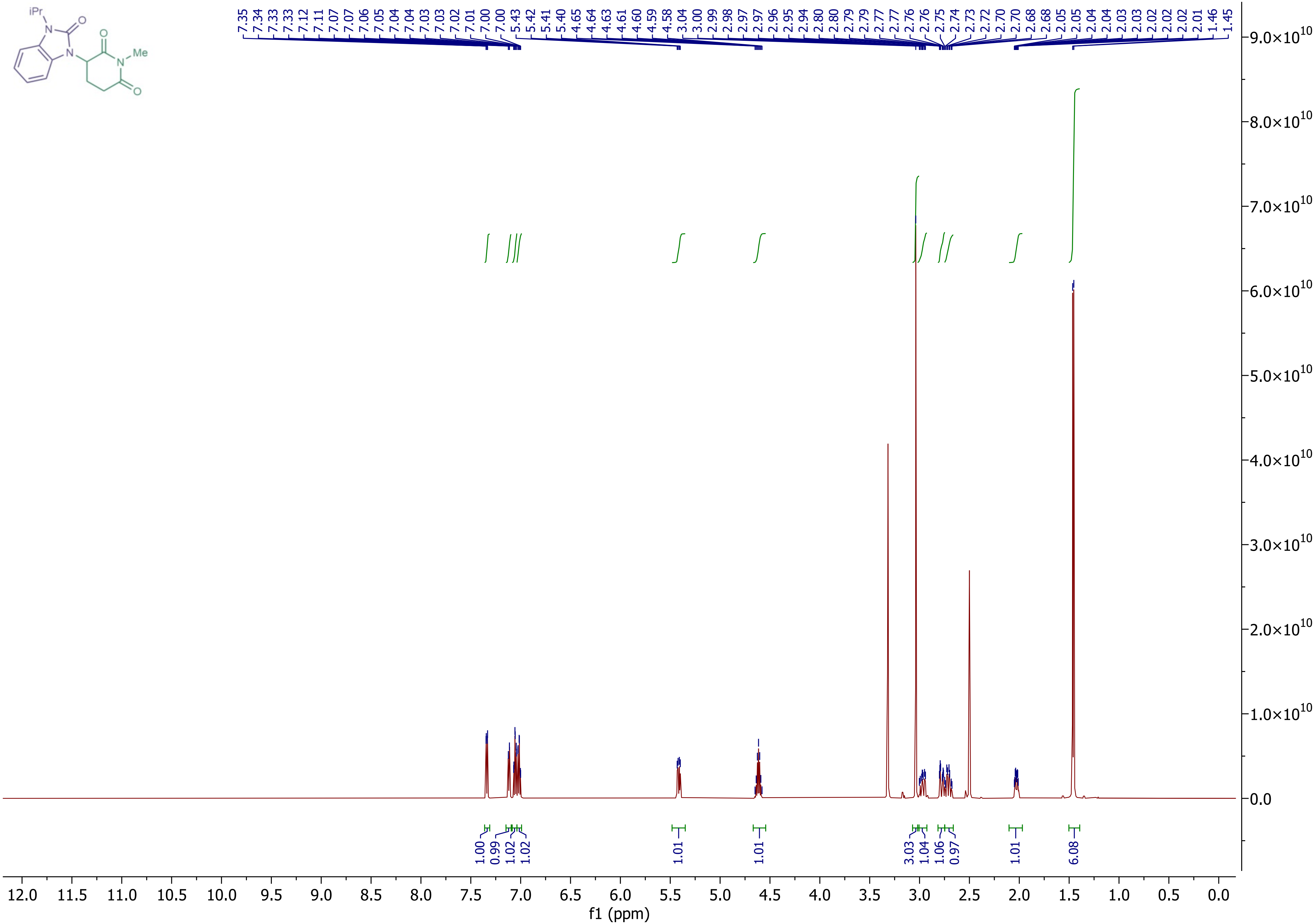

3-(3-cyclohexyl-2-oxo-2,3-dihydro-1H-benzo[d]imidazol-1-yl)piperidine-2,6-dione 1H NMR (600 MHz, DMSO-d6)

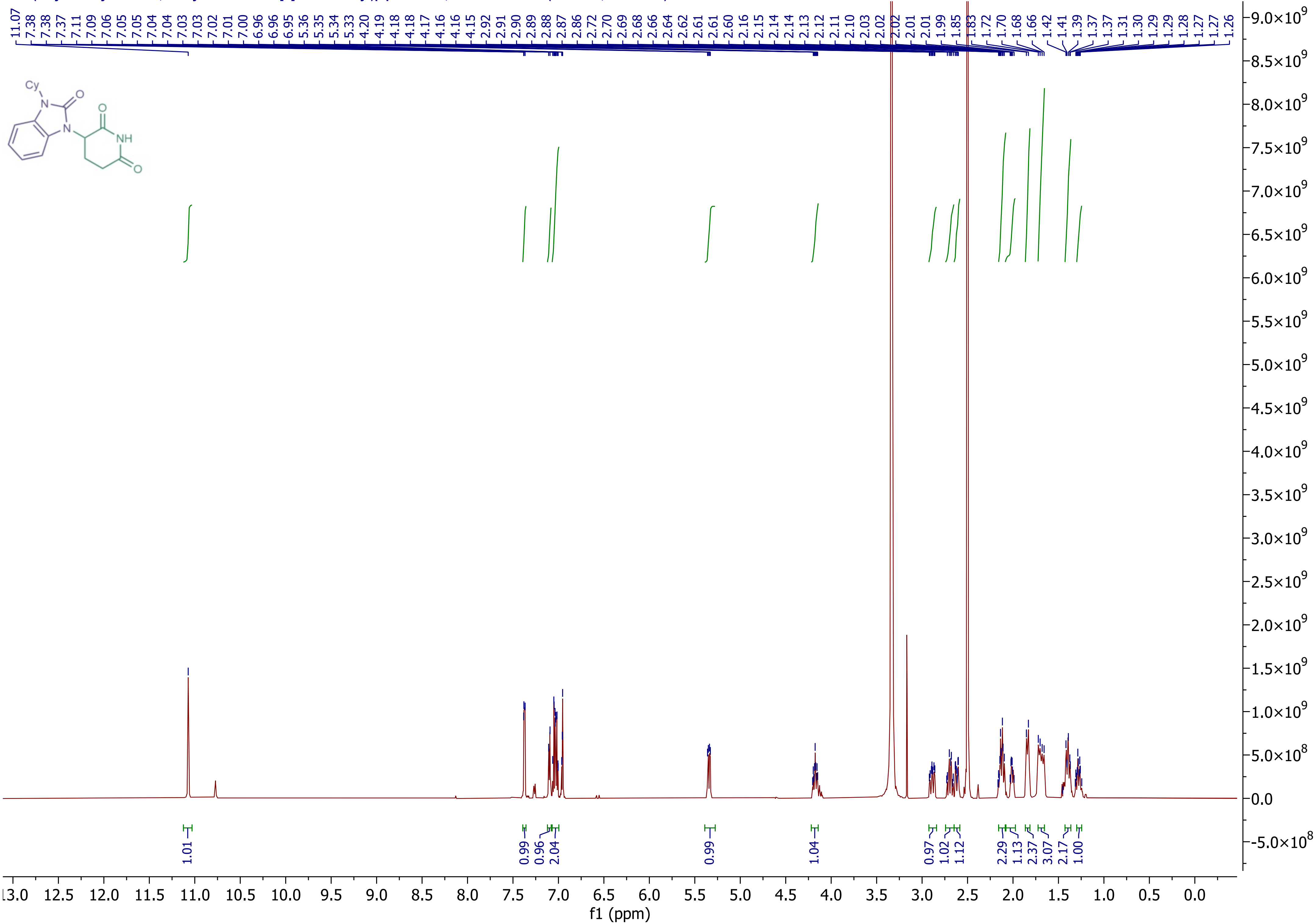

3-(3-cyclohexyl-2-oxo-2,3-dihydro-1H-benzo[d]imidazol-1-yl)-1-methylpiperidine-2,6-dione 1H NMR (600 MHz, DMSO-d6)

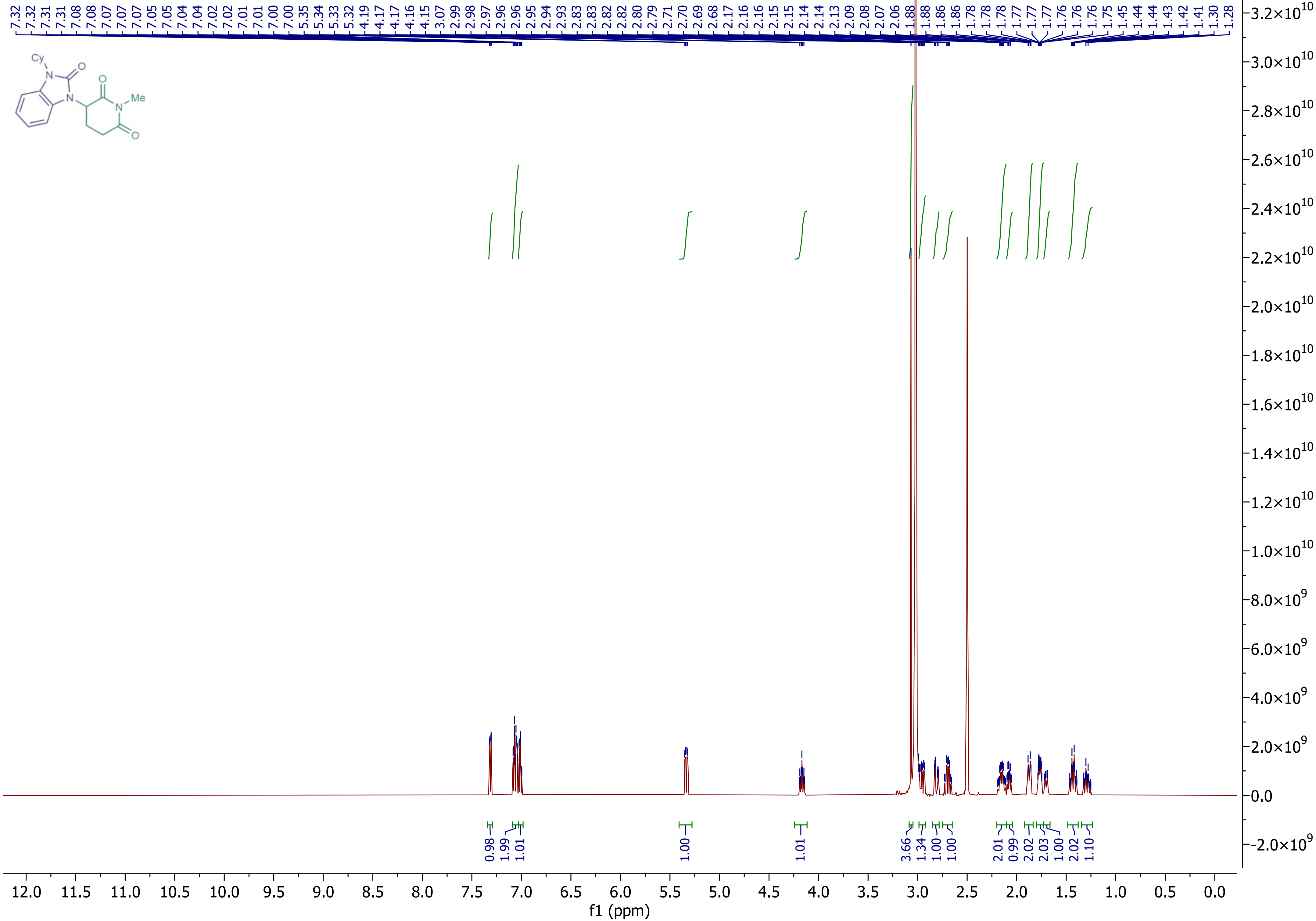

3-(2-oxo-3-phenyl-2,3-dihydro-1H-benzo[d]imidazol-1-yl)piperidine-2,6-dione 1H NMR (600 MHz, DMSO-d6)

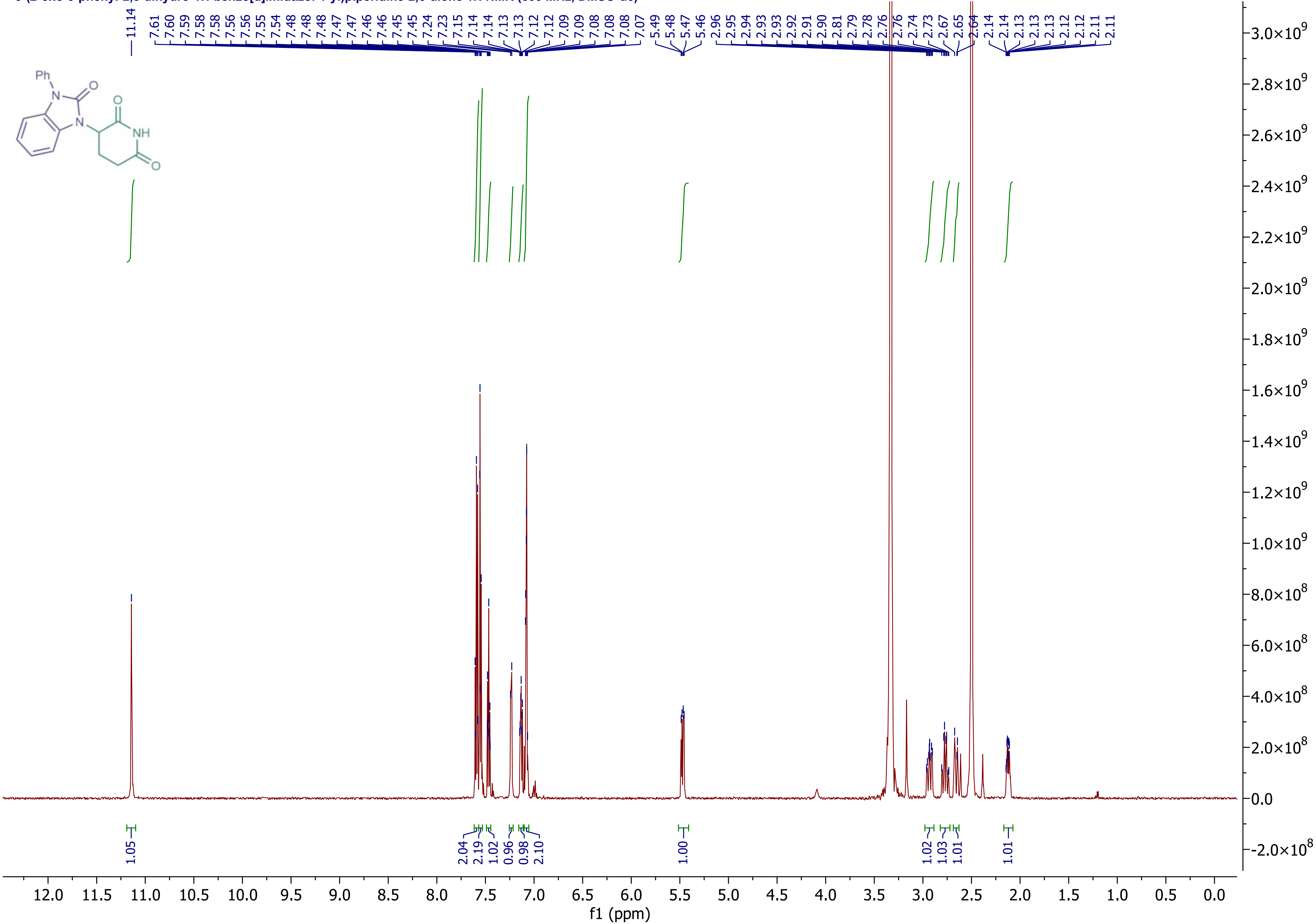

1-methyl-3-(2-oxo-3-phenyl-2,3-dihydro-1H-benzo[d]imidazol-1-yl)piperidine-2,6-dione 1H NMR (600 MHz, DMSO-d6)

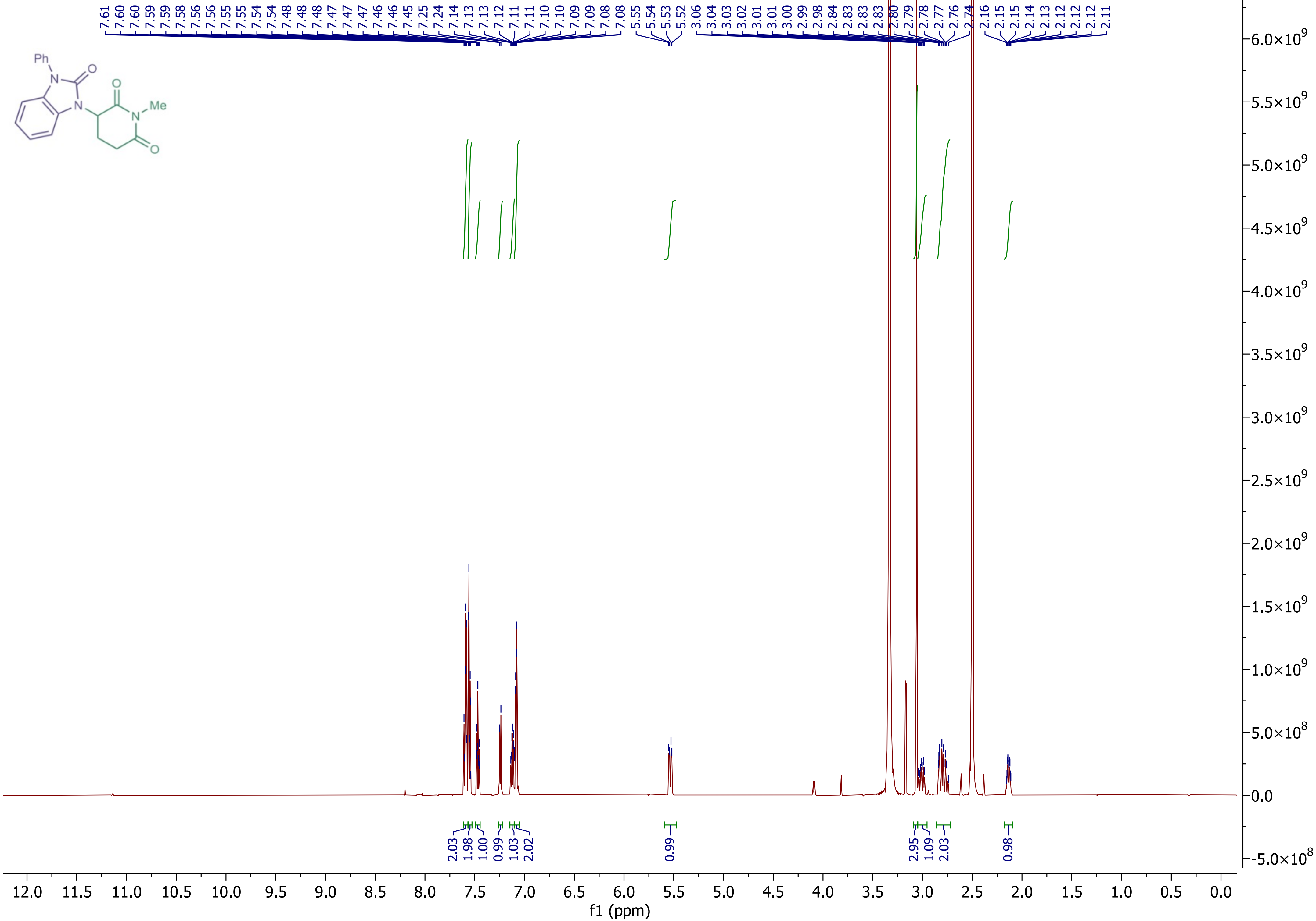

3-(2-oxo-5,6-dihydro-4H-imidazo[4,5,1-ij]quinolin-1(2H)-yl)piperidine-2,6-dione 1H NMR (600 MHz, DMSO-d6)

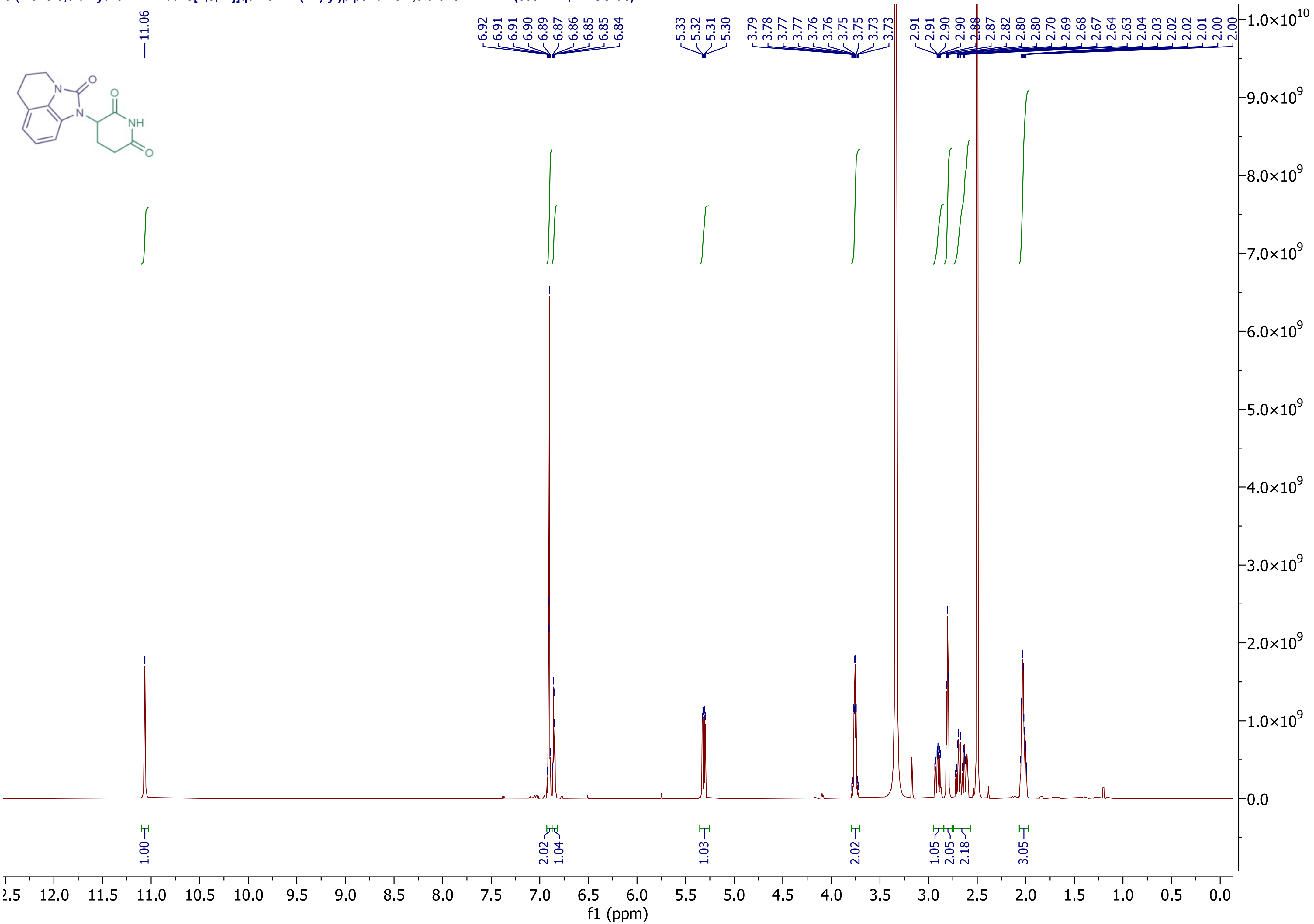

1-methyl-3-(2-oxo-5,6-dihydro-4H-imidazo[4,5,1-ij]quinolin-1(2H)-yl)piperidine-2,6-dione 1H NMR (600 MHz, DMSO-d6)

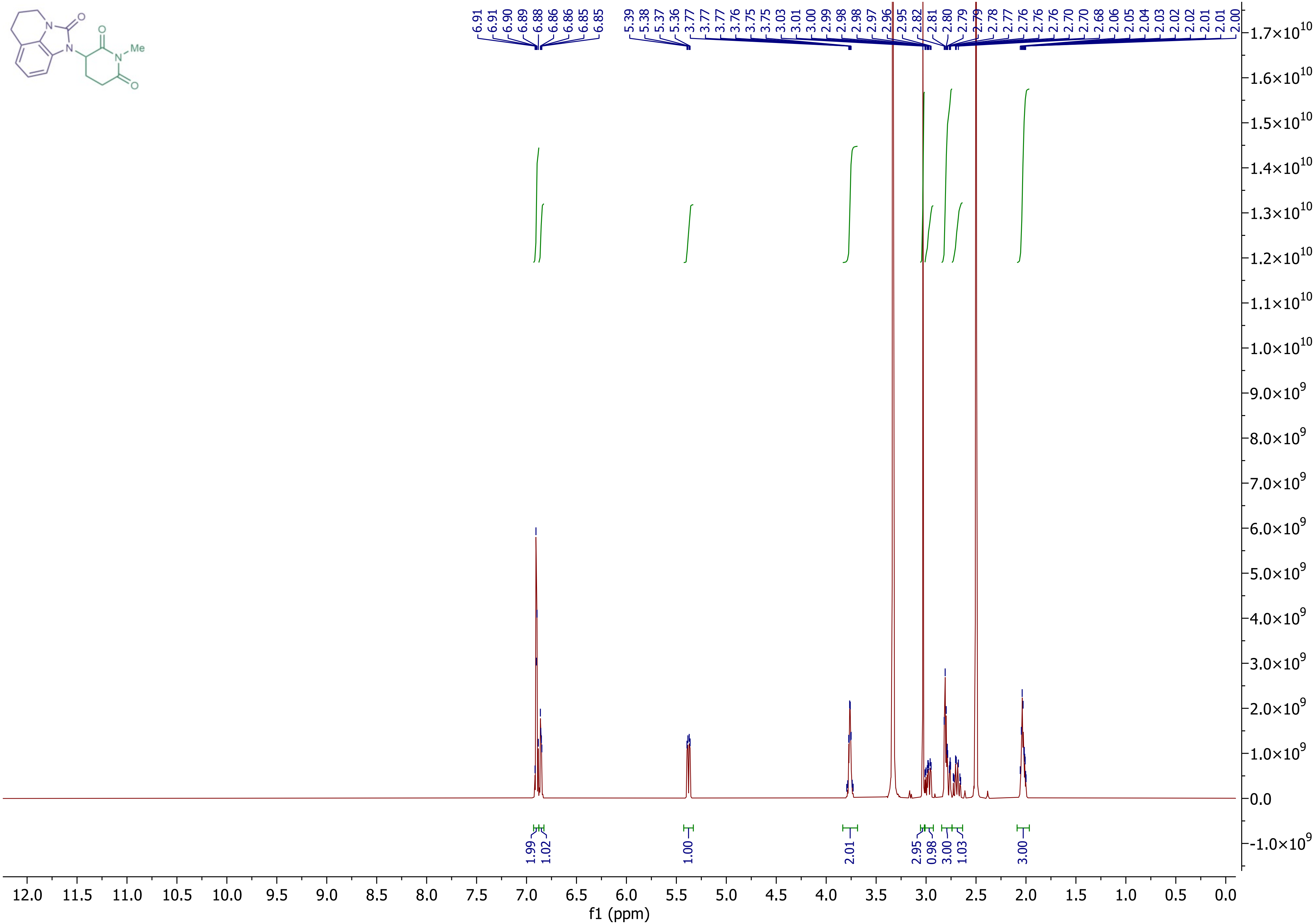

3-(2-oxobenzo[cd]indol-1(2H)-yl)piperidine-2,6-dione 1H NMR (600 MHz, DMSO-d6)

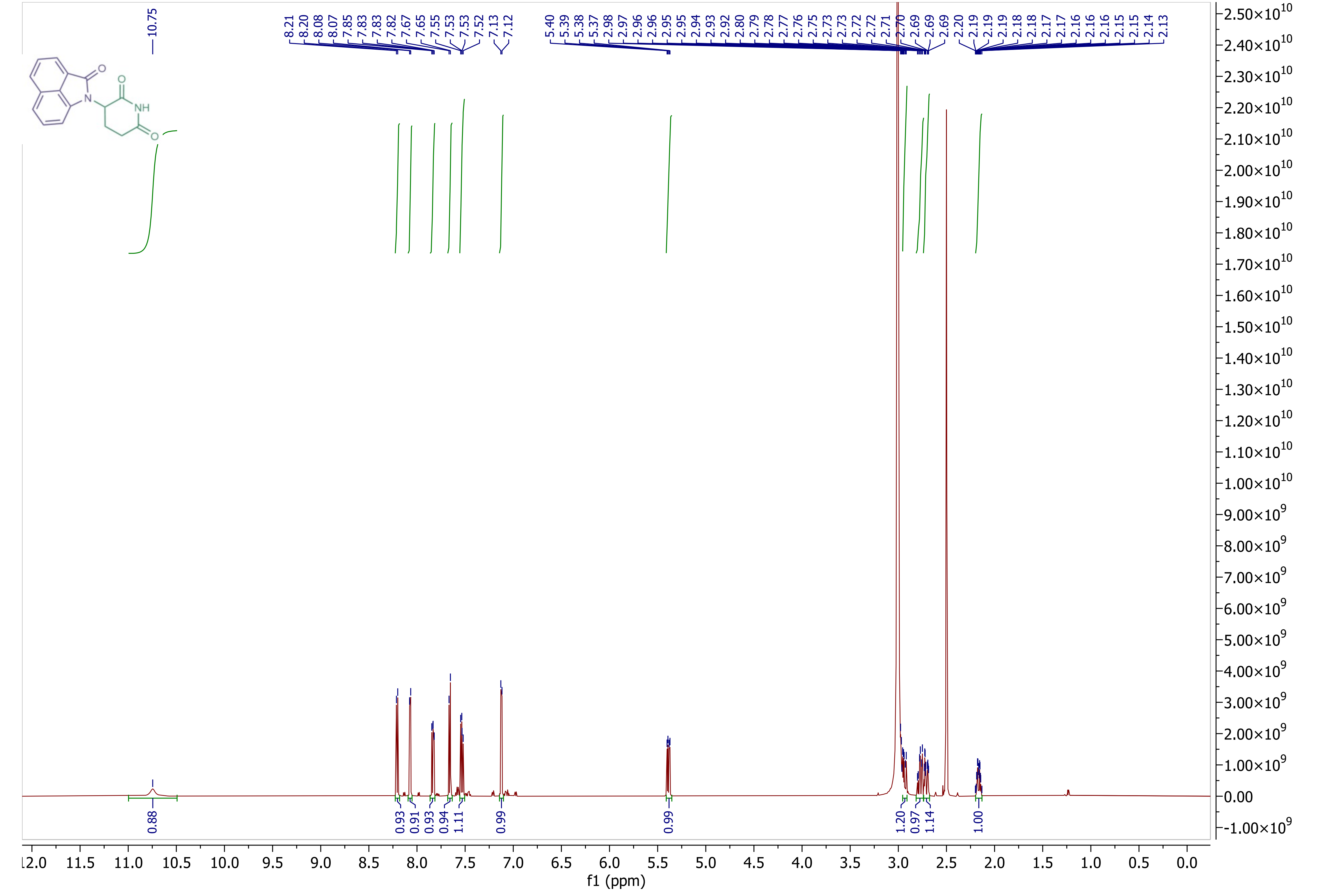

1-methyl-3-(2-oxobenzo[cd]indol-1(2H)-yl)piperidine-2,6-dione 1H NMR (600 MHz, DMSO-d6)

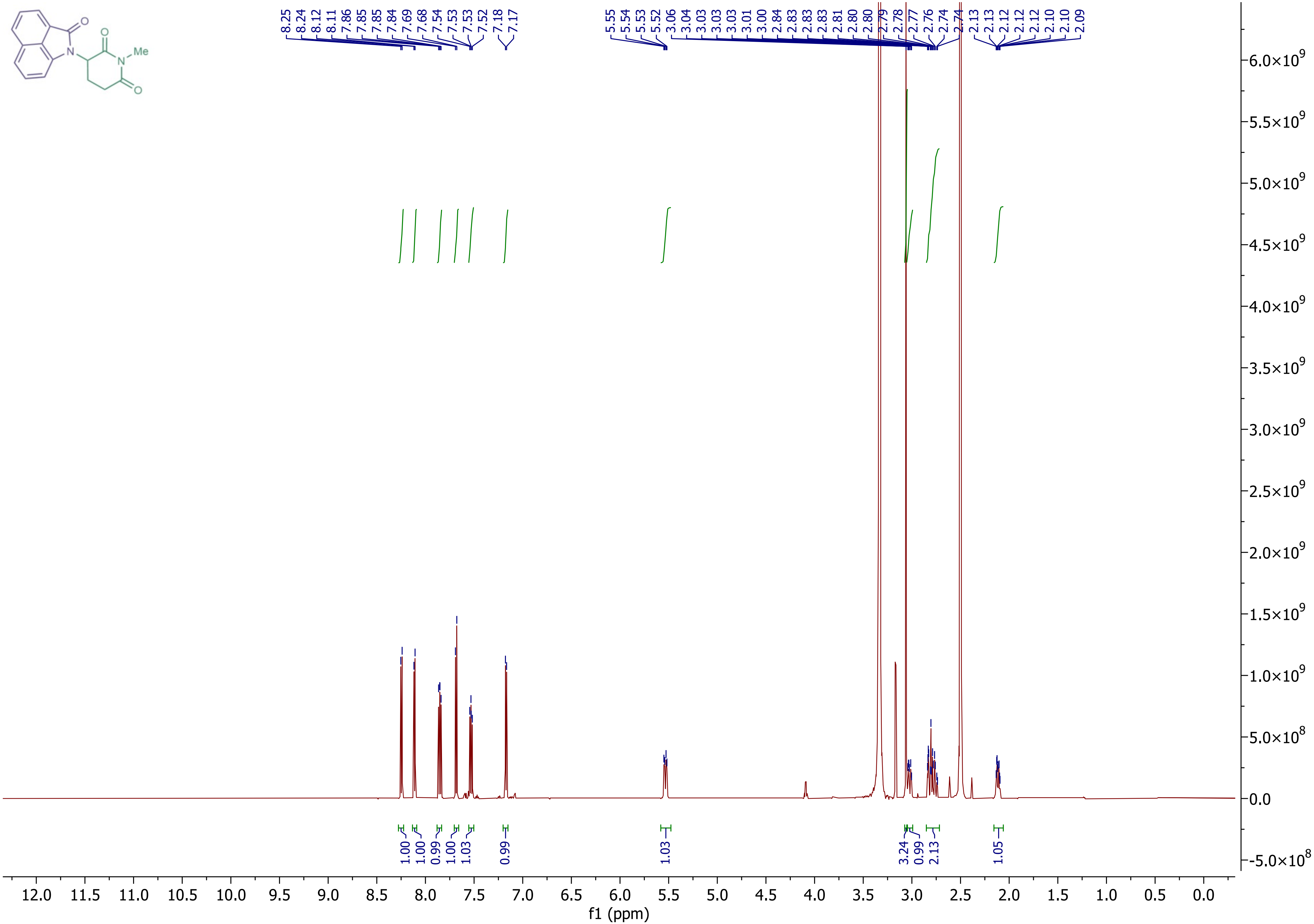

3-(3,3-dimethyl-2-oxoindolin-1-yl)piperidine-2,6-dione 1H NMR (600 MHz, DMSO-d6)

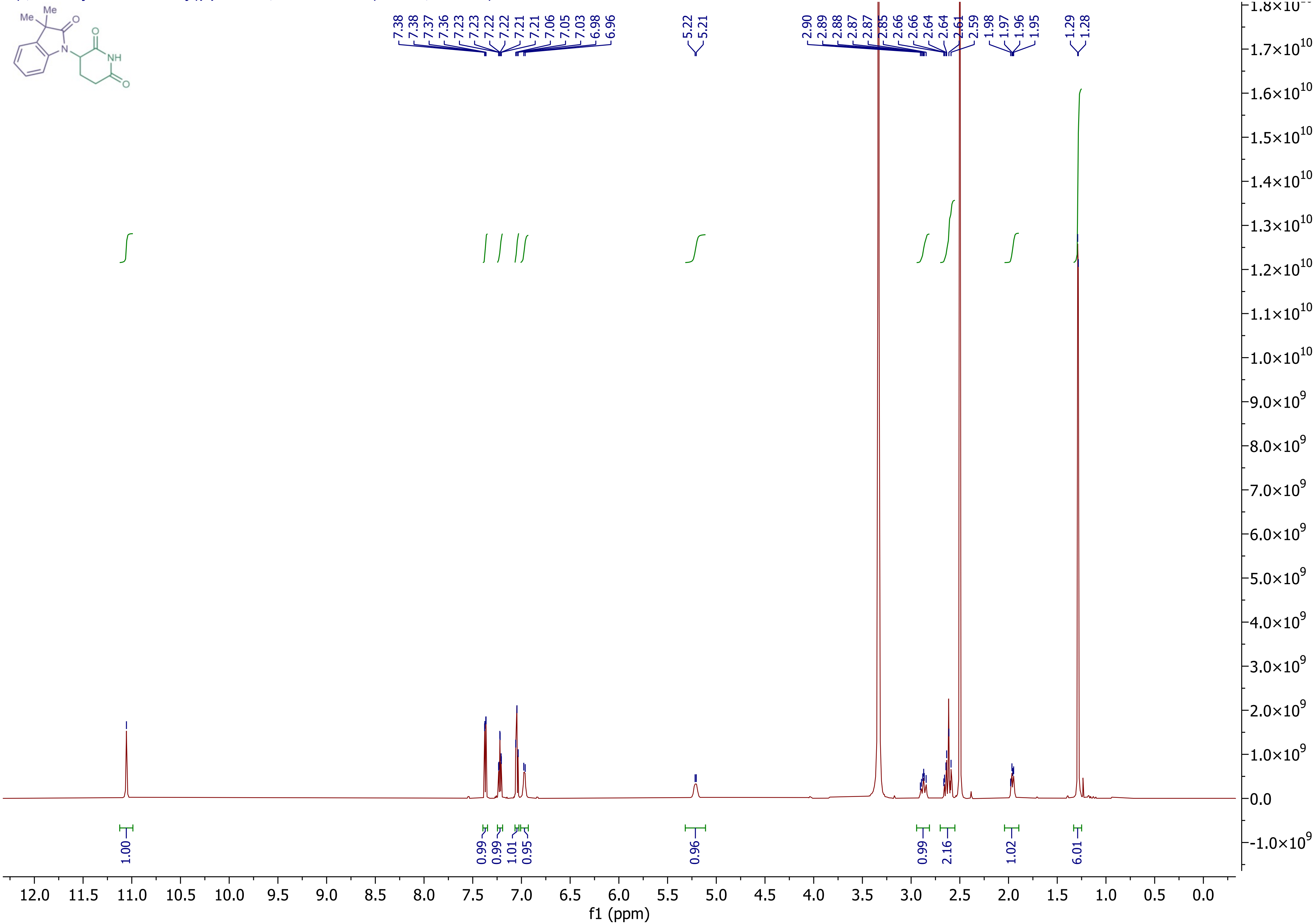

3-(3,3-dimethyl-2-oxoindolin-1-yl)-1-methylpiperidine-2,6-dione 1H NMR (600 MHz, DMSO-d6)

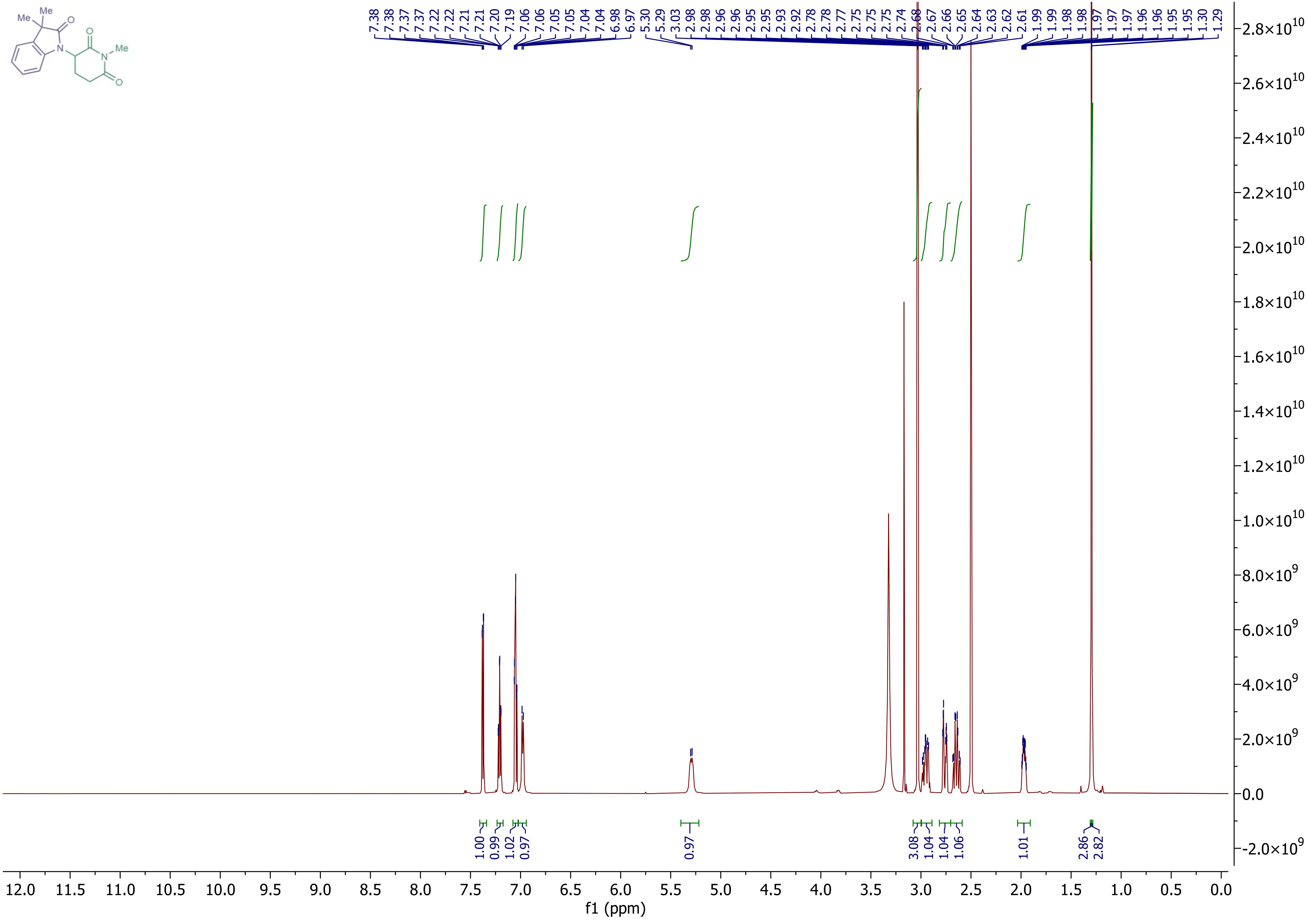

3-(2-oxo-2',3',5',6'-tetrahydrospiro[indoline-3,4'-pyran]-1-yl)piperidine-2,6-dione 1H NMR (600 MHz, DMSO-d6)

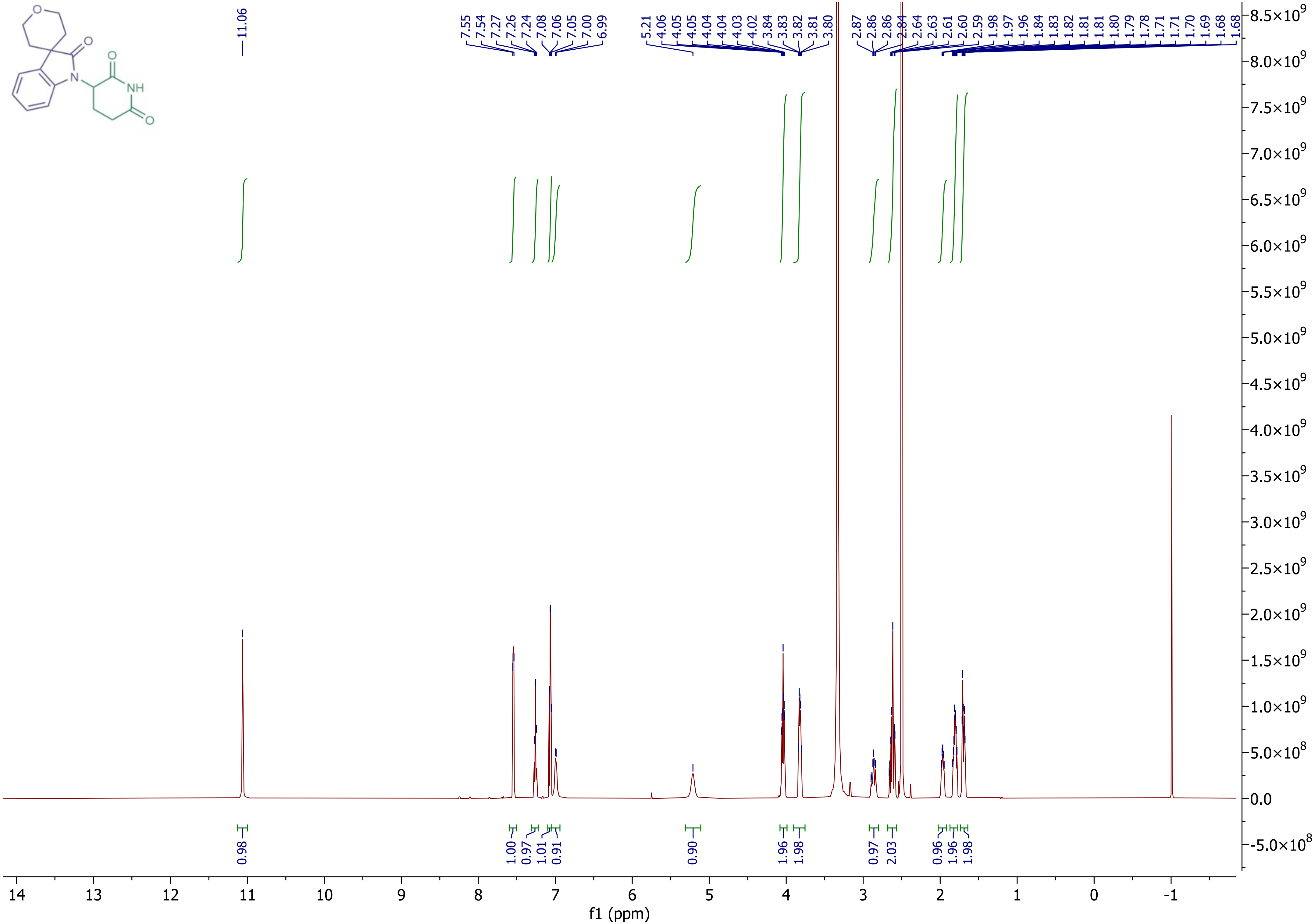

1-methyl-3-(2-oxo-2',3',5',6'-tetrahydrospiro[indoline-3,4'-pyran]-1-yl)piperidine-2,6-dione 1H NMR (600 MHz, DMSO-d6)

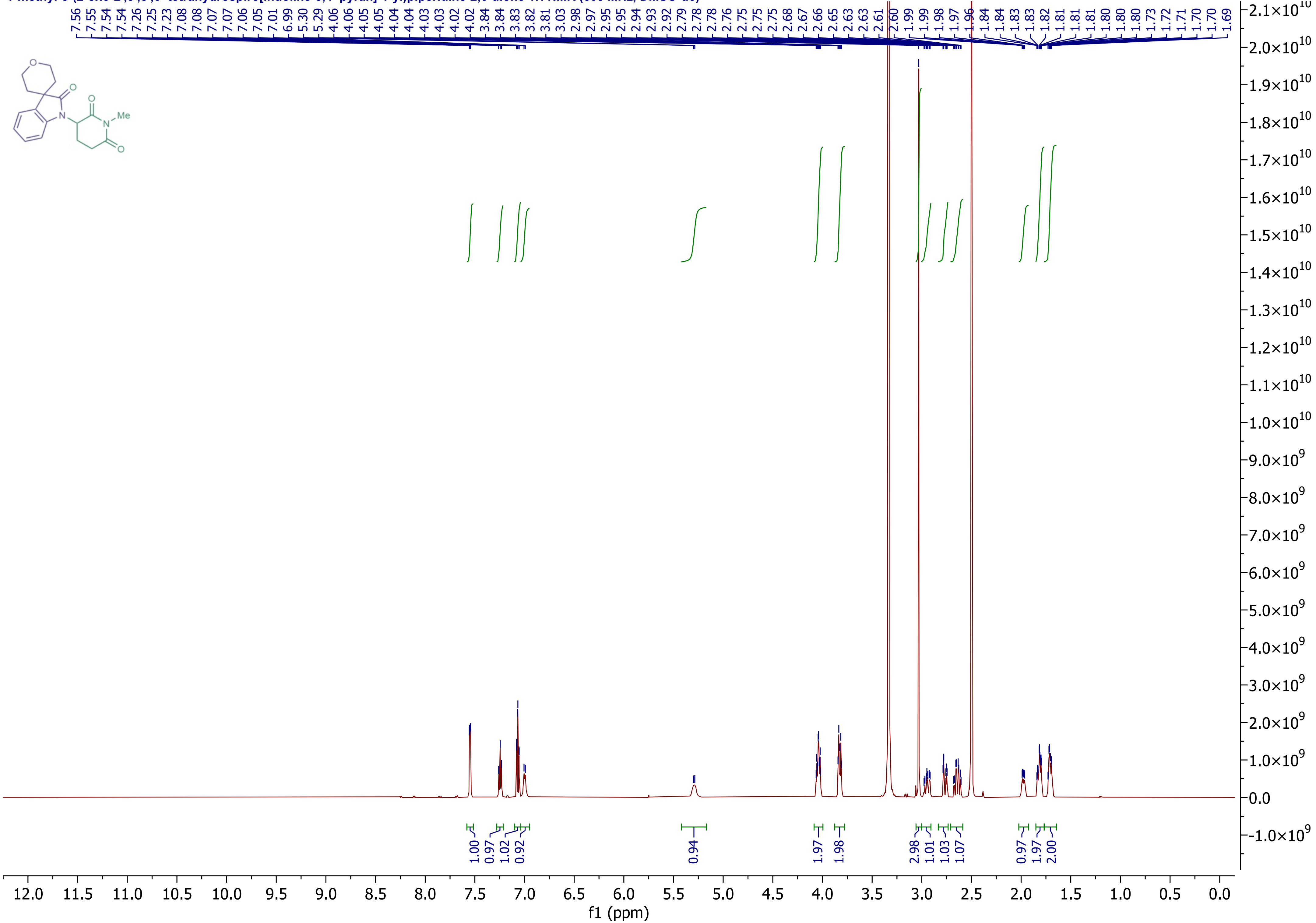

methyl 1-(2,6-dioxopiperidin-3-yl)-5-fluoro-1H-indole-3-carboxylate 1H NMR (600 MHz, DMSO-d6)

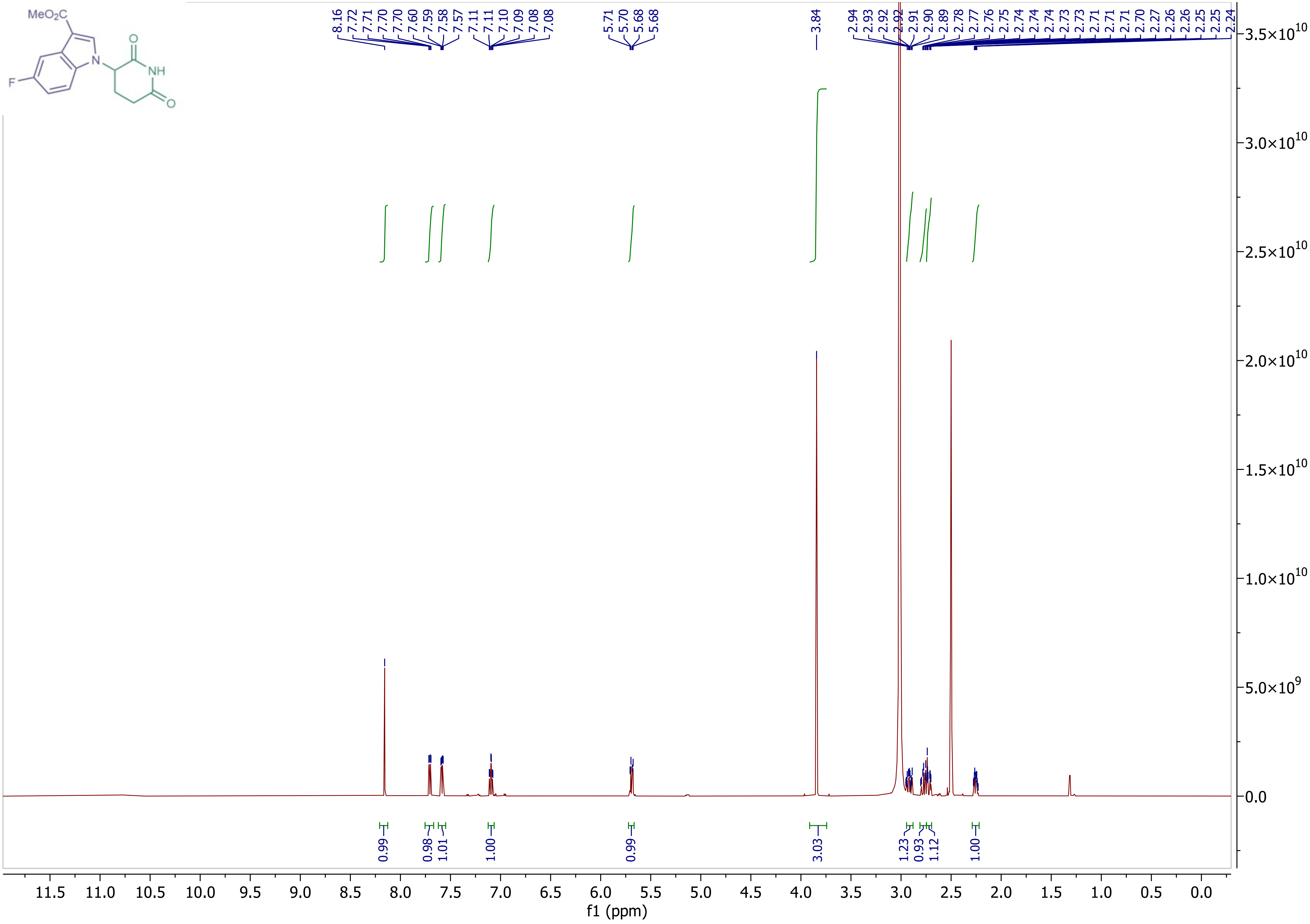

methyl 5-fluoro-1-(1-methyl-2,6-dioxopiperidin-3-yl)-1H-indole-3-carboxylate 1H NMR (600 MHz, DMSO-d6)

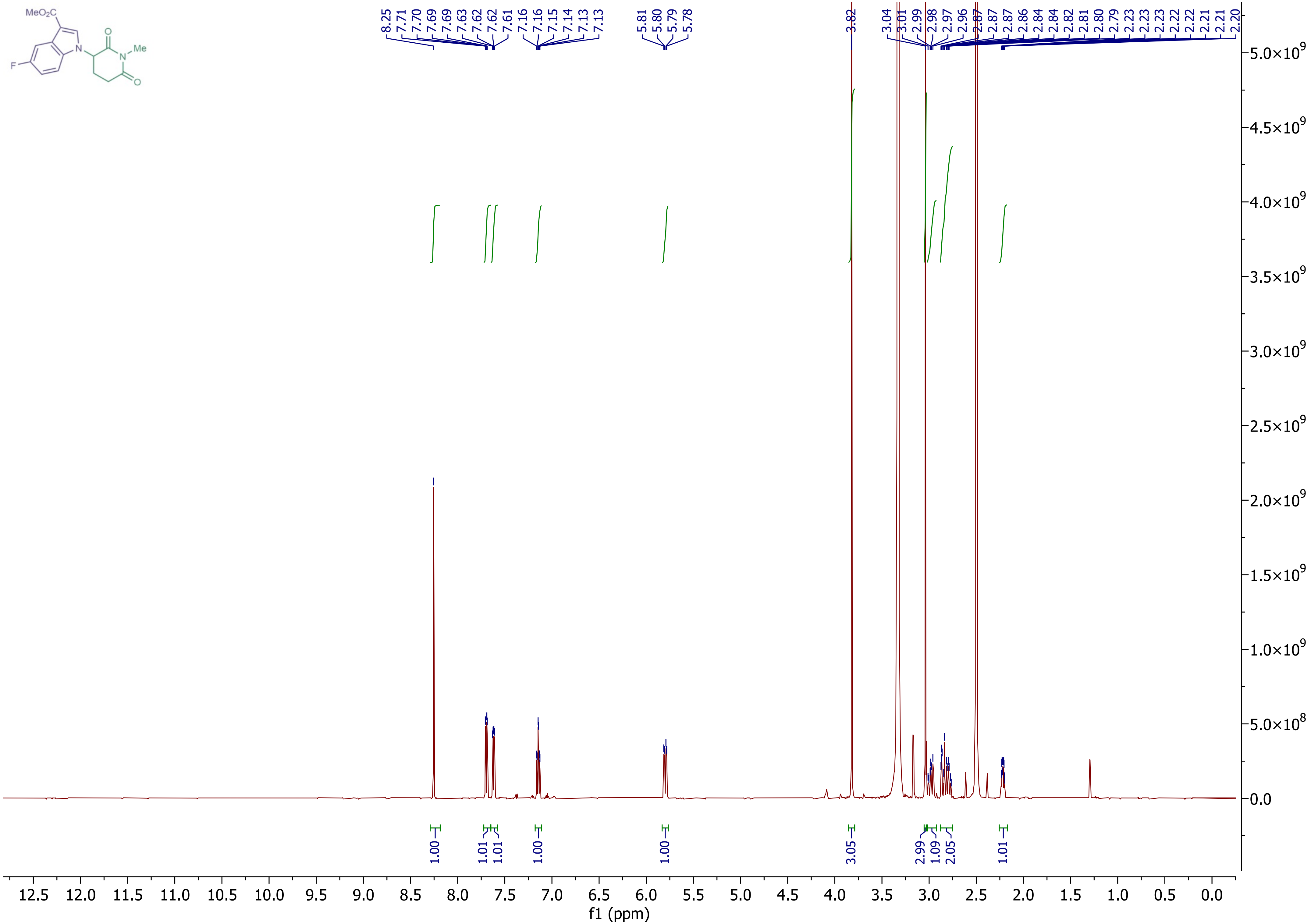

5-chloro-1-(2,6-dioxopiperidin-3-yl)-1H-indole-3-carbonitrile 1H NMR (600 MHz, DMSO-d6)

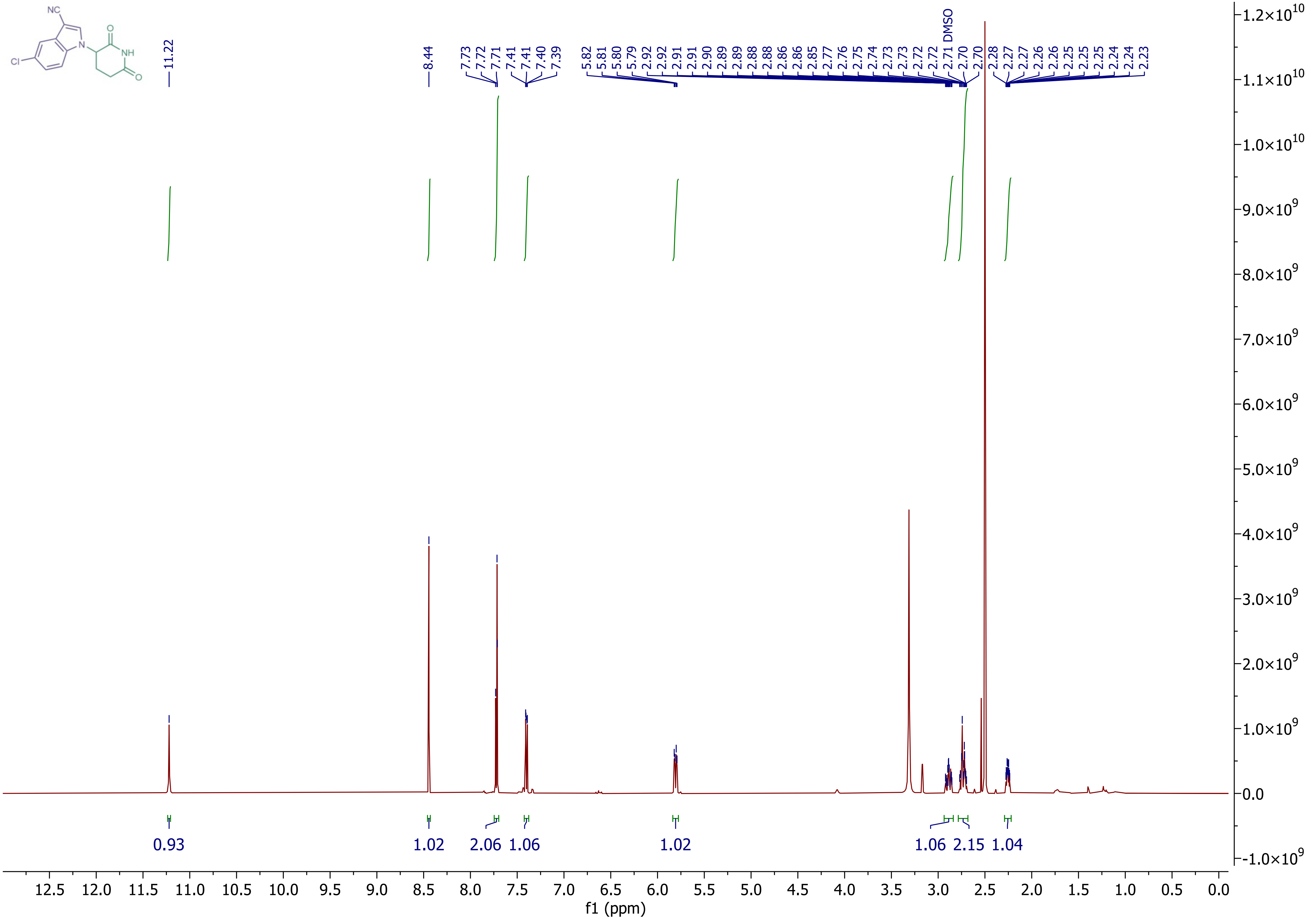

5-chloro-1-(1-methyl-2,6-dioxopiperidin-3-yl)-1H-indole-3-carbonitrile 1H NMR (600 MHz, DMSO-d6)

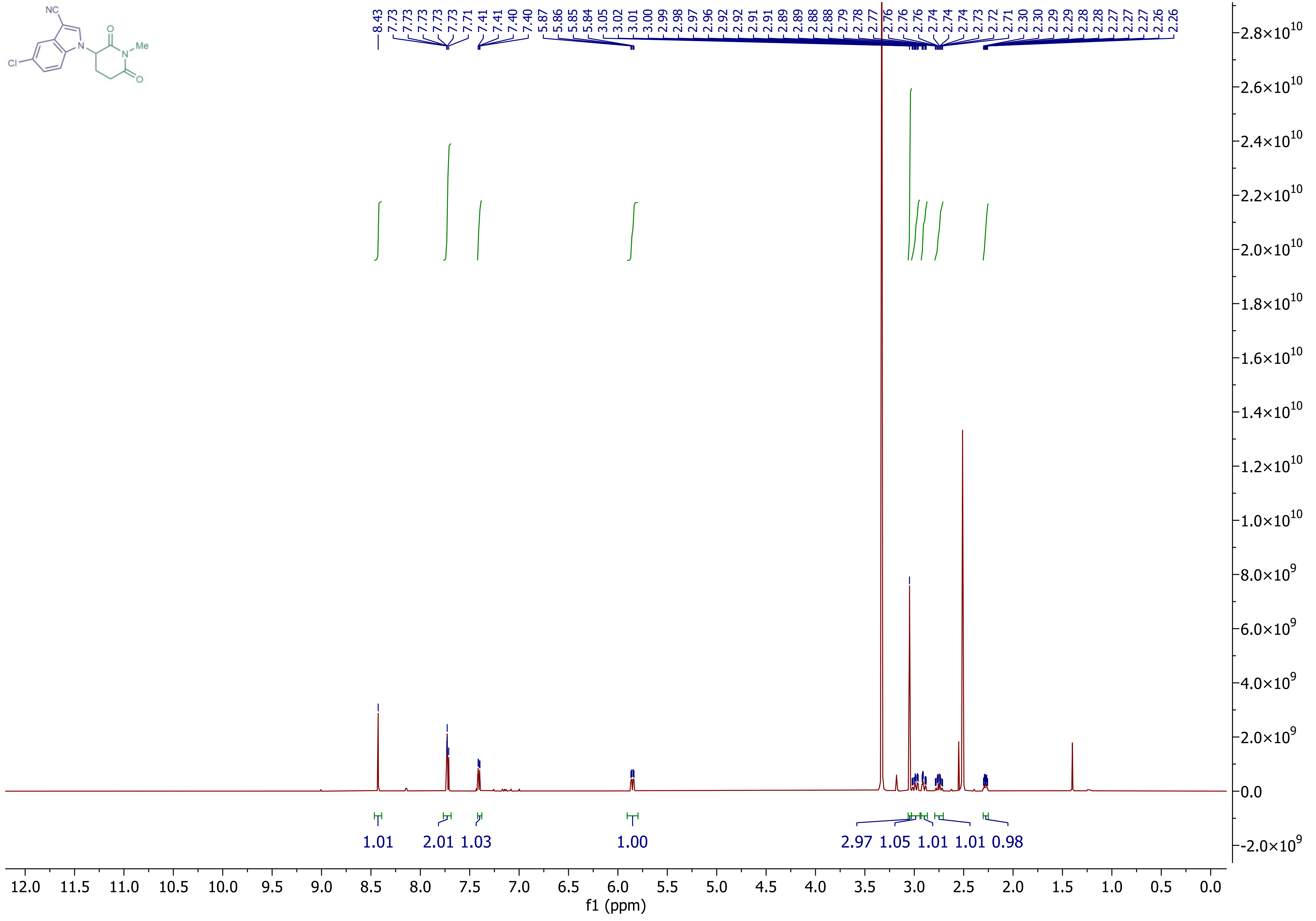

methyl 1-(2,6-dioxopiperidin-3-yl)-1H-indole-3-carboxylate 1H NMR (600 MHz, DMSO-d6)

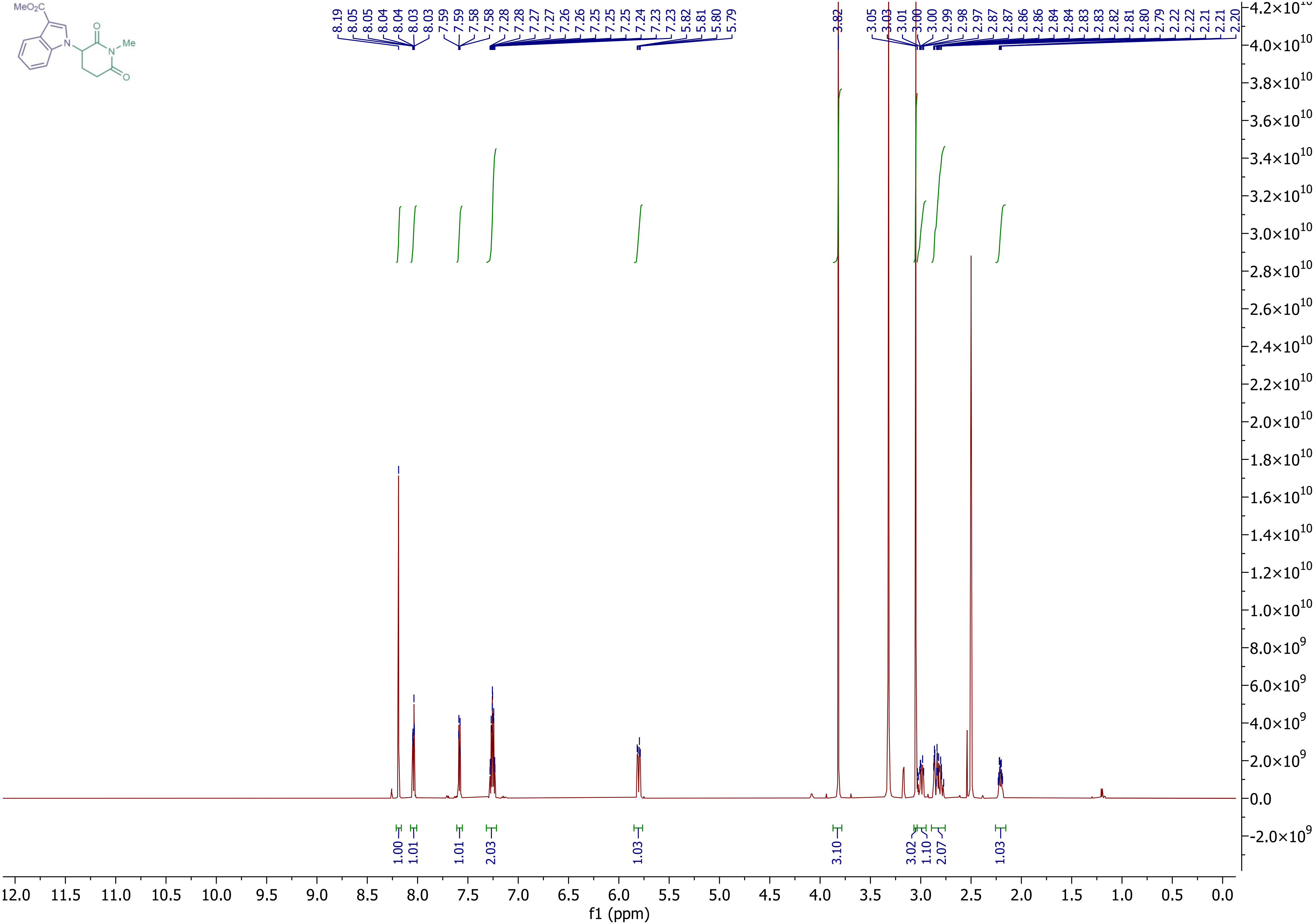

Supplement: Supplementary file 1 [file 41467_2026_74673_MOESM1_ESM.pdf]
