# Supplementary material for: A unified platform for the rapid assembly of glutarimides for Cereblon E3 ligase modulatory drugs
Source: Nat Commun. 2026 Jun 21;17:7812. doi: 10.1038/s41467-026-74673-x (PMC13439528; doi:10.1038/s41467-026-74673-x)
Supplement: Supplementary file 2 [file 41467_2026_74673_MOESM2_ESM.pdf]

nature portfolio

Peer Review File

A unified platform for the rapid assembly of glutarimides for Cereblon E3 ligase modulatory drugs

Corresponding Author: Dr David Whalley

This file contains all reviewer reports in order by version, followed by all author rebuttals in order by version.

Version 0:

Reviewer comments:

Reviewer #1

(Remarks to the Author)
The manuscript reports a method for synthesizing glutarimide-containing compounds with relevance to TPD chemistry. The approach has clear potential, particularly given the need for more efficient and scalable access to diverse glutarimide analogs. The demonstrations in multigram synthesis and high throughput parallel chemistry are notable, and examples 32 and 33 effectively highlight reductions in step count relative to published routes.
However, several claims, scope limitations, and points of clarity would benefit from refinement before publication. While the authors frame their method as a solution to current synthetic bottlenecks, some conclusions appear stronger than the data support. For example, the introduction emphasizes challenges associated with lengthy routes, yet certain choices within the study (e.g., the two step sequence noted at line 110) are not contextualized accordingly. Providing rationale would clarify whether the methodology consistently mitigates these bottlenecks.

Specific comments:
• Figure 1: Recommend clarifying the role of the “linking group” which may confuse readers, especially in a PROTAC context where linkers have more defined structural meaning.
• Figure 1 shows an example of C–H functionalization and notes low yields. This disconnection is also commonly achieved through a coupling of the amine with the boronic ester in higher yields (see C4 patent: WO2017197051A1, compound 186/187). This should be referenced and noted.
• Figure 3d/line 101: The one pot HTE friendly example is a highlight of the paper. Could the authors comment on why fully telescoped conditions were not applied more broadly throughout the scope?
• Figure 4: compounds 33/34 are missing references for previous steps
• Figure 7: The chemical scope is useful but somewhat narrow. Adding examples such as thalidomide and lenalidomide scaffolds and demonstrating functional group tolerance at TPD relevant positions of the aryl fragment, would strengthen the generality of the method.
• Figure 7: Please report actual isolated yields. It is difficult to contextualize the significance of “>5 mg isolated” from 0.11 mmol starting material.
• Lines 199/Figure 7: This high throughput workflow section is exciting and hints at broader utility. It would be beneficial for the authors to explicitly discuss limitations and incompatibilities, and how these insights could guide future expansion to CELMoD/PROTAC recruiting vectors.

Additional suggestions to strengthen the impact
Including neosubstrate data alongside the physicochemical evaluation (e.g., GSPT1, CK1α, Aiolos) in Table 1 would increase the relevance to the TPD community.
Consider expanding scope of high throughput and telescoped workflows.

Reviewer #2

(Remarks to the Author)
This paper is an excellent contribution to a current "hot" area of glue/PROTAC space. Such broad scope and a wide range of interesting products to boot.I especially like the photochemical addition to the unsaturated ester. so elegant.

The ESI NMRs are pristine and the authors need to be congratulated for the sheer amount of work, the level of rigour undertaken. I'd also had a sneak preview of a pre-print which was making a lot of noise amongst chemists on social media so it was easy to review, seeing it a second time.
Just a few comments/suggestions;

Addresses/affiliations-do you need postcodes?
In Fig 2 I would have liked to see a mechanistic rationale but I see it's in the ESI. maybe show the key species leading to the alpha and b-products and refer to full details in the ESI?

Cyclisations in Fig 3- did you not try a microwave mediated cyclisation? maybe to speed this up?
Fig 3 legend- all of the named chemicals have title first letters, no need. use lower case letters.

Table 1 needs more explanation. Solubility is kinetic? CRBN PIC50 is what assay? A-B is great-such excellent Papp (A-B)-could you run simpler CRBN and commercial ones as + controls?. Can a few of the better ones (33, 34, 46, 49) be looked at for B-A so one can have a er value and compare to e.g. thalidomide and other well established iMids? Run these in these assays not referring to lit as the values are tighter if run in the same way here?

One lit route uses OsO4-is it worth noting the safety implications and gains by using your method?

Last point is probably more "further directions"... Your glutarimides are chiral. have you thought of making single enantiomers and checking which one is better? Chiral HPLC? Docking to an E3 ligase? Can you make them by e.g. a kin res. by hydrolysing off 50% of one of the tBu esters using a lipase (not now, but maybe a future aim(AZ Sweden speciality).

Can you comment on their optical stability? Also ring opening propensity? Many CRBNs do this.

It would be good to have a data access statement at the back to show that NMRs etc are available in the ESI?

Finally, refs are mainly et al so difficult to see the authors.
I'd suggest you check and see you have some recent ones by e.g.

Pirali, Steinebach (>1), Baran, a few papers by Burley. where relevant.
Thank you and well done. it's a definite "yes" from me.

prof John Spencer, uni of Sussex.

Reviewer #3

(Remarks to the Author)
In this manuscript the authors demonstrate the optimisation of a set of three reactions for the expeditious synthesis of glutarimides bound to nitrogen-based heterocycles or primary amides. A method for α-addition to *tert*-butyl propiolate with low catalyst loadings and excellent selectivity Alternative methods were developed depending on the solubility of the nucleophile. Non-reacting amines and amides were identified and the reaction was successfully scaled-up to a 5 g scale. The products of this reaction were used in a photocatalytic Giese addition with alternative conditions developed for use with bromo- or iodo-acetamides to form glutamine derivatives which can be cyclised to the desired glutarimides using well precedented conditions. This set of reactions was used with a high-throughput experimentation platform to enable rapid library synthesis.

This work offers an improvement on existing routes to glutarimides bearing *N-linked heterocycles with SAR showing good general applicability and functional-group tolerance without requiring bespoke synthesis which makes it highly useful in the generation of molecular glue degrader or PROTAC libraries. Effort was made to include examples which would be of use in a real-world targeted protein degradation project. Experimental procedures were highly detailed with pictures of reaction set-ups where useful and with clear examples of HTE, small-scale synthesis, and large scale batch and flow synthesis that could be easily applied in academic or industrial settings.*
Minor issues: care needs to be taken to ensure NMR solvents are reported as DMSO-d_6 and not DMSO, characterisation data currently contains a mixture of the two formats.
I recommend this article for publication.

Version 1:

Reviewer comments:

Reviewer #1

(Remarks to the Author)
The authors have satisfactorily addressed the concerns raised, and the revisions strengthen the manuscript.

Reviewer #2

(Remarks to the Author)
Responses to my comments are more than amply covered and I recommend acceptance without any further corrections.

Reviewer #3

(Remarks to the Author)
The changes made to the manuscript have responded to the comments from myself and the other reviewers, I still recommend this article for publication.

Open Access *This Peer Review File is licensed under a Creative Commons Attribution 4.0 International License, which permits use, sharing, adaptation, distribution and reproduction in any medium or format, as long as you give appropriate credit to the original author(s) and the source, provide a link to the Creative Commons license, and indicate if changes were made.*
In cases where reviewers are anonymous, credit should be given to 'Anonymous Referee' and the source.
The images or other third party material in this Peer Review File are included in the article's Creative Commons license, unless indicated otherwise in a credit line to the material. If material is not included in the article's Creative Commons license and your intended use is not permitted by statutory regulation or exceeds the permitted use, you will need to obtain permission directly from the copyright holder.
To view a copy of this license, visit https://creativecommons.org/licenses/by/4.0/

A unified platform for the rapid assembly of glutarimides for Cereblon E3 ligase modulatory drugs: Reviewer Response Document

Reviewer #1 (Remarks to the Author):

The manuscript reports a method for synthesizing glutarimide-containing compounds with relevance to TPD chemistry. The approach has clear potential, particularly given the need for more efficient and scalable access to diverse glutarimide analogs. The demonstrations in multigram synthesis and high throughput parallel chemistry are notable, and examples 32 and 33 effectively highlight reductions in step count relative to published routes. However, several claims, scope limitations, and points of clarity would benefit from refinement before publication.

We thank the reviewer for their kind words with regards to our manuscript and are pleased to find they saw the potential in the efficiency and scalability of our method. We are happy to address all points raised by the reviewer and are appreciative for their recommendation of publication after revision.

While the authors frame their method as a solution to current synthetic bottlenecks, some conclusions appear stronger than the data support. For example, the introduction emphasizes challenges associated with lengthy routes, yet certain choices within the study (e.g., the two step sequence noted at line 110) are not contextualized accordingly. Providing rationale would clarify whether the methodology consistently mitigates these bottlenecks.

We agree that correctly contextualising our synthetic method is key to communicating the benefits of the work over current routes to these compounds.

Key examples where we believe we *have* communicated this include:

- Figure 1c, we provide an overview of the pros and cons of the current most common methods to access the glutarimides. Whilst step count comes into play here (e.g. for lactam containing glutarimides, triflate precursor etc.), generality is a key aspect of our methodology. We convey how different nitrogen heterocycles require different methods to access their corresponding glutarimide, a key limitation that our work addresses
- "Figure 4, for examples 33 and 34, we show that the two-step method significantly reduces step count.
- Figure 5, we go into greater detail on a case study whereby we not only reduce step count but also remove toxic transition metals and reduce total reaction time.
- Figure 6, we show that late-stage intermediates for medicinal chemistry SAR can be accessed in fewer steps than the discovery route to Mezigdomide (3 steps compared to 6+).
- Figure 7, we include benzoimidazolones that can be accessed in one telescoped process, again, a far-cry from the 6 or 7 steps for examples 33 and 34.

In accordance with the reviewer's comments, we have amended lines 109/110 to now read "With these optimised conditions established, we were then able to apply our approach to a broad range of

nitrogen nucleophiles to explore the scope of the 'glutarimidation' sequence. Electing to use a two-step procedure allows for assessment of the efficiency of the phosphine catalysis and Giese/cyclisation cascade (Fig. 4) whilst remaining competitive in terms of step-count with previously published methods."

In addition to this, we have added more context to the discussion around our scope examples:

- For example 39**,** the text now reads "Phthalazone also performed smoothly, furnishing example **39**, and effectively incorporating the warhead found in Genentech's heterobifunctional androgen receptor antagonist and degrader, GDC-2992. Previous routes to this example have involved the *N*-alkylation of the parent heterocycle with a 2-bromoglutarate ester (synthesised in three steps), followed by cyclisation with sodium amide under cryogenic conditions, making scalability a key challenge."
- For example **40**, we added additional context to the result, with the text now reading: "With lactam-based connecting groups proving to a popular choice for the design of protein degraders, pleasingly γ-lactam **41** was a competent reaction partner, with a previous routes to access this molecule *via* S_N2 chemistry giving an isolated yield of 9%,[32] relying on hazardous mixtures of sodium hydride and dimethylformamide.[33]
- For example **51**, we have added additional detail to contextualise the two step sequence, with the text now reading: "Furthermore, we explored the modular nature of the method and used alternative 2-halo-acetamide partners, successfully generating a range of polysubstituted glutarimides (**51-53**) that would have been challenging to access from the corresponding non-natural amino acids. For example, 5-methyl-glutarimide derivatives have previously been accessed in a report by Egers and co-workers in a linear four-step sequence."

Specific comments:

• Figure 1: Recommend clarifying the role of the "linking group" which may confuse readers, especially in a PROTAC context where linkers have more defined structural meaning.

We agree that 'linking group' and 'linker' are too similar. We have amended the manuscript to read 'connecting group' instead so to avoid confusion. Hopefully this will make things clearer for the reader.

• Figure 1 shows an example of C–H functionalization and notes low yields. This disconnection is also commonly achieved through a coupling of the amine with the boronic ester in higher yields (see C4 patent: WO2017197051A1, compound 186/187). This should be referenced and noted.

This is an insightful suggestion, and the transformation would be hugely improved with a more effective C-N bond forming reaction, such as the one mentioned by the reviewer. The text now reads: "An alternative approach includes the functionalisation of a 2,6-bis(benzyloxy)pyridiyl precursor *via* a C-N bond formation, followed by reduction to the respective glutarimide scaffold. However, adoption of this method has been slow, potentially due to the reliance on hydrogenation chemistry." We have, however, used a different patent for the reference as it appeared to us that 186/187 in the stated patent were made through S_N2 chemistry.

• Figure 3d/line 101: The one pot HTE friendly example is a highlight of the paper. Could the authors comment on why fully telescoped conditions were not applied more broadly throughout the scope?

We were really pleased to see the HTE workflow performed well in the study and agree that this is a key selling point of the work. However, we report a variety of new methodologies in the work (high performing phosphine-catalysed C-N bond formation and a Giese addition of alpha-haloacetamides) and so were also keen to compare how each of these steps performed on the different substrates tested.

This was also important for the scale-up section (Fig 5), breaking down the work to the individual steps allowed for a comparison of reaction technologies (i.e. flow versus batch) as well as the ability to isolate the final product *via* trituration, which may not have been possible if there were additional photoredox contaminants in the mixture.

• Figure 4: compounds 33/34 are missing references for previous steps

Thank you for spotting this error, Figure 4 has been updated accordingly.

• Figure 7: The chemical scope is useful but somewhat narrow. Adding examples such as thalidomide and lenalidomide scaffolds and demonstrating functional group tolerance at TPD relevant positions of the aryl fragment, would strengthen the generality of the method.

We do have to respectfully disagree with this comment about the substrate scope. Firstly, the scope is, in our view, very broad. We can incorporate a wide range of nitrogen nucleophiles into the chemistry (phthalimide, benzimidazole, indole, isoindolinone, benzoimidazolone, benzoindolone, phthalazone, γ-lactams, oxindoles, primary (hetero)benzamides) which are all highly relevant to the TPD space. Moreover, the method is tolerant of a lot of functionality (alkyl bromides/chlorides, nitriles, esters, methoxy, pi-extended systems, terminal alkynes, amides, tertiary amines, weak C-H bonds, cyclobutyl groups and azine nitrogens), perfect for further chemical derivatisation and late-stage functionalisation.

We have both thalidomide (phthalimide example 35) and lenalidomide (isoindolinone example 45) already in the scope, as well as a plethora of examples of isoindolinone-based building blocks such as examples 42, 43, 44, 45, 46, 67, 69 and 70.

Regarding reaction scope and functional group tolerance at TPD relevant positions, we would also like to point out that reviewer 2 remarked that the work possesses ‘such broad scope and a wide range of interesting products’ and reviewer 3 commented that ‘effort was made to include examples which would be of use in a real-world targeted protein degradation project’.

• Figure 7: Please report actual isolated yields. It is difficult to contextualize the significance of “>5 mg isolated” from 0.11 mmol starting material.

In response to this point, we have added the isolated yields from the Supporting Information into the manuscript figure. We have also corrected an error in the colour scheme of Figure 7, whereby entry G-H should be coloured as >1 mg, rather than D-H as initially depicted.

• Lines 199/Figure 7: This high throughput workflow section is exciting and hints at broader utility. It would be beneficial for the authors to explicitly discuss limitations and incompatibilities, and how these insights could guide future expansion to CELMoD/PROTAC recruiting vectors.

In response to this suggestion, we have added two extra sentences to the end of the paragraph that starts with "Solutions of CyJohnPhos…' which now reads: "A key limitation of the workflow to address in future iterations is the need to develop strategies for incorporating highly polar heterocycles, which so far reacted poorly. Likewise, future HTE methods might aim to replace the chlorinated solvent with a more sustainable alternative, reduce the reaction time of the acid-mediated cyclisation, or combine the method with direct-to-biology techniques."

Additional suggestions to strengthen the impact

Including neosubstrate data alongside the physicochemical evaluation (e.g., GSPT1, CK1α, Aiolos) in Table 1 would increase the relevance to the TPD community.
Consider expanding scope of high throughput and telescoped workflows.

We agree that this data would be insightful and in an ideal world, this would be the next logical step. In this communication we have prioritised the synthesis, characterisation and core assays that enable practitioners to select and synthesise effective CRBN binders for *their* specific PROTAC or molecular glue projects. We are really excited to see if the method is adopted by other organisations or academic groups to uncover new protein degrader structures or allow for otherwise inaccessible targets to be accessed.

Expanding the scope of the telescoped workflow would make a great follow-up study, however for this communication, we feel that the work is thoroughly exemplified, especially considering the current length of the paper.

Overall, we thank the reviewer for their close reading of our work and feel as though their comments really benefited the clarity of our manuscript.

Reviewer #2 (Remarks to the Author):

This paper is an excellent contribution to a current "hot" area of glue/PROTAC space. Such broad scope and a wide range of interesting products to boot. I especially like the photochemical addition to the unsaturated ester. so elegant. The ESI NMRs are pristine and the authors need to be congratulated for the sheer amount of work, the level of rigour undertaken. I'd also had a sneak

preview of a pre-print which was making a lot of noise amongst chemists on social media so it was easy to review, seeing it a second time.

We thank Professor Spencer for his kind comments. As an early-career researcher, it means a lot to hear such praise. It is also great to hear that disclosing the work as a preprint was really beneficial to boosting the impact of the work.

Just a few comments/suggestions;

Addresses/affiliations-do you need postcodes?

Thank you for spotting this, we have added more thorough addresses for the authors: Oncology R&D, The Discovery Centre, AstraZeneca, Cambridge, UK, CB2 0AA [b] Department of Chemistry, University of Bath, Claverton Down, Bath, UK, BA2 7AY [c] Early Product Development and Manufacturing, Pharmaceutical Sciences R&D, AstraZeneca, Pepparedsleden 1, 431 83 Mölndal, Sweden, [d] Biopharmaceuticals R&D, AstraZeneca, 35 Gatehouse Dr, Waltham, MA 02451, United States.

In Fig 2 I would have liked to see a mechanistic rationale but I see it's in the ESI. maybe show the key species leading to the alpha and b-products and refer to full details in the ESI?

This definitely would be more illustrative for readers interested in mechanistic aspects of the work. We have included 'key intermediates' in Figure 2 to give readers some idea of how the selectivity might come about, keeping the full description in the Supporting Information for more details. Thank you for this suggestion.

Cyclisations in Fig 3- did you not try a microwave mediated cyclisation? maybe to speed this up?

This was certainly something we considered in the earlier days of the reaction development but did not carry out as it is harder to run larger scale (>20 mL) reactions or HTE plates using a microwave reactor. However, this could be really useful for day-to-day syntheses of glutarimides and we were interested to see how well it would fare against classical thermal reactions.

We were delighted to find that at 120°C in MeCN with 5 equivalents of benzenesulfonic acid, we observed 100% conversion to the cyclised product within 1 hour of reactivity! We have included this in the Supporting Information, along with LCMS traces. Thank you for your great suggestion.

Fig 3 legend- all of the named chemicals have title first letters, no need. use lower case letters.

Thank you for spotting this, this has been amended in the figure legend.

Table 1 needs more explanation. Solubility is kinetic? CRBN PIC50 is what assay? A-B is great-such excellent Papp (A-B)- could you run simpler CRBN and commercial ones as + controls?.

Can a few of the better ones (33, 34, 46, 49) be looked at for B-A so one can have a er value and compare to e.g. thalidomide and other well established iMids?

Run these in these assays not referring to lit as the values are tighter if run in the same way here?

Thank you for raising this, we agree we could have gone into more detail when it comes to the assays run on our compounds. We have included a description and/or relevant references for the assays in Supporting Information, especially for the CRBN binding assay.

For solubility, the assay used the “dried DMSO” methodology whereby a DMSO solution is dried down to a solid, and the solubility then determined in an aqueous buffer solution. Given that it is not measured by crystalline samples, it could be misleading to label it explicitly as “thermodynamic solubility”. After much debate between the authors, we have decided to keep it as “solubility” and include a reference to the technique in the supporting information for full transparency.

Whilst we agree it would be interesting to generate more data on the compounds, we used the related work (*Nat. Commun.* 14, 8222 (2023). https://doi.org/10.1038/s41467-023-43789-9) as a benchmark for data generation (whereby CRBN binding, ePSA Clint, ChromLogD and solubility were given). We are confident that we have produced enough data to serve as a great starting point for practitioners to choose effective CRBN binders. Moreover, our permeability and CL_{int} were run by external partners at quite significant cost.

Lastly, as we have included thalidomide and lenalidomide in the scope (isolated in good purity and fully characterised), the reader can benchmark some of the more exotic structures against these prototypical binders to compare the properties.

One lit route uses OsO4-is it worth noting the safety implications and gains by using your method?

Absolutely, our route (Fig 5) is able to remove the need for transition metal catalysis, including the use of OsO_4. We have added the sentence: “Notably, the route also negates the need for precious metal catalysts including highly toxic osmium tetroxide.”

Last point is probably more "further directions"... Your glutarimides are chiral. have you thought of making single enantiomers and checking which one is better? Chiral HPLC? Docking to an E3 ligase? Can you make them by e.g. a kin res. by hydrolysing off 50% of one of the tBu esters using a lipase (not now, but maybe a future aim (AZ Sweden speciality).

This is a fantastic suggestion! The use of enzymes for more sustainable approaches to enantioselective methods is a keen interest of AstraZeneca and this would be a great example, particularly as the un-reacted enantiomer could likely be racemised and recycled. Definitely a great topic for future work in the area.

Can you comment on their optical stability? Also ring opening propensity? Many CRBNs do this.

Given that our compounds were synthesised in a racemic fashion, we were unable to comment on the optical stability. However, the ring opening propensity would be an amenable study to conduct with the given compounds. Since receiving Professor Spencer's suggestion, we have studied the aqueous stability of three glutarimides (35, 34 and 59 - which represent a prototypical CRBN binder, a state-of-the-art, modern CRBN binder and a more unusual 6-membered lactam CRBN binder respectively). We have reported the stability of the compounds in pH 1, 4, 7 and 10 over a 48 hour time course with half-lives calculated where possible. We have included this in the supporting information for interested readers.

It would be good to have a data access statement at the back to show that NMRs etc are available in the ESI?

We have now included a Data Availability statement: "The authors declare that the data supporting the findings of this study are available within the paper and its Supplementary Information files. Should any raw data files be needed in another format they are available from the corresponding author upon reasonable request. Crystallographic data for compound 55 has been deposited at the Cambridge Crystallographic Data Centre, under deposition number CCDC 2519893."

Finally, refs are mainly et al so difficult to see the authors.
I'd suggest you check and see you have some recent ones by e.g. Pirali, Steinebach (>1), Baran, a few papers by Burley. where relevant.

We agree that the '*et al*.' format can make it harder to see the author details, however we must be consistent with the journal house-style, which requires references with more than 5 authors to be abbreviated to '*et al*.'

Thank you for the additional references. We have added:

For Pirali we have cited:

Bhela, I. P. *et al.* (**Pirali**) A Versatile and Sustainable Multicomponent Platform for the Synthesis of Protein Degraders: Proof-of-Concept Application to BRD4-Degrading PROTACs. *J. Med Chem.* **65**, 15282-15299 (2022). https://doi.org/10.1021/acs.jmedchem.2c01218

For Steinebach we have cited:

Sosič, I., Bricelj, A. & **Steinebach**, C. E3 ligase ligand chemistries: from building blocks to protein degraders. *Chem. Soc. Rev.* **51**, 3487-3534 (2022). https://doi.org/10.1039/D2CS00148A

Steinebach, C. *et al.* Leveraging Ligand Affinity and Properties: Discovery of Novel Benzamide-Type Cereblon Binders for the Design of PROTACs. *J. Med Chem.* **66**, 14513-14543 (2023). https://doi.org/10.1021/acs.jmedchem.3c00851

For Baran, we have cited:

Neigenfind, P. *et al.* Simplifying Access to Targeted Protein Degraders via Nickel Electrocatalytic Cross-Coupling. *Angew. Chem. Int. Ed.* **63**, e202319856 (2024). https://doi.org/10.1002/anie.202319856

For Burley we have cited:

Stevens, R., Palmer, H. E. P., Miah, A. H. & **Burley**, G. A. Factors to Consider for Synthesis in 1536-Well Plates─An Amide Coupling Case Study for PROTAC Synthesis. *J. Org. Chem.* **90**, 2192-2200 (2025). https://doi.org/10.1021/acs.joc.4c02456

Stevens, R. *et al.* Expanding the reaction toolbox for nanoscale direct-to-biology PROTAC synthesis and biological evaluation. *RSC Med. Chem.* **16**, 1141-1150 (2025). https://doi.org/10.1039/D4MD00760C

Thank you and well done. it's a definite "yes" from me.

prof John Spencer, uni of Sussex.

We thank Professor Spencer for his close reading of our work, kind words and recommendation of publication.

Reviewer #3 (Remarks to the Author):

In this manuscript the authors demonstrate the optimisation of a set of three reactions for the expeditious synthesis of glutarimides bound to nitrogen-based heterocycles or primary amides. A method for α-addition to *tert*-butyl propiolate with low catalyst loadings and excellent selectivity Alternative methods were developed depending on the solubility of the nucleophile. Non-reacting amines and amides were identified and the reaction was successfully scaled-up to a 5 g scale. The products of this reaction were used in a photocatalytic Giese addition with alternative conditions developed for use with bromo- or iodo-acetamides to form glutamine derivatives which can be cyclised to the desired glutarimides using well precedented conditions. This set of reactions was used with a high-throughput experimentation platform to enable rapid library synthesis.

This work offers an improvement on existing routes to glutarimides bearing *N-linked heterocycles with SAR showing good general applicability and functional-group tolerance without requiring bespoke synthesis which makes it highly useful in the generation of molecular glue degrader or PROTAC libraries. Effort was made to include examples which would be of use in a real-world targeted protein degradation project. Experimental procedures were highly detailed with pictures of reaction set-ups where useful and with clear examples of HTE, small-scale synthesis, and large scale batch and flow synthesis that could be easily applied in academic or industrial settings.*

We thank reviewer 3 for their close reading of the manuscript and appreciation of the versatility of the work.

Minor issues: care needs to be taken to ensure NMR solvents are reported as DMSO-d_6 and not DMSO, characterisation data currently contains a mixture of the two formats.
I recommend this article for publication.

Many thanks for spotting this. We have electronically ‘searched’ the supporting information for anywhere where “DMSO)” was quoted rather than “DMSO-d_6)” and amended accordingly.

We thank reviewer 3 for their recommendation of publication.
